# Small Changes in pH Have Direct Effects on Marine Bacterial Community Composition: A Microcosm Approach

**DOI:** 10.1371/journal.pone.0047035

**Published:** 2012-10-11

**Authors:** Evamaria Krause, Antje Wichels, Luis Giménez, Mirko Lunau, Markus B. Schilhabel, Gunnar Gerdts

**Affiliations:** 1 Alfred Wegener Institute for Polar and Marine Research, Biologische Anstalt Helgoland, Helgoland, Germany; 2 School of Ocean Sciences, Bangor University, Menai Bridge, Wales, UK; 3 Leibniz Institute for Baltic Sea Research, Rostock, Germany; 4 Institute of Clinical Molecular Biology, Christian-Albrechts-University Kiel, Kiel, Germany; University of Delaware, United States of America

## Abstract

As the atmospheric CO_2_ concentration rises, more CO_2_ will dissolve in the oceans, leading to a reduction in pH. Effects of ocean acidification on bacterial communities have mainly been studied in biologically complex systems, in which indirect effects, mediated through food web interactions, come into play. These approaches come close to nature but suffer from low replication and neglect seasonality. To comprehensively investigate direct pH effects, we conducted highly-replicated laboratory acidification experiments with the natural bacterial community from Helgoland Roads (North Sea). Seasonal variability was accounted for by repeating the experiment four times (spring, summer, autumn, winter). Three dilution approaches were used to select for different ecological strategies, i.e. fast-growing or low-nutrient adapted bacteria. The pH levels investigated were *in situ* seawater pH (8.15–8.22), pH 7.82 and pH 7.67, representing the present-day situation and two acidification scenarios projected for the North Sea for the year 2100. In all seasons, both automated ribosomal intergenic spacer analysis and 16S ribosomal amplicon pyrosequencing revealed pH-dependent community shifts for two of the dilution approaches. Bacteria susceptible to changes in pH were different members of *Gammaproteobacteria*, *Flavobacteriaceae*, *Rhodobacteraceae*, *Campylobacteraceae* and further less abundant groups. Their specific response to reduced pH was often context-dependent. Bacterial abundance was not influenced by pH. Our findings suggest that already moderate changes in pH have the potential to cause compositional shifts, depending on the community assembly and environmental factors. By identifying pH-susceptible groups, this study provides insights for more directed, in-depth community analyses in large-scale and long-term experiments.

## Introduction

Since the beginning of the industrial period, the oceans have taken up one-quarter to one-third of anthropogenic CO_2_ emissions [1], [2]. This has already led to a reduction in surface ocean pH of 0.1 units, which may reach up to 0.7 units assuming the depletion of all fossil fuel reserves during the next three centuries [3]. In contrast, pH has constantly remained above 8.1 for the last 23 million years [4]. By the year 2100, atmospheric pCO_2_ values of 700 or 1000 ppm may lower mean surface pH in the southern North Sea to 7.82 or 7.67, respectively [5].

The effects of the anticipated rapid reduction in pH on marine organisms, and their ability to adapt, will determine future marine biodiversity and ecosystem functions. Yet the impact of ocean acidification on different groups of marine organisms remains under debate [6], [7], especially regarding heterotrophic bacteria as important players in marine biogeochemical cycles. Joint et al. [8] recently argued that microbe-dependent processes will not substantially change in a more acidic ocean, as marine microbes already experience large regional, temporal and depth-dependent pH variability, and even greater pH ranges are observed in freshwater lakes. This view was challenged by a meta-analysis on microbe-related ocean acidification research, which identified nitrogen fixation, cyanobacterial photosynthesis and elemental ratios as affected by changes in seawater carbonate chemistry [9]. Concerning other microbial processes and especially heterotrophic bacteria however, results have often been inconsistent and Liu et al. [9] concluded that “more research is needed at multi-species and community scales”.

What we know about ocean acidification effects on bacterial communities predominantly stems from complex systems such as symbiotic microbial communities of corals or large-scale mesocosm experiments. At reduced pH, coral microbial communities were found to shift to bacteria associated with stressed or diseased hosts [10], [11], which could however not be confirmed at natural CO_2_ vent sites [12]. Furthermore, a decrease in the relative abundance of *Alphaproteobacteria* and an increase in the relative abundance of *Flavobacteriales* were observed in natural biofilms from the Great Barrier Reef [13].

Knowledge on the seawater bacterial community has remained scarce though. In mesocosm experiments, only minor indications of bacterial community shifts with pH were found [14], [15]. Notably, these findings relied on only one or two replicates per pCO_2_ treatment, which is a common problem in mesocosm studies. Although these experiments are biologically highly complex, involving indirect pH effects through food web interactions, they are usually carried out in low replication, due to logistical challenges and high costs. As a consequence, these experimental designs preclude a robust statistical interpretation. Furthermore, the natural variability of bacterial communities, which is characterized by seasonally recurring patterns [16]–[18], is not taken into account.

Therefore, a straightforward small-scale approach is needed to allow for high replication and the consideration of differently assembled communities. The problem of limited culturability of marine bacteria can be addressed by dilution experiments. A certain fraction of natural bacterioplankton is able to grow in particle-free, unenriched seawater [19], although the confinement to experimental containers causes shifts in community structure often described as the “bottle effect” [20]. To alleviate this bias and select for typical marine bacteria, the concept of dilution culture was developed [21]. Originally, this approach followed a dilution to near extinction strategy to isolate previously uncultured marine strains [22]. The idea was developed further to study diluted cultures of entire bacterioplankton assemblages, in which depending on the dilution strategy, different parts of the bacterial community are selected for [23], [24]. Dilution experiments were successful in e.g. identifying selective grazing mortality of bacterial groups [25].

Here we present a highly replicated, culture-dependent investigation of direct pH effects on bacterial communities from the North Sea. The sampling station Helgoland Roads is well suited for this approach, as seasonal and long-term microbiological data are available [26]–[29] and dilution or enrichment experiments have been carried out before [25], [30]. We used three dilution approaches to study different bacterial groups and repeated the experiment in different seasons. We show that small changes in pH have direct effects on bacterial community composition and identify *Gammaproteobacteria*, *Flavobacteriaceae*, *Rhodobacteraceae* and *Campylobacteraceae* as phylogenetic groups responding most notably to differences in pH.

## Materials and Methods

### Ethics statement

No specific permits were required for the sampling and activities performed in the study.

### Experimental set-up and sampling

We conducted the experiment in spring, summer and autumn, 2010, and in winter 2011 (Table 1) with surface water sampled at 1 m depth at sampling station Helgoland Roads (54°11.3′N, 7°54.0′E), North Sea. Water samples were subjected to three different dilution protocols and three different pH levels (see sections below), yielding a total of nine different treatments. Each treatment was replicated five times in autoclaved one liter borosilicate glass bottles. Bottles were filled with 1100 ml to keep the headspace small (volume 50–60 ml) to prevent excessive CO_2_ exchange at the air-seawater interface observed in systems open to the atmosphere [31]. Bottles were incubated in the dark at approximately *in situ* temperature (Table 1) and were mixed daily by inversion. Samples were taken after four weeks of incubation.

**Table 1:** Sampling dates, initial seawater characteristics and incubation temperatures.

Season	Sampling day	pH_NBS_ *in situ*	Temperature [°C]^1^	Bacteria ml^−1^ fraction <10 µm	Incubation temperature [°C]
spring	April 8, 2010	8.26	4.7	5.0×10^5^	5.0
summer	July 1, 2010	8.22	15.8	1.9×10^6^	15.0
autumn	October 14, 2010	8.15	14.2	6.5×10^5^	14.0
winter	January 20, 2011	8.19	3.2	3.3×10^5^	3.0

1 Seawater temperatures were obtained from the Helgoland Roads time-series and were directly measured in a seawater sample on the vessel on station [76].

We used the following three dilution protocols: ‘no dilution’, ‘serial dilution’ (diluted weekly 1∶1000 starting after one week), and ‘initial dilution’ (initially diluted 1∶1000). Larger organisms were removed by filtration through 10 µm Isopore™ Membrane Filters (TCTP-type, Millipore, Eschborn, Germany). For the dilutions, we used seawater collected on the respective initial sampling date, which had been repeatedly sterilized by filtration (0.2 µm Isopore™ Membrane Filters, GTTP-type, Millipore).

The pH levels were the current *in situ* seawater pH (8.15–8.26, Table 1), pH 7.82 and 7.67. We acidified the initial seawater samples (fraction <10 µm) and the sterile-filtered seawater used for the dilutions with 2 M HCl. To determine pH at the beginning and after the four weeks of incubation, we used a ProLab 3000 pH meter with an IoLine pH combination electrode with temperature sensor (type IL-pHT-A170MF-DIN-N), calibrated with standard buffer solutions (pH 4.01, 6.87, 9.18) (all materials: SI Analytics, Mainz, Germany). All pH measurements were carried out at the respective incubation temperature and are reported on the National Bureau of Standards (NBS) scale.

For cell counts, samples of 4 ml were fixed with formaldehyde (1% [w/v] final concentration) for 1 h at room temperature and were subsequently stored at −80°C until further analysis. Aliquots of 500 µl were stained for 10 min with 10 µl of a freshly prepared 400× SYBR Green (invitrogen™, Life Technologies, Paisley, UK) solution in sterile filtered dimethyl sulfoxide (DMSO). Directly prior to staining the cells, we added 10 µl of a diluted solution of Fluoresbrite® Polychromatic Red Microspheres 1.0 µm (Polysciences Europe, Eppelheim, Germany) as an internal counting standard (final concentration of about 10% of the expected number of cells). We analysed samples with an Accuri C6 flow cytometer (BD Accuri Cytometers, Ann Arbor, MI, USA) with the fluidics setting “slow” for 1.5 min. To reduce noise, we set a threshold on FL1-H of 550 for winter and spring and of 600 for summer and autumn samples, respectively. The actual flow through was calibrated with BD Trucount™ Controls (BD Biosciences, San Jose, CA, USA).

Biomass was collected on 0.2 µm Isopore™ Membrane Filters (GTTP-type, 47 mm diameter, Millipore) and DNA extraction was performed as previously described [29]. DNA concentration and purity were determined by photometry using an Infinite 200 PRO NanoQuant (Tecan, Männedorf, Switzerland).

### ARISA

For ARISA fingerprints, we used forward primer L-D-Bact-132-a-A-18 (5′-CCGGGTTTCCCCATTCGG-3′) and reverse primer S-D-Bact-1522-b-S-20 (5′-TGCGGCTGGATCCCCTCCTT-3′) [32], the latter labeled with an infrared dye. PCR reactions were performed in volumes of 25 µl containing 10 ng template DNA, 2.5 µl Taq Buffer (10×), 5 µl TaqMaster PCR Enhancer (5×), 0.7 µl of each primer (20 µM), 0.75 µl dNTPs (2.5 mM each), and 1.4 U Taq DNA polymerase (5 Prime, Hamburg, Germany). Cycling conditions were: 95°C for 3 min, followed by 30 cycles of 95°C for 1 min, 50°C for 1 min and 68°C for 1 min, with a final step at 68°C for 5 min. Aliquots of PCR products were verified on agarose gels and depending on agarose gel band intensities, original or diluted PCR products were mixed with an equal volume of formamide containing stop mix. Samples were heated to 95°C for 2 min, subsequently kept on ice for 10 min, and 0.25 to 0.5 µl were separated in 5.5% polyacrilamide gels. Running conditions were 1500 V for 14 h on a LI-COR 4300 DNA Analyzer. A pre-run of 15 min at 45°C was carried out to precondition the gel and sequencer prior to loading the samples. As a size reference, we used a 50–1500 bp standard (all materials: LI-COR Biosciences, Lincoln, NE, USA).

ARISA gels were analyzed with the Bionumerics 5.10 software (Applied Maths, Sint-Martens-Latem, Belgium). Bands with intensities lower than 4% of the maximum value of the respective lane and bands smaller than 300 bp were neglected. Binning to band classes was performed according to Brown et al. [33]. Beta-diversity was calculated with the Jaccard coefficient.

### 16S ribosomal amplicon pyrosequencing

Based on ARISA results, samples were selected for pyrosequencing of the bacterial 16S rDNA (pH *in situ* and 7.67 of all ‘no dilution’ and ‘serial dilution’ treatments and of the ‘initial dilution’ in summer; additionally starting communities). Primers used to construct the amplicon library were of the structure 5′-[Roche's adaptor for long reads (Lib-L)] - [template-specific sequence]-3′. As template specific sequences we used forward primer GM3 (5′-AGAGTTTGATCMTGGC-3′) and reverse primer 907 (5′-CCGTCAATTCMTTTGAGTTT-3′) [34]. To pool all 93 samples in a single run, 93 different forward primers were constructed, each containing a different 10 bp long Multiplex Identifier (Roche) between the adaptor sequence and the template specific sequence. Amplifications were conducted in two duplicate reactions of 50 µl, each containing 20 ng of template DNA, 5 µl Taq Buffer (10×), 10 µl TaqMaster PCR Enhancer (5×), 1.25 µl of each primer (20 µM), 5 µl dNTPs (2.5 mM each), and 2.5 U of Taq DNA polymerase (5 Prime). Cycling conditions were: 94°C for 10 min, followed by 20 cycles of 94°C for 1 min, 44°C for 1.5 min and 68°C for 2 min, with a final step at 68°C for 15 min. The duplicate reactions were combined and purified with the QIAquick PCR purification kit (Qiagen, Valencia, CA, USA). Amplicons were pooled equimolarly, including 2 µl of a negative control with a separate Multiplex Identifier to check for contaminations, and sequenced on 1/2 plate on a Roche 454 GS-FLX Titanium platform at the Institute of Clinical Molecular Biology (IKMB), University of Kiel, Germany. Sequence data were deposited in the NCBI Sequence Read Archive (accession number SRP014019).

Sequences were processed with the MOTHUR software (version 1.23.0, [35]). Flowgrams were trimmed to 360 to 800 flows and the shhh.flows command with standard settings was used to remove sequencing noise. After the removal of primer and barcode sequences, unique sequences were aligned to the SILVA reference database. After removing chimeras (chimera.uchime routine) and chloroplast sequences, 163,029 sequences remained (20,419 of them unique). Sequences started and ended at the same alignment position, 95% being of a length between 381 and 420 bp. A distance matrix was created and sequences were grouped into operational taxonomic units (OTUs) on a 97% level. The number of sequences in each sample was normalized by randomly selecting the number of sequences present in the smallest sample (n = 494). This resulted in a subset containing 2133 OTUs which we used for all further analyses. Sample coverage and richness (observed number of OTUs) were obtained as implemented in MOTHUR. OTU abundance data were square root transformed and beta-diversity was calculated with the Bray-Curtis coefficient.

### Statistical analysis

The experiment was based on a crossed factorial design, consisting of the three factors ‘season’ (four levels, fixed), ‘dilution’ (three levels, fixed), and ‘pH’ (three levels, fixed). For pyrosequencing, the factors ‘dilution’ and ‘pH’ consisted of only two levels and the summer ‘initial dilution’ was analyzed separately. In case of a significant influence of ‘pH’ or of an interaction term involving ‘pH’, *post-hoc* comparisons for the factor ‘pH’ within the highest ranking interaction were performed.

Bacterial abundance and richness were analyzed through factorial ANOVAs [36] with Statistica 7.1 (StatSoft, Tulsa, OK, USA), using Tukey's HSD test for *post-hoc* comparisons. Beta-diversity was analyzed by permutational multivariate ANOVA (PERMANOVA, [37]) and similarity percentage analysis (SIMPER). SIMPER allowed us to calculate the total similarity within and dissimilarity between treatments, and to determine characteristic and discriminatory OTUs. To visualize patterns of the influence of ‘pH’ within ‘season’-‘dilution’ combinations, we performed principal co-ordinate analysis (PCO, [38]). For all multivariate analyses, we used Primer 6 with the add-on package PERMANOVA+ (PRIMER-E Ltd, Plymouth, UK).

## Results

### Environmental data, pH manipulations, initial community composition and bacterial abundance

**Figure 1:**
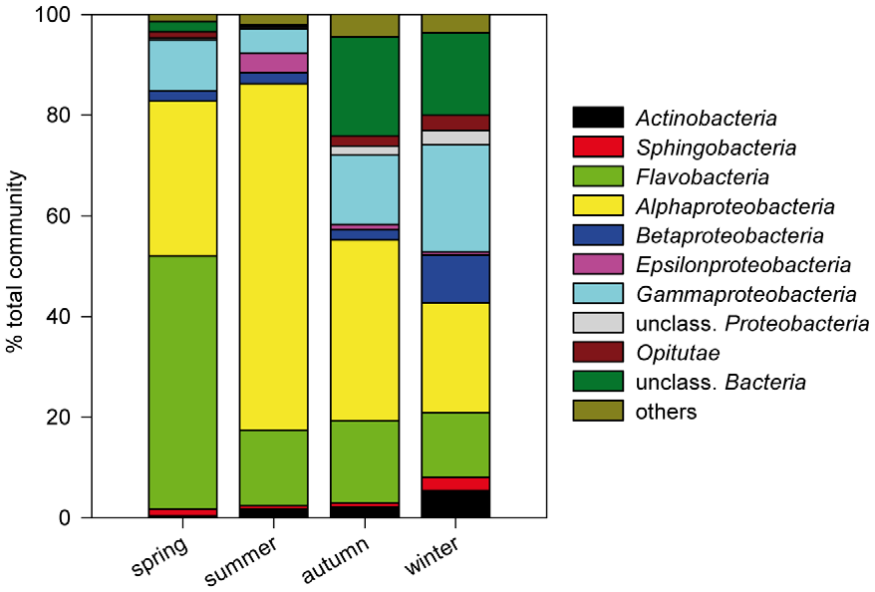
Relative abundance of bacterial classes in the starting communities (seawater fraction <10 µm). The chart was constructed based on the relative abundances of OTUs (16S ribosomal amplicon pyrosequencing) in the standardized subsample (n = 494 sequences). Classes represented by less than 2% of sequences are summarized under “others”.

*In situ* pH varied from 8.15 to 8.22 and seawater temperature from 3.2 to 15.8°C (Table 1). At the end of the experiment, the mean absolute deviations from the initial pH values were 0.10±0.06 (‘no dilution’, n = 60), 0.07±0.05 (‘serial dilution’, n = 59) and 0.62±0.28 (‘initial dilution’, n = 60) (see Table S1 for pH in all ‘season’-‘dilution’ combinations). Initial bacterial abundance ranged from 3.3×10^5^ to 1.9×10^6^ cells ml^−1^ (Table 1). The starting community was more diverse in autumn and winter (134 and 181 observed OTUs) compared to spring and summer (69 and 54 observed OTUs). Starting communities were dominated by sequences of *Alphaproteobacteria* (especially in summer), *Flavobacteria* (especially in spring) and *Gammaproteobacteria*. Additionally, up to 20% *Bacteria* that could not be assigned to classes (autumn and winter) and 10% *Betaproteobacteria* (winter) occurred (Figure 1).

After the four weeks of incubation, bacterial abundance ranged between 1.1×10^5^ and 6.1×10^6^ cells ml^−1^ and was significantly influenced by ‘season’ (F_3,143_ = 21.451, p<0.001), ‘dilution’ (F_2,143_ = 111.279, p<0.001), and their interaction (F_6,143_ = 14.399, p<0.001), but not by ‘pH’ (F_2,143_ = 1.6163, p = 0.202) (Figure S1).

### Bacterial community composition

Based on ARISA fingerprints, all three experimental factors and their interactions significantly influenced bacterial community structure (PERMANOVA, Table 2). The highest amount of variation was explained by ‘season’, ‘dilution’ and their interaction term (Sq. root, Table 2). Communities in both ‘no dilution’ and ‘serial dilution’ were significantly influenced by ‘pH’ in all seasons (Table 3), predominantly already at pH 7.82. Additionally, communities from both lowered pH treatments differed from each other, except in summer. ‘Initial dilution’-communities in contrast were influenced by ‘pH’ only in spring and summer.

**Table 2:** PERMANOVA main tests of bacterial community composition based on Jaccard dissimilarities of ARISA profiles.

Sources of variation	d.f.	SS	*pseudo F*	*p (perm)* 1	Sq. root
Season	3	104240	24.066	**0.001**	27.297
Dilution	2	99744	34.543	**0.001**	28.508
pH	2	8222.3	2.8475	**0.001**	6.6905
Season×Dilution	6	162610	18.772	**0.001**	41.498
Season×pH	6	20929	2.416	**0.001**	11.713
Dilution×pH	4	15976	2.7663	**0.001**	11.33
Season×Dilution×pH	12	48537	2.8015	**0.001**	22.881
Residuals	143	206460			37.997
Total	178	667400			

1 Significant results (*p (perm)*<0.05) are highlighted in bold.

Displayed are tests for the factors ‘season’, ‘dilution’, ‘pH’ and their interactions and the partitioning of multivariate variation. p-values were obtained using type III sums of squares and 999 permutations under the full model. d.f.: degrees of freedom, SS: sums of squares, Sq. root: square root of the component of variation attributable to that factor in the model, in units of Jaccard dissimilarities.

**Table 3:** PERMANOVA pair-wise comparisons of bacterial community composition based on Jaccard dissimilarities of ARISA profiles.

		no dilution		serial dilution		initial dilution	
Season	Comparison	*t (perm)*	*p (perm)* 1	*t (perm)*	*p (perm)* 1	*t (perm)*	*p (perm)* 1
spring	pH *in situ* vs. 7.82	1.7578	**0.009**	2.0402	**0.008**	1.4123	**0.022**
	pH *in situ* vs. 7.67	3.4604	**0.014**	1.4949	**0.032**	1.187	0.059
	pH 7.82 vs. 7.67	2.1161	**0.014**	1.7501	**0.017**	1.8286	**0.008**
summer	pH *in situ* vs. 7.82	1.38	**0.043**	1.7313	**0.011**	1.8089	**0.006**
	pH *in situ* vs. 7.67	1.7055	**0.008**	2.0209	**0.011**	1.5411	**0.01**
	pH 7.82 vs. 7.67	0.79417	0.836	1.2412	0.123	1.138	0.148
autumn	pH *in situ* vs. 7.82	1.7541	**0.006**	1.2752	0.12	1.179	0.083
	pH *in situ* vs. 7.67	2.0978	**0.011**	2.2008	**0.008**	1.1401	0.148
	pH 7.82 vs. 7.67	1.619	**0.005**	1.7405	**0.012**	0.86025	0.916
winter	pH *in situ* vs. 7.82	1.2116	0.163	3.3652	**0.007**	1.3223	0.051
	pH *in situ* vs. 7.67	1.3323	**0.046**	3.4765	**0.01**	1.3268	0.095
	pH 7.82 vs. 7.67	1.4745	**0.009**	3.3868	**0.008**	1.0803	0.259

1 Significant results (*p (perm)*<0.05) are highlighted in bold.

Displayed are pair-wise *a posteriori* comparisons of the factor ‘pH’ within ‘season’-‘dilution’ combinations, with at least 125 unique permutations per comparison.

For 16S ribosomal amplicon pyrosequencing, we selected all ‘season’-‘dilution’ combinations for which significant differences between communities at pH *in situ* and 7.67 had been observed (Table 3). Sequencing data confirmed that community structure in both ‘no dilution’ and ‘serial dilution’ was significantly influenced by all experimental factors and interactions (PERMANOVA, Table 4). In accordance with ARISA results, ‘season’, ‘dilution’ and their interaction term were the highest sources of variation (Sq. root, Table 4). Except for the ‘serial dilution’ in autumn, all ‘pH’-dependent differences were confirmed (Table 5 and Table 6). PCO plots of ‘season’-‘dilution’ combinations are shown in Figure S2 (based on ARISA) and Figure S3 (based on 16S ribosomal amplicon pyrosequencing).

**Table 4:** PERMANOVA main tests of bacterial community composition based on Bray-Curtis dissimilarities of OTUs (16S ribosomal amplicon pyrosequencing; ‘no dilution’ and ‘serial dilution’).

Sources of variation	d.f.	SS	*pseudo F*	*p (perm)* 1	Sq. root
Season	3	74205	22.705	**0.001**	34.644
Dilution	1	71118	65.279	**0.001**	42.167
pH	1	3637.6	3.339	**0.005**	8.0436
Season×Dilution	3	62133	19.011	**0.001**	44.63
Season×pH	3	10129	3.0993	**0.001**	15.237
Dilution×pH	1	3431.6	3.1499	**0.002**	10.906
Season×Dilution×pH	3	10272	3.1429	**0.001**	21.771
Residuals	63	68634			33.007
Total	78	303900			

1 Significant results (*p (perm)*<0.05) are highlighted in bold.

Displayed are tests for the factors ‘season’, ‘dilution’, ‘pH’ and their interactions and the partitioning of multivariate variation for ’no dilution’ and ‘serial dilution’ treatments. p-values were obtained using type III sums of squares and 999 permutations under the full model. d.f.: degrees of freedom, SS: sums of squares, Sq. root: square root of the component of variation attributable to that factor in the model, in units of Bray-Curtis dissimilarities.

**Table 5:** PERMANOVA pair-wise comparisons of bacterial community composition based on Bray-Curtis dissimilarities of OTUs (16S ribosomal amplicon pyrosequencing; ‘no dilution’ and ‘serial dilution’).

Comparison	no dilution		serial dilution	
pH *in situ* vs. 7.67	*t (perm)*	*p (perm)* 1	*t (perm)* 1	*p (perm)* 1
spring	2.3732	**0.009**	1.5667	**0.039**
summer	1.1759	**0.042**	1.9808	**0.013**
autumn	1.5233	**0.005**	1.2019	0.146
winter	1.3372	**0.008**	3.7336	**0.013**

1 Significant results (*p (perm)*<0.05) are highlighted in bold.

Displayed are pair-wise *a posteriori* comparisons of the factor ‘pH’ within ‘season’-‘dilution’ combinations for ‘no dilution’ and ‘serial dilution’ treatments, with at least 125 unique permutations per comparison.

**Table 6:** PERMANOVA main tests of bacterial community composition based on Bray-Curtis dissimilarities of OTUs (16S ribosomal amplicon pyrosequencing; ‘initial dilution’ summer).

Sources of variation	d.f.	SS	*pseudo F*	*p (perm)* 1	Sq. root
pH	1	1922.8	1.9165	**0.011**	14.384
Residuals	7	7023.1			31.675
Total	8	8945.9			

1 Significant results (*p (perm)*<0.05) are highlighted in bold.

Displayed are the test for the factor ‘pH’ and the partitioning of multivariate variation for the ‘initial dilution’ (only summer experiment). p-values were obtained using type III sums of squares and 999 permutations under the full model. d.f.: degrees of freedom, SS: sums of squares, Sq. root: square root of the component of variation attributable to that factor in the model, in units of Bray-Curtis dissimilarities.

Sample coverage obtained by pyrosequencing ranged from 72.1 to 99.8% (Table S2), yielding bacterial richness estimates of 8 to 218 OTUs per sample (Figure S4). In both ‘no dilution’ and ‘serial dilution’, richness was significantly influenced by ‘season’ (F_3,63_ = 122.201, p<0.0001), ‘dilution’ (F_1,63_ = 1901.215, p<0.0001) and ‘pH’ (F_1,63_ = 29.608, p<0.0001), and the interactions ‘season’x‘dilution’ (F_3,63_ = 138.125, p<0.0001), ‘season’x‘pH’ (F_3,63_ = 9.317, p<0.0001), ‘dilution’x‘pH’ (F_1,63_ = 28.395, p<0.0001), and ‘season’x‘dilution’x‘pH’ (F_3,63_ = 2.880, p<0.05). For the ‘serial dilution’, no ‘pH’-dependent differences were found, whereas for the ‘no dilution’, richness was significantly higher at pH 7.67 than at pH *in situ* in spring (p<0.001) and autumn (p<0.001, Tukey's HSD test, 63 d.f.). In the ‘initial dilution’ in summer, richness was not significantly influenced by ‘pH’ (F_1,7_ = 1.093, p = 0.331).

### Bacterial groups in the ‘season’-‘dilution’ combinations

**Figure 2:**
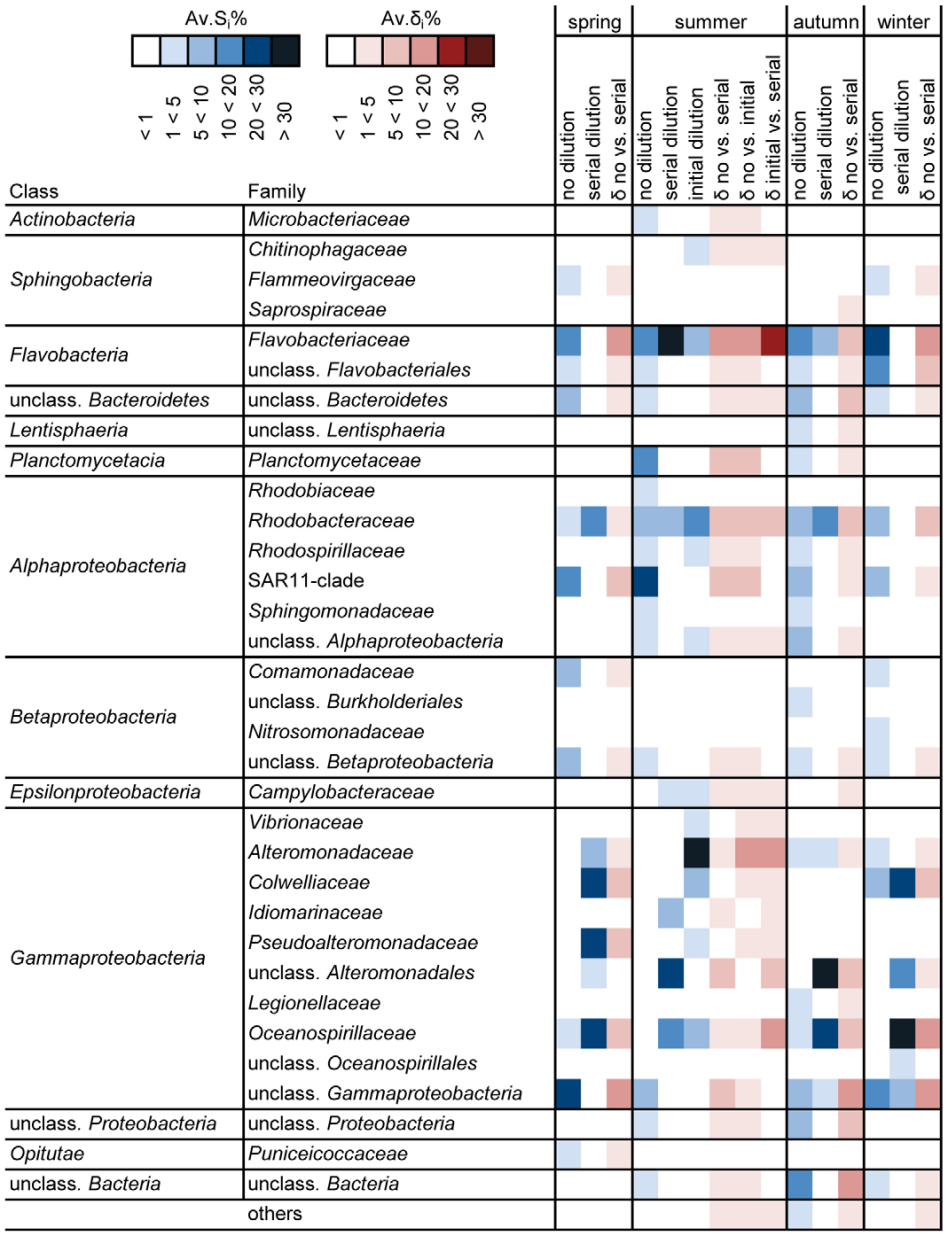
Bacterial groups in the ‘season’-‘dilution’ combinations, based on SIMPER analysis of pH *in situ* levels. Displayed are phylogenetic groups jointly contributing to 90% of the total similarity within and dissimilarity between pH *in situ* levels of the different dilution treatments, separately for each season (no: ‘no dilution’, serial: ‘serial dilution’, initial: ‘initial dilution’). The heat map summarizes the contributions of single OTUs (16S ribosomal amplicon pyrosequencing) on the family level. Av.S_i_%: average percentage contribution of the i th species to the total similarity, Av.δ_i_%: average percentage contribution of the i th species to the total dissimilarity. Bacterial families contributing less than 1% to both totals are summarized under “others”. The amount of contribution is indicated by the color of cells, darker colors represent higher contributions.

Considering only pH *in situ* treatments, i.e. disregarding pH effects, communities in different dilutions within a given season differed between 86 and 97% from each other. Classes predominantly discriminating between ‘no dilution’ and ‘serial dilution’ were *Flavobacteria*, *Alphaproteobacteria* and *Gammaproteobacteria*. (Figure 2; see Table S3 for similarity and Table S4 for dissimilarity analyses).

‘No dilution’-communities were characterized mainly by *Flavobacteria*, *Alphaproteobacteria* and *Gammaproteobacteria* which could not be assigned to families. *Flavobacteria* were the most important group in spring and winter, while in summer, *Alphaproteobacteria*, especially of the SAR11-clade, dominated. In autumn, the community was more diverse. Less abundant groups characteristic for ‘no dilution’-communities included *Planctomycetacia*, *Betaproteobacteria*, and *Bacteria* that could not be assigned to classes.

In contrast, ‘serial dilution’-communities were dominated by known representatives of the *Gammaproteobacteria*. Important groups were *Oceanospirillaceae* (all seasons), unclassified *Alteromonadales* (summer, autumn, winter), *Colwelliaceae* (spring, winter) and *Pseudoalteromonadaceae* (spring). In certain ‘serial dilution’-communities, *Flavobacteriaceae* and *Rhodobacteraceae* additionally accounted for high contributions to total similarities. The ‘initial dilution’-community in summer was dominated by *Gammaproteobacteria*, *Flavobacteriaceae* and *Rhodobacteraceae* as well. Within the *Gammaproteobacteria* however, *Alteromonadaceae* dominated.

### Bacterial groups responding differently to pH

**Figure 3:**
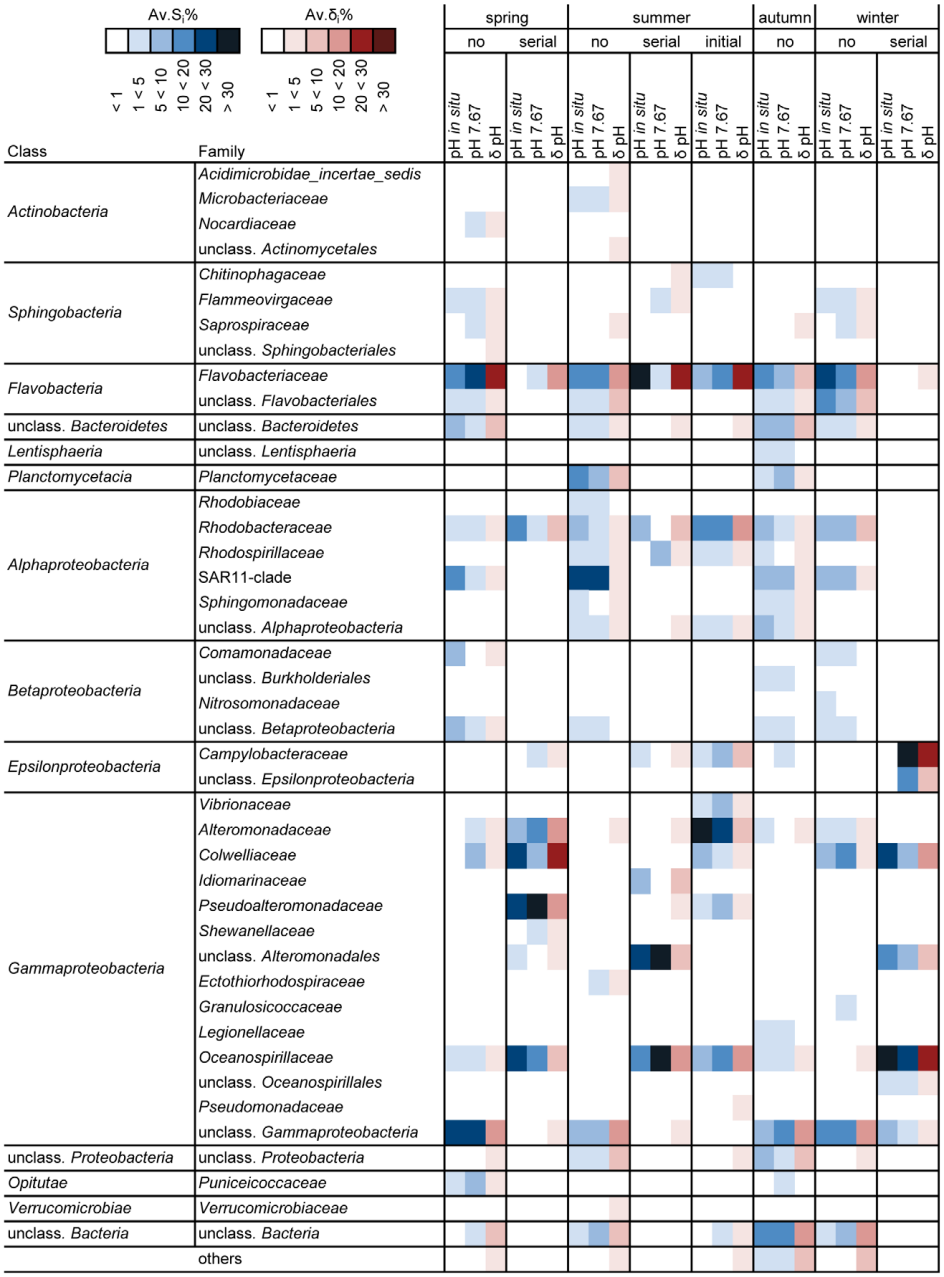
Bacterial groups responding differently to pH, based on SIMPER analysis. Displayed are phylogenetic groups jointly contributing to 90% of the total similarity within and dissimilarity between pH levels, separately for each ‘season’-‘dilution’ combination (no: ‘no dilution’, serial: ‘serial dilution’, initial: ‘initial dilution’). The heat map summarizes the contributions of single OTUs (16S ribosomal amplicon pyrosequencing) on the family level. Av.S_i_%: average percentage contribution of the i th species to the total similarity, Av.δ_i_%: average percentage contribution of the i th species to the total dissimilarity. Bacterial families contributing less than 1% to both totals are summarized under “others”. The amount of contribution is indicated by the color of cells, darker colors represent higher contributions.

In ‘season’-‘dilution’ combinations with significant ‘pH’ effects (Table 5 and Table 6), communities of the two pH treatments differed between 49 and 63% from each other. The most important discriminatory classes were *Gammaproteobacteria*, *Flavobacteria*, *Alphaproteobacteria* and *Epsilonproteobacteria* (Figure 3; see Table S3 for similarity and Table S5 for dissimilarity analyses).

*Gammaproteobacterial* families contributing to total dissimilarities were *Oceanospirillaceae*, *Colwelliaceae*, *Alteromonadaceae*, *Pseudoalteromonadaceae*, and to a lesser extend *Idiomarinaceae*, *Shewanellaceae* and *Vibrionaceae*. In spring and winter, *Oceanospirillaceae* were more characteristic for pH *in situ*, while in summer, they were more characteristic for pH 7.67. *Colwelliaceae* contributed more to total similarities at pH *in situ* in the ‘serial dilution’ in spring and winter and the ‘initial dilution’ in summer. In the ‘no dilution’ however, the opposite occurred. *Alteromonadaceae* dominated in the summer ‘initial dilution’, were they contributed more to total similarity at pH *in situ*. The same was found for the ‘no dilution’ in autumn, but in spring, they were more characteristic for pH 7.67. *Pseudoalteromonadaceae* contributed to total dissimilarity in spring and summer, and were always more characteristic for pH 7.67. Concerning *Gammaproteobacterial* families occurring in only one ‘season’-‘dilution’ combination, *Idiomarinaceae* contributed more to total similarity at pH *in situ*, while *Shewanellaceae* and *Vibrionaceae* contributed more to total similarity at pH 7.67.

The *Flavobacterial* family *Flavobacteriaceae* was the group with the overall highest contributions to total dissimilarities. In spring, *Flavobacteriaceae* were more characteristic for pH 7.67, but in autumn and winter, they were more characteristic for pH *in situ*. In summer, no consistent trend was found.

Within the *Alphaproteobacteria*, high contributions to total dissimilarity were attributed to *Rhodobacteraceae*, and to lesser extends to SAR11-clade, *Rhodospirillaceae* and *Sphingomonadaceae*. For *Rhodobacteraceae*, higher contributions to total similarity were generally observed at pH *in situ*. SAR11-clade contributed more to total similarity at pH *in situ* in spring, but no important differences were found in the other seasons. The other *Alphaproteobacterial* families did not show consistent trends.

Within the *Epsilonproteobacteria*, *Campylobacteraceae*, represented exclusively by the genus *Arcobacter*, contributed a third to total similarity at pH 7.67 in the ‘serial dilution’ in winter but were absent at pH *in situ*. Similar but less pronounced tendencies were found in spring and autumn.

Discriminatory groups that were less abundant or present only in one or few ‘season’-‘dilution’ combinations predominantly belonged to the classes *Sphingobacteria*, *Planctomycetacia*, *Actinobacteria*, *Betaproteobacteria* and *Opitutae*. Notably, also various groups that could not be assigned to families contributed to ‘pH’-dependent dissimilarities.

## Discussion

The impact of ocean acidification on bacterial communities remains under debate as studies conducted so far are highly complex but lack sufficient replication. Here we present a thorough analysis of diversely assembled bacterial communities, shaped by the experimental factors season and dilution. Season takes into account phenology, i.e. climate-driven annually recurring patterns of pelagic bacterial community composition [16]. Dilution on the other hand acts as a selective factor for the two major general ecological strategies observed among bacterioplankton: Groups efficiently competing at ambient nutrient levels and groups efficiently exploiting environmental patchiness, i.e. nutrient availability [39]. The first group was selected for in the undiluted incubations while different members of the second type were selected for by the dilution strategies. Despite the differently assembled communities, we observed community shifts in the undiluted incubations and in one of the dilution strategies in all seasons. We identified bacterial groups susceptible to changes in pH, highlighting that their specific response is often context-dependent.

### Considerations on perturbations of the carbonate system using HCl

We used HCl to adjust pH to values expected for the North Sea for the year 2100. Values remained relatively stable for ‘no dilution’ and ‘serial dilution’ treatments, but larger deviations were observed for the ‘initial dilution’. Interestingly, pH-dependent community shifts were least frequently found in initially diluted communities, probably at least partly due to these pH instabilities. Thus our 16S ribosomal amplicon pyrosequencing analyses and consequently our main findings and conclusions predominantly rely on ‘no dilution’ and ‘serial dilution’ treatments. Despite the relatively high pH deviations in ‘initial dilution’ treatments, we decided to report these results as well to present the complete experiment.

Although acidifying seawater with HCl has been extensively applied in ocean acidification studies [31], [40], it is not the most accurate approach in mimicking future changes in carbonate chemistry. In the course of ocean acidification, seawater CO_2_ uptake will cause a reduction in pH accompanied by an increase in dissolved inorganic carbon (DIC, composed of the species H_2_CO_3_, CO_2_(aq), HCO_3_^−^ and CO_3_^2−^) but no change in total alkalinity (TA). In contrast, additions of HCl lead to a decrease in pH at constant DIC and decreasing TA [40], [41]. Still both methods lead to similar results, i.e. a decrease in pH and [CO_3_^2−^] and an increase in [CO_2_] and [HCO_3_^−^] [40]. For pCO_2_ values not considerably exceeding 700 ppm, all carbonate system parameters change in similar magnitude with both perturbation methods [40]. Higher acid perturbations lead to larger differences however, e.g. acidification by HCl to a pH of 7.5 leads to [HCO_3_^−^] values being about 22% lower compared to values reached by acidification to this pH by CO_2_ uptake [41]. Whether the small differences in carbonate system parameters between the two methods are problematic thus depends on whether the processes investigated are sensitive to small differences in [HCO_3_^−^] [40].

Concerning bacterial communities, the ability of certain groups to compete at different pH levels may depend both on changes in pH per se and on processes involving HCO_3_^−^ availability. Changes in pH are likely to have a physiological effect, as bacteria living in the alkaline marine milieu (with pH values generally above 8.0) have to invert energy into the homeostasis of their cytoplasmic pH (7.4–7.8) [42]. Consequently, if the difference between external and internal pH becomes smaller due to ocean acidification, bacteria may profit energetically, depending on their pH homoeostasis mechanisms [43]. In a study investigating two marine bacterial strains, a *Cytophaga* strain exhibited a decreased respiration rate and thus enhanced growth efficiency at higher pCO_2_, which was not found for a *Roseobacter* strain though [43]. In addition, both strains exhibited higher rates of heterotrophic CO_2_ fixation, subsequently released as DOM, at higher pCO_2_ levels [43], which demonstrates that [HCO_3_^−^] plays an important role as well. With our perturbation approach using HCl, pH-dependent changes were likely to be detected, while the consequences of rising DIC availability may have been underestimated. Yet as all carbonate system species ([CO_2_], [HCO_3_^−^], [CO_3_^2−^], [H^+^]) will change in the same direction with both HCl and CO_2_ perturbations, biological responses are unlikely to differ substantially and results should be directly comparable [31], [40]. This has already been demonstrated for other organisms in ocean acidification studies systematically comparing both methods for pCO_2_ values of up to 1200 ppm, namely for corals [44] and calcifying algae [45].

### Influence of pH on bacterial community structure and abundance

We combined ARISA and extensive 16S ribosomal amplicon pyrosequencing to permit a robust statistical analysis. The strategy of simultaneously pyrosequencing a large number of samples, accepting fewer sequences instead of deep sampling, was recently applied to analyze bacterioplankton communities [46] and was highly suitable for our study regarding the comparatively low bacterial diversity in laboratory incubations.

Not surprisingly, both methods revealed that the greatest influence on bacterial community composition could be attributed to season and dilution. Within the differently assembled communities, pH had a significant effect on all ‘no dilution’ and ‘serial dilution’ incubations (excluding pyrosequencing results for the ‘serial dilution’ in autumn). This indicates that even at moderate pH differences, community shifts can be observed. Furthermore, analysis of the complete data set by ARISA revealed that differences in community structure were predominantly observed already at pH 7.82, suggesting that a tipping point for bacterial community shifts may be reached even earlier. Communities from pH 7.82 and 7.67 also differed from each other (except in summer) indicating that slight differences in the degree of pH reduction are crucial. For ‘initial dilution’-communities however, we observed pH-dependent differences only in spring and summer. Possibly, in addition to the pH instabilities discussed above, the variability between replicates introduced by the 1000-fold dilution at the beginning masked pH effects in autumn and winter, where initial diversity was higher.

In agreement with community structure, richness was predominantly influenced by season and dilution. Differences between pH levels were only observed in ‘no dilution’-communities in spring and autumn, with higher richness at pH 7.67. Increased bacterial diversity at reduced pH has been reported for coastal microbial biofilms exposed to natural CO_2_ vents under low, but not under ambient light conditions [47] and for coral microbial communities [10], where it was assumed to be the outcome of a temporary disturbance of community equilibrium [48]. Although our results provide only minor evidence to support this hypothesis, a moderate reduction in pH seems at least not to reduce bacterial diversity, as observed for other environmental stressors such as chemical compounds [49]. Despite the good agreement concerning community structure, bacterial richness estimates were not consistent between 16S ribosomal amplicon pyrosequencing and ARISA (data not shown). Limitations of ARISA in estimating bacterial richness have previously been reported [50], [51], but similarly, pyrosequencing can lead to an overestimation of richness due to sequencing errors and the presence of chimeric sequences [52]. Using a subsample of equal number of sequences for all analyses however, we at least “standardized” these error sources [53] and therefore consider pyrosequencing richness estimates comparable between samples of our study.

Concerning bacterial abundance, a reduction in pH down to 7.67 did not have an effect, which is in agreement with previous findings from mesocosm studies [14], [15], [54]. In this context, possible pH effects on nutrient availability and utilization should be considered. It has been reported that with decreasing seawater pH, bioavailability of dissolved iron decreases [55]. Additionally, significant changes in phosphate, silicate and ammonia have been predicted for the seawater pH reductions expected due to ocean acidification [56], [57]. For hydrolysis reactions of dissolved organic matter such as organic acids, proteins and humic materials, a strong pH influence was predicted as well [58], but no detailed investigations exist. Furthermore, lower pH was shown to lead to an increased activity of total protease in mesocosms [54] and extracellular α- and β-glucosidase in laboratory experiments investigating direct pH effects [59]. In contrast, different responses of these enzymes were found after short-term incubation (3 h) at reduced pH [60]. In our experiment, the lack of pH-dependent differences in bacterial abundances suggests no change in overall nutrient availability or utilization at reduced pH.

### Bacterial groups in the ‘season’-‘dilution’ combinations

The specific members of bacterial groups establishing in the dilutions differed with the initial seasonal starting communities, but the following general trends were observed: Both dilution strategies resulted in the selection of known representatives of culturable *Gammaproteobacteria*, which has previously been reported for initially diluted seawater incubations [23], [25]. Dilution and unavoidable disruption of some cells during filtration result in more nutrients per individual cell than in the original sample, which *Gammaproteobacteria* exploit most efficiently [20], [23], [26], [61]. Apparently, when an incubation is diluted only after one week (and then repeatedly), other groups of *Gammaproteobacteria* establish than in an initial dilution. The comparatively high diversity maintained in the undiluted treatment may in turn be explained by the lack of nutrient input, preventing the dominance of fast-growing bacteria. The aspect of grazing may also have played a role in structuring the communities, as neither prefiltration nor dilution can completely prevent the development of grazers [25]. Likewise, lysis of bacterial cells due to bacteriophages cannot be excluded. Whether viral pressure differed between the pH levels cannot be determined as direct pH effects on marine planktonic bacteriophages have not been investigated so far [62].

### Bacterial groups responding differently to pH

Various bacterial groups contributed to pH-dependent dissimilarities, predominantly different members of *Gammaproteobacteria*, *Flavobacteriaceae*, *Rhodobacteraceae* and *Campylobacteraceae*. Further discriminatory groups included *Sphingobacteria*, SAR11-clade, *Planctomycetacia*, *Betaproteobacteria*, *Actinobacteria*, *Opitutae* and bacteria that could not be assigned to families. Responses of bacterial groups were generally context-dependent, i.e. different in particular season and growth condition combinations.

Concerning *Gammaproteobacteria*, mostly the *Oceanospirillaceae* and families of the order *Alteromonadales* (*Colwelliaceae*, *Alteromonadaceae*, *Pseudoalteromonadaceae*, *Idiomarinaceae*, *Shewanellaceae* and others) were affected by pH. Among these groups, *Pseudoalteromonadaceae* were consistently found more characteristic for pH 7.67. In contrast, the response of *Oceanospirillaceae* and *Alteromonadaceae* was dependent on season, as *Oceanospirillaceae* were more characteristic for pH *in situ* in spring and winter, but more characteristic for pH 7.67 in summer; the opposite occurred for *Alteromonadaceae*. *Colwelliaceae* were more dependent on dilution, being more characteristic for pH *in situ* in undiluted treatments and more characteristic for pH 7.67 in diluted treatments. The dependence of the pH-response of *Gammaproteobacteria* on different environmental factors is supported by contrasting results obtained for two coral species transferred to high CO_2_ vent sites [12]. Although they occurred only in one ‘season’-‘dilution’ combination and did not reach high abundances, it is interesting to note that *Vibrionaceae* were more characteristic for pH 7.67, as an increase in *Vibrionaceae* and other disease-associated bacteria at reduced pH has previously been reported in corals [10], [11]. It remains to be elucidated whether this is a general trend, as the family contains human and animal pathogens [63], and is of increasing importance in temperate waters due to climate change [64]–[67].

*Flavobacteriaceae* were the family with overall highest contributions to pH-dependent dissimilarities, but no season or dilution specific trends were found. The *Alphaproteobacterial* groups *Rhodobacteraceae* and SAR11-clade were generally more characteristic for pH *in situ*. A change in the relative abundances of *Flavobacteriaceae* and *Rhodobacteracea*e in response to pH has previously been reported in biofilms from the Great Barrier Reef [13], with *Rhodobacteraceae* decreasing and *Flavobacteriaeae* increasing with decreasing pH. In contrast, in a laboratory experiment with coral microbial communities, an increase of the relative abundance of *Rhodobacteraceae* at low pH was reported [10]. Thus within both of these complex groups, pH responses differ.

A striking growth of *Campylobacteraceae* was observed in the winter ‘serial dilution’ at pH 7.67, but not at pH *in situ*. Also in the other seasons, *Campylobacteraceae* were generally more characteristic for pH 7.67. This family was only represented by members of the genus *Arcobacter*, which has previously been reported at the study site [26]. Members of this genus are considered emerging human pathogens, often associated with fecal contamination of waters [68], [69]. *Arcobacter* have also been described in terrestrial and marine animals, with shellfish being a potential source of human infection [70]. It may seem surprising that this group was most important in the winter experiment, but in context of food preservation, *Arcobacter* have previously been reported to grow at refrigeration temperatures [70]. Regarding pH tolerance, *Arcobacter* spp. were reported to grow at pH 5.5 to 8.0 with a growth optimum at pH 6.0 to 7.5 [71]. Moreover, members of the *Campylobacteraceae* generally grow within broad pH ranges, and growth especially at low pH values was often reported regarding the survival in contaminated foods and the stomach transit [71], [72], [73].

Concerning the less abundant groups, *Opitutae* (*Verrucomicrobia*) are noteworthy as *Verrucomicrobia* have only recently been reported to be nearly ubiquitously distributed and are especially abundant in waters around Helgoland [74]. They were represented by the family *Puniceicoccaceae* and were more characteristic for pH 7.67 in spring and autumn. Similarly in corals, members of *Verrucomicrobiae* were only present at reduced pH [10].

In summary, no consistent trend throughout all ‘season’-‘dilution’ combinations was found for the majority of bacterial groups. Notable exceptions (neglecting groups that were only present in one ‘season’-‘dilution’ combination) were *Pseudoalteromonadaceae* and *Opitutae*, which were always more characteristic for pH 7.67, suggesting that they may profit from pH reductions. The same tendency was found for *Arcobacter*. In contrast, groups that were generally more characteristic for pH *in situ* were *Rhodobacteraceae* and SAR11-clade, hinting at difficulties of these groups to cope with reductions in pH.

Regarding the lack of additional trends, the following aspects have to be considered: First, bacterial families such as the *Rhodobacteraceae* are highly diverse and different members occur at certain time points, while being of very low importance in other seasons [27], [75]. Thus, supposing that different responses to pH occur at the genus, species, or even ecotype level, an analysis on the family level can only depict very rough patterns. Second, different environmental factors are likely to influence the response of bacterial groups to pH. In our experiment, a dependence on season could - besides the identity aspect mentioned above - hint at temperature effects, while a dependence on dilution could be associated with nutrient effects. Additionally, the co-occurring species must be taken into account. The high contribution to total similarity of *Arcobacter* at pH 7.67 in the winter ‘serial dilution’-community for instance, may in turn have influenced the comparatively lower contribution of *Oceanospirillaceae* at this pH and vice versa.

In conclusion already moderate changes in pH have the potential to cause shifts in bacterial communities, depending on the pool of bacterial groups constituting this community and on the prevailing environmental conditions. We identified members of the *Gammaproteobacteria*, *Flavobacteriaceae*, *Rhodobacteraceae* and *Campylobacteraceae* as susceptible to changes in pH and confirmed some results obtained in previous studies. To ultimately draw conclusions about functional implications, the question remains whether a change in species composition observed in *in vitro* incubations starting with an abrupt change in pH is extendable to natural bacterioplankton communities and how adaptation will affect long-term consequences. Therefore, to test whether the patterns observed also exist in more complex environments, we suggest that the groups identified in this study deserve special attention in future long-term and mesocosm experiments.

## Supporting Information

Figure S1 **Bacterial abundance in the different treatments.** No significant pH effect on bacterial abundance was found (ANOVA).
(TIF)

Figure S2 **Influence of the factor ‘pH’ on bacterial community composition (ARISA).** Displayed are principal co-ordinate analysis plots (PCOs) for each ‘season’-‘dilution’ combination based on Jaccard dissimilarities of ARISA profiles. Symbol shape represents the pH level (circles: pH *in situ*, squares: pH 7.82, triangles: pH 7.67).
(TIF)

Figure S3 **Influence of the factor ‘pH’ on bacterial community composition (16S ribosomal amplicon pyrosequencing).** Displayed are principal co-ordinate analysis plots (PCOs) for each ‘season’-‘dilution’ combination based on Bray-Curtis dissimilarities of OTUs (16S ribosomal amplicon pyrosequencing). Symbol shape represents the pH level (circles: pH *in situ*, triangles: pH 7.67).
(TIF)

Figure S4 **Bacterial richness in the different treatments.** Richness was determined based on the number of OTUs (16S ribosomal amplicon pyrosequencing) in the standardized subsample (n = 494 sequences) and was compared by ANOVA, with Tukey's HSD test for *post-hoc* comparisons. Asterisks (*) represent significant differences (p<0.05) between pH levels within a ‘season’-‘dilution’ combination.
(TIF)

Table S1 **pH after the four weeks of incubation.**
(PDF)

Table S2 **Sample coverage obtained by 16S ribosomal amplicon pyrosequencing, based on the standardized subsample (n = 494 sequences).**
(PDF)

Table S3 **Results of the SIMPER analysis giving the similarities within ‘season’-‘dilution’-‘pH’ combinations.** Displayed are the OTUs (16S ribosomal amplicon pyrosequencing) that predominantly contributed to 90% of to the total similarity. Av.A_i_: average abundance of the i th species over all samples of the treatment, Av.S_i_: average contribution of the i th species to the total similarity, Av.S_i_/SD: the average value of the i th species as a typifying species, Av.S_i_%: average percentage contribution of the i th species to the total similarity, ∑Av.S_i_%: average cumulative contribution to the total similarity.
(PDF)

Table S4 **Results of the SIMPER analysis giving the dissimilarities between pH** ***in situ*** **levels of the different dilution treatments, separately for each season.** Displayed are the OTUs (16S ribosomal amplicon pyrosequencing) that predominantly contributed to 90% of to the total dissimilarity. Av.A_i_: average abundance of the i th species over all samples of the treatment (no: ‘no dilution’, serial: ‘serial dilution’, initial: ‘initial dilution’), Av.δ_i_: average contribution of the i th species to the total dissimilarity, Av.δ_i_/SD: the average value of the i th species as a discriminating species, Av.δ_i_%: average percentage contribution of the i th species to the total dissimilarity, ∑Av.δ_i_%: average cumulative contribution to the total dissimilarity.
(PDF)

Table S5 **Results of the SIMPER analysis giving the dissimilarities between the pH levels** ***in situ*** **and 7.67 of ‘season’-‘dilution’ combinations significantly influenced by ‘pH’ according to PERMANOVA.** Displayed are the OTUs (16S ribosomal amplicon pyrosequencing) that predominantly contributed to 90% of to the total dissimilarity. Av.A_i_: average abundance of the i th species over all samples of the treatment (no: ‘no dilution’, serial: ‘serial dilution’, initial: ‘initial dilution’), Av.δ_i_: average contribution of the i th species to the total dissimilarity, Av.δ_i_/SD: the average value of the i th species as a discriminating species, Av.δ_i_%: average percentage contribution of the i th species to the total dissimilarity, ΣAv.δ_i_%: average cumulative contribution to the total dissimilarity.
(PDF)

## References

[1] Feely RA, Doney SC, Cooley SR (2009) Ocean acidification: Present conditions and future changes in a high-CO_2_ world. Oceanography 22: 36–47.

[2] Sabine CL, Feely RA, Gruber N, Key RM, Lee K, et al (2004) The oceanic sink for anthropogenic CO_2_ . Science 305: 367–371. 15256665 10.1126/science.1097403

[3] Caldeira K, Wickett ME (2003) Anthropogenic carbon and ocean pH. Nature 425: 365–365. 14508477 10.1038/425365a

[4] Pearson PN, Palmer MR (2000) Atmospheric carbon dioxide concentrations over the past 60 million years. Nature 406: 695–699. 10963587 10.1038/35021000

[5] Blackford JC, Gilbert FJ (2007) pH variability and CO_2_ induced acidification in the North Sea. J Mar Syst 64: 229–241.

[6] Dupont S, Dorey N, Thorndyke M (2010) What meta-analysis can tell us about vulnerability of marine biodiversity to ocean acidification? Estuar Coast Shelf Sci 89: 182–185.

[7] Hendriks IE, Duarte CM, Álvarez M (2010) Vulnerability of marine biodiversity to ocean acidification: A meta-analysis. Estuar Coast Shelf Sci 86: 157–164.

[8] Joint I, Doney SC, Karl DM (2011) Will ocean acidification affect marine microbes? ISME J 5: 1–7. 20535222 10.1038/ismej.2010.79 PMC3105673

[9] Liu JW, Weinbauer MG, Maier C, Dai M, Gattuso JP (2010) Effect of ocean acidification on microbial diversity and on microbe-driven biogeochemistry and ecosystem functioning. Aquat Microb Ecol 61: 291–305.

[10] Meron D, Atias E, Kruh LI, Elifantz H, Minz D, et al (2011) The impact of reduced pH on the microbial community of the coral *Acropora eurystoma* . ISME J 5: 51–60. 20668489 10.1038/ismej.2010.102 PMC3105665

[11] Vega Thurber R, Willner-Hall D, Rodriguez-Mueller B, Desnues C, Edwards RA, et al (2009) Metagenomic analysis of stressed coral holobionts. Environ Microbiol 11: 2148–2163. 19397678 10.1111/j.1462-2920.2009.01935.x

[12] Meron D, Rodolfo-Metalpa R, Cunning R, Baker AC, Fine M, et al (2012) Changes in coral microbial communities in response to a natural pH gradient. ISME J 6: 1775–1785. 22437157 10.1038/ismej.2012.19 PMC3498918

[13] Witt V, Wild C, Anthony KRN, Diaz-Pulido G, Uthicke S (2011) Effects of ocean acidification on microbial community composition of, and oxygen fluxes through, biofilms from the Great Barrier Reef. Environ Microbiol 13: 2976–2989. 21906222 10.1111/j.1462-2920.2011.02571.x

[14] Allgaier M, Riebesell U, Vogt M, Thyrhaug R, Grossart HP (2008) Coupling of heterotrophic bacteria to phytoplankton bloom development at different *p*CO_2_ levels: A mesocosm study. Biogeosciences 5: 1007–1022.

[15] Newbold LK, Oliver AE, Booth T, Tiwari B, Desantis T, et al (2012) The response of marine picoplankton to ocean acidification. Environ Microbiol 14: 2293–2307. 22591022 10.1111/j.1462-2920.2012.02762.x

[16] Andersson AF, Riemann L, Bertilsson S (2010) Pyrosequencing reveals contrasting seasonal dynamics of taxa within Baltic Sea bacterioplankton communities. ISME J 4: 171–181. 19829318 10.1038/ismej.2009.108

[17] Fuhrman JA, Hewson I, Schwalbach MS, Steele JA, Brown MV, et al (2006) Annually reoccurring bacterial communities are predictable from ocean conditions. Proc Nat Acad Sci USA 103: 13104–13109. 16938845 10.1073/pnas.0602399103 PMC1559760

[18] Gilbert JA, Steele JA, Caporaso JG, Steinbrueck L, Reeder J, et al (2012) Defining seasonal marine microbial community dynamics. ISME J 6: 298–308. 21850055 10.1038/ismej.2011.107 PMC3260500

[19] Ammerman JW, Fuhrman JA, Hagstrom A, Azam F (1984) Bacterioplankton growth in seawater: I. Growth kinetics and cellular characteristics in seawater cultures. Mar Ecol Prog Ser 18: 31–39.

[20] Ferguson RL, Buckley EN, Palumbo AV (1984) Response of marine bacterioplankton to differential filtration and confinement. Appl Environ Microbiol 47: 49–55. 6696422 10.1128/aem.47.1.49-55.1984 PMC239610

[21] Button DK, Schut F, Quang P, Martin R, Robertson BR (1993) Viability and isolation of marine bacteria by dilution culture: Theory, procedures, and inital results. Appl Environ Microbiol 59: 881–891. 16348896 10.1128/aem.59.3.881-891.1993 PMC202203

[22] Schut F, Devries EJ, Gottschal JC, Robertson BR, Harder W, et al (1993) Isolation of typical marine bacteria by dilution culture: Growth, maintenance, and characteristics of isolates under laboratory conditions. Appl Environ Microbiol 59: 2150–2160. 16348992 10.1128/aem.59.7.2150-2160.1993 PMC182250

[23] Fuchs BM, Zubkov MV, Sahm K, Burkill PH, Amann R (2000) Changes in community composition during dilution cultures of marine bacterioplankton as assessed by flow cytometric and molecular biological techniques. Environ Microbiol 2: 191–201. 11220305 10.1046/j.1462-2920.2000.00092.x

[24] Pinhassi J, Berman T (2003) Differential growth response of colony-forming α- and γ-proteobacteria in dilution culture and nutrient addition experiments from Lake Kinneret (Israel), the eastern Mediterranean Sea, and the Gulf of Eilat. Appl Environ Microbiol 69: 199–211. 12513996 10.1128/AEM.69.1.199-211.2003 PMC152472

[25] Beardsley C, Pernthaler J, Wosniok W, Amann R (2003) Are readily culturable bacteria in coastal North Sea waters suppressed by selective grazing mortality? Appl Environ Microbiol 69: 2624–2630. 12732530 10.1128/AEM.69.5.2624-2630.2003 PMC154555

[26] Eilers H, Pernthaler J, Glöckner FO, Amann R (2000) Culturability and in situ abundance of pelagic bacteria from the North Sea. Appl Environ Microbiol 66: 3044–3051. 10877804 10.1128/aem.66.7.3044-3051.2000 PMC92109

[27] Eilers H, Pernthaler J, Peplies J, Glöckner FO, Gerdts G, et al (2001) Isolation of novel pelagic bacteria from the German Bight and their seasonal contributions to surface picoplankton. Appl Environ Microbiol 67: 5134–5142. 11679337 10.1128/AEM.67.11.5134-5142.2001 PMC93282

[28] Gerdts G, Wichels A, Döpke H, Klings KW, Gunkel W, et al (2004) 40-year long-term study of microbial parameters near Helgoland (German Bight, North Sea): Historical view and future perspectives. Helgol Mar Res 58: 230–242.

[29] Sapp M, Wichels A, Wiltshire KH, Gerdts G (2007) Bacterial community dynamics during the winter-spring transition in the North Sea. FEMS Microbiol Ecol 59: 622–637. 17381518 10.1111/j.1574-6941.2006.00238.x

[30] Eilers H, Pernthaler J, Amann R (2000) Succession of pelagic marine bacteria during enrichment: A close look at cultivation-induced shifts. Appl Environ Microbiol 66: 4634–4640. 11055904 10.1128/aem.66.11.4634-4640.2000 PMC92360

[31] Gattuso JP, Lavigne H (2009) Technical note: Approaches and software tools to investigate the impact of ocean acidification. Biogeosciences 6: 2121–2133.

[32] Ranjard L, Brothier E, Nazaret S (2000) Sequencing bands of ribosomal intergenic spacer analysis fingerprints for characterization and microscale distribution of soil bacterium populations responding to mercury spiking. Appl Environ Microbiol 66: 5334–5339. 11097911 10.1128/aem.66.12.5334-5339.2000 PMC92465

[33] Brown MV, Schwalbach MS, Hewson I, Fuhrman JA (2005) Coupling 16S-ITS rDNA clone libraries and automated ribosomal intergenic spacer analysis to show marine microbial diversity: Development and application to a time series. Environ Microbiol 7: 1466–1479. 16104869 10.1111/j.1462-2920.2005.00835.x

[34] Teeling H, Fuchs BM, Becher D, Klockow C, Gardebrecht A, et al (2012) Substrate-controlled succession of marine bacterioplankton populations induced by a phytoplankton bloom. Science 336: 608–611. 22556258 10.1126/science.1218344

[35] Schloss PD, Westcott SL, Ryabin T, Hall JR, Hartmann M, et al (2009) Introducing mothur: Open-source, platform-independent, community-supported software for describing and comparing microbial communities. Appl Environ Microbiol 75: 7537–7541. 19801464 10.1128/AEM.01541-09 PMC2786419

[36] Underwood AJ (1996) Experiments in ecology - Their logical design and interpretation using analysis of variance. Camebridge: Camebridge University Press. 524 p.

[37] Anderson MJ (2001) A new method for non-parametric multivariate analysis of variance. Austral Ecol 26: 32–46.

[38] Legendre P, Legendre L (1998) Numerical ecology. Amsterdam: Elsevier Science B.V. 853 p.

[39] Giovannoni SJ, Stingl U (2005) Molecular diversity and ecology of microbial plankton. Nature 437: 343–348. 16163344 10.1038/nature04158

[40] Schulz KG, Barcelos e Ramos JBE, Zeebe RE, Riebesell U (2009) CO_2_ perturbation experiments: Similarities and differences between dissolved inorganic carbon and total alkalinity manipulations. Biogeosciences 6: 2145–2153.

[41] Hurd CL, Hepburn CD, Currie KI, Raven JA, Hunter KA (2009) Testing the effects of ocean acidification on algal metabolism: Considerations for experimental designs. J Phycol 45: 1236–1251. 27032579 10.1111/j.1529-8817.2009.00768.x

[42] Padan E, Bibi E, Ito M, Krulwich TA (2005) Alkaline pH homeostasis in bacteria: New insights. Biochim Biophys Acta 1717: 67–88. 16277975 10.1016/j.bbamem.2005.09.010 PMC3072713

[43] Teira E, Fernández A, Álvarez-Salgado XA, García-Martín EE, Serret P, et al (2012) Response of two marine bacterial isolates to high CO_2_ concentration. Mar Ecol Prog Ser 453: 27–36.

[44] Schneider K, Erez J (2006) The effect of carbonate chemistry on calcification and photosynthesis in the hermatypic coral *Acropora eurystoma* . Limnol Oceanogr 51: 1284–1293.

[45] Hoppe CJM, Langer G, Rost B (2011) *Emiliania huxleyi* shows identical responses to elevated pCO_2_ in TA and DIC manipulations. J Exp Mar Biol Ecol 406: 54–62.

[46] Fortunato CS, Herfort L, Zuber P, Baptista AM, Crump BC (2012) Spatial variability overwhelms seasonal patterns in bacterioplankton communities across a river to ocean gradient. ISME J 6: 554–563. 22011718 10.1038/ismej.2011.135 PMC3280145

[47] Lidbury I, Johnson V, Hall-Spencer JM, Munn CB, Cunliffe M (2012) Community-level response of coastal microbial biofilms to ocean acidification in a natural carbon dioxide vent ecosystem. Mar Pollut Bull 64: 1063–1066. 22414852 10.1016/j.marpolbul.2012.02.011

[48] Connell JH (1978) Diversity in tropical rain forests and coral reefs. Science 199: 1302–1310. 17840770 10.1126/science.199.4335.1302

[49] Atlas RM, Horowitz A, Krichevsky M, Bej AK (1991) Response of microbial populations to environmental disturbance. Microb Ecol 22: 249–256. 24194340 10.1007/BF02540227

[50] Crosby LD, Criddle CS (2003) Understanding bias in microbial community analysis techniques due to rrn operon copy number heterogeneity. Biotechniques 34: 790–802. 12703304 10.2144/03344rr01

[51] Kovacs A, Yacoby K, Gophna U (2010) A systematic assessment of automated ribosomal intergenic spacer analysis (ARISA) as a tool for estimating bacterial richness. Res Microbiol 161: 192–197. 20138144 10.1016/j.resmic.2010.01.006

[52] Kunin V, Engelbrektson A, Ochman H, Hugenholtz P (2010) Wrinkles in the rare biosphere: Pyrosequencing errors can lead to artificial inflation of diversity estimates. Environ Microbiol 12: 118–123. 19725865 10.1111/j.1462-2920.2009.02051.x

[53] Schloss PD, Gevers D, Westcott SL (2011) Reducing the effects of PCR amplification and sequencing artifacts on 16S rRNA-based studies. PloS One 6: e27310. 22194782 10.1371/journal.pone.0027310 PMC3237409

[54] Grossart HP, Allgaier M, Passow U, Riebesell U (2006) Testing the effect of CO_2_ concentration on the dynamics of marine heterotrophic bacterioplankton. Limnol Oceanogr 51: 1–11.

[55] Shi D, Xu Y, Hopkinson BM, Morel FMM (2010) Effect of ocean acidification on iron availability to marine phytoplankton. Science 327: 676–679. 20075213 10.1126/science.1183517

[56] Raven J, Caldeira K, Elderfield H, Hoegh-Guldberg O, Liss P, et al (2005) Ocean acidification due to increasing atmospheric carbon dioxide. Policy Document 12/05 Roy Soc Rep 12.

[57] Zeebe RE, Wolf-Gladrow D (2001) CO_2_ in seawater: Equilibrium, kinetics, isotopes. Amsterdam: Elsevier. 346 p.

[58] Doney SC, Fabry VJ, Feely RA, Kleypas JA (2009) Ocean acidification: The other CO_2_ problem. Ann Rev Mar Sci 1: 169–192. 10.1146/annurev.marine.010908.163834 21141034

[59] Piontek J, Lunau M, Händel N, Borchard C, Wurst M, et al (2010) Acidification increases microbial polysaccharide degradation in the ocean. Biogeosciences 7: 1615–1624.

[60] Yamada N, Suzumura M (2010) Effects of seawater acidification on hydrolytic enzyme activities. J Oceanogr 66: 233–241.

[61] Goldman JC, Dennett MR (1985) Susceptibility of some marine-phytoplankton species to cell breakage during filtration and post-filtration rinsing. J Exp Mar Bio Ecol 86: 47–58.

[62] Danovaro R, Corinaldesi C, Dell'Anno A, Fuhrman JA, Middelburg JJ, et al (2011) Marine viruses and global climate change. FEMS Microbiol Rev 35: 993–1034. 21204862 10.1111/j.1574-6976.2010.00258.x

[63] Thompson FL, Iida T, Jean Swings J (2004) Biodiversity of Vibrios. Microbiol Mol Biol Rev 68: 403–431. 15353563 10.1128/MMBR.68.3.403-431.2004 PMC515257

[64] Baker-Austin C, Stockley L, Rangdale R, Martinez-Urtaza J (2010) Environmental occurrence and clinical impact of *Vibrio vulnificus* and *Vibrio parahaemolyticus*: A European perspective. Environ Microbiol Rep 2: 7–18. 23765993 10.1111/j.1758-2229.2009.00096.x

[65] Colwell R (1996) Global climate and infectious disease: The cholera paradigm. Science 274: 2025–2031. 8953025 10.1126/science.274.5295.2025

[66] Oberbeckmann S, Wichels A, Wiltshire KH, Gerdts G (2011) Occurrence of *Vibrio parahaemolyticus* and *Vibrio alginolyticus* in the German Bight over a seasonal cycle. Antonie Van Leeuwenhoek 100: 291–307. 21598011 10.1007/s10482-011-9586-x

[67] Paz S, Bisharat N, Paz E, Kidar O, Cohen D (2007) Climate change and the emergence of *Vibrio vulnificus* disease in Israel. Environ Res 103: 390–396. 16949069 10.1016/j.envres.2006.07.002

[68] Collado L, Inza I, Guarro J, Figueras MJ (2008) Presence of *Arcobacter* spp. in environmental waters correlates with high levels of fecal pollution. Environ Microbiol 10: 1635–1640. 18215159 10.1111/j.1462-2920.2007.01555.x

[69] Snelling WJ, Matsuda M, Moore JE, Dooley JSG (2006) Under the microscope: *Arcobacter* . Lett Appl Microbiol 42: 7–14. 16411912 10.1111/j.1472-765X.2005.01841.x

[70] Collado L, Figueras MJ (2011) Taxonomy, epidemiology, and clinical relevance of the genus *Arcobacter* . Clin Microbiol Rev 24: 174–192. 21233511 10.1128/CMR.00034-10 PMC3021208

[71] D'Sa EM, Harrison MA (2005) Effect of pH, NaCl content, and temperature on growth and survival of *Arcobacter* spp. J Food Protect 68: 18–25. 10.4315/0362-028x-68.1.18 15690799

[72] Reid AN, Pandey R, Palyada K, Naikare H, Stintzi A (2008) Identification of *Campylobacter jejuni* genes involved in the response to acidic pH and stomach transit. Appl Environ Microbiol 74: 1583–1597. 18192414 10.1128/AEM.01507-07 PMC2258634

[73] Murphy C, Carroll C, Jordan KN (2006) Environmental survival mechanisms of the foodborne pathogen *Campylobacter jejuni* . J Appl Microbiol 100: 623–632. 16553716 10.1111/j.1365-2672.2006.02903.x

[74] Freitas S, Hatosy S, Fuhrman JA, Huse SM, Mark Welch DB, et al (2012) Global distribution and diversity of marine *Verrucomicrobia* . ISME J 6: 1499–1505. 22318305 10.1038/ismej.2012.3 PMC3400412

[75] Pernthaler J, Amann R (2005) Fate of heterotrophic microbes in pelagic habitats: Focus on populations. Microbiol Mol Biol Rev 69: 440–461. 16148306 10.1128/MMBR.69.3.440-461.2005 PMC1197807

[76] Wiltshire KH, Malzahn AM, Wirtz K, Greve W, Janisch S, et al (2008) Resilience of North Sea phytoplankton spring bloom dynamics: An analysis of long-term data at Helgoland Roads. Limnol Oceanogr 53: 1294–1302.

