# Supplementary figures and images for: Small Changes in pH Have Direct Effects on Marine Bacterial Community Composition: A Microcosm Approach
Source: PLoS One. 2012 Oct 11;7(10):e47035. doi: 10.1371/journal.pone.0047035 (PMC3469576; doi:10.1371/journal.pone.0047035)

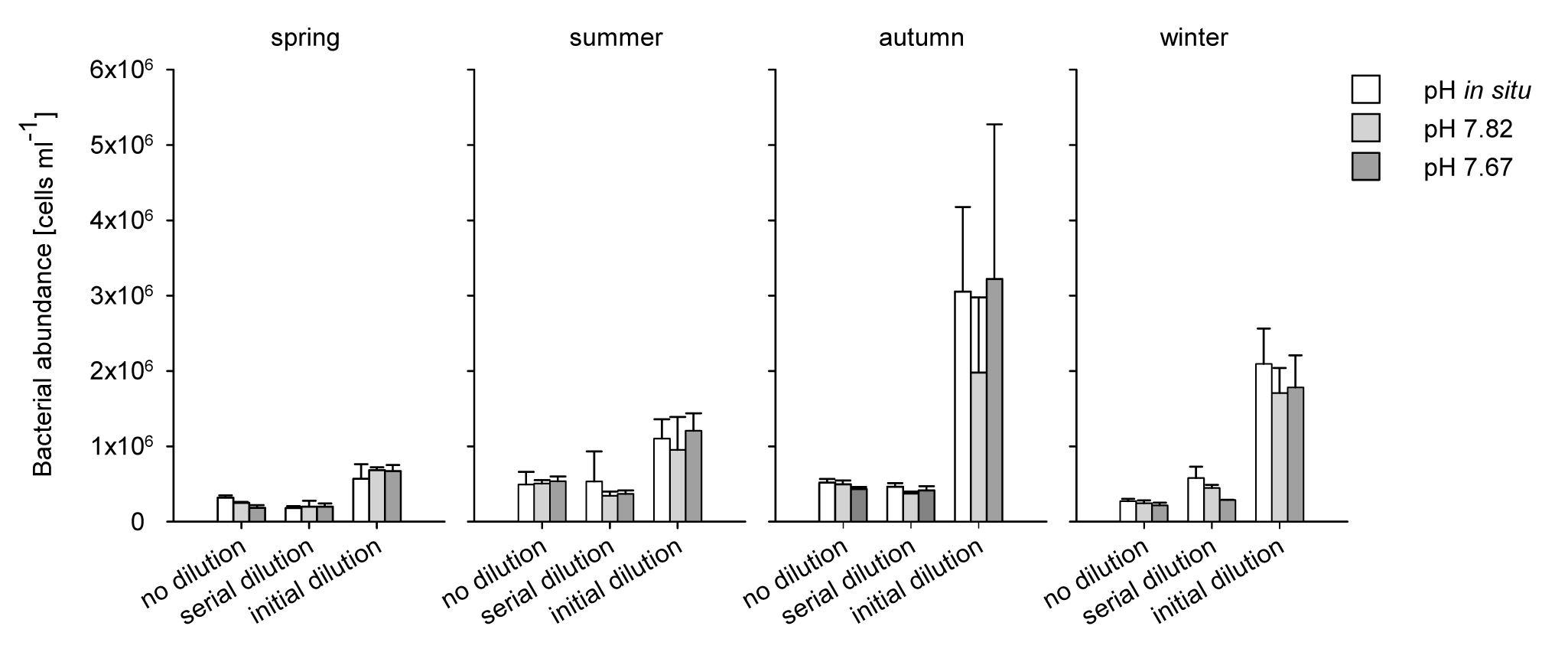

Supplement: Figure S1 — Bacterial abundance in the different treatments. No significant pH effect on bacterial abundance was found (ANOVA). (TIF) [file pone.0047035.s001.tif]

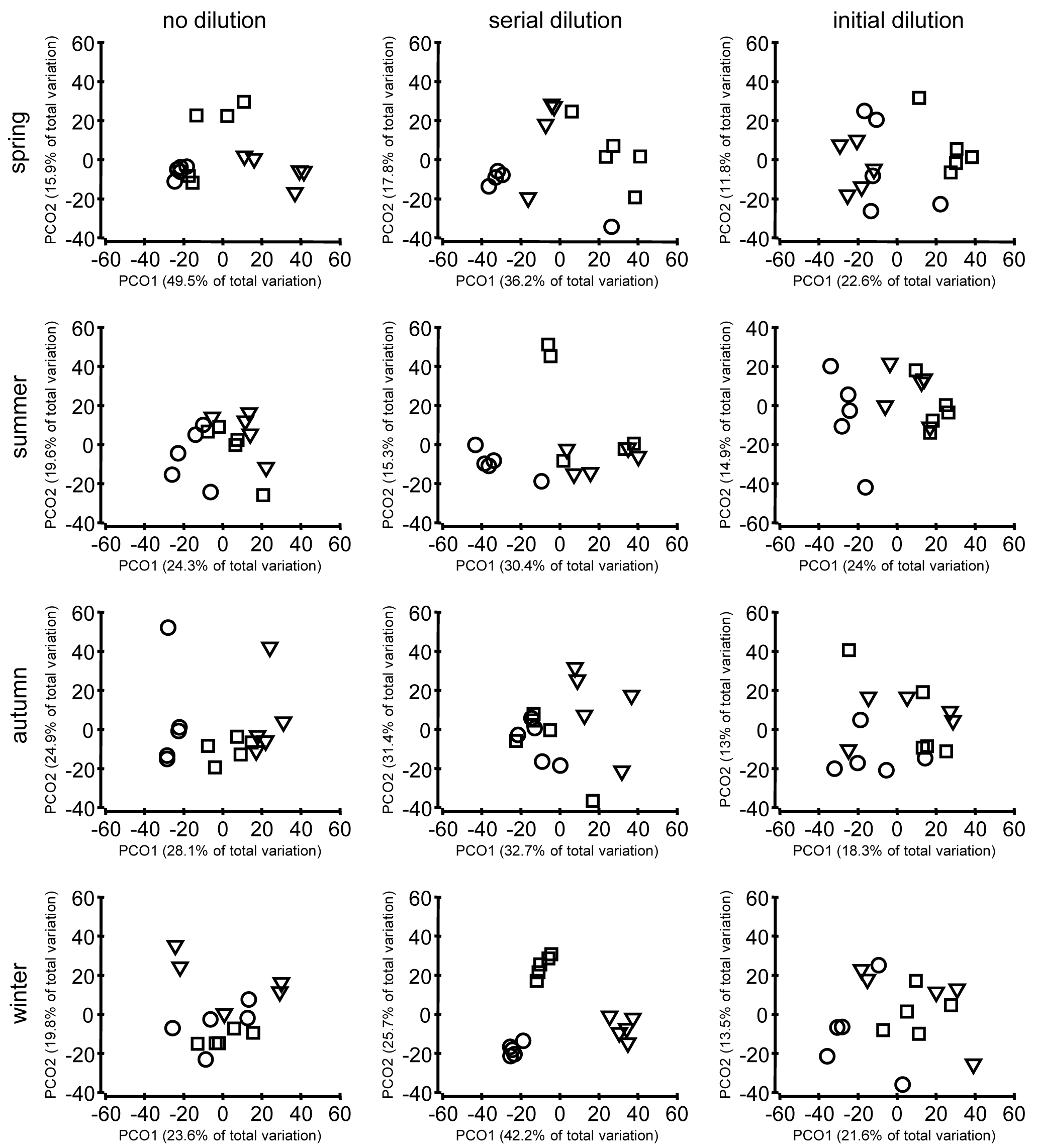

Supplement: Figure S2 — Influence of the factor ‘pH’ on bacterial community composition (ARISA). Displayed are principal co-ordinate analysis plots (PCOs) for each ‘season’-‘dilution’ combination based on Jaccard dissimilarities of ARISA profiles. Symbol shape represents the pH level (circles: pH in situ, squares: pH 7.82, triangles: pH 7.67). (TIF) [file pone.0047035.s002.tif]

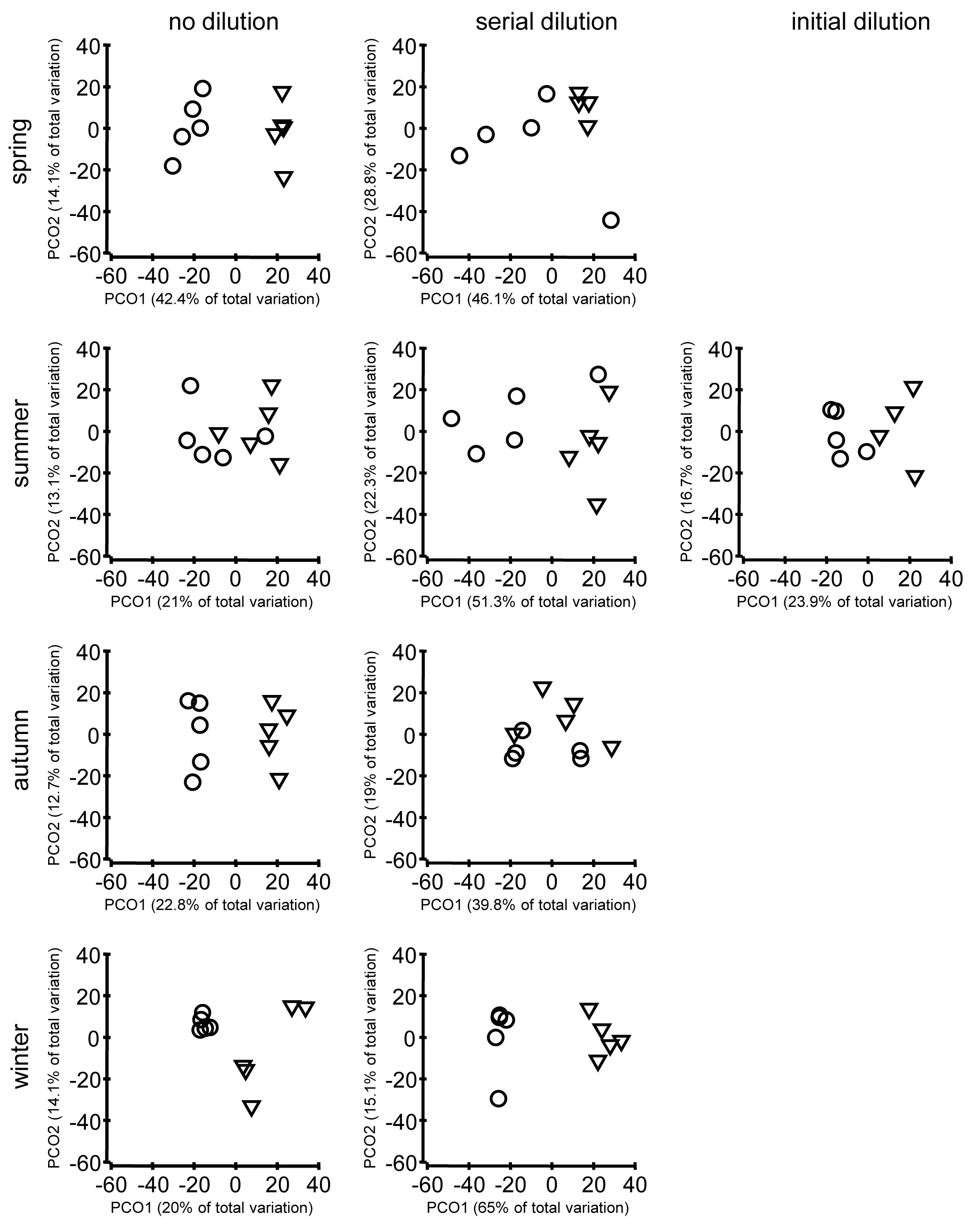

Supplement: Figure S3 — Influence of the factor ‘pH’ on bacterial community composition (16S ribosomal amplicon pyrosequencing). Displayed are principal co-ordinate analysis plots (PCOs) for each ‘season’-‘dilution’ combination based on Bray-Curtis dissimilarities of OTUs (16S ribosomal amplicon pyrosequencing). Symbol shape represents the pH level (circles: pH in situ, triangles: pH 7.67). (TIF) [file pone.0047035.s003.tif]

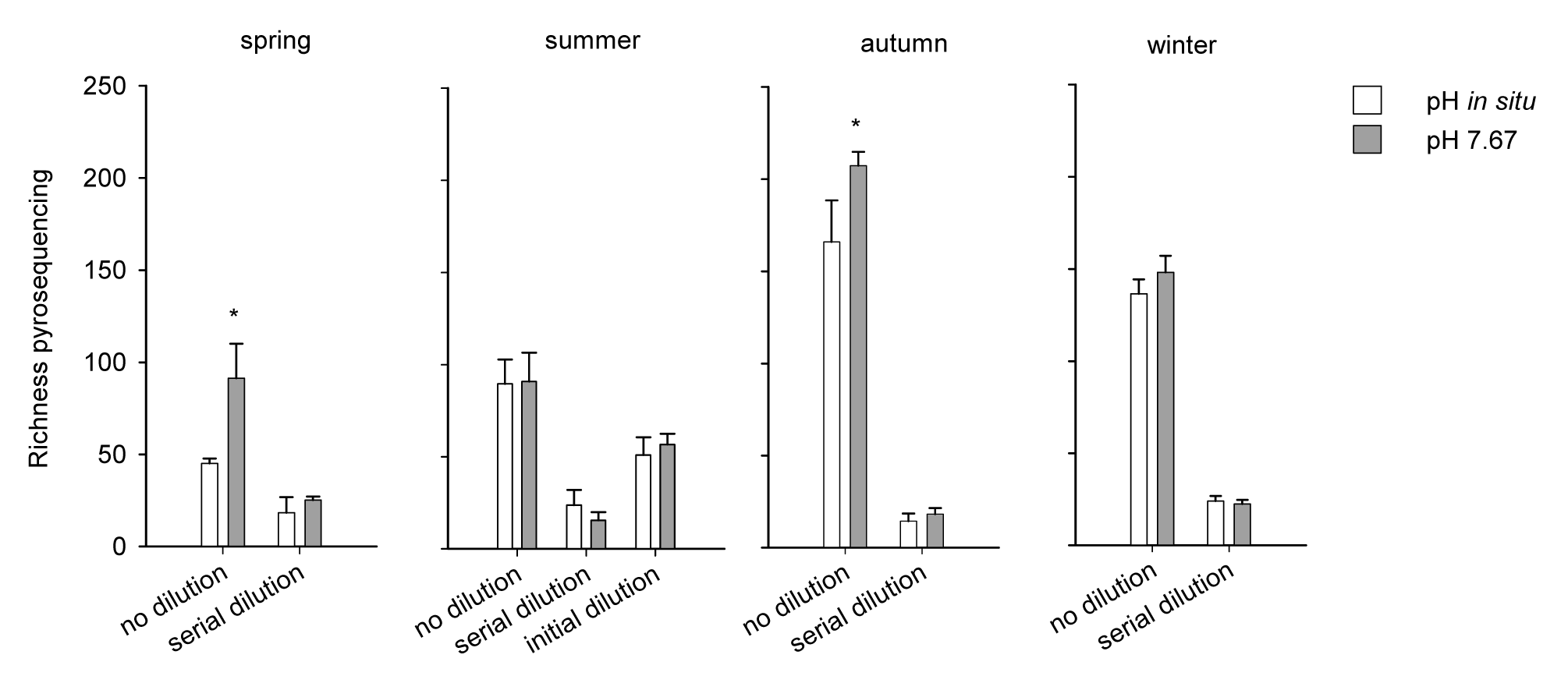

Supplement: Figure S4 — Bacterial richness in the different treatments. Richness was determined based on the number of OTUs (16S ribosomal amplicon pyrosequencing) in the standardized subsample (n = 494 sequences) and was compared by ANOVA, with Tukey's HSD test for post-hoc comparisons. Asterisks (*) represent significant differences (p<0.05) between pH levels within a ‘season’-‘dilution’ combination. (TIF) [file pone.0047035.s004.tif]
