# Supplementary material for: Small Changes in pH Have Direct Effects on Marine Bacterial Community Composition: A Microcosm Approach
Source: PLoS One. 2012 Oct 11;7(10):e47035. doi: 10.1371/journal.pone.0047035 (PMC3469576; doi:10.1371/journal.pone.0047035)
Supplement: Table S1 — pH after the four weeks of incubation. (PDF) [file pone.0047035.s005.pdf]

Table S1. pH after the four weeks of incubation.

season	dilution	pH *in situ*	pH 7.82	pH 7.67
spring	no dilution	8.20 ± 0.00	7.79 ± 0.00	7.64 ± 0.01
	serial dilution	8.04 ± 0.01	7.90 ± 0.01	7.75 ± 0.03
	initial dilution	8.08 ± 0.01	7.56 ± 0.02	7.44 ± 0.03
summer	no dilution	8.05 ± 0.00	7.61 ± 0.01	7.49 ± 0.00
	serial dilution	8.21 ± 0.01	7.94 ± 0.03	7.84 ± 0.01
	initial dilution	7.27 ± 0.03	7.06 ± 0.05	6.93 ± 0.03
autumn	no dilution	8.06 ± 0.00	7.71 ± 0.01	7.59 ± 0.00
	serial dilution	8.08 ± 0.01	7.79 ± 0.01	7.72 ± 0.02
	initial dilution	7.22 ± 0.05	6.96 ± 0.03	6.95 ± 0.04
winter	no dilution	8.10 ± 0.01	7.75 ± 0.01	7.63 ± 0.01
	serial dilution	8.16 ± 0.01	7.89 ± 0.02	7.78 ± 0.02
	initial dilution	7.33 ± 0.10	7.24 ± 0.02	7.24 ± 0.01

Given are the means of five replicate incubations ± standard deviation. Starting values for the pH *in situ* treatment were 8.26 (spring), 8.22 (summer), 8.15 (autumn) and 8.19 (winter), respectively.
