# Supplementary material for: Small Changes in pH Have Direct Effects on Marine Bacterial Community Composition: A Microcosm Approach
Source: PLoS One. 2012 Oct 11;7(10):e47035. doi: 10.1371/journal.pone.0047035 (PMC3469576; doi:10.1371/journal.pone.0047035)
Supplement: Table S2 — Sample coverage obtained by 16S ribosomal amplicon pyrosequencing, based on the standardized subsample (n = 494 sequences). (PDF) [file pone.0047035.s006.pdf]

Table S2. Sample coverage obtained by 16S ribosomal amplicon pyrosequencing, based on the standardized subsample (n=494 sequences).

**Sample**		**spring**	**summer**	**autumn**	**winter**
d0		92.5%	95.1%	83.2%	76.5%
'no dilution'	pH *in situ*	94.9%	90.3%	76.5%	82.6%
		95.3%	90.7%	79.4%	81.2%
		95.7%	90.5%	84.2%	84.6%
		96.2%	88.3%	83.0%	81.4%
		97.0%	85.2%	76.5%	82.0%
	pH 7.67	94.5%	87.9%	72.3%	80.4%
		88.1%	92.5%	72.1%	77.5%
		89.7%	89.9%	72.7%	82.8%
		90.1%	87.7%	76.3%	80.8%
		89.5%	90.7%	74.7%	80.0%
'serial dilution'	pH *in situ*	99.6%	98.0%	99.2%	97.6%
		98.4%	99.2%	99.8%	98.2%
		96.8%	97.6%	98.4%	98.4%
		99.4%	97.8%	98.2%	97.8%
		98.4%	98.4%	98.4%	96.8%
	pH 7.67	98.6%	98.0%	98.2%	98.6%
		98.6%	97.6%	99.4%	99.2%
		99.0%	98.2%	98.8%	98.8%
		98.6%	99.4%	98.4%	98.4%
		-	98.8%	98.6%	97.6%
'initial dilution'	pH *in situ*	-	95.3%	-	-
		-	96.0%	-	-
		-	94.7%	-	-
		-	93.7%	-	-
		-	92.5%	-	-
	pH 7.67	-	93.9%	-	-
		-	94.1%	-	-
		-	-	-	-
		-	95.5%	-	-
		-	95.3%	-	-
