# Supplementary material for: Small Changes in pH Have Direct Effects on Marine Bacterial Community Composition: A Microcosm Approach
Source: PLoS One. 2012 Oct 11;7(10):e47035. doi: 10.1371/journal.pone.0047035 (PMC3469576; doi:10.1371/journal.pone.0047035)
Supplement: Table S3 — Results of the SIMPER analysis giving the similarities within ‘season’-‘dilution’-‘pH’ combinations. Displayed are the OTUs (16S ribosomal amplicon pyrosequencing) that predominantly contributed to 90% of to the total similarity. Av.Ai: average abundance of the i th species over all samples of the treatment, Av.Si: average contribution of the i th species to the total similarity, Av.Si/SD: the average value of the i th species as a typifying species, Av.Si%: average percentage contribution of the i th species to the total similarity, ∑Av.Si%: average cumulative contribution to the total similarity. (PDF) [file pone.0047035.s007.pdf]

Table S3. Results of the SIMPER analysis giving the similarities within ‘season’-‘dilution’-‘pH’ combinations.

Displayed are the OTUs (16S ribosomal amplicon pyrosequencing) that predominantly contributed to 90% of to the total similarity. $Av.A_i$: average abundance of the i th species over all samples of the treatment, $Av.S_i$: average contribution of the i th species to the total similarity, $Av.S_i/SD$: the average value of the i th species as a typifying species, $Av.S_i\%$: average percentage contribution of the i th species to the total similarity, $\sum Av.S_i\%$: average cumulative contribution to the total similarity.

OTU	$Av.A_i$	$Av.S_i$	$Av.S_i/SD$	$Av.S_i\%$	$\sum Av.S_i\%$	Genus	Family	Order	Class	Phylum	Domain
Spring 'no dilution' pH *in situ* (average similarity: 60.6%)											
Otu0005	9.45	7.11	2.22	11.74	11.74	*Pelagibacter*	SAR11-clade	*Rickettsiales*	*Alphaproteobacteria*	*Proteobacteria*	*Bacteria*
Otu0134	7.21	5.58	2.89	9.22	20.96	unclass. *Gammaproteobacteria*	unclass. *Gammaproteobacteria*	unclass. *Gammaproteobacteria*	*Gammaproteobacteria*	*Proteobacteria*	*Bacteria*
Otu0029	5.69	4.63	10.95	7.64	28.6	unclass. *Bacteroidetes*	unclass. *Bacteroidetes*	unclass. *Bacteroidetes*	unclass. *Bacteroidetes*	*Bacteroidetes*	*Bacteria*
Otu0022	5.16	4.3	7.95	7.09	35.69	unclass. *Betaproteobacteria*	unclass. *Betaproteobacteria*	unclass. *Betaproteobacteria*	*Betaproteobacteria*	*Proteobacteria*	*Bacteria*
Otu0008	5.12	4.22	6.59	6.97	42.66	unclass. *Gammaproteobacteria*	unclass. *Gammaproteobacteria*	unclass. *Gammaproteobacteria*	*Gammaproteobacteria*	*Proteobacteria*	*Bacteria*
Otu0002	6.43	4.05	1.78	6.69	49.35	unclass. *Flavobacteriaceae*	*Flavobacteriaceae*	*Flavobacteriales*	*Flavobacteria*	*Bacteroidetes*	*Bacteria*
Otu0012	4.52	3.42	2.19	5.65	54.99	unclass. *Comamonadaceae*	*Comamonadaceae*	*Burkholderiales*	*Betaproteobacteria*	*Proteobacteria*	*Bacteria*
Otu0001	6.06	3.35	1.34	5.54	60.53	unclass. *Flavobacteriaceae*	*Flavobacteriaceae*	*Flavobacteriales*	*Flavobacteria*	*Bacteroidetes*	*Bacteria*
Otu0135	3.24	2.33	2.31	3.84	64.37	unclass. *Gammaproteobacteria*	unclass. *Gammaproteobacteria*	unclass. *Gammaproteobacteria*	*Gammaproteobacteria*	*Proteobacteria*	*Bacteria*
Otu0014	3.76	2.21	1.43	3.64	68.02	*Reinekea*	*Oceanospirillaceae*	*Oceanospirillales*	*Gammaproteobacteria*	*Proteobacteria*	*Bacteria*
Otu0007	3.2	2.16	1.16	3.57	71.58	unclass. *Flavobacteriales*	unclass. *Flavobacteriales*	*Flavobacteriales*	*Flavobacteria*	*Bacteroidetes*	*Bacteria*
Otu0013	2.74	1.87	3.99	3.09	74.67	unclass. *Flavobacteriaceae*	*Flavobacteriaceae*	*Flavobacteriales*	*Flavobacteria*	*Bacteroidetes*	*Bacteria*
Otu0010	3.32	1.83	1.5	3.02	77.69	unclass. *Flavobacteriaceae*	*Flavobacteriaceae*	*Flavobacteriales*	*Flavobacteria*	*Bacteroidetes*	*Bacteria*
Otu0018	1.91	1.36	8.42	2.24	79.93	unclass. *Puniceicoccaceae*	*Puniceicoccaceae*	*Puniceicoccales*	*Opitutae*	*Verrucomicrobia*	*Bacteria*
Otu0044	1.63	1.26	3.99	2.08	82	unclass. *Gammaproteobacteria*	unclass. *Gammaproteobacteria*	unclass. *Gammaproteobacteria*	*Gammaproteobacteria*	*Proteobacteria*	*Bacteria*
Otu0056	1.59	1.15	3.06	1.9	83.9	unclass. *Gammaproteobacteria*	unclass. *Gammaproteobacteria*	unclass. *Gammaproteobacteria*	*Gammaproteobacteria*	*Proteobacteria*	*Bacteria*

OTU	Av.A_i	Av.S_i	Av.S_i/SD	Av.S_i%	∑Av.S_i%	Genus	Family	Order	Class	Phylum	Domain
Otu0052	1.49	1.04	5.72	1.71	85.61	unclass. *Betaproteobacteria*	unclass. *Betaproteobacteria*	unclass. *Betaproteobacteria*	*Betaproteobacteria*	*Proteobacteria*	*Bacteria*
Otu0041	1.76	0.96	1.06	1.59	87.2	*Sulfitobacter*	*Rhodobacteraceae*	*Rhodobacterales*	*Alphaproteobacteria*	*Proteobacteria*	*Bacteria*
Otu0020	1.78	0.96	0.96	1.58	88.78	unclass. *Flammeovirgaceae*	*Flammeovirgaceae*	*Sphingobacteriales*	*Sphingobacteria*	*Bacteroidetes*	*Bacteria*
Otu0139	1.76	0.96	1.12	1.58	90.36	unclass. *Gammaproteobacteria*	unclass. *Gammaproteobacteria*	unclass. *Gammaproteobacteria*	*Gammaproteobacteria*	*Proteobacteria*	*Bacteria*
Spring 'no dilution' pH 7.67 (average similarity: 57.1%)											
Otu0002	7.01	3.65	3.39	6.4	6.4	unclass. *Flavobacteriaceae*	*Flavobacteriaceae*	*Flavobacteriales*	*Flavobacteria*	*Bacteroidetes*	*Bacteria*
Otu0018	5.96	3.4	11.57	5.95	12.34	unclass. *Puniceicoccaceae*	*Puniceicoccaceae*	*Puniceicoccales*	*Opitutae*	*Verrucomicrobia*	*Bacteria*
Otu0013	5.62	3.33	9.8	5.83	18.17	unclass. *Flavobacteriaceae*	*Flavobacteriaceae*	*Flavobacteriales*	*Flavobacteria*	*Bacteroidetes*	*Bacteria*
Otu0134	5.85	3.08	2.4	5.39	23.56	unclass. *Gammaproteobacteria*	unclass. *Gammaproteobacteria*	unclass. *Gammaproteobacteria*	*Gammaproteobacteria*	*Proteobacteria*	*Bacteria*
Otu0010	5.77	2.84	3.21	4.98	28.54	unclass. *Flavobacteriaceae*	*Flavobacteriaceae*	*Flavobacteriales*	*Flavobacteria*	*Bacteroidetes*	*Bacteria*
Otu0056	4.87	2.81	8.75	4.93	33.47	unclass. *Gammaproteobacteria*	unclass. *Gammaproteobacteria*	unclass. *Gammaproteobacteria*	*Gammaproteobacteria*	*Proteobacteria*	*Bacteria*
Otu0005	4.62	2.14	3.84	3.74	37.21	*Pelagibacter*	SAR11-clade	*Rickettsiales*	*Alphaproteobacteria*	*Proteobacteria*	*Bacteria*
Otu0001	4.13	2.13	3.64	3.72	40.94	unclass. *Flavobacteriaceae*	*Flavobacteriaceae*	*Flavobacteriales*	*Flavobacteria*	*Bacteroidetes*	*Bacteria*
Otu0139	3.91	2.06	8.22	3.61	44.54	unclass. *Gammaproteobacteria*	unclass. *Gammaproteobacteria*	unclass. *Gammaproteobacteria*	*Gammaproteobacteria*	*Proteobacteria*	*Bacteria*
Otu0007	4.03	1.82	3	3.19	47.74	unclass. *Flavobacteriales*	unclass. *Flavobacteriales*	*Flavobacteriales*	*Flavobacteria*	*Bacteroidetes*	*Bacteria*
Otu0014	3.88	1.61	2.4	2.82	50.56	*Reinekea*	*Oceanospirillaceae*	*Oceanospirillales*	*Gammaproteobacteria*	*Proteobacteria*	*Bacteria*
Otu0055	2.31	1.31	8.79	2.29	52.85	*Colwellia*	*Colwelliaceae*	*Alteromonadales*	*Gammaproteobacteria*	*Proteobacteria*	*Bacteria*
Otu0041	2.71	1.29	3.45	2.26	55.11	*Sulfitobacter*	*Rhodobacteraceae*	*Rhodobacterales*	*Alphaproteobacteria*	*Proteobacteria*	*Bacteria*
Otu0025	2.74	1.17	1.72	2.05	57.16	*Polaribacter*	*Flavobacteriaceae*	*Flavobacteriales*	*Flavobacteria*	*Bacteroidetes*	*Bacteria*
Otu0151	2.27	1.14	5.09	2	59.16	*Rhodococcus*	*Nocardiaceae*	*Actinomycetales*	*Actinobacteria*	*Actinobacteria*	*Bacteria*
Otu0019	2.32	1.13	3.82	1.99	61.15	unclass. *Gammaproteobacteria*	unclass. *Gammaproteobacteria*	unclass. *Gammaproteobacteria*	*Gammaproteobacteria*	*Proteobacteria*	*Bacteria*
Otu0022	2.41	1.09	3.14	1.91	63.06	unclass. *Betaproteobacteria*	unclass. *Betaproteobacteria*	unclass. *Betaproteobacteria*	*Betaproteobacteria*	*Proteobacteria*	*Bacteria*
Otu0143	2.11	1.01	2.97	1.78	64.84	unclass. *Gammaproteobacteria*	unclass. *Gammaproteobacteria*	unclass. *Gammaproteobacteria*	*Gammaproteobacteria*	*Proteobacteria*	*Bacteria*
Otu0135	1.86	0.96	6.21	1.67	66.51	unclass. *Gammaproteobacteria*	unclass. *Gammaproteobacteria*	unclass. *Gammaproteobacteria*	*Gammaproteobacteria*	*Proteobacteria*	*Bacteria*
Otu0232	1.66	0.92	6.15	1.6	68.11	unclass. *Rhodobacteraceae*	*Rhodobacteraceae*	*Rhodobacterales*	*Alphaproteobacteria*	*Proteobacteria*	*Bacteria*
Otu0020	2.15	0.91	2.63	1.59	69.7	unclass. *Flammeovirgaceae*	*Flammeovirgaceae*	*Sphingobacteriales*	*Sphingobacteria*	*Bacteroidetes*	*Bacteria*
Otu0035	1.91	0.89	3.71	1.55	71.25	*Colwellia*	*Colwelliaceae*	*Alteromonadales*	*Gammaproteobacteria*	*Proteobacteria*	*Bacteria*

OTU	Av.A$_i$	Av.S$_i$	Av.S$_i$/SD	Av.S$_i$%	∑Av.S$_i$%	Genus	Family	Order	Class	Phylum	Domain
Otu0044	1.81	0.84	3.12	1.48	72.73	unclass. *Gammaproteobacteria*	unclass. *Gammaproteobacteria*	unclass. *Gammaproteobacteria*	*Gammaproteobacteria*	*Proteobacteria*	*Bacteria*
Otu0144	1.49	0.74	3.27	1.29	74.02	*Lewinella*	*Saprospiraceae*	*Sphingobacteriales*	*Sphingobacteria*	*Bacteroidetes*	*Bacteria*
Otu0062	1.93	0.7	0.92	1.22	75.25	*Haliea*	*Alteromonadaceae*	*Alteromonadales*	*Gammaproteobacteria*	*Proteobacteria*	*Bacteria*
Otu0070	1.43	0.69	6.01	1.21	76.45	*Winogradskyella*	*Flavobacteriaceae*	*Flavobacteriales*	*Flavobacteria*	*Bacteroidetes*	*Bacteria*
Otu0169	1.17	0.63	7.02	1.1	77.55	unclass. *Bacteria*	unclass. *Bacteria*	unclass. *Bacteria*	unclass. *Bacteria*	unclass. *Bacteria*	*Bacteria*
Otu0037	1.08	0.6	12.32	1.06	78.61	*Marinobacter*	*Alteromonadaceae*	*Alteromonadales*	*Gammaproteobacteria*	*Proteobacteria*	*Bacteria*
Otu0006	1.36	0.55	1.15	0.97	79.58	*Polaribacter*	*Flavobacteriaceae*	*Flavobacteriales*	*Flavobacteria*	*Bacteroidetes*	*Bacteria*
Otu0092	1.31	0.53	1.16	0.93	80.51	unclass. *Bacteroidetes*	unclass. *Bacteroidetes*	unclass. *Bacteroidetes*	unclass. *Bacteroidetes*	*Bacteroidetes*	*Bacteria*
Otu0012	1.48	0.5	1.03	0.87	81.38	unclass. *Comamonadaceae*	*Comamonadaceae*	*Burkholderiales*	*Betaproteobacteria*	*Proteobacteria*	*Bacteria*
Otu0160	1.34	0.47	1.09	0.82	82.2	*Colwellia*	*Colwelliaceae*	*Alteromonadales*	*Gammaproteobacteria*	*Proteobacteria*	*Bacteria*
Otu0145	1.17	0.41	1.13	0.72	82.92	unclass. *Gammaproteobacteria*	unclass. *Gammaproteobacteria*	unclass. *Gammaproteobacteria*	*Gammaproteobacteria*	*Proteobacteria*	*Bacteria*
Otu0213	0.97	0.39	1.12	0.68	83.6	unclass. *Gammaproteobacteria*	unclass. *Gammaproteobacteria*	unclass. *Gammaproteobacteria*	*Gammaproteobacteria*	*Proteobacteria*	*Bacteria*
Otu0150	0.97	0.39	1.14	0.68	84.28	unclass. *Bacteria*	unclass. *Bacteria*	unclass. *Bacteria*	unclass. *Bacteria*	unclass. *Bacteria*	*Bacteria*
Otu0148	1.08	0.38	1.14	0.67	84.95	unclass. *Sphingobacteriales*	unclass. *Sphingobacteriales*	*Sphingobacteriales*	*Sphingobacteria*	*Bacteroidetes*	*Bacteria*
Otu0119	0.95	0.37	1.15	0.65	85.59	*Colwellia*	*Colwelliaceae*	*Alteromonadales*	*Gammaproteobacteria*	*Proteobacteria*	*Bacteria*
Otu0174	0.88	0.36	1.15	0.64	86.23	unclass. *Flavobacteriaceae*	*Flavobacteriaceae*	*Flavobacteriales*	*Flavobacteria*	*Bacteroidetes*	*Bacteria*
Otu0042	0.95	0.34	1.16	0.6	86.83	*Rhodococcus*	*Nocardiaceae*	*Actinomycetales*	*Actinobacteria*	*Actinobacteria*	*Bacteria*
Otu0146	0.95	0.34	1.16	0.6	87.43	*Reichenbachiella*	*Flammeovirgaceae*	*Sphingobacteriales*	*Sphingobacteria*	*Bacteroidetes*	*Bacteria*
Otu0187	0.8	0.34	1.16	0.6	88.04	*Lentisphaera*	*Lentisphaeraceae*	*Lentisphaerales*	*Lentisphaeria*	*Lentisphaerae*	*Bacteria*
Otu0237	0.95	0.34	1.16	0.6	88.64	unclass. *Flavobacteriaceae*	*Flavobacteriaceae*	*Flavobacteriales*	*Flavobacteria*	*Bacteroidetes*	*Bacteria*
Otu0149	0.77	0.22	0.61	0.39	89.03	unclass. *Gammaproteobacteria*	unclass. *Gammaproteobacteria*	unclass. *Gammaproteobacteria*	*Gammaproteobacteria*	*Proteobacteria*	*Bacteria*
Otu0177	0.83	0.22	0.61	0.39	89.42	*Haliea*	*Alteromonadaceae*	*Alteromonadales*	*Gammaproteobacteria*	*Proteobacteria*	*Bacteria*
Otu0029	1.39	0.21	0.59	0.38	89.8	unclass. *Bacteroidetes*	unclass. *Bacteroidetes*	unclass. *Bacteroidetes*	unclass. *Bacteroidetes*	*Bacteroidetes*	*Bacteria*
Otu0009	0.77	0.21	0.62	0.37	90.17	unclass. *Rhodobacteraceae*	*Rhodobacteraceae*	*Rhodobacterales*	*Alphaproteobacteria*	*Proteobacteria*	*Bacteria*
Spring 'serial dilution' pH *in situ* (average similarity: 48.0%)											
Otu0027	11.23	11.78	1.38	24.55	24.55	*Pseudoalteromonas*	*Pseudoalteromonadaceae*	*Alteromonadales*	*Gammaproteobacteria*	*Proteobacteria*	*Bacteria*
Otu0106	7.05	9.25	1.46	19.29	43.85	*Marinomonas*	*Oceanospirillaceae*	*Oceanospirillales*	*Gammaproteobacteria*	*Proteobacteria*	*Bacteria*

OTU	Av.A_i	Av.S_i	Av.S_i/SD	Av.S_i%	∑Av.S_i%	Genus	Family	Order	Class	Phylum	Domain
Otu0035	9.32	7.97	0.9	16.62	60.46	*Colwellia*	*Colwelliaceae*	*Alteromonadales*	*Gammaproteobacteria*	*Proteobacteria*	*Bacteria*
Otu0107	4.07	5.41	3.34	11.28	71.74	unclass. *Rhodobacteraceae*	*Rhodobacteraceae*	*Rhodobacterales*	*Alphaproteobacteria*	*Proteobacteria*	*Bacteria*
Otu0036	3.68	4.31	1.82	8.98	80.73	*Glaciecola*	*Alteromonadaceae*	*Alteromonadales*	*Gammaproteobacteria*	*Proteobacteria*	*Bacteria*
Otu0111	2.28	2.27	0.87	4.74	85.46	*Marinomonas*	*Oceanospirillaceae*	*Oceanospirillales*	*Gammaproteobacteria*	*Proteobacteria*	*Bacteria*
Otu0108	1.4	1.02	1.12	2.12	87.58	unclass. *Colwelliaceae*	*Colwelliaceae*	*Alteromonadales*	*Gammaproteobacteria*	*Proteobacteria*	*Bacteria*
Otu0113	1.95	0.83	0.51	1.73	89.31	*Colwellia*	*Colwelliaceae*	*Alteromonadales*	*Gammaproteobacteria*	*Proteobacteria*	*Bacteria*
Otu0132	1.08	0.79	0.61	1.64	90.95	unclass. *Alteromonadales*	unclass. *Alteromonadales*	*Alteromonadales*	*Gammaproteobacteria*	*Proteobacteria*	*Bacteria*
Spring 'serial dilution' pH 7.67 (average similarity: 67.4%)											
Otu0027	18.57	25.53	13.36	37.87	37.87	*Pseudoalteromonas*	*Pseudoalteromonadaceae*	*Alteromonadales*	*Gammaproteobacteria*	*Proteobacteria*	*Bacteria*
Otu0106	4.44	5	3.5	7.42	45.29	*Marinomonas*	*Oceanospirillaceae*	*Oceanospirillales*	*Gammaproteobacteria*	*Proteobacteria*	*Bacteria*
Otu0109	3.81	4.5	14.57	6.67	51.95	*Alteromonas*	*Alteromonadaceae*	*Alteromonadales*	*Gammaproteobacteria*	*Proteobacteria*	*Bacteria*
Otu0039	4.41	4.28	3.46	6.36	58.31	*Pseudoalteromonas*	*Pseudoalteromonadaceae*	*Alteromonadales*	*Gammaproteobacteria*	*Proteobacteria*	*Bacteria*
Otu0111	3	3.11	3.37	4.62	62.93	*Marinomonas*	*Oceanospirillaceae*	*Oceanospirillales*	*Gammaproteobacteria*	*Proteobacteria*	*Bacteria*
Otu0114	2.57	3.03	4.57	4.5	67.43	*Shewanella*	*Shewanellaceae*	*Alteromonadales*	*Gammaproteobacteria*	*Proteobacteria*	*Bacteria*
Otu0059	2.27	2.64	3.95	3.92	71.35	*Flavobacterium*	*Flavobacteriaceae*	*Flavobacteriales*	*Flavobacteria*	*Bacteroidetes*	*Bacteria*
Otu0113	2.22	2.6	3.32	3.86	75.21	*Colwellia*	*Colwelliaceae*	*Alteromonadales*	*Gammaproteobacteria*	*Proteobacteria*	*Bacteria*
Otu0115	2.07	2.48	5.26	3.68	78.89	*Glaciecola*	*Alteromonadaceae*	*Alteromonadales*	*Gammaproteobacteria*	*Proteobacteria*	*Bacteria*
Otu0117	1.87	1.96	3.23	2.9	81.79	*Arcobacter*	*Campylobacteraceae*	*Campylobacterales*	*Epsilonproteobacteria*	*Proteobacteria*	*Bacteria*
Otu0035	2.06	1.76	0.9	2.61	84.39	*Colwellia*	*Colwelliaceae*	*Alteromonadales*	*Gammaproteobacteria*	*Proteobacteria*	*Bacteria*
Otu0120	1.29	1.52	6.43	2.26	86.65	*Pseudoalteromonas*	*Pseudoalteromonadaceae*	*Alteromonadales*	*Gammaproteobacteria*	*Proteobacteria*	*Bacteria*
Otu0107	2.41	1.45	0.77	2.16	88.81	unclass. *Rhodobacteraceae*	*Rhodobacteraceae*	*Rhodobacterales*	*Alphaproteobacteria*	*Proteobacteria*	*Bacteria*
Otu0036	1.53	1.22	0.91	1.81	90.62	*Glaciecola*	*Alteromonadaceae*	*Alteromonadales*	*Gammaproteobacteria*	*Proteobacteria*	*Bacteria*
Summer 'no dilution' pH *in situ* (average similarity: 47.9%)											
Otu0005	15.27	10.06	9.45	21	21	*Pelagibacter*	SAR11-clade	*Rickettsiales*	*Alphaproteobacteria*	*Proteobacteria*	*Bacteria*
Otu0691	5.44	3.02	4.19	6.31	27.31	*Planctomyces*	*Planctomycetaceae*	*Planctomycetales*	*Planctomycetacia*	*Planctomycetes*	*Bacteria*
Otu0001	4.58	2.74	5.4	5.72	33.03	unclass. *Flavobacteriaceae*	*Flavobacteriaceae*	*Flavobacteriales*	*Flavobacteria*	*Bacteroidetes*	*Bacteria*
Otu0003	3.21	2.12	13.52	4.42	37.45	unclass. *Rhodobacteraceae*	*Rhodobacteraceae*	*Rhodobacterales*	*Alphaproteobacteria*	*Proteobacteria*	*Bacteria*

OTU	$Av.A_i$	$Av.S_i$	$Av.S_i/SD$	$Av.S_i\%$	$\sum Av.S_i\%$	Genus	Family	Order	Class	Phylum	Domain
Otu0052	2.91	1.76	7.89	3.67	41.12	unclass. *Betaproteobacteria*	unclass. *Betaproteobacteria*	unclass. *Betaproteobacteria*	*Betaproteobacteria*	*Proteobacteria*	*Bacteria*
Otu0203	3.14	1.67	3.31	3.49	44.61	*Pelagibacter*	SAR11-clade	*Rickettsiales*	*Alphaproteobacteria*	*Proteobacteria*	*Bacteria*
Otu0693	3.22	1.58	2.54	3.29	47.9	*Planctomyces*	*Planctomycetaceae*	*Planctomycetales*	*Planctomycetacia*	*Planctomycetes*	*Bacteria*
Otu0007	2.06	1.23	4.26	2.58	50.48	unclass. *Flavobacteriales*	unclass. *Flavobacteriales*	*Flavobacteriales*	*Flavobacteria*	*Bacteroidetes*	*Bacteria*
Otu0706	1.99	1.16	6.04	2.42	52.9	unclass. *Planctomycetaceae*	*Planctomycetaceae*	*Planctomycetales*	*Planctomycetacia*	*Planctomycetes*	*Bacteria*
Otu0692	2.21	1.11	2.28	2.32	55.22	unclass. *Bacteria*	unclass. *Bacteria*	unclass. *Bacteria*	unclass. *Bacteria*	unclass. *Bacteria*	*Bacteria*
Otu0461	1.89	0.9	4.06	1.87	57.09	unclass. *Microbacteriaceae*	*Microbacteriaceae*	*Actinomycetales*	*Actinobacteria*	*Actinobacteria*	*Bacteria*
Otu0068	1.51	0.89	4.97	1.85	58.94	*Pelagibacter*	SAR11-clade	*Rickettsiales*	*Alphaproteobacteria*	*Proteobacteria*	*Bacteria*
Otu0519	1.72	0.82	3.81	1.71	60.65	unclass. *Rhodospirillaceae*	*Rhodospirillaceae*	*Rhodospirillales*	*Alphaproteobacteria*	*Proteobacteria*	*Bacteria*
Otu0463	1.43	0.8	4.36	1.67	62.32	unclass. *Flavobacteriaceae*	*Flavobacteriaceae*	*Flavobacteriales*	*Flavobacteria*	*Bacteroidetes*	*Bacteria*
Otu0473	1.63	0.8	4.85	1.67	63.99	unclass. *Alphaproteobacteria*	unclass. *Alphaproteobacteria*	unclass. *Alphaproteobacteria*	*Alphaproteobacteria*	*Proteobacteria*	*Bacteria*
Otu0019	1.33	0.69	18.39	1.45	65.44	unclass. *Gammaproteobacteria*	unclass. *Gammaproteobacteria*	unclass. *Gammaproteobacteria*	*Gammaproteobacteria*	*Proteobacteria*	*Bacteria*
Otu0521	1.08	0.69	18.39	1.45	66.88	unclass. *Rhodobacteraceae*	*Rhodobacteraceae*	*Rhodobacterales*	*Alphaproteobacteria*	*Proteobacteria*	*Bacteria*
Otu0495	1.53	0.63	1.14	1.31	68.19	unclass. *Flavobacteriales*	unclass. *Flavobacteriales*	*Flavobacteriales*	*Flavobacteria*	*Bacteroidetes*	*Bacteria*
Otu0512	1.58	0.6	0.95	1.24	69.44	*Rhodopirellula*	*Planctomycetaceae*	*Planctomycetales*	*Planctomycetacia*	*Planctomycetes*	*Bacteria*
Otu0046	1.29	0.59	1.08	1.23	70.67	unclass. *Flavobacteriaceae*	*Flavobacteriaceae*	*Flavobacteriales*	*Flavobacteria*	*Bacteroidetes*	*Bacteria*
Otu0157	1.37	0.56	1.08	1.17	71.84	unclass. *Gammaproteobacteria*	unclass. *Gammaproteobacteria*	unclass. *Gammaproteobacteria*	*Gammaproteobacteria*	*Proteobacteria*	*Bacteria*
Otu0450	1.66	0.52	0.94	1.09	72.93	*Sphingobium*	*Sphingomonadaceae*	*Sphingomonadales*	*Alphaproteobacteria*	*Proteobacteria*	*Bacteria*
Otu0469	1.52	0.51	1.1	1.07	74	unclass. *Bacteria*	unclass. *Bacteria*	unclass. *Bacteria*	unclass. *Bacteria*	unclass. *Bacteria*	*Bacteria*
Otu0354	1.11	0.49	1.12	1.03	75.03	*Anderseniella*	*Rhodobiaceae*	*Rhizobiales*	*Alphaproteobacteria*	*Proteobacteria*	*Bacteria*
Otu0053	1.29	0.47	1.06	0.99	76.02	unclass. *Rhodobacteraceae*	*Rhodobacteraceae*	*Rhodobacterales*	*Alphaproteobacteria*	*Proteobacteria*	*Bacteria*
Otu0186	0.97	0.46	1.13	0.97	76.98	unclass. *Planctomycetaceae*	*Planctomycetaceae*	*Planctomycetales*	*Planctomycetacia*	*Planctomycetes*	*Bacteria*
Otu0704	0.97	0.46	1.14	0.96	77.95	unclass. *Proteobacteria*	unclass. *Proteobacteria*	unclass. *Proteobacteria*	unclass. *Proteobacteria*	*Proteobacteria*	*Bacteria*
Otu0503	1.15	0.46	1.1	0.95	78.9	unclass. *Bacteroidetes*	unclass. *Bacteroidetes*	unclass. *Bacteroidetes*	unclass. *Bacteroidetes*	*Bacteroidetes*	*Bacteria*
Otu0502	1.03	0.44	1.14	0.93	79.83	unclass. *Gammaproteobacteria*	unclass. *Gammaproteobacteria*	unclass. *Gammaproteobacteria*	*Gammaproteobacteria*	*Proteobacteria*	*Bacteria*
Otu0703	1.03	0.44	1.13	0.92	80.74	unclass. *Gammaproteobacteria*	unclass. *Gammaproteobacteria*	unclass. *Gammaproteobacteria*	*Gammaproteobacteria*	*Proteobacteria*	*Bacteria*
Otu0245	0.97	0.44	1.12	0.91	81.65	unclass. *Flavobacteriales*	unclass. *Flavobacteriales*	*Flavobacteriales*	*Flavobacteria*	*Bacteroidetes*	*Bacteria*

OTU	Av.A_i	Av.S_i	Av.S_i/SD	Av.S_i%	∑Av.S_i%	Genus	Family	Order	Class	Phylum	Domain
Otu0010	0.88	0.41	1.16	0.85	82.51	unclass. *Flavobacteriaceae*	*Flavobacteriaceae*	*Flavobacteriales*	*Flavobacteria*	*Bacteroidetes*	*Bacteria*
Otu0705	1.19	0.38	0.62	0.79	83.3	*Planctomyces*	*Planctomycetaceae*	*Planctomycetales*	*Planctomycetacia*	*Planctomycetes*	*Bacteria*
Otu0289	1.08	0.26	0.58	0.53	83.83	unclass. *Bacteroidetes*	unclass. *Bacteroidetes*	unclass. *Bacteroidetes*	unclass. *Bacteroidetes*	*Bacteroidetes*	*Bacteria*
Otu0701	0.88	0.23	0.6	0.49	84.32	unclass. *Bacteria*	unclass. *Bacteria*	unclass. *Bacteria*	unclass. *Bacteria*	unclass. *Bacteria*	*Bacteria*
Otu0084	0.93	0.23	0.6	0.48	84.8	unclass. *Gammaproteobacteria*	unclass. *Gammaproteobacteria*	unclass. *Gammaproteobacteria*	*Gammaproteobacteria*	*Proteobacteria*	*Bacteria*
Otu0532	0.77	0.23	0.6	0.48	85.28	unclass. *Rhodobacteraceae*	*Rhodobacteraceae*	*Rhodobacterales*	*Alphaproteobacteria*	*Proteobacteria*	*Bacteria*
Otu0261	0.77	0.23	0.61	0.48	85.77	*Winogradskyella*	*Flavobacteriaceae*	*Flavobacteriales*	*Flavobacteria*	*Bacteroidetes*	*Bacteria*
Otu0142	0.68	0.22	0.62	0.46	86.22	unclass. *Gammaproteobacteria*	unclass. *Gammaproteobacteria*	unclass. *Gammaproteobacteria*	*Gammaproteobacteria*	*Proteobacteria*	*Bacteria*
Otu0504	0.68	0.22	0.62	0.46	86.68	unclass. *Alphaproteobacteria*	unclass. *Alphaproteobacteria*	unclass. *Alphaproteobacteria*	*Alphaproteobacteria*	*Proteobacteria*	*Bacteria*
Otu0697	0.6	0.22	0.62	0.46	87.14	unclass. *Flavobacteriaceae*	*Flavobacteriaceae*	*Flavobacteriales*	*Flavobacteria*	*Bacteroidetes*	*Bacteria*
Otu0714	0.68	0.22	0.62	0.46	87.59	unclass. *Alphaproteobacteria*	unclass. *Alphaproteobacteria*	unclass. *Alphaproteobacteria*	*Alphaproteobacteria*	*Proteobacteria*	*Bacteria*
Otu0696	0.68	0.21	0.62	0.45	88.04	unclass. *Flavobacteriaceae*	*Flavobacteriaceae*	*Flavobacteriales*	*Flavobacteria*	*Bacteroidetes*	*Bacteria*
Otu0695	1.12	0.21	0.62	0.43	88.48	unclass. *Proteobacteria*	unclass. *Proteobacteria*	unclass. *Proteobacteria*	unclass. *Proteobacteria*	*Proteobacteria*	*Bacteria*
Otu0041	0.75	0.2	0.62	0.42	88.9	*Sulfitobacter*	*Rhodobacteraceae*	*Rhodobacterales*	*Alphaproteobacteria*	*Proteobacteria*	*Bacteria*
Otu0408	0.75	0.2	0.62	0.42	89.32	unclass. *Flavobacteriaceae*	*Flavobacteriaceae*	*Flavobacteriales*	*Flavobacteria*	*Bacteroidetes*	*Bacteria*
Otu0694	0.75	0.2	0.62	0.42	89.75	unclass. *Alphaproteobacteria*	unclass. *Alphaproteobacteria*	unclass. *Alphaproteobacteria*	*Alphaproteobacteria*	*Proteobacteria*	*Bacteria*
Otu0476	0.6	0.2	0.62	0.42	90.17	unclass. *Actinomycetales*	unclass. *Actinomycetales*	*Actinomycetales*	*Actinobacteria*	*Actinobacteria*	*Bacteria*
Summer 'no dilution' pH 7.67 (average similarity: 49.0%)											
Otu0005	15.85	10.4	7.53	21.23	21.23	*Pelagibacter*	SAR11-clade	*Rickettsiales*	*Alphaproteobacteria*	*Proteobacteria*	*Bacteria*
Otu0469	3.64	2.13	3.02	4.34	25.57	unclass. *Bacteria*	unclass. *Bacteria*	unclass. *Bacteria*	unclass. *Bacteria*	unclass. *Bacteria*	*Bacteria*
Otu0691	2.99	1.88	3.55	3.84	29.41	*Planctomyces*	*Planctomycetaceae*	*Planctomycetales*	*Planctomycetacia*	*Planctomycetes*	*Bacteria*
Otu0001	3.07	1.54	2.63	3.14	32.55	unclass. *Flavobacteriaceae*	*Flavobacteriaceae*	*Flavobacteriales*	*Flavobacteria*	*Bacteroidetes*	*Bacteria*
Otu0203	3.03	1.46	2.25	2.98	35.54	*Pelagibacter*	SAR11-clade	*Rickettsiales*	*Alphaproteobacteria*	*Proteobacteria*	*Bacteria*
Otu0084	2.23	1.43	19.17	2.92	38.45	unclass. *Gammaproteobacteria*	unclass. *Gammaproteobacteria*	unclass. *Gammaproteobacteria*	*Gammaproteobacteria*	*Proteobacteria*	*Bacteria*
Otu0010	2.58	1.38	4.35	2.82	41.27	unclass. *Flavobacteriaceae*	*Flavobacteriaceae*	*Flavobacteriales*	*Flavobacteria*	*Bacteroidetes*	*Bacteria*
Otu0692	2.58	1.37	4.75	2.8	44.07	unclass. *Bacteria*	unclass. *Bacteria*	unclass. *Bacteria*	unclass. *Bacteria*	unclass. *Bacteria*	*Bacteria*
Otu0693	2.46	1.33	6.27	2.72	46.79	*Planctomyces*	*Planctomycetaceae*	*Planctomycetales*	*Planctomycetacia*	*Planctomycetes*	*Bacteria*

OTU	$Av.A_i$	$Av.S_i$	$Av.S_i/SD$	$Av.S_i\%$	$\sum Av.S_i\%$	Genus	Family	Order	Class	Phylum	Domain
Otu0003	2.58	1.32	2	2.7	49.49	unclass. *Rhodobacteraceae*	*Rhodobacteraceae*	*Rhodobacterales*	*Alphaproteobacteria*	*Proteobacteria*	*Bacteria*
Otu0052	2.27	1.31	4.23	2.67	52.15	unclass. *Betaproteobacteria*	unclass. *Betaproteobacteria*	unclass. *Betaproteobacteria*	*Betaproteobacteria*	*Proteobacteria*	*Bacteria*
Otu0706	1.88	1.05	3.37	2.14	54.29	unclass. *Planctomycetaceae*	*Planctomycetaceae*	*Planctomycetales*	*Planctomycetacia*	*Planctomycetes*	*Bacteria*
Otu0519	1.95	1.03	8.88	2.11	56.4	unclass. *Rhodospirillaceae*	*Rhodospirillaceae*	*Rhodospirillales*	*Alphaproteobacteria*	*Proteobacteria*	*Bacteria*
Otu0002	1.81	0.91	2.51	1.85	58.25	unclass. *Flavobacteriaceae*	*Flavobacteriaceae*	*Flavobacteriales*	*Flavobacteria*	*Bacteroidetes*	*Bacteria*
Otu0779	2.48	0.88	1.09	1.8	60.05	unclass. *Ectothiorhodospiraceae*	*Ectothiorhodospiraceae*	*Chromatiales*	*Gammaproteobacteria*	*Proteobacteria*	*Bacteria*
Otu0277	1.56	0.87	5.66	1.78	61.83	unclass. *Flavobacteriaceae*	*Flavobacteriaceae*	*Flavobacteriales*	*Flavobacteria*	*Bacteroidetes*	*Bacteria*
Otu0354	1.38	0.79	4.42	1.61	63.45	*Anderseniella*	*Rhodobiaceae*	*Rhizobiales*	*Alphaproteobacteria*	*Proteobacteria*	*Bacteria*
Otu0695	1.38	0.79	4.92	1.61	65.06	unclass. *Proteobacteria*	unclass. *Proteobacteria*	unclass. *Proteobacteria*	unclass. *Proteobacteria*	*Proteobacteria*	*Bacteria*
Otu0473	1.46	0.77	5.74	1.57	66.63	unclass. *Alphaproteobacteria*	unclass. *Alphaproteobacteria*	unclass. *Alphaproteobacteria*	*Alphaproteobacteria*	*Proteobacteria*	*Bacteria*
Otu0710	1.31	0.77	5.88	1.57	68.2	unclass. *Gammaproteobacteria*	unclass. *Gammaproteobacteria*	unclass. *Gammaproteobacteria*	*Gammaproteobacteria*	*Proteobacteria*	*Bacteria*
Otu0503	1.41	0.76	8.69	1.56	69.75	unclass. *Bacteroidetes*	unclass. *Bacteroidetes*	unclass. *Bacteroidetes*	unclass. *Bacteroidetes*	*Bacteroidetes*	*Bacteria*
Otu0461	1.45	0.75	4.31	1.53	71.28	unclass. *Microbacteriaceae*	*Microbacteriaceae*	*Actinomycetales*	*Actinobacteria*	*Actinobacteria*	*Bacteria*
Otu0046	1.44	0.69	1.16	1.4	72.68	unclass. *Flavobacteriaceae*	*Flavobacteriaceae*	*Flavobacteriales*	*Flavobacteria*	*Bacteroidetes*	*Bacteria*
Otu0352	1.47	0.66	1.13	1.35	74.03	unclass. *Flavobacteriaceae*	*Flavobacteriaceae*	*Flavobacteriales*	*Flavobacteria*	*Bacteroidetes*	*Bacteria*
Otu0053	1.25	0.56	1.16	1.15	75.17	unclass. *Rhodobacteraceae*	*Rhodobacteraceae*	*Rhodobacterales*	*Alphaproteobacteria*	*Proteobacteria*	*Bacteria*
Otu0495	1.11	0.5	1.13	1.02	76.19	unclass. *Flavobacteriales*	unclass. *Flavobacteriales*	*Flavobacteriales*	*Flavobacteria*	*Bacteroidetes*	*Bacteria*
Otu0060	1.08	0.45	1.09	0.92	77.11	unclass. *Actinomycetales*	unclass. *Actinomycetales*	*Actinomycetales*	*Actinobacteria*	*Actinobacteria*	*Bacteria*
Otu0004	1.09	0.45	1.05	0.91	78.03	unclass. *Flavobacteriales*	unclass. *Flavobacteriales*	*Flavobacteriales*	*Flavobacteria*	*Bacteroidetes*	*Bacteria*
Otu0752	0.8	0.43	1.16	0.88	78.9	unclass. *Gammaproteobacteria*	unclass. *Gammaproteobacteria*	unclass. *Gammaproteobacteria*	*Gammaproteobacteria*	*Proteobacteria*	*Bacteria*
Otu0502	1.13	0.43	1.12	0.87	79.77	unclass. *Gammaproteobacteria*	unclass. *Gammaproteobacteria*	unclass. *Gammaproteobacteria*	*Gammaproteobacteria*	*Proteobacteria*	*Bacteria*
Otu0463	1.37	0.4	0.62	0.82	80.59	unclass. *Flavobacteriaceae*	*Flavobacteriaceae*	*Flavobacteriales*	*Flavobacteria*	*Bacteroidetes*	*Bacteria*
Otu0704	0.88	0.4	1.16	0.81	81.4	unclass. *Proteobacteria*	unclass. *Proteobacteria*	unclass. *Proteobacteria*	unclass. *Proteobacteria*	*Proteobacteria*	*Bacteria*
Otu0708	0.8	0.4	1.16	0.81	82.2	unclass. *Proteobacteria*	unclass. *Proteobacteria*	unclass. *Proteobacteria*	unclass. *Proteobacteria*	*Proteobacteria*	*Bacteria*
Otu0480	0.98	0.34	0.62	0.69	82.89	*Ilumatobacter*	*Acidimicrobidae_incertae_sedis*	*Actinobacteria_order_incertae_sedis*	*Actinobacteria*	*Actinobacteria*	*Bacteria*
Otu0007	1.13	0.32	0.61	0.66	83.55	unclass. *Flavobacteriales*	unclass. *Flavobacteriales*	*Flavobacteriales*	*Flavobacteria*	*Bacteroidetes*	*Bacteria*
Otu0245	0.98	0.3	0.62	0.62	84.17	unclass. *Flavobacteriales*	unclass. *Flavobacteriales*	*Flavobacteriales*	*Flavobacteria*	*Bacteroidetes*	*Bacteria*

OTU	Av.A_i	Av.S_i	Av.S_i/SD	Av.S_i%	∑Av.S_i%	Genus	Family	Order	Class	Phylum	Domain
Otu0450	1.2	0.28	0.62	0.58	84.74	*Sphingobium*	*Sphingomonadaceae*	*Sphingomonadales*	*Alphaproteobacteria*	*Proteobacteria*	*Bacteria*
Otu0490	0.91	0.28	0.62	0.58	85.32	unclass. *Burkholderiales*	unclass. *Burkholderiales*	*Burkholderiales*	*Betaproteobacteria*	*Proteobacteria*	*Bacteria*
Otu0512	0.89	0.26	0.58	0.53	85.85	*Rhodopirellula*	*Planctomycetaceae*	*Planctomycetales*	*Planctomycetacia*	*Planctomycetes*	*Bacteria*
Otu0142	0.83	0.25	0.61	0.51	86.36	unclass. *Gammaproteobacteria*	unclass. *Gammaproteobacteria*	unclass. *Gammaproteobacteria*	*Gammaproteobacteria*	*Proteobacteria*	*Bacteria*
Otu0261	0.99	0.23	0.58	0.48	86.84	*Winogradskyella*	*Flavobacteriaceae*	*Flavobacteriales*	*Flavobacteria*	*Bacteroidetes*	*Bacteria*
Otu0521	0.77	0.23	0.61	0.48	87.31	unclass. *Rhodobacteraceae*	*Rhodobacteraceae*	*Rhodobacterales*	*Alphaproteobacteria*	*Proteobacteria*	*Bacteria*
Otu0468	0.88	0.23	0.6	0.47	87.78	unclass. *Flavobacteriaceae*	*Flavobacteriaceae*	*Flavobacteriales*	*Flavobacteria*	*Bacteroidetes*	*Bacteria*
Otu0759	0.77	0.23	0.61	0.47	88.25	unclass. *Verrucomicrobiaceae*	*Verrucomicrobiaceae*	*Verrucomicrobiales*	*Verrucomicrobiae*	*Verrucomicrobia*	*Bacteria*
Otu0510	0.77	0.23	0.61	0.47	88.71	unclass. *Gammaproteobacteria*	unclass. *Gammaproteobacteria*	unclass. *Gammaproteobacteria*	*Gammaproteobacteria*	*Proteobacteria*	*Bacteria*
Otu0782	0.77	0.23	0.62	0.46	89.18	unclass. *Gammaproteobacteria*	unclass. *Gammaproteobacteria*	unclass. *Gammaproteobacteria*	*Gammaproteobacteria*	*Proteobacteria*	*Bacteria*
Otu0347	0.6	0.22	0.62	0.45	89.63	unclass. *Flavobacteriaceae*	*Flavobacteriaceae*	*Flavobacteriales*	*Flavobacteria*	*Bacteroidetes*	*Bacteria*
Otu0019	0.6	0.22	0.62	0.45	90.08	unclass. *Gammaproteobacteria*	unclass. *Gammaproteobacteria*	unclass. *Gammaproteobacteria*	*Gammaproteobacteria*	*Proteobacteria*	*Bacteria*
Summer 'serial dilution' pH *in situ* (average similarity: 47.1%)											
Otu0132	11.11	9.97	1.14	21.16	21.16	unclass. *Alteromonadales*	unclass. *Alteromonadales*	*Alteromonadales*	*Gammaproteobacteria*	*Proteobacteria*	*Bacteria*
Otu0459	10.06	9.08	1.31	19.28	40.44	*Croceibacter*	*Flavobacteriaceae*	*Flavobacteriales*	*Flavobacteria*	*Bacteroidetes*	*Bacteria*
Otu0471	6.52	7.15	1.74	15.18	55.63	*Oceaniserpentilla*	*Oceanospirillaceae*	*Oceanospirillales*	*Gammaproteobacteria*	*Proteobacteria*	*Bacteria*
Otu0125	4.15	3.1	1.06	6.59	62.21	*Leeuwenhoekiella*	*Flavobacteriaceae*	*Flavobacteriales*	*Flavobacteria*	*Bacteroidetes*	*Bacteria*
Otu0521	4.58	2.85	1.09	6.05	68.26	unclass. *Rhodobacteraceae*	*Rhodobacteraceae*	*Rhodobacterales*	*Alphaproteobacteria*	*Proteobacteria*	*Bacteria*
Otu0525	2.2	2.11	1.79	4.48	72.74	*Reinekea*	*Oceanospirillaceae*	*Oceanospirillales*	*Gammaproteobacteria*	*Proteobacteria*	*Bacteria*
Otu0523	2.3	1.6	1.01	3.39	76.13	*Pseudidiomarina*	*Idiomarinaceae*	*Alteromonadales*	*Gammaproteobacteria*	*Proteobacteria*	*Bacteria*
Otu0524	1.85	1.51	1.13	3.21	79.35	*Pseudidiomarina*	*Idiomarinaceae*	*Alteromonadales*	*Gammaproteobacteria*	*Proteobacteria*	*Bacteria*
Otu0460	3.42	1.51	0.82	3.21	82.56	*Arcobacter*	*Campylobacteraceae*	*Campylobacterales*	*Epsilonproteobacteria*	*Proteobacteria*	*Bacteria*
Otu0522	1.75	1.4	1.14	2.97	85.53	unclass. *Flavobacteriaceae*	*Flavobacteriaceae*	*Flavobacteriales*	*Flavobacteria*	*Bacteroidetes*	*Bacteria*
Otu0531	1.7	1.16	1.1	2.46	87.99	unclass. *Flavobacteriaceae*	*Flavobacteriaceae*	*Flavobacteriales*	*Flavobacteria*	*Bacteroidetes*	*Bacteria*
Otu0537	1.38	1.08	1.14	2.29	90.28	unclass. *Flavobacteriaceae*	*Flavobacteriaceae*	*Flavobacteriales*	*Flavobacteria*	*Bacteroidetes*	*Bacteria*
Summer 'serial dilution' pH 7.67 (average similarity: 63.1%)											
Otu0471	13.99	21.82	3.37	34.59	34.59	*Oceaniserpentilla*	*Oceanospirillaceae*	*Oceanospirillales*	*Gammaproteobacteria*	*Proteobacteria*	*Bacteria*

OTU	Av.A$_i$	Av.S$_i$	Av.S$_i$/SD	Av.S$_i$%	∑Av.S$_i$%	Genus	Family	Order	Class	Phylum	Domain
Otu0132	13.17	19.12	2.01	30.31	64.9	unclass. *Alteromonadales*	unclass. *Alteromonadales*	*Alteromonadales*	*Gammaproteobacteria*	*Proteobacteria*	*Bacteria*
Otu0525	6.66	10.29	4.22	16.31	81.2	*Reinekea*	*Oceanospirillaceae*	*Oceanospirillales*	*Gammaproteobacteria*	*Proteobacteria*	*Bacteria*
Otu0533	2.33	3.16	6.16	5.01	86.22	*Nisaea*	*Rhodospirillaceae*	*Rhodospirillales*	*Alphaproteobacteria*	*Proteobacteria*	*Bacteria*
Otu0180	1.45	2.12	3.85	3.36	89.57	*Reichenbachiella*	*Flammeovirgaceae*	*Sphingobacteriales*	*Sphingobacteria*	*Bacteroidetes*	*Bacteria*
Otu0531	0.88	1.17	1.15	1.85	91.43	unclass. *Flavobacteriaceae*	*Flavobacteriaceae*	*Flavobacteriales*	*Flavobacteria*	*Bacteroidetes*	*Bacteria*
Summer 'initial dilution' pH *in situ* (average similarity: 55.6%)											
Otu0109	18.3	18.69	9.04	33.63	33.63	*Alteromonas*	*Alteromonadaceae*	*Alteromonadales*	*Gammaproteobacteria*	*Proteobacteria*	*Bacteria*
Otu0035	3.47	3.14	3.93	5.64	39.28	*Colwellia*	*Colwelliaceae*	*Alteromonadales*	*Gammaproteobacteria*	*Proteobacteria*	*Bacteria*
Otu0041	3.93	3.09	4.81	5.56	44.84	*Sulfitobacter*	*Rhodobacteraceae*	*Rhodobacterales*	*Alphaproteobacteria*	*Proteobacteria*	*Bacteria*
Otu0521	3.2	2.25	1.12	4.05	48.89	unclass. *Rhodobacteraceae*	*Rhodobacteraceae*	*Rhodobacterales*	*Alphaproteobacteria*	*Proteobacteria*	*Bacteria*
Otu0487	2.26	2.12	7.33	3.81	52.7	unclass. *Chitinophagaceae*	*Chitinophagaceae*	*Sphingobacteriales*	*Sphingobacteria*	*Bacteroidetes*	*Bacteria*
Otu0115	2.35	2.1	5.41	3.77	56.48	*Glaciecola*	*Alteromonadaceae*	*Alteromonadales*	*Gammaproteobacteria*	*Proteobacteria*	*Bacteria*
Otu0532	2.73	2.06	2.06	3.71	60.19	unclass. *Rhodobacteraceae*	*Rhodobacteraceae*	*Rhodobacterales*	*Alphaproteobacteria*	*Proteobacteria*	*Bacteria*
Otu0027	2.28	1.57	3.16	2.83	63.02	*Pseudoalteromonas*	*Pseudoalteromonadaceae*	*Alteromonadales*	*Gammaproteobacteria*	*Proteobacteria*	*Bacteria*
Otu0588	1.8	1.49	4.44	2.69	65.7	*Amphritea*	*Oceanospirillaceae*	*Oceanospirillales*	*Gammaproteobacteria*	*Proteobacteria*	*Bacteria*
Otu0525	1.62	1.42	3.8	2.55	68.26	*Reinekea*	*Oceanospirillaceae*	*Oceanospirillales*	*Gammaproteobacteria*	*Proteobacteria*	*Bacteria*
Otu0553	1.75	1.4	3.98	2.51	70.77	*Vibrio*	*Vibrionaceae*	*Vibrionales*	*Gammaproteobacteria*	*Proteobacteria*	*Bacteria*
Otu0110	2.08	1.33	1.15	2.4	73.17	unclass. *Rhodobacteraceae*	*Rhodobacteraceae*	*Rhodobacterales*	*Alphaproteobacteria*	*Proteobacteria*	*Bacteria*
Otu0576	1.81	1.31	2.53	2.35	75.52	unclass. *Alphaproteobacteria*	unclass. *Alphaproteobacteria*	unclass. *Alphaproteobacteria*	*Alphaproteobacteria*	*Proteobacteria*	*Bacteria*
Otu0583	1.17	1.1	6.61	1.97	77.49	unclass. *Flavobacteriaceae*	*Flavobacteriaceae*	*Flavobacteriales*	*Flavobacteria*	*Bacteroidetes*	*Bacteria*
Otu0489	1	1.05	10.72	1.89	79.38	unclass. *Vibrionaceae*	*Vibrionaceae*	*Vibrionales*	*Gammaproteobacteria*	*Proteobacteria*	*Bacteria*
Otu0551	1.57	0.96	1.05	1.73	81.11	*Arcobacter*	*Campylobacteraceae*	*Campylobacterales*	*Epsilonproteobacteria*	*Proteobacteria*	*Bacteria*
Otu0261	1.24	0.81	1.1	1.46	82.57	*Winogradskyella*	*Flavobacteriaceae*	*Flavobacteriales*	*Flavobacteria*	*Bacteroidetes*	*Bacteria*
Otu0542	1.09	0.67	1.11	1.21	83.77	unclass. *Oceanospirillaceae*	*Oceanospirillaceae*	*Oceanospirillales*	*Gammaproteobacteria*	*Proteobacteria*	*Bacteria*
Otu0578	0.97	0.65	1.11	1.16	84.94	*Winogradskyella*	*Flavobacteriaceae*	*Flavobacteriales*	*Flavobacteria*	*Bacteroidetes*	*Bacteria*
Otu0599	0.8	0.65	1.15	1.16	86.1	*Winogradskyella*	*Flavobacteriaceae*	*Flavobacteriales*	*Flavobacteria*	*Bacteroidetes*	*Bacteria*

OTU	$Av.A_i$	$Av.S_i$	$Av.S_i/SD$	$Av.S_i\%$	$\sum Av.S_i\%$	Genus	Family	Order	Class	Phylum	Domain
Otu0036	0.95	0.61	1.15	1.09	87.19	*Glaciecola*	*Alteromonadaceae*	*Alteromonadales*	*Gammaproteobacteria*	*Proteobacteria*	*Bacteria*
Otu0460	1.32	0.61	1.15	1.09	88.28	*Arcobacter*	*Campylobacteraceae*	*Campylobacterales*	*Epsilonproteobacteria*	*Proteobacteria*	*Bacteria*
Otu0533	0.95	0.6	1.16	1.08	89.37	*Nisaea*	*Rhodospirillaceae*	*Rhodospirillales*	*Alphaproteobacteria*	*Proteobacteria*	*Bacteria*
Otu0107	1.25	0.49	0.6	0.89	90.26	unclass. *Rhodobacteraceae*	*Rhodobacteraceae*	*Rhodobacterales*	*Alphaproteobacteria*	*Proteobacteria*	*Bacteria*
Summer 'initial dilution' pH 7.67 (average similarity: 55.0%)											
Otu0109	14.05	10.62	7.03	19.32	19.32	*Alteromonas*	*Alteromonadaceae*	*Alteromonadales*	*Gammaproteobacteria*	*Proteobacteria*	*Bacteria*
Otu0551	6.75	3.77	4.9	6.86	26.18	*Arcobacter*	*Campylobacteraceae*	*Campylobacterales*	*Epsilonproteobacteria*	*Proteobacteria*	*Bacteria*
Otu0588	4.34	3.05	3.45	5.56	31.73	*Amphritea*	*Oceanospirillaceae*	*Oceanospirillales*	*Gammaproteobacteria*	*Proteobacteria*	*Bacteria*
Otu0027	3.93	2.86	17.05	5.2	36.93	*Pseudoalteromonas*	*Pseudoalteromonadaceae*	*Alteromonadales*	*Gammaproteobacteria*	*Proteobacteria*	*Bacteria*
Otu0576	3.39	2.38	5.81	4.34	41.27	unclass. *Alphaproteobacteria*	unclass. *Alphaproteobacteria*	unclass. *Alphaproteobacteria*	*Alphaproteobacteria*	*Proteobacteria*	*Bacteria*
Otu0115	2.56	1.87	2.44	3.4	44.67	*Glaciecola*	*Alteromonadaceae*	*Alteromonadales*	*Gammaproteobacteria*	*Proteobacteria*	*Bacteria*
Otu0035	2.9	1.75	4.93	3.18	47.84	*Colwellia*	*Colwelliaceae*	*Alteromonadales*	*Gammaproteobacteria*	*Proteobacteria*	*Bacteria*
Otu0487	2.54	1.74	5.15	3.17	51.02	unclass. *Chitinophagaceae*	*Chitinophagaceae*	*Sphingobacteriales*	*Sphingobacteria*	*Bacteroidetes*	*Bacteria*
Otu0532	2.64	1.72	2.6	3.13	54.15	unclass. *Rhodobacteraceae*	*Rhodobacteraceae*	*Rhodobacterales*	*Alphaproteobacteria*	*Proteobacteria*	*Bacteria*
Otu0583	2.84	1.62	1.63	2.95	57.1	unclass. *Flavobacteriaceae*	*Flavobacteriaceae*	*Flavobacteriales*	*Flavobacteria*	*Bacteroidetes*	*Bacteria*
Otu0553	2.25	1.44	2.02	2.62	59.73	*Vibrio*	*Vibrionaceae*	*Vibrionales*	*Gammaproteobacteria*	*Proteobacteria*	*Bacteria*
Otu0525	2.36	1.44	2.18	2.61	62.34	*Reinekea*	*Oceanospirillaceae*	*Oceanospirillales*	*Gammaproteobacteria*	*Proteobacteria*	*Bacteria*
Otu0489	2.2	1.4	2.15	2.54	64.88	unclass. *Vibrionaceae*	*Vibrionaceae*	*Vibrionales*	*Gammaproteobacteria*	*Proteobacteria*	*Bacteria*
Otu0578	2.18	1.38	1.8	2.51	67.4	*Winogradskyella*	*Flavobacteriaceae*	*Flavobacteriales*	*Flavobacteria*	*Bacteroidetes*	*Bacteria*
Otu0041	2.89	1.32	0.91	2.41	69.8	*Sulfitobacter*	*Rhodobacteraceae*	*Rhodobacterales*	*Alphaproteobacteria*	*Proteobacteria*	*Bacteria*
Otu0261	1.77	1.22	18.19	2.21	72.02	*Winogradskyella*	*Flavobacteriaceae*	*Flavobacteriales*	*Flavobacteria*	*Bacteroidetes*	*Bacteria*
Otu0107	1.72	1.11	3.76	2.03	74.04	unclass. *Rhodobacteraceae*	*Rhodobacteraceae*	*Rhodobacterales*	*Alphaproteobacteria*	*Proteobacteria*	*Bacteria*
Otu0521	1.98	1.07	0.91	1.94	75.99	unclass. *Rhodobacteraceae*	*Rhodobacteraceae*	*Rhodobacterales*	*Alphaproteobacteria*	*Proteobacteria*	*Bacteria*
Otu0580	1.37	0.96	3.9	1.75	77.74	*Neptuniibacter*	*Oceanospirillaceae*	*Oceanospirillales*	*Gammaproteobacteria*	*Proteobacteria*	*Bacteria*
Otu0072	1.29	0.92	4.71	1.68	79.42	*Sulfitobacter*	*Rhodobacteraceae*	*Rhodobacterales*	*Alphaproteobacteria*	*Proteobacteria*	*Bacteria*
Otu0460	1.51	0.91	8.54	1.66	81.08	*Arcobacter*	*Campylobacteraceae*	*Campylobacterales*	*Epsilonproteobacteria*	*Proteobacteria*	*Bacteria*
Otu0615	1.29	0.91	8.54	1.66	82.75	*Neptuniibacter*	*Oceanospirillaceae*	*Oceanospirillales*	*Gammaproteobacteria*	*Proteobacteria*	*Bacteria*

OTU	$Av.A_i$	$Av.S_i$	$Av.S_i/SD$	$Av.S_i\%$	$\sum Av.S_i\%$	Genus	Family	Order	Class	Phylum	Domain
Otu0536	1.1	0.86	18.19	1.56	84.31	*Maribacter*	*Flavobacteriaceae*	*Flavobacteriales*	*Flavobacteria*	*Bacteroidetes*	*Bacteria*
Otu0533	1.62	0.71	0.89	1.29	85.6	*Nisaea*	*Rhodospirillaceae*	*Rhodospirillales*	*Alphaproteobacteria*	*Proteobacteria*	*Bacteria*
Otu0608	1.21	0.58	0.91	1.06	86.66	unclass. *Flavobacteriaceae*	*Flavobacteriaceae*	*Flavobacteriales*	*Flavobacteria*	*Bacteroidetes*	*Bacteria*
Otu0234	2.68	0.58	0.8	1.05	87.71	*Arcobacter*	*Campylobacteraceae*	*Campylobacterales*	*Epsilonproteobacteria*	*Proteobacteria*	*Bacteria*
Otu0346	1.56	0.58	0.8	1.05	88.76	unclass. *Bacteria*	unclass. *Bacteria*	unclass. *Bacteria*	unclass. *Bacteria*	unclass. *Bacteria*	*Bacteria*
Otu0459	1.22	0.48	0.88	0.88	89.64	*Croceibacter*	*Flavobacteriaceae*	*Flavobacteriales*	*Flavobacteria*	*Bacteroidetes*	*Bacteria*
Otu0110	0.85	0.44	0.91	0.8	90.45	unclass. *Rhodobacteraceae*	*Rhodobacteraceae*	*Rhodobacterales*	*Alphaproteobacteria*	*Proteobacteria*	*Bacteria*
Autumn 'no dilution' pH *in situ* (average similarity: 47.1%)											
Otu1090	5.7	2.07	11.11	4.4	4.4	unclass. *Bacteria*	unclass. *Bacteria*	unclass. *Bacteria*	unclass. *Bacteria*	unclass. *Bacteria*	*Bacteria*
Otu0068	3.94	1.51	8.64	3.21	7.61	*Pelagibacter*	SAR11-clade	*Rickettsiales*	*Alphaproteobacteria*	*Proteobacteria*	*Bacteria*
Otu0053	3.86	1.44	5.37	3.05	10.66	unclass. *Rhodobacteraceae*	*Rhodobacteraceae*	*Rhodobacterales*	*Alphaproteobacteria*	*Proteobacteria*	*Bacteria*
Otu1091	3.7	1.34	9.27	2.84	13.5	unclass. *Flavobacteriaceae*	*Flavobacteriaceae*	*Flavobacteriales*	*Flavobacteria*	*Bacteroidetes*	*Bacteria*
Otu0823	3.6	1.33	7.21	2.82	16.33	unclass. *Legionellaceae*	*Legionellaceae*	*Legionellales*	*Gammaproteobacteria*	*Proteobacteria*	*Bacteria*
Otu0001	4.28	1.22	2.44	2.58	18.91	unclass. *Flavobacteriaceae*	*Flavobacteriaceae*	*Flavobacteriales*	*Flavobacteria*	*Bacteroidetes*	*Bacteria*
Otu0005	4.32	1.18	2.18	2.49	21.4	*Pelagibacter*	SAR11-clade	*Rickettsiales*	*Alphaproteobacteria*	*Proteobacteria*	*Bacteria*
Otu0714	3.04	1.14	6.85	2.42	23.83	unclass. *Alphaproteobacteria*	unclass. *Alphaproteobacteria*	unclass. *Alphaproteobacteria*	*Alphaproteobacteria*	*Proteobacteria*	*Bacteria*
Otu1094	2.68	1.08	18.31	2.29	26.11	unclass. *Bacteroidetes*	unclass. *Bacteroidetes*	unclass. *Bacteroidetes*	unclass. *Bacteroidetes*	*Bacteroidetes*	*Bacteria*
Otu0272	3.28	1.04	1.84	2.21	28.32	unclass. *Flavobacteriaceae*	*Flavobacteriaceae*	*Flavobacteriales*	*Flavobacteria*	*Bacteroidetes*	*Bacteria*
Otu0408	2.67	1.03	28.01	2.18	30.5	unclass. *Flavobacteriaceae*	*Flavobacteriaceae*	*Flavobacteriales*	*Flavobacteria*	*Bacteroidetes*	*Bacteria*
Otu1093	3.01	0.99	8.09	2.11	32.61	unclass. *Proteobacteria*	unclass. *Proteobacteria*	unclass. *Proteobacteria*	unclass. *Proteobacteria*	*Proteobacteria*	*Bacteria*
Otu0826	2.8	0.99	3.79	2.11	34.71	unclass. *Bacteroidetes*	unclass. *Bacteroidetes*	unclass. *Bacteroidetes*	unclass. *Bacteroidetes*	*Bacteroidetes*	*Bacteria*
Otu0052	2.7	0.99	11.14	2.11	36.82	unclass. *Betaproteobacteria*	unclass. *Betaproteobacteria*	unclass. *Betaproteobacteria*	*Betaproteobacteria*	*Proteobacteria*	*Bacteria*
Otu0692	2.7	0.91	7.31	1.94	38.76	unclass. *Bacteria*	unclass. *Bacteria*	unclass. *Bacteria*	unclass. *Bacteria*	unclass. *Bacteria*	*Bacteria*
Otu1023	2.35	0.91	18.31	1.93	40.69	unclass. *Lentisphaeria*	unclass. *Lentisphaeria*	unclass. *Lentisphaeria*	*Lentisphaeria*	*Lentisphaerae*	*Bacteria*
Otu0681	2.85	0.89	3.15	1.88	42.57	unclass. *Bacteria*	unclass. *Bacteria*	unclass. *Bacteria*	unclass. *Bacteria*	unclass. *Bacteria*	*Bacteria*
Otu0469	2.44	0.86	4.64	1.82	44.39	unclass. *Bacteria*	unclass. *Bacteria*	unclass. *Bacteria*	unclass. *Bacteria*	unclass. *Bacteria*	*Bacteria*
Otu0764	2.18	0.84	14.08	1.79	46.18	unclass. *Proteobacteria*	unclass. *Proteobacteria*	unclass. *Proteobacteria*	unclass. *Proteobacteria*	*Proteobacteria*	*Bacteria*

OTU	$Av.A_i$	$Av.S_i$	$Av.S_i/SD$	$Av.S_i\%$	$\sum Av.S_i\%$	Genus	Family	Order	Class	Phylum	Domain
Otu0983	2.52	0.83	5.83	1.76	47.94	unclass. *Flavobacteriales*	unclass. *Flavobacteriales*	*Flavobacteriales*	*Flavobacteria*	*Bacteroidetes*	*Bacteria*
Otu0691	1.94	0.74	8.99	1.57	49.51	*Planctomyces*	*Planctomycetaceae*	*Planctomycetales*	*Planctomycetacia*	*Planctomycetes*	*Bacteria*
Otu0490	1.94	0.74	13.46	1.56	51.08	unclass. *Burkholderiales*	unclass. *Burkholderiales*	*Burkholderiales*	*Betaproteobacteria*	*Proteobacteria*	*Bacteria*
Otu0467	2.24	0.71	2.13	1.5	52.58	unclass. *Rhodospirillaceae*	*Rhodospirillaceae*	*Rhodospirillales*	*Alphaproteobacteria*	*Proteobacteria*	*Bacteria*
Otu0473	1.92	0.7	4.82	1.48	54.06	unclass. *Alphaproteobacteria*	unclass. *Alphaproteobacteria*	unclass. *Alphaproteobacteria*	*Alphaproteobacteria*	*Proteobacteria*	*Bacteria*
Otu1097	1.77	0.65	5.05	1.38	55.43	unclass. *Proteobacteria*	unclass. *Proteobacteria*	unclass. *Proteobacteria*	unclass. *Proteobacteria*	*Proteobacteria*	*Bacteria*
Otu0048	1.71	0.62	6.71	1.31	56.74	unclass. *Flavobacteriaceae*	*Flavobacteriaceae*	*Flavobacteriales*	*Flavobacteria*	*Bacteroidetes*	*Bacteria*
Otu0541	1.6	0.61	9.33	1.3	58.05	unclass. *Rhodobacteraceae*	*Rhodobacteraceae*	*Rhodobacterales*	*Alphaproteobacteria*	*Proteobacteria*	*Bacteria*
Otu0596	1.79	0.61	5.29	1.29	59.34	*Neptunomonas*	*Oceanospirillaceae*	*Oceanospirillales*	*Gammaproteobacteria*	*Proteobacteria*	*Bacteria*
Otu0494	2.08	0.6	2.93	1.27	60.61	unclass. *Flavobacteriaceae*	*Flavobacteriaceae*	*Flavobacteriales*	*Flavobacteria*	*Bacteroidetes*	*Bacteria*
Otu0776	1.76	0.55	4.22	1.17	61.79	unclass. *Proteobacteria*	unclass. *Proteobacteria*	unclass. *Proteobacteria*	unclass. *Proteobacteria*	*Proteobacteria*	*Bacteria*
Otu0705	2.29	0.55	1.1	1.16	62.95	*Planctomyces*	*Planctomycetaceae*	*Planctomycetales*	*Planctomycetacia*	*Planctomycetes*	*Bacteria*
Otu0281	1.52	0.54	4.7	1.15	64.1	unclass. *Flavobacteriales*	unclass. *Flavobacteriales*	*Flavobacteriales*	*Flavobacteria*	*Bacteroidetes*	*Bacteria*
Otu0509	1.39	0.51	6.06	1.08	65.18	unclass. *Gammaproteobacteria*	unclass. *Gammaproteobacteria*	unclass. *Gammaproteobacteria*	*Gammaproteobacteria*	*Proteobacteria*	*Bacteria*
Otu0771	1.48	0.49	3.04	1.03	66.21	unclass. *Rhodobacteraceae*	*Rhodobacteraceae*	*Rhodobacterales*	*Alphaproteobacteria*	*Proteobacteria*	*Bacteria*
Otu0972	1.48	0.48	3.9	1.02	67.22	*Nisaea*	*Rhodospirillaceae*	*Rhodospirillales*	*Alphaproteobacteria*	*Proteobacteria*	*Bacteria*
Otu1081	1.66	0.47	2.54	0.99	68.22	unclass. *Flavobacteriales*	unclass. *Flavobacteriales*	*Flavobacteriales*	*Flavobacteria*	*Bacteroidetes*	*Bacteria*
Otu0694	1.41	0.46	4.44	0.98	69.19	unclass. *Alphaproteobacteria*	unclass. *Alphaproteobacteria*	unclass. *Alphaproteobacteria*	*Alphaproteobacteria*	*Proteobacteria*	*Bacteria*
Otu1105	1.37	0.46	6.47	0.97	70.16	unclass. *Proteobacteria*	unclass. *Proteobacteria*	unclass. *Proteobacteria*	unclass. *Proteobacteria*	*Proteobacteria*	*Bacteria*
Otu0695	1.29	0.43	5.6	0.92	71.08	unclass. *Proteobacteria*	unclass. *Proteobacteria*	unclass. *Proteobacteria*	unclass. *Proteobacteria*	*Proteobacteria*	*Bacteria*
Otu0084	1.23	0.42	8.07	0.9	71.98	unclass. *Gammaproteobacteria*	unclass. *Gammaproteobacteria*	unclass. *Gammaproteobacteria*	*Gammaproteobacteria*	*Proteobacteria*	*Bacteria*
Otu0737	1.08	0.41	18.31	0.86	72.85	unclass. *Gammaproteobacteria*	unclass. *Gammaproteobacteria*	unclass. *Gammaproteobacteria*	*Gammaproteobacteria*	*Proteobacteria*	*Bacteria*
Otu0696	1.48	0.4	1.13	0.85	73.7	unclass. *Flavobacteriaceae*	*Flavobacteriaceae*	*Flavobacteriales*	*Flavobacteria*	*Bacteroidetes*	*Bacteria*
Otu0485	1.26	0.35	1.16	0.75	74.45	unclass. *Alphaproteobacteria*	unclass. *Alphaproteobacteria*	unclass. *Alphaproteobacteria*	*Alphaproteobacteria*	*Proteobacteria*	*Bacteria*
Otu0521	1.49	0.34	1.04	0.73	75.17	unclass. *Rhodobacteraceae*	*Rhodobacteraceae*	*Rhodobacterales*	*Alphaproteobacteria*	*Proteobacteria*	*Bacteria*
Otu1092	1.84	0.33	0.62	0.69	75.87	*Cycloclasticus*	*Piscirickettsiaceae*	*Thiotrichales*	*Gammaproteobacteria*	*Proteobacteria*	*Bacteria*
Otu1096	1.18	0.31	1.12	0.66	76.53	unclass. *Lentisphaeria*	unclass. *Lentisphaeria*	unclass. *Lentisphaeria*	*Lentisphaeria*	*Lentisphaerae*	*Bacteria*

OTU	$Av.A_i$	$Av.S_i$	$Av.S_i/SD$	$Av.S_i\%$	$\sum Av.S_i\%$	Genus	Family	Order	Class	Phylum	Domain
Otu0960	1.23	0.3	1.1	0.63	77.16	unclass. *Bacteria*	unclass. *Bacteria*	unclass. *Bacteria*	unclass. *Bacteria*	unclass. *Bacteria*	*Bacteria*
Otu0261	1.17	0.29	1.14	0.62	77.79	*Winogradskyella*	*Flavobacteriaceae*	*Flavobacteriales*	*Flavobacteria*	*Bacteroidetes*	*Bacteria*
Otu1113	1.11	0.29	1.11	0.61	78.39	unclass. *Gammaproteobacteria*	unclass. *Gammaproteobacteria*	unclass. *Gammaproteobacteria*	*Gammaproteobacteria*	*Proteobacteria*	*Bacteria*
Otu1115	1.17	0.29	1.11	0.61	79	*Marinobacter*	*Alteromonadaceae*	*Alteromonadales*	*Gammaproteobacteria*	*Proteobacteria*	*Bacteria*
Otu0778	1.17	0.28	1.12	0.6	79.6	unclass. *Gammaproteobacteria*	unclass. *Gammaproteobacteria*	unclass. *Gammaproteobacteria*	*Gammaproteobacteria*	*Proteobacteria*	*Bacteria*
Otu1125	1.11	0.28	1.12	0.6	80.21	*Planctomyces*	*Planctomycetaceae*	*Planctomycetales*	*Planctomycetacia*	*Planctomycetes*	*Bacteria*
Otu0621	1.15	0.28	1.11	0.59	80.8	*Colwellia*	*Colwelliaceae*	*Alteromonadales*	*Gammaproteobacteria*	*Proteobacteria*	*Bacteria*
Otu1100	1.09	0.27	1.09	0.58	81.38	unclass. *Planctomycetaceae*	*Planctomycetaceae*	*Planctomycetales*	*Planctomycetacia*	*Planctomycetes*	*Bacteria*
Otu0898	1.13	0.27	1.12	0.57	81.95	*Sphingopyxis*	*Sphingomonadaceae*	*Sphingomonadales*	*Alphaproteobacteria*	*Proteobacteria*	*Bacteria*
Otu0255	0.97	0.25	1.13	0.54	82.49	*Sphingopyxis*	*Sphingomonadaceae*	*Sphingomonadales*	*Alphaproteobacteria*	*Proteobacteria*	*Bacteria*
Otu1111	0.88	0.25	1.16	0.53	83.02	unclass. *Proteobacteria*	unclass. *Proteobacteria*	unclass. *Proteobacteria*	unclass. *Proteobacteria*	*Proteobacteria*	*Bacteria*
Otu0601	0.8	0.25	1.16	0.52	83.54	unclass. *Flammeovirgaceae*	*Flammeovirgaceae*	*Sphingobacteriales*	*Sphingobacteria*	*Bacteroidetes*	*Bacteria*
Otu0817	0.8	0.25	1.16	0.52	84.06	unclass. *Bacteroidetes*	unclass. *Bacteroidetes*	unclass. *Bacteroidetes*	unclass. *Bacteroidetes*	*Bacteroidetes*	*Bacteria*
Otu0170	0.8	0.24	1.16	0.51	84.57	unclass. *Bacteria*	unclass. *Bacteria*	unclass. *Bacteria*	unclass. *Bacteria*	unclass. *Bacteria*	*Bacteria*
Otu0137	0.88	0.24	1.16	0.5	85.08	unclass. *Bacteroidetes*	unclass. *Bacteroidetes*	unclass. *Bacteroidetes*	unclass. *Bacteroidetes*	*Bacteroidetes*	*Bacteria*
Otu0202	0.88	0.24	1.16	0.5	85.58	unclass. *Alteromonadaceae*	*Alteromonadaceae*	*Alteromonadales*	*Gammaproteobacteria*	*Proteobacteria*	*Bacteria*
Otu0959	0.88	0.24	1.16	0.5	86.08	unclass. *Rhodobacteraceae*	*Rhodobacteraceae*	*Rhodobacterales*	*Alphaproteobacteria*	*Proteobacteria*	*Bacteria*
Otu0710	1.24	0.23	0.62	0.49	86.57	unclass. *Gammaproteobacteria*	unclass. *Gammaproteobacteria*	unclass. *Gammaproteobacteria*	*Gammaproteobacteria*	*Proteobacteria*	*Bacteria*
Otu0186	0.85	0.18	0.62	0.38	86.95	unclass. *Planctomycetaceae*	*Planctomycetaceae*	*Planctomycetales*	*Planctomycetacia*	*Planctomycetes*	*Bacteria*
Otu0645	0.97	0.16	0.62	0.34	87.29	*Arcobacter*	*Campylobacteraceae*	*Campylobacterales*	*Epsilonproteobacteria*	*Proteobacteria*	*Bacteria*
Otu0157	1.04	0.16	0.59	0.34	87.64	unclass. *Gammaproteobacteria*	unclass. *Gammaproteobacteria*	unclass. *Gammaproteobacteria*	*Gammaproteobacteria*	*Proteobacteria*	*Bacteria*
Otu0682	0.89	0.15	0.6	0.33	87.96	unclass. *Oceanospirillaceae*	*Oceanospirillaceae*	*Oceanospirillales*	*Gammaproteobacteria*	*Proteobacteria*	*Bacteria*
Otu0846	0.83	0.14	0.61	0.31	88.27	unclass. *Nannocystaceae*	*Nannocystaceae*	*Myxococcales*	*Deltaproteobacteria*	*Proteobacteria*	*Bacteria*
Otu0611	0.83	0.14	0.61	0.3	88.57	unclass. *Bacteroidetes*	unclass. *Bacteroidetes*	unclass. *Bacteroidetes*	unclass. *Bacteroidetes*	*Bacteroidetes*	*Bacteria*
Otu0243	0.77	0.14	0.6	0.3	88.87	unclass. *Gammaproteobacteria*	unclass. *Gammaproteobacteria*	unclass. *Gammaproteobacteria*	*Gammaproteobacteria*	*Proteobacteria*	*Bacteria*
Otu1118	0.77	0.14	0.61	0.29	89.17	unclass. *Oceanospirillales*	unclass. *Oceanospirillales*	*Oceanospirillales*	*Gammaproteobacteria*	*Proteobacteria*	*Bacteria*
Otu0742	0.77	0.14	0.61	0.29	89.46	unclass. *Rhodobacteraceae*	*Rhodobacteraceae*	*Rhodobacterales*	*Alphaproteobacteria*	*Proteobacteria*	*Bacteria*

OTU	$Av.A_i$	$Av.S_i$	$Av.S_i/SD$	$Av.S_i\%$	$\sum Av.S_i\%$	Genus	Family	Order	Class	Phylum	Domain
Otu0502	0.83	0.14	0.61	0.29	89.74	unclass. *Gammaproteobacteria*	unclass. *Gammaproteobacteria*	unclass. *Gammaproteobacteria*	*Gammaproteobacteria*	*Proteobacteria*	*Bacteria*
Otu1099	0.77	0.13	0.61	0.28	90.02	unclass. *Lentisphaeria*	unclass. *Lentisphaeria*	unclass. *Lentisphaeria*	*Lentisphaeria*	*Lentisphaerae*	*Bacteria*
Autumn 'no dilution' pH 7.67 (average similarity: 41.4%)											
Otu0823	4.66	1.37	3.1	3.3	3.3	unclass. *Legionellaceae*	*Legionellaceae*	*Legionellales*	*Gammaproteobacteria*	*Proteobacteria*	*Bacteria*
Otu0068	4.27	1.27	7.8	3.06	6.36	*Pelagibacter*	SAR11-clade	*Rickettsiales*	*Alphaproteobacteria*	*Proteobacteria*	*Bacteria*
Otu0691	4.06	1.16	2.77	2.8	9.16	*Planctomyces*	*Planctomycetaceae*	*Planctomycetales*	*Planctomycetacia*	*Planctomycetes*	*Bacteria*
Otu1091	2.89	0.97	24.66	2.35	11.51	unclass. *Flavobacteriaceae*	*Flavobacteriaceae*	*Flavobacteriales*	*Flavobacteria*	*Bacteroidetes*	*Bacteria*
Otu1094	3.09	0.96	7.44	2.31	13.82	unclass. *Bacteroidetes*	unclass. *Bacteroidetes*	unclass. *Bacteroidetes*	unclass. *Bacteroidetes*	*Bacteroidetes*	*Bacteria*
Otu1023	2.9	0.93	41	2.26	16.08	unclass. *Lentisphaeria*	unclass. *Lentisphaeria*	unclass. *Lentisphaeria*	*Lentisphaeria*	*Lentisphaerae*	*Bacteria*
Otu0005	3.46	0.91	2.18	2.19	18.27	*Pelagibacter*	SAR11-clade	*Rickettsiales*	*Alphaproteobacteria*	*Proteobacteria*	*Bacteria*
Otu0705	2.94	0.85	3.37	2.05	20.32	*Planctomyces*	*Planctomycetaceae*	*Planctomycetales*	*Planctomycetacia*	*Planctomycetes*	*Bacteria*
Otu0053	2.81	0.83	2.84	2.01	22.33	unclass. *Rhodobacteraceae*	*Rhodobacteraceae*	*Rhodobacterales*	*Alphaproteobacteria*	*Proteobacteria*	*Bacteria*
Otu0735	2.76	0.83	3.78	2	24.33	unclass. *Gammaproteobacteria*	unclass. *Gammaproteobacteria*	unclass. *Gammaproteobacteria*	*Gammaproteobacteria*	*Proteobacteria*	*Bacteria*
Otu1093	2.75	0.8	5.96	1.94	26.27	unclass. *Proteobacteria*	unclass. *Proteobacteria*	unclass. *Proteobacteria*	unclass. *Proteobacteria*	*Proteobacteria*	*Bacteria*
Otu0765	2.45	0.78	75.16	1.89	28.16	*Pelagicoccus*	*Puniceicoccaceae*	*Puniceicoccales*	*Opitutae*	*Verrucomicrobia*	*Bacteria*
Otu0490	2.22	0.72	7.42	1.74	29.9	unclass. *Burkholderiales*	unclass. *Burkholderiales*	*Burkholderiales*	*Betaproteobacteria*	*Proteobacteria*	*Bacteria*
Otu0469	2.49	0.72	3.63	1.73	31.63	unclass. *Bacteria*	unclass. *Bacteria*	unclass. *Bacteria*	unclass. *Bacteria*	unclass. *Bacteria*	*Bacteria*
Otu0261	2.47	0.7	4.52	1.68	33.32	*Winogradskyella*	*Flavobacteriaceae*	*Flavobacteriales*	*Flavobacteria*	*Bacteroidetes*	*Bacteria*
Otu0826	2.19	0.67	4.48	1.62	34.93	unclass. *Bacteroidetes*	unclass. *Bacteroidetes*	unclass. *Bacteroidetes*	unclass. *Bacteroidetes*	*Bacteroidetes*	*Bacteria*
Otu0714	2.12	0.67	6.54	1.61	36.55	unclass. *Alphaproteobacteria*	unclass. *Alphaproteobacteria*	unclass. *Alphaproteobacteria*	*Alphaproteobacteria*	*Proteobacteria*	*Bacteria*
Otu0692	2.39	0.67	3.81	1.61	38.16	unclass. *Bacteria*	unclass. *Bacteria*	unclass. *Bacteria*	unclass. *Bacteria*	unclass. *Bacteria*	*Bacteria*
Otu0052	2.32	0.66	4.18	1.59	39.75	unclass. *Betaproteobacteria*	unclass. *Betaproteobacteria*	unclass. *Betaproteobacteria*	*Betaproteobacteria*	*Proteobacteria*	*Bacteria*
Otu0830	2.18	0.64	4.77	1.56	41.3	unclass. *Gammaproteobacteria*	unclass. *Gammaproteobacteria*	unclass. *Gammaproteobacteria*	*Gammaproteobacteria*	*Proteobacteria*	*Bacteria*
Otu1090	2.33	0.64	3.67	1.54	42.84	unclass. *Bacteria*	unclass. *Bacteria*	unclass. *Bacteria*	unclass. *Bacteria*	unclass. *Bacteria*	*Bacteria*
Otu0281	2.15	0.61	2.57	1.47	44.32	unclass. *Flavobacteriales*	unclass. *Flavobacteriales*	*Flavobacteriales*	*Flavobacteria*	*Bacteroidetes*	*Bacteria*
Otu0710	1.89	0.56	5.96	1.34	45.66	unclass. *Gammaproteobacteria*	unclass. *Gammaproteobacteria*	unclass. *Gammaproteobacteria*	*Gammaproteobacteria*	*Proteobacteria*	*Bacteria*
Otu0681	1.59	0.51	13.68	1.22	46.89	unclass. *Bacteria*	unclass. *Bacteria*	unclass. *Bacteria*	unclass. *Bacteria*	unclass. *Bacteria*	*Bacteria*

OTU	Av.A_i	Av.S_i	Av.S_i/SD	Av.S_i%	∑Av.S_i%	Genus	Family	Order	Class	Phylum	Domain
Otu0359	1.68	0.5	3.34	1.2	48.09	unclass. *Gammaproteobacteria*	unclass. *Gammaproteobacteria*	unclass. *Gammaproteobacteria*	*Gammaproteobacteria*	*Proteobacteria*	*Bacteria*
Otu1062	1.68	0.48	3.65	1.16	49.25	unclass. *Gammaproteobacteria*	unclass. *Gammaproteobacteria*	unclass. *Gammaproteobacteria*	*Gammaproteobacteria*	*Proteobacteria*	*Bacteria*
Otu1096	1.52	0.47	4.04	1.14	50.39	unclass. *Lentisphaeria*	unclass. *Lentisphaeria*	unclass. *Lentisphaeria*	*Lentisphaeria*	*Lentisphaerae*	*Bacteria*
Otu0645	1.52	0.47	4.15	1.14	51.53	*Arcobacter*	*Campylobacteraceae*	*Campylobacterales*	*Epsilonproteobacteria*	*Proteobacteria*	*Bacteria*
Otu0737	1.58	0.47	4.15	1.14	52.67	unclass. *Gammaproteobacteria*	unclass. *Gammaproteobacteria*	unclass. *Gammaproteobacteria*	*Gammaproteobacteria*	*Proteobacteria*	*Bacteria*
Otu0495	1.71	0.45	4.92	1.08	53.75	unclass. *Flavobacteriales*	unclass. *Flavobacteriales*	*Flavobacteriales*	*Flavobacteria*	*Bacteroidetes*	*Bacteria*
Otu0521	1.43	0.41	4.29	0.98	54.73	unclass. *Rhodobacteraceae*	*Rhodobacteraceae*	*Rhodobacterales*	*Alphaproteobacteria*	*Proteobacteria*	*Bacteria*
Otu0084	1.52	0.4	4.5	0.98	55.71	unclass. *Gammaproteobacteria*	unclass. *Gammaproteobacteria*	unclass. *Gammaproteobacteria*	*Gammaproteobacteria*	*Proteobacteria*	*Bacteria*
Otu0272	1.31	0.39	5.33	0.95	56.66	unclass. *Flavobacteriaceae*	*Flavobacteriaceae*	*Flavobacteriales*	*Flavobacteria*	*Bacteroidetes*	*Bacteria*
Otu0758	1.37	0.39	5.8	0.95	57.61	unclass. *Bacteria*	unclass. *Bacteria*	unclass. *Bacteria*	unclass. *Bacteria*	unclass. *Bacteria*	*Bacteria*
Otu0682	1.35	0.38	4.69	0.91	58.52	unclass. *Oceanospirillaceae*	*Oceanospirillaceae*	*Oceanospirillales*	*Gammaproteobacteria*	*Proteobacteria*	*Bacteria*
Otu1107	1.35	0.38	4.86	0.91	59.43	unclass. *Planctomycetaceae*	*Planctomycetaceae*	*Planctomycetales*	*Planctomycetacia*	*Planctomycetes*	*Bacteria*
Otu0137	1.23	0.37	8.19	0.88	60.31	unclass. *Bacteroidetes*	unclass. *Bacteroidetes*	unclass. *Bacteroidetes*	unclass. *Bacteroidetes*	*Bacteroidetes*	*Bacteria*
Otu1081	1.68	0.35	1.1	0.85	61.17	unclass. *Flavobacteriales*	unclass. *Flavobacteriales*	*Flavobacteriales*	*Flavobacteria*	*Bacteroidetes*	*Bacteria*
Otu1092	1.89	0.31	0.62	0.75	61.91	*Cycloclasticus*	*Piscirickettsiaceae*	*Thiotrichales*	*Gammaproteobacteria*	*Proteobacteria*	*Bacteria*
Otu1113	1.13	0.3	1.16	0.72	62.63	unclass. *Gammaproteobacteria*	unclass. *Gammaproteobacteria*	unclass. *Gammaproteobacteria*	*Gammaproteobacteria*	*Proteobacteria*	*Bacteria*
Otu0694	1.19	0.3	1.16	0.72	63.35	unclass. *Alphaproteobacteria*	unclass. *Alphaproteobacteria*	unclass. *Alphaproteobacteria*	*Alphaproteobacteria*	*Proteobacteria*	*Bacteria*
Otu1125	1.53	0.28	1.04	0.68	64.03	*Planctomyces*	*Planctomycetaceae*	*Planctomycetales*	*Planctomycetacia*	*Planctomycetes*	*Bacteria*
Otu0255	1.23	0.26	1.1	0.64	64.67	*Sphingopyxis*	*Sphingomonadaceae*	*Sphingomonadales*	*Alphaproteobacteria*	*Proteobacteria*	*Bacteria*
Otu0895	1.18	0.26	1.1	0.64	65.31	unclass. *Actinobacteria*	unclass. *Actinobacteria*	unclass. *Actinobacteria*	*Actinobacteria*	*Actinobacteria*	*Bacteria*
Otu0001	1.18	0.26	1.1	0.64	65.95	unclass. *Flavobacteriaceae*	*Flavobacteriaceae*	*Flavobacteriales*	*Flavobacteria*	*Bacteroidetes*	*Bacteria*
Otu0473	1.11	0.26	1.12	0.62	66.56	unclass. *Alphaproteobacteria*	unclass. *Alphaproteobacteria*	unclass. *Alphaproteobacteria*	*Alphaproteobacteria*	*Proteobacteria*	*Bacteria*
Otu1193	1.05	0.26	1.12	0.62	67.18	unclass. *Saprospiraceae*	*Saprospiraceae*	*Sphingobacteriales*	*Sphingobacteria*	*Bacteroidetes*	*Bacteria*
Otu1099	1.29	0.25	1.12	0.61	67.8	unclass. *Lentisphaeria*	unclass. *Lentisphaeria*	unclass. *Lentisphaeria*	*Lentisphaeria*	*Lentisphaerae*	*Bacteria*
Otu0156	1.29	0.25	1.12	0.61	68.41	unclass. *Gammaproteobacteria*	unclass. *Gammaproteobacteria*	unclass. *Gammaproteobacteria*	*Gammaproteobacteria*	*Proteobacteria*	*Bacteria*
Otu0186	1.17	0.25	1.12	0.61	69.03	unclass. *Planctomycetaceae*	*Planctomycetaceae*	*Planctomycetales*	*Planctomycetacia*	*Planctomycetes*	*Bacteria*
Otu0696	1.11	0.25	1.12	0.61	69.64	unclass. *Flavobacteriaceae*	*Flavobacteriaceae*	*Flavobacteriales*	*Flavobacteria*	*Bacteroidetes*	*Bacteria*

OTU	$Av.A_i$	$Av.S_i$	$Av.S_i/SD$	$Av.S_i\%$	$\sum Av.S_i\%$	Genus	Family	Order	Class	Phylum	Domain
Otu0194	1.29	0.25	1	0.61	70.25	unclass. *Bacteroidetes*	unclass. *Bacteroidetes*	unclass. *Bacteroidetes*	unclass. *Bacteroidetes*	*Bacteroidetes*	*Bacteria*
Otu0517	1.05	0.25	1.12	0.61	70.86	unclass. *Bacteroidetes*	unclass. *Bacteroidetes*	unclass. *Bacteroidetes*	unclass. *Bacteroidetes*	*Bacteroidetes*	*Bacteria*
Otu1147	1.11	0.25	1.12	0.61	71.47	unclass. *Bacteria*	unclass. *Bacteria*	unclass. *Bacteria*	unclass. *Bacteria*	unclass. *Bacteria*	*Bacteria*
Otu0849	1.21	0.25	1.12	0.61	72.08	unclass. *Alphaproteobacteria*	unclass. *Alphaproteobacteria*	unclass. *Alphaproteobacteria*	*Alphaproteobacteria*	*Proteobacteria*	*Bacteria*
Otu0596	1.17	0.25	1.12	0.61	72.69	*Neptunomonas*	*Oceanospirillaceae*	*Oceanospirillales*	*Gammaproteobacteria*	*Proteobacteria*	*Bacteria*
Otu0503	1.15	0.24	1.08	0.57	73.26	unclass. *Bacteroidetes*	unclass. *Bacteroidetes*	unclass. *Bacteroidetes*	unclass. *Bacteroidetes*	*Bacteroidetes*	*Bacteria*
Otu0450	1.09	0.23	1.09	0.57	73.83	*Sphingobium*	*Sphingomonadaceae*	*Sphingomonadales*	*Alphaproteobacteria*	*Proteobacteria*	*Bacteria*
Otu0003	1.21	0.23	1.13	0.55	74.37	unclass. *Rhodobacteraceae*	*Rhodobacteraceae*	*Rhodobacterales*	*Alphaproteobacteria*	*Proteobacteria*	*Bacteria*
Otu0157	1.46	0.23	1.14	0.55	74.92	unclass. *Gammaproteobacteria*	unclass. *Gammaproteobacteria*	unclass. *Gammaproteobacteria*	*Gammaproteobacteria*	*Proteobacteria*	*Bacteria*
Otu0509	1.08	0.23	1.14	0.55	75.47	unclass. *Gammaproteobacteria*	unclass. *Gammaproteobacteria*	unclass. *Gammaproteobacteria*	*Gammaproteobacteria*	*Proteobacteria*	*Bacteria*
Otu0971	0.97	0.23	1.13	0.54	76.01	unclass. *Gammaproteobacteria*	unclass. *Gammaproteobacteria*	unclass. *Gammaproteobacteria*	*Gammaproteobacteria*	*Proteobacteria*	*Bacteria*
Otu0134	1.17	0.23	1.13	0.54	76.56	unclass. *Gammaproteobacteria*	unclass. *Gammaproteobacteria*	unclass. *Gammaproteobacteria*	*Gammaproteobacteria*	*Proteobacteria*	*Bacteria*
Otu0541	1.03	0.23	1.13	0.54	77.1	unclass. *Rhodobacteraceae*	*Rhodobacteraceae*	*Rhodobacterales*	*Alphaproteobacteria*	*Proteobacteria*	*Bacteria*
Otu1333	1.03	0.23	1.13	0.54	77.64	unclass. *Legionellaceae*	*Legionellaceae*	*Legionellales*	*Gammaproteobacteria*	*Proteobacteria*	*Bacteria*
Otu1209	1.03	0.22	1.13	0.54	78.19	unclass. *Bacteria*	unclass. *Bacteria*	unclass. *Bacteria*	unclass. *Bacteria*	unclass. *Bacteria*	*Bacteria*
Otu0844	1.08	0.22	1.14	0.54	78.73	unclass. *Flavobacteriaceae*	*Flavobacteriaceae*	*Flavobacteriales*	*Flavobacteria*	*Bacteroidetes*	*Bacteria*
Otu0761	0.88	0.21	1.16	0.51	79.24	unclass. *Verrucomicrobiaceae*	*Verrucomicrobiaceae*	*Verrucomicrobiales*	*Verrucomicrobiae*	*Verrucomicrobia*	*Bacteria*
Otu1430	0.88	0.21	1.16	0.51	79.74	unclass. *Bacteria*	unclass. *Bacteria*	unclass. *Bacteria*	unclass. *Bacteria*	unclass. *Bacteria*	*Bacteria*
Otu0707	0.8	0.21	1.16	0.51	80.25	*Haliea*	*Alteromonadaceae*	*Alteromonadales*	*Gammaproteobacteria*	*Proteobacteria*	*Bacteria*
Otu1270	0.88	0.21	1.16	0.51	80.76	unclass. *Flavobacteriaceae*	*Flavobacteriaceae*	*Flavobacteriales*	*Flavobacteria*	*Bacteroidetes*	*Bacteria*
Otu0394	0.8	0.21	1.16	0.51	81.27	unclass. *Bacteria*	unclass. *Bacteria*	unclass. *Bacteria*	unclass. *Bacteria*	unclass. *Bacteria*	*Bacteria*
Otu0502	0.95	0.21	1.16	0.51	81.77	unclass. *Gammaproteobacteria*	unclass. *Gammaproteobacteria*	unclass. *Gammaproteobacteria*	*Gammaproteobacteria*	*Proteobacteria*	*Bacteria*
Otu0973	0.8	0.21	1.16	0.51	82.28	unclass. *Flavobacteriaceae*	*Flavobacteriaceae*	*Flavobacteriales*	*Flavobacteria*	*Bacteroidetes*	*Bacteria*
Otu1198	0.88	0.21	1.16	0.51	82.78	unclass. *Gammaproteobacteria*	unclass. *Gammaproteobacteria*	unclass. *Gammaproteobacteria*	*Gammaproteobacteria*	*Proteobacteria*	*Bacteria*
Otu1386	1.21	0.17	0.61	0.41	83.19	unclass. *Bacteria*	unclass. *Bacteria*	unclass. *Bacteria*	unclass. *Bacteria*	unclass. *Bacteria*	*Bacteria*
Otu1350	1.18	0.15	0.56	0.36	83.56	unclass. *Bacteria*	unclass. *Bacteria*	unclass. *Bacteria*	unclass. *Bacteria*	unclass. *Bacteria*	*Bacteria*
Otu0048	0.91	0.15	0.62	0.36	83.91	unclass. *Flavobacteriaceae*	*Flavobacteriaceae*	*Flavobacteriales*	*Flavobacteria*	*Bacteroidetes*	*Bacteria*

OTU	Av.A$_i$	Av.S$_i$	Av.S$_i$/SD	Av.S$_i$%	∑Av.S$_i$%	Genus	Family	Order	Class	Phylum	Domain
Otu0139	1.04	0.13	0.59	0.32	84.23	unclass. *Gammaproteobacteria*	unclass. *Gammaproteobacteria*	unclass. *Gammaproteobacteria*	*Gammaproteobacteria*	*Proteobacteria*	*Bacteria*
Otu1102	1.21	0.13	0.59	0.32	84.55	unclass. *Proteobacteria*	unclass. *Proteobacteria*	unclass. *Proteobacteria*	unclass. *Proteobacteria*	*Proteobacteria*	*Bacteria*
Otu1173	0.89	0.13	0.59	0.32	84.87	*Opitutus*	*Opitutaceae*	*Opitutales*	*Opitutae*	*Verrucomicrobia*	*Bacteria*
Otu0898	1.04	0.13	0.59	0.31	85.19	*Sphingopyxis*	*Sphingomonadaceae*	*Sphingomonadales*	*Alphaproteobacteria*	*Proteobacteria*	*Bacteria*
Otu0986	0.77	0.12	0.61	0.29	85.48	unclass. *Planctomycetaceae*	*Planctomycetaceae*	*Planctomycetales*	*Planctomycetacia*	*Planctomycetes*	*Bacteria*
Otu1234	0.83	0.12	0.61	0.29	85.77	unclass. *Oceanospirillaceae*	*Oceanospirillaceae*	*Oceanospirillales*	*Gammaproteobacteria*	*Proteobacteria*	*Bacteria*
Otu0075	0.77	0.12	0.61	0.29	86.05	unclass. *Alphaproteobacteria*	unclass. *Alphaproteobacteria*	unclass. *Alphaproteobacteria*	*Alphaproteobacteria*	*Proteobacteria*	*Bacteria*
Otu0464	0.83	0.12	0.61	0.29	86.34	unclass. *Bacteroidetes*	unclass. *Bacteroidetes*	unclass. *Bacteroidetes*	unclass. *Bacteroidetes*	*Bacteroidetes*	*Bacteria*
Otu0485	0.83	0.12	0.61	0.29	86.63	unclass. *Alphaproteobacteria*	unclass. *Alphaproteobacteria*	unclass. *Alphaproteobacteria*	*Alphaproteobacteria*	*Proteobacteria*	*Bacteria*
Otu0467	1.01	0.12	0.61	0.29	86.91	unclass. *Rhodospirillaceae*	*Rhodospirillaceae*	*Rhodospirillales*	*Alphaproteobacteria*	*Proteobacteria*	*Bacteria*
Otu0142	0.6	0.11	0.62	0.26	87.17	unclass. *Gammaproteobacteria*	unclass. *Gammaproteobacteria*	unclass. *Gammaproteobacteria*	*Gammaproteobacteria*	*Proteobacteria*	*Bacteria*
Otu0734	0.68	0.11	0.62	0.26	87.43	unclass. *Bacteria*	unclass. *Bacteria*	unclass. *Bacteria*	unclass. *Bacteria*	unclass. *Bacteria*	*Bacteria*
Otu1219	0.6	0.11	0.62	0.26	87.68	unclass. *Bacteria*	unclass. *Bacteria*	unclass. *Bacteria*	unclass. *Bacteria*	unclass. *Bacteria*	*Bacteria*
Otu0395	0.68	0.11	0.62	0.26	87.94	unclass. *Sphingobacteriales*	unclass. *Sphingobacteriales*	*Sphingobacteriales*	*Sphingobacteria*	*Bacteroidetes*	*Bacteria*
Otu1109	0.6	0.11	0.62	0.26	88.19	unclass. *Saprospiraceae*	*Saprospiraceae*	*Sphingobacteriales*	*Sphingobacteria*	*Bacteroidetes*	*Bacteria*
Otu1135	0.68	0.11	0.62	0.26	88.45	unclass. *Sphingobacteriales*	unclass. *Sphingobacteriales*	*Sphingobacteriales*	*Sphingobacteria*	*Bacteroidetes*	*Bacteria*
Otu0601	0.6	0.11	0.62	0.25	88.71	unclass. *Flammeovirgaceae*	*Flammeovirgaceae*	*Sphingobacteriales*	*Sphingobacteria*	*Bacteroidetes*	*Bacteria*
Otu0846	0.68	0.11	0.62	0.25	88.96	unclass. *Nannocystaceae*	*Nannocystaceae*	*Myxococcales*	*Deltaproteobacteria*	*Proteobacteria*	*Bacteria*
Otu0998	0.75	0.11	0.62	0.25	89.22	unclass. *Gammaproteobacteria*	unclass. *Gammaproteobacteria*	unclass. *Gammaproteobacteria*	*Gammaproteobacteria*	*Proteobacteria*	*Bacteria*
Otu0408	0.68	0.11	0.62	0.25	89.47	unclass. *Flavobacteriaceae*	*Flavobacteriaceae*	*Flavobacteriales*	*Flavobacteria*	*Bacteroidetes*	*Bacteria*
Otu0767	0.6	0.11	0.62	0.25	89.72	unclass. *Bacteria*	unclass. *Bacteria*	unclass. *Bacteria*	unclass. *Bacteria*	unclass. *Bacteria*	*Bacteria*
Otu1195	0.68	0.1	0.62	0.25	89.98	unclass. *Bacteria*	unclass. *Bacteria*	unclass. *Bacteria*	unclass. *Bacteria*	unclass. *Bacteria*	*Bacteria*
Otu1256	0.68	0.1	0.62	0.25	90.23	unclass. *Sphingobacteriales*	unclass. *Sphingobacteriales*	*Sphingobacteriales*	*Sphingobacteria*	*Bacteroidetes*	*Bacteria*
Autumn 'serial dilution' pH *in situ* (average similarity: 66.8%)											
Otu0132	16.35	26.63	2.66	39.89	39.89	unclass. *Alteromonadales*	unclass. *Alteromonadales*	*Alteromonadales*	*Gammaproteobacteria*	*Proteobacteria*	*Bacteria*
Otu0106	9.15	11.09	1.94	16.61	56.5	*Marinomonas*	*Oceanospirillaceae*	*Oceanospirillales*	*Gammaproteobacteria*	*Proteobacteria*	*Bacteria*
Otu0107	7.12	10.47	4.17	15.68	72.18	unclass. *Rhodobacteraceae*	*Rhodobacteraceae*	*Rhodobacterales*	*Alphaproteobacteria*	*Proteobacteria*	*Bacteria*

OTU	Av.A_i	Av.S_i	Av.S_i/SD	Av.S_i%	∑Av.S_i%	Genus	Family	Order	Class	Phylum	Domain
Otu0471	2.82	4.56	3.38	6.83	79.01	*Oceaniserpentilla*	*Oceanospirillaceae*	*Oceanospirillales*	*Gammaproteobacteria*	*Proteobacteria*	*Bacteria*
Otu0001	3.02	3.66	3.38	5.49	84.5	unclass. *Flavobacteriaceae*	*Flavobacteriaceae*	*Flavobacteriales*	*Flavobacteria*	*Bacteroidetes*	*Bacteria*
Otu0123	2.23	2.67	1.03	4	88.5	unclass. *Gammaproteobacteria*	unclass. *Gammaproteobacteria*	unclass. *Gammaproteobacteria*	*Gammaproteobacteria*	*Proteobacteria*	*Bacteria*
Otu0036	2.12	2.64	3.79	3.96	92.46	*Glaciecola*	*Alteromonadaceae*	*Alteromonadales*	*Gammaproteobacteria*	*Proteobacteria*	*Bacteria*
Autumn 'serial dilution' pH 7.67 (average similarity: 61.5%)											
Otu0132	13.24	18.33	3.29	29.8	29.8	unclass. *Alteromonadales*	unclass. *Alteromonadales*	*Alteromonadales*	*Gammaproteobacteria*	*Proteobacteria*	*Bacteria*
Otu0106	11.14	11.89	1.81	19.33	49.13	*Marinomonas*	*Oceanospirillaceae*	*Oceanospirillales*	*Gammaproteobacteria*	*Proteobacteria*	*Bacteria*
Otu0471	6.73	7.09	2.06	11.52	60.65	*Oceaniserpentilla*	*Oceanospirillaceae*	*Oceanospirillales*	*Gammaproteobacteria*	*Proteobacteria*	*Bacteria*
Otu0107	5.35	6.96	4.15	11.32	71.97	unclass. *Rhodobacteraceae*	*Rhodobacteraceae*	*Rhodobacterales*	*Alphaproteobacteria*	*Proteobacteria*	*Bacteria*
Otu0551	4.01	4.71	2.9	7.65	79.63	*Arcobacter*	*Campylobacteraceae*	*Campylobacterales*	*Epsilonproteobacteria*	*Proteobacteria*	*Bacteria*
Otu0036	3.66	4.03	2.37	6.56	86.19	*Glaciecola*	*Alteromonadaceae*	*Alteromonadales*	*Gammaproteobacteria*	*Proteobacteria*	*Bacteria*
Otu0621	1.78	1.59	0.98	2.58	88.77	*Colwellia*	*Colwelliaceae*	*Alteromonadales*	*Gammaproteobacteria*	*Proteobacteria*	*Bacteria*
Otu0123	1.74	1.47	0.99	2.39	91.16	unclass. *Gammaproteobacteria*	unclass. *Gammaproteobacteria*	unclass. *Gammaproteobacteria*	*Gammaproteobacteria*	*Proteobacteria*	*Bacteria*
Winter 'no dilution' pH *in situ* (average similarity: 46.3%)											
Otu0010	7.33	3.29	11.97	7.1	7.1	unclass. *Flavobacteriaceae*	*Flavobacteriaceae*	*Flavobacteriales*	*Flavobacteria*	*Bacteroidetes*	*Bacteria*
Otu1815	5.57	2.24	3.87	4.83	11.93	unclass. *Flavobacteriales*	unclass. *Flavobacteriales*	*Flavobacteriales*	*Flavobacteria*	*Bacteroidetes*	*Bacteria*
Otu0041	4.85	1.98	13.44	4.28	16.22	*Sulfitobacter*	*Rhodobacteraceae*	*Rhodobacterales*	*Alphaproteobacteria*	*Proteobacteria*	*Bacteria*
Otu0134	4.57	1.9	5.21	4.1	20.31	unclass. *Gammaproteobacteria*	unclass. *Gammaproteobacteria*	unclass. *Gammaproteobacteria*	*Gammaproteobacteria*	*Proteobacteria*	*Bacteria*
Otu0236	4.16	1.83	8.98	3.94	24.26	unclass. *Gammaproteobacteria*	unclass. *Gammaproteobacteria*	unclass. *Gammaproteobacteria*	*Gammaproteobacteria*	*Proteobacteria*	*Bacteria*
Otu1283	4.37	1.75	3.81	3.77	28.03	*Croceibacter*	*Flavobacteriaceae*	*Flavobacteriales*	*Flavobacteria*	*Bacteroidetes*	*Bacteria*
Otu0055	3.73	1.51	5.73	3.26	31.29	*Colwellia*	*Colwelliaceae*	*Alteromonadales*	*Gammaproteobacteria*	*Proteobacteria*	*Bacteria*
Otu0068	3.4	1.38	5.01	2.98	34.28	*Pelagibacter*	SAR11-clade	*Rickettsiales*	*Alphaproteobacteria*	*Proteobacteria*	*Bacteria*
Otu0160	3.51	1.31	4.03	2.83	37.11	*Colwellia*	*Colwelliaceae*	*Alteromonadales*	*Gammaproteobacteria*	*Proteobacteria*	*Bacteria*
Otu0139	4.22	1.29	1.83	2.78	39.89	unclass. *Gammaproteobacteria*	unclass. *Gammaproteobacteria*	unclass. *Gammaproteobacteria*	*Gammaproteobacteria*	*Proteobacteria*	*Bacteria*
Otu1694	2.94	1.13	2	2.45	42.34	unclass. *Flavobacteriales*	unclass. *Flavobacteriales*	*Flavobacteriales*	*Flavobacteria*	*Bacteroidetes*	*Bacteria*
Otu0047	2.51	0.98	5.58	2.12	44.46	unclass. *Rhodobacteraceae*	*Rhodobacteraceae*	*Rhodobacterales*	*Alphaproteobacteria*	*Proteobacteria*	*Bacteria*
Otu1822	2.52	0.95	3.88	2.06	46.52	unclass. *Flavobacteriales*	unclass. *Flavobacteriales*	*Flavobacteriales*	*Flavobacteria*	*Bacteroidetes*	*Bacteria*

OTU	Av.A_i	Av.S_i	Av.S_i/SD	Av.S_i%	∑Av.S_i%	Genus	Family	Order	Class	Phylum	Domain
Otu0005	2.51	0.95	7.01	2.06	48.58	*Pelagibacter*	SAR11-clade	*Rickettsiales*	*Alphaproteobacteria*	*Proteobacteria*	*Bacteria*
Otu0469	1.92	0.81	6.53	1.74	50.32	unclass. *Bacteria*	unclass. *Bacteria*	unclass. *Bacteria*	unclass. *Bacteria*	unclass. *Bacteria*	*Bacteria*
Otu0164	1.94	0.78	2.95	1.68	52.01	*Croceibacter*	*Flavobacteriaceae*	*Flavobacteriales*	*Flavobacteria*	*Bacteroidetes*	*Bacteria*
Otu0032	1.79	0.73	3.37	1.57	53.58	unclass. *Gammaproteobacteria*	unclass. *Gammaproteobacteria*	unclass. *Gammaproteobacteria*	*Gammaproteobacteria*	*Proteobacteria*	*Bacteria*
Otu0157	1.75	0.72	10.86	1.56	55.14	unclass. *Gammaproteobacteria*	unclass. *Gammaproteobacteria*	unclass. *Gammaproteobacteria*	*Gammaproteobacteria*	*Proteobacteria*	*Bacteria*
Otu0001	1.91	0.72	2.72	1.55	56.69	unclass. *Flavobacteriaceae*	*Flavobacteriaceae*	*Flavobacteriales*	*Flavobacteria*	*Bacteroidetes*	*Bacteria*
Otu0163	1.87	0.71	2.88	1.54	58.23	unclass. *Gammaproteobacteria*	unclass. *Gammaproteobacteria*	unclass. *Gammaproteobacteria*	*Gammaproteobacteria*	*Proteobacteria*	*Bacteria*
Otu1720	1.86	0.7	2.92	1.5	59.73	unclass. *Bacteria*	unclass. *Bacteria*	unclass. *Bacteria*	unclass. *Bacteria*	unclass. *Bacteria*	*Bacteria*
Otu0076	1.63	0.66	3.63	1.42	61.15	unclass. *Flammeovirgaceae*	*Flammeovirgaceae*	*Sphingobacteriales*	*Sphingobacteria*	*Bacteroidetes*	*Bacteria*
Otu0035	1.78	0.64	2.42	1.39	62.54	*Colwellia*	*Colwelliaceae*	*Alteromonadales*	*Gammaproteobacteria*	*Proteobacteria*	*Bacteria*
Otu1146	1.58	0.64	4.45	1.39	63.92	*Nitrosospira*	*Nitrosomonadaceae*	*Nitrosomonadales*	*Betaproteobacteria*	*Proteobacteria*	*Bacteria*
Otu0124	1.51	0.61	4.81	1.33	65.25	*Loktanella*	*Rhodobacteraceae*	*Rhodobacterales*	*Alphaproteobacteria*	*Proteobacteria*	*Bacteria*
Otu0056	1.39	0.6	6.56	1.29	66.54	unclass. *Gammaproteobacteria*	unclass. *Gammaproteobacteria*	unclass. *Gammaproteobacteria*	*Gammaproteobacteria*	*Proteobacteria*	*Bacteria*
Otu0270	1.55	0.6	3.18	1.29	67.83	unclass. *Bacteroidetes*	unclass. *Bacteroidetes*	unclass. *Bacteroidetes*	unclass. *Bacteroidetes*	*Bacteroidetes*	*Bacteria*
Otu0012	1.49	0.54	5.94	1.16	68.99	unclass. *Comamonadaceae*	*Comamonadaceae*	*Burkholderiales*	*Betaproteobacteria*	*Proteobacteria*	*Bacteria*
Otu0113	1.91	0.54	1.06	1.16	70.14	*Colwellia*	*Colwelliaceae*	*Alteromonadales*	*Gammaproteobacteria*	*Proteobacteria*	*Bacteria*
Otu0044	1.35	0.51	4.74	1.11	71.25	unclass. *Gammaproteobacteria*	unclass. *Gammaproteobacteria*	unclass. *Gammaproteobacteria*	*Gammaproteobacteria*	*Proteobacteria*	*Bacteria*
Otu0177	1.17	0.5	7.33	1.08	72.33	*Haliea*	*Alteromonadaceae*	*Alteromonadales*	*Gammaproteobacteria*	*Proteobacteria*	*Bacteria*
Otu0262	1.17	0.5	8.16	1.08	73.41	unclass. *Gammaproteobacteria*	unclass. *Gammaproteobacteria*	unclass. *Gammaproteobacteria*	*Gammaproteobacteria*	*Proteobacteria*	*Bacteria*
Otu0291	1.23	0.5	8.52	1.08	74.49	unclass. *Flavobacteriaceae*	*Flavobacteriaceae*	*Flavobacteriales*	*Flavobacteria*	*Bacteroidetes*	*Bacteria*
Otu0572	2.33	0.45	0.94	0.96	75.45	*Croceibacter*	*Flavobacteriaceae*	*Flavobacteriales*	*Flavobacteria*	*Bacteroidetes*	*Bacteria*
Otu0122	1.58	0.45	1.01	0.96	76.41	*Glaciecola*	*Alteromonadaceae*	*Alteromonadales*	*Gammaproteobacteria*	*Proteobacteria*	*Bacteria*
Otu0261	1.51	0.42	1.15	0.91	77.32	*Winogradskyella*	*Flavobacteriaceae*	*Flavobacteriales*	*Flavobacteria*	*Bacteroidetes*	*Bacteria*
Otu0143	1.28	0.37	1.09	0.79	78.11	unclass. *Gammaproteobacteria*	unclass. *Gammaproteobacteria*	unclass. *Gammaproteobacteria*	*Gammaproteobacteria*	*Proteobacteria*	*Bacteria*
Otu0022	1.34	0.35	0.98	0.76	78.87	unclass. *Betaproteobacteria*	unclass. *Betaproteobacteria*	unclass. *Betaproteobacteria*	*Betaproteobacteria*	*Proteobacteria*	*Bacteria*
Otu0002	1.11	0.35	1.12	0.75	79.62	unclass. *Flavobacteriaceae*	*Flavobacteriaceae*	*Flavobacteriales*	*Flavobacteria*	*Bacteroidetes*	*Bacteria*
Otu0052	1.38	0.33	0.62	0.71	80.33	unclass. *Betaproteobacteria*	unclass. *Betaproteobacteria*	unclass. *Betaproteobacteria*	*Betaproteobacteria*	*Proteobacteria*	*Bacteria*

OTU	Av.A_i	Av.S_i	Av.S_i/SD	Av.S_i%	∑Av.S_i%	Genus	Family	Order	Class	Phylum	Domain
Otu0140	1.09	0.32	1.08	0.7	81.03	*Oleispira*	*Oceanospirillaceae*	*Oceanospirillales*	*Gammaproteobacteria*	*Proteobacteria*	*Bacteria*
Otu0268	1.15	0.32	1.08	0.7	81.73	unclass. *Flavobacteriaceae*	*Flavobacteriaceae*	*Flavobacteriales*	*Flavobacteria*	*Bacteroidetes*	*Bacteria*
Otu0366	1.09	0.32	1.09	0.68	82.41	unclass. *Saprospiraceae*	*Saprospiraceae*	*Sphingobacteriales*	*Sphingobacteria*	*Bacteroidetes*	*Bacteria*
Otu0009	0.97	0.31	1.13	0.67	83.08	unclass. *Rhodobacteraceae*	*Rhodobacteraceae*	*Rhodobacterales*	*Alphaproteobacteria*	*Proteobacteria*	*Bacteria*
Otu0133	0.97	0.3	1.13	0.65	83.73	*Colwellia*	*Colwelliaceae*	*Alteromonadales*	*Gammaproteobacteria*	*Proteobacteria*	*Bacteria*
Otu1723	1.08	0.3	1.13	0.65	84.39	unclass. *Bacteroidetes*	unclass. *Bacteroidetes*	unclass. *Bacteroidetes*	unclass. *Bacteroidetes*	*Bacteroidetes*	*Bacteria*
Otu0203	0.88	0.29	1.16	0.62	85.01	*Pelagibacter*	SAR11-clade	*Rickettsiales*	*Alphaproteobacteria*	*Proteobacteria*	*Bacteria*
Otu1816	0.8	0.29	1.16	0.62	85.63	unclass. *Flavobacteriales*	unclass. *Flavobacteriales*	*Flavobacteriales*	*Flavobacteria*	*Bacteroidetes*	*Bacteria*
Otu0170	0.88	0.29	1.16	0.62	86.26	unclass. *Bacteria*	unclass. *Bacteria*	unclass. *Bacteria*	unclass. *Bacteria*	unclass. *Bacteria*	*Bacteria*
Otu0348	0.8	0.29	1.16	0.62	86.88	unclass. *Flavobacteriaceae*	*Flavobacteriaceae*	*Flavobacteriales*	*Flavobacteria*	*Bacteroidetes*	*Bacteria*
Otu0046	0.95	0.28	1.16	0.61	87.49	unclass. *Flavobacteriaceae*	*Flavobacteriaceae*	*Flavobacteriales*	*Flavobacteria*	*Bacteroidetes*	*Bacteria*
Otu0369	0.8	0.28	1.16	0.61	88.1	unclass. *Deltaproteobacteria*	unclass. *Deltaproteobacteria*	unclass. *Deltaproteobacteria*	*Deltaproteobacteria*	*Proteobacteria*	*Bacteria*
Otu1837	0.88	0.28	1.16	0.61	88.71	unclass. *Flavobacteriales*	unclass. *Flavobacteriales*	*Flavobacteriales*	*Flavobacteria*	*Bacteroidetes*	*Bacteria*
Otu0013	1.09	0.25	0.62	0.55	89.26	unclass. *Flavobacteriaceae*	*Flavobacteriaceae*	*Flavobacteriales*	*Flavobacteria*	*Bacteroidetes*	*Bacteria*
Otu0062	0.95	0.18	0.59	0.39	89.65	*Haliea*	*Alteromonadaceae*	*Alteromonadales*	*Gammaproteobacteria*	*Proteobacteria*	*Bacteria*
Otu0118	1.04	0.18	0.59	0.38	90.03	*Colwellia*	*Colwelliaceae*	*Alteromonadales*	*Gammaproteobacteria*	*Proteobacteria*	*Bacteria*
Winter 'no dilution' pH 7.67 (average similarity: 40.6%)											
Otu0010	6.83	2.94	13.81	7.25	7.25	unclass. *Flavobacteriaceae*	*Flavobacteriaceae*	*Flavobacteriales*	*Flavobacteria*	*Bacteroidetes*	*Bacteria*
Otu0055	6.14	2.42	8.96	5.95	13.2	*Colwellia*	*Colwelliaceae*	*Alteromonadales*	*Gammaproteobacteria*	*Proteobacteria*	*Bacteria*
Otu0139	5.78	1.96	3.12	4.83	18.03	unclass. *Gammaproteobacteria*	unclass. *Gammaproteobacteria*	unclass. *Gammaproteobacteria*	*Gammaproteobacteria*	*Proteobacteria*	*Bacteria*
Otu1692	3.71	1.49	10.87	3.68	21.71	unclass. *Rhodobacteraceae*	*Rhodobacteraceae*	*Rhodobacterales*	*Alphaproteobacteria*	*Proteobacteria*	*Bacteria*
Otu0160	3.81	1.49	6.83	3.67	25.39	*Colwellia*	*Colwelliaceae*	*Alteromonadales*	*Gammaproteobacteria*	*Proteobacteria*	*Bacteria*
Otu1815	4.03	1.47	3.24	3.61	29	unclass. *Flavobacteriales*	unclass. *Flavobacteriales*	*Flavobacteriales*	*Flavobacteria*	*Bacteroidetes*	*Bacteria*
Otu0134	3.58	1.35	3.89	3.32	32.32	unclass. *Gammaproteobacteria*	unclass. *Gammaproteobacteria*	unclass. *Gammaproteobacteria*	*Gammaproteobacteria*	*Proteobacteria*	*Bacteria*
Otu0366	3.02	1.3	14.75	3.21	35.54	unclass. *Saprospiraceae*	*Saprospiraceae*	*Sphingobacteriales*	*Sphingobacteria*	*Bacteroidetes*	*Bacteria*
Otu0469	3.36	1.27	5.6	3.12	38.66	unclass. *Bacteria*	unclass. *Bacteria*	unclass. *Bacteria*	unclass. *Bacteria*	unclass. *Bacteria*	*Bacteria*
Otu0068	3.42	1.27	4.56	3.12	41.78	*Pelagibacter*	SAR11-clade	*Rickettsiales*	*Alphaproteobacteria*	*Proteobacteria*	*Bacteria*

OTU	Av.A_i	Av.S_i	Av.S_i/SD	Av.S_i%	∑Av.S_i%	Genus	Family	Order	Class	Phylum	Domain
Otu0047	3.28	1.24	3.82	3.06	44.84	unclass. *Rhodobacteraceae*	*Rhodobacteraceae*	*Rhodobacterales*	*Alphaproteobacteria*	*Proteobacteria*	*Bacteria*
Otu0005	2.76	1.03	7.32	2.55	47.39	*Pelagibacter*	SAR11-clade	*Rickettsiales*	*Alphaproteobacteria*	*Proteobacteria*	*Bacteria*
Otu0066	2.32	0.85	3.69	2.09	49.48	*Granulosicoccus*	*Granulosicoccaceae*	*Chromatiales*	*Gammaproteobacteria*	*Proteobacteria*	*Bacteria*
Otu0035	2.06	0.75	4.19	1.84	51.32	*Colwellia*	*Colwelliaceae*	*Alteromonadales*	*Gammaproteobacteria*	*Proteobacteria*	*Bacteria*
Otu1283	2.5	0.7	2.44	1.73	53.06	*Croceibacter*	*Flavobacteriaceae*	*Flavobacteriales*	*Flavobacteria*	*Bacteroidetes*	*Bacteria*
Otu0012	1.91	0.7	7.72	1.73	54.78	unclass. *Comamonadaceae*	*Comamonadaceae*	*Burkholderiales*	*Betaproteobacteria*	*Proteobacteria*	*Bacteria*
Otu0052	1.66	0.69	8.94	1.7	56.49	unclass. *Betaproteobacteria*	unclass. *Betaproteobacteria*	unclass. *Betaproteobacteria*	*Betaproteobacteria*	*Proteobacteria*	*Bacteria*
Otu1822	1.87	0.62	1.16	1.54	58.02	unclass. *Flavobacteriales*	unclass. *Flavobacteriales*	*Flavobacteriales*	*Flavobacteria*	*Bacteroidetes*	*Bacteria*
Otu0157	1.46	0.59	4.76	1.44	59.47	unclass. *Gammaproteobacteria*	unclass. *Gammaproteobacteria*	unclass. *Gammaproteobacteria*	*Gammaproteobacteria*	*Proteobacteria*	*Bacteria*
Otu0261	1.66	0.55	2.86	1.36	60.83	*Winogradskyella*	*Flavobacteriaceae*	*Flavobacteriales*	*Flavobacteria*	*Bacteroidetes*	*Bacteria*
Otu0013	1.35	0.49	4.29	1.21	62.04	unclass. *Flavobacteriaceae*	*Flavobacteriaceae*	*Flavobacteriales*	*Flavobacteria*	*Bacteroidetes*	*Bacteria*
Otu0076	1.64	0.48	1.16	1.18	63.22	unclass. *Flammeovirgaceae*	*Flammeovirgaceae*	*Sphingobacteriales*	*Sphingobacteria*	*Bacteroidetes*	*Bacteria*
Otu0267	1.23	0.48	8.25	1.17	64.39	unclass. *Flavobacteriaceae*	*Flavobacteriaceae*	*Flavobacteriales*	*Flavobacteria*	*Bacteroidetes*	*Bacteria*
Otu0713	1.17	0.48	8.36	1.17	65.57	unclass. *Bacteria*	unclass. *Bacteria*	unclass. *Bacteria*	unclass. *Bacteria*	unclass. *Bacteria*	*Bacteria*
Otu0341	1.38	0.43	1.14	1.06	66.63	unclass. *Gammaproteobacteria*	unclass. *Gammaproteobacteria*	unclass. *Gammaproteobacteria*	*Gammaproteobacteria*	*Proteobacteria*	*Bacteria*
Otu0163	1.46	0.38	1.07	0.93	67.56	unclass. *Gammaproteobacteria*	unclass. *Gammaproteobacteria*	unclass. *Gammaproteobacteria*	*Gammaproteobacteria*	*Proteobacteria*	*Bacteria*
Otu0262	1.29	0.37	1.07	0.91	68.47	unclass. *Gammaproteobacteria*	unclass. *Gammaproteobacteria*	unclass. *Gammaproteobacteria*	*Gammaproteobacteria*	*Proteobacteria*	*Bacteria*
Otu1694	1.78	0.37	0.62	0.9	69.37	unclass. *Flavobacteriales*	unclass. *Flavobacteriales*	*Flavobacteriales*	*Flavobacteria*	*Bacteroidetes*	*Bacteria*
Otu0213	1.05	0.33	1.12	0.82	70.19	unclass. *Gammaproteobacteria*	unclass. *Gammaproteobacteria*	unclass. *Gammaproteobacteria*	*Gammaproteobacteria*	*Proteobacteria*	*Bacteria*
Otu0022	1.09	0.31	1.08	0.77	70.95	unclass. *Betaproteobacteria*	unclass. *Betaproteobacteria*	unclass. *Betaproteobacteria*	*Betaproteobacteria*	*Proteobacteria*	*Bacteria*
Otu0001	1.24	0.31	1.08	0.76	71.71	unclass. *Flavobacteriaceae*	*Flavobacteriaceae*	*Flavobacteriales*	*Flavobacteria*	*Bacteroidetes*	*Bacteria*
Otu0094	1.09	0.3	1.08	0.75	72.46	unclass. *Flavobacteriaceae*	*Flavobacteriaceae*	*Flavobacteriales*	*Flavobacteria*	*Bacteroidetes*	*Bacteria*
Otu0032	1.03	0.3	1.13	0.73	73.19	unclass. *Gammaproteobacteria*	unclass. *Gammaproteobacteria*	unclass. *Gammaproteobacteria*	*Gammaproteobacteria*	*Proteobacteria*	*Bacteria*
Otu0270	1.03	0.29	1.14	0.73	73.91	unclass. *Bacteroidetes*	unclass. *Bacteroidetes*	unclass. *Bacteroidetes*	unclass. *Bacteroidetes*	*Bacteroidetes*	*Bacteria*
Otu0291	1.03	0.29	1.14	0.73	74.64	unclass. *Flavobacteriaceae*	*Flavobacteriaceae*	*Flavobacteriales*	*Flavobacteria*	*Bacteroidetes*	*Bacteria*
Otu0177	1.03	0.29	1.14	0.72	75.36	*Haliea*	*Alteromonadaceae*	*Alteromonadales*	*Gammaproteobacteria*	*Proteobacteria*	*Bacteria*
Otu0113	0.8	0.28	1.16	0.68	76.04	*Colwellia*	*Colwelliaceae*	*Alteromonadales*	*Gammaproteobacteria*	*Proteobacteria*	*Bacteria*

OTU	$Av.A_i$	$Av.S_i$	$Av.S_i/SD$	$Av.S_i\%$	$\sum Av.S_i\%$	Genus	Family	Order	Class	Phylum	Domain
Otu0056	0.95	0.28	1.16	0.68	76.72	unclass. *Gammaproteobacteria*	unclass. *Gammaproteobacteria*	unclass. *Gammaproteobacteria*	*Gammaproteobacteria*	*Proteobacteria*	*Bacteria*
Otu0621	0.88	0.28	1.16	0.68	77.41	*Colwellia*	*Colwelliaceae*	*Alteromonadales*	*Gammaproteobacteria*	*Proteobacteria*	*Bacteria*
Otu0025	1	0.27	1.16	0.68	78.08	*Polaribacter*	*Flavobacteriaceae*	*Flavobacteriales*	*Flavobacteria*	*Bacteroidetes*	*Bacteria*
Otu0232	0.88	0.27	1.16	0.68	78.76	unclass. *Rhodobacteraceae*	*Rhodobacteraceae*	*Rhodobacterales*	*Alphaproteobacteria*	*Proteobacteria*	*Bacteria*
Otu0009	1	0.27	1.16	0.68	79.43	unclass. *Rhodobacteraceae*	*Rhodobacteraceae*	*Rhodobacterales*	*Alphaproteobacteria*	*Proteobacteria*	*Bacteria*
Otu0042	0.88	0.27	1.16	0.68	80.11	*Rhodococcus*	*Nocardiaceae*	*Actinomycetales*	*Actinobacteria*	*Actinobacteria*	*Bacteria*
Otu0234	0.88	0.27	1.16	0.68	80.78	*Arcobacter*	*Campylobacteraceae*	*Campylobacterales*	*Epsilonproteobacteria*	*Proteobacteria*	*Bacteria*
Otu0508	0.8	0.27	1.16	0.68	81.46	unclass. *Flavobacteriales*	unclass. *Flavobacteriales*	*Flavobacteriales*	*Flavobacteria*	*Bacteroidetes*	*Bacteria*
Otu0172	0.88	0.27	1.16	0.67	82.13	unclass. *Gammaproteobacteria*	unclass. *Gammaproteobacteria*	unclass. *Gammaproteobacteria*	*Gammaproteobacteria*	*Proteobacteria*	*Bacteria*
Otu1119	0.8	0.27	1.16	0.67	82.79	unclass. *Bacteroidetes*	unclass. *Bacteroidetes*	unclass. *Bacteroidetes*	unclass. *Bacteroidetes*	*Bacteroidetes*	*Bacteria*
Otu1720	1.22	0.23	0.6	0.58	83.37	unclass. *Bacteria*	unclass. *Bacteria*	unclass. *Bacteria*	unclass. *Bacteria*	unclass. *Bacteria*	*Bacteria*
Otu0041	1.09	0.23	0.62	0.57	83.94	*Sulfitobacter*	*Rhodobacteraceae*	*Rhodobacterales*	*Alphaproteobacteria*	*Proteobacteria*	*Bacteria*
Otu0026	1.22	0.23	0.6	0.57	84.51	unclass. *Rhodobacteraceae*	*Rhodobacteraceae*	*Rhodobacterales*	*Alphaproteobacteria*	*Proteobacteria*	*Bacteria*
Otu1937	0.91	0.2	0.62	0.49	85	unclass. *Gammaproteobacteria*	unclass. *Gammaproteobacteria*	unclass. *Gammaproteobacteria*	*Gammaproteobacteria*	*Proteobacteria*	*Bacteria*
Otu0062	1.17	0.19	0.62	0.47	85.47	*Haliea*	*Alteromonadaceae*	*Alteromonadales*	*Gammaproteobacteria*	*Proteobacteria*	*Bacteria*
Otu1828	1.09	0.19	0.57	0.46	85.92	unclass. *Chromatiales*	unclass. *Chromatiales*	*Chromatiales*	*Gammaproteobacteria*	*Proteobacteria*	*Bacteria*
Otu0753	1.09	0.19	0.57	0.46	86.38	unclass. *Flavobacteriales*	unclass. *Flavobacteriales*	*Flavobacteriales*	*Flavobacteria*	*Bacteroidetes*	*Bacteria*
Otu0164	0.93	0.16	0.61	0.39	86.77	*Croceibacter*	*Flavobacteriaceae*	*Flavobacteriales*	*Flavobacteria*	*Bacteroidetes*	*Bacteria*
Otu1146	0.83	0.15	0.61	0.38	87.15	*Nitrosospira*	*Nitrosomonadaceae*	*Nitrosomonadales*	*Betaproteobacteria*	*Proteobacteria*	*Bacteria*
Otu0124	1.01	0.15	0.61	0.38	87.53	*Loktanella*	*Rhodobacteraceae*	*Rhodobacterales*	*Alphaproteobacteria*	*Proteobacteria*	*Bacteria*
Otu0203	0.83	0.15	0.61	0.38	87.91	*Pelagibacter*	SAR11-clade	*Rickettsiales*	*Alphaproteobacteria*	*Proteobacteria*	*Bacteria*
Otu0140	0.77	0.15	0.61	0.38	88.29	*Oleispira*	*Oceanospirillaceae*	*Oceanospirillales*	*Gammaproteobacteria*	*Proteobacteria*	*Bacteria*
Otu1723	0.77	0.15	0.61	0.38	88.67	unclass. *Bacteroidetes*	unclass. *Bacteroidetes*	unclass. *Bacteroidetes*	unclass. *Bacteroidetes*	*Bacteroidetes*	*Bacteria*
Otu0359	0.68	0.14	0.62	0.34	89.01	unclass. *Gammaproteobacteria*	unclass. *Gammaproteobacteria*	unclass. *Gammaproteobacteria*	*Gammaproteobacteria*	*Proteobacteria*	*Bacteria*
Otu0282	0.68	0.14	0.62	0.34	89.36	*Haliea*	*Alteromonadaceae*	*Alteromonadales*	*Gammaproteobacteria*	*Proteobacteria*	*Bacteria*
Otu0313	0.68	0.14	0.62	0.34	89.7	unclass. *Gammaproteobacteria*	unclass. *Gammaproteobacteria*	unclass. *Gammaproteobacteria*	*Gammaproteobacteria*	*Proteobacteria*	*Bacteria*
Otu0878	0.6	0.14	0.62	0.34	90.04	unclass. *Bacteria*	unclass. *Bacteria*	unclass. *Bacteria*	unclass. *Bacteria*	unclass. *Bacteria*	*Bacteria*

OTU	$Av.A_i$	$Av.S_i$	$Av.S_i/SD$	$Av.S_i\%$	$\sum Av.S_i\%$	Genus	Family	Order	Class	Phylum	Domain
Winter 'serial dilution' pH *in situ* (average similarity: 67.6%)											
Otu0106	12.27	15.17	6.64	22.43	22.43	*Marinomonas*	*Oceanospirillaceae*	*Oceanospirillales*	*Gammaproteobacteria*	*Proteobacteria*	*Bacteria*
Otu0132	8.64	10.31	12.97	15.24	37.68	unclass. *Alteromonadales*	unclass. *Alteromonadales*	*Alteromonadales*	*Gammaproteobacteria*	*Proteobacteria*	*Bacteria*
Otu0055	9.17	9.42	1.68	13.93	51.61	*Colwellia*	*Colwelliaceae*	*Alteromonadales*	*Gammaproteobacteria*	*Proteobacteria*	*Bacteria*
Otu1777	5.49	6.5	5.78	9.61	61.22	*Oceaniserpentilla*	*Oceanospirillaceae*	*Oceanospirillales*	*Gammaproteobacteria*	*Proteobacteria*	*Bacteria*
Otu0160	5.46	5.3	2.27	7.83	69.05	*Colwellia*	*Colwelliaceae*	*Alteromonadales*	*Gammaproteobacteria*	*Proteobacteria*	*Bacteria*
Otu0123	5.62	5.01	3.57	7.41	76.45	unclass. *Gammaproteobacteria*	unclass. *Gammaproteobacteria*	unclass. *Gammaproteobacteria*	*Gammaproteobacteria*	*Proteobacteria*	*Bacteria*
Otu0471	4.28	4.46	3.87	6.59	83.04	*Oceaniserpentilla*	*Oceanospirillaceae*	*Oceanospirillales*	*Gammaproteobacteria*	*Proteobacteria*	*Bacteria*
Otu0571	1.95	2.27	4.25	3.35	86.4	*Marinomonas*	*Oceanospirillaceae*	*Oceanospirillales*	*Gammaproteobacteria*	*Proteobacteria*	*Bacteria*
Otu0035	2.68	2.22	3.52	3.28	89.67	*Colwellia*	*Colwelliaceae*	*Alteromonadales*	*Gammaproteobacteria*	*Proteobacteria*	*Bacteria*
Otu0209	1.54	1.53	3.16	2.26	91.93	unclass. *Oceanospirillales*	unclass. *Oceanospirillales*	*Oceanospirillales*	*Gammaproteobacteria*	*Proteobacteria*	*Bacteria*
Winter 'serial dilution' pH 7.67 (average similarity: 73.6%)											
Otu0117	14.6	18.3	9.49	24.85	24.85	*Arcobacter*	*Campylobacteraceae*	*Campylobacterales*	*Epsilonproteobacteria*	*Proteobacteria*	*Bacteria*
Otu0106	7.72	8.81	4.15	11.96	36.81	*Marinomonas*	*Oceanospirillaceae*	*Oceanospirillales*	*Gammaproteobacteria*	*Proteobacteria*	*Bacteria*
Otu1748	6.37	7.52	5.26	10.21	47.02	unclass. *Epsilonproteobacteria*	unclass. *Epsilonproteobacteria*	unclass. *Epsilonproteobacteria*	*Epsilonproteobacteria*	*Proteobacteria*	*Bacteria*
Otu1777	5.32	6.56	8.77	8.91	55.93	*Oceaniserpentilla*	*Oceanospirillaceae*	*Oceanospirillales*	*Gammaproteobacteria*	*Proteobacteria*	*Bacteria*
Otu0234	4.68	5.34	5.33	7.25	63.18	*Arcobacter*	*Campylobacteraceae*	*Campylobacterales*	*Epsilonproteobacteria*	*Proteobacteria*	*Bacteria*
Otu0686	5.23	4.89	2.28	6.64	69.83	*Marinomonas*	*Oceanospirillaceae*	*Oceanospirillales*	*Gammaproteobacteria*	*Proteobacteria*	*Bacteria*
Otu0132	4.05	4.69	6.41	6.36	76.19	unclass. *Alteromonadales*	unclass. *Alteromonadales*	*Alteromonadales*	*Gammaproteobacteria*	*Proteobacteria*	*Bacteria*
Otu0055	4.54	3.89	2.05	5.28	81.47	*Colwellia*	*Colwelliaceae*	*Alteromonadales*	*Gammaproteobacteria*	*Proteobacteria*	*Bacteria*
Otu0123	2.75	2.66	5.33	3.61	85.08	unclass. *Gammaproteobacteria*	unclass. *Gammaproteobacteria*	unclass. *Gammaproteobacteria*	*Gammaproteobacteria*	*Proteobacteria*	*Bacteria*
Otu1780	1.81	1.89	2.99	2.57	87.65	unclass. *Oceanospirillales*	unclass. *Oceanospirillales*	*Oceanospirillales*	*Gammaproteobacteria*	*Proteobacteria*	*Bacteria*
Otu1801	1.31	1.51	4.86	2.05	89.7	*Arcobacter*	*Campylobacteraceae*	*Campylobacterales*	*Epsilonproteobacteria*	*Proteobacteria*	*Bacteria*
Otu0209	1.38	1.1	1.08	1.49	91.19	unclass. *Oceanospirillales*	unclass. *Oceanospirillales*	*Oceanospirillales*	*Gammaproteobacteria*	*Proteobacteria*	*Bacteria*
