# Supplementary material for: Small Changes in pH Have Direct Effects on Marine Bacterial Community Composition: A Microcosm Approach
Source: PLoS One. 2012 Oct 11;7(10):e47035. doi: 10.1371/journal.pone.0047035 (PMC3469576; doi:10.1371/journal.pone.0047035)
Supplement: Table S4 — Results of the SIMPER analysis giving the dissimilarities between pH in situ levels of the different dilution treatments, separately for each season. Displayed are the OTUs (16S ribosomal amplicon pyrosequencing) that predominantly contributed to 90% of to the total dissimilarity. Av.Ai: average abundance of the i th species over all samples of the treatment (no: ‘no dilution’, serial: ‘serial dilution’, initial: ‘initial dilution’), Av.δi: average contribution of the i th species to the total dissimilarity, Av.δi/SD: the average value of the i th species as a discriminating species, Av.δi%: average percentage contribution of the i th species to the total dissimilarity, ∑Av.δi%: average cumulative contribution to the total dissimilarity. (PDF) [file pone.0047035.s008.pdf]

Table S4. Results of the SIMPER analysis giving the dissimilarities between pH *in situ* levels of the different dilution treatments, separately for each season.

Displayed are the OTUs (16S ribosomal amplicon pyrosequencing) that predominantly contributed to 90% of to the total dissimilarity. $Av.A_i$: average abundance of the i th species over all samples of the treatment (no: ‘no dilution’, serial: ‘serial dilution’, initial: ‘initial dilution’), $Av.\delta_i$: average contribution of the i th species to the total dissimilarity, $Av.\delta_i/SD$: the average value of the i th species as a discriminating species, $Av.\delta_i\%$: average percentage contribution of the i th species to the total dissimilarity, $\sum Av.\delta_i\%$: average cumulative contribution to the total dissimilarity.

OTU	$Av.A_i$ (no)	$Av.A_i$ (serial)	$Av.\delta_i$	$Av.\delta_i/SD$	$Av.\delta_i$ %	$\sum Av.\delta_i$ %	Genus	Family	Order	Class	Phylum	Domain
Spring pH *in situ* 'no dilution' vs. 'serial dilution' (average dissimilarity: 93.1%)												
Otu0027	1.37	11.23	5.93	1.51	6.37	6.37	*Pseudoalteromonas*	*Pseudoalteromonadaceae*	*Alteromonadales*	*Gammaproteobacteria*	*Proteobacteria*	*Bacteria*
Otu0005	9.45	0	5.65	2.92	6.07	12.44	*Pelagibacter*	SAR11-clade	*Rickettsiales*	*Alphaproteobacteria*	*Proteobacteria*	*Bacteria*
Otu0035	0.97	9.32	5.05	1.18	5.43	17.87	*Colwellia*	*Colwelliaceae*	*Alteromonadales*	*Gammaproteobacteria*	*Proteobacteria*	*Bacteria*
Otu0106	0	7.05	4.29	2.19	4.61	22.48	*Marinomonas*	*Oceanospirillaceae*	*Oceanospirillales*	*Gammaproteobacteria*	*Proteobacteria*	*Bacteria*
Otu0134	7.21	0	4.28	3.58	4.6	27.08	unclass. *Gammaproteobacteria*	unclass. *Gammaproteobacteria*	unclass. *Gammaproteobacteria*	*Gammaproteobacteria*	*Proteobacteria*	*Bacteria*
Otu0002	6.43	0	3.77	2.16	4.05	31.14	unclass. *Flavobacteriaceae*	*Flavobacteriaceae*	*Flavobacteriales*	*Flavobacteria*	*Bacteroidetes*	*Bacteria*
Otu0001	6.06	0	3.57	1.67	3.83	34.97	unclass. *Flavobacteriaceae*	*Flavobacteriaceae*	*Flavobacteriales*	*Flavobacteria*	*Bacteroidetes*	*Bacteria*
Otu0029	5.69	0	3.37	4.89	3.62	38.6	unclass. *Bacteroidetes*	unclass. *Bacteroidetes*	unclass. *Bacteroidetes*	unclass. *Bacteroidetes*	*Bacteroidetes*	*Bacteria*
Otu0022	5.16	0	3.06	5.65	3.29	41.89	unclass. *Betaproteobacteria*	unclass. *Betaproteobacteria*	unclass. *Betaproteobacteria*	*Betaproteobacteria*	*Proteobacteria*	*Bacteria*
Otu0008	5.12	0	3.04	5.33	3.26	45.15	unclass. *Gammaproteobacteria*	unclass. *Gammaproteobacteria*	unclass. *Gammaproteobacteria*	*Gammaproteobacteria*	*Proteobacteria*	*Bacteria*
Otu0010	3.32	3.24	2.74	1.28	2.95	48.1	unclass. *Flavobacteriaceae*	*Flavobacteriaceae*	*Flavobacteriales*	*Flavobacteria*	*Bacteroidetes*	*Bacteria*
Otu0012	4.52	0	2.67	3.07	2.87	50.97	unclass. *Comamonadaceae*	*Comamonadaceae*	*Burkholderiales*	*Betaproteobacteria*	*Proteobacteria*	*Bacteria*

OTU	Av.A$_i$ (no)	Av.A$_i$ (serial)	Av.δ$_i$	Av.δ$_i$/SD	Av.δ$_i$ %	∑Av.δ$_i$ %	Genus	Family	Order	Class	Phylum	Domain
Otu0107	0	4.07	2.39	3.76	2.56	53.53	unclass. *Rhodobacteraceae*	*Rhodobacteraceae*	*Rhodobacterales*	*Alphaproteobacteria*	*Proteobacteria*	*Bacteria*
Otu0014	3.76	0	2.24	1.81	2.41	55.94	*Reinekea*	*Oceanospirillaceae*	*Oceanospirillales*	*Gammaproteobacteria*	*Proteobacteria*	*Bacteria*
Otu0135	3.24	0	1.92	2.74	2.06	58	unclass. *Gammaproteobacteria*	unclass. *Gammaproteobacteria*	unclass. *Gammaproteobacteria*	*Gammaproteobacteria*	*Proteobacteria*	*Bacteria*
Otu0007	3.2	0	1.89	1.93	2.03	60.03	unclass. *Flavobacteriales*	unclass. *Flavobacteriales*	*Flavobacteriales*	*Flavobacteria*	*Bacteroidetes*	*Bacteria*
Otu0036	0.75	3.68	1.75	1.91	1.88	61.91	*Glaciecola*	*Alteromonadaceae*	*Alteromonadales*	*Gammaproteobacteria*	*Proteobacteria*	*Bacteria*
Otu0013	2.74	0.2	1.51	2.25	1.62	63.53	unclass. *Flavobacteriaceae*	*Flavobacteriaceae*	*Flavobacteriales*	*Flavobacteria*	*Bacteroidetes*	*Bacteria*
Otu0111	0	2.28	1.42	1.4	1.52	65.05	*Marinomonas*	*Oceanospirillaceae*	*Oceanospirillales*	*Gammaproteobacteria*	*Proteobacteria*	*Bacteria*
Otu0018	1.91	0	1.12	2.71	1.21	66.26	unclass. *Puniceicoccaceae*	*Puniceicoccaceae*	*Puniceicoccales*	*Opitutae*	*Verrucomicrobia*	*Bacteria*
Otu0020	1.78	0	1.07	1.53	1.15	67.41	unclass. *Flammeovirgaceae*	*Flammeovirgaceae*	*Sphingobacteriales*	*Sphingobacteria*	*Bacteroidetes*	*Bacteria*
Otu0139	1.76	0	1.03	1.68	1.11	68.52	unclass. *Gammaproteobacteria*	unclass. *Gammaproteobacteria*	unclass. *Gammaproteobacteria*	*Gammaproteobacteria*	*Proteobacteria*	*Bacteria*
Otu0113	0.6	1.95	1.02	1.1	1.09	69.61	*Colwellia*	*Colwelliaceae*	*Alteromonadales*	*Gammaproteobacteria*	*Proteobacteria*	*Bacteria*
Otu0044	1.63	0	0.96	4.27	1.03	70.64	unclass. *Gammaproteobacteria*	unclass. *Gammaproteobacteria*	unclass. *Gammaproteobacteria*	*Gammaproteobacteria*	*Proteobacteria*	*Bacteria*
Otu0056	1.59	0	0.95	2.9	1.02	71.66	unclass. *Gammaproteobacteria*	unclass. *Gammaproteobacteria*	unclass. *Gammaproteobacteria*	*Gammaproteobacteria*	*Proteobacteria*	*Bacteria*
Otu0109	0	1.72	0.91	0.58	0.97	72.63	*Alteromonas*	*Alteromonadaceae*	*Alteromonadales*	*Gammaproteobacteria*	*Proteobacteria*	*Bacteria*
Otu0015	1.5	0	0.89	1.68	0.95	73.59	*Polaribacter*	*Flavobacteriaceae*	*Flavobacteriales*	*Flavobacteria*	*Bacteroidetes*	*Bacteria*
Otu0052	1.49	0	0.88	2.59	0.94	74.53	unclass. *Betaproteobacteria*	unclass. *Betaproteobacteria*	unclass. *Betaproteobacteria*	*Betaproteobacteria*	*Proteobacteria*	*Bacteria*
Otu0041	1.76	0.89	0.81	1.43	0.88	75.4	*Sulfitobacter*	*Rhodobacteraceae*	*Rhodobacterales*	*Alphaproteobacteria*	*Proteobacteria*	*Bacteria*
Otu0108	0	1.4	0.79	1.26	0.85	76.25	unclass. *Colwelliaceae*	*Colwelliaceae*	*Alteromonadales*	*Gammaproteobacteria*	*Proteobacteria*	*Bacteria*
Otu0059	0	1.29	0.77	1	0.83	77.08	*Flavobacterium*	*Flavobacteriaceae*	*Flavobacteriales*	*Flavobacteria*	*Bacteroidetes*	*Bacteria*
Otu0123	0	1.12	0.66	0.96	0.71	77.79	unclass. *Gammaproteobacteria*	unclass. *Gammaproteobacteria*	unclass. *Gammaproteobacteria*	*Gammaproteobacteria*	*Proteobacteria*	*Bacteria*
Otu0132	0	1.08	0.65	1.14	0.7	78.48	unclass. *Alteromonadales*	unclass. *Alteromonadales*	*Alteromonadales*	*Gammaproteobacteria*	*Proteobacteria*	*Bacteria*
Otu0017	1	0	0.57	0.78	0.61	79.1	unclass. *Flavobacteriales*	unclass. *Flavobacteriales*	*Flavobacteriales*	*Flavobacteria*	*Bacteroidetes*	*Bacteria*
Otu0115	0	1.02	0.53	0.49	0.57	79.67	*Glaciecola*	*Alteromonadaceae*	*Alteromonadales*	*Gammaproteobacteria*	*Proteobacteria*	*Bacteria*
Otu0006	0.88	0	0.5	0.73	0.54	80.21	*Polaribacter*	*Flavobacteriaceae*	*Flavobacteriales*	*Flavobacteria*	*Bacteroidetes*	*Bacteria*
Otu0110	0	0.96	0.5	0.49	0.54	80.74	unclass. *Rhodobacteraceae*	*Rhodobacteraceae*	*Rhodobacterales*	*Alphaproteobacteria*	*Proteobacteria*	*Bacteria*
Otu0055	1.08	0.91	0.48	1.31	0.52	81.26	*Colwellia*	*Colwelliaceae*	*Alteromonadales*	*Gammaproteobacteria*	*Proteobacteria*	*Bacteria*
Otu0136	0.68	0	0.41	0.78	0.44	81.7	unclass. *Bacteroidetes*	unclass. *Bacteroidetes*	unclass. *Bacteroidetes*	unclass. *Bacteroidetes*	*Bacteroidetes*	*Bacteria*

OTU	Av.A$_i$ (no)	Av.A$_i$ (serial)	Av.δ$_i$	Av.δ$_i$/SD	Av.δ$_i$ %	∑Av.δ$_i$ %	Genus	Family	Order	Class	Phylum	Domain
Otu0114	0	0.75	0.41	0.8	0.44	82.14	*Shewanella*	*Shewanellaceae*	*Alteromonadales*	*Gammaproteobacteria*	*Proteobacteria*	*Bacteria*
Otu0039	0	0.68	0.39	1.15	0.42	82.56	*Pseudoalteromonas*	*Pseudoalteromonadaceae*	*Alteromonadales*	*Gammaproteobacteria*	*Proteobacteria*	*Bacteria*
Otu0009	0.63	0	0.37	0.79	0.4	82.96	unclass. *Rhodobacteraceae*	*Rhodobacteraceae*	*Rhodobacterales*	*Alphaproteobacteria*	*Proteobacteria*	*Bacteria*
Otu0175	0.6	0	0.35	1.19	0.38	83.34	unclass. *Flavobacteriaceae*	*Flavobacteriaceae*	*Flavobacteriales*	*Flavobacteria*	*Bacteroidetes*	*Bacteria*
Otu0151	0.6	0	0.35	1.19	0.38	83.72	*Rhodococcus*	*Nocardiaceae*	*Actinomycetales*	*Actinobacteria*	*Actinobacteria*	*Bacteria*
Otu0160	0.57	0	0.34	0.8	0.36	84.08	*Colwellia*	*Colwelliaceae*	*Alteromonadales*	*Gammaproteobacteria*	*Proteobacteria*	*Bacteria*
Otu0032	0.55	0	0.32	0.74	0.35	84.43	unclass. *Gammaproteobacteria*	unclass. *Gammaproteobacteria*	unclass. *Gammaproteobacteria*	*Gammaproteobacteria*	*Proteobacteria*	*Bacteria*
Otu0176	0.2	0.48	0.32	0.88	0.34	84.77	*Colwellia*	*Colwelliaceae*	*Alteromonadales*	*Gammaproteobacteria*	*Proteobacteria*	*Bacteria*
Otu0120	0	0.48	0.3	0.77	0.32	85.09	*Pseudoalteromonas*	*Pseudoalteromonadaceae*	*Alteromonadales*	*Gammaproteobacteria*	*Proteobacteria*	*Bacteria*
Otu0011	0.48	0	0.29	0.78	0.31	85.4	unclass. *Flavobacteriaceae*	*Flavobacteriaceae*	*Flavobacteriales*	*Flavobacteria*	*Bacteroidetes*	*Bacteria*
Otu0118	0.4	0.4	0.29	0.94	0.31	85.71	*Colwellia*	*Colwelliaceae*	*Alteromonadales*	*Gammaproteobacteria*	*Proteobacteria*	*Bacteria*
Otu0019	0.48	0	0.28	0.78	0.3	86.01	unclass. *Gammaproteobacteria*	unclass. *Gammaproteobacteria*	unclass. *Gammaproteobacteria*	*Gammaproteobacteria*	*Proteobacteria*	*Bacteria*
Otu0068	0.48	0	0.28	0.78	0.3	86.3	*Pelagibacter*	SAR11-clade	*Rickettsiales*	*Alphaproteobacteria*	*Proteobacteria*	*Bacteria*
Otu0112	0	0.48	0.26	0.79	0.28	86.59	*Polaribacter*	*Flavobacteriaceae*	*Flavobacteriales*	*Flavobacteria*	*Bacteroidetes*	*Bacteria*
Otu0227	0.4	0	0.24	0.8	0.26	86.85	unclass. *Gammaproteobacteria*	unclass. *Gammaproteobacteria*	unclass. *Gammaproteobacteria*	*Gammaproteobacteria*	*Proteobacteria*	*Bacteria*
Otu0133	0.4	0	0.24	0.8	0.26	87.11	*Colwellia*	*Colwelliaceae*	*Alteromonadales*	*Gammaproteobacteria*	*Proteobacteria*	*Bacteria*
Otu0141	0.4	0	0.24	0.8	0.26	87.36	unclass. *Bacteroidetes*	unclass. *Bacteroidetes*	unclass. *Bacteroidetes*	unclass. *Bacteroidetes*	*Bacteroidetes*	*Bacteria*
Otu0159	0.4	0	0.24	0.8	0.26	87.62	unclass. *Saprospiraceae*	*Saprospiraceae*	*Sphingobacteriales*	*Sphingobacteria*	*Bacteroidetes*	*Bacteria*
Otu0042	0.4	0	0.24	0.8	0.26	87.88	*Rhodococcus*	*Nocardiaceae*	*Actinomycetales*	*Actinobacteria*	*Actinobacteria*	*Bacteria*
Otu0157	0.4	0	0.24	0.8	0.26	88.14	unclass. *Gammaproteobacteria*	unclass. *Gammaproteobacteria*	unclass. *Gammaproteobacteria*	*Gammaproteobacteria*	*Proteobacteria*	*Bacteria*
Otu0164	0.4	0	0.24	0.8	0.26	88.39	*Croceibacter*	*Flavobacteriaceae*	*Flavobacteriales*	*Flavobacteria*	*Bacteroidetes*	*Bacteria*
Otu0147	0.4	0	0.24	0.8	0.26	88.65	*Dasania*	*Pseudomonadales_incertae_sedis*	*Pseudomonadales*	*Gammaproteobacteria*	*Proteobacteria*	*Bacteria*
Otu0140	0	0.4	0.23	0.8	0.25	88.9	*Oleispira*	*Oceanospirillaceae*	*Oceanospirillales*	*Gammaproteobacteria*	*Proteobacteria*	*Bacteria*
Otu0051	0.4	0	0.23	0.8	0.25	89.15	unclass. *Flavobacteriaceae*	*Flavobacteriaceae*	*Flavobacteriales*	*Flavobacteria*	*Bacteroidetes*	*Bacteria*
Otu0124	0	0.4	0.21	0.49	0.22	89.37	*Loktanella*	*Rhodobacteraceae*	*Rhodobacterales*	*Alphaproteobacteria*	*Proteobacteria*	*Bacteria*
Otu0003	0.28	0	0.17	0.49	0.18	89.56	unclass. *Rhodobacteraceae*	*Rhodobacteraceae*	*Rhodobacterales*	*Alphaproteobacteria*	*Proteobacteria*	*Bacteria*
Otu0143	0.28	0	0.17	0.49	0.18	89.74	unclass. *Gammaproteobacteria*	unclass. *Gammaproteobacteria*	unclass. *Gammaproteobacteria*	*Gammaproteobacteria*	*Proteobacteria*	*Bacteria*

OTU	Av.A_i (no)	Av.A_i (serial)	Av.δ_i	Av.δ_i/SD	Av.δ_i %	∑Av.δ_i %	Genus	Family	Order	Class	Phylum	Domain
Otu0339	0.28	0	0.17	0.49	0.18	89.93	unclass. *Proteobacteria*	unclass. *Proteobacteria*	unclass. *Proteobacteria*	unclass. *Proteobacteria*	*Proteobacteria*	*Bacteria*
Otu0137	0.28	0	0.17	0.49	0.18	90.11	unclass. *Bacteroidetes*	unclass. *Bacteroidetes*	unclass. *Bacteroidetes*	unclass. *Bacteroidetes*	*Bacteroidetes*	*Bacteria*
Summer pH *in situ* 'no dilution' vs. 'serial dilution' (average dissimilarity: 97.4%)												
Otu0005	15.27	0.2	7.06	6.15	7.25	7.25	*Pelagibacter*	SAR11-clade	*Rickettsiales*	*Alphaproteobacteria*	*Proteobacteria*	*Bacteria*
Otu0132	0	11.11	5.44	1.45	5.59	12.83	unclass. *Alteromonadales*	unclass. *Alteromonadales*	*Alteromonadales*	*Gammaproteobacteria*	*Proteobacteria*	*Bacteria*
Otu0459	0.2	10.06	4.44	1.97	4.56	17.39	*Croceibacter*	*Flavobacteriaceae*	*Flavobacteriales*	*Flavobacteria*	*Bacteroidetes*	*Bacteria*
Otu0471	0	6.52	3.06	2.58	3.14	20.53	*Oceaniserpentilla*	*Oceanospirillaceae*	*Oceanospirillales*	*Gammaproteobacteria*	*Proteobacteria*	*Bacteria*
Otu0691	5.44	0	2.52	3.59	2.59	23.12	*Planctomyces*	*Planctomycetaceae*	*Planctomycetales*	*Planctomycetacia*	*Planctomycetes*	*Bacteria*
Otu0001	4.58	0	2.14	4.15	2.2	25.32	unclass. *Flavobacteriaceae*	*Flavobacteriaceae*	*Flavobacteriales*	*Flavobacteria*	*Bacteroidetes*	*Bacteria*
Otu0125	0	4.15	1.86	1.61	1.91	27.23	*Leeuwenhoekiella*	*Flavobacteriaceae*	*Flavobacteriales*	*Flavobacteria*	*Bacteroidetes*	*Bacteria*
Otu0521	1.08	4.58	1.74	1.56	1.78	29.01	unclass. *Rhodobacteraceae*	*Rhodobacteraceae*	*Rhodobacterales*	*Alphaproteobacteria*	*Proteobacteria*	*Bacteria*
Otu0693	3.22	0	1.51	2.16	1.55	30.56	*Planctomyces*	*Planctomycetaceae*	*Planctomycetales*	*Planctomycetacia*	*Planctomycetes*	*Bacteria*
Otu0003	3.21	0	1.5	6.09	1.54	32.1	unclass. *Rhodobacteraceae*	*Rhodobacteraceae*	*Rhodobacterales*	*Alphaproteobacteria*	*Proteobacteria*	*Bacteria*
Otu0203	3.14	0	1.47	2.64	1.51	33.61	*Pelagibacter*	SAR11-clade	*Rickettsiales*	*Alphaproteobacteria*	*Proteobacteria*	*Bacteria*
Otu0460	0.28	3.42	1.42	1.1	1.46	35.07	*Arcobacter*	*Campylobacteraceae*	*Campylobacterales*	*Epsilonproteobacteria*	*Proteobacteria*	*Bacteria*
Otu0052	2.91	0	1.36	4.3	1.4	36.47	unclass. *Betaproteobacteria*	unclass. *Betaproteobacteria*	unclass. *Betaproteobacteria*	*Betaproteobacteria*	*Proteobacteria*	*Bacteria*
Otu0525	0	2.2	1.02	2.08	1.05	37.52	*Reinekea*	*Oceanospirillaceae*	*Oceanospirillales*	*Gammaproteobacteria*	*Proteobacteria*	*Bacteria*
Otu0692	2.21	0	1.02	2.53	1.05	38.57	unclass. *Bacteria*	unclass. *Bacteria*	unclass. *Bacteria*	unclass. *Bacteria*	unclass. *Bacteria*	*Bacteria*
Otu0523	0	2.3	1.02	1.57	1.04	39.61	*Pseudidiomarina*	*Idiomarinaceae*	*Alteromonadales*	*Gammaproteobacteria*	*Proteobacteria*	*Bacteria*
Otu0007	2.06	0	0.96	4.03	0.99	40.6	unclass. *Flavobacteriales*	unclass. *Flavobacteriales*	*Flavobacteriales*	*Flavobacteria*	*Bacteroidetes*	*Bacteria*
Otu0706	1.99	0	0.93	3.93	0.95	41.55	unclass. *Planctomycetaceae*	*Planctomycetaceae*	*Planctomycetales*	*Planctomycetacia*	*Planctomycetes*	*Bacteria*
Otu0524	0	1.85	0.83	1.82	0.85	42.4	*Pseudidiomarina*	*Idiomarinaceae*	*Alteromonadales*	*Gammaproteobacteria*	*Proteobacteria*	*Bacteria*
Otu0461	1.89	0.2	0.81	1.42	0.83	43.22	unclass. *Microbacteriaceae*	*Microbacteriaceae*	*Actinomycetales*	*Actinobacteria*	*Actinobacteria*	*Bacteria*
Otu0519	1.72	0	0.8	1.92	0.82	44.05	unclass. *Rhodospirillaceae*	*Rhodospirillaceae*	*Rhodospirillales*	*Alphaproteobacteria*	*Proteobacteria*	*Bacteria*
Otu0450	1.66	0	0.79	1.16	0.81	44.86	*Sphingobium*	*Sphingomonadaceae*	*Sphingomonadales*	*Alphaproteobacteria*	*Proteobacteria*	*Bacteria*
Otu0522	0	1.75	0.78	1.8	0.8	45.65	unclass. *Flavobacteriaceae*	*Flavobacteriaceae*	*Flavobacteriales*	*Flavobacteria*	*Bacteroidetes*	*Bacteria*
Otu0531	0	1.7	0.76	1.56	0.78	46.43	unclass. *Flavobacteriaceae*	*Flavobacteriaceae*	*Flavobacteriales*	*Flavobacteria*	*Bacteroidetes*	*Bacteria*

OTU	Av.A_i (no)	Av.A_i (serial)	Av.δ_i	Av.δ_i/SD	Av.δ_i %	∑Av.δ_i %	Genus	Family	Order	Class	Phylum	Domain
Otu0512	1.58	0	0.75	1.41	0.77	47.2	*Rhodopirellula*	*Planctomycetaceae*	*Planctomycetales*	*Planctomycetacia*	*Planctomycetes*	*Bacteria*
Otu0473	1.63	0	0.74	2.45	0.76	47.96	unclass. *Alphaproteobacteria*	unclass. *Alphaproteobacteria*	unclass. *Alphaproteobacteria*	*Alphaproteobacteria*	*Proteobacteria*	*Bacteria*
Otu0495	1.53	0	0.7	1.69	0.72	48.68	unclass. *Flavobacteriales*	unclass. *Flavobacteriales*	*Flavobacteriales*	*Flavobacteria*	*Bacteroidetes*	*Bacteria*
Otu0469	1.52	0	0.7	1.28	0.72	49.4	unclass. *Bacteria*	unclass. *Bacteria*	unclass. *Bacteria*	unclass. *Bacteria*	unclass. *Bacteria*	*Bacteria*
Otu0068	1.51	0	0.7	4.41	0.72	50.12	*Pelagibacter*	SAR11-clade	*Rickettsiales*	*Alphaproteobacteria*	*Proteobacteria*	*Bacteria*
Otu0463	1.43	0	0.67	3.25	0.68	50.81	unclass. *Flavobacteriaceae*	*Flavobacteriaceae*	*Flavobacteriales*	*Flavobacteria*	*Bacteroidetes*	*Bacteria*
Otu0157	1.37	0	0.65	1.59	0.67	51.48	unclass. *Gammaproteobacteria*	unclass. *Gammaproteobacteria*	unclass. *Gammaproteobacteria*	*Gammaproteobacteria*	*Proteobacteria*	*Bacteria*
Otu0019	1.33	0	0.63	1.79	0.65	52.12	unclass. *Gammaproteobacteria*	unclass. *Gammaproteobacteria*	unclass. *Gammaproteobacteria*	*Gammaproteobacteria*	*Proteobacteria*	*Bacteria*
Otu0526	0	1.39	0.62	1.02	0.64	52.76	*Maribacter*	*Flavobacteriaceae*	*Flavobacteriales*	*Flavobacteria*	*Bacteroidetes*	*Bacteria*
Otu0537	0	1.38	0.62	1.75	0.64	53.4	unclass. *Flavobacteriaceae*	*Flavobacteriaceae*	*Flavobacteriales*	*Flavobacteria*	*Bacteroidetes*	*Bacteria*
Otu0046	1.29	0	0.62	1.72	0.63	54.03	unclass. *Flavobacteriaceae*	*Flavobacteriaceae*	*Flavobacteriales*	*Flavobacteria*	*Bacteroidetes*	*Bacteria*
Otu0487	0	1.38	0.61	0.79	0.63	54.66	unclass. *Chitinophagaceae*	*Chitinophagaceae*	*Sphingobacteriales*	*Sphingobacteria*	*Bacteroidetes*	*Bacteria*
Otu0053	1.29	0	0.58	1.53	0.6	55.26	unclass. *Rhodobacteraceae*	*Rhodobacteraceae*	*Rhodobacterales*	*Alphaproteobacteria*	*Proteobacteria*	*Bacteria*
Otu0705	1.19	0	0.55	1.18	0.57	55.83	*Planctomyces*	*Planctomycetaceae*	*Planctomycetales*	*Planctomycetacia*	*Planctomycetes*	*Bacteria*
Otu0695	1.12	0	0.53	0.8	0.55	56.37	unclass. *Proteobacteria*	unclass. *Proteobacteria*	unclass. *Proteobacteria*	unclass. *Proteobacteria*	*Proteobacteria*	*Bacteria*
Otu0549	0	1.26	0.53	0.49	0.54	56.92	unclass. *Bacteroidetes*	unclass. *Bacteroidetes*	unclass. *Bacteroidetes*	unclass. *Bacteroidetes*	*Bacteroidetes*	*Bacteria*
Otu0123	0	1.08	0.53	1.18	0.54	57.46	unclass. *Gammaproteobacteria*	unclass. *Gammaproteobacteria*	unclass. *Gammaproteobacteria*	*Gammaproteobacteria*	*Proteobacteria*	*Bacteria*
Otu0503	1.15	0	0.52	1.65	0.53	57.99	unclass. *Bacteroidetes*	unclass. *Bacteroidetes*	unclass. *Bacteroidetes*	unclass. *Bacteroidetes*	*Bacteroidetes*	*Bacteria*
Otu0354	1.11	0	0.51	1.76	0.53	58.52	*Anderseniella*	*Rhodobiaceae*	*Rhizobiales*	*Alphaproteobacteria*	*Proteobacteria*	*Bacteria*
Otu0435	1.12	0	0.51	0.6	0.53	59.05	unclass. *Flavobacteriales*	unclass. *Flavobacteriales*	*Flavobacteriales*	*Flavobacteria*	*Bacteroidetes*	*Bacteria*
Otu0289	1.08	0	0.5	0.99	0.52	59.56	unclass. *Bacteroidetes*	unclass. *Bacteroidetes*	unclass. *Bacteroidetes*	unclass. *Bacteroidetes*	*Bacteroidetes*	*Bacteria*
Otu0027	0.2	1.11	0.5	0.78	0.51	60.07	*Pseudoalteromonas*	*Pseudoalteromonadaceae*	*Alteromonadales*	*Gammaproteobacteria*	*Proteobacteria*	*Bacteria*
Otu0041	0.75	0.88	0.48	1.24	0.49	60.56	*Sulfitobacter*	*Rhodobacteraceae*	*Rhodobacterales*	*Alphaproteobacteria*	*Proteobacteria*	*Bacteria*
Otu0703	1.03	0	0.48	1.67	0.49	61.05	unclass. *Gammaproteobacteria*	unclass. *Gammaproteobacteria*	unclass. *Gammaproteobacteria*	*Gammaproteobacteria*	*Proteobacteria*	*Bacteria*
Otu0502	1.03	0	0.48	1.76	0.49	61.54	unclass. *Gammaproteobacteria*	unclass. *Gammaproteobacteria*	unclass. *Gammaproteobacteria*	*Gammaproteobacteria*	*Proteobacteria*	*Bacteria*
Otu0186	0.97	0	0.46	1.79	0.47	62.01	unclass. *Planctomycetaceae*	*Planctomycetaceae*	*Planctomycetales*	*Planctomycetacia*	*Planctomycetes*	*Bacteria*
Otu0704	0.97	0	0.46	1.82	0.47	62.49	unclass. *Proteobacteria*	unclass. *Proteobacteria*	unclass. *Proteobacteria*	unclass. *Proteobacteria*	*Proteobacteria*	*Bacteria*

OTU	Av.A_i (no)	Av.A_i (serial)	Av.δ_i	Av.δ_i/SD	Av.δ_i %	∑Av.δ_i %	Genus	Family	Order	Class	Phylum	Domain
Otu0753	0.95	0	0.45	0.64	0.46	62.95	unclass. *Flavobacteriales*	unclass. *Flavobacteriales*	*Flavobacteriales*	*Flavobacteria*	*Bacteroidetes*	*Bacteria*
Otu0245	0.97	0	0.45	1.77	0.46	63.4	unclass. *Flavobacteriales*	unclass. *Flavobacteriales*	*Flavobacteriales*	*Flavobacteria*	*Bacteroidetes*	*Bacteria*
Otu0084	0.93	0	0.43	1.02	0.45	63.85	unclass. *Gammaproteobacteria*	unclass. *Gammaproteobacteria*	unclass. *Gammaproteobacteria*	*Gammaproteobacteria*	*Proteobacteria*	*Bacteria*
Otu0779	0.95	0	0.43	0.69	0.44	64.29	unclass. *Ectothiorhodospiraceae*	*Ectothiorhodospiraceae*	*Chromatiales*	*Gammaproteobacteria*	*Proteobacteria*	*Bacteria*
Otu0029	0	1	0.42	0.49	0.43	64.72	unclass. *Bacteroidetes*	unclass. *Bacteroidetes*	unclass. *Bacteroidetes*	unclass. *Bacteroidetes*	*Bacteroidetes*	*Bacteria*
Otu0701	0.88	0	0.41	1.05	0.42	65.14	unclass. *Bacteria*	unclass. *Bacteria*	unclass. *Bacteria*	unclass. *Bacteria*	unclass. *Bacteria*	*Bacteria*
Otu0548	0	0.98	0.41	0.49	0.42	65.57	unclass. *Bacteroidetes*	unclass. *Bacteroidetes*	unclass. *Bacteroidetes*	unclass. *Bacteroidetes*	*Bacteroidetes*	*Bacteria*
Otu0010	0.88	0	0.41	1.74	0.42	65.99	unclass. *Flavobacteriaceae*	*Flavobacteriaceae*	*Flavobacteriales*	*Flavobacteria*	*Bacteroidetes*	*Bacteria*
Otu0109	0	0.88	0.38	1.09	0.39	66.38	*Alteromonas*	*Alteromonadaceae*	*Alteromonadales*	*Gammaproteobacteria*	*Proteobacteria*	*Bacteria*
Otu0527	0	0.83	0.38	1.13	0.39	66.77	*Idiomarina*	*Idiomarinaceae*	*Alteromonadales*	*Gammaproteobacteria*	*Proteobacteria*	*Bacteria*
Otu0532	0.77	0.88	0.37	1.18	0.38	67.15	unclass. *Rhodobacteraceae*	*Rhodobacteraceae*	*Rhodobacterales*	*Alphaproteobacteria*	*Proteobacteria*	*Bacteria*
Otu0611	0.72	0	0.35	0.49	0.36	67.51	unclass. *Bacteroidetes*	unclass. *Bacteroidetes*	unclass. *Bacteroidetes*	unclass. *Bacteroidetes*	*Bacteroidetes*	*Bacteria*
Otu0694	0.75	0	0.35	1.05	0.36	67.87	unclass. *Alphaproteobacteria*	unclass. *Alphaproteobacteria*	unclass. *Alphaproteobacteria*	*Alphaproteobacteria*	*Proteobacteria*	*Bacteria*
Otu0408	0.75	0	0.34	1.13	0.35	68.21	unclass. *Flavobacteriaceae*	*Flavobacteriaceae*	*Flavobacteriales*	*Flavobacteria*	*Bacteroidetes*	*Bacteria*
Otu0714	0.68	0	0.33	1.14	0.34	68.55	unclass. *Alphaproteobacteria*	unclass. *Alphaproteobacteria*	unclass. *Alphaproteobacteria*	*Alphaproteobacteria*	*Proteobacteria*	*Bacteria*
Otu0504	0.68	0	0.33	1.14	0.34	68.89	unclass. *Alphaproteobacteria*	unclass. *Alphaproteobacteria*	unclass. *Alphaproteobacteria*	*Alphaproteobacteria*	*Proteobacteria*	*Bacteria*
Otu0142	0.68	0	0.33	1.15	0.34	69.23	unclass. *Gammaproteobacteria*	unclass. *Gammaproteobacteria*	unclass. *Gammaproteobacteria*	*Gammaproteobacteria*	*Proteobacteria*	*Bacteria*
Otu0696	0.68	0	0.33	1.13	0.33	69.56	unclass. *Flavobacteriaceae*	*Flavobacteriaceae*	*Flavobacteriales*	*Flavobacteria*	*Bacteroidetes*	*Bacteria*
Otu0261	0.77	0.4	0.32	1.17	0.33	69.89	*Winogradskyella*	*Flavobacteriaceae*	*Flavobacteriales*	*Flavobacteria*	*Bacteroidetes*	*Bacteria*
Otu0700	0.68	0	0.31	1.17	0.32	70.21	unclass. *Alphaproteobacteria*	unclass. *Alphaproteobacteria*	unclass. *Alphaproteobacteria*	*Alphaproteobacteria*	*Proteobacteria*	*Bacteria*
Otu0782	0.68	0	0.31	1.13	0.32	70.52	unclass. *Gammaproteobacteria*	unclass. *Gammaproteobacteria*	unclass. *Gammaproteobacteria*	*Gammaproteobacteria*	*Proteobacteria*	*Bacteria*
Otu0048	0.63	0	0.3	0.79	0.31	70.83	unclass. *Flavobacteriaceae*	*Flavobacteriaceae*	*Flavobacteriales*	*Flavobacteria*	*Bacteroidetes*	*Bacteria*
Otu0781	0.63	0	0.3	0.49	0.3	71.14	unclass. *Thiotrichales*	unclass. *Thiotrichales*	*Thiotrichales*	*Gammaproteobacteria*	*Proteobacteria*	*Bacteria*
Otu0697	0.6	0	0.29	1.18	0.3	71.43	unclass. *Flavobacteriaceae*	*Flavobacteriaceae*	*Flavobacteriales*	*Flavobacteria*	*Bacteroidetes*	*Bacteria*
Otu0020	0.63	0	0.28	0.79	0.29	71.72	unclass. *Flammeovirgaceae*	*Flammeovirgaceae*	*Sphingobacteriales*	*Sphingobacteria*	*Bacteroidetes*	*Bacteria*
Otu0550	0.28	0.49	0.28	0.68	0.29	72.01	unclass. *Flavobacteriales*	unclass. *Flavobacteriales*	*Flavobacteriales*	*Flavobacteria*	*Bacteroidetes*	*Bacteria*
Otu0505	0.63	0	0.28	0.79	0.29	72.3	unclass. *Chitinophagaceae*	*Chitinophagaceae*	*Sphingobacteriales*	*Sphingobacteria*	*Bacteroidetes*	*Bacteria*

OTU	$Av.A_i$ (no)	$Av.A_i$ (serial)	$Av.\delta_i$	$Av.\delta_i/SD$	$Av.\delta_i$ %	$\sum Av.\delta_i$ %	Genus	Family	Order	Class	Phylum	Domain
Otu0737	0.63	0	0.28	0.79	0.29	72.58	unclass. *Gammaproteobacteria*	unclass. *Gammaproteobacteria*	unclass. *Gammaproteobacteria*	*Gammaproteobacteria*	*Proteobacteria*	*Bacteria*
Otu0110	0	0.55	0.28	0.72	0.29	72.87	unclass. *Rhodobacteraceae*	*Rhodobacteraceae*	*Rhodobacterales*	*Alphaproteobacteria*	*Proteobacteria*	*Bacteria*
Otu0476	0.6	0	0.28	1.18	0.28	73.15	unclass. *Actinomycetales*	unclass. *Actinomycetales*	*Actinomycetales*	*Actinobacteria*	*Actinobacteria*	*Bacteria*
Otu0272	0.6	0	0.28	1.18	0.28	73.43	unclass. *Flavobacteriaceae*	*Flavobacteriaceae*	*Flavobacteriales*	*Flavobacteria*	*Bacteroidetes*	*Bacteria*
Otu0368	0.6	0	0.28	1.18	0.28	73.72	unclass. *Saprospiraceae*	*Saprospiraceae*	*Sphingobacteriales*	*Sphingobacteria*	*Bacteroidetes*	*Bacteria*
Otu0191	0.6	0	0.28	1.18	0.28	74	unclass. *Proteobacteria*	unclass. *Proteobacteria*	unclass. *Proteobacteria*	unclass. *Proteobacteria*	*Proteobacteria*	*Bacteria*
Otu0485	0.6	0	0.28	1.18	0.28	74.28	unclass. *Alphaproteobacteria*	unclass. *Alphaproteobacteria*	unclass. *Alphaproteobacteria*	*Alphaproteobacteria*	*Proteobacteria*	*Bacteria*
Otu0468	0.57	0	0.27	0.79	0.28	74.56	unclass. *Flavobacteriaceae*	*Flavobacteriaceae*	*Flavobacteriales*	*Flavobacteria*	*Bacteroidetes*	*Bacteria*
Otu0721	0.6	0	0.27	1.18	0.28	74.84	unclass. *Alphaproteobacteria*	unclass. *Alphaproteobacteria*	unclass. *Alphaproteobacteria*	*Alphaproteobacteria*	*Proteobacteria*	*Bacteria*
Otu0768	0.6	0	0.27	1.18	0.28	75.12	unclass. *Flavobacteriales*	unclass. *Flavobacteriales*	*Flavobacteriales*	*Flavobacteria*	*Bacteroidetes*	*Bacteria*
Otu0533	0	0.55	0.25	0.77	0.26	75.37	*Nisaea*	*Rhodospirillaceae*	*Rhodospirillales*	*Alphaproteobacteria*	*Proteobacteria*	*Bacteria*
Otu0538	0	0.48	0.23	0.79	0.24	75.61	*Oceaniserpentilla*	*Oceanospirillaceae*	*Oceanospirillales*	*Gammaproteobacteria*	*Proteobacteria*	*Bacteria*
Otu0080	0.48	0	0.23	0.77	0.24	75.85	*Polaribacter*	*Flavobacteriaceae*	*Flavobacteriales*	*Flavobacteria*	*Bacteroidetes*	*Bacteria*
Otu0497	0.48	0	0.23	0.77	0.24	76.09	*Opitutus*	*Opitutaceae*	*Opitutales*	*Opitutae*	*Verrucomicrobia*	*Bacteria*
Otu0754	0.48	0	0.23	0.77	0.24	76.33	unclass. *Flavobacteriales*	unclass. *Flavobacteriales*	*Flavobacteriales*	*Flavobacteria*	*Bacteroidetes*	*Bacteria*
Otu0710	0.48	0	0.23	0.78	0.23	76.56	unclass. *Gammaproteobacteria*	unclass. *Gammaproteobacteria*	unclass. *Gammaproteobacteria*	*Gammaproteobacteria*	*Proteobacteria*	*Bacteria*
Otu0218	0.48	0	0.22	0.79	0.22	76.78	unclass. *Flavobacteriaceae*	*Flavobacteriaceae*	*Flavobacteriales*	*Flavobacteria*	*Bacteroidetes*	*Bacteria*
Otu0758	0.48	0	0.22	0.79	0.22	77	unclass. *Bacteria*	unclass. *Bacteria*	unclass. *Bacteria*	unclass. *Bacteria*	unclass. *Bacteria*	*Bacteria*
Otu0221	0.48	0	0.22	0.76	0.22	77.22	unclass. *Flavobacteriaceae*	*Flavobacteriaceae*	*Flavobacteriales*	*Flavobacteria*	*Bacteroidetes*	*Bacteria*
Otu0798	0.48	0	0.22	0.76	0.22	77.45	unclass. *Gammaproteobacteria*	unclass. *Gammaproteobacteria*	unclass. *Gammaproteobacteria*	*Gammaproteobacteria*	*Proteobacteria*	*Bacteria*
Otu0732	0.48	0	0.22	0.76	0.22	77.67	unclass. *Proteobacteria*	unclass. *Proteobacteria*	unclass. *Proteobacteria*	unclass. *Proteobacteria*	*Proteobacteria*	*Bacteria*
Otu0180	0	0.48	0.21	0.78	0.22	77.89	*Reichenbachiella*	*Flammeovirgaceae*	*Sphingobacteriales*	*Sphingobacteria*	*Bacteroidetes*	*Bacteria*
Otu0529	0	0.48	0.21	0.78	0.22	78.11	unclass. *Sphingobacteriales*	unclass. *Sphingobacteriales*	*Sphingobacteriales*	*Sphingobacteria*	*Bacteroidetes*	*Bacteria*
Otu0535	0	0.48	0.21	0.78	0.22	78.32	*Loktanella*	*Rhodobacteraceae*	*Rhodobacterales*	*Alphaproteobacteria*	*Proteobacteria*	*Bacteria*
Otu0840	0.49	0	0.21	0.49	0.21	78.54	unclass. *Flavobacteriaceae*	*Flavobacteriaceae*	*Flavobacteriales*	*Flavobacteria*	*Bacteroidetes*	*Bacteria*
Otu0038	0.4	0	0.2	0.79	0.2	78.74	unclass. *Proteobacteria*	unclass. *Proteobacteria*	unclass. *Proteobacteria*	unclass. *Proteobacteria*	*Proteobacteria*	*Bacteria*
Otu0216	0.4	0	0.2	0.79	0.2	78.94	*Pelagibacter*	SAR11-clade	*Rickettsiales*	*Alphaproteobacteria*	*Proteobacteria*	*Bacteria*

OTU	Av.A$_i$ (no)	Av.A$_i$ (serial)	Av.δ$_i$	Av.δ$_i$/SD	Av.δ$_i$ %	∑Av.δ$_i$ %	Genus	Family	Order	Class	Phylum	Domain
Otu0735	0.4	0	0.19	0.79	0.2	79.13	unclass. *Gammaproteobacteria*	unclass. *Gammaproteobacteria*	unclass. *Gammaproteobacteria*	*Gammaproteobacteria*	*Proteobacteria*	*Bacteria*
Otu0745	0.4	0	0.19	0.79	0.2	79.33	*Planctomyces*	*Planctomycetaceae*	*Planctomycetales*	*Planctomycetacia*	*Planctomycetes*	*Bacteria*
Otu0002	0.4	0	0.19	0.79	0.2	79.53	unclass. *Flavobacteriaceae*	*Flavobacteriaceae*	*Flavobacteriales*	*Flavobacteria*	*Bacteroidetes*	*Bacteria*
Otu0277	0.4	0	0.19	0.79	0.2	79.72	unclass. *Flavobacteriaceae*	*Flavobacteriaceae*	*Flavobacteriales*	*Flavobacteria*	*Bacteroidetes*	*Bacteria*
Otu0724	0.4	0	0.19	0.79	0.2	79.92	unclass. *Bacteria*	unclass. *Bacteria*	unclass. *Bacteria*	unclass. *Bacteria*	unclass. *Bacteria*	*Bacteria*
Otu0746	0.4	0	0.19	0.79	0.2	80.12	unclass. *Flavobacteriaceae*	*Flavobacteriaceae*	*Flavobacteriales*	*Flavobacteria*	*Bacteroidetes*	*Bacteria*
Otu0202	0.4	0	0.19	0.79	0.2	80.31	unclass. *Alteromonadaceae*	*Alteromonadaceae*	*Alteromonadales*	*Gammaproteobacteria*	*Proteobacteria*	*Bacteria*
Otu0051	0.4	0	0.19	0.79	0.2	80.51	unclass. *Flavobacteriaceae*	*Flavobacteriaceae*	*Flavobacteriales*	*Flavobacteria*	*Bacteroidetes*	*Bacteria*
Otu0352	0.4	0	0.19	0.79	0.2	80.71	unclass. *Flavobacteriaceae*	*Flavobacteriaceae*	*Flavobacteriales*	*Flavobacteria*	*Bacteroidetes*	*Bacteria*
Otu0595	0.4	0	0.19	0.79	0.2	80.9	unclass. *Legionellaceae*	*Legionellaceae*	*Legionellales*	*Gammaproteobacteria*	*Proteobacteria*	*Bacteria*
Otu0783	0.4	0	0.19	0.49	0.19	81.09	unclass. *Gammaproteobacteria*	unclass. *Gammaproteobacteria*	unclass. *Gammaproteobacteria*	*Gammaproteobacteria*	*Proteobacteria*	*Bacteria*
Otu0534	0	0.4	0.19	0.8	0.19	81.28	unclass. *Alphaproteobacteria*	unclass. *Alphaproteobacteria*	unclass. *Alphaproteobacteria*	*Alphaproteobacteria*	*Proteobacteria*	*Bacteria*
Otu0727	0.4	0	0.18	0.79	0.19	81.47	unclass. *Deltaproteobacteria*	unclass. *Deltaproteobacteria*	unclass. *Deltaproteobacteria*	*Deltaproteobacteria*	*Proteobacteria*	*Bacteria*
Otu0472	0.4	0	0.18	0.79	0.19	81.66	unclass. *Bacteroidetes*	unclass. *Bacteroidetes*	unclass. *Bacteroidetes*	unclass. *Bacteroidetes*	*Bacteroidetes*	*Bacteria*
Otu0490	0.4	0	0.18	0.79	0.19	81.84	unclass. *Burkholderiales*	unclass. *Burkholderiales*	*Burkholderiales*	*Betaproteobacteria*	*Proteobacteria*	*Bacteria*
Otu0766	0.4	0	0.18	0.79	0.19	82.03	*Lentisphaera*	*Lentisphaeraceae*	*Lentisphaerales*	*Lentisphaeria*	*Lentisphaerae*	*Bacteria*
Otu0774	0.4	0	0.18	0.79	0.19	82.22	unclass. *Bacteria*	unclass. *Bacteria*	unclass. *Bacteria*	unclass. *Bacteria*	unclass. *Bacteria*	*Bacteria*
Otu0541	0.4	0	0.18	0.79	0.18	82.4	unclass. *Rhodobacteraceae*	*Rhodobacteraceae*	*Rhodobacterales*	*Alphaproteobacteria*	*Proteobacteria*	*Bacteria*
Otu0736	0.4	0	0.18	0.79	0.18	82.58	*Persicirhabdus*	*Verrucomicrobiaceae*	*Verrucomicrobiales*	*Verrucomicrobiae*	*Verrucomicrobia*	*Bacteria*
Otu0811	0.4	0	0.18	0.79	0.18	82.76	unclass. *Bacteria*	unclass. *Bacteria*	unclass. *Bacteria*	unclass. *Bacteria*	unclass. *Bacteria*	*Bacteria*
Otu0255	0.4	0	0.18	0.79	0.18	82.95	*Sphingopyxis*	*Sphingomonadaceae*	*Sphingomonadales*	*Alphaproteobacteria*	*Proteobacteria*	*Bacteria*
Otu0467	0.4	0	0.18	0.79	0.18	83.13	unclass. *Rhodospirillaceae*	*Rhodospirillaceae*	*Rhodospirillales*	*Alphaproteobacteria*	*Proteobacteria*	*Bacteria*
Otu0470	0.4	0	0.18	0.79	0.18	83.31	unclass. *Flavobacteriaceae*	*Flavobacteriaceae*	*Flavobacteriales*	*Flavobacteria*	*Bacteroidetes*	*Bacteria*
Otu0707	0.4	0	0.18	0.79	0.18	83.49	*Haliea*	*Alteromonadaceae*	*Alteromonadales*	*Gammaproteobacteria*	*Proteobacteria*	*Bacteria*
Otu0787	0.4	0	0.18	0.79	0.18	83.67	unclass. *Verrucomicrobia*	unclass. *Verrucomicrobia*	unclass. *Verrucomicrobia*	unclass. *Verrucomicrobia*	*Verrucomicrobia*	*Bacteria*
Otu0819	0.4	0	0.18	0.79	0.18	83.86	unclass. *Bacteria*	unclass. *Bacteria*	unclass. *Bacteria*	unclass. *Bacteria*	unclass. *Bacteria*	*Bacteria*
Otu0072	0.35	0	0.17	0.49	0.17	84.03	*Sulfitobacter*	*Rhodobacteraceae*	*Rhodobacterales*	*Alphaproteobacteria*	*Proteobacteria*	*Bacteria*

OTU	Av.A_i (no)	Av.A_i (serial)	Av.δ_i	Av.δ_i/SD	Av.δ_i %	∑Av.δ_i %	Genus	Family	Order	Class	Phylum	Domain
Otu0757	0.35	0	0.17	0.49	0.17	84.2	unclass. *Gammaproteobacteria*	unclass. *Gammaproteobacteria*	unclass. *Gammaproteobacteria*	*Gammaproteobacteria*	*Proteobacteria*	*Bacteria*
Otu0789	0.35	0	0.16	0.49	0.17	84.37	unclass. *Proteobacteria*	unclass. *Proteobacteria*	unclass. *Proteobacteria*	unclass. *Proteobacteria*	*Proteobacteria*	*Bacteria*
Otu0031	0.35	0	0.16	0.49	0.17	84.54	unclass. *Gammaproteobacteria*	unclass. *Gammaproteobacteria*	unclass. *Gammaproteobacteria*	*Gammaproteobacteria*	*Proteobacteria*	*Bacteria*
Otu0380	0.35	0	0.16	0.49	0.17	84.7	unclass. *Proteobacteria*	unclass. *Proteobacteria*	unclass. *Proteobacteria*	unclass. *Proteobacteria*	*Proteobacteria*	*Bacteria*
Otu0814	0.35	0	0.16	0.49	0.17	84.87	unclass. *Proteobacteria*	unclass. *Proteobacteria*	unclass. *Proteobacteria*	unclass. *Proteobacteria*	*Proteobacteria*	*Bacteria*
Otu0009	0.2	0.2	0.15	0.67	0.15	85.02	unclass. *Rhodobacteraceae*	*Rhodobacteraceae*	*Rhodobacterales*	*Alphaproteobacteria*	*Proteobacteria*	*Bacteria*
Otu0060	0.35	0	0.15	0.49	0.15	85.17	unclass. *Actinomycetales*	unclass. *Actinomycetales*	*Actinomycetales*	*Actinobacteria*	*Actinobacteria*	*Bacteria*
Otu0630	0.35	0	0.15	0.49	0.15	85.32	unclass. *Flavobacteriales*	unclass. *Flavobacteriales*	*Flavobacteriales*	*Flavobacteria*	*Bacteroidetes*	*Bacteria*
Otu0555	0.2	0.2	0.14	0.67	0.15	85.46	*Maricaulis*	*Hyphomonadaceae*	*Caulobacterales*	*Alphaproteobacteria*	*Proteobacteria*	*Bacteria*
Otu0017	0.28	0	0.14	0.49	0.14	85.61	unclass. *Flavobacteriales*	unclass. *Flavobacteriales*	*Flavobacteriales*	*Flavobacteria*	*Bacteroidetes*	*Bacteria*
Otu0030	0.28	0	0.14	0.49	0.14	85.75	unclass. *Gammaproteobacteria*	unclass. *Gammaproteobacteria*	unclass. *Gammaproteobacteria*	*Gammaproteobacteria*	*Proteobacteria*	*Bacteria*
Otu0759	0.28	0	0.14	0.49	0.14	85.89	unclass. *Verrucomicrobiaceae*	*Verrucomicrobiaceae*	*Verrucomicrobiales*	*Verrucomicrobiae*	*Verrucomicrobia*	*Bacteria*
Otu0075	0.28	0	0.13	0.49	0.14	86.03	unclass. *Alphaproteobacteria*	unclass. *Alphaproteobacteria*	unclass. *Alphaproteobacteria*	*Alphaproteobacteria*	*Proteobacteria*	*Bacteria*
Otu0282	0.28	0	0.13	0.49	0.14	86.16	*Haliea*	*Alteromonadaceae*	*Alteromonadales*	*Gammaproteobacteria*	*Proteobacteria*	*Bacteria*
Otu0743	0.28	0	0.13	0.49	0.14	86.3	unclass. *Verrucomicrobia*	unclass. *Verrucomicrobia*	unclass. *Verrucomicrobia*	unclass. *Verrucomicrobia*	*Verrucomicrobia*	*Bacteria*
Otu0761	0.28	0	0.13	0.49	0.14	86.43	unclass. *Verrucomicrobiaceae*	*Verrucomicrobiaceae*	*Verrucomicrobiales*	*Verrucomicrobiae*	*Verrucomicrobia*	*Bacteria*
Otu0810	0.28	0	0.13	0.49	0.14	86.57	unclass. *Gammaproteobacteria*	unclass. *Gammaproteobacteria*	unclass. *Gammaproteobacteria*	*Gammaproteobacteria*	*Proteobacteria*	*Bacteria*
Otu0822	0.28	0	0.13	0.49	0.14	86.71	unclass. *Proteobacteria*	unclass. *Proteobacteria*	unclass. *Proteobacteria*	unclass. *Proteobacteria*	*Proteobacteria*	*Bacteria*
Otu0827	0.28	0	0.13	0.49	0.14	86.84	unclass. *Bacteria*	unclass. *Bacteria*	unclass. *Bacteria*	unclass. *Bacteria*	unclass. *Bacteria*	*Bacteria*
Otu0036	0	0.28	0.12	0.49	0.13	86.97	*Glaciecola*	*Alteromonadaceae*	*Alteromonadales*	*Gammaproteobacteria*	*Proteobacteria*	*Bacteria*
Otu0772	0.28	0	0.12	0.49	0.12	87.09	unclass. *Alphaproteobacteria*	unclass. *Alphaproteobacteria*	unclass. *Alphaproteobacteria*	*Alphaproteobacteria*	*Proteobacteria*	*Bacteria*
Otu0839	0.28	0	0.12	0.49	0.12	87.21	unclass. *Bacteria*	unclass. *Bacteria*	unclass. *Bacteria*	unclass. *Bacteria*	unclass. *Bacteria*	*Bacteria*
Otu0167	0.2	0	0.1	0.49	0.1	87.31	unclass. *Rhodobacteraceae*	*Rhodobacteraceae*	*Rhodobacterales*	*Alphaproteobacteria*	*Proteobacteria*	*Bacteria*
Otu0213	0.2	0	0.1	0.49	0.1	87.42	unclass. *Gammaproteobacteria*	unclass. *Gammaproteobacteria*	unclass. *Gammaproteobacteria*	*Gammaproteobacteria*	*Proteobacteria*	*Bacteria*
Otu0290	0.2	0	0.1	0.49	0.1	87.52	unclass. *Flavobacteriaceae*	*Flavobacteriaceae*	*Flavobacteriales*	*Flavobacteria*	*Bacteroidetes*	*Bacteria*
Otu0464	0.2	0	0.1	0.49	0.1	87.62	unclass. *Bacteroidetes*	unclass. *Bacteroidetes*	unclass. *Bacteroidetes*	unclass. *Bacteroidetes*	*Bacteroidetes*	*Bacteria*
Otu0482	0.2	0	0.1	0.49	0.1	87.72	unclass. *Microbacteriaceae*	*Microbacteriaceae*	*Actinomycetales*	*Actinobacteria*	*Actinobacteria*	*Bacteria*

OTU	Av.A_i (no)	Av.A_i (serial)	Av.δ_i	Av.δ_i/SD	Av.δ_i %	∑Av.δ_i %	Genus	Family	Order	Class	Phylum	Domain
Otu0594	0.2	0	0.1	0.49	0.1	87.82	unclass. *Bacteria*	unclass. *Bacteria*	unclass. *Bacteria*	unclass. *Bacteria*	unclass. *Bacteria*	*Bacteria*
Otu0666	0.2	0	0.1	0.49	0.1	87.92	unclass. *Deltaproteobacteria*	unclass. *Deltaproteobacteria*	unclass. *Deltaproteobacteria*	*Deltaproteobacteria*	*Proteobacteria*	*Bacteria*
Otu0702	0.2	0	0.1	0.49	0.1	88.02	unclass. *Flavobacteriales*	unclass. *Flavobacteriales*	*Flavobacteriales*	*Flavobacteria*	*Bacteroidetes*	*Bacteria*
Otu0709	0.2	0	0.1	0.49	0.1	88.12	unclass. *Bacteria*	unclass. *Bacteria*	unclass. *Bacteria*	unclass. *Bacteria*	unclass. *Bacteria*	*Bacteria*
Otu0712	0.2	0	0.1	0.49	0.1	88.22	unclass. *Alphaproteobacteria*	unclass. *Alphaproteobacteria*	unclass. *Alphaproteobacteria*	*Alphaproteobacteria*	*Proteobacteria*	*Bacteria*
Otu0719	0.2	0	0.1	0.49	0.1	88.32	unclass. *Flavobacteriales*	unclass. *Flavobacteriales*	*Flavobacteriales*	*Flavobacteria*	*Bacteroidetes*	*Bacteria*
Otu0722	0.2	0	0.1	0.49	0.1	88.42	unclass. *Rhodobacteraceae*	*Rhodobacteraceae*	*Rhodobacterales*	*Alphaproteobacteria*	*Proteobacteria*	*Bacteria*
Otu0723	0.2	0	0.1	0.49	0.1	88.52	unclass. *Microbacteriaceae*	*Microbacteriaceae*	*Actinomycetales*	*Actinobacteria*	*Actinobacteria*	*Bacteria*
Otu0726	0.2	0	0.1	0.49	0.1	88.63	unclass. *Bacteroidetes*	unclass. *Bacteroidetes*	unclass. *Bacteroidetes*	unclass. *Bacteroidetes*	*Bacteroidetes*	*Bacteria*
Otu0728	0.2	0	0.1	0.49	0.1	88.73	unclass. *Gammaproteobacteria*	unclass. *Gammaproteobacteria*	unclass. *Gammaproteobacteria*	*Gammaproteobacteria*	*Proteobacteria*	*Bacteria*
Otu0729	0.2	0	0.1	0.49	0.1	88.83	unclass. *Bacteroidetes*	unclass. *Bacteroidetes*	unclass. *Bacteroidetes*	unclass. *Bacteroidetes*	*Bacteroidetes*	*Bacteria*
Otu0731	0.2	0	0.1	0.49	0.1	88.93	*Lentisphaera*	*Lentisphaeraceae*	*Lentisphaerales*	*Lentisphaeria*	*Lentisphaerae*	*Bacteria*
Otu0747	0.2	0	0.1	0.49	0.1	89.03	unclass. *Rhodobacteraceae*	*Rhodobacteraceae*	*Rhodobacterales*	*Alphaproteobacteria*	*Proteobacteria*	*Bacteria*
Otu0748	0.2	0	0.1	0.49	0.1	89.13	unclass. *Proteobacteria*	unclass. *Proteobacteria*	unclass. *Proteobacteria*	unclass. *Proteobacteria*	*Proteobacteria*	*Bacteria*
Otu0749	0.2	0	0.1	0.49	0.1	89.23	unclass. *Gammaproteobacteria*	unclass. *Gammaproteobacteria*	unclass. *Gammaproteobacteria*	*Gammaproteobacteria*	*Proteobacteria*	*Bacteria*
Otu0750	0.2	0	0.1	0.49	0.1	89.33	unclass. *Gammaproteobacteria*	unclass. *Gammaproteobacteria*	unclass. *Gammaproteobacteria*	*Gammaproteobacteria*	*Proteobacteria*	*Bacteria*
Otu0083	0.2	0	0.1	0.49	0.1	89.43	unclass. *Rhodobacteraceae*	*Rhodobacteraceae*	*Rhodobacterales*	*Alphaproteobacteria*	*Proteobacteria*	*Bacteria*
Otu0165	0.2	0	0.1	0.49	0.1	89.53	*Marinomonas*	*Oceanospirillaceae*	*Oceanospirillales*	*Gammaproteobacteria*	*Proteobacteria*	*Bacteria*
Otu0174	0.2	0	0.1	0.49	0.1	89.63	unclass. *Flavobacteriaceae*	*Flavobacteriaceae*	*Flavobacteriales*	*Flavobacteria*	*Bacteroidetes*	*Bacteria*
Otu0178	0.2	0	0.1	0.49	0.1	89.73	unclass. *Actinobacteria*	unclass. *Actinobacteria*	unclass. *Actinobacteria*	*Actinobacteria*	*Actinobacteria*	*Bacteria*
Otu0381	0.2	0	0.1	0.49	0.1	89.83	unclass. *Chitinophagaceae*	*Chitinophagaceae*	*Sphingobacteriales*	*Sphingobacteria*	*Bacteroidetes*	*Bacteria*
Otu0483	0.2	0	0.1	0.49	0.1	89.93	unclass. *Flavobacteriaceae*	*Flavobacteriaceae*	*Flavobacteriales*	*Flavobacteria*	*Bacteroidetes*	*Bacteria*
Otu0499	0.2	0	0.1	0.49	0.1	90.03	unclass. *Flavobacteriaceae*	*Flavobacteriaceae*	*Flavobacteriales*	*Flavobacteria*	*Bacteroidetes*	*Bacteria*
Autumn pH *in situ* 'no dilution' vs. 'serial dilution' (average dissimilarity: 97.4%)												
Otu0132	0	16.35	5.53	3.48	5.68	5.68	unclass. *Alteromonadales*	unclass. *Alteromonadales*	*Alteromonadales*	*Gammaproteobacteria*	*Proteobacteria*	*Bacteria*
Otu0106	0	9.15	3.05	1.94	3.13	8.81	*Marinomonas*	*Oceanospirillaceae*	*Oceanospirillales*	*Gammaproteobacteria*	*Proteobacteria*	*Bacteria*
Otu0107	0	7.12	2.38	3.23	2.44	11.25	unclass. *Rhodobacteraceae*	*Rhodobacteraceae*	*Rhodobacterales*	*Alphaproteobacteria*	*Proteobacteria*	*Bacteria*

OTU	Av.A_i (no)	Av.A_i (serial)	Av.δ_i	Av.δ_i/SD	Av.δ_i %	∑Av.δ_i %	Genus	Family	Order	Class	Phylum	Domain
Otu1090	5.7	0	1.9	8.49	1.95	13.21	unclass. *Bacteria*	unclass. *Bacteria*	unclass. *Bacteria*	unclass. *Bacteria*	unclass. *Bacteria*	*Bacteria*
Otu0005	4.32	0	1.5	1.67	1.54	14.75	*Pelagibacter*	SAR11-clade	*Rickettsiales*	*Alphaproteobacteria*	*Proteobacteria*	*Bacteria*
Otu0068	3.94	0	1.33	6.39	1.37	16.12	*Pelagibacter*	SAR11-clade	*Rickettsiales*	*Alphaproteobacteria*	*Proteobacteria*	*Bacteria*
Otu0053	3.86	0	1.31	5.04	1.34	17.46	unclass. *Rhodobacteraceae*	*Rhodobacteraceae*	*Rhodobacterales*	*Alphaproteobacteria*	*Proteobacteria*	*Bacteria*
Otu1091	3.7	0	1.24	7.96	1.27	18.73	unclass. *Flavobacteriaceae*	*Flavobacteriaceae*	*Flavobacteriales*	*Flavobacteria*	*Bacteroidetes*	*Bacteria*
Otu0823	3.6	0	1.21	5.67	1.25	19.98	unclass. *Legionellaceae*	*Legionellaceae*	*Legionellales*	*Gammaproteobacteria*	*Proteobacteria*	*Bacteria*
Otu0272	3.28	0	1.11	2.58	1.14	21.12	unclass. *Flavobacteriaceae*	*Flavobacteriaceae*	*Flavobacteriales*	*Flavobacteria*	*Bacteroidetes*	*Bacteria*
Otu0714	3.04	0	1.03	5.86	1.06	22.17	unclass. *Alphaproteobacteria*	unclass. *Alphaproteobacteria*	unclass. *Alphaproteobacteria*	*Alphaproteobacteria*	*Proteobacteria*	*Bacteria*
Otu1093	3.01	0	1	4.4	1.02	23.19	unclass. *Proteobacteria*	unclass. *Proteobacteria*	unclass. *Proteobacteria*	unclass. *Proteobacteria*	*Proteobacteria*	*Bacteria*
Otu0826	2.8	0	0.96	3.66	0.98	24.18	unclass. *Bacteroidetes*	unclass. *Bacteroidetes*	unclass. *Bacteroidetes*	unclass. *Bacteroidetes*	*Bacteroidetes*	*Bacteria*
Otu0471	0	2.82	0.95	3.79	0.98	25.15	*Oceaniserpentilla*	*Oceanospirillaceae*	*Oceanospirillales*	*Gammaproteobacteria*	*Proteobacteria*	*Bacteria*
Otu0681	2.85	0	0.94	3.26	0.97	26.12	unclass. *Bacteria*	unclass. *Bacteria*	unclass. *Bacteria*	unclass. *Bacteria*	unclass. *Bacteria*	*Bacteria*
Otu0692	2.7	0	0.91	3.74	0.93	27.05	unclass. *Bacteria*	unclass. *Bacteria*	unclass. *Bacteria*	unclass. *Bacteria*	unclass. *Bacteria*	*Bacteria*
Otu0052	2.7	0	0.91	6.27	0.93	27.98	unclass. *Betaproteobacteria*	unclass. *Betaproteobacteria*	unclass. *Betaproteobacteria*	*Betaproteobacteria*	*Proteobacteria*	*Bacteria*
Otu1094	2.68	0	0.9	12.88	0.93	28.91	unclass. *Bacteroidetes*	unclass. *Bacteroidetes*	unclass. *Bacteroidetes*	unclass. *Bacteroidetes*	*Bacteroidetes*	*Bacteria*
Otu0408	2.67	0	0.9	26.28	0.92	29.83	unclass. *Flavobacteriaceae*	*Flavobacteriaceae*	*Flavobacteriales*	*Flavobacteria*	*Bacteroidetes*	*Bacteria*
Otu0983	2.52	0	0.83	4.66	0.86	30.69	unclass. *Flavobacteriales*	unclass. *Flavobacteriales*	*Flavobacteriales*	*Flavobacteria*	*Bacteroidetes*	*Bacteria*
Otu0469	2.44	0	0.82	4.57	0.84	31.53	unclass. *Bacteria*	unclass. *Bacteria*	unclass. *Bacteria*	unclass. *Bacteria*	unclass. *Bacteria*	*Bacteria*
Otu1023	2.35	0	0.79	6.93	0.82	32.35	unclass. *Lentisphaeria*	unclass. *Lentisphaeria*	unclass. *Lentisphaeria*	*Lentisphaeria*	*Lentisphaerae*	*Bacteria*
Otu0001	4.28	3.02	0.78	1.12	0.8	33.15	unclass. *Flavobacteriaceae*	*Flavobacteriaceae*	*Flavobacteriales*	*Flavobacteria*	*Bacteroidetes*	*Bacteria*
Otu0467	2.24	0	0.77	2.48	0.79	33.94	unclass. *Rhodospirillaceae*	*Rhodospirillaceae*	*Rhodospirillales*	*Alphaproteobacteria*	*Proteobacteria*	*Bacteria*
Otu0705	2.29	0	0.74	1.72	0.76	34.7	*Planctomyces*	*Planctomycetaceae*	*Planctomycetales*	*Planctomycetacia*	*Planctomycetes*	*Bacteria*
Otu0764	2.18	0	0.73	10.28	0.75	35.45	unclass. *Proteobacteria*	unclass. *Proteobacteria*	unclass. *Proteobacteria*	unclass. *Proteobacteria*	*Proteobacteria*	*Bacteria*
Otu0123	0.2	2.23	0.72	1.64	0.74	36.19	unclass. *Gammaproteobacteria*	unclass. *Gammaproteobacteria*	unclass. *Gammaproteobacteria*	*Gammaproteobacteria*	*Proteobacteria*	*Bacteria*
Otu0494	2.08	0	0.69	2.59	0.71	36.89	unclass. *Flavobacteriaceae*	*Flavobacteriaceae*	*Flavobacteriales*	*Flavobacteria*	*Bacteroidetes*	*Bacteria*
Otu0551	0	2	0.67	1.67	0.69	37.58	*Arcobacter*	*Campylobacteraceae*	*Campylobacterales*	*Epsilonproteobacteria*	*Proteobacteria*	*Bacteria*
Otu0691	1.94	0	0.66	6.52	0.67	38.25	*Planctomyces*	*Planctomycetaceae*	*Planctomycetales*	*Planctomycetacia*	*Planctomycetes*	*Bacteria*

OTU	$Av.A_i$ (no)	$Av.A_i$ (serial)	$Av.\delta_i$	$Av.\delta_i/SD$	$Av.\delta_i$ %	$\sum Av.\delta_i$ %	Genus	Family	Order	Class	Phylum	Domain
Otu0473	1.92	0	0.65	4.9	0.67	38.92	unclass. *Alphaproteobacteria*	unclass. *Alphaproteobacteria*	unclass. *Alphaproteobacteria*	*Alphaproteobacteria*	*Proteobacteria*	*Bacteria*
Otu0490	1.94	0	0.65	11.6	0.67	39.59	unclass. *Burkholderiales*	unclass. *Burkholderiales*	*Burkholderiales*	*Betaproteobacteria*	*Proteobacteria*	*Bacteria*
Otu0036	0.2	2.12	0.65	1.54	0.67	40.26	*Glaciecola*	*Alteromonadaceae*	*Alteromonadales*	*Gammaproteobacteria*	*Proteobacteria*	*Bacteria*
Otu1092	1.84	0	0.61	1.17	0.63	40.88	*Cycloclasticus*	*Piscirickettsiaceae*	*Thiotrichales*	*Gammaproteobacteria*	*Proteobacteria*	*Bacteria*
Otu0596	1.79	0	0.6	3.62	0.62	41.5	*Neptunomonas*	*Oceanospirillaceae*	*Oceanospirillales*	*Gammaproteobacteria*	*Proteobacteria*	*Bacteria*
Otu1097	1.77	0	0.6	5.05	0.61	42.11	unclass. *Proteobacteria*	unclass. *Proteobacteria*	unclass. *Proteobacteria*	unclass. *Proteobacteria*	*Proteobacteria*	*Bacteria*
Otu0776	1.76	0	0.58	3.32	0.6	42.71	unclass. *Proteobacteria*	unclass. *Proteobacteria*	unclass. *Proteobacteria*	unclass. *Proteobacteria*	*Proteobacteria*	*Bacteria*
Otu0048	1.71	0	0.58	4.78	0.59	43.3	unclass. *Flavobacteriaceae*	*Flavobacteriaceae*	*Flavobacteriales*	*Flavobacteria*	*Bacteroidetes*	*Bacteria*
Otu1081	1.66	0	0.54	2.27	0.56	43.86	unclass. *Flavobacteriales*	unclass. *Flavobacteriales*	*Flavobacteriales*	*Flavobacteria*	*Bacteroidetes*	*Bacteria*
Otu0541	1.6	0	0.54	8.95	0.55	44.42	unclass. *Rhodobacteraceae*	*Rhodobacteraceae*	*Rhodobacterales*	*Alphaproteobacteria*	*Proteobacteria*	*Bacteria*
Otu0281	1.52	0	0.51	5.84	0.52	44.94	unclass. *Flavobacteriales*	unclass. *Flavobacteriales*	*Flavobacteriales*	*Flavobacteria*	*Bacteroidetes*	*Bacteria*
Otu0771	1.48	0	0.51	2.78	0.52	45.46	unclass. *Rhodobacteraceae*	*Rhodobacteraceae*	*Rhodobacterales*	*Alphaproteobacteria*	*Proteobacteria*	*Bacteria*
Otu0972	1.48	0	0.49	3.63	0.51	45.96	*Nisaea*	*Rhodospirillaceae*	*Rhodospirillales*	*Alphaproteobacteria*	*Proteobacteria*	*Bacteria*
Otu0696	1.48	0	0.48	1.9	0.49	46.46	unclass. *Flavobacteriaceae*	*Flavobacteriaceae*	*Flavobacteriales*	*Flavobacteria*	*Bacteroidetes*	*Bacteria*
Otu0694	1.41	0	0.48	2.94	0.49	46.95	unclass. *Alphaproteobacteria*	unclass. *Alphaproteobacteria*	unclass. *Alphaproteobacteria*	*Alphaproteobacteria*	*Proteobacteria*	*Bacteria*
Otu0509	1.39	0	0.47	4.65	0.48	47.43	unclass. *Gammaproteobacteria*	unclass. *Gammaproteobacteria*	unclass. *Gammaproteobacteria*	*Gammaproteobacteria*	*Proteobacteria*	*Bacteria*
Otu0546	0	1.37	0.46	1.36	0.47	47.9	unclass. *Bacteria*	unclass. *Bacteria*	unclass. *Bacteria*	unclass. *Bacteria*	unclass. *Bacteria*	*Bacteria*
Otu1105	1.37	0	0.45	4.45	0.47	48.37	unclass. *Proteobacteria*	unclass. *Proteobacteria*	unclass. *Proteobacteria*	unclass. *Proteobacteria*	*Proteobacteria*	*Bacteria*
Otu0521	1.49	0.4	0.43	1.3	0.45	48.81	unclass. *Rhodobacteraceae*	*Rhodobacteraceae*	*Rhodobacterales*	*Alphaproteobacteria*	*Proteobacteria*	*Bacteria*
Otu0695	1.29	0	0.43	4.41	0.44	49.25	unclass. *Proteobacteria*	unclass. *Proteobacteria*	unclass. *Proteobacteria*	unclass. *Proteobacteria*	*Proteobacteria*	*Bacteria*
Otu0710	1.24	0	0.43	1.18	0.44	49.69	unclass. *Gammaproteobacteria*	unclass. *Gammaproteobacteria*	unclass. *Gammaproteobacteria*	*Gammaproteobacteria*	*Proteobacteria*	*Bacteria*
Otu0485	1.26	0	0.42	1.94	0.43	50.12	unclass. *Alphaproteobacteria*	unclass. *Alphaproteobacteria*	unclass. *Alphaproteobacteria*	*Alphaproteobacteria*	*Proteobacteria*	*Bacteria*
Otu0084	1.23	0	0.41	4.57	0.42	50.54	unclass. *Gammaproteobacteria*	unclass. *Gammaproteobacteria*	unclass. *Gammaproteobacteria*	*Gammaproteobacteria*	*Proteobacteria*	*Bacteria*
Otu0960	1.23	0	0.4	1.72	0.41	50.95	unclass. *Bacteria*	unclass. *Bacteria*	unclass. *Bacteria*	unclass. *Bacteria*	unclass. *Bacteria*	*Bacteria*
Otu1096	1.18	0	0.4	1.85	0.41	51.36	unclass. *Lentisphaeria*	unclass. *Lentisphaeria*	unclass. *Lentisphaeria*	*Lentisphaeria*	*Lentisphaerae*	*Bacteria*
Otu0898	1.13	0	0.4	1.43	0.41	51.77	*Sphingopyxis*	*Sphingomonadaceae*	*Sphingomonadales*	*Alphaproteobacteria*	*Proteobacteria*	*Bacteria*
Otu0261	1.17	0	0.39	1.79	0.4	52.17	*Winogradskyella*	*Flavobacteriaceae*	*Flavobacteriales*	*Flavobacteria*	*Bacteroidetes*	*Bacteria*

OTU	$Av.A_i$ (no)	$Av.A_i$ (serial)	$Av.\delta_i$	$Av.\delta_i/SD$	$Av.\delta_i$ %	$\sum Av.\delta_i$ %	Genus	Family	Order	Class	Phylum	Domain
Otu1115	1.17	0	0.39	1.64	0.4	52.56	*Marinobacter*	*Alteromonadaceae*	*Alteromonadales*	*Gammaproteobacteria*	*Proteobacteria*	*Bacteria*
Otu0778	1.17	0	0.38	1.76	0.39	52.95	unclass. *Gammaproteobacteria*	unclass. *Gammaproteobacteria*	unclass. *Gammaproteobacteria*	*Gammaproteobacteria*	*Proteobacteria*	*Bacteria*
Otu0157	1.04	0	0.37	1.03	0.38	53.33	unclass. *Gammaproteobacteria*	unclass. *Gammaproteobacteria*	unclass. *Gammaproteobacteria*	*Gammaproteobacteria*	*Proteobacteria*	*Bacteria*
Otu1100	1.09	0	0.37	1.68	0.38	53.71	unclass. *Planctomycetaceae*	*Planctomycetaceae*	*Planctomycetales*	*Planctomycetacia*	*Planctomycetes*	*Bacteria*
Otu1113	1.11	0	0.36	1.79	0.37	54.08	unclass. *Gammaproteobacteria*	unclass. *Gammaproteobacteria*	unclass. *Gammaproteobacteria*	*Gammaproteobacteria*	*Proteobacteria*	*Bacteria*
Otu0737	1.08	0	0.36	8.36	0.37	54.45	unclass. *Gammaproteobacteria*	unclass. *Gammaproteobacteria*	unclass. *Gammaproteobacteria*	*Gammaproteobacteria*	*Proteobacteria*	*Bacteria*
Otu1125	1.11	0	0.36	1.83	0.37	54.82	*Planctomyces*	*Planctomycetaceae*	*Planctomycetales*	*Planctomycetacia*	*Planctomycetes*	*Bacteria*
Otu0840	1.01	0	0.34	0.8	0.35	55.18	unclass. *Flavobacteriaceae*	*Flavobacteriaceae*	*Flavobacteriales*	*Flavobacteria*	*Bacteroidetes*	*Bacteria*
Otu1102	0.93	0	0.33	0.77	0.33	55.51	unclass. *Proteobacteria*	unclass. *Proteobacteria*	unclass. *Proteobacteria*	unclass. *Proteobacteria*	*Proteobacteria*	*Bacteria*
Otu0255	0.97	0	0.32	1.81	0.32	55.83	*Sphingopyxis*	*Sphingomonadaceae*	*Sphingomonadales*	*Alphaproteobacteria*	*Proteobacteria*	*Bacteria*
Otu0645	0.97	0	0.31	1.17	0.31	56.15	*Arcobacter*	*Campylobacteraceae*	*Campylobacterales*	*Epsilonproteobacteria*	*Proteobacteria*	*Bacteria*
Otu0682	0.89	0	0.3	1.15	0.31	56.46	unclass. *Oceanospirillaceae*	*Oceanospirillaceae*	*Oceanospirillales*	*Gammaproteobacteria*	*Proteobacteria*	*Bacteria*
Otu1111	0.88	0	0.3	1.86	0.31	56.77	unclass. *Proteobacteria*	unclass. *Proteobacteria*	unclass. *Proteobacteria*	unclass. *Proteobacteria*	*Proteobacteria*	*Bacteria*
Otu0186	0.85	0	0.3	1.19	0.3	57.07	unclass. *Planctomycetaceae*	*Planctomycetaceae*	*Planctomycetales*	*Planctomycetacia*	*Planctomycetes*	*Bacteria*
Otu1217	0.85	0	0.29	0.75	0.3	57.38	unclass. *Alteromonadales*	unclass. *Alteromonadales*	*Alteromonadales*	*Gammaproteobacteria*	*Proteobacteria*	*Bacteria*
Otu0202	0.88	0	0.29	1.77	0.3	57.67	unclass. *Alteromonadaceae*	*Alteromonadaceae*	*Alteromonadales*	*Gammaproteobacteria*	*Proteobacteria*	*Bacteria*
Otu0959	0.88	0	0.29	1.77	0.3	57.97	unclass. *Rhodobacteraceae*	*Rhodobacteraceae*	*Rhodobacterales*	*Alphaproteobacteria*	*Proteobacteria*	*Bacteria*
Otu0137	0.88	0	0.29	1.85	0.3	58.27	unclass. *Bacteroidetes*	unclass. *Bacteroidetes*	unclass. *Bacteroidetes*	unclass. *Bacteroidetes*	*Bacteroidetes*	*Bacteria*
Otu0611	0.83	0	0.29	1.11	0.29	58.56	unclass. *Bacteroidetes*	unclass. *Bacteroidetes*	unclass. *Bacteroidetes*	unclass. *Bacteroidetes*	*Bacteroidetes*	*Bacteria*
Otu0846	0.83	0	0.29	1.15	0.29	58.86	unclass. *Nannocystaceae*	*Nannocystaceae*	*Myxococcales*	*Deltaproteobacteria*	*Proteobacteria*	*Bacteria*
Otu0844	0.85	0	0.28	1.03	0.29	59.14	unclass. *Flavobacteriaceae*	*Flavobacteriaceae*	*Flavobacteriales*	*Flavobacteria*	*Bacteroidetes*	*Bacteria*
Otu1219	0.88	0	0.28	0.78	0.29	59.43	unclass. *Bacteria*	unclass. *Bacteria*	unclass. *Bacteria*	unclass. *Bacteria*	unclass. *Bacteria*	*Bacteria*
Otu0601	0.8	0	0.27	1.93	0.28	59.7	unclass. *Flammeovirgaceae*	*Flammeovirgaceae*	*Sphingobacteriales*	*Sphingobacteria*	*Bacteroidetes*	*Bacteria*
Otu0817	0.8	0	0.27	1.93	0.28	59.98	unclass. *Bacteroidetes*	unclass. *Bacteroidetes*	unclass. *Bacteroidetes*	unclass. *Bacteroidetes*	*Bacteroidetes*	*Bacteria*
Otu0502	0.83	0	0.27	1.16	0.28	60.26	unclass. *Gammaproteobacteria*	unclass. *Gammaproteobacteria*	unclass. *Gammaproteobacteria*	*Gammaproteobacteria*	*Proteobacteria*	*Bacteria*
Otu0170	0.8	0	0.27	1.93	0.27	60.53	unclass. *Bacteria*	unclass. *Bacteria*	unclass. *Bacteria*	unclass. *Bacteria*	unclass. *Bacteria*	*Bacteria*
Otu0947	0.8	0	0.26	0.49	0.27	60.81	unclass. *Bacteroidetes*	unclass. *Bacteroidetes*	unclass. *Bacteroidetes*	unclass. *Bacteroidetes*	*Bacteroidetes*	*Bacteria*

OTU	Av.A_i (no)	Av.A_i (serial)	Av.δ_i	Av.δ_i/SD	Av.δ_i %	∑Av.δ_i %	Genus	Family	Order	Class	Phylum	Domain
Otu0243	0.77	0	0.26	1.14	0.27	61.08	unclass. *Gammaproteobacteria*	unclass. *Gammaproteobacteria*	unclass. *Gammaproteobacteria*	*Gammaproteobacteria*	*Proteobacteria*	*Bacteria*
Otu0621	1.15	0.6	0.26	1.21	0.27	61.34	*Colwellia*	*Colwelliaceae*	*Alteromonadales*	*Gammaproteobacteria*	*Proteobacteria*	*Bacteria*
Otu0375	0.75	0	0.26	1.09	0.27	61.61	unclass. *Bacteroidetes*	unclass. *Bacteroidetes*	unclass. *Bacteroidetes*	unclass. *Bacteroidetes*	*Bacteroidetes*	*Bacteria*
Otu0986	0.75	0	0.26	1.04	0.26	61.87	unclass. *Planctomycetaceae*	*Planctomycetaceae*	*Planctomycetales*	*Planctomycetacia*	*Planctomycetes*	*Bacteria*
Otu1118	0.77	0	0.26	1.19	0.26	62.14	unclass. *Oceanospirillales*	unclass. *Oceanospirillales*	*Oceanospirillales*	*Gammaproteobacteria*	*Proteobacteria*	*Bacteria*
Otu0742	0.77	0	0.25	1.19	0.26	62.4	unclass. *Rhodobacteraceae*	*Rhodobacteraceae*	*Rhodobacterales*	*Alphaproteobacteria*	*Proteobacteria*	*Bacteria*
Otu0191	0.68	0	0.25	0.79	0.25	62.65	unclass. *Proteobacteria*	unclass. *Proteobacteria*	unclass. *Proteobacteria*	unclass. *Proteobacteria*	*Proteobacteria*	*Bacteria*
Otu1099	0.77	0	0.25	1.16	0.25	62.9	unclass. *Lentisphaeria*	unclass. *Lentisphaeria*	unclass. *Lentisphaeria*	*Lentisphaeria*	*Lentisphaerae*	*Bacteria*
Otu1109	0.77	0	0.24	1.17	0.25	63.15	unclass. *Saprospiraceae*	*Saprospiraceae*	*Sphingobacteriales*	*Sphingobacteria*	*Bacteroidetes*	*Bacteria*
Otu0765	0.68	0	0.24	1.15	0.24	63.39	*Pelagicoccus*	*Puniceicoccaceae*	*Puniceicoccales*	*Opitutae*	*Verrucomicrobia*	*Bacteria*
Otu1110	0.68	0	0.24	1.15	0.24	63.64	unclass. *Alphaproteobacteria*	unclass. *Alphaproteobacteria*	unclass. *Alphaproteobacteria*	*Alphaproteobacteria*	*Proteobacteria*	*Bacteria*
Otu1281	0.63	0	0.24	0.49	0.24	63.88	unclass. *Proteobacteria*	unclass. *Proteobacteria*	unclass. *Proteobacteria*	unclass. *Proteobacteria*	*Proteobacteria*	*Bacteria*
Otu0284	0.68	0	0.24	1.18	0.24	64.12	unclass. *Saprospiraceae*	*Saprospiraceae*	*Sphingobacteriales*	*Sphingobacteria*	*Bacteroidetes*	*Bacteria*
Otu1107	0.68	0	0.23	1.18	0.24	64.36	unclass. *Planctomycetaceae*	*Planctomycetaceae*	*Planctomycetales*	*Planctomycetacia*	*Planctomycetes*	*Bacteria*
Otu0758	0.69	0	0.23	0.8	0.23	64.6	unclass. *Bacteria*	unclass. *Bacteria*	unclass. *Bacteria*	unclass. *Bacteria*	unclass. *Bacteria*	*Bacteria*
Otu1186	0.68	0	0.23	1.13	0.23	64.83	unclass. *Bacteria*	unclass. *Bacteria*	unclass. *Bacteria*	unclass. *Bacteria*	unclass. *Bacteria*	*Bacteria*
Otu1101	0.68	0	0.23	1.17	0.23	65.06	*Porphyrobacter*	*Erythrobacteraceae*	*Sphingomonadales*	*Alphaproteobacteria*	*Proteobacteria*	*Bacteria*
Otu0503	0.68	0	0.23	1.18	0.23	65.29	unclass. *Bacteroidetes*	unclass. *Bacteroidetes*	unclass. *Bacteroidetes*	unclass. *Bacteroidetes*	*Bacteroidetes*	*Bacteria*
Otu0483	0.68	0	0.22	1.13	0.23	65.52	unclass. *Flavobacteriaceae*	*Flavobacteriaceae*	*Flavobacteriales*	*Flavobacteria*	*Bacteroidetes*	*Bacteria*
Otu1119	0.68	0	0.22	1.17	0.22	65.75	unclass. *Bacteroidetes*	unclass. *Bacteroidetes*	unclass. *Bacteroidetes*	unclass. *Bacteroidetes*	*Bacteroidetes*	*Bacteria*
Otu0075	0.63	0	0.21	0.8	0.22	65.96	unclass. *Alphaproteobacteria*	unclass. *Alphaproteobacteria*	unclass. *Alphaproteobacteria*	*Alphaproteobacteria*	*Proteobacteria*	*Bacteria*
Otu0044	0.6	0	0.21	1.2	0.22	66.18	unclass. *Gammaproteobacteria*	unclass. *Gammaproteobacteria*	unclass. *Gammaproteobacteria*	*Gammaproteobacteria*	*Proteobacteria*	*Bacteria*
Otu0504	0.6	0	0.2	1.19	0.21	66.39	unclass. *Alphaproteobacteria*	unclass. *Alphaproteobacteria*	unclass. *Alphaproteobacteria*	*Alphaproteobacteria*	*Proteobacteria*	*Bacteria*
Otu0703	0.63	0	0.2	0.79	0.21	66.6	unclass. *Gammaproteobacteria*	unclass. *Gammaproteobacteria*	unclass. *Gammaproteobacteria*	*Gammaproteobacteria*	*Proteobacteria*	*Bacteria*
Otu1095	0.6	0	0.2	0.75	0.2	66.8	unclass. *Bacteria*	unclass. *Bacteria*	unclass. *Bacteria*	unclass. *Bacteria*	unclass. *Bacteria*	*Bacteria*
Otu0571	0	0.6	0.2	0.73	0.2	67.01	*Marinomonas*	*Oceanospirillaceae*	*Oceanospirillales*	*Gammaproteobacteria*	*Proteobacteria*	*Bacteria*
Otu0677	0.6	0	0.2	0.73	0.2	67.21	*Thalassobius*	*Rhodobacteraceae*	*Rhodobacterales*	*Alphaproteobacteria*	*Proteobacteria*	*Bacteria*

OTU	Av.A_i (no)	Av.A_i (serial)	Av.δ_i	Av.δ_i/SD	Av.δ_i %	∑Av.δ_i %	Genus	Family	Order	Class	Phylum	Domain
Otu1133	0.6	0	0.2	1.19	0.2	67.41	unclass. *Bacteria*	unclass. *Bacteria*	unclass. *Bacteria*	unclass. *Bacteria*	unclass. *Bacteria*	*Bacteria*
Otu0062	0.55	0	0.19	0.73	0.2	67.61	*Haliea*	*Alteromonadaceae*	*Alteromonadales*	*Gammaproteobacteria*	*Proteobacteria*	*Bacteria*
Otu0707	0.55	0	0.19	0.73	0.2	67.8	*Haliea*	*Alteromonadaceae*	*Alteromonadales*	*Gammaproteobacteria*	*Proteobacteria*	*Bacteria*
Otu0333	0.6	0	0.19	1.2	0.2	68	unclass. *Gammaproteobacteria*	unclass. *Gammaproteobacteria*	unclass. *Gammaproteobacteria*	*Gammaproteobacteria*	*Proteobacteria*	*Bacteria*
Otu0974	0.57	0	0.18	0.8	0.19	68.19	unclass. *Flavobacteriaceae*	*Flavobacteriaceae*	*Flavobacteriales*	*Flavobacteria*	*Bacteroidetes*	*Bacteria*
Otu0379	0.55	0	0.18	0.78	0.19	68.38	unclass. *Proteobacteria*	unclass. *Proteobacteria*	unclass. *Proteobacteria*	unclass. *Proteobacteria*	*Proteobacteria*	*Bacteria*
Otu0143	0.48	0	0.18	0.77	0.18	68.56	unclass. *Gammaproteobacteria*	unclass. *Gammaproteobacteria*	unclass. *Gammaproteobacteria*	*Gammaproteobacteria*	*Proteobacteria*	*Bacteria*
Otu0192	0.48	0	0.18	0.77	0.18	68.74	unclass. *Gammaproteobacteria*	unclass. *Gammaproteobacteria*	unclass. *Gammaproteobacteria*	*Gammaproteobacteria*	*Proteobacteria*	*Bacteria*
Otu0359	0.48	0	0.17	0.79	0.18	68.92	unclass. *Gammaproteobacteria*	unclass. *Gammaproteobacteria*	unclass. *Gammaproteobacteria*	*Gammaproteobacteria*	*Proteobacteria*	*Bacteria*
Otu1114	0.48	0	0.17	0.79	0.18	69.09	unclass. *Microbacteriaceae*	*Microbacteriaceae*	*Actinomycetales*	*Actinobacteria*	*Actinobacteria*	*Bacteria*
Otu1193	0.48	0	0.17	0.77	0.18	69.27	unclass. *Saprospiraceae*	*Saprospiraceae*	*Sphingobacteriales*	*Sphingobacteria*	*Bacteroidetes*	*Bacteria*
Otu0731	0.45	0	0.17	0.49	0.17	69.44	*Lentisphaera*	*Lentisphaeraceae*	*Lentisphaerales*	*Lentisphaeria*	*Lentisphaerae*	*Bacteria*
Otu0761	0.48	0	0.17	0.79	0.17	69.61	unclass. *Verrucomicrobiaceae*	*Verrucomicrobiaceae*	*Verrucomicrobiales*	*Verrucomicrobiae*	*Verrucomicrobia*	*Bacteria*
Otu0812	0.48	0	0.16	0.79	0.17	69.78	unclass. *Gammaproteobacteria*	unclass. *Gammaproteobacteria*	unclass. *Gammaproteobacteria*	*Gammaproteobacteria*	*Proteobacteria*	*Bacteria*
Otu0809	0.49	0	0.16	0.49	0.17	69.95	unclass. *Gammaproteobacteria*	unclass. *Gammaproteobacteria*	unclass. *Gammaproteobacteria*	*Gammaproteobacteria*	*Proteobacteria*	*Bacteria*
Otu0570	0	0.48	0.16	0.78	0.16	70.11	*Enhydrobacter*	*Moraxellaceae*	*Pseudomonadales*	*Gammaproteobacteria*	*Proteobacteria*	*Bacteria*
Otu0046	0.48	0	0.16	0.78	0.16	70.27	unclass. *Flavobacteriaceae*	*Flavobacteriaceae*	*Flavobacteriales*	*Flavobacteria*	*Bacteroidetes*	*Bacteria*
Otu0213	0.48	0	0.16	0.79	0.16	70.43	unclass. *Gammaproteobacteria*	unclass. *Gammaproteobacteria*	unclass. *Gammaproteobacteria*	*Gammaproteobacteria*	*Proteobacteria*	*Bacteria*
Otu0744	0.48	0	0.16	0.79	0.16	70.6	unclass. *Gammaproteobacteria*	unclass. *Gammaproteobacteria*	unclass. *Gammaproteobacteria*	*Gammaproteobacteria*	*Proteobacteria*	*Bacteria*
Otu1343	0.48	0	0.16	0.79	0.16	70.76	*Planctomyces*	*Planctomycetaceae*	*Planctomycetales*	*Planctomycetacia*	*Planctomycetes*	*Bacteria*
Otu0510	0.48	0	0.16	0.78	0.16	70.92	unclass. *Gammaproteobacteria*	unclass. *Gammaproteobacteria*	unclass. *Gammaproteobacteria*	*Gammaproteobacteria*	*Proteobacteria*	*Bacteria*
Otu0421	0.48	0	0.16	0.77	0.16	71.08	unclass. *Gammaproteobacteria*	unclass. *Gammaproteobacteria*	unclass. *Gammaproteobacteria*	*Gammaproteobacteria*	*Proteobacteria*	*Bacteria*
Otu0833	0.48	0	0.15	0.79	0.16	71.23	*Alcanivorax*	*Alcanivoracaceae*	*Oceanospirillales*	*Gammaproteobacteria*	*Proteobacteria*	*Bacteria*
Otu1197	0.4	0	0.15	0.49	0.15	71.39	unclass. *Gammaproteobacteria*	unclass. *Gammaproteobacteria*	unclass. *Gammaproteobacteria*	*Gammaproteobacteria*	*Proteobacteria*	*Bacteria*
Otu0041	0.2	0.4	0.15	0.86	0.15	71.54	*Sulfitobacter*	*Rhodobacteraceae*	*Rhodobacterales*	*Alphaproteobacteria*	*Proteobacteria*	*Bacteria*
Otu0622	0.2	0.4	0.15	0.87	0.15	71.69	*Colwellia*	*Colwelliaceae*	*Alteromonadales*	*Gammaproteobacteria*	*Proteobacteria*	*Bacteria*
Otu0738	0.4	0	0.15	0.8	0.15	71.84	unclass. *Alphaproteobacteria*	unclass. *Alphaproteobacteria*	unclass. *Alphaproteobacteria*	*Alphaproteobacteria*	*Proteobacteria*	*Bacteria*

OTU	Av.A_i (no)	Av.A_i (serial)	Av.δ_i	Av.δ_i/SD	Av.δ_i %	∑Av.δ_i %	Genus	Family	Order	Class	Phylum	Domain
Otu1296	0.4	0	0.15	0.8	0.15	71.99	unclass. *Proteobacteria*	unclass. *Proteobacteria*	unclass. *Proteobacteria*	unclass. *Proteobacteria*	*Proteobacteria*	*Bacteria*
Otu1300	0.4	0	0.15	0.8	0.15	72.13	unclass. *Proteobacteria*	unclass. *Proteobacteria*	unclass. *Proteobacteria*	unclass. *Proteobacteria*	*Proteobacteria*	*Bacteria*
Otu0011	0.4	0	0.14	0.8	0.15	72.28	unclass. *Flavobacteriaceae*	*Flavobacteriaceae*	*Flavobacteriales*	*Flavobacteria*	*Bacteroidetes*	*Bacteria*
Otu0915	0.4	0	0.14	0.8	0.15	72.42	unclass. *Alphaproteobacteria*	unclass. *Alphaproteobacteria*	unclass. *Alphaproteobacteria*	*Alphaproteobacteria*	*Proteobacteria*	*Bacteria*
Otu0219	0.4	0	0.14	0.8	0.14	72.57	unclass. *Erythrobacteraceae*	*Erythrobacteraceae*	*Sphingomonadales*	*Alphaproteobacteria*	*Proteobacteria*	*Bacteria*
Otu0368	0.4	0	0.14	0.79	0.14	72.71	unclass. *Saprospiraceae*	*Saprospiraceae*	*Sphingobacteriales*	*Sphingobacteria*	*Bacteroidetes*	*Bacteria*
Otu0300	0.4	0	0.14	0.8	0.14	72.85	unclass. *Gammaproteobacteria*	unclass. *Gammaproteobacteria*	unclass. *Gammaproteobacteria*	*Gammaproteobacteria*	*Proteobacteria*	*Bacteria*
Otu1061	0	0.4	0.13	0.8	0.14	72.98	unclass. *Bacteria*	unclass. *Bacteria*	unclass. *Bacteria*	unclass. *Bacteria*	unclass. *Bacteria*	*Bacteria*
Otu0426	0.4	0	0.13	0.8	0.14	73.12	unclass. *Gammaproteobacteria*	unclass. *Gammaproteobacteria*	unclass. *Gammaproteobacteria*	*Gammaproteobacteria*	*Proteobacteria*	*Bacteria*
Otu0998	0.4	0	0.13	0.8	0.14	73.26	unclass. *Gammaproteobacteria*	unclass. *Gammaproteobacteria*	unclass. *Gammaproteobacteria*	*Gammaproteobacteria*	*Proteobacteria*	*Bacteria*
Otu1130	0.4	0	0.13	0.8	0.14	73.4	unclass. *Bacteria*	unclass. *Bacteria*	unclass. *Bacteria*	unclass. *Bacteria*	unclass. *Bacteria*	*Bacteria*
Otu1333	0.4	0	0.13	0.8	0.14	73.53	unclass. *Legionellaceae*	*Legionellaceae*	*Legionellales*	*Gammaproteobacteria*	*Proteobacteria*	*Bacteria*
Otu1348	0.4	0	0.13	0.8	0.14	73.67	unclass. *Gammaproteobacteria*	unclass. *Gammaproteobacteria*	unclass. *Gammaproteobacteria*	*Gammaproteobacteria*	*Proteobacteria*	*Bacteria*
Otu0177	0.4	0	0.13	0.8	0.13	73.8	*Haliea*	*Alteromonadaceae*	*Alteromonadales*	*Gammaproteobacteria*	*Proteobacteria*	*Bacteria*
Otu0194	0.4	0	0.13	0.8	0.13	73.93	unclass. *Bacteroidetes*	unclass. *Bacteroidetes*	unclass. *Bacteroidetes*	unclass. *Bacteroidetes*	*Bacteroidetes*	*Bacteria*
Otu0464	0.4	0	0.13	0.8	0.13	74.07	unclass. *Bacteroidetes*	unclass. *Bacteroidetes*	unclass. *Bacteroidetes*	unclass. *Bacteroidetes*	*Bacteroidetes*	*Bacteria*
Otu0973	0.4	0	0.13	0.8	0.13	74.2	unclass. *Flavobacteriaceae*	*Flavobacteriaceae*	*Flavobacteriales*	*Flavobacteria*	*Bacteroidetes*	*Bacteria*
Otu1153	0.4	0	0.13	0.8	0.13	74.33	unclass. *Alphaproteobacteria*	unclass. *Alphaproteobacteria*	unclass. *Alphaproteobacteria*	*Alphaproteobacteria*	*Proteobacteria*	*Bacteria*
Otu0203	0.4	0	0.13	0.8	0.13	74.46	*Pelagibacter*	SAR11-clade	*Rickettsiales*	*Alphaproteobacteria*	*Proteobacteria*	*Bacteria*
Otu0741	0.4	0	0.13	0.8	0.13	74.6	unclass. *Alphaproteobacteria*	unclass. *Alphaproteobacteria*	unclass. *Alphaproteobacteria*	*Alphaproteobacteria*	*Proteobacteria*	*Bacteria*
Otu0859	0.4	0	0.13	0.8	0.13	74.73	unclass. *Flavobacteriaceae*	*Flavobacteriaceae*	*Flavobacteriales*	*Flavobacteria*	*Bacteroidetes*	*Bacteria*
Otu0934	0.4	0	0.13	0.8	0.13	74.86	unclass. *Bacteria*	unclass. *Bacteria*	unclass. *Bacteria*	unclass. *Bacteria*	unclass. *Bacteria*	*Bacteria*
Otu1147	0.4	0	0.13	0.8	0.13	74.99	unclass. *Bacteria*	unclass. *Bacteria*	unclass. *Bacteria*	unclass. *Bacteria*	unclass. *Bacteria*	*Bacteria*
Otu1234	0.4	0	0.13	0.8	0.13	75.12	unclass. *Oceanospirillaceae*	*Oceanospirillaceae*	*Oceanospirillales*	*Gammaproteobacteria*	*Proteobacteria*	*Bacteria*
Otu0495	0.4	0	0.13	0.8	0.13	75.25	unclass. *Flavobacteriales*	unclass. *Flavobacteriales*	*Flavobacteriales*	*Flavobacteria*	*Bacteroidetes*	*Bacteria*
Otu1049	0.4	0	0.13	0.8	0.13	75.38	unclass. *Gammaproteobacteria*	unclass. *Gammaproteobacteria*	unclass. *Gammaproteobacteria*	*Gammaproteobacteria*	*Proteobacteria*	*Bacteria*
Otu1106	0.4	0	0.13	0.8	0.13	75.51	unclass. *Bacteria*	unclass. *Bacteria*	unclass. *Bacteria*	unclass. *Bacteria*	unclass. *Bacteria*	*Bacteria*

OTU	Av.A_i (no)	Av.A_i (serial)	Av.δ_i	Av.δ_i/SD	Av.δ_i %	∑Av.δ_i %	Genus	Family	Order	Class	Phylum	Domain
Otu1116	0.4	0	0.13	0.8	0.13	75.63	unclass. *Gammaproteobacteria*	unclass. *Gammaproteobacteria*	unclass. *Gammaproteobacteria*	*Gammaproteobacteria*	*Proteobacteria*	*Bacteria*
Otu1131	0.4	0	0.13	0.8	0.13	75.76	unclass. *Bacteria*	unclass. *Bacteria*	unclass. *Bacteria*	unclass. *Bacteria*	unclass. *Bacteria*	*Bacteria*
Otu1148	0.4	0	0.13	0.8	0.13	75.89	unclass. *Gammaproteobacteria*	unclass. *Gammaproteobacteria*	unclass. *Gammaproteobacteria*	*Gammaproteobacteria*	*Proteobacteria*	*Bacteria*
Otu1199	0.4	0	0.13	0.8	0.13	76.02	unclass. *Rhodobacteraceae*	*Rhodobacteraceae*	*Rhodobacterales*	*Alphaproteobacteria*	*Proteobacteria*	*Bacteria*
Otu1350	0.4	0	0.12	0.49	0.13	76.15	unclass. *Bacteria*	unclass. *Bacteria*	unclass. *Bacteria*	unclass. *Bacteria*	unclass. *Bacteria*	*Bacteria*
Otu0849	0.35	0	0.12	0.49	0.12	76.27	unclass. *Alphaproteobacteria*	unclass. *Alphaproteobacteria*	unclass. *Alphaproteobacteria*	*Alphaproteobacteria*	*Proteobacteria*	*Bacteria*
Otu1098	0.35	0	0.11	0.49	0.11	76.38	unclass. *Flavobacteriaceae*	*Flavobacteriaceae*	*Flavobacteriales*	*Flavobacteria*	*Bacteroidetes*	*Bacteria*
Otu0821	0.35	0	0.11	0.49	0.11	76.49	unclass. *Bacteria*	unclass. *Bacteria*	unclass. *Bacteria*	unclass. *Bacteria*	unclass. *Bacteria*	*Bacteria*
Otu1030	0.35	0	0.11	0.49	0.11	76.6	unclass. *Proteobacteria*	unclass. *Proteobacteria*	unclass. *Proteobacteria*	unclass. *Proteobacteria*	*Proteobacteria*	*Bacteria*
Otu0040	0.28	0	0.11	0.49	0.11	76.71	*Erythrobacter*	*Erythrobacteraceae*	*Sphingomonadales*	*Alphaproteobacteria*	*Proteobacteria*	*Bacteria*
Otu1283	0.28	0	0.11	0.49	0.11	76.82	*Croceibacter*	*Flavobacteriaceae*	*Flavobacteriales*	*Flavobacteria*	*Bacteroidetes*	*Bacteria*
Otu0140	0.2	0.2	0.1	0.67	0.11	76.93	*Oleispira*	*Oceanospirillaceae*	*Oceanospirillales*	*Gammaproteobacteria*	*Proteobacteria*	*Bacteria*
Otu0142	0.28	0	0.1	0.49	0.1	77.03	unclass. *Gammaproteobacteria*	unclass. *Gammaproteobacteria*	unclass. *Gammaproteobacteria*	*Gammaproteobacteria*	*Proteobacteria*	*Bacteria*
Otu0513	0.28	0	0.1	0.49	0.1	77.13	unclass. *Bacteroidetes*	unclass. *Bacteroidetes*	unclass. *Bacteroidetes*	unclass. *Bacteroidetes*	*Bacteroidetes*	*Bacteria*
Otu0699	0.28	0	0.1	0.49	0.1	77.23	unclass. *Flavobacteriales*	unclass. *Flavobacteriales*	*Flavobacteriales*	*Flavobacteria*	*Bacteroidetes*	*Bacteria*
Otu0739	0.28	0	0.1	0.49	0.1	77.34	unclass. *Proteobacteria*	unclass. *Proteobacteria*	unclass. *Proteobacteria*	unclass. *Proteobacteria*	*Proteobacteria*	*Bacteria*
Otu1076	0.28	0	0.1	0.49	0.1	77.44	*Acinetobacter*	*Moraxellaceae*	*Pseudomonadales*	*Gammaproteobacteria*	*Proteobacteria*	*Bacteria*
Otu1317	0.28	0	0.1	0.49	0.1	77.54	unclass. *Bacteria*	unclass. *Bacteria*	unclass. *Bacteria*	unclass. *Bacteria*	unclass. *Bacteria*	*Bacteria*
Otu1058	0	0.28	0.1	0.49	0.1	77.64	unclass. *Proteobacteria*	unclass. *Proteobacteria*	unclass. *Proteobacteria*	unclass. *Proteobacteria*	*Proteobacteria*	*Bacteria*
Otu0035	0.28	0	0.09	0.49	0.1	77.73	*Colwellia*	*Colwelliaceae*	*Alteromonadales*	*Gammaproteobacteria*	*Proteobacteria*	*Bacteria*
Otu0256	0.28	0	0.09	0.49	0.1	77.83	*Glaciecola*	*Alteromonadaceae*	*Alteromonadales*	*Gammaproteobacteria*	*Proteobacteria*	*Bacteria*
Otu1007	0.28	0	0.09	0.49	0.1	77.93	unclass. *Erythrobacteraceae*	*Erythrobacteraceae*	*Sphingomonadales*	*Alphaproteobacteria*	*Proteobacteria*	*Bacteria*
Otu1233	0.28	0	0.09	0.49	0.1	78.02	unclass. *Flavobacteriaceae*	*Flavobacteriaceae*	*Flavobacteriales*	*Flavobacteria*	*Bacteroidetes*	*Bacteria*
Otu1276	0.28	0	0.09	0.49	0.1	78.12	unclass. *Flammeovirgaceae*	*Flammeovirgaceae*	*Sphingobacteriales*	*Sphingobacteria*	*Bacteroidetes*	*Bacteria*
Otu1134	0.28	0	0.09	0.49	0.09	78.21	unclass. *Gammaproteobacteria*	unclass. *Gammaproteobacteria*	unclass. *Gammaproteobacteria*	*Gammaproteobacteria*	*Proteobacteria*	*Bacteria*
Otu0030	0.28	0	0.09	0.49	0.09	78.3	unclass. *Gammaproteobacteria*	unclass. *Gammaproteobacteria*	unclass. *Gammaproteobacteria*	*Gammaproteobacteria*	*Proteobacteria*	*Bacteria*
Otu0518	0.28	0	0.09	0.49	0.09	78.39	unclass. *Proteobacteria*	unclass. *Proteobacteria*	unclass. *Proteobacteria*	unclass. *Proteobacteria*	*Proteobacteria*	*Bacteria*

OTU	Av.A_i (no)	Av.A_i (serial)	Av.δ_i	Av.δ_i/SD	Av.δ_i %	∑Av.δ_i %	Genus	Family	Order	Class	Phylum	Domain
Otu0724	0.28	0	0.09	0.49	0.09	78.48	unclass. *Bacteria*	unclass. *Bacteria*	unclass. *Bacteria*	unclass. *Bacteria*	unclass. *Bacteria*	*Bacteria*
Otu0734	0.28	0	0.09	0.49	0.09	78.57	unclass. *Bacteria*	unclass. *Bacteria*	unclass. *Bacteria*	unclass. *Bacteria*	unclass. *Bacteria*	*Bacteria*
Otu0832	0.28	0	0.09	0.49	0.09	78.66	unclass. *Rhodospirillaceae*	*Rhodospirillaceae*	*Rhodospirillales*	*Alphaproteobacteria*	*Proteobacteria*	*Bacteria*
Otu1211	0.28	0	0.09	0.49	0.09	78.75	unclass. *Oceanospirillaceae*	*Oceanospirillaceae*	*Oceanospirillales*	*Gammaproteobacteria*	*Proteobacteria*	*Bacteria*
Otu1235	0.28	0	0.09	0.49	0.09	78.84	unclass. *Proteobacteria*	unclass. *Proteobacteria*	unclass. *Proteobacteria*	unclass. *Proteobacteria*	*Proteobacteria*	*Bacteria*
Otu1278	0.28	0	0.09	0.49	0.09	78.93	unclass. *Betaproteobacteria*	unclass. *Betaproteobacteria*	unclass. *Betaproteobacteria*	*Betaproteobacteria*	*Proteobacteria*	*Bacteria*
Otu0042	0.2	0	0.08	0.49	0.08	79	*Rhodococcus*	*Nocardiaceae*	*Actinomycetales*	*Actinobacteria*	*Actinobacteria*	*Bacteria*
Otu0093	0.2	0	0.08	0.49	0.08	79.08	*Aestuariicola*	*Flavobacteriaceae*	*Flavobacteriales*	*Flavobacteria*	*Bacteroidetes*	*Bacteria*
Otu0253	0.2	0	0.08	0.49	0.08	79.16	unclass. *Saprospiraceae*	*Saprospiraceae*	*Sphingobacteriales*	*Sphingobacteria*	*Bacteroidetes*	*Bacteria*
Otu0280	0.2	0	0.08	0.49	0.08	79.23	*Marinobacter*	*Alteromonadaceae*	*Alteromonadales*	*Gammaproteobacteria*	*Proteobacteria*	*Bacteria*
Otu0324	0.2	0	0.08	0.49	0.08	79.31	*Nisaea*	*Rhodospirillaceae*	*Rhodospirillales*	*Alphaproteobacteria*	*Proteobacteria*	*Bacteria*
Otu0558	0.2	0	0.08	0.49	0.08	79.39	unclass. *Gammaproteobacteria*	unclass. *Gammaproteobacteria*	unclass. *Gammaproteobacteria*	*Gammaproteobacteria*	*Proteobacteria*	*Bacteria*
Otu0704	0.2	0	0.08	0.49	0.08	79.47	unclass. *Proteobacteria*	unclass. *Proteobacteria*	unclass. *Proteobacteria*	unclass. *Proteobacteria*	*Proteobacteria*	*Bacteria*
Otu0740	0.2	0	0.08	0.49	0.08	79.54	unclass. *Betaproteobacteria*	unclass. *Betaproteobacteria*	unclass. *Betaproteobacteria*	*Betaproteobacteria*	*Proteobacteria*	*Bacteria*
Otu0928	0.2	0	0.08	0.49	0.08	79.62	*Crocinitomix*	*Cryomorphaceae*	*Flavobacteriales*	*Flavobacteria*	*Bacteroidetes*	*Bacteria*
Otu1202	0.2	0	0.08	0.49	0.08	79.7	unclass. *Gammaproteobacteria*	unclass. *Gammaproteobacteria*	unclass. *Gammaproteobacteria*	*Gammaproteobacteria*	*Proteobacteria*	*Bacteria*
Otu1215	0.2	0	0.08	0.49	0.08	79.77	unclass. *Gammaproteobacteria*	unclass. *Gammaproteobacteria*	unclass. *Gammaproteobacteria*	*Gammaproteobacteria*	*Proteobacteria*	*Bacteria*
Otu1282	0.2	0	0.08	0.49	0.08	79.85	unclass. *Gammaproteobacteria*	unclass. *Gammaproteobacteria*	unclass. *Gammaproteobacteria*	*Gammaproteobacteria*	*Proteobacteria*	*Bacteria*
Otu1284	0.2	0	0.08	0.49	0.08	79.93	unclass. *Bacteroidetes*	unclass. *Bacteroidetes*	unclass. *Bacteroidetes*	unclass. *Bacteroidetes*	*Bacteroidetes*	*Bacteria*
Otu1285	0.2	0	0.08	0.49	0.08	80	unclass. *Myxococcales*	unclass. *Myxococcales*	*Myxococcales*	*Deltaproteobacteria*	*Proteobacteria*	*Bacteria*
Otu1287	0.2	0	0.08	0.49	0.08	80.08	unclass. *Flavobacteriaceae*	*Flavobacteriaceae*	*Flavobacteriales*	*Flavobacteria*	*Bacteroidetes*	*Bacteria*
Otu1288	0.2	0	0.08	0.49	0.08	80.16	unclass. *Bacteria*	unclass. *Bacteria*	unclass. *Bacteria*	unclass. *Bacteria*	unclass. *Bacteria*	*Bacteria*
Otu1289	0.2	0	0.08	0.49	0.08	80.24	unclass. *Bacteroidetes*	unclass. *Bacteroidetes*	unclass. *Bacteroidetes*	unclass. *Bacteroidetes*	*Bacteroidetes*	*Bacteria*
Otu1290	0.2	0	0.08	0.49	0.08	80.31	unclass. *Bacteria*	unclass. *Bacteria*	unclass. *Bacteria*	unclass. *Bacteria*	unclass. *Bacteria*	*Bacteria*
Otu1291	0.2	0	0.08	0.49	0.08	80.39	unclass. *Gammaproteobacteria*	unclass. *Gammaproteobacteria*	unclass. *Gammaproteobacteria*	*Gammaproteobacteria*	*Proteobacteria*	*Bacteria*
Otu1292	0.2	0	0.08	0.49	0.08	80.47	unclass. *Deltaproteobacteria*	unclass. *Deltaproteobacteria*	unclass. *Deltaproteobacteria*	*Deltaproteobacteria*	*Proteobacteria*	*Bacteria*
Otu1293	0.2	0	0.08	0.49	0.08	80.54	unclass. *Bacteroidetes*	unclass. *Bacteroidetes*	unclass. *Bacteroidetes*	unclass. *Bacteroidetes*	*Bacteroidetes*	*Bacteria*

OTU	Av.A_i (no)	Av.A_i (serial)	Av.δ_i	Av.δ_i/SD	Av.δ_i %	∑Av.δ_i %	Genus	Family	Order	Class	Phylum	Domain
Otu1294	0.2	0	0.08	0.49	0.08	80.62	unclass. *Bacteria*	unclass. *Bacteria*	unclass. *Bacteria*	unclass. *Bacteria*	unclass. *Bacteria*	*Bacteria*
Otu1295	0.2	0	0.08	0.49	0.08	80.7	unclass. *Actinomycetales*	unclass. *Actinomycetales*	*Actinomycetales*	*Actinobacteria*	*Actinobacteria*	*Bacteria*
Otu1298	0.2	0	0.08	0.49	0.08	80.78	unclass. *Gammaproteobacteria*	unclass. *Gammaproteobacteria*	unclass. *Gammaproteobacteria*	*Gammaproteobacteria*	*Proteobacteria*	*Bacteria*
Otu1301	0.2	0	0.08	0.49	0.08	80.85	unclass. *Alphaproteobacteria*	unclass. *Alphaproteobacteria*	unclass. *Alphaproteobacteria*	*Alphaproteobacteria*	*Proteobacteria*	*Bacteria*
Otu1302	0.2	0	0.08	0.49	0.08	80.93	unclass. *Bacteroidetes*	unclass. *Bacteroidetes*	unclass. *Bacteroidetes*	unclass. *Bacteroidetes*	*Bacteroidetes*	*Bacteria*
Otu1303	0.2	0	0.08	0.49	0.08	81.01	unclass. *Acidobacteria_Gp17*	unclass. *Acidobacteria_Gp17*	unclass. *Acidobacteria_Gp17*	*Acidobacteria_Gp17*	*Acidobacteria*	*Bacteria*
Otu1304	0.2	0	0.08	0.49	0.08	81.08	unclass. *Bacteria*	unclass. *Bacteria*	unclass. *Bacteria*	unclass. *Bacteria*	unclass. *Bacteria*	*Bacteria*
Otu1305	0.2	0	0.08	0.49	0.08	81.16	unclass. *Sphingobacteriales*	unclass. *Sphingobacteriales*	*Sphingobacteriales*	*Sphingobacteria*	*Bacteroidetes*	*Bacteria*
Otu1306	0.2	0	0.08	0.49	0.08	81.24	unclass. *Gammaproteobacteria*	unclass. *Gammaproteobacteria*	unclass. *Gammaproteobacteria*	*Gammaproteobacteria*	*Proteobacteria*	*Bacteria*
Otu1307	0.2	0	0.08	0.49	0.08	81.31	unclass. *Gammaproteobacteria*	unclass. *Gammaproteobacteria*	unclass. *Gammaproteobacteria*	*Gammaproteobacteria*	*Proteobacteria*	*Bacteria*
Otu1308	0.2	0	0.08	0.49	0.08	81.39	unclass. *Flavobacteriaceae*	*Flavobacteriaceae*	*Flavobacteriales*	*Flavobacteria*	*Bacteroidetes*	*Bacteria*
Otu1309	0.2	0	0.08	0.49	0.08	81.47	unclass. *Gammaproteobacteria*	unclass. *Gammaproteobacteria*	unclass. *Gammaproteobacteria*	*Gammaproteobacteria*	*Proteobacteria*	*Bacteria*
Otu1310	0.2	0	0.08	0.49	0.08	81.55	unclass. *Gammaproteobacteria*	unclass. *Gammaproteobacteria*	unclass. *Gammaproteobacteria*	*Gammaproteobacteria*	*Proteobacteria*	*Bacteria*
Otu1311	0.2	0	0.08	0.49	0.08	81.62	unclass. *Rhodobacteraceae*	*Rhodobacteraceae*	*Rhodobacterales*	*Alphaproteobacteria*	*Proteobacteria*	*Bacteria*
Otu1312	0.2	0	0.08	0.49	0.08	81.7	unclass. *Proteobacteria*	unclass. *Proteobacteria*	unclass. *Proteobacteria*	unclass. *Proteobacteria*	*Proteobacteria*	*Bacteria*
Otu1314	0.2	0	0.08	0.49	0.08	81.78	unclass. *Acidobacteria_Gp22*	unclass. *Acidobacteria_Gp22*	unclass. *Acidobacteria_Gp22*	*Acidobacteria_Gp22*	*Acidobacteria*	*Bacteria*
Otu1316	0.2	0	0.08	0.49	0.08	81.85	unclass. *Gammaproteobacteria*	unclass. *Gammaproteobacteria*	unclass. *Gammaproteobacteria*	*Gammaproteobacteria*	*Proteobacteria*	*Bacteria*
Otu0115	0.2	0	0.07	0.49	0.07	81.93	*Glaciecola*	*Alteromonadaceae*	*Alteromonadales*	*Gammaproteobacteria*	*Proteobacteria*	*Bacteria*
Otu0156	0.2	0	0.07	0.49	0.07	82	unclass. *Gammaproteobacteria*	unclass. *Gammaproteobacteria*	unclass. *Gammaproteobacteria*	*Gammaproteobacteria*	*Proteobacteria*	*Bacteria*
Otu0270	0.2	0	0.07	0.49	0.07	82.07	unclass. *Bacteroidetes*	unclass. *Bacteroidetes*	unclass. *Bacteroidetes*	unclass. *Bacteroidetes*	*Bacteroidetes*	*Bacteria*
Otu0303	0.2	0	0.07	0.49	0.07	82.14	unclass. *Bacteroidetes*	unclass. *Bacteroidetes*	unclass. *Bacteroidetes*	unclass. *Bacteroidetes*	*Bacteroidetes*	*Bacteria*
Otu0349	0.2	0	0.07	0.49	0.07	82.21	unclass. *Gammaproteobacteria*	unclass. *Gammaproteobacteria*	unclass. *Gammaproteobacteria*	*Gammaproteobacteria*	*Proteobacteria*	*Bacteria*
Otu0362	0.2	0	0.07	0.49	0.07	82.29	unclass. *Bacteroidetes*	unclass. *Bacteroidetes*	unclass. *Bacteroidetes*	unclass. *Bacteroidetes*	*Bacteroidetes*	*Bacteria*
Otu0367	0.2	0	0.07	0.49	0.07	82.36	unclass. *Bacteroidetes*	unclass. *Bacteroidetes*	unclass. *Bacteroidetes*	unclass. *Bacteroidetes*	*Bacteroidetes*	*Bacteria*
Otu0370	0.2	0	0.07	0.49	0.07	82.43	unclass. *Gammaproteobacteria*	unclass. *Gammaproteobacteria*	unclass. *Gammaproteobacteria*	*Gammaproteobacteria*	*Proteobacteria*	*Bacteria*
Otu0376	0.2	0	0.07	0.49	0.07	82.5	unclass. *Gammaproteobacteria*	unclass. *Gammaproteobacteria*	unclass. *Gammaproteobacteria*	*Gammaproteobacteria*	*Proteobacteria*	*Bacteria*
Otu0394	0.2	0	0.07	0.49	0.07	82.57	unclass. *Bacteria*	unclass. *Bacteria*	unclass. *Bacteria*	unclass. *Bacteria*	unclass. *Bacteria*	*Bacteria*

OTU	Av.A_i (no)	Av.A_i (serial)	Av.δ_i	Av.δ_i/SD	Av.δ_i %	∑Av.δ_i %	Genus	Family	Order	Class	Phylum	Domain
Otu0732	0.2	0	0.07	0.49	0.07	82.64	unclass. *Proteobacteria*	unclass. *Proteobacteria*	unclass. *Proteobacteria*	unclass. *Proteobacteria*	*Proteobacteria*	*Bacteria*
Otu0772	0.2	0	0.07	0.49	0.07	82.72	unclass. *Alphaproteobacteria*	unclass. *Alphaproteobacteria*	unclass. *Alphaproteobacteria*	*Alphaproteobacteria*	*Proteobacteria*	*Bacteria*
Otu0808	0.2	0	0.07	0.49	0.07	82.79	unclass. *Saprospiraceae*	*Saprospiraceae*	*Sphingobacteriales*	*Sphingobacteria*	*Bacteroidetes*	*Bacteria*
Otu0892	0.2	0	0.07	0.49	0.07	82.86	unclass. *Bacteroidetes*	unclass. *Bacteroidetes*	unclass. *Bacteroidetes*	unclass. *Bacteroidetes*	*Bacteroidetes*	*Bacteria*
Otu0933	0.2	0	0.07	0.49	0.07	82.93	*Haliea*	*Alteromonadaceae*	*Alteromonadales*	*Gammaproteobacteria*	*Proteobacteria*	*Bacteria*
Otu1001	0.2	0	0.07	0.49	0.07	83	unclass. *Flavobacteriales*	unclass. *Flavobacteriales*	*Flavobacteriales*	*Flavobacteria*	*Bacteroidetes*	*Bacteria*
Otu1127	0.2	0	0.07	0.49	0.07	83.08	unclass. *Flavobacteriales*	unclass. *Flavobacteriales*	*Flavobacteriales*	*Flavobacteria*	*Bacteroidetes*	*Bacteria*
Otu1146	0.2	0	0.07	0.49	0.07	83.15	*Nitrosospira*	*Nitrosomonadaceae*	*Nitrosomonadales*	*Betaproteobacteria*	*Proteobacteria*	*Bacteria*
Otu1170	0.2	0	0.07	0.49	0.07	83.22	unclass. *Bacteria*	unclass. *Bacteria*	unclass. *Bacteria*	unclass. *Bacteria*	unclass. *Bacteria*	*Bacteria*
Otu1195	0.2	0	0.07	0.49	0.07	83.29	unclass. *Bacteria*	unclass. *Bacteria*	unclass. *Bacteria*	unclass. *Bacteria*	unclass. *Bacteria*	*Bacteria*
Otu1198	0.2	0	0.07	0.49	0.07	83.36	unclass. *Gammaproteobacteria*	unclass. *Gammaproteobacteria*	unclass. *Gammaproteobacteria*	*Gammaproteobacteria*	*Proteobacteria*	*Bacteria*
Otu1237	0.2	0	0.07	0.49	0.07	83.43	unclass. *Gammaproteobacteria*	unclass. *Gammaproteobacteria*	unclass. *Gammaproteobacteria*	*Gammaproteobacteria*	*Proteobacteria*	*Bacteria*
Otu1286	0.2	0	0.07	0.49	0.07	83.51	unclass. *Gammaproteobacteria*	unclass. *Gammaproteobacteria*	unclass. *Gammaproteobacteria*	*Gammaproteobacteria*	*Proteobacteria*	*Bacteria*
Otu1318	0.2	0	0.07	0.49	0.07	83.58	unclass. *Bacteroidetes*	unclass. *Bacteroidetes*	unclass. *Bacteroidetes*	unclass. *Bacteroidetes*	*Bacteroidetes*	*Bacteria*
Otu1319	0.2	0	0.07	0.49	0.07	83.65	unclass. *Gammaproteobacteria*	unclass. *Gammaproteobacteria*	unclass. *Gammaproteobacteria*	*Gammaproteobacteria*	*Proteobacteria*	*Bacteria*
Otu1320	0.2	0	0.07	0.49	0.07	83.72	unclass. *Bacteria*	unclass. *Bacteria*	unclass. *Bacteria*	unclass. *Bacteria*	unclass. *Bacteria*	*Bacteria*
Otu1321	0.2	0	0.07	0.49	0.07	83.79	unclass. *Flavobacteriaceae*	*Flavobacteriaceae*	*Flavobacteriales*	*Flavobacteria*	*Bacteroidetes*	*Bacteria*
Otu1322	0.2	0	0.07	0.49	0.07	83.87	unclass. *Bacteria*	unclass. *Bacteria*	unclass. *Bacteria*	unclass. *Bacteria*	unclass. *Bacteria*	*Bacteria*
Otu1323	0.2	0	0.07	0.49	0.07	83.94	unclass. *Oceanospirillaceae*	*Oceanospirillaceae*	*Oceanospirillales*	*Gammaproteobacteria*	*Proteobacteria*	*Bacteria*
Otu1324	0.2	0	0.07	0.49	0.07	84.01	unclass. *Alphaproteobacteria*	unclass. *Alphaproteobacteria*	unclass. *Alphaproteobacteria*	*Alphaproteobacteria*	*Proteobacteria*	*Bacteria*
Otu1325	0.2	0	0.07	0.49	0.07	84.08	*Algibacter*	*Flavobacteriaceae*	*Flavobacteriales*	*Flavobacteria*	*Bacteroidetes*	*Bacteria*
Otu1326	0.2	0	0.07	0.49	0.07	84.15	unclass. *Gammaproteobacteria*	unclass. *Gammaproteobacteria*	unclass. *Gammaproteobacteria*	*Gammaproteobacteria*	*Proteobacteria*	*Bacteria*
Otu1327	0.2	0	0.07	0.49	0.07	84.23	unclass. *Gammaproteobacteria*	unclass. *Gammaproteobacteria*	unclass. *Gammaproteobacteria*	*Gammaproteobacteria*	*Proteobacteria*	*Bacteria*
Otu1329	0.2	0	0.07	0.49	0.07	84.3	unclass. *Gammaproteobacteria*	unclass. *Gammaproteobacteria*	unclass. *Gammaproteobacteria*	*Gammaproteobacteria*	*Proteobacteria*	*Bacteria*
Otu1330	0.2	0	0.07	0.49	0.07	84.37	unclass. *Bacteria*	unclass. *Bacteria*	unclass. *Bacteria*	unclass. *Bacteria*	unclass. *Bacteria*	*Bacteria*
Otu1331	0.2	0	0.07	0.49	0.07	84.44	unclass. *Saprospiraceae*	*Saprospiraceae*	*Sphingobacteriales*	*Sphingobacteria*	*Bacteroidetes*	*Bacteria*
Otu1334	0.2	0	0.07	0.49	0.07	84.51	unclass. *Gammaproteobacteria*	unclass. *Gammaproteobacteria*	unclass. *Gammaproteobacteria*	*Gammaproteobacteria*	*Proteobacteria*	*Bacteria*

OTU	Av.A_i (no)	Av.A_i (serial)	Av.δ_i	Av.δ_i/SD	Av.δ_i %	∑Av.δ_i %	Genus	Family	Order	Class	Phylum	Domain
Otu1335	0.2	0	0.07	0.49	0.07	84.58	unclass. *Chromatiales*	unclass. *Chromatiales*	*Chromatiales*	*Gammaproteobacteria*	*Proteobacteria*	*Bacteria*
Otu1336	0.2	0	0.07	0.49	0.07	84.66	unclass. *Bacteria*	unclass. *Bacteria*	unclass. *Bacteria*	unclass. *Bacteria*	unclass. *Bacteria*	*Bacteria*
Otu1337	0.2	0	0.07	0.49	0.07	84.73	unclass. *Bacteria*	unclass. *Bacteria*	unclass. *Bacteria*	unclass. *Bacteria*	unclass. *Bacteria*	*Bacteria*
Otu1338	0.2	0	0.07	0.49	0.07	84.8	unclass. *Bacteroidetes*	unclass. *Bacteroidetes*	unclass. *Bacteroidetes*	unclass. *Bacteroidetes*	*Bacteroidetes*	*Bacteria*
Otu1339	0.2	0	0.07	0.49	0.07	84.87	unclass. *Bacteria*	unclass. *Bacteria*	unclass. *Bacteria*	unclass. *Bacteria*	unclass. *Bacteria*	*Bacteria*
Otu1340	0.2	0	0.07	0.49	0.07	84.94	unclass. *Proteobacteria*	unclass. *Proteobacteria*	unclass. *Proteobacteria*	unclass. *Proteobacteria*	*Proteobacteria*	*Bacteria*
Otu1341	0.2	0	0.07	0.49	0.07	85.02	unclass. *Gammaproteobacteria*	unclass. *Gammaproteobacteria*	unclass. *Gammaproteobacteria*	*Gammaproteobacteria*	*Proteobacteria*	*Bacteria*
Otu1342	0.2	0	0.07	0.49	0.07	85.09	unclass. *Actinobacteria*	unclass. *Actinobacteria*	unclass. *Actinobacteria*	*Actinobacteria*	*Actinobacteria*	*Bacteria*
Otu1344	0.2	0	0.07	0.49	0.07	85.16	unclass. *Gammaproteobacteria*	unclass. *Gammaproteobacteria*	unclass. *Gammaproteobacteria*	*Gammaproteobacteria*	*Proteobacteria*	*Bacteria*
Otu1345	0.2	0	0.07	0.49	0.07	85.23	unclass. *Bacteria*	unclass. *Bacteria*	unclass. *Bacteria*	unclass. *Bacteria*	unclass. *Bacteria*	*Bacteria*
Otu1346	0.2	0	0.07	0.49	0.07	85.3	unclass. *Planctomycetaceae*	*Planctomycetaceae*	*Planctomycetales*	*Planctomycetacia*	*Planctomycetes*	*Bacteria*
Otu1347	0.2	0	0.07	0.49	0.07	85.37	unclass. *Gammaproteobacteria*	unclass. *Gammaproteobacteria*	unclass. *Gammaproteobacteria*	*Gammaproteobacteria*	*Proteobacteria*	*Bacteria*
Otu1349	0.2	0	0.07	0.49	0.07	85.45	unclass. *Bacteria*	unclass. *Bacteria*	unclass. *Bacteria*	unclass. *Bacteria*	unclass. *Bacteria*	*Bacteria*
Otu1066	0	0.2	0.07	0.49	0.07	85.52	unclass. *Bacteria*	unclass. *Bacteria*	unclass. *Bacteria*	unclass. *Bacteria*	unclass. *Bacteria*	*Bacteria*
Otu0538	0	0.2	0.07	0.49	0.07	85.59	*Oceaniserpentilla*	*Oceanospirillaceae*	*Oceanospirillales*	*Gammaproteobacteria*	*Proteobacteria*	*Bacteria*
Otu0165	0	0.2	0.07	0.49	0.07	85.66	*Marinomonas*	*Oceanospirillaceae*	*Oceanospirillales*	*Gammaproteobacteria*	*Proteobacteria*	*Bacteria*
Otu0322	0	0.2	0.07	0.49	0.07	85.73	unclass. *Bacteria*	unclass. *Bacteria*	unclass. *Bacteria*	unclass. *Bacteria*	unclass. *Bacteria*	*Bacteria*
Otu1059	0	0.2	0.07	0.49	0.07	85.8	unclass. *Bacteria*	unclass. *Bacteria*	unclass. *Bacteria*	unclass. *Bacteria*	unclass. *Bacteria*	*Bacteria*
Otu1068	0	0.2	0.07	0.49	0.07	85.87	unclass. *Bacteria*	unclass. *Bacteria*	unclass. *Bacteria*	unclass. *Bacteria*	unclass. *Bacteria*	*Bacteria*
Otu0077	0.2	0	0.07	0.49	0.07	85.93	unclass. *Bacteria*	unclass. *Bacteria*	unclass. *Bacteria*	unclass. *Bacteria*	unclass. *Bacteria*	*Bacteria*
Otu0078	0.2	0	0.07	0.49	0.07	86	*Erythrobacter*	*Erythrobacteraceae*	*Sphingomonadales*	*Alphaproteobacteria*	*Proteobacteria*	*Bacteria*
Otu0196	0.2	0	0.07	0.49	0.07	86.07	unclass. *Flavobacteriaceae*	*Flavobacteriaceae*	*Flavobacteriales*	*Flavobacteria*	*Bacteroidetes*	*Bacteria*
Otu0239	0.2	0	0.07	0.49	0.07	86.14	unclass. *Gammaproteobacteria*	unclass. *Gammaproteobacteria*	unclass. *Gammaproteobacteria*	*Gammaproteobacteria*	*Proteobacteria*	*Bacteria*
Otu0297	0.2	0	0.07	0.49	0.07	86.21	*Lutibacter*	*Flavobacteriaceae*	*Flavobacteriales*	*Flavobacteria*	*Bacteroidetes*	*Bacteria*
Otu0304	0.2	0	0.07	0.49	0.07	86.27	unclass. *Bacteria*	unclass. *Bacteria*	unclass. *Bacteria*	unclass. *Bacteria*	unclass. *Bacteria*	*Bacteria*
Otu0316	0.2	0	0.07	0.49	0.07	86.34	unclass. *Bacteria*	unclass. *Bacteria*	unclass. *Bacteria*	unclass. *Bacteria*	unclass. *Bacteria*	*Bacteria*
Otu0332	0.2	0	0.07	0.49	0.07	86.41	unclass. *Gammaproteobacteria*	unclass. *Gammaproteobacteria*	unclass. *Gammaproteobacteria*	*Gammaproteobacteria*	*Proteobacteria*	*Bacteria*

OTU	Av.A_i (no)	Av.A_i (serial)	Av.δ_i	Av.δ_i/SD	Av.δ_i %	∑Av.δ_i %	Genus	Family	Order	Class	Phylum	Domain
Otu0342	0.2	0	0.07	0.49	0.07	86.48	unclass. *Gammaproteobacteria*	unclass. *Gammaproteobacteria*	unclass. *Gammaproteobacteria*	*Gammaproteobacteria*	*Proteobacteria*	*Bacteria*
Otu0373	0.2	0	0.07	0.49	0.07	86.54	unclass. *Bacteria*	unclass. *Bacteria*	unclass. *Bacteria*	unclass. *Bacteria*	unclass. *Bacteria*	*Bacteria*
Otu0378	0.2	0	0.07	0.49	0.07	86.61	unclass. *Rickettsiaceae*	*Rickettsiaceae*	*Rickettsiales*	*Alphaproteobacteria*	*Proteobacteria*	*Bacteria*
Otu0403	0.2	0	0.07	0.49	0.07	86.68	unclass. *Acidobacteria_Gp10*	unclass. *Acidobacteria_Gp10*	unclass. *Acidobacteria_Gp10*	*Acidobacteria_Gp10*	*Acidobacteria*	*Bacteria*
Otu0431	0.2	0	0.07	0.49	0.07	86.75	unclass. *Flammeovirgaceae*	*Flammeovirgaceae*	*Sphingobacteriales*	*Sphingobacteria*	*Bacteroidetes*	*Bacteria*
Otu0491	0.2	0	0.07	0.49	0.07	86.82	unclass. *Flavobacteriales*	unclass. *Flavobacteriales*	*Flavobacteriales*	*Flavobacteria*	*Bacteroidetes*	*Bacteria*
Otu0582	0.2	0	0.07	0.49	0.07	86.88	unclass. *Rhodobacteraceae*	*Rhodobacteraceae*	*Rhodobacterales*	*Alphaproteobacteria*	*Proteobacteria*	*Bacteria*
Otu0651	0.2	0	0.07	0.49	0.07	86.95	*Arcobacter*	*Campylobacteraceae*	*Campylobacterales*	*Epsilonproteobacteria*	*Proteobacteria*	*Bacteria*
Otu0709	0.2	0	0.07	0.49	0.07	87.02	unclass. *Bacteria*	unclass. *Bacteria*	unclass. *Bacteria*	unclass. *Bacteria*	unclass. *Bacteria*	*Bacteria*
Otu0795	0.2	0	0.07	0.49	0.07	87.09	unclass. *Bacteria*	unclass. *Bacteria*	unclass. *Bacteria*	unclass. *Bacteria*	unclass. *Bacteria*	*Bacteria*
Otu0855	0.2	0	0.07	0.49	0.07	87.16	*Dasania*	*Pseudomonadales_incertae_sedis*	*Pseudomonadales*	*Gammaproteobacteria*	*Proteobacteria*	*Bacteria*
Otu0936	0.2	0	0.07	0.49	0.07	87.22	unclass. *Gammaproteobacteria*	unclass. *Gammaproteobacteria*	unclass. *Gammaproteobacteria*	*Gammaproteobacteria*	*Proteobacteria*	*Bacteria*
Otu0951	0.2	0	0.07	0.49	0.07	87.29	*Haliea*	*Alteromonadaceae*	*Alteromonadales*	*Gammaproteobacteria*	*Proteobacteria*	*Bacteria*
Otu1022	0.2	0	0.07	0.49	0.07	87.36	unclass. *Gammaproteobacteria*	unclass. *Gammaproteobacteria*	unclass. *Gammaproteobacteria*	*Gammaproteobacteria*	*Proteobacteria*	*Bacteria*
Otu1120	0.2	0	0.07	0.49	0.07	87.43	unclass. *Bacteria*	unclass. *Bacteria*	unclass. *Bacteria*	unclass. *Bacteria*	unclass. *Bacteria*	*Bacteria*
Otu1132	0.2	0	0.07	0.49	0.07	87.5	unclass. *Flavobacteriales*	unclass. *Flavobacteriales*	*Flavobacteriales*	*Flavobacteria*	*Bacteroidetes*	*Bacteria*
Otu1163	0.2	0	0.07	0.49	0.07	87.56	unclass. *Bacteria*	unclass. *Bacteria*	unclass. *Bacteria*	unclass. *Bacteria*	unclass. *Bacteria*	*Bacteria*
Otu1177	0.2	0	0.07	0.49	0.07	87.63	unclass. *Erythrobacteraceae*	*Erythrobacteraceae*	*Sphingomonadales*	*Alphaproteobacteria*	*Proteobacteria*	*Bacteria*
Otu1190	0.2	0	0.07	0.49	0.07	87.7	unclass. *Gammaproteobacteria*	unclass. *Gammaproteobacteria*	unclass. *Gammaproteobacteria*	*Gammaproteobacteria*	*Proteobacteria*	*Bacteria*
Otu1218	0.2	0	0.07	0.49	0.07	87.77	unclass. *Bacteria*	unclass. *Bacteria*	unclass. *Bacteria*	unclass. *Bacteria*	unclass. *Bacteria*	*Bacteria*
Otu1220	0.2	0	0.07	0.49	0.07	87.84	unclass. *Bacteria*	unclass. *Bacteria*	unclass. *Bacteria*	unclass. *Bacteria*	unclass. *Bacteria*	*Bacteria*
Otu1221	0.2	0	0.07	0.49	0.07	87.9	unclass. *Flavobacteriales*	unclass. *Flavobacteriales*	*Flavobacteriales*	*Flavobacteria*	*Bacteroidetes*	*Bacteria*
Otu1222	0.2	0	0.07	0.49	0.07	87.97	unclass. *Flavobacteriaceae*	*Flavobacteriaceae*	*Flavobacteriales*	*Flavobacteria*	*Bacteroidetes*	*Bacteria*
Otu1225	0.2	0	0.07	0.49	0.07	88.04	unclass. *Proteobacteria*	unclass. *Proteobacteria*	unclass. *Proteobacteria*	unclass. *Proteobacteria*	*Proteobacteria*	*Bacteria*
Otu1227	0.2	0	0.07	0.49	0.07	88.11	unclass. *Bacteria*	unclass. *Bacteria*	unclass. *Bacteria*	unclass. *Bacteria*	unclass. *Bacteria*	*Bacteria*
Otu1230	0.2	0	0.07	0.49	0.07	88.18	unclass. *Bacteroidetes*	unclass. *Bacteroidetes*	unclass. *Bacteroidetes*	unclass. *Bacteroidetes*	*Bacteroidetes*	*Bacteria*
Otu1232	0.2	0	0.07	0.49	0.07	88.24	*Haliea*	*Alteromonadaceae*	*Alteromonadales*	*Gammaproteobacteria*	*Proteobacteria*	*Bacteria*

OTU	Av.A_i (no)	Av.A_i (serial)	Av.δ_i	Av.δ_i/SD	Av.δ_i %	∑Av.δ_i %	Genus	Family	Order	Class	Phylum	Domain
Otu1239	0.2	0	0.07	0.49	0.07	88.31	*Sneathiella*	*Sneathiellaceae*	*Sneathiellales*	*Alphaproteobacteria*	*Proteobacteria*	*Bacteria*
Otu1240	0.2	0	0.07	0.49	0.07	88.38	unclass. *Gammaproteobacteria*	unclass. *Gammaproteobacteria*	unclass. *Gammaproteobacteria*	*Gammaproteobacteria*	*Proteobacteria*	*Bacteria*
Otu1241	0.2	0	0.07	0.49	0.07	88.45	unclass. *Gammaproteobacteria*	unclass. *Gammaproteobacteria*	unclass. *Gammaproteobacteria*	*Gammaproteobacteria*	*Proteobacteria*	*Bacteria*
Otu1243	0.2	0	0.07	0.49	0.07	88.52	unclass. *Bacteria*	unclass. *Bacteria*	unclass. *Bacteria*	unclass. *Bacteria*	unclass. *Bacteria*	*Bacteria*
Otu1244	0.2	0	0.07	0.49	0.07	88.58	unclass. *Bacteria*	unclass. *Bacteria*	unclass. *Bacteria*	unclass. *Bacteria*	unclass. *Bacteria*	*Bacteria*
Otu1246	0.2	0	0.07	0.49	0.07	88.65	unclass. *Gammaproteobacteria*	unclass. *Gammaproteobacteria*	unclass. *Gammaproteobacteria*	*Gammaproteobacteria*	*Proteobacteria*	*Bacteria*
Otu1248	0.2	0	0.07	0.49	0.07	88.72	unclass. *Bacteria*	unclass. *Bacteria*	unclass. *Bacteria*	unclass. *Bacteria*	unclass. *Bacteria*	*Bacteria*
Otu1250	0.2	0	0.07	0.49	0.07	88.79	unclass. *Bacteria*	unclass. *Bacteria*	unclass. *Bacteria*	unclass. *Bacteria*	unclass. *Bacteria*	*Bacteria*
Otu1251	0.2	0	0.07	0.49	0.07	88.86	unclass. *Bacteria*	unclass. *Bacteria*	unclass. *Bacteria*	unclass. *Bacteria*	unclass. *Bacteria*	*Bacteria*
Otu1252	0.2	0	0.07	0.49	0.07	88.92	unclass. *Flavobacteriaceae*	*Flavobacteriaceae*	*Flavobacteriales*	*Flavobacteria*	*Bacteroidetes*	*Bacteria*
Otu1253	0.2	0	0.07	0.49	0.07	88.99	unclass. *Proteobacteria*	unclass. *Proteobacteria*	unclass. *Proteobacteria*	unclass. *Proteobacteria*	*Proteobacteria*	*Bacteria*
Otu1254	0.2	0	0.07	0.49	0.07	89.06	unclass. *Gammaproteobacteria*	unclass. *Gammaproteobacteria*	unclass. *Gammaproteobacteria*	*Gammaproteobacteria*	*Proteobacteria*	*Bacteria*
Otu1257	0.2	0	0.07	0.49	0.07	89.13	unclass. *Gammaproteobacteria*	unclass. *Gammaproteobacteria*	unclass. *Gammaproteobacteria*	*Gammaproteobacteria*	*Proteobacteria*	*Bacteria*
Otu1258	0.2	0	0.07	0.49	0.07	89.19	*Amphritea*	*Oceanospirillaceae*	*Oceanospirillales*	*Gammaproteobacteria*	*Proteobacteria*	*Bacteria*
Otu1259	0.2	0	0.07	0.49	0.07	89.26	unclass. *Nannocystaceae*	*Nannocystaceae*	*Myxococcales*	*Deltaproteobacteria*	*Proteobacteria*	*Bacteria*
Otu1260	0.2	0	0.07	0.49	0.07	89.33	*Marinobacterium*	*Alteromonadaceae*	*Alteromonadales*	*Gammaproteobacteria*	*Proteobacteria*	*Bacteria*
Otu1261	0.2	0	0.07	0.49	0.07	89.4	unclass. *Gammaproteobacteria*	unclass. *Gammaproteobacteria*	unclass. *Gammaproteobacteria*	*Gammaproteobacteria*	*Proteobacteria*	*Bacteria*
Otu1262	0.2	0	0.07	0.49	0.07	89.47	unclass. *Bacteroidetes*	unclass. *Bacteroidetes*	unclass. *Bacteroidetes*	unclass. *Bacteroidetes*	*Bacteroidetes*	*Bacteria*
Otu1263	0.2	0	0.07	0.49	0.07	89.53	unclass. *Gammaproteobacteria*	unclass. *Gammaproteobacteria*	unclass. *Gammaproteobacteria*	*Gammaproteobacteria*	*Proteobacteria*	*Bacteria*
Otu1264	0.2	0	0.07	0.49	0.07	89.6	unclass. *Bacteria*	unclass. *Bacteria*	unclass. *Bacteria*	unclass. *Bacteria*	unclass. *Bacteria*	*Bacteria*
Otu1265	0.2	0	0.07	0.49	0.07	89.67	unclass. *Bacteria*	unclass. *Bacteria*	unclass. *Bacteria*	unclass. *Bacteria*	unclass. *Bacteria*	*Bacteria*
Otu1266	0.2	0	0.07	0.49	0.07	89.74	unclass. *Gammaproteobacteria*	unclass. *Gammaproteobacteria*	unclass. *Gammaproteobacteria*	*Gammaproteobacteria*	*Proteobacteria*	*Bacteria*
Otu1269	0.2	0	0.07	0.49	0.07	89.81	unclass. *Bacteria*	unclass. *Bacteria*	unclass. *Bacteria*	unclass. *Bacteria*	unclass. *Bacteria*	*Bacteria*
Otu1271	0.2	0	0.07	0.49	0.07	89.87	unclass. *Alteromonadales*	unclass. *Alteromonadales*	*Alteromonadales*	*Gammaproteobacteria*	*Proteobacteria*	*Bacteria*
Otu1272	0.2	0	0.07	0.49	0.07	89.94	unclass. *Bacteria*	unclass. *Bacteria*	unclass. *Bacteria*	unclass. *Bacteria*	unclass. *Bacteria*	*Bacteria*
Otu1275	0.2	0	0.07	0.49	0.07	90.01	unclass. *Flavobacteriaceae*	*Flavobacteriaceae*	*Flavobacteriales*	*Flavobacteria*	*Bacteroidetes*	*Bacteria*

Winter pH *in situ* 'no dilution' vs. 'serial dilution' (average dissimilarity: 92.4%)

OTU	Av.A_i (no)	Av.A_i (serial)	Av.δ_i	Av.δ_i/SD	Av.δ_i %	∑Av.δ_i %	Genus	Family	Order	Class	Phylum	Domain
Otu0106	0	12.27	4.36	6.29	4.72	4.72	*Marinomonas*	*Oceanospirillaceae*	*Oceanospirillales*	*Gammaproteobacteria*	*Proteobacteria*	*Bacteria*
Otu0132	0	8.64	3.07	4.38	3.32	8.04	unclass. *Alteromonadales*	unclass. *Alteromonadales*	*Alteromonadales*	*Gammaproteobacteria*	*Proteobacteria*	*Bacteria*
Otu0010	7.33	0.97	2.26	6.92	2.45	10.49	unclass. *Flavobacteriaceae*	*Flavobacteriaceae*	*Flavobacteriales*	*Flavobacteria*	*Bacteroidetes*	*Bacteria*
Otu0055	3.73	9.17	2.14	2.23	2.32	12.8	*Colwellia*	*Colwelliaceae*	*Alteromonadales*	*Gammaproteobacteria*	*Proteobacteria*	*Bacteria*
Otu0123	0	5.62	2	1.74	2.17	14.97	unclass. *Gammaproteobacteria*	unclass. *Gammaproteobacteria*	unclass. *Gammaproteobacteria*	*Gammaproteobacteria*	*Proteobacteria*	*Bacteria*
Otu1815	5.57	0	1.98	4.08	2.14	17.11	unclass. *Flavobacteriales*	unclass. *Flavobacteriales*	*Flavobacteriales*	*Flavobacteria*	*Bacteroidetes*	*Bacteria*
Otu1777	0	5.49	1.95	5.16	2.11	19.22	*Oceaniserpentilla*	*Oceanospirillaceae*	*Oceanospirillales*	*Gammaproteobacteria*	*Proteobacteria*	*Bacteria*
Otu0041	4.85	0.2	1.66	3.24	1.8	21.01	*Sulfitobacter*	*Rhodobacteraceae*	*Rhodobacterales*	*Alphaproteobacteria*	*Proteobacteria*	*Bacteria*
Otu0134	4.57	0	1.62	5.26	1.75	22.77	unclass. *Gammaproteobacteria*	unclass. *Gammaproteobacteria*	unclass. *Gammaproteobacteria*	*Gammaproteobacteria*	*Proteobacteria*	*Bacteria*
Otu1283	4.37	0	1.55	3.99	1.68	24.45	*Croceibacter*	*Flavobacteriaceae*	*Flavobacteriales*	*Flavobacteria*	*Bacteroidetes*	*Bacteria*
Otu0471	0	4.28	1.52	2.93	1.64	26.09	*Oceaniserpentilla*	*Oceanospirillaceae*	*Oceanospirillales*	*Gammaproteobacteria*	*Proteobacteria*	*Bacteria*
Otu0139	4.22	0	1.51	1.75	1.63	27.72	unclass. *Gammaproteobacteria*	unclass. *Gammaproteobacteria*	unclass. *Gammaproteobacteria*	*Gammaproteobacteria*	*Proteobacteria*	*Bacteria*
Otu0236	4.16	0	1.47	9.37	1.6	29.32	unclass. *Gammaproteobacteria*	unclass. *Gammaproteobacteria*	unclass. *Gammaproteobacteria*	*Gammaproteobacteria*	*Proteobacteria*	*Bacteria*
Otu0068	3.4	0	1.21	4.31	1.31	30.62	*Pelagibacter*	SAR11-clade	*Rickettsiales*	*Alphaproteobacteria*	*Proteobacteria*	*Bacteria*
Otu1694	2.94	0	1.04	2.91	1.13	31.75	unclass. *Flavobacteriales*	unclass. *Flavobacteriales*	*Flavobacteriales*	*Flavobacteria*	*Bacteroidetes*	*Bacteria*
Otu0160	3.51	5.46	0.93	1.46	1.01	32.76	*Colwellia*	*Colwelliaceae*	*Alteromonadales*	*Gammaproteobacteria*	*Proteobacteria*	*Bacteria*
Otu0047	2.51	0	0.9	3.23	0.97	33.73	unclass. *Rhodobacteraceae*	*Rhodobacteraceae*	*Rhodobacterales*	*Alphaproteobacteria*	*Proteobacteria*	*Bacteria*
Otu1822	2.52	0	0.89	3.21	0.97	34.7	unclass. *Flavobacteriales*	unclass. *Flavobacteriales*	*Flavobacteriales*	*Flavobacteria*	*Bacteroidetes*	*Bacteria*
Otu0005	2.51	0	0.89	2.98	0.96	35.66	*Pelagibacter*	SAR11-clade	*Rickettsiales*	*Alphaproteobacteria*	*Proteobacteria*	*Bacteria*
Otu0409	2.38	0	0.83	0.66	0.9	36.56	unclass. *Flavobacteriales*	unclass. *Flavobacteriales*	*Flavobacteriales*	*Flavobacteria*	*Bacteroidetes*	*Bacteria*
Otu0572	2.33	0	0.82	1.11	0.89	37.45	*Croceibacter*	*Flavobacteriaceae*	*Flavobacteriales*	*Flavobacteria*	*Bacteroidetes*	*Bacteria*
Otu0571	0	1.95	0.69	4.28	0.75	38.2	*Marinomonas*	*Oceanospirillaceae*	*Oceanospirillales*	*Gammaproteobacteria*	*Proteobacteria*	*Bacteria*
Otu0164	1.94	0	0.69	3.76	0.75	38.94	*Croceibacter*	*Flavobacteriaceae*	*Flavobacteriales*	*Flavobacteria*	*Bacteroidetes*	*Bacteria*
Otu0113	1.91	0	0.69	1.58	0.74	39.68	*Colwellia*	*Colwelliaceae*	*Alteromonadales*	*Gammaproteobacteria*	*Proteobacteria*	*Bacteria*
Otu0469	1.92	0	0.68	5.49	0.74	40.42	unclass. *Bacteria*	unclass. *Bacteria*	unclass. *Bacteria*	unclass. *Bacteria*	unclass. *Bacteria*	*Bacteria*
Otu0001	1.91	0	0.68	3.07	0.73	41.15	unclass. *Flavobacteriaceae*	*Flavobacteriaceae*	*Flavobacteriales*	*Flavobacteria*	*Bacteroidetes*	*Bacteria*
Otu1720	1.86	0	0.66	2.91	0.72	41.87	unclass. *Bacteria*	unclass. *Bacteria*	unclass. *Bacteria*	unclass. *Bacteria*	unclass. *Bacteria*	*Bacteria*

OTU	$Av.A_i$ (no)	$Av.A_i$ (serial)	$Av.\delta_i$	$Av.\delta_i/SD$	$Av.\delta_i$ %	$\sum Av.\delta_i$ %	Genus	Family	Order	Class	Phylum	Domain
Otu0163	1.87	0	0.66	3.43	0.71	42.59	unclass. *Gammaproteobacteria*	unclass. *Gammaproteobacteria*	unclass. *Gammaproteobacteria*	*Gammaproteobacteria*	*Proteobacteria*	*Bacteria*
Otu0032	1.79	0	0.64	4.25	0.69	43.27	unclass. *Gammaproteobacteria*	unclass. *Gammaproteobacteria*	unclass. *Gammaproteobacteria*	*Gammaproteobacteria*	*Proteobacteria*	*Bacteria*
Otu0157	1.75	0	0.62	4.82	0.67	43.94	unclass. *Gammaproteobacteria*	unclass. *Gammaproteobacteria*	unclass. *Gammaproteobacteria*	*Gammaproteobacteria*	*Proteobacteria*	*Bacteria*
Otu0122	1.58	0	0.56	1.62	0.61	44.55	*Glaciecola*	*Alteromonadaceae*	*Alteromonadales*	*Gammaproteobacteria*	*Proteobacteria*	*Bacteria*
Otu1146	1.58	0	0.56	4.72	0.6	45.15	*Nitrosospira*	*Nitrosomonadaceae*	*Nitrosomonadales*	*Betaproteobacteria*	*Proteobacteria*	*Bacteria*
Otu0270	1.55	0	0.55	3.41	0.59	45.75	unclass. *Bacteroidetes*	unclass. *Bacteroidetes*	unclass. *Bacteroidetes*	unclass. *Bacteroidetes*	*Bacteroidetes*	*Bacteria*
Otu0209	0	1.54	0.54	2.33	0.59	46.33	unclass. *Oceanospirillales*	unclass. *Oceanospirillales*	*Oceanospirillales*	*Gammaproteobacteria*	*Proteobacteria*	*Bacteria*
Otu0124	1.51	0	0.54	4.37	0.58	46.92	*Loktanella*	*Rhodobacteraceae*	*Rhodobacterales*	*Alphaproteobacteria*	*Proteobacteria*	*Bacteria*
Otu0261	1.51	0	0.53	1.57	0.58	47.49	*Winogradskyella*	*Flavobacteriaceae*	*Flavobacteriales*	*Flavobacteria*	*Bacteroidetes*	*Bacteria*
Otu0012	1.49	0	0.53	2.42	0.57	48.07	unclass. *Comamonadaceae*	*Comamonadaceae*	*Burkholderiales*	*Betaproteobacteria*	*Proteobacteria*	*Bacteria*
Otu0035	1.78	2.68	0.52	0.98	0.56	48.63	*Colwellia*	*Colwelliaceae*	*Alteromonadales*	*Gammaproteobacteria*	*Proteobacteria*	*Bacteria*
Otu0076	1.63	0.2	0.51	2.52	0.55	49.18	unclass. *Flammeovirgaceae*	*Flammeovirgaceae*	*Sphingobacteriales*	*Sphingobacteria*	*Bacteroidetes*	*Bacteria*
Otu0052	1.38	0	0.5	1.19	0.54	49.71	unclass. *Betaproteobacteria*	unclass. *Betaproteobacteria*	unclass. *Betaproteobacteria*	*Betaproteobacteria*	*Proteobacteria*	*Bacteria*
Otu0056	1.39	0	0.49	6.26	0.53	50.25	unclass. *Gammaproteobacteria*	unclass. *Gammaproteobacteria*	unclass. *Gammaproteobacteria*	*Gammaproteobacteria*	*Proteobacteria*	*Bacteria*
Otu0022	1.34	0	0.48	1.43	0.52	50.77	unclass. *Betaproteobacteria*	unclass. *Betaproteobacteria*	unclass. *Betaproteobacteria*	*Betaproteobacteria*	*Proteobacteria*	*Bacteria*
Otu0044	1.35	0	0.48	3.13	0.52	51.29	unclass. *Gammaproteobacteria*	unclass. *Gammaproteobacteria*	unclass. *Gammaproteobacteria*	*Gammaproteobacteria*	*Proteobacteria*	*Bacteria*
Otu0143	1.28	0	0.46	1.64	0.49	51.78	unclass. *Gammaproteobacteria*	unclass. *Gammaproteobacteria*	unclass. *Gammaproteobacteria*	*Gammaproteobacteria*	*Proteobacteria*	*Bacteria*
Otu0291	1.23	0	0.43	4.25	0.47	52.25	unclass. *Flavobacteriaceae*	*Flavobacteriaceae*	*Flavobacteriales*	*Flavobacteria*	*Bacteroidetes*	*Bacteria*
Otu0177	1.17	0	0.41	5.17	0.45	52.7	*Haliea*	*Alteromonadaceae*	*Alteromonadales*	*Gammaproteobacteria*	*Proteobacteria*	*Bacteria*
Otu0262	1.17	0	0.41	5.8	0.45	53.15	unclass. *Gammaproteobacteria*	unclass. *Gammaproteobacteria*	unclass. *Gammaproteobacteria*	*Gammaproteobacteria*	*Proteobacteria*	*Bacteria*
Otu0268	1.15	0	0.41	1.57	0.44	53.59	unclass. *Flavobacteriaceae*	*Flavobacteriaceae*	*Flavobacteriales*	*Flavobacteria*	*Bacteroidetes*	*Bacteria*
Otu0002	1.11	0	0.4	1.77	0.43	54.02	unclass. *Flavobacteriaceae*	*Flavobacteriaceae*	*Flavobacteriales*	*Flavobacteria*	*Bacteroidetes*	*Bacteria*
Otu0013	1.09	0	0.39	1.2	0.42	54.44	unclass. *Flavobacteriaceae*	*Flavobacteriaceae*	*Flavobacteriales*	*Flavobacteria*	*Bacteroidetes*	*Bacteria*
Otu1781	0	1.09	0.39	1.68	0.42	54.86	*Oceaniserpentilla*	*Oceanospirillaceae*	*Oceanospirillales*	*Gammaproteobacteria*	*Proteobacteria*	*Bacteria*
Otu0366	1.09	0	0.38	1.69	0.41	55.28	unclass. *Saprospiraceae*	*Saprospiraceae*	*Sphingobacteriales*	*Sphingobacteria*	*Bacteroidetes*	*Bacteria*
Otu1723	1.08	0	0.38	1.63	0.41	55.69	unclass. *Bacteroidetes*	unclass. *Bacteroidetes*	unclass. *Bacteroidetes*	unclass. *Bacteroidetes*	*Bacteroidetes*	*Bacteria*
Otu0118	1.04	0	0.36	1.06	0.39	56.08	*Colwellia*	*Colwelliaceae*	*Alteromonadales*	*Gammaproteobacteria*	*Proteobacteria*	*Bacteria*

OTU	Av.A_i (no)	Av.A_i (serial)	Av.δ_i	Av.δ_i/SD	Av.δ_i %	∑Av.δ_i %	Genus	Family	Order	Class	Phylum	Domain
Otu0269	0.99	0	0.35	1.08	0.38	56.45	unclass. *Gammaproteobacteria*	unclass. *Gammaproteobacteria*	unclass. *Gammaproteobacteria*	*Gammaproteobacteria*	*Proteobacteria*	*Bacteria*
Otu0009	0.97	0	0.35	1.81	0.37	56.83	unclass. *Rhodobacteraceae*	*Rhodobacteraceae*	*Rhodobacterales*	*Alphaproteobacteria*	*Proteobacteria*	*Bacteria*
Otu0062	0.95	0	0.34	1.09	0.37	57.2	*Haliea*	*Alteromonadaceae*	*Alteromonadales*	*Gammaproteobacteria*	*Proteobacteria*	*Bacteria*
Otu0133	0.97	0	0.34	1.82	0.37	57.56	*Colwellia*	*Colwelliaceae*	*Alteromonadales*	*Gammaproteobacteria*	*Proteobacteria*	*Bacteria*
Otu0046	0.95	0	0.33	1.69	0.36	57.92	unclass. *Flavobacteriaceae*	*Flavobacteriaceae*	*Flavobacteriales*	*Flavobacteria*	*Bacteroidetes*	*Bacteria*
Otu0542	0	0.91	0.32	1.18	0.35	58.27	unclass. *Oceanospirillaceae*	*Oceanospirillaceae*	*Oceanospirillales*	*Gammaproteobacteria*	*Proteobacteria*	*Bacteria*
Otu0170	0.88	0	0.32	1.8	0.34	58.61	unclass. *Bacteria*	unclass. *Bacteria*	unclass. *Bacteria*	unclass. *Bacteria*	unclass. *Bacteria*	*Bacteria*
Otu0203	0.88	0	0.31	1.86	0.34	58.95	*Pelagibacter*	SAR11-clade	*Rickettsiales*	*Alphaproteobacteria*	*Proteobacteria*	*Bacteria*
Otu0232	0.89	0	0.31	1.13	0.34	59.29	unclass. *Rhodobacteraceae*	*Rhodobacteraceae*	*Rhodobacterales*	*Alphaproteobacteria*	*Proteobacteria*	*Bacteria*
Otu0066	0.88	0	0.31	1.1	0.34	59.63	*Granulosicoccus*	*Granulosicoccaceae*	*Chromatiales*	*Gammaproteobacteria*	*Proteobacteria*	*Bacteria*
Otu1837	0.88	0	0.31	1.85	0.33	59.96	unclass. *Flavobacteriales*	unclass. *Flavobacteriales*	*Flavobacteriales*	*Flavobacteria*	*Bacteroidetes*	*Bacteria*
Otu1081	0.82	0	0.29	0.49	0.31	60.27	unclass. *Flavobacteriales*	unclass. *Flavobacteriales*	*Flavobacteriales*	*Flavobacteria*	*Bacteroidetes*	*Bacteria*
Otu1816	0.8	0	0.29	1.96	0.31	60.58	unclass. *Flavobacteriales*	unclass. *Flavobacteriales*	*Flavobacteriales*	*Flavobacteria*	*Bacteroidetes*	*Bacteria*
Otu0117	0.4	0.88	0.28	1.17	0.31	60.89	*Arcobacter*	*Campylobacteraceae*	*Campylobacterales*	*Epsilonproteobacteria*	*Proteobacteria*	*Bacteria*
Otu0348	0.8	0	0.28	1.95	0.31	61.2	unclass. *Flavobacteriaceae*	*Flavobacteriaceae*	*Flavobacteriales*	*Flavobacteria*	*Bacteroidetes*	*Bacteria*
Otu0369	0.8	0	0.28	1.96	0.3	61.5	unclass. *Deltaproteobacteria*	unclass. *Deltaproteobacteria*	unclass. *Deltaproteobacteria*	*Deltaproteobacteria*	*Proteobacteria*	*Bacteria*
Otu0036	0.77	0	0.27	1.16	0.3	61.8	*Glaciecola*	*Alteromonadaceae*	*Alteromonadales*	*Gammaproteobacteria*	*Proteobacteria*	*Bacteria*
Otu1780	0	0.77	0.27	0.68	0.29	62.09	unclass. *Oceanospirillales*	unclass. *Oceanospirillales*	*Oceanospirillales*	*Gammaproteobacteria*	*Proteobacteria*	*Bacteria*
Otu1692	0.77	0	0.27	0.76	0.29	62.38	unclass. *Rhodobacteraceae*	*Rhodobacteraceae*	*Rhodobacterales*	*Alphaproteobacteria*	*Proteobacteria*	*Bacteria*
Otu0025	0.75	0	0.26	0.8	0.28	62.67	*Polaribacter*	*Flavobacteriaceae*	*Flavobacteriales*	*Flavobacteria*	*Bacteroidetes*	*Bacteria*
Otu0140	1.09	1.08	0.25	1.22	0.27	62.94	*Oleispira*	*Oceanospirillaceae*	*Oceanospirillales*	*Gammaproteobacteria*	*Proteobacteria*	*Bacteria*
Otu1821	0.68	0	0.24	1.14	0.27	63.2	unclass. *Bacteroidetes*	unclass. *Bacteroidetes*	unclass. *Bacteroidetes*	unclass. *Bacteroidetes*	*Bacteroidetes*	*Bacteria*
Otu0521	0.68	0	0.24	1.16	0.26	63.47	unclass. *Rhodobacteraceae*	*Rhodobacteraceae*	*Rhodobacterales*	*Alphaproteobacteria*	*Proteobacteria*	*Bacteria*
Otu1629	0.68	0	0.24	1.15	0.26	63.73	unclass. *Flammeovirgaceae*	*Flammeovirgaceae*	*Sphingobacteriales*	*Sphingobacteria*	*Bacteroidetes*	*Bacteria*
Otu0026	0.68	0	0.24	1.16	0.26	63.99	unclass. *Rhodobacteraceae*	*Rhodobacteraceae*	*Rhodobacterales*	*Alphaproteobacteria*	*Proteobacteria*	*Bacteria*
Otu1873	0.68	0	0.24	1.16	0.26	64.25	unclass. *Bacteria*	unclass. *Bacteria*	unclass. *Bacteria*	unclass. *Bacteria*	unclass. *Bacteria*	*Bacteria*
Otu0216	0.63	0	0.22	0.79	0.24	64.48	*Pelagibacter*	SAR11-clade	*Rickettsiales*	*Alphaproteobacteria*	*Proteobacteria*	*Bacteria*

OTU	Av.A_i (no)	Av.A_i (serial)	Av.δ_i	Av.δ_i/SD	Av.δ_i %	∑Av.δ_i %	Genus	Family	Order	Class	Phylum	Domain
Otu0051	0.6	0	0.21	1.2	0.23	64.72	unclass. *Flavobacteriaceae*	*Flavobacteriaceae*	*Flavobacteriales*	*Flavobacteria*	*Bacteroidetes*	*Bacteria*
Otu0176	0	0.6	0.21	1.2	0.23	64.95	*Colwellia*	*Colwelliaceae*	*Alteromonadales*	*Gammaproteobacteria*	*Proteobacteria*	*Bacteria*
Otu1783	0	0.6	0.21	1.2	0.23	65.18	*Marinomonas*	*Oceanospirillaceae*	*Oceanospirillales*	*Gammaproteobacteria*	*Proteobacteria*	*Bacteria*
Otu0364	0.6	0	0.21	1.2	0.23	65.4	unclass. *Flavobacteriaceae*	*Flavobacteriaceae*	*Flavobacteriales*	*Flavobacteria*	*Bacteroidetes*	*Bacteria*
Otu0169	0.6	0	0.21	1.2	0.23	65.63	unclass. *Bacteria*	unclass. *Bacteria*	unclass. *Bacteria*	unclass. *Bacteria*	unclass. *Bacteria*	*Bacteria*
Otu0899	0.6	0	0.21	1.2	0.23	65.86	unclass. *Betaproteobacteria*	unclass. *Betaproteobacteria*	unclass. *Betaproteobacteria*	*Betaproteobacteria*	*Proteobacteria*	*Bacteria*
Otu1828	0.6	0	0.21	0.73	0.23	66.09	unclass. *Chromatiales*	unclass. *Chromatiales*	*Chromatiales*	*Gammaproteobacteria*	*Proteobacteria*	*Bacteria*
Otu0234	0.6	0.2	0.2	1.1	0.22	66.3	*Arcobacter*	*Campylobacteraceae*	*Campylobacterales*	*Epsilonproteobacteria*	*Proteobacteria*	*Bacteria*
Otu0027	0.57	0	0.2	0.8	0.21	66.52	*Pseudoalteromonas*	*Pseudoalteromonadaceae*	*Alteromonadales*	*Gammaproteobacteria*	*Proteobacteria*	*Bacteria*
Otu0173	0.55	0	0.2	0.76	0.21	66.73	unclass. *Alteromonadaceae*	*Alteromonadaceae*	*Alteromonadales*	*Gammaproteobacteria*	*Proteobacteria*	*Bacteria*
Otu0290	0.55	0	0.19	0.76	0.21	66.94	unclass. *Flavobacteriaceae*	*Flavobacteriaceae*	*Flavobacteriales*	*Flavobacteria*	*Bacteroidetes*	*Bacteria*
Otu0533	0	0.48	0.17	0.78	0.19	67.12	*Nisaea*	*Rhodospirillaceae*	*Rhodospirillales*	*Alphaproteobacteria*	*Proteobacteria*	*Bacteria*
Otu1084	0	0.48	0.17	0.78	0.18	67.31	*Marinomonas*	*Oceanospirillaceae*	*Oceanospirillales*	*Gammaproteobacteria*	*Proteobacteria*	*Bacteria*
Otu1119	0.48	0	0.17	0.78	0.18	67.49	unclass. *Bacteroidetes*	unclass. *Bacteroidetes*	unclass. *Bacteroidetes*	unclass. *Bacteroidetes*	*Bacteroidetes*	*Bacteria*
Otu1231	0	0.48	0.17	0.78	0.18	67.68	unclass. *Gammaproteobacteria*	unclass. *Gammaproteobacteria*	unclass. *Gammaproteobacteria*	*Gammaproteobacteria*	*Proteobacteria*	*Bacteria*
Otu0686	0.4	0.4	0.17	0.94	0.18	67.86	*Marinomonas*	*Oceanospirillaceae*	*Oceanospirillales*	*Gammaproteobacteria*	*Proteobacteria*	*Bacteria*
Otu0172	0.48	0	0.17	0.78	0.18	68.04	unclass. *Gammaproteobacteria*	unclass. *Gammaproteobacteria*	unclass. *Gammaproteobacteria*	*Gammaproteobacteria*	*Proteobacteria*	*Bacteria*
Otu0202	0.48	0	0.17	0.78	0.18	68.22	unclass. *Alteromonadaceae*	*Alteromonadaceae*	*Alteromonadales*	*Gammaproteobacteria*	*Proteobacteria*	*Bacteria*
Otu1848	0.48	0	0.17	0.78	0.18	68.41	unclass. *Gammaproteobacteria*	unclass. *Gammaproteobacteria*	unclass. *Gammaproteobacteria*	*Gammaproteobacteria*	*Proteobacteria*	*Bacteria*
Otu0254	0.48	0	0.17	0.78	0.18	68.59	unclass. *Flavobacteriaceae*	*Flavobacteriaceae*	*Flavobacteriales*	*Flavobacteria*	*Bacteroidetes*	*Bacteria*
Otu0333	0.48	0	0.17	0.78	0.18	68.77	unclass. *Gammaproteobacteria*	unclass. *Gammaproteobacteria*	unclass. *Gammaproteobacteria*	*Gammaproteobacteria*	*Proteobacteria*	*Bacteria*
Otu0072	0.48	0	0.17	0.78	0.18	68.95	*Sulfitobacter*	*Rhodobacteraceae*	*Rhodobacterales*	*Alphaproteobacteria*	*Proteobacteria*	*Bacteria*
Otu0153	0.48	0	0.17	0.78	0.18	69.13	unclass. *Gammaproteobacteria*	unclass. *Gammaproteobacteria*	unclass. *Gammaproteobacteria*	*Gammaproteobacteria*	*Proteobacteria*	*Bacteria*
Otu0310	0.48	0	0.17	0.78	0.18	69.32	unclass. *Bacteria*	unclass. *Bacteria*	unclass. *Bacteria*	unclass. *Bacteria*	unclass. *Bacteria*	*Bacteria*
Otu0673	0.48	0	0.17	0.78	0.18	69.5	*Thalassolituus*	*Oceanospirillaceae*	*Oceanospirillales*	*Gammaproteobacteria*	*Proteobacteria*	*Bacteria*
Otu1111	0.45	0	0.16	0.49	0.17	69.67	unclass. *Proteobacteria*	unclass. *Proteobacteria*	unclass. *Proteobacteria*	unclass. *Proteobacteria*	*Proteobacteria*	*Bacteria*
Otu0347	0.4	0	0.15	0.49	0.16	69.83	unclass. *Flavobacteriaceae*	*Flavobacteriaceae*	*Flavobacteriales*	*Flavobacteria*	*Bacteroidetes*	*Bacteria*

OTU	Av.A_i (no)	Av.A_i (serial)	Av.δ_i	Av.δ_i/SD	Av.δ_i %	∑Av.δ_i %	Genus	Family	Order	Class	Phylum	Domain
Otu0283	0.4	0	0.15	0.8	0.16	69.99	unclass. *Gammaproteobacteria*	unclass. *Gammaproteobacteria*	unclass. *Gammaproteobacteria*	*Gammaproteobacteria*	*Proteobacteria*	*Bacteria*
Otu1697	0.4	0	0.15	0.8	0.16	70.15	unclass. *Bacteria*	unclass. *Bacteria*	unclass. *Bacteria*	unclass. *Bacteria*	unclass. *Bacteria*	*Bacteria*
Otu1861	0.4	0	0.15	0.8	0.16	70.3	unclass. *Gammaproteobacteria*	unclass. *Gammaproteobacteria*	unclass. *Gammaproteobacteria*	*Gammaproteobacteria*	*Proteobacteria*	*Bacteria*
Otu0094	0.4	0	0.14	0.8	0.16	70.46	unclass. *Flavobacteriaceae*	*Flavobacteriaceae*	*Flavobacteriales*	*Flavobacteria*	*Bacteroidetes*	*Bacteria*
Otu1267	0.4	0	0.14	0.8	0.16	70.62	unclass. *Gammaproteobacteria*	unclass. *Gammaproteobacteria*	unclass. *Gammaproteobacteria*	*Gammaproteobacteria*	*Proteobacteria*	*Bacteria*
Otu1818	0.4	0	0.14	0.8	0.16	70.77	unclass. *Bacteria*	unclass. *Bacteria*	unclass. *Bacteria*	unclass. *Bacteria*	unclass. *Bacteria*	*Bacteria*
Otu1836	0.4	0	0.14	0.8	0.16	70.93	unclass. *Bacteroidetes*	unclass. *Bacteroidetes*	unclass. *Bacteroidetes*	unclass. *Bacteroidetes*	*Bacteroidetes*	*Bacteria*
Otu0272	0.4	0	0.14	0.8	0.16	71.09	unclass. *Flavobacteriaceae*	*Flavobacteriaceae*	*Flavobacteriales*	*Flavobacteria*	*Bacteroidetes*	*Bacteria*
Otu0057	0.4	0	0.14	0.8	0.16	71.24	*Ulvibacter*	*Flavobacteriaceae*	*Flavobacteriales*	*Flavobacteria*	*Bacteroidetes*	*Bacteria*
Otu0497	0.4	0	0.14	0.8	0.16	71.4	*Opitutus*	*Opitutaceae*	*Opitutales*	*Opitutae*	*Verrucomicrobia*	*Bacteria*
Otu1480	0.4	0	0.14	0.8	0.16	71.55	unclass. *Flavobacteriaceae*	*Flavobacteriaceae*	*Flavobacteriales*	*Flavobacteria*	*Bacteroidetes*	*Bacteria*
Otu1079	0	0.4	0.14	0.8	0.15	71.71	*Marinomonas*	*Oceanospirillaceae*	*Oceanospirillales*	*Gammaproteobacteria*	*Proteobacteria*	*Bacteria*
Otu1065	0	0.4	0.14	0.8	0.15	71.86	*Marinomonas*	*Oceanospirillaceae*	*Oceanospirillales*	*Gammaproteobacteria*	*Proteobacteria*	*Bacteria*
Otu0015	0.4	0	0.14	0.8	0.15	72.02	*Polaribacter*	*Flavobacteriaceae*	*Flavobacteriales*	*Flavobacteria*	*Bacteroidetes*	*Bacteria*
Otu0019	0.4	0	0.14	0.8	0.15	72.17	unclass. *Gammaproteobacteria*	unclass. *Gammaproteobacteria*	unclass. *Gammaproteobacteria*	*Gammaproteobacteria*	*Proteobacteria*	*Bacteria*
Otu0093	0.4	0	0.14	0.8	0.15	72.32	*Aestuariicola*	*Flavobacteriaceae*	*Flavobacteriales*	*Flavobacteria*	*Bacteroidetes*	*Bacteria*
Otu0443	0.4	0	0.14	0.8	0.15	72.47	unclass. *Gammaproteobacteria*	unclass. *Gammaproteobacteria*	unclass. *Gammaproteobacteria*	*Gammaproteobacteria*	*Proteobacteria*	*Bacteria*
Otu0858	0.4	0	0.14	0.8	0.15	72.63	unclass. *Flavobacteriaceae*	*Flavobacteriaceae*	*Flavobacteriales*	*Flavobacteria*	*Bacteroidetes*	*Bacteria*
Otu1827	0.4	0	0.14	0.8	0.15	72.78	unclass. *Acidobacteria_Gp22*	unclass. *Acidobacteria_Gp22*	unclass. *Acidobacteria_Gp22*	*Acidobacteria_Gp22*	*Acidobacteria*	*Bacteria*
Otu1795	0.2	0.28	0.14	0.69	0.15	72.93	*Colwellia*	*Colwelliaceae*	*Alteromonadales*	*Gammaproteobacteria*	*Proteobacteria*	*Bacteria*
Otu0448	0.4	0	0.14	0.8	0.15	73.08	unclass. *Desulfuromonadaceae*	*Desulfuromonadaceae*	*Desulfuromonadales*	*Deltaproteobacteria*	*Proteobacteria*	*Bacteria*
Otu1875	0.4	0	0.14	0.8	0.15	73.24	unclass. *Bacteria*	unclass. *Bacteria*	unclass. *Bacteria*	unclass. *Bacteria*	unclass. *Bacteria*	*Bacteria*
Otu0319	0.4	0	0.14	0.8	0.15	73.39	unclass. *Flammeovirgaceae*	*Flammeovirgaceae*	*Sphingobacteriales*	*Sphingobacteria*	*Bacteroidetes*	*Bacteria*
Otu0356	0.4	0	0.14	0.8	0.15	73.54	unclass. *Comamonadaceae*	*Comamonadaceae*	*Burkholderiales*	*Betaproteobacteria*	*Proteobacteria*	*Bacteria*
Otu1913	0.4	0	0.14	0.8	0.15	73.69	unclass. *Gammaproteobacteria*	unclass. *Gammaproteobacteria*	unclass. *Gammaproteobacteria*	*Gammaproteobacteria*	*Proteobacteria*	*Bacteria*
Otu0370	0.4	0	0.14	0.8	0.15	73.84	unclass. *Gammaproteobacteria*	unclass. *Gammaproteobacteria*	unclass. *Gammaproteobacteria*	*Gammaproteobacteria*	*Proteobacteria*	*Bacteria*
Otu0696	0.4	0	0.14	0.8	0.15	74	unclass. *Flavobacteriaceae*	*Flavobacteriaceae*	*Flavobacteriales*	*Flavobacteria*	*Bacteroidetes*	*Bacteria*

OTU	Av.A_i (no)	Av.A_i (serial)	Av.δ_i	Av.δ_i/SD	Av.δ_i %	∑Av.δ_i %	Genus	Family	Order	Class	Phylum	Domain
Otu1824	0.4	0	0.14	0.8	0.15	74.15	unclass. *Gammaproteobacteria*	unclass. *Gammaproteobacteria*	unclass. *Gammaproteobacteria*	*Gammaproteobacteria*	*Proteobacteria*	*Bacteria*
Otu1851	0.4	0	0.14	0.8	0.15	74.3	*Aureispira*	*Saprospiraceae*	*Sphingobacteriales*	*Sphingobacteria*	*Bacteroidetes*	*Bacteria*
Otu1566	0.4	0	0.14	0.8	0.15	74.45	*Gaetbulibacter*	*Flavobacteriaceae*	*Flavobacteriales*	*Flavobacteria*	*Bacteroidetes*	*Bacteria*
Otu1841	0.4	0	0.14	0.8	0.15	74.6	unclass. *Flavobacteriaceae*	*Flavobacteriaceae*	*Flavobacteriales*	*Flavobacteria*	*Bacteroidetes*	*Bacteria*
Otu0191	0.4	0	0.14	0.8	0.15	74.75	unclass. *Proteobacteria*	unclass. *Proteobacteria*	unclass. *Proteobacteria*	unclass. *Proteobacteria*	*Proteobacteria*	*Bacteria*
Otu0282	0.4	0	0.14	0.49	0.15	74.9	*Haliea*	*Alteromonadaceae*	*Alteromonadales*	*Gammaproteobacteria*	*Proteobacteria*	*Bacteria*
Otu0119	0.35	0	0.13	0.49	0.14	75.04	*Colwellia*	*Colwelliaceae*	*Alteromonadales*	*Gammaproteobacteria*	*Proteobacteria*	*Bacteria*
Otu1883	0.35	0	0.13	0.49	0.14	75.18	unclass. *Rhodobacteraceae*	*Rhodobacteraceae*	*Rhodobacterales*	*Alphaproteobacteria*	*Proteobacteria*	*Bacteria*
Otu0413	0.35	0	0.12	0.49	0.13	75.31	*Eudoraea*	*Flavobacteriaceae*	*Flavobacteriales*	*Flavobacteria*	*Bacteroidetes*	*Bacteria*
Otu0151	0.28	0	0.11	0.49	0.11	75.43	*Rhodococcus*	*Nocardiaceae*	*Actinomycetales*	*Actinobacteria*	*Actinobacteria*	*Bacteria*
Otu0230	0.28	0	0.11	0.49	0.11	75.54	unclass. *Rhodobacteraceae*	*Rhodobacteraceae*	*Rhodobacterales*	*Alphaproteobacteria*	*Proteobacteria*	*Bacteria*
Otu0354	0.28	0	0.11	0.49	0.11	75.65	*Anderseniella*	*Rhodobiaceae*	*Rhizobiales*	*Alphaproteobacteria*	*Proteobacteria*	*Bacteria*
Otu0459	0.28	0	0.11	0.49	0.11	75.77	*Croceibacter*	*Flavobacteriaceae*	*Flavobacteriales*	*Flavobacteria*	*Bacteroidetes*	*Bacteria*
Otu1188	0.28	0	0.11	0.49	0.11	75.88	unclass. *Flammeovirgaceae*	*Flammeovirgaceae*	*Sphingobacteriales*	*Sphingobacteria*	*Bacteroidetes*	*Bacteria*
Otu0490	0.28	0	0.1	0.49	0.11	75.99	unclass. *Burkholderiales*	unclass. *Burkholderiales*	*Burkholderiales*	*Betaproteobacteria*	*Proteobacteria*	*Bacteria*
Otu1274	0.28	0	0.1	0.49	0.11	76.1	unclass. *Gammaproteobacteria*	unclass. *Gammaproteobacteria*	unclass. *Gammaproteobacteria*	*Gammaproteobacteria*	*Proteobacteria*	*Bacteria*
Otu1909	0.28	0	0.1	0.49	0.11	76.21	unclass. *Bacteria*	unclass. *Bacteria*	unclass. *Bacteria*	unclass. *Bacteria*	unclass. *Bacteria*	*Bacteria*
Otu1910	0.28	0	0.1	0.49	0.11	76.32	unclass. *Gammaproteobacteria*	unclass. *Gammaproteobacteria*	unclass. *Gammaproteobacteria*	*Gammaproteobacteria*	*Proteobacteria*	*Bacteria*
Otu1920	0.28	0	0.1	0.49	0.11	76.43	unclass. *Gammaproteobacteria*	unclass. *Gammaproteobacteria*	unclass. *Gammaproteobacteria*	*Gammaproteobacteria*	*Proteobacteria*	*Bacteria*
Otu1922	0.28	0	0.1	0.49	0.11	76.54	unclass. *Bacteria*	unclass. *Bacteria*	unclass. *Bacteria*	unclass. *Bacteria*	unclass. *Bacteria*	*Bacteria*
Otu0267	0.28	0	0.1	0.49	0.11	76.64	unclass. *Flavobacteriaceae*	*Flavobacteriaceae*	*Flavobacteriales*	*Flavobacteria*	*Bacteroidetes*	*Bacteria*
Otu0341	0.28	0	0.1	0.49	0.11	76.75	unclass. *Gammaproteobacteria*	unclass. *Gammaproteobacteria*	unclass. *Gammaproteobacteria*	*Gammaproteobacteria*	*Proteobacteria*	*Bacteria*
Otu1819	0.28	0	0.1	0.49	0.11	76.86	unclass. *Bacteroidetes*	unclass. *Bacteroidetes*	unclass. *Bacteroidetes*	unclass. *Bacteroidetes*	*Bacteroidetes*	*Bacteria*
Otu0115	0	0.28	0.1	0.49	0.11	76.96	*Glaciecola*	*Alteromonadaceae*	*Alteromonadales*	*Gammaproteobacteria*	*Proteobacteria*	*Bacteria*
Otu0256	0.28	0	0.1	0.49	0.11	77.07	*Glaciecola*	*Alteromonadaceae*	*Alteromonadales*	*Gammaproteobacteria*	*Proteobacteria*	*Bacteria*
Otu0508	0.28	0	0.1	0.49	0.11	77.18	unclass. *Flavobacteriales*	unclass. *Flavobacteriales*	*Flavobacteriales*	*Flavobacteria*	*Bacteroidetes*	*Bacteria*
Otu0985	0.28	0	0.1	0.49	0.11	77.28	unclass. *Gammaproteobacteria*	unclass. *Gammaproteobacteria*	unclass. *Gammaproteobacteria*	*Gammaproteobacteria*	*Proteobacteria*	*Bacteria*

OTU	Av.A_i (no)	Av.A_i (serial)	Av.δ_i	Av.δ_i/SD	Av.δ_i %	∑Av.δ_i %	Genus	Family	Order	Class	Phylum	Domain
Otu1854	0.28	0	0.1	0.49	0.11	77.39	unclass. *Gammaproteobacteria*	unclass. *Gammaproteobacteria*	unclass. *Gammaproteobacteria*	*Gammaproteobacteria*	*Proteobacteria*	*Bacteria*
Otu0185	0.28	0	0.1	0.49	0.11	77.5	unclass. *Bacteria*	unclass. *Bacteria*	unclass. *Bacteria*	unclass. *Bacteria*	unclass. *Bacteria*	*Bacteria*
Otu0271	0.28	0	0.1	0.49	0.11	77.6	unclass. *Proteobacteria*	unclass. *Proteobacteria*	unclass. *Proteobacteria*	unclass. *Proteobacteria*	*Proteobacteria*	*Bacteria*
Otu0691	0.28	0	0.1	0.49	0.11	77.71	*Planctomyces*	*Planctomycetaceae*	*Planctomycetales*	*Planctomycetacia*	*Planctomycetes*	*Bacteria*
Otu0880	0.28	0	0.1	0.49	0.11	77.82	unclass. *Flavobacteriaceae*	*Flavobacteriaceae*	*Flavobacteriales*	*Flavobacteria*	*Bacteroidetes*	*Bacteria*
Otu1228	0.28	0	0.1	0.49	0.11	77.92	unclass. *Desulfobulbaceae*	*Desulfobulbaceae*	*Desulfobacterales*	*Deltaproteobacteria*	*Proteobacteria*	*Bacteria*
Otu1696	0.28	0	0.1	0.49	0.11	78.03	*Pelagibacter*	SAR11-clade	*Rickettsiales*	*Alphaproteobacteria*	*Proteobacteria*	*Bacteria*
Otu0028	0.2	0	0.07	0.49	0.08	78.11	unclass. *Gammaproteobacteria*	unclass. *Gammaproteobacteria*	unclass. *Gammaproteobacteria*	*Gammaproteobacteria*	*Proteobacteria*	*Bacteria*
Otu0156	0.2	0	0.07	0.49	0.08	78.19	unclass. *Gammaproteobacteria*	unclass. *Gammaproteobacteria*	unclass. *Gammaproteobacteria*	*Gammaproteobacteria*	*Proteobacteria*	*Bacteria*
Otu0159	0.2	0	0.07	0.49	0.08	78.27	unclass. *Saprospiraceae*	*Saprospiraceae*	*Sphingobacteriales*	*Sphingobacteria*	*Bacteroidetes*	*Bacteria*
Otu0168	0.2	0	0.07	0.49	0.08	78.35	unclass. *Flavobacteriaceae*	*Flavobacteriaceae*	*Flavobacteriales*	*Flavobacteria*	*Bacteroidetes*	*Bacteria*
Otu0192	0.2	0	0.07	0.49	0.08	78.43	unclass. *Gammaproteobacteria*	unclass. *Gammaproteobacteria*	unclass. *Gammaproteobacteria*	*Gammaproteobacteria*	*Proteobacteria*	*Bacteria*
Otu0204	0.2	0	0.07	0.49	0.08	78.51	unclass. *Colwelliaceae*	*Colwelliaceae*	*Alteromonadales*	*Gammaproteobacteria*	*Proteobacteria*	*Bacteria*
Otu0208	0.2	0	0.07	0.49	0.08	78.59	unclass. *Saprospiraceae*	*Saprospiraceae*	*Sphingobacteriales*	*Sphingobacteria*	*Bacteroidetes*	*Bacteria*
Otu0217	0.2	0	0.07	0.49	0.08	78.68	unclass. *Flavobacteriales*	unclass. *Flavobacteriales*	*Flavobacteriales*	*Flavobacteria*	*Bacteroidetes*	*Bacteria*
Otu0240	0.2	0	0.07	0.49	0.08	78.76	unclass. *Chromatiales*	unclass. *Chromatiales*	*Chromatiales*	*Gammaproteobacteria*	*Proteobacteria*	*Bacteria*
Otu0295	0.2	0	0.07	0.49	0.08	78.84	*Haliea*	*Alteromonadaceae*	*Alteromonadales*	*Gammaproteobacteria*	*Proteobacteria*	*Bacteria*
Otu0314	0.2	0	0.07	0.49	0.08	78.92	unclass. *Flavobacteriaceae*	*Flavobacteriaceae*	*Flavobacteriales*	*Flavobacteria*	*Bacteroidetes*	*Bacteria*
Otu0359	0.2	0	0.07	0.49	0.08	79	unclass. *Gammaproteobacteria*	unclass. *Gammaproteobacteria*	unclass. *Gammaproteobacteria*	*Gammaproteobacteria*	*Proteobacteria*	*Bacteria*
Otu0398	0.2	0	0.07	0.49	0.08	79.08	unclass. *Flavobacteriaceae*	*Flavobacteriaceae*	*Flavobacteriales*	*Flavobacteria*	*Bacteroidetes*	*Bacteria*
Otu0436	0.2	0	0.07	0.49	0.08	79.16	unclass. *Rhodobacteraceae*	*Rhodobacteraceae*	*Rhodobacterales*	*Alphaproteobacteria*	*Proteobacteria*	*Bacteria*
Otu0479	0.2	0	0.07	0.49	0.08	79.24	unclass. *Rhodospirillaceae*	*Rhodospirillaceae*	*Rhodospirillales*	*Alphaproteobacteria*	*Proteobacteria*	*Bacteria*
Otu0718	0.2	0	0.07	0.49	0.08	79.32	*Aureispira*	*Saprospiraceae*	*Sphingobacteriales*	*Sphingobacteria*	*Bacteroidetes*	*Bacteria*
Otu0765	0.2	0	0.07	0.49	0.08	79.4	*Pelagicoccus*	*Puniceicoccaceae*	*Puniceicoccales*	*Opitutae*	*Verrucomicrobia*	*Bacteria*
Otu0931	0.2	0	0.07	0.49	0.08	79.48	*Robiginitalea*	*Flavobacteriaceae*	*Flavobacteriales*	*Flavobacteria*	*Bacteroidetes*	*Bacteria*
Otu1008	0.2	0	0.07	0.49	0.08	79.56	unclass. *Flavobacteriaceae*	*Flavobacteriaceae*	*Flavobacteriales*	*Flavobacteria*	*Bacteroidetes*	*Bacteria*
Otu1017	0.2	0	0.07	0.49	0.08	79.64	unclass. *Actinomycetales*	unclass. *Actinomycetales*	*Actinomycetales*	*Actinobacteria*	*Actinobacteria*	*Bacteria*

OTU	Av.A_i (no)	Av.A_i (serial)	Av.δ_i	Av.δ_i/SD	Av.δ_i %	∑Av.δ_i %	Genus	Family	Order	Class	Phylum	Domain
Otu1027	0.2	0	0.07	0.49	0.08	79.72	unclass. *Epsilonproteobacteria*	unclass. *Epsilonproteobacteria*	unclass. *Epsilonproteobacteria*	*Epsilonproteobacteria*	*Proteobacteria*	*Bacteria*
Otu1137	0.2	0	0.07	0.49	0.08	79.8	unclass. *Gammaproteobacteria*	unclass. *Gammaproteobacteria*	unclass. *Gammaproteobacteria*	*Gammaproteobacteria*	*Proteobacteria*	*Bacteria*
Otu1180	0.2	0	0.07	0.49	0.08	79.88	unclass. *Nitrosomonadaceae*	*Nitrosomonadaceae*	*Nitrosomonadales*	*Betaproteobacteria*	*Proteobacteria*	*Bacteria*
Otu1211	0.2	0	0.07	0.49	0.08	79.97	unclass. *Oceanospirillaceae*	*Oceanospirillaceae*	*Oceanospirillales*	*Gammaproteobacteria*	*Proteobacteria*	*Bacteria*
Otu1249	0.2	0	0.07	0.49	0.08	80.05	unclass. *Gammaproteobacteria*	unclass. *Gammaproteobacteria*	unclass. *Gammaproteobacteria*	*Gammaproteobacteria*	*Proteobacteria*	*Bacteria*
Otu1505	0.2	0	0.07	0.49	0.08	80.13	unclass. *Desulfobacteraceae*	*Desulfobacteraceae*	*Desulfobacterales*	*Deltaproteobacteria*	*Proteobacteria*	*Bacteria*
Otu1587	0.2	0	0.07	0.49	0.08	80.21	unclass. *Bacteria*	unclass. *Bacteria*	unclass. *Bacteria*	unclass. *Bacteria*	unclass. *Bacteria*	*Bacteria*
Otu1774	0.2	0	0.07	0.49	0.08	80.29	unclass. *Flavobacteriaceae*	*Flavobacteriaceae*	*Flavobacteriales*	*Flavobacteria*	*Bacteroidetes*	*Bacteria*
Otu1880	0.2	0	0.07	0.49	0.08	80.37	unclass. *Bacteria*	unclass. *Bacteria*	unclass. *Bacteria*	unclass. *Bacteria*	unclass. *Bacteria*	*Bacteria*
Otu1881	0.2	0	0.07	0.49	0.08	80.45	unclass. *Bacteria*	unclass. *Bacteria*	unclass. *Bacteria*	unclass. *Bacteria*	unclass. *Bacteria*	*Bacteria*
Otu1882	0.2	0	0.07	0.49	0.08	80.53	*Truepera*	*Trueperaceae*	*Deinococcales*	*Deinococci*	*Deinococcus-Thermus*	*Bacteria*
Otu1885	0.2	0	0.07	0.49	0.08	80.61	unclass. *Gammaproteobacteria*	unclass. *Gammaproteobacteria*	unclass. *Gammaproteobacteria*	*Gammaproteobacteria*	*Proteobacteria*	*Bacteria*
Otu1886	0.2	0	0.07	0.49	0.08	80.69	unclass. *Bacteria*	unclass. *Bacteria*	unclass. *Bacteria*	unclass. *Bacteria*	unclass. *Bacteria*	*Bacteria*
Otu1888	0.2	0	0.07	0.49	0.08	80.77	unclass. *Bacteria*	unclass. *Bacteria*	unclass. *Bacteria*	unclass. *Bacteria*	unclass. *Bacteria*	*Bacteria*
Otu1889	0.2	0	0.07	0.49	0.08	80.85	unclass. *Gammaproteobacteria*	unclass. *Gammaproteobacteria*	unclass. *Gammaproteobacteria*	*Gammaproteobacteria*	*Proteobacteria*	*Bacteria*
Otu1890	0.2	0	0.07	0.49	0.08	80.93	unclass. *Bacteria*	unclass. *Bacteria*	unclass. *Bacteria*	unclass. *Bacteria*	unclass. *Bacteria*	*Bacteria*
Otu1891	0.2	0	0.07	0.49	0.08	81.01	unclass. *Flavobacteriales*	unclass. *Flavobacteriales*	*Flavobacteriales*	*Flavobacteria*	*Bacteroidetes*	*Bacteria*
Otu1892	0.2	0	0.07	0.49	0.08	81.09	unclass. *Bacteroidetes*	unclass. *Bacteroidetes*	unclass. *Bacteroidetes*	unclass. *Bacteroidetes*	*Bacteroidetes*	*Bacteria*
Otu1896	0.2	0	0.07	0.49	0.08	81.18	unclass. *Gammaproteobacteria*	unclass. *Gammaproteobacteria*	unclass. *Gammaproteobacteria*	*Gammaproteobacteria*	*Proteobacteria*	*Bacteria*
Otu1898	0.2	0	0.07	0.49	0.08	81.26	*Winogradskyella*	*Flavobacteriaceae*	*Flavobacteriales*	*Flavobacteria*	*Bacteroidetes*	*Bacteria*
Otu1899	0.2	0	0.07	0.49	0.08	81.34	unclass. *Ectothiorhodospiraceae*	*Ectothiorhodospiraceae*	*Chromatiales*	*Gammaproteobacteria*	*Proteobacteria*	*Bacteria*
Otu1900	0.2	0	0.07	0.49	0.08	81.42	unclass. *Flavobacteriaceae*	*Flavobacteriaceae*	*Flavobacteriales*	*Flavobacteria*	*Bacteroidetes*	*Bacteria*
Otu1901	0.2	0	0.07	0.49	0.08	81.5	*Glaciecola*	*Alteromonadaceae*	*Alteromonadales*	*Gammaproteobacteria*	*Proteobacteria*	*Bacteria*
Otu1902	0.2	0	0.07	0.49	0.08	81.58	unclass. *Bacteria*	unclass. *Bacteria*	unclass. *Bacteria*	unclass. *Bacteria*	unclass. *Bacteria*	*Bacteria*
Otu1903	0.2	0	0.07	0.49	0.08	81.66	unclass. *Flavobacteriales*	unclass. *Flavobacteriales*	*Flavobacteriales*	*Flavobacteria*	*Bacteroidetes*	*Bacteria*
Otu1904	0.2	0	0.07	0.49	0.08	81.74	unclass. *Flavobacteriales*	unclass. *Flavobacteriales*	*Flavobacteriales*	*Flavobacteria*	*Bacteroidetes*	*Bacteria*
Otu1905	0.2	0	0.07	0.49	0.08	81.82	*Dasania*	*Pseudomonadales_incertae_sedis*	*Pseudomonadales*	*Gammaproteobacteria*	*Proteobacteria*	*Bacteria*

OTU	Av.A_i (no)	Av.A_i (serial)	Av.δ_i	Av.δ_i/SD	Av.δ_i %	∑Av.δ_i %	Genus	Family	Order	Class	Phylum	Domain
Otu1906	0.2	0	0.07	0.49	0.08	81.9	unclass. *Bacteria*	unclass. *Bacteria*	unclass. *Bacteria*	unclass. *Bacteria*	unclass. *Bacteria*	*Bacteria*
Otu1907	0.2	0	0.07	0.49	0.08	81.98	unclass. *Flavobacteriaceae*	*Flavobacteriaceae*	*Flavobacteriales*	*Flavobacteria*	*Bacteroidetes*	*Bacteria*
Otu1908	0.2	0	0.07	0.49	0.08	82.06	unclass. *Flavobacteriaceae*	*Flavobacteriaceae*	*Flavobacteriales*	*Flavobacteria*	*Bacteroidetes*	*Bacteria*
Otu0573	0	0.2	0.07	0.49	0.08	82.14	*Thalassospira*	*Rhodospirillaceae*	*Rhodospirillales*	*Alphaproteobacteria*	*Proteobacteria*	*Bacteria*
Otu1069	0	0.2	0.07	0.49	0.08	82.22	*Marinomonas*	*Oceanospirillaceae*	*Oceanospirillales*	*Gammaproteobacteria*	*Proteobacteria*	*Bacteria*
Otu1778	0	0.2	0.07	0.49	0.08	82.3	*Arcobacter*	*Campylobacteraceae*	*Campylobacterales*	*Epsilonproteobacteria*	*Proteobacteria*	*Bacteria*
Otu1779	0	0.2	0.07	0.49	0.08	82.37	*Marinomonas*	*Oceanospirillaceae*	*Oceanospirillales*	*Gammaproteobacteria*	*Proteobacteria*	*Bacteria*
Otu1784	0	0.2	0.07	0.49	0.08	82.45	unclass. *Gammaproteobacteria*	unclass. *Gammaproteobacteria*	unclass. *Gammaproteobacteria*	*Gammaproteobacteria*	*Proteobacteria*	*Bacteria*
Otu1785	0	0.2	0.07	0.49	0.08	82.53	*Colwellia*	*Colwelliaceae*	*Alteromonadales*	*Gammaproteobacteria*	*Proteobacteria*	*Bacteria*
Otu1786	0	0.2	0.07	0.49	0.08	82.61	unclass. *Gammaproteobacteria*	unclass. *Gammaproteobacteria*	unclass. *Gammaproteobacteria*	*Gammaproteobacteria*	*Proteobacteria*	*Bacteria*
Otu1787	0	0.2	0.07	0.49	0.08	82.69	unclass. *Gammaproteobacteria*	unclass. *Gammaproteobacteria*	unclass. *Gammaproteobacteria*	*Gammaproteobacteria*	*Proteobacteria*	*Bacteria*
Otu1064	0	0.2	0.07	0.49	0.08	82.76	*Marinomonas*	*Oceanospirillaceae*	*Oceanospirillales*	*Gammaproteobacteria*	*Proteobacteria*	*Bacteria*
Otu1790	0	0.2	0.07	0.49	0.08	82.84	unclass. *Bacteria*	unclass. *Bacteria*	unclass. *Bacteria*	unclass. *Bacteria*	unclass. *Bacteria*	*Bacteria*
Otu1791	0	0.2	0.07	0.49	0.08	82.92	*Colwellia*	*Colwelliaceae*	*Alteromonadales*	*Gammaproteobacteria*	*Proteobacteria*	*Bacteria*
Otu0546	0	0.2	0.07	0.49	0.08	83	unclass. *Bacteria*	unclass. *Bacteria*	unclass. *Bacteria*	unclass. *Bacteria*	unclass. *Bacteria*	*Bacteria*
Otu0562	0	0.2	0.07	0.49	0.08	83.07	*Maribacter*	*Flavobacteriaceae*	*Flavobacteriales*	*Flavobacteria*	*Bacteroidetes*	*Bacteria*
Otu0563	0	0.2	0.07	0.49	0.08	83.15	unclass. *Oceanospirillaceae*	*Oceanospirillaceae*	*Oceanospirillales*	*Gammaproteobacteria*	*Proteobacteria*	*Bacteria*
Otu1260	0	0.2	0.07	0.49	0.08	83.23	*Marinobacterium*	*Alteromonadaceae*	*Alteromonadales*	*Gammaproteobacteria*	*Proteobacteria*	*Bacteria*
Otu1788	0	0.2	0.07	0.49	0.08	83.3	*Colwellia*	*Colwelliaceae*	*Alteromonadales*	*Gammaproteobacteria*	*Proteobacteria*	*Bacteria*
Otu1798	0	0.2	0.07	0.49	0.08	83.38	unclass. *Alteromonadales*	unclass. *Alteromonadales*	*Alteromonadales*	*Gammaproteobacteria*	*Proteobacteria*	*Bacteria*
Otu1799	0	0.2	0.07	0.49	0.08	83.46	unclass. *Gammaproteobacteria*	unclass. *Gammaproteobacteria*	unclass. *Gammaproteobacteria*	*Gammaproteobacteria*	*Proteobacteria*	*Bacteria*
Otu1800	0	0.2	0.07	0.49	0.08	83.54	unclass. *Alteromonadales*	unclass. *Alteromonadales*	*Alteromonadales*	*Gammaproteobacteria*	*Proteobacteria*	*Bacteria*
Otu0011	0.2	0	0.07	0.49	0.08	83.61	unclass. *Flavobacteriaceae*	*Flavobacteriaceae*	*Flavobacteriales*	*Flavobacteria*	*Bacteroidetes*	*Bacteria*
Otu0088	0.2	0	0.07	0.49	0.08	83.69	unclass. *Flavobacteriaceae*	*Flavobacteriaceae*	*Flavobacteriales*	*Flavobacteria*	*Bacteroidetes*	*Bacteria*
Otu0108	0.2	0	0.07	0.49	0.08	83.77	unclass. *Colwelliaceae*	*Colwelliaceae*	*Alteromonadales*	*Gammaproteobacteria*	*Proteobacteria*	*Bacteria*
Otu0150	0.2	0	0.07	0.49	0.08	83.84	unclass. *Bacteria*	unclass. *Bacteria*	unclass. *Bacteria*	unclass. *Bacteria*	unclass. *Bacteria*	*Bacteria*
Otu0190	0.2	0	0.07	0.49	0.08	83.92	unclass. *Proteobacteria*	unclass. *Proteobacteria*	unclass. *Proteobacteria*	unclass. *Proteobacteria*	*Proteobacteria*	*Bacteria*

OTU	Av.A_i (no)	Av.A_i (serial)	Av.δ_i	Av.δ_i/SD	Av.δ_i %	∑Av.δ_i %	Genus	Family	Order	Class	Phylum	Domain
Otu0221	0.2	0	0.07	0.49	0.08	84	unclass. *Flavobacteriaceae*	*Flavobacteriaceae*	*Flavobacteriales*	*Flavobacteria*	*Bacteroidetes*	*Bacteria*
Otu0229	0.2	0	0.07	0.49	0.08	84.08	unclass. *Saprospiraceae*	*Saprospiraceae*	*Sphingobacteriales*	*Sphingobacteria*	*Bacteroidetes*	*Bacteria*
Otu0231	0.2	0	0.07	0.49	0.08	84.15	unclass. *Gammaproteobacteria*	unclass. *Gammaproteobacteria*	unclass. *Gammaproteobacteria*	*Gammaproteobacteria*	*Proteobacteria*	*Bacteria*
Otu0238	0.2	0	0.07	0.49	0.08	84.23	*Haliea*	*Alteromonadaceae*	*Alteromonadales*	*Gammaproteobacteria*	*Proteobacteria*	*Bacteria*
Otu0242	0.2	0	0.07	0.49	0.08	84.31	unclass. *Gammaproteobacteria*	unclass. *Gammaproteobacteria*	unclass. *Gammaproteobacteria*	*Gammaproteobacteria*	*Proteobacteria*	*Bacteria*
Otu0278	0.2	0	0.07	0.49	0.08	84.38	unclass. *Gammaproteobacteria*	unclass. *Gammaproteobacteria*	unclass. *Gammaproteobacteria*	*Gammaproteobacteria*	*Proteobacteria*	*Bacteria*
Otu0302	0.2	0	0.07	0.49	0.08	84.46	*Colwellia*	*Colwelliaceae*	*Alteromonadales*	*Gammaproteobacteria*	*Proteobacteria*	*Bacteria*
Otu0312	0.2	0	0.07	0.49	0.08	84.54	unclass. *Oceanospirillaceae*	*Oceanospirillaceae*	*Oceanospirillales*	*Gammaproteobacteria*	*Proteobacteria*	*Bacteria*
Otu0315	0.2	0	0.07	0.49	0.08	84.61	unclass. *Gammaproteobacteria*	unclass. *Gammaproteobacteria*	unclass. *Gammaproteobacteria*	*Gammaproteobacteria*	*Proteobacteria*	*Bacteria*
Otu0334	0.2	0	0.07	0.49	0.08	84.69	unclass. *Flavobacteriaceae*	*Flavobacteriaceae*	*Flavobacteriales*	*Flavobacteria*	*Bacteroidetes*	*Bacteria*
Otu0335	0.2	0	0.07	0.49	0.08	84.77	unclass. *Flavobacteriaceae*	*Flavobacteriaceae*	*Flavobacteriales*	*Flavobacteria*	*Bacteroidetes*	*Bacteria*
Otu0565	0.2	0	0.07	0.49	0.08	84.85	*Staphylococcus*	*Staphylococcaceae*	*Bacillales*	*Bacilli*	*Firmicutes*	*Bacteria*
Otu0618	0.2	0	0.07	0.49	0.08	84.92	*Glaciecola*	*Alteromonadaceae*	*Alteromonadales*	*Gammaproteobacteria*	*Proteobacteria*	*Bacteria*
Otu0760	0.2	0	0.07	0.49	0.08	85	unclass. *Bacteria*	unclass. *Bacteria*	unclass. *Bacteria*	unclass. *Bacteria*	unclass. *Bacteria*	*Bacteria*
Otu0780	0.2	0	0.07	0.49	0.08	85.08	unclass. *Gammaproteobacteria*	unclass. *Gammaproteobacteria*	unclass. *Gammaproteobacteria*	*Gammaproteobacteria*	*Proteobacteria*	*Bacteria*
Otu0798	0.2	0	0.07	0.49	0.08	85.15	unclass. *Gammaproteobacteria*	unclass. *Gammaproteobacteria*	unclass. *Gammaproteobacteria*	*Gammaproteobacteria*	*Proteobacteria*	*Bacteria*
Otu1052	0.2	0	0.07	0.49	0.08	85.23	unclass. *Desulfobacteraceae*	*Desulfobacteraceae*	*Desulfobacterales*	*Deltaproteobacteria*	*Proteobacteria*	*Bacteria*
Otu1115	0.2	0	0.07	0.49	0.08	85.31	*Marinobacter*	*Alteromonadaceae*	*Alteromonadales*	*Gammaproteobacteria*	*Proteobacteria*	*Bacteria*
Otu1204	0.2	0	0.07	0.49	0.08	85.38	unclass. *Bacteroidetes*	unclass. *Bacteroidetes*	unclass. *Bacteroidetes*	unclass. *Bacteroidetes*	*Bacteroidetes*	*Bacteria*
Otu1246	0.2	0	0.07	0.49	0.08	85.46	unclass. *Gammaproteobacteria*	unclass. *Gammaproteobacteria*	unclass. *Gammaproteobacteria*	*Gammaproteobacteria*	*Proteobacteria*	*Bacteria*
Otu1403	0.2	0	0.07	0.49	0.08	85.54	unclass. *Bacteroidetes*	unclass. *Bacteroidetes*	unclass. *Bacteroidetes*	unclass. *Bacteroidetes*	*Bacteroidetes*	*Bacteria*
Otu1620	0.2	0	0.07	0.49	0.08	85.62	*Nitrospira*	*Nitrospiraceae*	*Nitrospirales*	*Nitrospira*	*Nitrospira*	*Bacteria*
Otu1741	0.2	0	0.07	0.49	0.08	85.69	*Pelagibacter*	SAR11-clade	*Rickettsiales*	*Alphaproteobacteria*	*Proteobacteria*	*Bacteria*
Otu1775	0.2	0	0.07	0.49	0.08	85.77	unclass. *Bacteria*	unclass. *Bacteria*	unclass. *Bacteria*	unclass. *Bacteria*	unclass. *Bacteria*	*Bacteria*
Otu1894	0.2	0	0.07	0.49	0.08	85.85	*Colwellia*	*Colwelliaceae*	*Alteromonadales*	*Gammaproteobacteria*	*Proteobacteria*	*Bacteria*
Otu1911	0.2	0	0.07	0.49	0.08	85.92	unclass. *Flavobacteriales*	unclass. *Flavobacteriales*	*Flavobacteriales*	*Flavobacteria*	*Bacteroidetes*	*Bacteria*
Otu1912	0.2	0	0.07	0.49	0.08	86	*Pelagibacter*	SAR11-clade	*Rickettsiales*	*Alphaproteobacteria*	*Proteobacteria*	*Bacteria*

OTU	$Av.A_i$ (no)	$Av.A_i$ (serial)	$Av.\delta_i$	$Av.\delta_i/SD$	$Av.\delta_i$ %	$\sum Av.\delta_i$ %	Genus	Family	Order	Class	Phylum	Domain
Otu1914	0.2	0	0.07	0.49	0.08	86.08	*Microscilla*	*Cytophagaceae*	*Sphingobacteriales*	*Sphingobacteria*	*Bacteroidetes*	*Bacteria*
Otu1915	0.2	0	0.07	0.49	0.08	86.15	unclass. *Proteobacteria*	unclass. *Proteobacteria*	unclass. *Proteobacteria*	unclass. *Proteobacteria*	*Proteobacteria*	*Bacteria*
Otu1917	0.2	0	0.07	0.49	0.08	86.23	unclass. *Bacteria*	unclass. *Bacteria*	unclass. *Bacteria*	unclass. *Bacteria*	unclass. *Bacteria*	*Bacteria*
Otu1918	0.2	0	0.07	0.49	0.08	86.31	unclass. *Flavobacteriaceae*	*Flavobacteriaceae*	*Flavobacteriales*	*Flavobacteria*	*Bacteroidetes*	*Bacteria*
Otu1919	0.2	0	0.07	0.49	0.08	86.39	unclass. *Bacteroidetes*	unclass. *Bacteroidetes*	unclass. *Bacteroidetes*	unclass. *Bacteroidetes*	*Bacteroidetes*	*Bacteria*
Otu1921	0.2	0	0.07	0.49	0.08	86.46	unclass. *Bacteria*	unclass. *Bacteria*	unclass. *Bacteria*	unclass. *Bacteria*	unclass. *Bacteria*	*Bacteria*
Otu1923	0.2	0	0.07	0.49	0.08	86.54	unclass. *Bacteria*	unclass. *Bacteria*	unclass. *Bacteria*	unclass. *Bacteria*	unclass. *Bacteria*	*Bacteria*
Otu1924	0.2	0	0.07	0.49	0.08	86.62	unclass. *Bacteria*	unclass. *Bacteria*	unclass. *Bacteria*	unclass. *Bacteria*	unclass. *Bacteria*	*Bacteria*
Otu1925	0.2	0	0.07	0.49	0.08	86.69	unclass. *Gammaproteobacteria*	unclass. *Gammaproteobacteria*	unclass. *Gammaproteobacteria*	*Gammaproteobacteria*	*Proteobacteria*	*Bacteria*
Otu1926	0.2	0	0.07	0.49	0.08	86.77	unclass. *Flavobacteriaceae*	*Flavobacteriaceae*	*Flavobacteriales*	*Flavobacteria*	*Bacteroidetes*	*Bacteria*
Otu1927	0.2	0	0.07	0.49	0.08	86.85	unclass. *Flavobacteriales*	unclass. *Flavobacteriales*	*Flavobacteriales*	*Flavobacteria*	*Bacteroidetes*	*Bacteria*
Otu1928	0.2	0	0.07	0.49	0.08	86.92	unclass. *Flavobacteriales*	unclass. *Flavobacteriales*	*Flavobacteriales*	*Flavobacteria*	*Bacteroidetes*	*Bacteria*
Otu1929	0.2	0	0.07	0.49	0.08	87	unclass. *OD1*	unclass. *OD1*	unclass. *OD1*	unclass. *OD1*	*OD1*	*Bacteria*
Otu1930	0.2	0	0.07	0.49	0.08	87.08	unclass. *Flavobacteriaceae*	*Flavobacteriaceae*	*Flavobacteriales*	*Flavobacteria*	*Bacteroidetes*	*Bacteria*
Otu1931	0.2	0	0.07	0.49	0.08	87.16	unclass. *Bacteria*	unclass. *Bacteria*	unclass. *Bacteria*	unclass. *Bacteria*	unclass. *Bacteria*	*Bacteria*
Otu1932	0.2	0	0.07	0.49	0.08	87.23	unclass. *Bacteria*	unclass. *Bacteria*	unclass. *Bacteria*	unclass. *Bacteria*	unclass. *Bacteria*	*Bacteria*
Otu1933	0.2	0	0.07	0.49	0.08	87.31	unclass. *Flavobacteriaceae*	*Flavobacteriaceae*	*Flavobacteriales*	*Flavobacteria*	*Bacteroidetes*	*Bacteria*
Otu1934	0.2	0	0.07	0.49	0.08	87.39	unclass. *Flavobacteriaceae*	*Flavobacteriaceae*	*Flavobacteriales*	*Flavobacteria*	*Bacteroidetes*	*Bacteria*
Otu1936	0.2	0	0.07	0.49	0.08	87.46	unclass. *Gammaproteobacteria*	unclass. *Gammaproteobacteria*	unclass. *Gammaproteobacteria*	*Gammaproteobacteria*	*Proteobacteria*	*Bacteria*
Otu1938	0.2	0	0.07	0.49	0.08	87.54	unclass. *Planctomycetaceae*	*Planctomycetaceae*	*Planctomycetales*	*Planctomycetacia*	*Planctomycetes*	*Bacteria*
Otu0525	0	0.2	0.07	0.49	0.08	87.62	*Reinekea*	*Oceanospirillaceae*	*Oceanospirillales*	*Gammaproteobacteria*	*Proteobacteria*	*Bacteria*
Otu1792	0	0.2	0.07	0.49	0.08	87.69	*Colwellia*	*Colwelliaceae*	*Alteromonadales*	*Gammaproteobacteria*	*Proteobacteria*	*Bacteria*
Otu1793	0	0.2	0.07	0.49	0.08	87.77	unclass. *Gammaproteobacteria*	unclass. *Gammaproteobacteria*	unclass. *Gammaproteobacteria*	*Gammaproteobacteria*	*Proteobacteria*	*Bacteria*
Otu0031	0.2	0	0.07	0.49	0.08	87.84	unclass. *Gammaproteobacteria*	unclass. *Gammaproteobacteria*	unclass. *Gammaproteobacteria*	*Gammaproteobacteria*	*Proteobacteria*	*Bacteria*
Otu0070	0.2	0	0.07	0.49	0.08	87.92	*Winogradskyella*	*Flavobacteriaceae*	*Flavobacteriales*	*Flavobacteria*	*Bacteroidetes*	*Bacteria*
Otu0142	0.2	0	0.07	0.49	0.08	88	unclass. *Gammaproteobacteria*	unclass. *Gammaproteobacteria*	unclass. *Gammaproteobacteria*	*Gammaproteobacteria*	*Proteobacteria*	*Bacteria*
Otu0144	0.2	0	0.07	0.49	0.08	88.07	*Lewinella*	*Saprospiraceae*	*Sphingobacteriales*	*Sphingobacteria*	*Bacteroidetes*	*Bacteria*

OTU	Av.A_i (no)	Av.A_i (serial)	Av.δ_i	Av.δ_i/SD	Av.δ_i %	∑Av.δ_i %	Genus	Family	Order	Class	Phylum	Domain
Otu0211	0.2	0	0.07	0.49	0.08	88.15	unclass. *Alphaproteobacteria*	unclass. *Alphaproteobacteria*	unclass. *Alphaproteobacteria*	*Alphaproteobacteria*	*Proteobacteria*	*Bacteria*
Otu0213	0.2	0	0.07	0.49	0.08	88.22	unclass. *Gammaproteobacteria*	unclass. *Gammaproteobacteria*	unclass. *Gammaproteobacteria*	*Gammaproteobacteria*	*Proteobacteria*	*Bacteria*
Otu0247	0.2	0	0.07	0.49	0.08	88.3	unclass. *Flavobacteriaceae*	*Flavobacteriaceae*	*Flavobacteriales*	*Flavobacteria*	*Bacteroidetes*	*Bacteria*
Otu0308	0.2	0	0.07	0.49	0.08	88.37	unclass. *Gammaproteobacteria*	unclass. *Gammaproteobacteria*	unclass. *Gammaproteobacteria*	*Gammaproteobacteria*	*Proteobacteria*	*Bacteria*
Otu0321	0.2	0	0.07	0.49	0.08	88.45	unclass. *Alphaproteobacteria*	unclass. *Alphaproteobacteria*	unclass. *Alphaproteobacteria*	*Alphaproteobacteria*	*Proteobacteria*	*Bacteria*
Otu0331	0.2	0	0.07	0.49	0.08	88.53	unclass. *Gammaproteobacteria*	unclass. *Gammaproteobacteria*	unclass. *Gammaproteobacteria*	*Gammaproteobacteria*	*Proteobacteria*	*Bacteria*
Otu0361	0.2	0	0.07	0.49	0.08	88.6	unclass. *Flavobacteriaceae*	*Flavobacteriaceae*	*Flavobacteriales*	*Flavobacteria*	*Bacteroidetes*	*Bacteria*
Otu0373	0.2	0	0.07	0.49	0.08	88.68	unclass. *Bacteria*	unclass. *Bacteria*	unclass. *Bacteria*	unclass. *Bacteria*	unclass. *Bacteria*	*Bacteria*
Otu0389	0.2	0	0.07	0.49	0.08	88.75	unclass. *Flavobacteriaceae*	*Flavobacteriaceae*	*Flavobacteriales*	*Flavobacteria*	*Bacteroidetes*	*Bacteria*
Otu0421	0.2	0	0.07	0.49	0.08	88.83	unclass. *Gammaproteobacteria*	unclass. *Gammaproteobacteria*	unclass. *Gammaproteobacteria*	*Gammaproteobacteria*	*Proteobacteria*	*Bacteria*
Otu0461	0.2	0	0.07	0.49	0.08	88.9	unclass. *Microbacteriaceae*	*Microbacteriaceae*	*Actinomycetales*	*Actinobacteria*	*Actinobacteria*	*Bacteria*
Otu0498	0.2	0	0.07	0.49	0.08	88.98	unclass. *Flavobacteriales*	unclass. *Flavobacteriales*	*Flavobacteriales*	*Flavobacteria*	*Bacteroidetes*	*Bacteria*
Otu0717	0.2	0	0.07	0.49	0.08	89.06	unclass. *Rhodobacteraceae*	*Rhodobacteraceae*	*Rhodobacterales*	*Alphaproteobacteria*	*Proteobacteria*	*Bacteria*
Otu0873	0.2	0	0.07	0.49	0.08	89.13	unclass. *Gammaproteobacteria*	unclass. *Gammaproteobacteria*	unclass. *Gammaproteobacteria*	*Gammaproteobacteria*	*Proteobacteria*	*Bacteria*
Otu0895	0.2	0	0.07	0.49	0.08	89.21	unclass. *Actinobacteria*	unclass. *Actinobacteria*	unclass. *Actinobacteria*	*Actinobacteria*	*Actinobacteria*	*Bacteria*
Otu1004	0.2	0	0.07	0.49	0.08	89.28	unclass. *Gammaproteobacteria*	unclass. *Gammaproteobacteria*	unclass. *Gammaproteobacteria*	*Gammaproteobacteria*	*Proteobacteria*	*Bacteria*
Otu1229	0.2	0	0.07	0.49	0.08	89.36	*Haliea*	*Alteromonadaceae*	*Alteromonadales*	*Gammaproteobacteria*	*Proteobacteria*	*Bacteria*
Otu1362	0.2	0	0.07	0.49	0.08	89.43	unclass. *Bacteria*	unclass. *Bacteria*	unclass. *Bacteria*	unclass. *Bacteria*	unclass. *Bacteria*	*Bacteria*
Otu1683	0.2	0	0.07	0.49	0.08	89.51	unclass. *Gammaproteobacteria*	unclass. *Gammaproteobacteria*	unclass. *Gammaproteobacteria*	*Gammaproteobacteria*	*Proteobacteria*	*Bacteria*
Otu1687	0.2	0	0.07	0.49	0.08	89.59	*Haliea*	*Alteromonadaceae*	*Alteromonadales*	*Gammaproteobacteria*	*Proteobacteria*	*Bacteria*
Otu1699	0.2	0	0.07	0.49	0.08	89.66	*Flavobacterium*	*Flavobacteriaceae*	*Flavobacteriales*	*Flavobacteria*	*Bacteroidetes*	*Bacteria*
Otu1703	0.2	0	0.07	0.49	0.08	89.74	unclass. *Proteobacteria*	unclass. *Proteobacteria*	unclass. *Proteobacteria*	unclass. *Proteobacteria*	*Proteobacteria*	*Bacteria*
Otu1735	0.2	0	0.07	0.49	0.08	89.81	*Sulfurovum*	*Helicobacteraceae*	*Campylobacterales*	*Epsilonproteobacteria*	*Proteobacteria*	*Bacteria*
Otu1748	0.2	0	0.07	0.49	0.08	89.89	unclass. *Epsilonproteobacteria*	unclass. *Epsilonproteobacteria*	unclass. *Epsilonproteobacteria*	*Epsilonproteobacteria*	*Proteobacteria*	*Bacteria*
Otu1817	0.2	0	0.07	0.49	0.08	89.96	unclass. *Flavobacteriaceae*	*Flavobacteriaceae*	*Flavobacteriales*	*Flavobacteria*	*Bacteroidetes*	*Bacteria*
Otu1820	0.2	0	0.07	0.49	0.08	90.04	*Glaciecola*	*Alteromonadaceae*	*Alteromonadales*	*Gammaproteobacteria*	*Proteobacteria*	*Bacteria*

OTU	Av.A_i (no)	Av.$_i$ (initial)	Av.δ_i	Av.δ_i/SD	Av.δ_i %	$\sum$Av.δ_i %	Genus	Family	Order	Class	Phylum	Domain
Summer pH *in situ* 'no dilution' vs. 'initial dilution' (average dissimilarity: 95.7%)												
Otu0109	0	18.3	7.68	8.57	8.03	8.03	*Alteromonas*	*Alteromonadaceae*	*Alteromonadales*	*Gammaproteobacteria*	*Proteobacteria*	*Bacteria*
Otu0005	15.27	0.2	6.32	8.24	6.6	14.63	*Pelagibacter*	SAR11-clade	*Rickettsiales*	*Alphaproteobacteria*	*Proteobacteria*	*Bacteria*
Otu0691	5.44	0	2.26	3.72	2.37	17	*Planctomyces*	*Planctomycetaceae*	*Planctomycetales*	*Planctomycetacia*	*Planctomycetes*	*Bacteria*
Otu0001	4.58	0	1.92	4.45	2.01	19.01	unclass. *Flavobacteriaceae*	*Flavobacteriaceae*	*Flavobacteriales*	*Flavobacteria*	*Bacteroidetes*	*Bacteria*
Otu0035	0.2	3.47	1.36	3.76	1.43	20.43	*Colwellia*	*Colwelliaceae*	*Alteromonadales*	*Gammaproteobacteria*	*Proteobacteria*	*Bacteria*
Otu0693	3.22	0	1.35	2.21	1.41	21.84	*Planctomyces*	*Planctomycetaceae*	*Planctomycetales*	*Planctomycetacia*	*Planctomycetes*	*Bacteria*
Otu0041	0.75	3.93	1.35	1.77	1.41	23.25	*Sulfitobacter*	*Rhodobacteraceae*	*Rhodobacterales*	*Alphaproteobacteria*	*Proteobacteria*	*Bacteria*
Otu0003	3.21	0	1.35	7.21	1.41	24.66	unclass. *Rhodobacteraceae*	*Rhodobacteraceae*	*Rhodobacterales*	*Alphaproteobacteria*	*Proteobacteria*	*Bacteria*
Otu0203	3.14	0	1.32	2.73	1.38	26.04	*Pelagibacter*	SAR11-clade	*Rickettsiales*	*Alphaproteobacteria*	*Proteobacteria*	*Bacteria*
Otu0052	2.91	0	1.22	4.62	1.28	27.32	unclass. *Betaproteobacteria*	unclass. *Betaproteobacteria*	unclass. *Betaproteobacteria*	*Betaproteobacteria*	*Proteobacteria*	*Bacteria*
Otu0521	1.08	3.2	1.07	2.51	1.12	28.44	unclass. *Rhodobacteraceae*	*Rhodobacteraceae*	*Rhodobacterales*	*Alphaproteobacteria*	*Proteobacteria*	*Bacteria*
Otu0115	0	2.35	0.98	4.13	1.03	29.47	*Glaciecola*	*Alteromonadaceae*	*Alteromonadales*	*Gammaproteobacteria*	*Proteobacteria*	*Bacteria*
Otu0487	0	2.26	0.94	6.82	0.98	30.45	unclass. *Chitinophagaceae*	*Chitinophagaceae*	*Sphingobacteriales*	*Sphingobacteria*	*Bacteroidetes*	*Bacteria*
Otu0692	2.21	0	0.91	2.58	0.96	31.41	unclass. *Bacteria*	unclass. *Bacteria*	unclass. *Bacteria*	unclass. *Bacteria*	unclass. *Bacteria*	*Bacteria*
Otu0007	2.06	0	0.86	4.32	0.9	32.31	unclass. *Flavobacteriales*	unclass. *Flavobacteriales*	*Flavobacteriales*	*Flavobacteria*	*Bacteroidetes*	*Bacteria*
Otu0027	0.2	2.28	0.85	1.83	0.89	33.2	*Pseudoalteromonas*	*Pseudoalteromonadaceae*	*Alteromonadales*	*Gammaproteobacteria*	*Proteobacteria*	*Bacteria*
Otu0110	0	2.08	0.85	1.83	0.89	34.09	unclass. *Rhodobacteraceae*	*Rhodobacteraceae*	*Rhodobacterales*	*Alphaproteobacteria*	*Proteobacteria*	*Bacteria*
Otu0706	1.99	0	0.83	4.15	0.87	34.96	unclass. *Planctomycetaceae*	*Planctomycetaceae*	*Planctomycetales*	*Planctomycetacia*	*Planctomycetes*	*Bacteria*
Otu0532	0.77	2.73	0.83	1.46	0.87	35.82	unclass. *Rhodobacteraceae*	*Rhodobacteraceae*	*Rhodobacterales*	*Alphaproteobacteria*	*Proteobacteria*	*Bacteria*
Otu0461	1.89	0	0.8	1.71	0.84	36.66	unclass. *Microbacteriaceae*	*Microbacteriaceae*	*Actinomycetales*	*Actinobacteria*	*Actinobacteria*	*Bacteria*
Otu0588	0	1.8	0.75	3.17	0.78	37.44	*Amphritea*	*Oceanospirillaceae*	*Oceanospirillales*	*Gammaproteobacteria*	*Proteobacteria*	*Bacteria*
Otu0576	0	1.81	0.75	2.22	0.78	38.22	unclass. *Alphaproteobacteria*	unclass. *Alphaproteobacteria*	unclass. *Alphaproteobacteria*	*Alphaproteobacteria*	*Proteobacteria*	*Bacteria*
Otu0519	1.72	0	0.72	1.96	0.75	38.98	unclass. *Rhodospirillaceae*	*Rhodospirillaceae*	*Rhodospirillales*	*Alphaproteobacteria*	*Proteobacteria*	*Bacteria*
Otu0553	0	1.75	0.72	3.25	0.75	39.73	*Vibrio*	*Vibrionaceae*	*Vibrionales*	*Gammaproteobacteria*	*Proteobacteria*	*Bacteria*

OTU	$Av.A_i$ (no)	$Av._i$ (initial)	$Av.\delta_i$	$Av.\delta_i/SD$	$Av.\delta_i$ %	$\sum Av.\delta_i$ %	Genus	Family	Order	Class	Phylum	Domain
Otu0525	0	1.62	0.68	4.09	0.71	40.44	*Reinekea*	*Oceanospirillaceae*	*Oceanospirillales*	*Gammaproteobacteria*	*Proteobacteria*	*Bacteria*
Otu0473	1.63	0	0.67	2.46	0.7	41.13	unclass. *Alphaproteobacteria*	unclass. *Alphaproteobacteria*	unclass. *Alphaproteobacteria*	*Alphaproteobacteria*	*Proteobacteria*	*Bacteria*
Otu0512	1.58	0	0.67	1.43	0.7	41.83	*Rhodopirellula*	*Planctomycetaceae*	*Planctomycetales*	*Planctomycetacia*	*Planctomycetes*	*Bacteria*
Otu0551	0	1.57	0.66	1.63	0.69	42.52	*Arcobacter*	*Campylobacteraceae*	*Campylobacterales*	*Epsilonproteobacteria*	*Proteobacteria*	*Bacteria*
Otu0450	1.66	0.2	0.65	1.12	0.68	43.2	*Sphingobium*	*Sphingomonadaceae*	*Sphingomonadales*	*Alphaproteobacteria*	*Proteobacteria*	*Bacteria*
Otu0495	1.53	0	0.63	1.7	0.66	43.86	unclass. *Flavobacteriales*	unclass. *Flavobacteriales*	*Flavobacteriales*	*Flavobacteria*	*Bacteroidetes*	*Bacteria*
Otu0469	1.52	0	0.63	1.3	0.66	44.52	unclass. *Bacteria*	unclass. *Bacteria*	unclass. *Bacteria*	unclass. *Bacteria*	unclass. *Bacteria*	*Bacteria*
Otu0068	1.51	0	0.63	4.69	0.66	45.18	*Pelagibacter*	SAR11-clade	*Rickettsiales*	*Alphaproteobacteria*	*Proteobacteria*	*Bacteria*
Otu0463	1.43	0	0.6	3.39	0.63	45.81	unclass. *Flavobacteriaceae*	*Flavobacteriaceae*	*Flavobacteriales*	*Flavobacteria*	*Bacteroidetes*	*Bacteria*
Otu0157	1.37	0	0.58	1.61	0.61	46.42	unclass. *Gammaproteobacteria*	unclass. *Gammaproteobacteria*	unclass. *Gammaproteobacteria*	*Gammaproteobacteria*	*Proteobacteria*	*Bacteria*
Otu0046	1.29	0	0.55	1.74	0.58	46.99	unclass. *Flavobacteriaceae*	*Flavobacteriaceae*	*Flavobacteriales*	*Flavobacteria*	*Bacteroidetes*	*Bacteria*
Otu0460	0.28	1.32	0.51	1.1	0.54	47.53	*Arcobacter*	*Campylobacteraceae*	*Campylobacterales*	*Epsilonproteobacteria*	*Proteobacteria*	*Bacteria*
Otu0107	0	1.25	0.51	1.11	0.54	48.07	unclass. *Rhodobacteraceae*	*Rhodobacteraceae*	*Rhodobacterales*	*Alphaproteobacteria*	*Proteobacteria*	*Bacteria*
Otu0705	1.19	0	0.5	1.19	0.52	48.59	*Planctomyces*	*Planctomycetaceae*	*Planctomycetales*	*Planctomycetacia*	*Planctomycetes*	*Bacteria*
Otu0583	0	1.17	0.49	5.08	0.51	49.1	unclass. *Flavobacteriaceae*	*Flavobacteriaceae*	*Flavobacteriales*	*Flavobacteria*	*Bacteroidetes*	*Bacteria*
Otu0019	1.33	0.2	0.48	1.37	0.5	49.6	unclass. *Gammaproteobacteria*	unclass. *Gammaproteobacteria*	unclass. *Gammaproteobacteria*	*Gammaproteobacteria*	*Proteobacteria*	*Bacteria*
Otu0053	1.29	0.2	0.48	1.4	0.5	50.1	unclass. *Rhodobacteraceae*	*Rhodobacteraceae*	*Rhodobacterales*	*Alphaproteobacteria*	*Proteobacteria*	*Bacteria*
Otu0695	1.12	0	0.48	0.81	0.5	50.6	unclass. *Proteobacteria*	unclass. *Proteobacteria*	unclass. *Proteobacteria*	unclass. *Proteobacteria*	*Proteobacteria*	*Bacteria*
Otu0503	1.15	0	0.47	1.67	0.49	51.09	unclass. *Bacteroidetes*	unclass. *Bacteroidetes*	unclass. *Bacteroidetes*	unclass. *Bacteroidetes*	*Bacteroidetes*	*Bacteria*
Otu0615	0	1.12	0.46	0.84	0.48	51.57	*Neptuniibacter*	*Oceanospirillaceae*	*Oceanospirillales*	*Gammaproteobacteria*	*Proteobacteria*	*Bacteria*
Otu0354	1.11	0	0.46	1.78	0.48	52.06	*Anderseniella*	*Rhodobiaceae*	*Rhizobiales*	*Alphaproteobacteria*	*Proteobacteria*	*Bacteria*
Otu0435	1.12	0	0.46	0.6	0.48	52.54	unclass. *Flavobacteriales*	unclass. *Flavobacteriales*	*Flavobacteriales*	*Flavobacteria*	*Bacteroidetes*	*Bacteria*
Otu0289	1.08	0	0.45	1	0.47	53.01	unclass. *Bacteroidetes*	unclass. *Bacteroidetes*	unclass. *Bacteroidetes*	unclass. *Bacteroidetes*	*Bacteroidetes*	*Bacteria*
Otu0542	0	1.09	0.45	1.72	0.47	53.48	unclass. *Oceanospirillaceae*	*Oceanospirillaceae*	*Oceanospirillales*	*Gammaproteobacteria*	*Proteobacteria*	*Bacteria*
Otu0703	1.03	0	0.43	1.7	0.45	53.92	unclass. *Gammaproteobacteria*	unclass. *Gammaproteobacteria*	unclass. *Gammaproteobacteria*	*Gammaproteobacteria*	*Proteobacteria*	*Bacteria*
Otu0502	1.03	0	0.43	1.78	0.45	54.37	unclass. *Gammaproteobacteria*	unclass. *Gammaproteobacteria*	unclass. *Gammaproteobacteria*	*Gammaproteobacteria*	*Proteobacteria*	*Bacteria*
Otu0489	0	1	0.42	12.95	0.44	54.81	unclass. *Vibrionaceae*	*Vibrionaceae*	*Vibrionales*	*Gammaproteobacteria*	*Proteobacteria*	*Bacteria*

OTU	$Av.A_i$ (no)	$Av._i$ (initial)	$Av.\delta_i$	$Av.\delta_i/SD$	$Av.\delta_i$ %	$\sum Av.\delta_i$ %	Genus	Family	Order	Class	Phylum	Domain
Otu0186	0.97	0	0.41	1.82	0.43	55.24	unclass. *Planctomycetaceae*	*Planctomycetaceae*	*Planctomycetales*	*Planctomycetacia*	*Planctomycetes*	*Bacteria*
Otu0704	0.97	0	0.41	1.84	0.43	55.67	unclass. *Proteobacteria*	unclass. *Proteobacteria*	unclass. *Proteobacteria*	unclass. *Proteobacteria*	*Proteobacteria*	*Bacteria*
Otu0753	0.95	0	0.4	0.64	0.42	56.09	unclass. *Flavobacteriales*	unclass. *Flavobacteriales*	*Flavobacteriales*	*Flavobacteria*	*Bacteroidetes*	*Bacteria*
Otu0245	0.97	0	0.4	1.79	0.42	56.51	unclass. *Flavobacteriales*	unclass. *Flavobacteriales*	*Flavobacteriales*	*Flavobacteria*	*Bacteroidetes*	*Bacteria*
Otu0578	0	0.97	0.4	1.77	0.42	56.92	*Winogradskyella*	*Flavobacteriaceae*	*Flavobacteriales*	*Flavobacteria*	*Bacteroidetes*	*Bacteria*
Otu0533	0	0.95	0.39	1.58	0.41	57.34	*Nisaea*	*Rhodospirillaceae*	*Rhodospirillales*	*Alphaproteobacteria*	*Proteobacteria*	*Bacteria*
Otu0084	0.93	0	0.39	1.03	0.41	57.74	unclass. *Gammaproteobacteria*	unclass. *Gammaproteobacteria*	unclass. *Gammaproteobacteria*	*Gammaproteobacteria*	*Proteobacteria*	*Bacteria*
Otu0036	0	0.95	0.39	1.68	0.41	58.15	*Glaciecola*	*Alteromonadaceae*	*Alteromonadales*	*Gammaproteobacteria*	*Proteobacteria*	*Bacteria*
Otu0779	0.95	0	0.39	0.69	0.4	58.55	unclass. *Ectothiorhodospiraceae*	*Ectothiorhodospiraceae*	*Chromatiales*	*Gammaproteobacteria*	*Proteobacteria*	*Bacteria*
Otu0580	0	0.91	0.37	1.19	0.39	58.95	*Neptuniibacter*	*Oceanospirillaceae*	*Oceanospirillales*	*Gammaproteobacteria*	*Proteobacteria*	*Bacteria*
Otu0701	0.88	0	0.37	1.06	0.39	59.33	unclass. *Bacteria*	unclass. *Bacteria*	unclass. *Bacteria*	unclass. *Bacteria*	unclass. *Bacteria*	*Bacteria*
Otu0611	0.72	0.2	0.37	0.62	0.38	59.72	unclass. *Bacteroidetes*	unclass. *Bacteroidetes*	unclass. *Bacteroidetes*	unclass. *Bacteroidetes*	*Bacteroidetes*	*Bacteria*
Otu0010	0.88	0.68	0.36	1.61	0.38	60.09	unclass. *Flavobacteriaceae*	*Flavobacteriaceae*	*Flavobacteriales*	*Flavobacteria*	*Bacteroidetes*	*Bacteria*
Otu0234	0	0.85	0.36	0.79	0.37	60.47	*Arcobacter*	*Campylobacteraceae*	*Campylobacterales*	*Epsilonproteobacteria*	*Proteobacteria*	*Bacteria*
Otu0261	0.77	1.24	0.35	1.33	0.37	60.84	*Winogradskyella*	*Flavobacteriaceae*	*Flavobacteriales*	*Flavobacteria*	*Bacteroidetes*	*Bacteria*
Otu0599	0	0.8	0.34	1.93	0.35	61.19	*Winogradskyella*	*Flavobacteriaceae*	*Flavobacteriales*	*Flavobacteria*	*Bacteroidetes*	*Bacteria*
Otu0232	0	0.8	0.34	1	0.35	61.54	unclass. *Rhodobacteraceae*	*Rhodobacteraceae*	*Rhodobacterales*	*Alphaproteobacteria*	*Proteobacteria*	*Bacteria*
Otu0165	0.2	0.83	0.33	1.16	0.34	61.88	*Marinomonas*	*Oceanospirillaceae*	*Oceanospirillales*	*Gammaproteobacteria*	*Proteobacteria*	*Bacteria*
Otu0694	0.75	0	0.31	1.06	0.33	62.21	unclass. *Alphaproteobacteria*	unclass. *Alphaproteobacteria*	unclass. *Alphaproteobacteria*	*Alphaproteobacteria*	*Proteobacteria*	*Bacteria*
Otu0408	0.75	0	0.3	1.13	0.32	62.53	unclass. *Flavobacteriaceae*	*Flavobacteriaceae*	*Flavobacteriales*	*Flavobacteria*	*Bacteroidetes*	*Bacteria*
Otu0535	0	0.75	0.3	0.49	0.32	62.84	*Loktanella*	*Rhodobacteraceae*	*Rhodobacterales*	*Alphaproteobacteria*	*Proteobacteria*	*Bacteria*
Otu0714	0.68	0	0.29	1.15	0.31	63.15	unclass. *Alphaproteobacteria*	unclass. *Alphaproteobacteria*	unclass. *Alphaproteobacteria*	*Alphaproteobacteria*	*Proteobacteria*	*Bacteria*
Otu0504	0.68	0	0.29	1.15	0.31	63.46	unclass. *Alphaproteobacteria*	unclass. *Alphaproteobacteria*	unclass. *Alphaproteobacteria*	*Alphaproteobacteria*	*Proteobacteria*	*Bacteria*
Otu0142	0.68	0	0.29	1.16	0.31	63.77	unclass. *Gammaproteobacteria*	unclass. *Gammaproteobacteria*	unclass. *Gammaproteobacteria*	*Gammaproteobacteria*	*Proteobacteria*	*Bacteria*
Otu0696	0.68	0	0.29	1.14	0.3	64.07	unclass. *Flavobacteriaceae*	*Flavobacteriaceae*	*Flavobacteriales*	*Flavobacteria*	*Bacteroidetes*	*Bacteria*
Otu0577	0	0.73	0.29	0.77	0.3	64.37	unclass. *Bacteroidetes*	unclass. *Bacteroidetes*	unclass. *Bacteroidetes*	unclass. *Bacteroidetes*	*Bacteroidetes*	*Bacteria*
Otu0346	0	0.68	0.28	1.16	0.29	64.67	unclass. *Bacteria*	unclass. *Bacteria*	unclass. *Bacteria*	unclass. *Bacteria*	unclass. *Bacteria*	*Bacteria*

OTU	$Av.A_i$ (no)	$Av._i$ (initial)	$Av.\delta_i$	$Av.\delta_i/SD$	$Av.\delta_i$ %	$\sum Av.\delta_i$ %	Genus	Family	Order	Class	Phylum	Domain
Otu0700	0.68	0	0.28	1.18	0.29	64.96	unclass. *Alphaproteobacteria*	unclass. *Alphaproteobacteria*	unclass. *Alphaproteobacteria*	*Alphaproteobacteria*	*Proteobacteria*	*Bacteria*
Otu0782	0.68	0	0.28	1.14	0.29	65.25	unclass. *Gammaproteobacteria*	unclass. *Gammaproteobacteria*	unclass. *Gammaproteobacteria*	*Gammaproteobacteria*	*Proteobacteria*	*Bacteria*
Otu0048	0.63	0	0.27	0.79	0.28	65.53	unclass. *Flavobacteriaceae*	*Flavobacteriaceae*	*Flavobacteriales*	*Flavobacteria*	*Bacteroidetes*	*Bacteria*
Otu0459	0.2	0.68	0.27	1.13	0.28	65.81	*Croceibacter*	*Flavobacteriaceae*	*Flavobacteriales*	*Flavobacteria*	*Bacteroidetes*	*Bacteria*
Otu0781	0.63	0	0.27	0.49	0.28	66.09	unclass. *Thiotrichales*	unclass. *Thiotrichales*	*Thiotrichales*	*Gammaproteobacteria*	*Proteobacteria*	*Bacteria*
Otu0697	0.6	0	0.26	1.19	0.27	66.36	unclass. *Flavobacteriaceae*	*Flavobacteriaceae*	*Flavobacteriales*	*Flavobacteria*	*Bacteroidetes*	*Bacteria*
Otu0575	0	0.65	0.26	0.71	0.27	66.63	unclass. *Bacteroidetes*	unclass. *Bacteroidetes*	unclass. *Bacteroidetes*	unclass. *Bacteroidetes*	*Bacteroidetes*	*Bacteria*
Otu0020	0.63	0	0.26	0.8	0.27	66.89	unclass. *Flammeovirgaceae*	*Flammeovirgaceae*	*Sphingobacteriales*	*Sphingobacteria*	*Bacteroidetes*	*Bacteria*
Otu0505	0.63	0	0.25	0.8	0.26	67.16	unclass. *Chitinophagaceae*	*Chitinophagaceae*	*Sphingobacteriales*	*Sphingobacteria*	*Bacteroidetes*	*Bacteria*
Otu0737	0.63	0	0.25	0.8	0.26	67.42	unclass. *Gammaproteobacteria*	unclass. *Gammaproteobacteria*	unclass. *Gammaproteobacteria*	*Gammaproteobacteria*	*Proteobacteria*	*Bacteria*
Otu0476	0.6	0	0.25	1.19	0.26	67.68	unclass. *Actinomycetales*	unclass. *Actinomycetales*	*Actinomycetales*	*Actinobacteria*	*Actinobacteria*	*Bacteria*
Otu0272	0.6	0	0.25	1.19	0.26	67.94	unclass. *Flavobacteriaceae*	*Flavobacteriaceae*	*Flavobacteriales*	*Flavobacteria*	*Bacteroidetes*	*Bacteria*
Otu0368	0.6	0	0.25	1.19	0.26	68.19	unclass. *Saprospiraceae*	*Saprospiraceae*	*Sphingobacteriales*	*Sphingobacteria*	*Bacteroidetes*	*Bacteria*
Otu0191	0.6	0	0.25	1.19	0.26	68.45	unclass. *Proteobacteria*	unclass. *Proteobacteria*	unclass. *Proteobacteria*	unclass. *Proteobacteria*	*Proteobacteria*	*Bacteria*
Otu0485	0.6	0	0.25	1.19	0.26	68.71	unclass. *Alphaproteobacteria*	unclass. *Alphaproteobacteria*	unclass. *Alphaproteobacteria*	*Alphaproteobacteria*	*Proteobacteria*	*Bacteria*
Otu0103	0	0.6	0.25	0.75	0.26	68.97	*Pseudomonas*	*Pseudomonadaceae*	*Pseudomonadales*	*Gammaproteobacteria*	*Proteobacteria*	*Bacteria*
Otu0113	0	0.55	0.24	0.75	0.26	69.22	*Colwellia*	*Colwelliaceae*	*Alteromonadales*	*Gammaproteobacteria*	*Proteobacteria*	*Bacteria*
Otu0721	0.6	0	0.24	1.19	0.25	69.48	unclass. *Alphaproteobacteria*	unclass. *Alphaproteobacteria*	unclass. *Alphaproteobacteria*	*Alphaproteobacteria*	*Proteobacteria*	*Bacteria*
Otu0768	0.6	0	0.24	1.19	0.25	69.73	unclass. *Flavobacteriales*	unclass. *Flavobacteriales*	*Flavobacteriales*	*Flavobacteria*	*Bacteroidetes*	*Bacteria*
Otu0468	0.57	0	0.24	0.8	0.25	69.99	unclass. *Flavobacteriaceae*	*Flavobacteriaceae*	*Flavobacteriales*	*Flavobacteria*	*Bacteroidetes*	*Bacteria*
Otu0607	0	0.55	0.24	0.74	0.25	70.23	unclass. *Flavobacteriaceae*	*Flavobacteriaceae*	*Flavobacteriales*	*Flavobacteria*	*Bacteroidetes*	*Bacteria*
Otu0244	0	0.55	0.24	0.74	0.25	70.48	unclass. *Rhodobacteraceae*	*Rhodobacteraceae*	*Rhodobacterales*	*Alphaproteobacteria*	*Proteobacteria*	*Bacteria*
Otu0597	0	0.55	0.24	0.74	0.25	70.73	*Arenibacter*	*Flavobacteriaceae*	*Flavobacteriales*	*Flavobacteria*	*Bacteroidetes*	*Bacteria*
Otu0595	0.4	0.48	0.23	1.01	0.24	70.97	unclass. *Legionellaceae*	*Legionellaceae*	*Legionellales*	*Gammaproteobacteria*	*Proteobacteria*	*Bacteria*
Otu0584	0	0.55	0.22	0.75	0.23	71.2	unclass. *Proteobacteria*	unclass. *Proteobacteria*	unclass. *Proteobacteria*	unclass. *Proteobacteria*	*Proteobacteria*	*Bacteria*
Otu0125	0	0.48	0.22	0.78	0.23	71.43	*Leeuwenhoekiella*	*Flavobacteriaceae*	*Flavobacteriales*	*Flavobacteria*	*Bacteroidetes*	*Bacteria*
Otu0608	0	0.48	0.21	0.77	0.22	71.64	unclass. *Flavobacteriaceae*	*Flavobacteriaceae*	*Flavobacteriales*	*Flavobacteria*	*Bacteroidetes*	*Bacteria*

OTU	Av.A_i (no)	Av.$_i$ (initial)	Av.δ_i	Av.δ_i/SD	Av.δ_i %	∑Av.δ_i %	Genus	Family	Order	Class	Phylum	Domain
Otu0080	0.48	0	0.21	0.77	0.22	71.86	*Polaribacter*	*Flavobacteriaceae*	*Flavobacteriales*	*Flavobacteria*	*Bacteroidetes*	*Bacteria*
Otu0497	0.48	0	0.21	0.77	0.22	72.08	*Opitutus*	*Opitutaceae*	*Opitutales*	*Opitutae*	*Verrucomicrobia*	*Bacteria*
Otu0754	0.48	0	0.21	0.77	0.22	72.29	unclass. *Flavobacteriales*	unclass. *Flavobacteriales*	*Flavobacteriales*	*Flavobacteria*	*Bacteroidetes*	*Bacteria*
Otu0324	0	0.48	0.2	0.79	0.21	72.51	*Nisaea*	*Rhodospirillaceae*	*Rhodospirillales*	*Alphaproteobacteria*	*Proteobacteria*	*Bacteria*
Otu0547	0	0.48	0.2	0.76	0.21	72.72	*Phenylobacterium*	*Caulobacteraceae*	*Caulobacterales*	*Alphaproteobacteria*	*Proteobacteria*	*Bacteria*
Otu0710	0.48	0	0.2	0.78	0.21	72.93	unclass. *Gammaproteobacteria*	unclass. *Gammaproteobacteria*	unclass. *Gammaproteobacteria*	*Gammaproteobacteria*	*Proteobacteria*	*Bacteria*
Otu0596	0	0.48	0.2	0.78	0.2	73.14	*Neptunomonas*	*Oceanospirillaceae*	*Oceanospirillales*	*Gammaproteobacteria*	*Proteobacteria*	*Bacteria*
Otu0218	0.48	0	0.19	0.79	0.2	73.34	unclass. *Flavobacteriaceae*	*Flavobacteriaceae*	*Flavobacteriales*	*Flavobacteria*	*Bacteroidetes*	*Bacteria*
Otu0758	0.48	0	0.19	0.79	0.2	73.54	unclass. *Bacteria*	unclass. *Bacteria*	unclass. *Bacteria*	unclass. *Bacteria*	unclass. *Bacteria*	*Bacteria*
Otu0221	0.48	0	0.19	0.77	0.2	73.75	unclass. *Flavobacteriaceae*	*Flavobacteriaceae*	*Flavobacteriales*	*Flavobacteria*	*Bacteroidetes*	*Bacteria*
Otu0798	0.48	0	0.19	0.77	0.2	73.95	unclass. *Gammaproteobacteria*	unclass. *Gammaproteobacteria*	unclass. *Gammaproteobacteria*	*Gammaproteobacteria*	*Proteobacteria*	*Bacteria*
Otu0732	0.48	0	0.19	0.77	0.2	74.15	unclass. *Proteobacteria*	unclass. *Proteobacteria*	unclass. *Proteobacteria*	unclass. *Proteobacteria*	*Proteobacteria*	*Bacteria*
Otu0423	0	0.48	0.19	0.78	0.2	74.35	unclass. *Flavobacteriaceae*	*Flavobacteriaceae*	*Flavobacteriales*	*Flavobacteria*	*Bacteroidetes*	*Bacteria*
Otu0543	0	0.48	0.19	0.78	0.2	74.55	*Muricauda*	*Flavobacteriaceae*	*Flavobacteriales*	*Flavobacteria*	*Bacteroidetes*	*Bacteria*
Otu0632	0	0.49	0.19	0.49	0.2	74.75	unclass. *Flavobacteriaceae*	*Flavobacteriaceae*	*Flavobacteriales*	*Flavobacteria*	*Bacteroidetes*	*Bacteria*
Otu0277	0.4	0.2	0.19	0.87	0.2	74.95	unclass. *Flavobacteriaceae*	*Flavobacteriaceae*	*Flavobacteriales*	*Flavobacteria*	*Bacteroidetes*	*Bacteria*
Otu0840	0.49	0	0.19	0.49	0.2	75.15	unclass. *Flavobacteriaceae*	*Flavobacteriaceae*	*Flavobacteriales*	*Flavobacteria*	*Bacteroidetes*	*Bacteria*
Otu0555	0.2	0.4	0.18	0.87	0.19	75.33	*Maricaulis*	*Hyphomonadaceae*	*Caulobacterales*	*Alphaproteobacteria*	*Proteobacteria*	*Bacteria*
Otu0038	0.4	0	0.17	0.8	0.18	75.52	unclass. *Proteobacteria*	unclass. *Proteobacteria*	unclass. *Proteobacteria*	unclass. *Proteobacteria*	*Proteobacteria*	*Bacteria*
Otu0216	0.4	0	0.17	0.8	0.18	75.7	*Pelagibacter*	SAR11-clade	*Rickettsiales*	*Alphaproteobacteria*	*Proteobacteria*	*Bacteria*
Otu0735	0.4	0	0.17	0.8	0.18	75.88	unclass. *Gammaproteobacteria*	unclass. *Gammaproteobacteria*	unclass. *Gammaproteobacteria*	*Gammaproteobacteria*	*Proteobacteria*	*Bacteria*
Otu0745	0.4	0	0.17	0.8	0.18	76.06	*Planctomyces*	*Planctomycetaceae*	*Planctomycetales*	*Planctomycetacia*	*Planctomycetes*	*Bacteria*
Otu0002	0.4	0	0.17	0.8	0.18	76.24	unclass. *Flavobacteriaceae*	*Flavobacteriaceae*	*Flavobacteriales*	*Flavobacteria*	*Bacteroidetes*	*Bacteria*
Otu0724	0.4	0	0.17	0.8	0.18	76.42	unclass. *Bacteria*	unclass. *Bacteria*	unclass. *Bacteria*	unclass. *Bacteria*	unclass. *Bacteria*	*Bacteria*
Otu0746	0.4	0	0.17	0.8	0.18	76.59	unclass. *Flavobacteriaceae*	*Flavobacteriaceae*	*Flavobacteriales*	*Flavobacteria*	*Bacteroidetes*	*Bacteria*
Otu0202	0.4	0	0.17	0.8	0.18	76.77	unclass. *Alteromonadaceae*	*Alteromonadaceae*	*Alteromonadales*	*Gammaproteobacteria*	*Proteobacteria*	*Bacteria*
Otu0051	0.4	0	0.17	0.8	0.18	76.95	unclass. *Flavobacteriaceae*	*Flavobacteriaceae*	*Flavobacteriales*	*Flavobacteria*	*Bacteroidetes*	*Bacteria*

OTU	Av.A_i (no)	Av.$_i$ (initial)	Av.δ_i	Av.δ_i/SD	Av.δ_i %	∑Av.δ_i %	Genus	Family	Order	Class	Phylum	Domain
Otu0352	0.4	0	0.17	0.8	0.18	77.13	unclass. *Flavobacteriaceae*	*Flavobacteriaceae*	*Flavobacteriales*	*Flavobacteria*	*Bacteroidetes*	*Bacteria*
Otu0568	0	0.4	0.17	0.8	0.18	77.31	unclass. *Flavobacteriales*	unclass. *Flavobacteriales*	*Flavobacteriales*	*Flavobacteria*	*Bacteroidetes*	*Bacteria*
Otu0112	0	0.4	0.17	0.8	0.18	77.49	*Polaribacter*	*Flavobacteriaceae*	*Flavobacteriales*	*Flavobacteria*	*Bacteroidetes*	*Bacteria*
Otu0783	0.4	0	0.17	0.49	0.18	77.66	unclass. *Gammaproteobacteria*	unclass. *Gammaproteobacteria*	unclass. *Gammaproteobacteria*	*Gammaproteobacteria*	*Proteobacteria*	*Bacteria*
Otu0604	0	0.4	0.17	0.79	0.18	77.84	unclass. *Bacteroidetes*	unclass. *Bacteroidetes*	unclass. *Bacteroidetes*	unclass. *Bacteroidetes*	*Bacteroidetes*	*Bacteria*
Otu0434	0	0.4	0.17	0.8	0.17	78.01	*Maribacter*	*Flavobacteriaceae*	*Flavobacteriales*	*Flavobacteria*	*Bacteroidetes*	*Bacteria*
Otu0598	0	0.4	0.17	0.8	0.17	78.19	*Arcobacter*	*Campylobacteraceae*	*Campylobacterales*	*Epsilonproteobacteria*	*Proteobacteria*	*Bacteria*
Otu0727	0.4	0	0.16	0.79	0.17	78.36	unclass. *Deltaproteobacteria*	unclass. *Deltaproteobacteria*	unclass. *Deltaproteobacteria*	*Deltaproteobacteria*	*Proteobacteria*	*Bacteria*
Otu0472	0.4	0	0.16	0.79	0.17	78.53	unclass. *Bacteroidetes*	unclass. *Bacteroidetes*	unclass. *Bacteroidetes*	unclass. *Bacteroidetes*	*Bacteroidetes*	*Bacteria*
Otu0490	0.4	0	0.16	0.79	0.17	78.7	unclass. *Burkholderiales*	unclass. *Burkholderiales*	*Burkholderiales*	*Betaproteobacteria*	*Proteobacteria*	*Bacteria*
Otu0766	0.4	0	0.16	0.79	0.17	78.87	*Lentisphaera*	*Lentisphaeraceae*	*Lentisphaerales*	*Lentisphaeria*	*Lentisphaerae*	*Bacteria*
Otu0774	0.4	0	0.16	0.79	0.17	79.04	unclass. *Bacteria*	unclass. *Bacteria*	unclass. *Bacteria*	unclass. *Bacteria*	unclass. *Bacteria*	*Bacteria*
Otu0564	0	0.4	0.16	0.49	0.17	79.21	unclass. *Bacteroidetes*	unclass. *Bacteroidetes*	unclass. *Bacteroidetes*	unclass. *Bacteroidetes*	*Bacteroidetes*	*Bacteria*
Otu0616	0	0.4	0.16	0.49	0.17	79.38	unclass. *Bacteria*	unclass. *Bacteria*	unclass. *Bacteria*	unclass. *Bacteria*	unclass. *Bacteria*	*Bacteria*
Otu0536	0	0.4	0.16	0.8	0.17	79.55	*Maribacter*	*Flavobacteriaceae*	*Flavobacteriales*	*Flavobacteria*	*Bacteroidetes*	*Bacteria*
Otu0582	0	0.4	0.16	0.8	0.17	79.72	unclass. *Rhodobacteraceae*	*Rhodobacteraceae*	*Rhodobacterales*	*Alphaproteobacteria*	*Proteobacteria*	*Bacteria*
Otu0593	0	0.4	0.16	0.8	0.17	79.89	*Psychroserpens*	*Flavobacteriaceae*	*Flavobacteriales*	*Flavobacteria*	*Bacteroidetes*	*Bacteria*
Otu0569	0	0.4	0.16	0.49	0.17	80.06	unclass. *Proteobacteria*	unclass. *Proteobacteria*	unclass. *Proteobacteria*	unclass. *Proteobacteria*	*Proteobacteria*	*Bacteria*
Otu0541	0.4	0	0.16	0.8	0.17	80.23	unclass. *Rhodobacteraceae*	*Rhodobacteraceae*	*Rhodobacterales*	*Alphaproteobacteria*	*Proteobacteria*	*Bacteria*
Otu0736	0.4	0	0.16	0.8	0.17	80.39	*Persicirhabdus*	*Verrucomicrobiaceae*	*Verrucomicrobiales*	*Verrucomicrobiae*	*Verrucomicrobia*	*Bacteria*
Otu0811	0.4	0	0.16	0.8	0.17	80.56	unclass. *Bacteria*	unclass. *Bacteria*	unclass. *Bacteria*	unclass. *Bacteria*	unclass. *Bacteria*	*Bacteria*
Otu0255	0.4	0	0.16	0.8	0.17	80.73	*Sphingopyxis*	*Sphingomonadaceae*	*Sphingomonadales*	*Alphaproteobacteria*	*Proteobacteria*	*Bacteria*
Otu0467	0.4	0	0.16	0.8	0.17	80.89	unclass. *Rhodospirillaceae*	*Rhodospirillaceae*	*Rhodospirillales*	*Alphaproteobacteria*	*Proteobacteria*	*Bacteria*
Otu0470	0.4	0	0.16	0.8	0.17	81.06	unclass. *Flavobacteriaceae*	*Flavobacteriaceae*	*Flavobacteriales*	*Flavobacteria*	*Bacteroidetes*	*Bacteria*
Otu0707	0.4	0	0.16	0.8	0.17	81.23	*Haliea*	*Alteromonadaceae*	*Alteromonadales*	*Gammaproteobacteria*	*Proteobacteria*	*Bacteria*
Otu0787	0.4	0	0.16	0.8	0.17	81.4	unclass. *Verrucomicrobia*	unclass. *Verrucomicrobia*	unclass. *Verrucomicrobia*	unclass. *Verrucomicrobia*	*Verrucomicrobia*	*Bacteria*
Otu0819	0.4	0	0.16	0.8	0.17	81.56	unclass. *Bacteria*	unclass. *Bacteria*	unclass. *Bacteria*	unclass. *Bacteria*	unclass. *Bacteria*	*Bacteria*

OTU	$Av.A_i$ (no)	$Av._i$ (initial)	$Av.\delta_i$	$Av.\delta_i/SD$	$Av.\delta_i$ %	$\sum Av.\delta_i$ %	Genus	Family	Order	Class	Phylum	Domain
Otu0263	0	0.4	0.16	0.8	0.17	81.73	*Krokinobacter*	*Flavobacteriaceae*	*Flavobacteriales*	*Flavobacteria*	*Bacteroidetes*	*Bacteria*
Otu0320	0	0.4	0.16	0.8	0.17	81.9	*Pseudoalteromonas*	*Pseudoalteromonadaceae*	*Alteromonadales*	*Gammaproteobacteria*	*Proteobacteria*	*Bacteria*
Otu0628	0	0.4	0.16	0.8	0.17	82.06	*Lacinutrix*	*Flavobacteriaceae*	*Flavobacteriales*	*Flavobacteria*	*Bacteroidetes*	*Bacteria*
Otu0111	0	0.4	0.16	0.8	0.17	82.23	*Marinomonas*	*Oceanospirillaceae*	*Oceanospirillales*	*Gammaproteobacteria*	*Proteobacteria*	*Bacteria*
Otu0500	0	0.4	0.16	0.8	0.17	82.39	*Marinobacter*	*Alteromonadaceae*	*Alteromonadales*	*Gammaproteobacteria*	*Proteobacteria*	*Bacteria*
Otu0072	0.35	0	0.15	0.49	0.16	82.55	*Sulfitobacter*	*Rhodobacteraceae*	*Rhodobacterales*	*Alphaproteobacteria*	*Proteobacteria*	*Bacteria*
Otu0757	0.35	0	0.15	0.49	0.16	82.71	unclass. *Gammaproteobacteria*	unclass. *Gammaproteobacteria*	unclass. *Gammaproteobacteria*	*Gammaproteobacteria*	*Proteobacteria*	*Bacteria*
Otu0789	0.35	0	0.15	0.49	0.15	82.86	unclass. *Proteobacteria*	unclass. *Proteobacteria*	unclass. *Proteobacteria*	unclass. *Proteobacteria*	*Proteobacteria*	*Bacteria*
Otu0031	0.35	0	0.15	0.49	0.15	83.01	unclass. *Gammaproteobacteria*	unclass. *Gammaproteobacteria*	unclass. *Gammaproteobacteria*	*Gammaproteobacteria*	*Proteobacteria*	*Bacteria*
Otu0380	0.35	0	0.15	0.49	0.15	83.17	unclass. *Proteobacteria*	unclass. *Proteobacteria*	unclass. *Proteobacteria*	unclass. *Proteobacteria*	*Proteobacteria*	*Bacteria*
Otu0814	0.35	0	0.15	0.49	0.15	83.32	unclass. *Proteobacteria*	unclass. *Proteobacteria*	unclass. *Proteobacteria*	unclass. *Proteobacteria*	*Proteobacteria*	*Bacteria*
Otu0579	0	0.35	0.14	0.49	0.15	83.46	unclass. *Bacteroidetes*	unclass. *Bacteroidetes*	unclass. *Bacteroidetes*	unclass. *Bacteroidetes*	*Bacteroidetes*	*Bacteria*
Otu0060	0.35	0	0.13	0.49	0.14	83.6	unclass. *Actinomycetales*	unclass. *Actinomycetales*	*Actinomycetales*	*Actinobacteria*	*Actinobacteria*	*Bacteria*
Otu0630	0.35	0	0.13	0.49	0.14	83.74	unclass. *Flavobacteriales*	unclass. *Flavobacteriales*	*Flavobacteriales*	*Flavobacteria*	*Bacteroidetes*	*Bacteria*
Otu0617	0.2	0.2	0.13	0.67	0.13	83.87	*Algoriphagus*	*Cyclobacteriaceae*	*Sphingobacteriales*	*Sphingobacteria*	*Bacteroidetes*	*Bacteria*
Otu0600	0	0.28	0.13	0.49	0.13	84	*Jannaschia*	*Rhodobacteraceae*	*Rhodobacterales*	*Alphaproteobacteria*	*Proteobacteria*	*Bacteria*
Otu0017	0.28	0	0.12	0.49	0.13	84.13	unclass. *Flavobacteriales*	unclass. *Flavobacteriales*	*Flavobacteriales*	*Flavobacteria*	*Bacteroidetes*	*Bacteria*
Otu0030	0.28	0	0.12	0.49	0.13	84.26	unclass. *Gammaproteobacteria*	unclass. *Gammaproteobacteria*	unclass. *Gammaproteobacteria*	*Gammaproteobacteria*	*Proteobacteria*	*Bacteria*
Otu0759	0.28	0	0.12	0.49	0.13	84.39	unclass. *Verrucomicrobiaceae*	*Verrucomicrobiaceae*	*Verrucomicrobiales*	*Verrucomicrobiae*	*Verrucomicrobia*	*Bacteria*
Otu0075	0.28	0	0.12	0.49	0.12	84.52	unclass. *Alphaproteobacteria*	unclass. *Alphaproteobacteria*	unclass. *Alphaproteobacteria*	*Alphaproteobacteria*	*Proteobacteria*	*Bacteria*
Otu0282	0.28	0	0.12	0.49	0.12	84.64	*Haliea*	*Alteromonadaceae*	*Alteromonadales*	*Gammaproteobacteria*	*Proteobacteria*	*Bacteria*
Otu0743	0.28	0	0.12	0.49	0.12	84.76	unclass. *Verrucomicrobia*	unclass. *Verrucomicrobia*	unclass. *Verrucomicrobia*	unclass. *Verrucomicrobia*	*Verrucomicrobia*	*Bacteria*
Otu0761	0.28	0	0.12	0.49	0.12	84.89	unclass. *Verrucomicrobiaceae*	*Verrucomicrobiaceae*	*Verrucomicrobiales*	*Verrucomicrobiae*	*Verrucomicrobia*	*Bacteria*
Otu0810	0.28	0	0.12	0.49	0.12	85.01	unclass. *Gammaproteobacteria*	unclass. *Gammaproteobacteria*	unclass. *Gammaproteobacteria*	*Gammaproteobacteria*	*Proteobacteria*	*Bacteria*
Otu0822	0.28	0	0.12	0.49	0.12	85.14	unclass. *Proteobacteria*	unclass. *Proteobacteria*	unclass. *Proteobacteria*	unclass. *Proteobacteria*	*Proteobacteria*	*Bacteria*
Otu0827	0.28	0	0.12	0.49	0.12	85.26	unclass. *Bacteria*	unclass. *Bacteria*	unclass. *Bacteria*	unclass. *Bacteria*	unclass. *Bacteria*	*Bacteria*
Otu0618	0	0.28	0.12	0.49	0.12	85.38	*Glaciecola*	*Alteromonadaceae*	*Alteromonadales*	*Gammaproteobacteria*	*Proteobacteria*	*Bacteria*

OTU	$Av.A_i$ (no)	$Av._i$ (initial)	$Av.\delta_i$	$Av.\delta_i/SD$	$Av.\delta_i$ %	$\sum Av.\delta_i$ %	Genus	Family	Order	Class	Phylum	Domain
Otu0574	0	0.28	0.11	0.49	0.12	85.5	unclass. *Alphaproteobacteria*	unclass. *Alphaproteobacteria*	unclass. *Alphaproteobacteria*	*Alphaproteobacteria*	*Proteobacteria*	*Bacteria*
Otu0585	0	0.28	0.11	0.49	0.12	85.62	*Neptuniibacter*	*Oceanospirillaceae*	*Oceanospirillales*	*Gammaproteobacteria*	*Proteobacteria*	*Bacteria*
Otu0635	0	0.28	0.11	0.49	0.12	85.73	unclass. *Rhodobacteraceae*	*Rhodobacteraceae*	*Rhodobacterales*	*Alphaproteobacteria*	*Proteobacteria*	*Bacteria*
Otu0641	0	0.28	0.11	0.49	0.12	85.85	*Flavobacterium*	*Flavobacteriaceae*	*Flavobacteriales*	*Flavobacteria*	*Bacteroidetes*	*Bacteria*
Otu0644	0	0.28	0.11	0.49	0.12	85.96	*Polaribacter*	*Flavobacteriaceae*	*Flavobacteriales*	*Flavobacteria*	*Bacteroidetes*	*Bacteria*
Otu0550	0.28	0	0.11	0.49	0.11	86.08	unclass. *Flavobacteriales*	unclass. *Flavobacteriales*	*Flavobacteriales*	*Flavobacteria*	*Bacteroidetes*	*Bacteria*
Otu0772	0.28	0	0.11	0.49	0.11	86.19	unclass. *Alphaproteobacteria*	unclass. *Alphaproteobacteria*	unclass. *Alphaproteobacteria*	*Alphaproteobacteria*	*Proteobacteria*	*Bacteria*
Otu0839	0.28	0	0.11	0.49	0.11	86.3	unclass. *Bacteria*	unclass. *Bacteria*	unclass. *Bacteria*	unclass. *Bacteria*	unclass. *Bacteria*	*Bacteria*
Otu0054	0	0.2	0.09	0.49	0.09	86.4	*Pseudomonas*	*Pseudomonadaceae*	*Pseudomonadales*	*Gammaproteobacteria*	*Proteobacteria*	*Bacteria*
Otu0180	0	0.2	0.09	0.49	0.09	86.49	*Reichenbachiella*	*Flammeovirgaceae*	*Sphingobacteriales*	*Sphingobacteria*	*Bacteroidetes*	*Bacteria*
Otu0297	0	0.2	0.09	0.49	0.09	86.58	*Lutibacter*	*Flavobacteriaceae*	*Flavobacteriales*	*Flavobacteria*	*Bacteroidetes*	*Bacteria*
Otu0609	0	0.2	0.09	0.49	0.09	86.68	unclass. *Alphaproteobacteria*	unclass. *Alphaproteobacteria*	unclass. *Alphaproteobacteria*	*Alphaproteobacteria*	*Proteobacteria*	*Bacteria*
Otu0610	0	0.2	0.09	0.49	0.09	86.77	*Alteromonas*	*Alteromonadaceae*	*Alteromonadales*	*Gammaproteobacteria*	*Proteobacteria*	*Bacteria*
Otu0612	0	0.2	0.09	0.49	0.09	86.87	unclass. *Alteromonadales*	unclass. *Alteromonadales*	*Alteromonadales*	*Gammaproteobacteria*	*Proteobacteria*	*Bacteria*
Otu0613	0	0.2	0.09	0.49	0.09	86.96	*Ulvibacter*	*Flavobacteriaceae*	*Flavobacteriales*	*Flavobacteria*	*Bacteroidetes*	*Bacteria*
Otu0614	0	0.2	0.09	0.49	0.09	87.05	unclass. *Flavobacteriaceae*	*Flavobacteriaceae*	*Flavobacteriales*	*Flavobacteria*	*Bacteroidetes*	*Bacteria*
Otu0118	0	0.2	0.09	0.49	0.09	87.15	*Colwellia*	*Colwelliaceae*	*Alteromonadales*	*Gammaproteobacteria*	*Proteobacteria*	*Bacteria*
Otu0544	0	0.2	0.09	0.49	0.09	87.24	*Alteromonas*	*Alteromonadaceae*	*Alteromonadales*	*Gammaproteobacteria*	*Proteobacteria*	*Bacteria*
Otu0561	0	0.2	0.09	0.49	0.09	87.33	*Cellulophaga*	*Flavobacteriaceae*	*Flavobacteriales*	*Flavobacteria*	*Bacteroidetes*	*Bacteria*
Otu0601	0	0.2	0.09	0.49	0.09	87.43	unclass. *Flammeovirgaceae*	*Flammeovirgaceae*	*Sphingobacteriales*	*Sphingobacteria*	*Bacteroidetes*	*Bacteria*
Otu0602	0	0.2	0.09	0.49	0.09	87.52	*Maribacter*	*Flavobacteriaceae*	*Flavobacteriales*	*Flavobacteria*	*Bacteroidetes*	*Bacteria*
Otu0603	0	0.2	0.09	0.49	0.09	87.61	unclass. *Sphingobacteriales*	unclass. *Sphingobacteriales*	*Sphingobacteriales*	*Sphingobacteria*	*Bacteroidetes*	*Bacteria*
Otu0605	0	0.2	0.09	0.49	0.09	87.7	unclass. *Flavobacteriaceae*	*Flavobacteriaceae*	*Flavobacteriales*	*Flavobacteria*	*Bacteroidetes*	*Bacteria*
Otu0167	0.2	0	0.09	0.49	0.09	87.8	unclass. *Rhodobacteraceae*	*Rhodobacteraceae*	*Rhodobacterales*	*Alphaproteobacteria*	*Proteobacteria*	*Bacteria*
Otu0213	0.2	0	0.09	0.49	0.09	87.89	unclass. *Gammaproteobacteria*	unclass. *Gammaproteobacteria*	unclass. *Gammaproteobacteria*	*Gammaproteobacteria*	*Proteobacteria*	*Bacteria*
Otu0290	0.2	0	0.09	0.49	0.09	87.98	unclass. *Flavobacteriaceae*	*Flavobacteriaceae*	*Flavobacteriales*	*Flavobacteria*	*Bacteroidetes*	*Bacteria*
Otu0464	0.2	0	0.09	0.49	0.09	88.07	unclass. *Bacteroidetes*	unclass. *Bacteroidetes*	unclass. *Bacteroidetes*	unclass. *Bacteroidetes*	*Bacteroidetes*	*Bacteria*

OTU	$Av.A_i$ (no)	$Av._i$ (initial)	$Av.\delta_i$	$Av.\delta_i/SD$	$Av.\delta_i$ %	$\sum Av.\delta_i$ %	Genus	Family	Order	Class	Phylum	Domain
Otu0482	0.2	0	0.09	0.49	0.09	88.16	unclass. *Microbacteriaceae*	*Microbacteriaceae*	*Actinomycetales*	*Actinobacteria*	*Actinobacteria*	*Bacteria*
Otu0594	0.2	0	0.09	0.49	0.09	88.25	unclass. *Bacteria*	unclass. *Bacteria*	unclass. *Bacteria*	unclass. *Bacteria*	unclass. *Bacteria*	*Bacteria*
Otu0666	0.2	0	0.09	0.49	0.09	88.35	unclass. *Deltaproteobacteria*	unclass. *Deltaproteobacteria*	unclass. *Deltaproteobacteria*	*Deltaproteobacteria*	*Proteobacteria*	*Bacteria*
Otu0702	0.2	0	0.09	0.49	0.09	88.44	unclass. *Flavobacteriales*	unclass. *Flavobacteriales*	*Flavobacteriales*	*Flavobacteria*	*Bacteroidetes*	*Bacteria*
Otu0709	0.2	0	0.09	0.49	0.09	88.53	unclass. *Bacteria*	unclass. *Bacteria*	unclass. *Bacteria*	unclass. *Bacteria*	unclass. *Bacteria*	*Bacteria*
Otu0712	0.2	0	0.09	0.49	0.09	88.62	unclass. *Alphaproteobacteria*	unclass. *Alphaproteobacteria*	unclass. *Alphaproteobacteria*	*Alphaproteobacteria*	*Proteobacteria*	*Bacteria*
Otu0719	0.2	0	0.09	0.49	0.09	88.71	unclass. *Flavobacteriales*	unclass. *Flavobacteriales*	*Flavobacteriales*	*Flavobacteria*	*Bacteroidetes*	*Bacteria*
Otu0722	0.2	0	0.09	0.49	0.09	88.8	unclass. *Rhodobacteraceae*	*Rhodobacteraceae*	*Rhodobacterales*	*Alphaproteobacteria*	*Proteobacteria*	*Bacteria*
Otu0723	0.2	0	0.09	0.49	0.09	88.9	unclass. *Microbacteriaceae*	*Microbacteriaceae*	*Actinomycetales*	*Actinobacteria*	*Actinobacteria*	*Bacteria*
Otu0726	0.2	0	0.09	0.49	0.09	88.99	unclass. *Bacteroidetes*	unclass. *Bacteroidetes*	unclass. *Bacteroidetes*	unclass. *Bacteroidetes*	*Bacteroidetes*	*Bacteria*
Otu0728	0.2	0	0.09	0.49	0.09	89.08	unclass. *Gammaproteobacteria*	unclass. *Gammaproteobacteria*	unclass. *Gammaproteobacteria*	*Gammaproteobacteria*	*Proteobacteria*	*Bacteria*
Otu0729	0.2	0	0.09	0.49	0.09	89.17	unclass. *Bacteroidetes*	unclass. *Bacteroidetes*	unclass. *Bacteroidetes*	unclass. *Bacteroidetes*	*Bacteroidetes*	*Bacteria*
Otu0731	0.2	0	0.09	0.49	0.09	89.26	*Lentisphaera*	*Lentisphaeraceae*	*Lentisphaerales*	*Lentisphaeria*	*Lentisphaerae*	*Bacteria*
Otu0747	0.2	0	0.09	0.49	0.09	89.35	unclass. *Rhodobacteraceae*	*Rhodobacteraceae*	*Rhodobacterales*	*Alphaproteobacteria*	*Proteobacteria*	*Bacteria*
Otu0748	0.2	0	0.09	0.49	0.09	89.45	unclass. *Proteobacteria*	unclass. *Proteobacteria*	unclass. *Proteobacteria*	unclass. *Proteobacteria*	*Proteobacteria*	*Bacteria*
Otu0749	0.2	0	0.09	0.49	0.09	89.54	unclass. *Gammaproteobacteria*	unclass. *Gammaproteobacteria*	unclass. *Gammaproteobacteria*	*Gammaproteobacteria*	*Proteobacteria*	*Bacteria*
Otu0750	0.2	0	0.09	0.49	0.09	89.63	unclass. *Gammaproteobacteria*	unclass. *Gammaproteobacteria*	unclass. *Gammaproteobacteria*	*Gammaproteobacteria*	*Proteobacteria*	*Bacteria*
Otu0083	0.2	0	0.09	0.49	0.09	89.72	unclass. *Rhodobacteraceae*	*Rhodobacteraceae*	*Rhodobacterales*	*Alphaproteobacteria*	*Proteobacteria*	*Bacteria*
Otu0174	0.2	0	0.09	0.49	0.09	89.81	unclass. *Flavobacteriaceae*	*Flavobacteriaceae*	*Flavobacteriales*	*Flavobacteria*	*Bacteroidetes*	*Bacteria*
Otu0178	0.2	0	0.09	0.49	0.09	89.9	unclass. *Actinobacteria*	unclass. *Actinobacteria*	unclass. *Actinobacteria*	*Actinobacteria*	*Actinobacteria*	*Bacteria*
Otu0381	0.2	0	0.09	0.49	0.09	89.99	unclass. *Chitinophagaceae*	*Chitinophagaceae*	*Sphingobacteriales*	*Sphingobacteria*	*Bacteroidetes*	*Bacteria*
Otu0483	0.2	0	0.09	0.49	0.09	90.09	unclass. *Flavobacteriaceae*	*Flavobacteriaceae*	*Flavobacteriales*	*Flavobacteria*	*Bacteroidetes*	*Bacteria*

OTU	Av.A$_i$ (serial)	Av.A$_i$ (initiall)	Av.δ$_i$	Av.δ$_i$/SD	Av.δ$_i$ %	∑Av.δ$_i$ %	Genus	Family	Order	Class	Phylum	Domain
Summer pH *in situ* 'serial dilution' vs. 'initial dilution' (average dissimilarity: 85.6%)												
Otu0109	0.88	18.3	10.68	4.51	12.47	12.47	*Alteromonas*	*Alteromonadaceae*	*Alteromonadales*	*Gammaproteobacteria*	*Proteobacteria*	*Bacteria*
Otu0132	11.11	0	7.2	1.38	8.41	20.88	unclass. *Alteromonadales*	unclass. *Alteromonadales*	*Alteromonadales*	*Gammaproteobacteria*	*Proteobacteria*	*Bacteria*
Otu0459	10.06	0.68	5.45	1.85	6.37	27.25	*Croceibacter*	*Flavobacteriaceae*	*Flavobacteriales*	*Flavobacteria*	*Bacteroidetes*	*Bacteria*
Otu0471	6.52	0	3.99	2.53	4.66	31.9	*Oceaniserpentilla*	*Oceanospirillaceae*	*Oceanospirillales*	*Gammaproteobacteria*	*Proteobacteria*	*Bacteria*
Otu0125	4.15	0.48	2.23	1.74	2.6	34.51	*Leeuwenhoekiella*	*Flavobacteriaceae*	*Flavobacteriales*	*Flavobacteria*	*Bacteroidetes*	*Bacteria*
Otu0035	0	3.47	2.08	4.32	2.43	36.94	*Colwellia*	*Colwelliaceae*	*Alteromonadales*	*Gammaproteobacteria*	*Proteobacteria*	*Bacteria*
Otu0041	0.88	3.93	1.91	1.56	2.23	39.17	*Sulfitobacter*	*Rhodobacteraceae*	*Rhodobacterales*	*Alphaproteobacteria*	*Proteobacteria*	*Bacteria*
Otu0521	4.58	3.2	1.83	1.34	2.13	41.31	unclass. *Rhodobacteraceae*	*Rhodobacteraceae*	*Rhodobacterales*	*Alphaproteobacteria*	*Proteobacteria*	*Bacteria*
Otu0460	3.42	1.32	1.67	1.14	1.95	43.26	*Arcobacter*	*Campylobacteraceae*	*Campylobacterales*	*Epsilonproteobacteria*	*Proteobacteria*	*Bacteria*
Otu0115	0	2.35	1.43	3.62	1.67	44.92	*Glaciecola*	*Alteromonadaceae*	*Alteromonadales*	*Gammaproteobacteria*	*Proteobacteria*	*Bacteria*
Otu0523	2.3	0	1.3	1.57	1.52	46.44	*Pseudidiomarina*	*Idiomarinaceae*	*Alteromonadales*	*Gammaproteobacteria*	*Proteobacteria*	*Bacteria*
Otu0532	0.88	2.73	1.18	1.49	1.38	47.82	unclass. *Rhodobacteraceae*	*Rhodobacteraceae*	*Rhodobacterales*	*Alphaproteobacteria*	*Proteobacteria*	*Bacteria*
Otu0027	1.11	2.28	1.17	1.62	1.37	49.19	*Pseudoalteromonas*	*Pseudoalteromonadaceae*	*Alteromonadales*	*Gammaproteobacteria*	*Proteobacteria*	*Bacteria*
Otu0487	1.38	2.26	1.13	2.4	1.32	50.51	unclass. *Chitinophagaceae*	*Chitinophagaceae*	*Sphingobacteriales*	*Sphingobacteria*	*Bacteroidetes*	*Bacteria*
Otu0588	0	1.8	1.08	3.02	1.26	51.78	*Amphritea*	*Oceanospirillaceae*	*Oceanospirillales*	*Gammaproteobacteria*	*Proteobacteria*	*Bacteria*
Otu0576	0	1.81	1.08	2.2	1.26	53.03	unclass. *Alphaproteobacteria*	unclass. *Alphaproteobacteria*	unclass. *Alphaproteobacteria*	*Alphaproteobacteria*	*Proteobacteria*	*Bacteria*
Otu0524	1.85	0	1.06	1.81	1.24	54.27	*Pseudidiomarina*	*Idiomarinaceae*	*Alteromonadales*	*Gammaproteobacteria*	*Proteobacteria*	*Bacteria*
Otu0110	0.55	2.08	1.01	1.76	1.18	55.45	unclass. *Rhodobacteraceae*	*Rhodobacteraceae*	*Rhodobacterales*	*Alphaproteobacteria*	*Proteobacteria*	*Bacteria*
Otu0522	1.75	0	0.99	1.79	1.16	56.61	unclass. *Flavobacteriaceae*	*Flavobacteriaceae*	*Flavobacteriales*	*Flavobacteria*	*Bacteroidetes*	*Bacteria*
Otu0531	1.7	0	0.97	1.58	1.13	57.75	unclass. *Flavobacteriaceae*	*Flavobacteriaceae*	*Flavobacteriales*	*Flavobacteria*	*Bacteroidetes*	*Bacteria*
Otu0553	0.2	1.75	0.93	2.17	1.09	58.83	*Vibrio*	*Vibrionaceae*	*Vibrionales*	*Gammaproteobacteria*	*Proteobacteria*	*Bacteria*
Otu0551	0.2	1.57	0.89	1.5	1.04	59.87	*Arcobacter*	*Campylobacteraceae*	*Campylobacterales*	*Epsilonproteobacteria*	*Proteobacteria*	*Bacteria*
Otu0537	1.38	0	0.8	1.77	0.93	60.8	unclass. *Flavobacteriaceae*	*Flavobacteriaceae*	*Flavobacteriales*	*Flavobacteria*	*Bacteroidetes*	*Bacteria*
Otu0526	1.39	0.2	0.78	1.06	0.91	61.71	*Maribacter*	*Flavobacteriaceae*	*Flavobacteriales*	*Flavobacteria*	*Bacteroidetes*	*Bacteria*

OTU	Av.A_i (serial)	Av.A_i (initiall)	Av.δ_i	Av.δ_i/SD	Av.δ_i %	∑Av.δ_i %	Genus	Family	Order	Class	Phylum	Domain
Otu0107	0	1.25	0.74	1.1	0.87	62.58	unclass. *Rhodobacteraceae*	*Rhodobacteraceae*	*Rhodobacterales*	*Alphaproteobacteria*	*Proteobacteria*	*Bacteria*
Otu0583	0	1.17	0.71	4.08	0.83	63.4	unclass. *Flavobacteriaceae*	*Flavobacteriaceae*	*Flavobacteriales*	*Flavobacteria*	*Bacteroidetes*	*Bacteria*
Otu0123	1.08	0	0.69	1.18	0.81	64.21	unclass. *Gammaproteobacteria*	unclass. *Gammaproteobacteria*	unclass. *Gammaproteobacteria*	*Gammaproteobacteria*	*Proteobacteria*	*Bacteria*
Otu0549	1.26	0	0.67	0.49	0.78	64.99	unclass. *Bacteroidetes*	unclass. *Bacteroidetes*	unclass. *Bacteroidetes*	unclass. *Bacteroidetes*	*Bacteroidetes*	*Bacteria*
Otu0615	0	1.12	0.66	0.84	0.78	65.77	*Neptuniibacter*	*Oceanospirillaceae*	*Oceanospirillales*	*Gammaproteobacteria*	*Proteobacteria*	*Bacteria*
Otu0525	2.2	1.62	0.64	1.6	0.74	66.51	*Reinekea*	*Oceanospirillaceae*	*Oceanospirillales*	*Gammaproteobacteria*	*Proteobacteria*	*Bacteria*
Otu0489	0	1	0.61	6.3	0.71	67.22	unclass. *Vibrionaceae*	*Vibrionaceae*	*Vibrionales*	*Gammaproteobacteria*	*Proteobacteria*	*Bacteria*
Otu0535	0.48	0.75	0.6	0.78	0.7	67.92	*Loktanella*	*Rhodobacteraceae*	*Rhodobacterales*	*Alphaproteobacteria*	*Proteobacteria*	*Bacteria*
Otu0261	0.4	1.24	0.59	1.51	0.69	68.61	*Winogradskyella*	*Flavobacteriaceae*	*Flavobacteriales*	*Flavobacteria*	*Bacteroidetes*	*Bacteria*
Otu0578	0	0.97	0.58	1.69	0.67	69.28	*Winogradskyella*	*Flavobacteriaceae*	*Flavobacteriales*	*Flavobacteria*	*Bacteroidetes*	*Bacteria*
Otu0542	0.2	1.09	0.58	1.47	0.67	69.95	unclass. *Oceanospirillaceae*	*Oceanospirillaceae*	*Oceanospirillales*	*Gammaproteobacteria*	*Proteobacteria*	*Bacteria*
Otu0580	0	0.91	0.54	1.17	0.63	70.58	*Neptuniibacter*	*Oceanospirillaceae*	*Oceanospirillales*	*Gammaproteobacteria*	*Proteobacteria*	*Bacteria*
Otu0029	1	0	0.53	0.49	0.62	71.2	unclass. *Bacteroidetes*	unclass. *Bacteroidetes*	unclass. *Bacteroidetes*	unclass. *Bacteroidetes*	*Bacteroidetes*	*Bacteria*
Otu0036	0.28	0.95	0.53	1.52	0.61	71.81	*Glaciecola*	*Alteromonadaceae*	*Alteromonadales*	*Gammaproteobacteria*	*Proteobacteria*	*Bacteria*
Otu0234	0	0.85	0.52	0.77	0.61	72.42	*Arcobacter*	*Campylobacteraceae*	*Campylobacterales*	*Epsilonproteobacteria*	*Proteobacteria*	*Bacteria*
Otu0548	0.98	0	0.52	0.49	0.6	73.02	unclass. *Bacteroidetes*	unclass. *Bacteroidetes*	unclass. *Bacteroidetes*	unclass. *Bacteroidetes*	*Bacteroidetes*	*Bacteria*
Otu0165	0	0.83	0.5	1.1	0.58	73.61	*Marinomonas*	*Oceanospirillaceae*	*Oceanospirillales*	*Gammaproteobacteria*	*Proteobacteria*	*Bacteria*
Otu0599	0	0.8	0.49	1.85	0.58	74.18	*Winogradskyella*	*Flavobacteriaceae*	*Flavobacteriales*	*Flavobacteria*	*Bacteroidetes*	*Bacteria*
Otu0232	0	0.8	0.49	0.97	0.57	74.76	unclass. *Rhodobacteraceae*	*Rhodobacteraceae*	*Rhodobacterales*	*Alphaproteobacteria*	*Proteobacteria*	*Bacteria*
Otu0527	0.83	0	0.49	1.12	0.57	75.33	*Idiomarina*	*Idiomarinaceae*	*Alteromonadales*	*Gammaproteobacteria*	*Proteobacteria*	*Bacteria*
Otu0533	0.55	0.95	0.48	1.23	0.56	75.89	*Nisaea*	*Rhodospirillaceae*	*Rhodospirillales*	*Alphaproteobacteria*	*Proteobacteria*	*Bacteria*
Otu0577	0	0.73	0.41	0.77	0.47	76.37	unclass. *Bacteroidetes*	unclass. *Bacteroidetes*	unclass. *Bacteroidetes*	unclass. *Bacteroidetes*	*Bacteroidetes*	*Bacteria*
Otu0346	0	0.68	0.41	1.14	0.47	76.84	unclass. *Bacteria*	unclass. *Bacteria*	unclass. *Bacteria*	unclass. *Bacteria*	unclass. *Bacteria*	*Bacteria*
Otu0010	0	0.68	0.38	0.78	0.44	77.29	unclass. *Flavobacteriaceae*	*Flavobacteriaceae*	*Flavobacteriales*	*Flavobacteria*	*Bacteroidetes*	*Bacteria*
Otu0103	0.2	0.6	0.38	0.87	0.44	77.73	*Pseudomonas*	*Pseudomonadaceae*	*Pseudomonadales*	*Gammaproteobacteria*	*Proteobacteria*	*Bacteria*
Otu0113	0	0.55	0.37	0.74	0.43	78.16	*Colwellia*	*Colwelliaceae*	*Alteromonadales*	*Gammaproteobacteria*	*Proteobacteria*	*Bacteria*
Otu0575	0	0.65	0.37	0.7	0.43	78.58	unclass. *Bacteroidetes*	unclass. *Bacteroidetes*	unclass. *Bacteroidetes*	unclass. *Bacteroidetes*	*Bacteroidetes*	*Bacteria*

OTU	Av.A$_i$ (serial)	Av.A$_i$ (initiall)	Av.δ$_i$	Av.δ$_i$/SD	Av.δ$_i$ %	∑Av.δ$_i$ %	Genus	Family	Order	Class	Phylum	Domain
Otu0607	0	0.55	0.35	0.72	0.41	78.99	unclass. *Flavobacteriaceae*	*Flavobacteriaceae*	*Flavobacteriales*	*Flavobacteria*	*Bacteroidetes*	*Bacteria*
Otu0244	0	0.55	0.35	0.72	0.41	79.4	unclass. *Rhodobacteraceae*	*Rhodobacteraceae*	*Rhodobacterales*	*Alphaproteobacteria*	*Proteobacteria*	*Bacteria*
Otu0597	0	0.55	0.35	0.73	0.4	79.8	*Arenibacter*	*Flavobacteriaceae*	*Flavobacteriales*	*Flavobacteria*	*Bacteroidetes*	*Bacteria*
Otu0547	0.2	0.48	0.32	0.84	0.37	80.18	*Phenylobacterium*	*Caulobacteraceae*	*Caulobacterales*	*Alphaproteobacteria*	*Proteobacteria*	*Bacteria*
Otu0538	0.48	0	0.31	0.79	0.36	80.54	*Oceaniserpentilla*	*Oceanospirillaceae*	*Oceanospirillales*	*Gammaproteobacteria*	*Proteobacteria*	*Bacteria*
Otu0180	0.48	0.2	0.31	0.89	0.36	80.9	*Reichenbachiella*	*Flammeovirgaceae*	*Sphingobacteriales*	*Sphingobacteria*	*Bacteroidetes*	*Bacteria*
Otu0584	0	0.55	0.31	0.74	0.36	81.26	unclass. *Proteobacteria*	unclass. *Proteobacteria*	unclass. *Proteobacteria*	unclass. *Proteobacteria*	*Proteobacteria*	*Bacteria*
Otu0608	0	0.48	0.31	0.75	0.36	81.61	unclass. *Flavobacteriaceae*	*Flavobacteriaceae*	*Flavobacteriales*	*Flavobacteria*	*Bacteroidetes*	*Bacteria*
Otu0324	0	0.48	0.3	0.78	0.35	81.96	*Nisaea*	*Rhodospirillaceae*	*Rhodospirillales*	*Alphaproteobacteria*	*Proteobacteria*	*Bacteria*
Otu0596	0	0.48	0.28	0.77	0.33	82.29	*Neptunomonas*	*Oceanospirillaceae*	*Oceanospirillales*	*Gammaproteobacteria*	*Proteobacteria*	*Bacteria*
Otu0529	0.48	0	0.27	0.78	0.32	82.61	unclass. *Sphingobacteriales*	unclass. *Sphingobacteriales*	*Sphingobacteriales*	*Sphingobacteria*	*Bacteroidetes*	*Bacteria*
Otu0423	0	0.48	0.27	0.77	0.32	82.92	unclass. *Flavobacteriaceae*	*Flavobacteriaceae*	*Flavobacteriales*	*Flavobacteria*	*Bacteroidetes*	*Bacteria*
Otu0595	0	0.48	0.27	0.77	0.32	83.24	unclass. *Legionellaceae*	*Legionellaceae*	*Legionellales*	*Gammaproteobacteria*	*Proteobacteria*	*Bacteria*
Otu0543	0	0.48	0.27	0.78	0.32	83.56	*Muricauda*	*Flavobacteriaceae*	*Flavobacteriales*	*Flavobacteria*	*Bacteroidetes*	*Bacteria*
Otu0632	0	0.49	0.27	0.49	0.31	83.87	unclass. *Flavobacteriaceae*	*Flavobacteriaceae*	*Flavobacteriales*	*Flavobacteria*	*Bacteroidetes*	*Bacteria*
Otu0550	0.49	0	0.26	0.49	0.3	84.17	unclass. *Flavobacteriales*	unclass. *Flavobacteriales*	*Flavobacteriales*	*Flavobacteria*	*Bacteroidetes*	*Bacteria*
Otu0555	0.2	0.4	0.25	0.86	0.29	84.46	*Maricaulis*	*Hyphomonadaceae*	*Caulobacterales*	*Alphaproteobacteria*	*Proteobacteria*	*Bacteria*
Otu0568	0	0.4	0.25	0.78	0.29	84.76	unclass. *Flavobacteriales*	unclass. *Flavobacteriales*	*Flavobacteriales*	*Flavobacteria*	*Bacteroidetes*	*Bacteria*
Otu0112	0	0.4	0.25	0.79	0.29	85.05	*Polaribacter*	*Flavobacteriaceae*	*Flavobacteriales*	*Flavobacteria*	*Bacteroidetes*	*Bacteria*
Otu0604	0	0.4	0.24	0.78	0.29	85.33	unclass. *Bacteroidetes*	unclass. *Bacteroidetes*	unclass. *Bacteroidetes*	unclass. *Bacteroidetes*	*Bacteroidetes*	*Bacteria*
Otu0434	0	0.4	0.24	0.78	0.28	85.61	*Maribacter*	*Flavobacteriaceae*	*Flavobacteriales*	*Flavobacteria*	*Bacteroidetes*	*Bacteria*
Otu0598	0	0.4	0.24	0.78	0.28	85.9	*Arcobacter*	*Campylobacteraceae*	*Campylobacterales*	*Epsilonproteobacteria*	*Proteobacteria*	*Bacteria*
Otu0534	0.4	0	0.24	0.79	0.28	86.18	unclass. *Alphaproteobacteria*	unclass. *Alphaproteobacteria*	unclass. *Alphaproteobacteria*	*Alphaproteobacteria*	*Proteobacteria*	*Bacteria*
Otu0564	0	0.4	0.23	0.49	0.27	86.45	unclass. *Bacteroidetes*	unclass. *Bacteroidetes*	unclass. *Bacteroidetes*	unclass. *Bacteroidetes*	*Bacteroidetes*	*Bacteria*
Otu0616	0	0.4	0.23	0.49	0.27	86.72	unclass. *Bacteria*	unclass. *Bacteria*	unclass. *Bacteria*	unclass. *Bacteria*	unclass. *Bacteria*	*Bacteria*
Otu0536	0	0.4	0.23	0.79	0.27	86.99	*Maribacter*	*Flavobacteriaceae*	*Flavobacteriales*	*Flavobacteria*	*Bacteroidetes*	*Bacteria*
Otu0582	0	0.4	0.23	0.79	0.27	87.26	unclass. *Rhodobacteraceae*	*Rhodobacteraceae*	*Rhodobacterales*	*Alphaproteobacteria*	*Proteobacteria*	*Bacteria*

OTU	Av.A_i (serial)	Av.A_i (initiall)	Av.δ_i	Av.δ_i/SD	Av.δ_i %	∑Av.δ_i %	Genus	Family	Order	Class	Phylum	Domain
Otu0593	0	0.4	0.23	0.79	0.27	87.53	*Psychroserpens*	*Flavobacteriaceae*	*Flavobacteriales*	*Flavobacteria*	*Bacteroidetes*	*Bacteria*
Otu0569	0	0.4	0.23	0.49	0.27	87.8	unclass. *Proteobacteria*	unclass. *Proteobacteria*	unclass. *Proteobacteria*	unclass. *Proteobacteria*	*Proteobacteria*	*Bacteria*
Otu0263	0	0.4	0.23	0.79	0.26	88.06	*Krokinobacter*	*Flavobacteriaceae*	*Flavobacteriales*	*Flavobacteria*	*Bacteroidetes*	*Bacteria*
Otu0320	0	0.4	0.23	0.79	0.26	88.33	*Pseudoalteromonas*	*Pseudoalteromonadaceae*	*Alteromonadales*	*Gammaproteobacteria*	*Proteobacteria*	*Bacteria*
Otu0628	0	0.4	0.23	0.79	0.26	88.59	*Lacinutrix*	*Flavobacteriaceae*	*Flavobacteriales*	*Flavobacteria*	*Bacteroidetes*	*Bacteria*
Otu0111	0	0.4	0.22	0.79	0.26	88.85	*Marinomonas*	*Oceanospirillaceae*	*Oceanospirillales*	*Gammaproteobacteria*	*Proteobacteria*	*Bacteria*
Otu0500	0	0.4	0.22	0.79	0.26	89.11	*Marinobacter*	*Alteromonadaceae*	*Alteromonadales*	*Gammaproteobacteria*	*Proteobacteria*	*Bacteria*
Otu0579	0	0.35	0.2	0.49	0.23	89.35	unclass. *Bacteroidetes*	unclass. *Bacteroidetes*	unclass. *Bacteroidetes*	unclass. *Bacteroidetes*	*Bacteroidetes*	*Bacteria*
Otu0544	0.2	0.2	0.2	0.66	0.23	89.58	*Alteromonas*	*Alteromonadaceae*	*Alteromonadales*	*Gammaproteobacteria*	*Proteobacteria*	*Bacteria*
Otu0561	0.2	0.2	0.2	0.66	0.23	89.81	*Cellulophaga*	*Flavobacteriaceae*	*Flavobacteriales*	*Flavobacteria*	*Bacteroidetes*	*Bacteria*
Otu0005	0.2	0.2	0.19	0.66	0.23	90.03	*Pelagibacter*	SAR11-clade	*Rickettsiales*	*Alphaproteobacteria*	*Proteobacteria*	*Bacteria*
