# Supplementary material for: Small Changes in pH Have Direct Effects on Marine Bacterial Community Composition: A Microcosm Approach
Source: PLoS One. 2012 Oct 11;7(10):e47035. doi: 10.1371/journal.pone.0047035 (PMC3469576; doi:10.1371/journal.pone.0047035)
Supplement: Table S5 — Results of the SIMPER analysis giving the dissimilarities between the pH levels in situ and 7.67 of ‘season’-‘dilution’ combinations significantly influenced by ‘pH’ according to PERMANOVA. Displayed are the OTUs (16S ribosomal amplicon pyrosequencing) that predominantly contributed to 90% of to the total dissimilarity. Av.Ai: average abundance of the i th species over all samples of the treatment (no: ‘no dilution’, serial: ‘serial dilution’, initial: ‘initial dilution’), Av.δi: average contribution of the i th species to the total dissimilarity, Av.δi/SD: the average value of the i th species as a discriminating species, Av.δi%: average percentage contribution of the i th species to the total dissimilarity, ΣAv.δi%: average cumulative contribution to the total dissimilarity. (PDF) [file pone.0047035.s009.pdf]

Table S5. Results of the SIMPER analysis giving the dissimilarities between the pH levels *in situ* and 7.67 of 'season'-'dilution' combinations significantly influenced by 'pH' according to PERMANOVA.

Displayed are the OTUs (16S ribosomal amplicon pyrosequencing) that predominantly contributed to 90% of to the total dissimilarity. $Av.A_i$: average abundance of the i th species over all samples of the treatment (no: 'no dilution', serial: 'serial dilution', initial: 'initial dilution'), $Av.\delta_i$: average contribution of the i th species to the total dissimilarity, $Av.\delta_i/SD$: the average value of the i th species as a discriminating species, $Av.\delta_i\%$: average percentage contribution of the i th species to the total dissimilarity, $\sum Av.\delta_i\%$: average cumulative contribution to the total dissimilarity.

OTU	$Av.A_i$ (*pH in situ*)	$Av.A_i$ (pH 7.67)	$Av.\delta_i$	$Av.\delta_i/SD$	$Av.\delta_i$ %	$\sum Av.\delta_i$ %	Genus	Family	Order	Class	Phylum	Domain
Spring 'no dilution' (average dissimilarity: 57.3%)												
Otu0005	9.45	4.62	1.95	1.85	3.4	3.4	*Pelagibacter*	SAR11-clade	*Rickettsiales*	*Alphaproteobacteria*	*Proteobacteria*	*Bacteria*
Otu0029	5.69	1.39	1.6	2.13	2.79	6.19	unclass. *Bacteroidetes*	unclass. *Bacteroidetes*	unclass. *Bacteroidetes*	unclass. *Bacteroidetes*	*Bacteroidetes*	*Bacteria*
Otu0008	5.12	0.98	1.53	2.41	2.67	8.86	unclass. *Gammaproteobacteria*	unclass. *Gammaproteobacteria*	unclass. *Gammaproteobacteria*	*Gammaproteobacteria*	*Proteobacteria*	*Bacteria*
Otu0018	1.91	5.96	1.48	4.11	2.59	11.45	unclass. *Puniceicoccaceae*	*Puniceicoccaceae*	*Puniceicoccales*	*Opitutae*	*Verrucomicrobia*	*Bacteria*
Otu0001	6.06	4.13	1.33	1.95	2.32	13.76	unclass. *Flavobacteriaceae*	*Flavobacteriaceae*	*Flavobacteriales*	*Flavobacteria*	*Bacteroidetes*	*Bacteria*
Otu0056	1.59	4.87	1.2	4.97	2.09	15.85	unclass. *Gammaproteobacteria*	unclass. *Gammaproteobacteria*	unclass. *Gammaproteobacteria*	*Gammaproteobacteria*	*Proteobacteria*	*Bacteria*
Otu0012	4.52	1.48	1.16	1.9	2.02	17.87	unclass. *Comamonadaceae*	*Comamonadaceae*	*Burkholderiales*	*Betaproteobacteria*	*Proteobacteria*	*Bacteria*
Otu0010	3.32	5.77	1.12	1.41	1.95	19.83	unclass. *Flavobacteriaceae*	*Flavobacteriaceae*	*Flavobacteriales*	*Flavobacteria*	*Bacteroidetes*	*Bacteria*
Otu0013	2.74	5.62	1.06	2.51	1.85	21.68	unclass. *Flavobacteriaceae*	*Flavobacteriaceae*	*Flavobacteriales*	*Flavobacteria*	*Bacteroidetes*	*Bacteria*
Otu0002	6.43	7.01	1.05	1.5	1.82	23.5	unclass. *Flavobacteriaceae*	*Flavobacteriaceae*	*Flavobacteriales*	*Flavobacteria*	*Bacteroidetes*	*Bacteria*
Otu0022	5.16	2.41	1	2.36	1.74	25.24	unclass. *Betaproteobacteria*	unclass. *Betaproteobacteria*	unclass. *Betaproteobacteria*	*Betaproteobacteria*	*Proteobacteria*	*Bacteria*
Otu0025	0.2	2.74	0.96	1.73	1.67	26.91	*Polaribacter*	*Flavobacteriaceae*	*Flavobacteriales*	*Flavobacteria*	*Bacteroidetes*	*Bacteria*

OTU	Av.A_i (*pH in situ*)	Av.A_i (pH 7.67)	Av.δ_i	Av.δ_i/SD	Av.δ_i %	∑Av.δ_i %	Genus	Family	Order	Class	Phylum	Domain
Otu0134	7.21	5.85	0.82	1.57	1.42	28.34	unclass. *Gammaproteobacteria*	unclass. *Gammaproteobacteria*	unclass. *Gammaproteobacteria*	*Gammaproteobacteria*	*Proteobacteria*	*Bacteria*
Otu0139	1.76	3.91	0.78	1.76	1.37	29.7	unclass. *Gammaproteobacteria*	unclass. *Gammaproteobacteria*	unclass. *Gammaproteobacteria*	*Gammaproteobacteria*	*Proteobacteria*	*Bacteria*
Otu0014	3.76	3.88	0.77	1.35	1.34	31.04	*Reinekea*	*Oceanospirillaceae*	*Oceanospirillales*	*Gammaproteobacteria*	*Proteobacteria*	*Bacteria*
Otu0007	3.2	4.03	0.69	1.1	1.2	32.24	unclass. *Flavobacteriales*	unclass. *Flavobacteriales*	*Flavobacteriales*	*Flavobacteria*	*Bacteroidetes*	*Bacteria*
Otu0019	0.48	2.32	0.68	1.96	1.18	33.42	unclass. *Gammaproteobacteria*	unclass. *Gammaproteobacteria*	unclass. *Gammaproteobacteria*	*Gammaproteobacteria*	*Proteobacteria*	*Bacteria*
Otu0062	0.2	1.93	0.67	1.49	1.17	34.59	*Haliea*	*Alteromonadaceae*	*Alteromonadales*	*Gammaproteobacteria*	*Proteobacteria*	*Bacteria*
Otu0143	0.28	2.11	0.67	2.55	1.16	35.76	unclass. *Gammaproteobacteria*	unclass. *Gammaproteobacteria*	unclass. *Gammaproteobacteria*	*Gammaproteobacteria*	*Proteobacteria*	*Bacteria*
Otu0232	0	1.66	0.61	5.32	1.06	36.82	unclass. *Rhodobacteraceae*	*Rhodobacteraceae*	*Rhodobacterales*	*Alphaproteobacteria*	*Proteobacteria*	*Bacteria*
Otu0151	0.6	2.27	0.6	2.42	1.04	37.86	*Rhodococcus*	*Nocardiaceae*	*Actinomycetales*	*Actinobacteria*	*Actinobacteria*	*Bacteria*
Otu0135	3.24	1.86	0.56	1.43	0.98	38.84	unclass. *Gammaproteobacteria*	unclass. *Gammaproteobacteria*	unclass. *Gammaproteobacteria*	*Gammaproteobacteria*	*Proteobacteria*	*Bacteria*
Otu0248	0.2	1.5	0.56	0.78	0.97	39.82	unclass. *Flavobacteriaceae*	*Flavobacteriaceae*	*Flavobacteriales*	*Flavobacteria*	*Bacteroidetes*	*Bacteria*
Otu0070	0	1.43	0.51	4.38	0.9	40.71	*Winogradskyella*	*Flavobacteriaceae*	*Flavobacteriales*	*Flavobacteria*	*Bacteroidetes*	*Bacteria*
Otu0015	1.5	0.2	0.5	1.62	0.88	41.59	*Polaribacter*	*Flavobacteriaceae*	*Flavobacteriales*	*Flavobacteria*	*Bacteroidetes*	*Bacteria*
Otu0144	0.2	1.49	0.48	2.13	0.83	42.42	*Lewinella*	*Saprospiraceae*	*Sphingobacteriales*	*Sphingobacteria*	*Bacteroidetes*	*Bacteria*
Otu0041	1.76	2.71	0.47	1.31	0.81	43.23	*Sulfitobacter*	*Rhodobacteraceae*	*Rhodobacterales*	*Alphaproteobacteria*	*Proteobacteria*	*Bacteria*
Otu0020	1.78	2.15	0.46	1.3	0.81	44.04	unclass. *Flammeovirgaceae*	*Flammeovirgaceae*	*Sphingobacteriales*	*Sphingobacteria*	*Bacteroidetes*	*Bacteria*
Otu0006	0.88	1.36	0.46	1.56	0.8	44.83	*Polaribacter*	*Flavobacteriaceae*	*Flavobacteriales*	*Flavobacteria*	*Bacteroidetes*	*Bacteria*
Otu0055	1.08	2.31	0.45	1.73	0.78	45.62	*Colwellia*	*Colwelliaceae*	*Alteromonadales*	*Gammaproteobacteria*	*Proteobacteria*	*Bacteria*
Otu0092	0.2	1.31	0.43	1.8	0.75	46.37	unclass. *Bacteroidetes*	unclass. *Bacteroidetes*	unclass. *Bacteroidetes*	unclass. *Bacteroidetes*	*Bacteroidetes*	*Bacteria*
Otu0017	1	0.8	0.42	1.25	0.73	47.1	unclass. *Flavobacteriales*	unclass. *Flavobacteriales*	*Flavobacteriales*	*Flavobacteria*	*Bacteroidetes*	*Bacteria*
Otu0027	1.37	0.48	0.41	1.35	0.71	47.81	*Pseudoalteromonas*	*Pseudoalteromonadaceae*	*Alteromonadales*	*Gammaproteobacteria*	*Proteobacteria*	*Bacteria*
Otu0052	1.49	0.4	0.4	1.38	0.7	48.51	unclass. *Betaproteobacteria*	unclass. *Betaproteobacteria*	unclass. *Betaproteobacteria*	*Betaproteobacteria*	*Proteobacteria*	*Bacteria*
Otu0037	0	1.08	0.4	4.18	0.7	49.21	*Marinobacter*	*Alteromonadaceae*	*Alteromonadales*	*Gammaproteobacteria*	*Proteobacteria*	*Bacteria*
Otu0148	0	1.08	0.39	1.64	0.68	49.89	unclass. *Sphingobacteriales*	unclass. *Sphingobacteriales*	*Sphingobacteriales*	*Sphingobacteria*	*Bacteroidetes*	*Bacteria*
Otu0160	0.57	1.34	0.38	1.45	0.66	50.55	*Colwellia*	*Colwelliaceae*	*Alteromonadales*	*Gammaproteobacteria*	*Proteobacteria*	*Bacteria*
Otu0145	0.2	1.17	0.37	1.61	0.64	51.19	unclass. *Gammaproteobacteria*	unclass. *Gammaproteobacteria*	unclass. *Gammaproteobacteria*	*Gammaproteobacteria*	*Proteobacteria*	*Bacteria*
Otu0035	0.97	1.91	0.36	1.39	0.63	51.83	*Colwellia*	*Colwelliaceae*	*Alteromonadales*	*Gammaproteobacteria*	*Proteobacteria*	*Bacteria*

OTU	Av.A_i (*pH in situ*)	Av.A_i (pH 7.67)	Av.δ_i	Av.δ_i/SD	Av.δ_i %	∑Av.δ_i %	Genus	Family	Order	Class	Phylum	Domain
Otu0213	0	0.97	0.36	1.78	0.62	52.44	unclass. *Gammaproteobacteria*	unclass. *Gammaproteobacteria*	unclass. *Gammaproteobacteria*	*Gammaproteobacteria*	*Proteobacteria*	*Bacteria*
Otu0150	0	0.97	0.35	1.85	0.61	53.06	unclass. *Bacteria*	unclass. *Bacteria*	unclass. *Bacteria*	unclass. *Bacteria*	unclass. *Bacteria*	*Bacteria*
Otu0169	0.2	1.17	0.35	2.1	0.61	53.67	unclass. *Bacteria*	unclass. *Bacteria*	unclass. *Bacteria*	unclass. *Bacteria*	unclass. *Bacteria*	*Bacteria*
Otu0069	0	0.97	0.34	0.98	0.6	54.27	unclass. *Bacteria*	unclass. *Bacteria*	unclass. *Bacteria*	unclass. *Bacteria*	unclass. *Bacteria*	*Bacteria*
Otu0237	0	0.95	0.33	1.76	0.58	54.85	unclass. *Flavobacteriaceae*	*Flavobacteriaceae*	*Flavobacteriales*	*Flavobacteria*	*Bacteroidetes*	*Bacteria*
Otu0136	0.68	0.8	0.32	1.19	0.57	55.42	unclass. *Bacteroidetes*	unclass. *Bacteroidetes*	unclass. *Bacteroidetes*	unclass. *Bacteroidetes*	*Bacteroidetes*	*Bacteria*
Otu0177	0.2	0.83	0.3	1.13	0.53	55.94	*Haliea*	*Alteromonadaceae*	*Alteromonadales*	*Gammaproteobacteria*	*Proteobacteria*	*Bacteria*
Otu0119	0.2	0.95	0.3	1.45	0.52	56.46	*Colwellia*	*Colwelliaceae*	*Alteromonadales*	*Gammaproteobacteria*	*Proteobacteria*	*Bacteria*
Otu0146	0.2	0.95	0.3	1.38	0.51	56.98	*Reichenbachiella*	*Flammeovirgaceae*	*Sphingobacteriales*	*Sphingobacteria*	*Bacteroidetes*	*Bacteria*
Otu0149	0	0.77	0.29	1.16	0.51	57.49	unclass. *Gammaproteobacteria*	unclass. *Gammaproteobacteria*	unclass. *Gammaproteobacteria*	*Gammaproteobacteria*	*Proteobacteria*	*Bacteria*
Otu0138	0	0.84	0.29	0.79	0.51	58	unclass. *Flavobacteriaceae*	*Flavobacteriaceae*	*Flavobacteriales*	*Flavobacteria*	*Bacteroidetes*	*Bacteria*
Otu0057	0	0.83	0.29	1.14	0.51	58.5	*Ulvibacter*	*Flavobacteriaceae*	*Flavobacteriales*	*Flavobacteria*	*Bacteroidetes*	*Bacteria*
Otu0140	0	0.83	0.29	1.16	0.5	59.01	*Oleispira*	*Oceanospirillaceae*	*Oceanospirillales*	*Gammaproteobacteria*	*Proteobacteria*	*Bacteria*
Otu0293	0.2	0.83	0.29	1.15	0.5	59.51	*Moritella*	*Moritellaceae*	*Alteromonadales*	*Gammaproteobacteria*	*Proteobacteria*	*Bacteria*
Otu0155	0	0.8	0.29	1.1	0.5	60.01	unclass. *Flavobacteriaceae*	*Flavobacteriaceae*	*Flavobacteriales*	*Flavobacteria*	*Bacteroidetes*	*Bacteria*
Otu0009	0.63	0.77	0.29	1.2	0.5	60.51	unclass. *Rhodobacteraceae*	*Rhodobacteraceae*	*Rhodobacterales*	*Alphaproteobacteria*	*Proteobacteria*	*Bacteria*
Otu0187	0	0.8	0.28	1.95	0.49	61	*Lentisphaera*	*Lentisphaeraceae*	*Lentisphaerales*	*Lentisphaeria*	*Lentisphaerae*	*Bacteria*
Otu0083	0	0.77	0.28	1.13	0.49	61.49	unclass. *Rhodobacteraceae*	*Rhodobacteraceae*	*Rhodobacterales*	*Alphaproteobacteria*	*Proteobacteria*	*Bacteria*
Otu0174	0.2	0.88	0.28	1.46	0.48	61.98	unclass. *Flavobacteriaceae*	*Flavobacteriaceae*	*Flavobacteriales*	*Flavobacteria*	*Bacteroidetes*	*Bacteria*
Otu0046	0.28	0.75	0.27	1.15	0.47	62.45	unclass. *Flavobacteriaceae*	*Flavobacteriaceae*	*Flavobacteriales*	*Flavobacteria*	*Bacteroidetes*	*Bacteria*
Otu0036	0.75	0	0.27	1.09	0.47	62.92	*Glaciecola*	*Alteromonadaceae*	*Alteromonadales*	*Gammaproteobacteria*	*Proteobacteria*	*Bacteria*
Otu0122	0	0.75	0.27	1.09	0.46	63.39	*Glaciecola*	*Alteromonadaceae*	*Alteromonadales*	*Gammaproteobacteria*	*Proteobacteria*	*Bacteria*
Otu0164	0.4	0.83	0.27	1.23	0.46	63.85	*Croceibacter*	*Flavobacteriaceae*	*Flavobacteriales*	*Flavobacteria*	*Bacteroidetes*	*Bacteria*
Otu0080	0.28	0.63	0.25	0.89	0.44	64.29	*Polaribacter*	*Flavobacteriaceae*	*Flavobacteriales*	*Flavobacteria*	*Bacteroidetes*	*Bacteria*
Otu0158	0.2	0.75	0.25	1.11	0.44	64.74	unclass. *Proteobacteria*	unclass. *Proteobacteria*	unclass. *Proteobacteria*	unclass. *Proteobacteria*	*Proteobacteria*	*Bacteria*
Otu0042	0.4	0.95	0.25	1.21	0.44	65.17	*Rhodococcus*	*Nocardiaceae*	*Actinomycetales*	*Actinobacteria*	*Actinobacteria*	*Bacteria*
Otu0163	0.2	0.75	0.25	1.09	0.44	65.61	unclass. *Gammaproteobacteria*	unclass. *Gammaproteobacteria*	unclass. *Gammaproteobacteria*	*Gammaproteobacteria*	*Proteobacteria*	*Bacteria*

OTU	Av.A_i (*pH in situ*)	Av.A_i (pH 7.67)	Av.δ_i	Av.δ_i/SD	Av.δ_i %	∑Av.δ_i %	Genus	Family	Order	Class	Phylum	Domain
Otu0157	0.4	0.77	0.25	1.19	0.44	66.05	unclass. *Gammaproteobacteria*	unclass. *Gammaproteobacteria*	unclass. *Gammaproteobacteria*	*Gammaproteobacteria*	*Proteobacteria*	*Bacteria*
Otu0391	0	0.68	0.24	1.15	0.42	66.47	unclass. *Gammaproteobacteria*	unclass. *Gammaproteobacteria*	unclass. *Gammaproteobacteria*	*Gammaproteobacteria*	*Proteobacteria*	*Bacteria*
Otu0257	0	0.68	0.24	1.14	0.41	66.88	*Salinisphaera*	*Salinisphaeraceae*	*Salinisphaerales*	*Gammaproteobacteria*	*Proteobacteria*	*Bacteria*
Otu0229	0.2	0.68	0.23	1.14	0.41	67.29	unclass. *Saprospiraceae*	*Saprospiraceae*	*Sphingobacteriales*	*Sphingobacteria*	*Bacteroidetes*	*Bacteria*
Otu0199	0	0.6	0.22	1.19	0.38	67.68	unclass. *Flavobacteriaceae*	*Flavobacteriaceae*	*Flavobacteriales*	*Flavobacteria*	*Bacteroidetes*	*Bacteria*
Otu0044	1.63	1.81	0.22	1.4	0.38	68.05	unclass. *Gammaproteobacteria*	unclass. *Gammaproteobacteria*	unclass. *Gammaproteobacteria*	*Gammaproteobacteria*	*Proteobacteria*	*Bacteria*
Otu0003	0.28	0.48	0.21	0.86	0.37	68.42	unclass. *Rhodobacteraceae*	*Rhodobacteraceae*	*Rhodobacterales*	*Alphaproteobacteria*	*Proteobacteria*	*Bacteria*
Otu0011	0.48	0.28	0.21	0.89	0.37	68.79	unclass. *Flavobacteriaceae*	*Flavobacteriaceae*	*Flavobacteriales*	*Flavobacteria*	*Bacteroidetes*	*Bacteria*
Otu0118	0.4	0.48	0.21	1.01	0.36	69.15	*Colwellia*	*Colwelliaceae*	*Alteromonadales*	*Gammaproteobacteria*	*Proteobacteria*	*Bacteria*
Otu0137	0.28	0.48	0.2	0.89	0.35	69.5	unclass. *Bacteroidetes*	unclass. *Bacteroidetes*	unclass. *Bacteroidetes*	unclass. *Bacteroidetes*	*Bacteroidetes*	*Bacteria*
Otu0051	0.4	0.48	0.2	1.01	0.35	69.86	unclass. *Flavobacteriaceae*	*Flavobacteriaceae*	*Flavobacteriales*	*Flavobacteria*	*Bacteroidetes*	*Bacteria*
Otu0032	0.55	0	0.2	0.75	0.35	70.2	unclass. *Gammaproteobacteria*	unclass. *Gammaproteobacteria*	unclass. *Gammaproteobacteria*	*Gammaproteobacteria*	*Proteobacteria*	*Bacteria*
Otu0265	0	0.55	0.19	0.76	0.34	70.54	unclass. *Bacteria*	unclass. *Bacteria*	unclass. *Bacteria*	unclass. *Bacteria*	unclass. *Bacteria*	*Bacteria*
Otu0113	0.6	0.4	0.19	1.01	0.33	70.87	*Colwellia*	*Colwelliaceae*	*Alteromonadales*	*Gammaproteobacteria*	*Proteobacteria*	*Bacteria*
Otu0251	0	0.55	0.19	0.77	0.33	71.2	unclass. *Bacteroidetes*	unclass. *Bacteroidetes*	unclass. *Bacteroidetes*	unclass. *Bacteroidetes*	*Bacteroidetes*	*Bacteria*
Otu0023	0	0.48	0.19	0.76	0.33	71.53	unclass. *Verrucomicrobiales*	unclass. *Verrucomicrobiales*	*Verrucomicrobiales*	*Verrucomicrobiae*	*Verrucomicrobia*	*Bacteria*
Otu0413	0	0.55	0.18	0.76	0.32	71.85	*Eudoraea*	*Flavobacteriaceae*	*Flavobacteriales*	*Flavobacteria*	*Bacteroidetes*	*Bacteria*
Otu0175	0.6	0.6	0.18	0.94	0.31	72.16	unclass. *Flavobacteriaceae*	*Flavobacteriaceae*	*Flavobacteriales*	*Flavobacteria*	*Bacteroidetes*	*Bacteria*
Otu0202	0	0.48	0.18	0.77	0.31	72.47	unclass. *Alteromonadaceae*	*Alteromonadaceae*	*Alteromonadales*	*Gammaproteobacteria*	*Proteobacteria*	*Bacteria*
Otu0068	0.48	0	0.17	0.78	0.3	72.77	*Pelagibacter*	SAR11-clade	*Rickettsiales*	*Alphaproteobacteria*	*Proteobacteria*	*Bacteria*
Otu0171	0	0.48	0.17	0.78	0.3	73.07	unclass. *Gammaproteobacteria*	unclass. *Gammaproteobacteria*	unclass. *Gammaproteobacteria*	*Gammaproteobacteria*	*Proteobacteria*	*Bacteria*
Otu0093	0	0.48	0.17	0.79	0.29	73.35	*Aestuariicola*	*Flavobacteriaceae*	*Flavobacteriales*	*Flavobacteria*	*Bacteroidetes*	*Bacteria*
Otu0173	0	0.48	0.17	0.79	0.29	73.64	unclass. *Alteromonadaceae*	*Alteromonadaceae*	*Alteromonadales*	*Gammaproteobacteria*	*Proteobacteria*	*Bacteria*
Otu0389	0	0.48	0.16	0.78	0.29	73.93	unclass. *Flavobacteriaceae*	*Flavobacteriaceae*	*Flavobacteriales*	*Flavobacteria*	*Bacteroidetes*	*Bacteria*
Otu0191	0.2	0.4	0.16	0.86	0.29	74.22	unclass. *Proteobacteria*	unclass. *Proteobacteria*	unclass. *Proteobacteria*	unclass. *Proteobacteria*	*Proteobacteria*	*Bacteria*
Otu0200	0	0.48	0.16	0.79	0.28	74.5	unclass. *Saprospiraceae*	*Saprospiraceae*	*Sphingobacteriales*	*Sphingobacteria*	*Bacteroidetes*	*Bacteria*
Otu0159	0.4	0.2	0.16	0.86	0.28	74.78	unclass. *Saprospiraceae*	*Saprospiraceae*	*Sphingobacteriales*	*Sphingobacteria*	*Bacteroidetes*	*Bacteria*

OTU	Av.A_i (*pH in situ*)	Av.A_i (pH 7.67)	Av.δ_i	Av.δ_i/SD	Av.δ_i %	∑Av.δ_i %	Genus	Family	Order	Class	Phylum	Domain
Otu0290	0.28	0.28	0.16	0.67	0.28	75.07	unclass. *Flavobacteriaceae*	*Flavobacteriaceae*	*Flavobacteriales*	*Flavobacteria*	*Bacteroidetes*	*Bacteria*
Otu0034	0	0.49	0.16	0.49	0.28	75.35	unclass. *Bacteria*	unclass. *Bacteria*	unclass. *Bacteria*	unclass. *Bacteria*	unclass. *Bacteria*	*Bacteria*
Otu0141	0.4	0.2	0.16	0.86	0.28	75.63	unclass. *Bacteroidetes*	unclass. *Bacteroidetes*	unclass. *Bacteroidetes*	unclass. *Bacteroidetes*	*Bacteroidetes*	*Bacteria*
Otu0192	0.2	0.4	0.16	0.86	0.27	75.9	unclass. *Gammaproteobacteria*	unclass. *Gammaproteobacteria*	unclass. *Gammaproteobacteria*	*Gammaproteobacteria*	*Proteobacteria*	*Bacteria*
Otu0409	0	0.45	0.16	0.49	0.27	76.17	unclass. *Flavobacteriales*	unclass. *Flavobacteriales*	*Flavobacteriales*	*Flavobacteria*	*Bacteroidetes*	*Bacteria*
Otu0254	0	0.4	0.15	0.79	0.27	76.44	unclass. *Flavobacteriaceae*	*Flavobacteriaceae*	*Flavobacteriales*	*Flavobacteria*	*Bacteroidetes*	*Bacteria*
Otu0227	0.4	0	0.15	0.8	0.26	76.7	unclass. *Gammaproteobacteria*	unclass. *Gammaproteobacteria*	unclass. *Gammaproteobacteria*	*Gammaproteobacteria*	*Proteobacteria*	*Bacteria*
Otu0133	0.4	0	0.15	0.8	0.26	76.95	*Colwellia*	*Colwelliaceae*	*Alteromonadales*	*Gammaproteobacteria*	*Proteobacteria*	*Bacteria*
Otu0167	0.28	0.2	0.15	0.69	0.26	77.21	unclass. *Rhodobacteraceae*	*Rhodobacteraceae*	*Rhodobacterales*	*Alphaproteobacteria*	*Proteobacteria*	*Bacteria*
Otu0124	0	0.4	0.15	0.8	0.26	77.47	*Loktanella*	*Rhodobacteraceae*	*Rhodobacterales*	*Alphaproteobacteria*	*Proteobacteria*	*Bacteria*
Otu0196	0	0.4	0.15	0.8	0.26	77.73	unclass. *Flavobacteriaceae*	*Flavobacteriaceae*	*Flavobacteriales*	*Flavobacteria*	*Bacteroidetes*	*Bacteria*
Otu0147	0.4	0	0.15	0.8	0.26	77.98	*Dasania*	*Pseudomonadales_incertae_sedis*	*Pseudomonadales*	*Gammaproteobacteria*	*Proteobacteria*	*Bacteria*
Otu0313	0	0.4	0.14	0.8	0.25	78.23	unclass. *Gammaproteobacteria*	unclass. *Gammaproteobacteria*	unclass. *Gammaproteobacteria*	*Gammaproteobacteria*	*Proteobacteria*	*Bacteria*
Otu0419	0	0.4	0.14	0.8	0.25	78.49	unclass. *Bacteria*	unclass. *Bacteria*	unclass. *Bacteria*	unclass. *Bacteria*	unclass. *Bacteria*	*Bacteria*
Otu0341	0	0.4	0.14	0.8	0.25	78.73	unclass. *Gammaproteobacteria*	unclass. *Gammaproteobacteria*	unclass. *Gammaproteobacteria*	*Gammaproteobacteria*	*Proteobacteria*	*Bacteria*
Otu0355	0	0.4	0.14	0.8	0.25	78.98	unclass. *Sphingobacteriales*	unclass. *Sphingobacteriales*	*Sphingobacteriales*	*Sphingobacteria*	*Bacteroidetes*	*Bacteria*
Otu0363	0	0.4	0.14	0.8	0.25	79.23	unclass. *Flavobacteriaceae*	*Flavobacteriaceae*	*Flavobacteriales*	*Flavobacteria*	*Bacteroidetes*	*Bacteria*
Otu0162	0	0.4	0.14	0.8	0.24	79.47	*Marinoscillum*	*Flammeovirgaceae*	*Sphingobacteriales*	*Sphingobacteria*	*Bacteroidetes*	*Bacteria*
Otu0394	0	0.4	0.14	0.8	0.24	79.71	unclass. *Bacteria*	unclass. *Bacteria*	unclass. *Bacteria*	unclass. *Bacteria*	unclass. *Bacteria*	*Bacteria*
Otu0392	0	0.4	0.14	0.8	0.24	79.95	unclass. *Bacteria*	unclass. *Bacteria*	unclass. *Bacteria*	unclass. *Bacteria*	unclass. *Bacteria*	*Bacteria*
Otu0249	0.2	0.2	0.12	0.67	0.21	80.16	unclass. *Bacteria*	unclass. *Bacteria*	unclass. *Bacteria*	unclass. *Bacteria*	unclass. *Bacteria*	*Bacteria*
Otu0256	0.2	0.2	0.12	0.67	0.2	80.36	*Glaciecola*	*Alteromonadaceae*	*Alteromonadales*	*Gammaproteobacteria*	*Proteobacteria*	*Bacteria*
Otu0245	0.2	0.2	0.12	0.67	0.2	80.57	unclass. *Flavobacteriales*	unclass. *Flavobacteriales*	*Flavobacteriales*	*Flavobacteria*	*Bacteroidetes*	*Bacteria*
Otu0271	0.2	0.2	0.12	0.67	0.2	80.77	unclass. *Proteobacteria*	unclass. *Proteobacteria*	unclass. *Proteobacteria*	unclass. *Proteobacteria*	*Proteobacteria*	*Bacteria*
Otu0049	0	0.28	0.12	0.49	0.2	80.97	unclass. *Bacteria*	unclass. *Bacteria*	unclass. *Bacteria*	unclass. *Bacteria*	unclass. *Bacteria*	*Bacteria*
Otu0170	0.2	0.2	0.12	0.67	0.2	81.18	unclass. *Bacteria*	unclass. *Bacteria*	unclass. *Bacteria*	unclass. *Bacteria*	unclass. *Bacteria*	*Bacteria*
Otu0261	0.2	0.2	0.12	0.67	0.2	81.38	*Winogradskyella*	*Flavobacteriaceae*	*Flavobacteriales*	*Flavobacteria*	*Bacteroidetes*	*Bacteria*

OTU	Av.A_i (*pH in situ*)	Av.A_i (pH 7.67)	Av.δ_i	Av.δ_i/SD	Av.δ_i %	∑Av.δ_i %	Genus	Family	Order	Class	Phylum	Domain
Otu0222	0	0.35	0.11	0.49	0.2	81.58	*Haliea*	*Alteromonadaceae*	*Alteromonadales*	*Gammaproteobacteria*	*Proteobacteria*	*Bacteria*
Otu0252	0	0.35	0.11	0.49	0.2	81.78	*Croceibacter*	*Flavobacteriaceae*	*Flavobacteriales*	*Flavobacteria*	*Bacteroidetes*	*Bacteria*
Otu0427	0	0.35	0.11	0.49	0.2	81.98	unclass. *Flavobacteriaceae*	*Flavobacteriaceae*	*Flavobacteriales*	*Flavobacteria*	*Bacteroidetes*	*Bacteria*
Otu0439	0	0.35	0.11	0.49	0.2	82.18	unclass. *Flavobacteriales*	unclass. *Flavobacteriales*	*Flavobacteriales*	*Flavobacteria*	*Bacteroidetes*	*Bacteria*
Otu0264	0.2	0.2	0.11	0.67	0.2	82.37	*Balneola*	*Chitinophagaceae*	*Sphingobacteriales*	*Sphingobacteria*	*Bacteroidetes*	*Bacteria*
Otu0339	0.28	0	0.11	0.49	0.18	82.55	unclass. *Proteobacteria*	unclass. *Proteobacteria*	unclass. *Proteobacteria*	unclass. *Proteobacteria*	*Proteobacteria*	*Bacteria*
Otu0016	0.28	0	0.1	0.49	0.17	82.73	unclass. *Gammaproteobacteria*	unclass. *Gammaproteobacteria*	unclass. *Gammaproteobacteria*	*Gammaproteobacteria*	*Proteobacteria*	*Bacteria*
Otu0064	0	0.28	0.1	0.49	0.17	82.9	unclass. *Flavobacteriaceae*	*Flavobacteriaceae*	*Flavobacteriales*	*Flavobacteria*	*Bacteroidetes*	*Bacteria*
Otu0306	0	0.28	0.1	0.49	0.17	83.07	unclass. *Bacteria*	unclass. *Bacteria*	unclass. *Bacteria*	unclass. *Bacteria*	unclass. *Bacteria*	*Bacteria*
Otu0410	0	0.28	0.1	0.49	0.17	83.24	*Lewinella*	*Saprospiraceae*	*Sphingobacteriales*	*Sphingobacteria*	*Bacteroidetes*	*Bacteria*
Otu0417	0	0.28	0.1	0.49	0.17	83.42	*Maribacter*	*Flavobacteriaceae*	*Flavobacteriales*	*Flavobacteria*	*Bacteroidetes*	*Bacteria*
Otu0168	0	0.28	0.09	0.49	0.16	83.58	unclass. *Flavobacteriaceae*	*Flavobacteriaceae*	*Flavobacteriales*	*Flavobacteria*	*Bacteroidetes*	*Bacteria*
Otu0224	0	0.28	0.09	0.49	0.16	83.74	unclass. *Flavobacteriaceae*	*Flavobacteriaceae*	*Flavobacteriales*	*Flavobacteria*	*Bacteroidetes*	*Bacteria*
Otu0154	0	0.2	0.08	0.49	0.14	83.88	unclass. *Gammaproteobacteria*	unclass. *Gammaproteobacteria*	unclass. *Gammaproteobacteria*	*Gammaproteobacteria*	*Proteobacteria*	*Bacteria*
Otu0188	0	0.2	0.08	0.49	0.14	84.03	*Ulvibacter*	*Flavobacteriaceae*	*Flavobacteriales*	*Flavobacteria*	*Bacteroidetes*	*Bacteria*
Otu0247	0	0.2	0.08	0.49	0.14	84.17	unclass. *Flavobacteriaceae*	*Flavobacteriaceae*	*Flavobacteriales*	*Flavobacteria*	*Bacteroidetes*	*Bacteria*
Otu0250	0	0.2	0.08	0.49	0.14	84.32	unclass. *Bacteria*	unclass. *Bacteria*	unclass. *Bacteria*	unclass. *Bacteria*	unclass. *Bacteria*	*Bacteria*
Otu0269	0	0.2	0.08	0.49	0.14	84.46	unclass. *Gammaproteobacteria*	unclass. *Gammaproteobacteria*	unclass. *Gammaproteobacteria*	*Gammaproteobacteria*	*Proteobacteria*	*Bacteria*
Otu0301	0	0.2	0.08	0.49	0.14	84.6	*Paracoccus*	*Rhodobacteraceae*	*Rhodobacterales*	*Alphaproteobacteria*	*Proteobacteria*	*Bacteria*
Otu0305	0	0.2	0.08	0.49	0.14	84.75	unclass. *Bacteria*	unclass. *Bacteria*	unclass. *Bacteria*	unclass. *Bacteria*	unclass. *Bacteria*	*Bacteria*
Otu0371	0	0.2	0.08	0.49	0.14	84.89	*Crocinitomix*	*Cryomorphaceae*	*Flavobacteriales*	*Flavobacteria*	*Bacteroidetes*	*Bacteria*
Otu0372	0	0.2	0.08	0.49	0.14	85.04	unclass. *Flavobacteriaceae*	*Flavobacteriaceae*	*Flavobacteriales*	*Flavobacteria*	*Bacteroidetes*	*Bacteria*
Otu0374	0	0.2	0.08	0.49	0.14	85.18	unclass. *Flavobacteriaceae*	*Flavobacteriaceae*	*Flavobacteriales*	*Flavobacteria*	*Bacteroidetes*	*Bacteria*
Otu0185	0	0.2	0.08	0.49	0.13	85.31	unclass. *Bacteria*	unclass. *Bacteria*	unclass. *Bacteria*	unclass. *Bacteria*	unclass. *Bacteria*	*Bacteria*
Otu0225	0	0.2	0.08	0.49	0.13	85.44	unclass. *Bacteria*	unclass. *Bacteria*	unclass. *Bacteria*	unclass. *Bacteria*	unclass. *Bacteria*	*Bacteria*
Otu0238	0	0.2	0.08	0.49	0.13	85.58	*Haliea*	*Alteromonadaceae*	*Alteromonadales*	*Gammaproteobacteria*	*Proteobacteria*	*Bacteria*
Otu0288	0	0.2	0.08	0.49	0.13	85.71	*Ulvibacter*	*Flavobacteriaceae*	*Flavobacteriales*	*Flavobacteria*	*Bacteroidetes*	*Bacteria*

OTU	Av.A_i (*pH in situ*)	Av.A_i (pH 7.67)	Av.δ_i	Av.δ_i/SD	Av.δ_i %	∑Av.δ_i %	Genus	Family	Order	Class	Phylum	Domain
Otu0328	0	0.2	0.08	0.49	0.13	85.84	unclass. *Proteobacteria*	unclass. *Proteobacteria*	unclass. *Proteobacteria*	unclass. *Proteobacteria*	*Proteobacteria*	*Bacteria*
Otu0343	0	0.2	0.08	0.49	0.13	85.97	unclass. *Proteobacteria*	unclass. *Proteobacteria*	unclass. *Proteobacteria*	unclass. *Proteobacteria*	*Proteobacteria*	*Bacteria*
Otu0384	0	0.2	0.08	0.49	0.13	86.1	unclass. *Bacteroidetes*	unclass. *Bacteroidetes*	unclass. *Bacteroidetes*	unclass. *Bacteroidetes*	*Bacteroidetes*	*Bacteria*
Otu0423	0	0.2	0.08	0.49	0.13	86.23	unclass. *Flavobacteriaceae*	*Flavobacteriaceae*	*Flavobacteriales*	*Flavobacteria*	*Bacteroidetes*	*Bacteria*
Otu0424	0	0.2	0.08	0.49	0.13	86.36	unclass. *Flavobacteriales*	unclass. *Flavobacteriales*	*Flavobacteriales*	*Flavobacteria*	*Bacteroidetes*	*Bacteria*
Otu0425	0	0.2	0.08	0.49	0.13	86.49	*Colwellia*	*Colwelliaceae*	*Alteromonadales*	*Gammaproteobacteria*	*Proteobacteria*	*Bacteria*
Otu0426	0	0.2	0.08	0.49	0.13	86.63	unclass. *Gammaproteobacteria*	unclass. *Gammaproteobacteria*	unclass. *Gammaproteobacteria*	*Gammaproteobacteria*	*Proteobacteria*	*Bacteria*
Otu0429	0	0.2	0.08	0.49	0.13	86.76	unclass. *Flavobacteriales*	unclass. *Flavobacteriales*	*Flavobacteriales*	*Flavobacteria*	*Bacteroidetes*	*Bacteria*
Otu0432	0	0.2	0.08	0.49	0.13	86.89	unclass. *Gammaproteobacteria*	unclass. *Gammaproteobacteria*	unclass. *Gammaproteobacteria*	*Gammaproteobacteria*	*Proteobacteria*	*Bacteria*
Otu0433	0	0.2	0.08	0.49	0.13	87.02	*Photobacterium*	*Vibrionaceae*	*Vibrionales*	*Gammaproteobacteria*	*Proteobacteria*	*Bacteria*
Otu0028	0.2	0	0.07	0.49	0.13	87.15	unclass. *Gammaproteobacteria*	unclass. *Gammaproteobacteria*	unclass. *Gammaproteobacteria*	*Gammaproteobacteria*	*Proteobacteria*	*Bacteria*
Otu0327	0.2	0	0.07	0.49	0.13	87.28	unclass. *Bacteria*	unclass. *Bacteria*	unclass. *Bacteria*	unclass. *Bacteria*	unclass. *Bacteria*	*Bacteria*
Otu0333	0.2	0	0.07	0.49	0.13	87.41	unclass. *Gammaproteobacteria*	unclass. *Gammaproteobacteria*	unclass. *Gammaproteobacteria*	*Gammaproteobacteria*	*Proteobacteria*	*Bacteria*
Otu0340	0.2	0	0.07	0.49	0.13	87.54	unclass. *Bacteria*	unclass. *Bacteria*	unclass. *Bacteria*	unclass. *Bacteria*	unclass. *Bacteria*	*Bacteria*
Otu0344	0.2	0	0.07	0.49	0.13	87.67	unclass. *Flavobacteriaceae*	*Flavobacteriaceae*	*Flavobacteriales*	*Flavobacteria*	*Bacteroidetes*	*Bacteria*
Otu0076	0.2	0	0.07	0.49	0.13	87.8	unclass. *Flammeovirgaceae*	*Flammeovirgaceae*	*Sphingobacteriales*	*Sphingobacteria*	*Bacteroidetes*	*Bacteria*
Otu0183	0.2	0	0.07	0.49	0.13	87.93	unclass. *Bacteroidetes*	unclass. *Bacteroidetes*	unclass. *Bacteroidetes*	unclass. *Bacteroidetes*	*Bacteroidetes*	*Bacteria*
Otu0255	0.2	0	0.07	0.49	0.13	88.06	*Sphingopyxis*	*Sphingomonadaceae*	*Sphingomonadales*	*Alphaproteobacteria*	*Proteobacteria*	*Bacteria*
Otu0258	0.2	0	0.07	0.49	0.13	88.18	*Haliea*	*Alteromonadaceae*	*Alteromonadales*	*Gammaproteobacteria*	*Proteobacteria*	*Bacteria*
Otu0152	0.2	0	0.07	0.49	0.13	88.31	unclass. *Gammaproteobacteria*	unclass. *Gammaproteobacteria*	unclass. *Gammaproteobacteria*	*Gammaproteobacteria*	*Proteobacteria*	*Bacteria*
Otu0153	0.2	0	0.07	0.49	0.13	88.44	unclass. *Gammaproteobacteria*	unclass. *Gammaproteobacteria*	unclass. *Gammaproteobacteria*	*Gammaproteobacteria*	*Proteobacteria*	*Bacteria*
Otu0156	0.2	0	0.07	0.49	0.13	88.57	unclass. *Gammaproteobacteria*	unclass. *Gammaproteobacteria*	unclass. *Gammaproteobacteria*	*Gammaproteobacteria*	*Proteobacteria*	*Bacteria*
Otu0176	0.2	0	0.07	0.49	0.13	88.7	*Colwellia*	*Colwelliaceae*	*Alteromonadales*	*Gammaproteobacteria*	*Proteobacteria*	*Bacteria*
Otu0179	0.2	0	0.07	0.49	0.13	88.83	unclass. *Sphingobacteriales*	unclass. *Sphingobacteriales*	*Sphingobacteriales*	*Sphingobacteria*	*Bacteroidetes*	*Bacteria*
Otu0181	0.2	0	0.07	0.49	0.13	88.96	unclass. *Domain*	unclass. *Domain*	unclass. *Domain*	unclass. *Domain*	unclass. *Domain*	unclass. *Domain*
Otu0197	0.2	0	0.07	0.49	0.13	89.09	unclass. *Bacteria*	unclass. *Bacteria*	unclass. *Bacteria*	unclass. *Bacteria*	unclass. *Bacteria*	*Bacteria*
Otu0208	0.2	0	0.07	0.49	0.13	89.22	unclass. *Saprospiraceae*	*Saprospiraceae*	*Sphingobacteriales*	*Sphingobacteria*	*Bacteroidetes*	*Bacteria*

OTU	Av.A_i (*pH in situ*)	Av.A_i (pH 7.67)	Av.δ_i	Av.δ_i/SD	Av.δ_i %	∑Av.δ_i %	Genus	Family	Order	Class	Phylum	Domain
Otu0226	0.2	0	0.07	0.49	0.13	89.35	unclass. *Bacteroidetes*	unclass. *Bacteroidetes*	unclass. *Bacteroidetes*	unclass. *Bacteroidetes*	*Bacteroidetes*	*Bacteria*
Otu0031	0.2	0	0.07	0.49	0.13	89.47	unclass. *Gammaproteobacteria*	unclass. *Gammaproteobacteria*	unclass. *Gammaproteobacteria*	*Gammaproteobacteria*	*Proteobacteria*	*Bacteria*
Otu0277	0.2	0	0.07	0.49	0.13	89.6	unclass. *Flavobacteriaceae*	*Flavobacteriaceae*	*Flavobacteriales*	*Flavobacteria*	*Bacteroidetes*	*Bacteria*
Otu0287	0.2	0	0.07	0.49	0.13	89.73	*Haliscomenobacter*	*Saprospiraceae*	*Sphingobacteriales*	*Sphingobacteria*	*Bacteroidetes*	*Bacteria*
Otu0296	0.2	0	0.07	0.49	0.13	89.85	*Polaribacter*	*Flavobacteriaceae*	*Flavobacteriales*	*Flavobacteria*	*Bacteroidetes*	*Bacteria*
Otu0309	0.2	0	0.07	0.49	0.13	89.98	unclass. *Bacteria*	unclass. *Bacteria*	unclass. *Bacteria*	unclass. *Bacteria*	unclass. *Bacteria*	*Bacteria*
Otu0311	0.2	0	0.07	0.49	0.13	90.11	unclass. *Gammaproteobacteria*	unclass. *Gammaproteobacteria*	unclass. *Gammaproteobacteria*	*Gammaproteobacteria*	*Proteobacteria*	*Bacteria*
Spring 'serial dilution' (average dissimilarity: 52.1%)												
Otu0035	9.32	2.06	6.2	1.2	11.89	11.89	*Colwellia*	*Colwelliaceae*	*Alteromonadales*	*Gammaproteobacteria*	*Proteobacteria*	*Bacteria*
Otu0027	11.23	18.57	5.74	1.25	11.02	22.9	*Pseudoalteromonas*	*Pseudoalteromonadaceae*	*Alteromonadales*	*Gammaproteobacteria*	*Proteobacteria*	*Bacteria*
Otu0106	7.05	4.44	2.99	2.06	5.73	28.64	*Marinomonas*	*Oceanospirillaceae*	*Oceanospirillales*	*Gammaproteobacteria*	*Proteobacteria*	*Bacteria*
Otu0039	0.68	4.41	2.86	1.78	5.49	34.13	*Pseudoalteromonas*	*Pseudoalteromonadaceae*	*Alteromonadales*	*Gammaproteobacteria*	*Proteobacteria*	*Bacteria*
Otu0109	1.72	3.81	2.78	2.99	5.34	39.46	*Alteromonas*	*Alteromonadaceae*	*Alteromonadales*	*Gammaproteobacteria*	*Proteobacteria*	*Bacteria*
Otu0010	3.24	0.35	2.22	0.58	4.26	43.72	unclass. *Flavobacteriaceae*	*Flavobacteriaceae*	*Flavobacteriales*	*Flavobacteria*	*Bacteroidetes*	*Bacteria*
Otu0036	3.68	1.53	1.87	1.85	3.58	47.31	*Glaciecola*	*Alteromonadaceae*	*Alteromonadales*	*Gammaproteobacteria*	*Proteobacteria*	*Bacteria*
Otu0115	1.02	2.07	1.71	4.68	3.28	50.59	*Glaciecola*	*Alteromonadaceae*	*Alteromonadales*	*Gammaproteobacteria*	*Proteobacteria*	*Bacteria*
Otu0107	4.07	2.41	1.69	1.36	3.24	53.83	unclass. *Rhodobacteraceae*	*Rhodobacteraceae*	*Rhodobacterales*	*Alphaproteobacteria*	*Proteobacteria*	*Bacteria*
Otu0113	1.95	2.22	1.51	2.6	2.89	56.73	*Colwellia*	*Colwelliaceae*	*Alteromonadales*	*Gammaproteobacteria*	*Proteobacteria*	*Bacteria*
Otu0114	0.75	2.57	1.48	1.61	2.84	59.56	*Shewanella*	*Shewanellaceae*	*Alteromonadales*	*Gammaproteobacteria*	*Proteobacteria*	*Bacteria*
Otu0117	0	1.87	1.44	2.37	2.76	62.33	*Arcobacter*	*Campylobacteraceae*	*Campylobacterales*	*Epsilonproteobacteria*	*Proteobacteria*	*Bacteria*
Otu0110	0.96	1.25	1.25	0.99	2.4	64.73	unclass. *Rhodobacteraceae*	*Rhodobacteraceae*	*Rhodobacterales*	*Alphaproteobacteria*	*Proteobacteria*	*Bacteria*
Otu0111	2.28	3	1.25	1.61	2.4	67.13	*Marinomonas*	*Oceanospirillaceae*	*Oceanospirillales*	*Gammaproteobacteria*	*Proteobacteria*	*Bacteria*
Otu0108	1.4	1.69	1.09	1.25	2.1	69.23	unclass. *Colwelliaceae*	*Colwelliaceae*	*Alteromonadales*	*Gammaproteobacteria*	*Proteobacteria*	*Bacteria*
Otu0059	1.29	2.27	1.08	1.45	2.07	71.3	*Flavobacterium*	*Flavobacteriaceae*	*Flavobacteriales*	*Flavobacteria*	*Bacteroidetes*	*Bacteria*
Otu0041	0.89	1.5	0.98	1.28	1.89	73.19	*Sulfitobacter*	*Rhodobacteraceae*	*Rhodobacterales*	*Alphaproteobacteria*	*Proteobacteria*	*Bacteria*
Otu0116	0	1.27	0.97	1.48	1.87	75.06	*Flavobacterium*	*Flavobacteriaceae*	*Flavobacteriales*	*Flavobacteria*	*Bacteroidetes*	*Bacteria*
Otu0123	1.12	1.22	0.88	1.29	1.69	76.75	unclass. *Gammaproteobacteria*	unclass. *Gammaproteobacteria*	unclass. *Gammaproteobacteria*	*Gammaproteobacteria*	*Proteobacteria*	*Bacteria*

OTU	Av.A_i (*pH in situ*)	Av.A_i (pH 7.67)	Av.δ_i	Av.δ_i/SD	Av.δ_i %	∑Av.δ_i %	Genus	Family	Order	Class	Phylum	Domain
Otu0055	0.91	0.61	0.83	1.3	1.58	78.33	*Colwellia*	*Colwelliaceae*	*Alteromonadales*	*Gammaproteobacteria*	*Proteobacteria*	*Bacteria*
Otu0112	0.48	1.26	0.82	1.17	1.58	79.91	*Polaribacter*	*Flavobacteriaceae*	*Flavobacteriales*	*Flavobacteria*	*Bacteroidetes*	*Bacteria*
Otu0132	1.08	0.85	0.7	1.51	1.34	81.25	unclass. *Alteromonadales*	unclass. *Alteromonadales*	*Alteromonadales*	*Gammaproteobacteria*	*Proteobacteria*	*Bacteria*
Otu0120	0.48	1.29	0.67	1.53	1.28	82.53	*Pseudoalteromonas*	*Pseudoalteromonadaceae*	*Alteromonadales*	*Gammaproteobacteria*	*Proteobacteria*	*Bacteria*
Otu0133	0	0.68	0.51	0.9	0.98	83.51	*Colwellia*	*Colwelliaceae*	*Alteromonadales*	*Gammaproteobacteria*	*Proteobacteria*	*Bacteria*
Otu0140	0.4	0.43	0.49	0.95	0.93	84.44	*Oleispira*	*Oceanospirillaceae*	*Oceanospirillales*	*Gammaproteobacteria*	*Proteobacteria*	*Bacteria*
Otu0124	0.4	0.43	0.48	0.73	0.93	85.37	*Loktanella*	*Rhodobacteraceae*	*Rhodobacterales*	*Alphaproteobacteria*	*Proteobacteria*	*Bacteria*
Otu0437	0	0.6	0.47	0.93	0.9	86.27	*Flavobacterium*	*Flavobacteriaceae*	*Flavobacteriales*	*Flavobacteria*	*Bacteroidetes*	*Bacteria*
Otu0572	0.2	0.43	0.42	0.72	0.8	87.07	*Croceibacter*	*Flavobacteriaceae*	*Flavobacteriales*	*Flavobacteria*	*Bacteroidetes*	*Bacteria*
Otu0176	0.48	0	0.39	0.77	0.75	87.81	*Colwellia*	*Colwelliaceae*	*Alteromonadales*	*Gammaproteobacteria*	*Proteobacteria*	*Bacteria*
Otu0438	0	0.5	0.38	0.97	0.73	88.55	*Tenacibaculum*	*Flavobacteriaceae*	*Flavobacteriales*	*Flavobacteria*	*Bacteroidetes*	*Bacteria*
Otu0118	0.4	0.25	0.35	0.88	0.67	89.22	*Colwellia*	*Colwelliaceae*	*Alteromonadales*	*Gammaproteobacteria*	*Proteobacteria*	*Bacteria*
Otu0320	0.2	0.35	0.35	0.73	0.67	89.89	*Pseudoalteromonas*	*Pseudoalteromonadaceae*	*Alteromonadales*	*Gammaproteobacteria*	*Proteobacteria*	*Bacteria*
Otu0119	0	0.35	0.28	0.56	0.53	90.42	*Colwellia*	*Colwelliaceae*	*Alteromonadales*	*Gammaproteobacteria*	*Proteobacteria*	*Bacteria*
Summer 'no dilution' (average dissimilarity: 53.4%)												
Otu0691	5.44	2.99	0.83	1.56	1.55	1.55	*Planctomyces*	*Planctomycetaceae*	*Planctomycetales*	*Planctomycetacia*	*Planctomycetes*	*Bacteria*
Otu0469	1.52	3.64	0.78	1.71	1.45	3.01	unclass. *Bacteria*	unclass. *Bacteria*	unclass. *Bacteria*	unclass. *Bacteria*	unclass. *Bacteria*	*Bacteria*
Otu0779	0.95	2.48	0.73	1.35	1.37	4.38	unclass. *Ectothiorhodospiraceae*	*Ectothiorhodospiraceae*	*Chromatiales*	*Gammaproteobacteria*	*Proteobacteria*	*Bacteria*
Otu0001	4.58	3.07	0.61	1.37	1.14	5.52	unclass. *Flavobacteriaceae*	*Flavobacteriaceae*	*Flavobacteriales*	*Flavobacteria*	*Bacteroidetes*	*Bacteria*
Otu0010	0.88	2.58	0.59	1.55	1.1	6.62	unclass. *Flavobacteriaceae*	*Flavobacteriaceae*	*Flavobacteriales*	*Flavobacteria*	*Bacteroidetes*	*Bacteria*
Otu0753	0.95	1.22	0.53	1.03	0.99	7.61	unclass. *Flavobacteriales*	unclass. *Flavobacteriales*	*Flavobacteriales*	*Flavobacteria*	*Bacteroidetes*	*Bacteria*
Otu0450	1.66	1.2	0.52	1.28	0.97	8.58	*Sphingobium*	*Sphingomonadaceae*	*Sphingomonadales*	*Alphaproteobacteria*	*Proteobacteria*	*Bacteria*
Otu0693	3.22	2.46	0.5	1.32	0.93	9.51	*Planctomyces*	*Planctomycetaceae*	*Planctomycetales*	*Planctomycetacia*	*Planctomycetes*	*Bacteria*
Otu0005	15.27	15.85	0.5	1.45	0.93	10.44	*Pelagibacter*	SAR11-clade	*Rickettsiales*	*Alphaproteobacteria*	*Proteobacteria*	*Bacteria*
Otu0002	0.4	1.81	0.48	1.62	0.91	11.35	unclass. *Flavobacteriaceae*	*Flavobacteriaceae*	*Flavobacteriales*	*Flavobacteria*	*Bacteroidetes*	*Bacteria*
Otu0203	3.14	3.03	0.48	1.38	0.89	12.24	*Pelagibacter*	SAR11-clade	*Rickettsiales*	*Alphaproteobacteria*	*Proteobacteria*	*Bacteria*
Otu0084	0.93	2.23	0.46	1.57	0.86	13.1	unclass. *Gammaproteobacteria*	unclass. *Gammaproteobacteria*	unclass. *Gammaproteobacteria*	*Gammaproteobacteria*	*Proteobacteria*	*Bacteria*

OTU	Av.A_i (*pH in situ*)	Av.A_i (pH 7.67)	Av.δ_i	Av.δ_i/SD	Av.δ_i %	∑Av.δ_i %	Genus	Family	Order	Class	Phylum	Domain
Otu0352	0.4	1.47	0.42	1.72	0.79	13.89	unclass. *Flavobacteriaceae*	*Flavobacteriaceae*	*Flavobacteriales*	*Flavobacteria*	*Bacteroidetes*	*Bacteria*
Otu0512	1.58	0.89	0.42	1.37	0.79	14.68	*Rhodopirellula*	*Planctomycetaceae*	*Planctomycetales*	*Planctomycetacia*	*Planctomycetes*	*Bacteria*
Otu0695	1.12	1.38	0.4	1.4	0.75	15.42	unclass. *Proteobacteria*	unclass. *Proteobacteria*	unclass. *Proteobacteria*	unclass. *Proteobacteria*	*Proteobacteria*	*Bacteria*
Otu0277	0.4	1.56	0.4	1.73	0.75	16.17	unclass. *Flavobacteriaceae*	*Flavobacteriaceae*	*Flavobacteriales*	*Flavobacteria*	*Bacteroidetes*	*Bacteria*
Otu0157	1.37	0.55	0.4	1.38	0.74	16.91	unclass. *Gammaproteobacteria*	unclass. *Gammaproteobacteria*	unclass. *Gammaproteobacteria*	*Gammaproteobacteria*	*Proteobacteria*	*Bacteria*
Otu0705	1.19	0.28	0.39	1.24	0.73	17.64	*Planctomyces*	*Planctomycetaceae*	*Planctomycetales*	*Planctomycetacia*	*Planctomycetes*	*Bacteria*
Otu0007	2.06	1.13	0.39	1.23	0.72	18.36	unclass. *Flavobacteriales*	unclass. *Flavobacteriales*	*Flavobacteriales*	*Flavobacteria*	*Bacteroidetes*	*Bacteria*
Otu0435	1.12	0	0.38	0.61	0.71	19.07	unclass. *Flavobacteriales*	unclass. *Flavobacteriales*	*Flavobacteriales*	*Flavobacteria*	*Bacteroidetes*	*Bacteria*
Otu0004	0	1.09	0.37	1.61	0.7	19.77	unclass. *Flavobacteriales*	unclass. *Flavobacteriales*	*Flavobacteriales*	*Flavobacteria*	*Bacteroidetes*	*Bacteria*
Otu0463	1.43	1.37	0.37	1.83	0.69	20.47	unclass. *Flavobacteriaceae*	*Flavobacteriaceae*	*Flavobacteriales*	*Flavobacteria*	*Bacteroidetes*	*Bacteria*
Otu0611	0.72	0.57	0.37	0.81	0.69	21.15	unclass. *Bacteroidetes*	unclass. *Bacteroidetes*	unclass. *Bacteroidetes*	unclass. *Bacteroidetes*	*Bacteroidetes*	*Bacteria*
Otu0060	0.35	1.08	0.36	1.45	0.67	21.83	unclass. *Actinomycetales*	unclass. *Actinomycetales*	*Actinomycetales*	*Actinobacteria*	*Actinobacteria*	*Bacteria*
Otu0692	2.21	2.58	0.36	1.49	0.67	22.5	unclass. *Bacteria*	unclass. *Bacteria*	unclass. *Bacteria*	unclass. *Bacteria*	unclass. *Bacteria*	*Bacteria*
Otu0905	0	1.06	0.35	0.49	0.66	23.16	unclass. *Gammaproteobacteria*	unclass. *Gammaproteobacteria*	unclass. *Gammaproteobacteria*	*Gammaproteobacteria*	*Proteobacteria*	*Bacteria*
Otu0289	1.08	0.48	0.35	1.14	0.66	23.82	unclass. *Bacteroidetes*	unclass. *Bacteroidetes*	unclass. *Bacteroidetes*	unclass. *Bacteroidetes*	*Bacteroidetes*	*Bacteria*
Otu0068	1.51	0.89	0.34	1.73	0.64	24.47	*Pelagibacter*	SAR11-clade	*Rickettsiales*	*Alphaproteobacteria*	*Proteobacteria*	*Bacteria*
Otu0480	0.2	0.98	0.33	1.24	0.62	25.09	*Ilumatobacter*	*Acidimicrobidae_incertae_sedis*	*Actinobacteria_order_incertae_ sedis*	*Actinobacteria*	*Actinobacteria*	*Bacteria*
Otu0003	3.21	2.58	0.33	1.38	0.62	25.71	unclass. *Rhodobacteraceae*	*Rhodobacteraceae*	*Rhodobacterales*	*Alphaproteobacteria*	*Proteobacteria*	*Bacteria*
Otu0048	0.63	0.8	0.32	1.06	0.6	26.31	unclass. *Flavobacteriaceae*	*Flavobacteriaceae*	*Flavobacteriales*	*Flavobacteria*	*Bacteroidetes*	*Bacteria*
Otu0461	1.89	1.45	0.32	0.99	0.59	26.9	unclass. *Microbacteriaceae*	*Microbacteriaceae*	*Actinomycetales*	*Actinobacteria*	*Actinobacteria*	*Bacteria*
Otu0696	0.68	0.77	0.32	1.16	0.59	27.5	unclass. *Flavobacteriaceae*	*Flavobacteriaceae*	*Flavobacteriales*	*Flavobacteria*	*Bacteroidetes*	*Bacteria*
Otu0261	0.77	0.99	0.31	1.3	0.59	28.08	*Winogradskyella*	*Flavobacteriaceae*	*Flavobacteriales*	*Flavobacteria*	*Bacteroidetes*	*Bacteria*
Otu0053	1.29	1.25	0.31	1.43	0.58	28.66	unclass. *Rhodobacteraceae*	*Rhodobacteraceae*	*Rhodobacterales*	*Alphaproteobacteria*	*Proteobacteria*	*Bacteria*
Otu0495	1.53	1.11	0.31	1.24	0.58	29.24	unclass. *Flavobacteriales*	unclass. *Flavobacteriales*	*Flavobacteriales*	*Flavobacteria*	*Bacteroidetes*	*Bacteria*
Otu0710	0.48	1.31	0.31	1.54	0.57	29.82	unclass. *Gammaproteobacteria*	unclass. *Gammaproteobacteria*	unclass. *Gammaproteobacteria*	*Gammaproteobacteria*	*Proteobacteria*	*Bacteria*
Otu0519	1.72	1.95	0.3	1.22	0.56	30.38	unclass. *Rhodospirillaceae*	*Rhodospirillaceae*	*Rhodospirillales*	*Alphaproteobacteria*	*Proteobacteria*	*Bacteria*
Otu0186	0.97	0.6	0.29	1.56	0.55	30.93	unclass. *Planctomycetaceae*	*Planctomycetaceae*	*Planctomycetales*	*Planctomycetacia*	*Planctomycetes*	*Bacteria*

OTU	Av.A_i (*pH in situ*)	Av.A_i (pH 7.67)	Av.δ_i	Av.δ_i/SD	Av.δ_i %	∑Av.δ_i %	Genus	Family	Order	Class	Phylum	Domain
Otu0701	0.88	0.2	0.29	1.09	0.55	31.48	unclass. *Bacteria*	unclass. *Bacteria*	unclass. *Bacteria*	unclass. *Bacteria*	unclass. *Bacteria*	*Bacteria*
Otu0468	0.57	0.88	0.29	1.12	0.55	32.03	unclass. *Flavobacteriaceae*	*Flavobacteriaceae*	*Flavobacteriales*	*Flavobacteria*	*Bacteroidetes*	*Bacteria*
Otu0490	0.4	0.91	0.29	1.29	0.54	32.57	unclass. *Burkholderiales*	unclass. *Burkholderiales*	*Burkholderiales*	*Betaproteobacteria*	*Proteobacteria*	*Bacteria*
Otu0697	0.6	0.69	0.28	1.47	0.53	33.1	unclass. *Flavobacteriaceae*	*Flavobacteriaceae*	*Flavobacteriales*	*Flavobacteria*	*Bacteroidetes*	*Bacteria*
Otu0052	2.91	2.27	0.27	1.17	0.51	33.6	unclass. *Betaproteobacteria*	unclass. *Betaproteobacteria*	unclass. *Betaproteobacteria*	*Betaproteobacteria*	*Proteobacteria*	*Bacteria*
Otu0031	0.35	0.75	0.27	1.15	0.51	34.11	unclass. *Gammaproteobacteria*	unclass. *Gammaproteobacteria*	unclass. *Gammaproteobacteria*	*Gammaproteobacteria*	*Proteobacteria*	*Bacteria*
Otu0708	0	0.8	0.27	1.94	0.51	34.62	unclass. *Proteobacteria*	unclass. *Proteobacteria*	unclass. *Proteobacteria*	unclass. *Proteobacteria*	*Proteobacteria*	*Bacteria*
Otu0245	0.97	0.98	0.27	1.35	0.51	35.13	unclass. *Flavobacteriales*	unclass. *Flavobacteriales*	*Flavobacteriales*	*Flavobacteria*	*Bacteroidetes*	*Bacteria*
Otu0947	0	0.72	0.27	0.49	0.5	35.63	unclass. *Bacteroidetes*	unclass. *Bacteroidetes*	unclass. *Bacteroidetes*	unclass. *Bacteroidetes*	*Bacteroidetes*	*Bacteria*
Otu0759	0.28	0.77	0.25	1.11	0.48	36.1	unclass. *Verrucomicrobiaceae*	*Verrucomicrobiaceae*	*Verrucomicrobiales*	*Verrucomicrobiae*	*Verrucomicrobia*	*Bacteria*
Otu0703	1.03	0.75	0.25	1.2	0.47	36.57	unclass. *Gammaproteobacteria*	unclass. *Gammaproteobacteria*	unclass. *Gammaproteobacteria*	*Gammaproteobacteria*	*Proteobacteria*	*Bacteria*
Otu0046	1.29	1.44	0.25	0.96	0.47	37.05	unclass. *Flavobacteriaceae*	*Flavobacteriaceae*	*Flavobacteriales*	*Flavobacteria*	*Bacteroidetes*	*Bacteria*
Otu0142	0.68	0.83	0.25	1.23	0.47	37.51	unclass. *Gammaproteobacteria*	unclass. *Gammaproteobacteria*	unclass. *Gammaproteobacteria*	*Gammaproteobacteria*	*Proteobacteria*	*Bacteria*
Otu0510	0.2	0.77	0.25	1.15	0.47	37.98	unclass. *Gammaproteobacteria*	unclass. *Gammaproteobacteria*	unclass. *Gammaproteobacteria*	*Gammaproteobacteria*	*Proteobacteria*	*Bacteria*
Otu0019	1.33	0.6	0.25	0.86	0.47	38.45	unclass. *Gammaproteobacteria*	unclass. *Gammaproteobacteria*	unclass. *Gammaproteobacteria*	*Gammaproteobacteria*	*Proteobacteria*	*Bacteria*
Otu0502	1.03	1.13	0.25	1.19	0.46	38.91	unclass. *Gammaproteobacteria*	unclass. *Gammaproteobacteria*	unclass. *Gammaproteobacteria*	*Gammaproteobacteria*	*Proteobacteria*	*Bacteria*
Otu0061	0.2	0.65	0.25	0.8	0.46	39.37	unclass. *Actinomycetales*	unclass. *Actinomycetales*	*Actinomycetales*	*Actinobacteria*	*Actinobacteria*	*Bacteria*
Otu0737	0.63	0.48	0.24	1.03	0.46	39.83	unclass. *Gammaproteobacteria*	unclass. *Gammaproteobacteria*	unclass. *Gammaproteobacteria*	*Gammaproteobacteria*	*Proteobacteria*	*Bacteria*
Otu0694	0.75	0.2	0.24	1.07	0.46	40.29	unclass. *Alphaproteobacteria*	unclass. *Alphaproteobacteria*	unclass. *Alphaproteobacteria*	*Alphaproteobacteria*	*Proteobacteria*	*Bacteria*
Otu0752	0.2	0.8	0.24	1.42	0.45	40.74	unclass. *Gammaproteobacteria*	unclass. *Gammaproteobacteria*	unclass. *Gammaproteobacteria*	*Gammaproteobacteria*	*Proteobacteria*	*Bacteria*
Otu0768	0.6	0.35	0.24	1.28	0.45	41.19	unclass. *Flavobacteriales*	unclass. *Flavobacteriales*	*Flavobacteriales*	*Flavobacteria*	*Bacteroidetes*	*Bacteria*
Otu0408	0.75	0.2	0.24	1.11	0.45	41.64	unclass. *Flavobacteriaceae*	*Flavobacteriaceae*	*Flavobacteriales*	*Flavobacteria*	*Bacteroidetes*	*Bacteria*
Otu0532	0.77	0.4	0.24	1.19	0.44	42.08	unclass. *Rhodobacteraceae*	*Rhodobacteraceae*	*Rhodobacterales*	*Alphaproteobacteria*	*Proteobacteria*	*Bacteria*
Otu0503	1.15	1.41	0.24	1.24	0.44	42.52	unclass. *Bacteroidetes*	unclass. *Bacteroidetes*	unclass. *Bacteroidetes*	unclass. *Bacteroidetes*	*Bacteroidetes*	*Bacteria*
Otu0272	0.6	0.57	0.24	1.32	0.44	42.97	unclass. *Flavobacteriaceae*	*Flavobacteriaceae*	*Flavobacteriales*	*Flavobacteria*	*Bacteroidetes*	*Bacteria*
Otu0380	0.35	0.57	0.23	0.91	0.44	43.4	unclass. *Proteobacteria*	unclass. *Proteobacteria*	unclass. *Proteobacteria*	unclass. *Proteobacteria*	*Proteobacteria*	*Bacteria*
Otu0475	0	0.68	0.23	1.18	0.43	43.83	unclass. *Microbacteriaceae*	*Microbacteriaceae*	*Actinomycetales*	*Actinobacteria*	*Actinobacteria*	*Bacteria*

OTU	Av.A_i (*pH in situ*)	Av.A_i (pH 7.67)	Av.δ_i	Av.δ_i/SD	Av.δ_i %	∑Av.δ_i %	Genus	Family	Order	Class	Phylum	Domain
Otu0782	0.68	0.77	0.23	1.14	0.43	44.26	unclass. *Gammaproteobacteria*	unclass. *Gammaproteobacteria*	unclass. *Gammaproteobacteria*	*Gammaproteobacteria*	*Proteobacteria*	*Bacteria*
Otu0714	0.68	0.48	0.23	1.13	0.43	44.69	unclass. *Alphaproteobacteria*	unclass. *Alphaproteobacteria*	unclass. *Alphaproteobacteria*	*Alphaproteobacteria*	*Proteobacteria*	*Bacteria*
Otu0041	0.75	0.4	0.23	1.1	0.42	45.11	*Sulfitobacter*	*Rhodobacteraceae*	*Rhodobacterales*	*Alphaproteobacteria*	*Proteobacteria*	*Bacteria*
Otu0020	0.63	0.2	0.23	0.92	0.42	45.54	unclass. *Flammeovirgaceae*	*Flammeovirgaceae*	*Sphingobacteriales*	*Sphingobacteria*	*Bacteroidetes*	*Bacteria*
Otu0473	1.63	1.46	0.23	1.09	0.42	45.96	unclass. *Alphaproteobacteria*	unclass. *Alphaproteobacteria*	unclass. *Alphaproteobacteria*	*Alphaproteobacteria*	*Proteobacteria*	*Bacteria*
Otu0221	0.48	0.57	0.22	0.99	0.42	46.38	unclass. *Flavobacteriaceae*	*Flavobacteriaceae*	*Flavobacteriales*	*Flavobacteria*	*Bacteroidetes*	*Bacteria*
Otu0732	0.48	0.35	0.22	0.89	0.41	46.79	unclass. *Proteobacteria*	unclass. *Proteobacteria*	unclass. *Proteobacteria*	unclass. *Proteobacteria*	*Proteobacteria*	*Bacteria*
Otu0368	0.6	0.28	0.22	1.25	0.41	47.2	unclass. *Saprospiraceae*	*Saprospiraceae*	*Sphingobacteriales*	*Sphingobacteria*	*Bacteroidetes*	*Bacteria*
Otu0781	0.63	0	0.22	0.49	0.41	47.61	unclass. *Thiotrichales*	unclass. *Thiotrichales*	*Thiotrichales*	*Gammaproteobacteria*	*Proteobacteria*	*Bacteria*
Otu0347	0	0.6	0.22	1.19	0.4	48.02	unclass. *Flavobacteriaceae*	*Flavobacteriaceae*	*Flavobacteriales*	*Flavobacteria*	*Bacteroidetes*	*Bacteria*
Otu0875	0	0.6	0.21	1.19	0.4	48.42	unclass. *Flavobacteriales*	unclass. *Flavobacteriales*	*Flavobacteriales*	*Flavobacteria*	*Bacteroidetes*	*Bacteria*
Otu0467	0.4	0.55	0.21	1	0.4	48.81	unclass. *Rhodospirillaceae*	*Rhodospirillaceae*	*Rhodospirillales*	*Alphaproteobacteria*	*Proteobacteria*	*Bacteria*
Otu0504	0.68	0.4	0.21	1.09	0.39	49.2	unclass. *Alphaproteobacteria*	unclass. *Alphaproteobacteria*	unclass. *Alphaproteobacteria*	*Alphaproteobacteria*	*Proteobacteria*	*Bacteria*
Otu0497	0.48	0.6	0.21	1.17	0.39	49.6	*Opitutus*	*Opitutaceae*	*Opitutales*	*Opitutae*	*Verrucomicrobia*	*Bacteria*
Otu0505	0.63	0	0.21	0.8	0.39	49.99	unclass. *Chitinophagaceae*	*Chitinophagaceae*	*Sphingobacteriales*	*Sphingobacteria*	*Bacteroidetes*	*Bacteria*
Otu0803	0.2	0.55	0.21	0.85	0.39	50.38	unclass. *Cryomorphaceae*	*Cryomorphaceae*	*Flavobacteriales*	*Flavobacteria*	*Bacteroidetes*	*Bacteria*
Otu0700	0.68	0.4	0.21	1.1	0.39	50.76	unclass. *Alphaproteobacteria*	unclass. *Alphaproteobacteria*	unclass. *Alphaproteobacteria*	*Alphaproteobacteria*	*Proteobacteria*	*Bacteria*
Otu0191	0.6	0	0.2	1.19	0.38	51.15	unclass. *Proteobacteria*	unclass. *Proteobacteria*	unclass. *Proteobacteria*	unclass. *Proteobacteria*	*Proteobacteria*	*Bacteria*
Otu0721	0.6	0	0.2	1.19	0.38	51.52	unclass. *Alphaproteobacteria*	unclass. *Alphaproteobacteria*	unclass. *Alphaproteobacteria*	*Alphaproteobacteria*	*Proteobacteria*	*Bacteria*
Otu0075	0.28	0.48	0.2	0.87	0.38	51.9	unclass. *Alphaproteobacteria*	unclass. *Alphaproteobacteria*	unclass. *Alphaproteobacteria*	*Alphaproteobacteria*	*Proteobacteria*	*Bacteria*
Otu0706	1.99	1.88	0.2	1.33	0.38	52.28	unclass. *Planctomycetaceae*	*Planctomycetaceae*	*Planctomycetales*	*Planctomycetacia*	*Planctomycetes*	*Bacteria*
Otu0521	1.08	0.77	0.2	1.24	0.37	52.65	unclass. *Rhodobacteraceae*	*Rhodobacteraceae*	*Rhodobacterales*	*Alphaproteobacteria*	*Proteobacteria*	*Bacteria*
Otu0754	0.48	0.28	0.2	0.88	0.37	53.02	unclass. *Flavobacteriales*	unclass. *Flavobacteriales*	*Flavobacteriales*	*Flavobacteria*	*Bacteroidetes*	*Bacteria*
Otu0833	0	0.6	0.2	1.2	0.37	53.39	*Alcanivorax*	*Alcanivoracaceae*	*Oceanospirillales*	*Gammaproteobacteria*	*Proteobacteria*	*Bacteria*
Otu0630	0.35	0.4	0.19	0.93	0.36	53.75	unclass. *Flavobacteriales*	unclass. *Flavobacteriales*	*Flavobacteriales*	*Flavobacteria*	*Bacteroidetes*	*Bacteria*
Otu0758	0.48	0.4	0.19	1.01	0.36	54.11	unclass. *Bacteria*	unclass. *Bacteria*	unclass. *Bacteria*	unclass. *Bacteria*	unclass. *Bacteria*	*Bacteria*
Otu0218	0.48	0.4	0.19	1.01	0.36	54.47	unclass. *Flavobacteriaceae*	*Flavobacteriaceae*	*Flavobacteriales*	*Flavobacteria*	*Bacteroidetes*	*Bacteria*

OTU	Av.A_i (*pH in situ*)	Av.A_i (pH 7.67)	Av.δ_i	Av.δ_i/SD	Av.δ_i %	∑Av.δ_i %	Genus	Family	Order	Class	Phylum	Domain
Otu0470	0.4	0.48	0.19	1.01	0.35	54.82	unclass. *Flavobacteriaceae*	*Flavobacteriaceae*	*Flavobacteriales*	*Flavobacteria*	*Bacteroidetes*	*Bacteria*
Otu0354	1.11	1.38	0.19	1.03	0.35	55.18	*Anderseniella*	*Rhodobiaceae*	*Rhizobiales*	*Alphaproteobacteria*	*Proteobacteria*	*Bacteria*
Otu0711	0	0.55	0.18	0.78	0.34	55.52	unclass. *Gammaproteobacteria*	unclass. *Gammaproteobacteria*	unclass. *Gammaproteobacteria*	*Gammaproteobacteria*	*Proteobacteria*	*Bacteria*
Otu0230	0.2	0.48	0.18	0.89	0.34	55.86	unclass. *Rhodobacteraceae*	*Rhodobacteraceae*	*Rhodobacterales*	*Alphaproteobacteria*	*Proteobacteria*	*Bacteria*
Otu0485	0.6	0.4	0.18	1.01	0.34	56.2	unclass. *Alphaproteobacteria*	unclass. *Alphaproteobacteria*	unclass. *Alphaproteobacteria*	*Alphaproteobacteria*	*Proteobacteria*	*Bacteria*
Otu0731	0.2	0.48	0.18	0.9	0.33	56.54	*Lentisphaera*	*Lentisphaeraceae*	*Lentisphaerales*	*Lentisphaeria*	*Lentisphaerae*	*Bacteria*
Otu0476	0.6	0.4	0.18	1.01	0.33	56.87	unclass. *Actinomycetales*	unclass. *Actinomycetales*	*Actinomycetales*	*Actinobacteria*	*Actinobacteria*	*Bacteria*
Otu0798	0.48	0.2	0.18	0.88	0.33	57.2	unclass. *Gammaproteobacteria*	unclass. *Gammaproteobacteria*	unclass. *Gammaproteobacteria*	*Gammaproteobacteria*	*Proteobacteria*	*Bacteria*
Otu0822	0.28	0.4	0.18	0.91	0.33	57.53	unclass. *Proteobacteria*	unclass. *Proteobacteria*	unclass. *Proteobacteria*	unclass. *Proteobacteria*	*Proteobacteria*	*Bacteria*
Otu0704	0.97	0.88	0.17	0.97	0.33	57.86	unclass. *Proteobacteria*	unclass. *Proteobacteria*	unclass. *Proteobacteria*	unclass. *Proteobacteria*	*Proteobacteria*	*Bacteria*
Otu0156	0.2	0.48	0.17	0.89	0.32	58.18	unclass. *Gammaproteobacteria*	unclass. *Gammaproteobacteria*	unclass. *Gammaproteobacteria*	*Gammaproteobacteria*	*Proteobacteria*	*Bacteria*
Otu0080	0.48	0	0.17	0.77	0.32	58.5	*Polaribacter*	*Flavobacteriaceae*	*Flavobacteriales*	*Flavobacteria*	*Bacteroidetes*	*Bacteria*
Otu0216	0.4	0.4	0.17	0.93	0.31	58.81	*Pelagibacter*	SAR11-clade	*Rickettsiales*	*Alphaproteobacteria*	*Proteobacteria*	*Bacteria*
Otu0148	0	0.48	0.16	0.79	0.31	59.12	unclass. *Sphingobacteriales*	unclass. *Sphingobacteriales*	*Sphingobacteriales*	*Sphingobacteria*	*Bacteroidetes*	*Bacteria*
Otu0728	0.2	0.4	0.16	0.87	0.3	59.42	unclass. *Gammaproteobacteria*	unclass. *Gammaproteobacteria*	unclass. *Gammaproteobacteria*	*Gammaproteobacteria*	*Proteobacteria*	*Bacteria*
Otu0022	0	0.48	0.16	0.77	0.3	59.71	unclass. *Betaproteobacteria*	unclass. *Betaproteobacteria*	unclass. *Betaproteobacteria*	*Betaproteobacteria*	*Proteobacteria*	*Bacteria*
Otu0840	0.49	0	0.16	0.49	0.29	60	unclass. *Flavobacteriaceae*	*Flavobacteriaceae*	*Flavobacteriales*	*Flavobacteria*	*Bacteroidetes*	*Bacteria*
Otu0259	0	0.48	0.16	0.79	0.29	60.3	*Haliscomenobacter*	*Saprospiraceae*	*Sphingobacteriales*	*Sphingobacteria*	*Bacteroidetes*	*Bacteria*
Otu0724	0.4	0.2	0.15	0.87	0.29	60.59	unclass. *Bacteria*	unclass. *Bacteria*	unclass. *Bacteria*	unclass. *Bacteria*	unclass. *Bacteria*	*Bacteria*
Otu0595	0.4	0.2	0.15	0.87	0.29	60.88	unclass. *Legionellaceae*	*Legionellaceae*	*Legionellales*	*Gammaproteobacteria*	*Proteobacteria*	*Bacteria*
Otu0038	0.4	0.2	0.15	0.86	0.29	61.16	unclass. *Proteobacteria*	unclass. *Proteobacteria*	unclass. *Proteobacteria*	unclass. *Proteobacteria*	*Proteobacteria*	*Bacteria*
Otu0482	0.2	0.4	0.15	0.87	0.29	61.45	unclass. *Microbacteriaceae*	*Microbacteriaceae*	*Actinomycetales*	*Actinobacteria*	*Actinobacteria*	*Bacteria*
Otu0735	0.4	0.2	0.15	0.86	0.29	61.74	unclass. *Gammaproteobacteria*	unclass. *Gammaproteobacteria*	unclass. *Gammaproteobacteria*	*Gammaproteobacteria*	*Proteobacteria*	*Bacteria*
Otu0174	0.2	0.4	0.15	0.86	0.28	62.03	unclass. *Flavobacteriaceae*	*Flavobacteriaceae*	*Flavobacteriales*	*Flavobacteria*	*Bacteroidetes*	*Bacteria*
Otu0454	0.2	0.4	0.15	0.86	0.28	62.31	unclass. *Actinobacteria*	unclass. *Actinobacteria*	unclass. *Actinobacteria*	*Actinobacteria*	*Actinobacteria*	*Bacteria*
Otu0012	0.2	0.4	0.15	0.86	0.28	62.59	unclass. *Comamonadaceae*	*Comamonadaceae*	*Burkholderiales*	*Betaproteobacteria*	*Proteobacteria*	*Bacteria*
Otu0464	0.2	0.4	0.15	0.86	0.28	62.87	unclass. *Bacteroidetes*	unclass. *Bacteroidetes*	unclass. *Bacteroidetes*	unclass. *Bacteroidetes*	*Bacteroidetes*	*Bacteria*

OTU	Av.A_i (*pH in situ*)	Av.A_i (pH 7.67)	Av.δ_i	Av.δ_i/SD	Av.δ_i %	$\sum$Av.δ_i %	Genus	Family	Order	Class	Phylum	Domain
Otu0787	0.4	0.2	0.15	0.87	0.28	63.15	unclass. *Verrucomicrobia*	unclass. *Verrucomicrobia*	unclass. *Verrucomicrobia*	unclass. *Verrucomicrobia*	*Verrucomicrobia*	*Bacteria*
Otu0666	0.2	0.28	0.15	0.69	0.27	63.42	unclass. *Deltaproteobacteria*	unclass. *Deltaproteobacteria*	unclass. *Deltaproteobacteria*	*Deltaproteobacteria*	*Proteobacteria*	*Bacteria*
Otu0867	0	0.45	0.14	0.49	0.27	63.69	unclass. *Proteobacteria*	unclass. *Proteobacteria*	unclass. *Proteobacteria*	unclass. *Proteobacteria*	*Proteobacteria*	*Bacteria*
Otu0745	0.4	0	0.14	0.8	0.26	63.95	*Planctomyces*	*Planctomycetaceae*	*Planctomycetales*	*Planctomycetacia*	*Planctomycetes*	*Bacteria*
Otu0746	0.4	0	0.14	0.8	0.26	64.21	unclass. *Flavobacteriaceae*	*Flavobacteriaceae*	*Flavobacteriales*	*Flavobacteria*	*Bacteroidetes*	*Bacteria*
Otu0202	0.4	0	0.14	0.8	0.26	64.48	unclass. *Alteromonadaceae*	*Alteromonadaceae*	*Alteromonadales*	*Gammaproteobacteria*	*Proteobacteria*	*Bacteria*
Otu0051	0.4	0	0.14	0.8	0.26	64.74	unclass. *Flavobacteriaceae*	*Flavobacteriaceae*	*Flavobacteriales*	*Flavobacteria*	*Bacteroidetes*	*Bacteria*
Otu0460	0.28	0.2	0.14	0.68	0.26	65	*Arcobacter*	*Campylobacteraceae*	*Campylobacterales*	*Epsilonproteobacteria*	*Proteobacteria*	*Bacteria*
Otu0783	0.4	0	0.14	0.49	0.26	65.26	unclass. *Gammaproteobacteria*	unclass. *Gammaproteobacteria*	unclass. *Gammaproteobacteria*	*Gammaproteobacteria*	*Proteobacteria*	*Bacteria*
Otu0462	0	0.4	0.14	0.79	0.26	65.52	*Haliea*	*Alteromonadaceae*	*Alteromonadales*	*Gammaproteobacteria*	*Proteobacteria*	*Bacteria*
Otu0555	0.2	0.28	0.14	0.69	0.26	65.78	*Maricaulis*	*Hyphomonadaceae*	*Caulobacterales*	*Alphaproteobacteria*	*Proteobacteria*	*Bacteria*
Otu0727	0.4	0	0.14	0.8	0.25	66.03	unclass. *Deltaproteobacteria*	unclass. *Deltaproteobacteria*	unclass. *Deltaproteobacteria*	*Deltaproteobacteria*	*Proteobacteria*	*Bacteria*
Otu0472	0.4	0	0.14	0.8	0.25	66.29	unclass. *Bacteroidetes*	unclass. *Bacteroidetes*	unclass. *Bacteroidetes*	unclass. *Bacteroidetes*	*Bacteroidetes*	*Bacteria*
Otu0766	0.4	0	0.14	0.8	0.25	66.54	*Lentisphaera*	*Lentisphaeraceae*	*Lentisphaerales*	*Lentisphaeria*	*Lentisphaerae*	*Bacteria*
Otu0774	0.4	0	0.14	0.8	0.25	66.79	unclass. *Bacteria*	unclass. *Bacteria*	unclass. *Bacteria*	unclass. *Bacteria*	unclass. *Bacteria*	*Bacteria*
Otu0477	0	0.4	0.13	0.8	0.25	67.04	*Ilumatobacter*	*Acidimicrobidae_incertae_sedis*	*Actinobacteria_order_incertae_sedis*	*Actinobacteria*	*Actinobacteria*	*Bacteria*
Otu0716	0	0.4	0.13	0.8	0.25	67.29	unclass. *Bacteroidetes*	unclass. *Bacteroidetes*	unclass. *Bacteroidetes*	unclass. *Bacteroidetes*	*Bacteroidetes*	*Bacteria*
Otu0862	0.2	0.28	0.13	0.69	0.25	67.54	unclass. *Gammaproteobacteria*	unclass. *Gammaproteobacteria*	unclass. *Gammaproteobacteria*	*Gammaproteobacteria*	*Proteobacteria*	*Bacteria*
Otu0911	0	0.4	0.13	0.49	0.25	67.79	unclass. *Bacteroidetes*	unclass. *Bacteroidetes*	unclass. *Bacteroidetes*	unclass. *Bacteroidetes*	*Bacteroidetes*	*Bacteria*
Otu0541	0.4	0	0.13	0.8	0.25	68.04	unclass. *Rhodobacteraceae*	*Rhodobacteraceae*	*Rhodobacterales*	*Alphaproteobacteria*	*Proteobacteria*	*Bacteria*
Otu0736	0.4	0	0.13	0.8	0.25	68.29	*Persicirhabdus*	*Verrucomicrobiaceae*	*Verrucomicrobiales*	*Verrucomicrobiae*	*Verrucomicrobia*	*Bacteria*
Otu0811	0.4	0	0.13	0.8	0.25	68.54	unclass. *Bacteria*	unclass. *Bacteria*	unclass. *Bacteria*	unclass. *Bacteria*	unclass. *Bacteria*	*Bacteria*
Otu0255	0.4	0	0.13	0.8	0.25	68.79	*Sphingopyxis*	*Sphingomonadaceae*	*Sphingomonadales*	*Alphaproteobacteria*	*Proteobacteria*	*Bacteria*
Otu0707	0.4	0	0.13	0.8	0.25	69.04	*Haliea*	*Alteromonadaceae*	*Alteromonadales*	*Gammaproteobacteria*	*Proteobacteria*	*Bacteria*
Otu0819	0.4	0	0.13	0.8	0.25	69.29	unclass. *Bacteria*	unclass. *Bacteria*	unclass. *Bacteria*	unclass. *Bacteria*	unclass. *Bacteria*	*Bacteria*
Otu0881	0	0.4	0.13	0.8	0.24	69.53	unclass. *Actinobacteria*	unclass. *Actinobacteria*	unclass. *Actinobacteria*	*Actinobacteria*	*Actinobacteria*	*Bacteria*
Otu0550	0.28	0.2	0.13	0.69	0.24	69.78	unclass. *Flavobacteriales*	unclass. *Flavobacteriales*	*Flavobacteriales*	*Flavobacteria*	*Bacteroidetes*	*Bacteria*

OTU	Av.A_i (*pH in situ*)	Av.A_i (pH 7.67)	Av.δ_i	Av.δ_i/SD	Av.δ_i %	$\sum$Av.δ_i %	Genus	Family	Order	Class	Phylum	Domain
Otu0583	0	0.4	0.13	0.8	0.24	70.02	unclass. *Flavobacteriaceae*	*Flavobacteriaceae*	*Flavobacteriales*	*Flavobacteria*	*Bacteroidetes*	*Bacteria*
Otu0841	0	0.35	0.13	0.49	0.24	70.26	unclass. *Gammaproteobacteria*	unclass. *Gammaproteobacteria*	unclass. *Gammaproteobacteria*	*Gammaproteobacteria*	*Proteobacteria*	*Bacteria*
Otu0072	0.35	0	0.12	0.49	0.23	70.49	*Sulfitobacter*	*Rhodobacteraceae*	*Rhodobacterales*	*Alphaproteobacteria*	*Proteobacteria*	*Bacteria*
Otu0757	0.35	0	0.12	0.49	0.23	70.73	unclass. *Gammaproteobacteria*	unclass. *Gammaproteobacteria*	unclass. *Gammaproteobacteria*	*Gammaproteobacteria*	*Proteobacteria*	*Bacteria*
Otu0789	0.35	0	0.12	0.49	0.22	70.95	unclass. *Proteobacteria*	unclass. *Proteobacteria*	unclass. *Proteobacteria*	unclass. *Proteobacteria*	*Proteobacteria*	*Bacteria*
Otu0814	0.35	0	0.12	0.49	0.22	71.18	unclass. *Proteobacteria*	unclass. *Proteobacteria*	unclass. *Proteobacteria*	unclass. *Proteobacteria*	*Proteobacteria*	*Bacteria*
Otu0912	0	0.35	0.12	0.49	0.22	71.39	unclass. *Gammaproteobacteria*	unclass. *Gammaproteobacteria*	unclass. *Gammaproteobacteria*	*Gammaproteobacteria*	*Proteobacteria*	*Bacteria*
Otu0914	0	0.35	0.12	0.49	0.22	71.61	unclass. *Gammaproteobacteria*	unclass. *Gammaproteobacteria*	unclass. *Gammaproteobacteria*	*Gammaproteobacteria*	*Proteobacteria*	*Bacteria*
Otu0763	0.2	0.2	0.12	0.67	0.22	71.82	*Planctomyces*	*Planctomycetaceae*	*Planctomycetales*	*Planctomycetacia*	*Planctomycetes*	*Bacteria*
Otu0755	0.2	0.2	0.11	0.67	0.22	72.04	unclass. *Proteobacteria*	unclass. *Proteobacteria*	unclass. *Proteobacteria*	unclass. *Proteobacteria*	*Proteobacteria*	*Bacteria*
Otu0044	0.2	0.2	0.11	0.67	0.21	72.25	unclass. *Gammaproteobacteria*	unclass. *Gammaproteobacteria*	unclass. *Gammaproteobacteria*	*Gammaproteobacteria*	*Proteobacteria*	*Bacteria*
Otu0514	0.2	0.2	0.11	0.67	0.21	72.46	unclass. *Microbacteriaceae*	*Microbacteriaceae*	*Actinomycetales*	*Actinobacteria*	*Actinobacteria*	*Bacteria*
Otu0383	0.2	0.2	0.11	0.67	0.21	72.68	unclass. *Actinomycetales*	unclass. *Actinomycetales*	*Actinomycetales*	*Actinobacteria*	*Actinobacteria*	*Bacteria*
Otu0723	0.2	0.2	0.11	0.67	0.21	72.89	unclass. *Microbacteriaceae*	*Microbacteriaceae*	*Actinomycetales*	*Actinobacteria*	*Actinobacteria*	*Bacteria*
Otu0729	0.2	0.2	0.11	0.67	0.21	73.09	unclass. *Bacteroidetes*	unclass. *Bacteroidetes*	unclass. *Bacteroidetes*	unclass. *Bacteroidetes*	*Bacteroidetes*	*Bacteria*
Otu0739	0.2	0.2	0.11	0.67	0.21	73.3	unclass. *Proteobacteria*	unclass. *Proteobacteria*	unclass. *Proteobacteria*	unclass. *Proteobacteria*	*Proteobacteria*	*Bacteria*
Otu0178	0.2	0.2	0.11	0.67	0.21	73.51	unclass. *Actinobacteria*	unclass. *Actinobacteria*	unclass. *Actinobacteria*	*Actinobacteria*	*Actinobacteria*	*Bacteria*
Otu0718	0	0.35	0.11	0.49	0.21	73.71	*Aureispira*	*Saprospiraceae*	*Sphingobacteriales*	*Sphingobacteria*	*Bacteroidetes*	*Bacteria*
Otu0009	0.2	0.2	0.11	0.67	0.2	73.92	unclass. *Rhodobacteraceae*	*Rhodobacteraceae*	*Rhodobacterales*	*Alphaproteobacteria*	*Proteobacteria*	*Bacteria*
Otu0832	0.2	0.2	0.11	0.67	0.2	74.12	unclass. *Rhodospirillaceae*	*Rhodospirillaceae*	*Rhodospirillales*	*Alphaproteobacteria*	*Proteobacteria*	*Bacteria*
Otu0838	0.2	0.2	0.11	0.67	0.2	74.33	unclass. *Bacteroidetes*	unclass. *Bacteroidetes*	unclass. *Bacteroidetes*	unclass. *Bacteroidetes*	*Bacteroidetes*	*Bacteria*
Otu0459	0.2	0.2	0.11	0.67	0.2	74.53	*Croceibacter*	*Flavobacteriaceae*	*Flavobacteriales*	*Flavobacteria*	*Bacteroidetes*	*Bacteria*
Otu0760	0.2	0.2	0.11	0.67	0.2	74.74	unclass. *Bacteria*	unclass. *Bacteria*	unclass. *Bacteria*	unclass. *Bacteria*	unclass. *Bacteria*	*Bacteria*
Otu0717	0.2	0.2	0.11	0.67	0.2	74.94	unclass. *Rhodobacteraceae*	*Rhodobacteraceae*	*Rhodobacterales*	*Alphaproteobacteria*	*Proteobacteria*	*Bacteria*
Otu0770	0.2	0.2	0.11	0.67	0.2	75.14	unclass. *Proteobacteria*	unclass. *Proteobacteria*	unclass. *Proteobacteria*	unclass. *Proteobacteria*	*Proteobacteria*	*Bacteria*
Otu0011	0.2	0.2	0.11	0.67	0.2	75.34	unclass. *Flavobacteriaceae*	*Flavobacteriaceae*	*Flavobacteriales*	*Flavobacteria*	*Bacteroidetes*	*Bacteria*
Otu0720	0.2	0.2	0.11	0.67	0.2	75.54	unclass. *Bacteria*	unclass. *Bacteria*	unclass. *Bacteria*	unclass. *Bacteria*	unclass. *Bacteria*	*Bacteria*

OTU	Av.A$_i$ (*pH in situ*)	Av.A$_i$ (pH 7.67)	Av.δ$_i$	Av.δ$_i$/SD	Av.δ$_i$ %	∑Av.δ$_i$ %	Genus	Family	Order	Class	Phylum	Domain
Otu0738	0.2	0.2	0.11	0.67	0.2	75.74	unclass. *Alphaproteobacteria*	unclass. *Alphaproteobacteria*	unclass. *Alphaproteobacteria*	*Alphaproteobacteria*	*Proteobacteria*	*Bacteria*
Otu0143	0.2	0.2	0.11	0.67	0.2	75.94	unclass. *Gammaproteobacteria*	unclass. *Gammaproteobacteria*	unclass. *Gammaproteobacteria*	*Gammaproteobacteria*	*Proteobacteria*	*Bacteria*
Otu0428	0.2	0.2	0.11	0.67	0.2	76.14	unclass. *Gammaproteobacteria*	unclass. *Gammaproteobacteria*	unclass. *Gammaproteobacteria*	*Gammaproteobacteria*	*Proteobacteria*	*Bacteria*
Otu0035	0.2	0.2	0.1	0.67	0.19	76.33	*Colwellia*	*Colwelliaceae*	*Alteromonadales*	*Gammaproteobacteria*	*Proteobacteria*	*Bacteria*
Otu0017	0.28	0	0.1	0.49	0.19	76.52	unclass. *Flavobacteriales*	unclass. *Flavobacteriales*	*Flavobacteriales*	*Flavobacteria*	*Bacteroidetes*	*Bacteria*
Otu0030	0.28	0	0.1	0.49	0.19	76.71	unclass. *Gammaproteobacteria*	unclass. *Gammaproteobacteria*	unclass. *Gammaproteobacteria*	*Gammaproteobacteria*	*Proteobacteria*	*Bacteria*
Otu0282	0.28	0	0.1	0.49	0.18	76.9	*Haliea*	*Alteromonadaceae*	*Alteromonadales*	*Gammaproteobacteria*	*Proteobacteria*	*Bacteria*
Otu0743	0.28	0	0.1	0.49	0.18	77.08	unclass. *Verrucomicrobia*	unclass. *Verrucomicrobia*	unclass. *Verrucomicrobia*	unclass. *Verrucomicrobia*	*Verrucomicrobia*	*Bacteria*
Otu0761	0.28	0	0.1	0.49	0.18	77.26	unclass. *Verrucomicrobiaceae*	*Verrucomicrobiaceae*	*Verrucomicrobiales*	*Verrucomicrobiae*	*Verrucomicrobia*	*Bacteria*
Otu0810	0.28	0	0.1	0.49	0.18	77.45	unclass. *Gammaproteobacteria*	unclass. *Gammaproteobacteria*	unclass. *Gammaproteobacteria*	*Gammaproteobacteria*	*Proteobacteria*	*Bacteria*
Otu0827	0.28	0	0.1	0.49	0.18	77.63	unclass. *Bacteria*	unclass. *Bacteria*	unclass. *Bacteria*	unclass. *Bacteria*	unclass. *Bacteria*	*Bacteria*
Otu0407	0	0.28	0.09	0.49	0.18	77.81	unclass. *Flavobacteriales*	unclass. *Flavobacteriales*	*Flavobacteriales*	*Flavobacteria*	*Bacteroidetes*	*Bacteria*
Otu0772	0.28	0	0.09	0.49	0.17	77.97	unclass. *Alphaproteobacteria*	unclass. *Alphaproteobacteria*	unclass. *Alphaproteobacteria*	*Alphaproteobacteria*	*Proteobacteria*	*Bacteria*
Otu0839	0.28	0	0.09	0.49	0.17	78.14	unclass. *Bacteria*	unclass. *Bacteria*	unclass. *Bacteria*	unclass. *Bacteria*	unclass. *Bacteria*	*Bacteria*
Otu0491	0	0.2	0.07	0.49	0.14	78.28	unclass. *Flavobacteriales*	unclass. *Flavobacteriales*	*Flavobacteriales*	*Flavobacteria*	*Bacteroidetes*	*Bacteria*
Otu0764	0	0.2	0.07	0.49	0.14	78.42	unclass. *Proteobacteria*	unclass. *Proteobacteria*	unclass. *Proteobacteria*	unclass. *Proteobacteria*	*Proteobacteria*	*Bacteria*
Otu0846	0	0.2	0.07	0.49	0.14	78.56	unclass. *Nannocystaceae*	*Nannocystaceae*	*Myxococcales*	*Deltaproteobacteria*	*Proteobacteria*	*Bacteria*
Otu0858	0	0.2	0.07	0.49	0.14	78.7	unclass. *Flavobacteriaceae*	*Flavobacteriaceae*	*Flavobacteriales*	*Flavobacteria*	*Bacteroidetes*	*Bacteria*
Otu0889	0	0.2	0.07	0.49	0.14	78.84	unclass. *Bacteria*	unclass. *Bacteria*	unclass. *Bacteria*	unclass. *Bacteria*	unclass. *Bacteria*	*Bacteria*
Otu0900	0	0.2	0.07	0.49	0.14	78.98	*Planctomyces*	*Planctomycetaceae*	*Planctomycetales*	*Planctomycetacia*	*Planctomycetes*	*Bacteria*
Otu0901	0	0.2	0.07	0.49	0.14	79.12	unclass. *Bacteria*	unclass. *Bacteria*	unclass. *Bacteria*	unclass. *Bacteria*	unclass. *Bacteria*	*Bacteria*
Otu0902	0	0.2	0.07	0.49	0.14	79.26	unclass. *Gammaproteobacteria*	unclass. *Gammaproteobacteria*	unclass. *Gammaproteobacteria*	*Gammaproteobacteria*	*Proteobacteria*	*Bacteria*
Otu0904	0	0.2	0.07	0.49	0.14	79.4	unclass. *Gammaproteobacteria*	unclass. *Gammaproteobacteria*	unclass. *Gammaproteobacteria*	*Gammaproteobacteria*	*Proteobacteria*	*Bacteria*
Otu0906	0	0.2	0.07	0.49	0.14	79.54	unclass. *Gammaproteobacteria*	unclass. *Gammaproteobacteria*	unclass. *Gammaproteobacteria*	*Gammaproteobacteria*	*Proteobacteria*	*Bacteria*
Otu0086	0	0.2	0.07	0.49	0.14	79.68	*Ilumatobacter*	*Acidimicrobidae_incertae_sedis*	*Actinobacteria_order_incertae_sedis*	*Actinobacteria*	*Actinobacteria*	*Bacteria*
Otu0109	0	0.2	0.07	0.49	0.14	79.82	*Alteromonas*	*Alteromonadaceae*	*Alteromonadales*	*Gammaproteobacteria*	*Proteobacteria*	*Bacteria*
Otu0196	0	0.2	0.07	0.49	0.14	79.96	unclass. *Flavobacteriaceae*	*Flavobacteriaceae*	*Flavobacteriales*	*Flavobacteria*	*Bacteroidetes*	*Bacteria*

OTU	Av.A_i (*pH in situ*)	Av.A_i (pH 7.67)	Av.δ_i	Av.δ_i/SD	Av.δ_i %	∑Av.δ_i %	Genus	Family	Order	Class	Phylum	Domain
Otu0201	0	0.2	0.07	0.49	0.14	80.1	unclass. *Alphaproteobacteria*	unclass. *Alphaproteobacteria*	unclass. *Alphaproteobacteria*	*Alphaproteobacteria*	*Proteobacteria*	*Bacteria*
Otu0465	0	0.2	0.07	0.49	0.14	80.24	unclass. *Bacteroidetes*	unclass. *Bacteroidetes*	unclass. *Bacteroidetes*	unclass. *Bacteroidetes*	*Bacteroidetes*	*Bacteria*
Otu0520	0	0.2	0.07	0.49	0.14	80.37	unclass. *Alphaproteobacteria*	unclass. *Alphaproteobacteria*	unclass. *Alphaproteobacteria*	*Alphaproteobacteria*	*Proteobacteria*	*Bacteria*
Otu0771	0	0.2	0.07	0.49	0.14	80.51	unclass. *Rhodobacteraceae*	*Rhodobacteraceae*	*Rhodobacterales*	*Alphaproteobacteria*	*Proteobacteria*	*Bacteria*
Otu0792	0	0.2	0.07	0.49	0.14	80.65	*Kordia*	*Flavobacteriaceae*	*Flavobacteriales*	*Flavobacteria*	*Bacteroidetes*	*Bacteria*
Otu0948	0	0.2	0.07	0.49	0.14	80.79	unclass. *Bacteria*	unclass. *Bacteria*	unclass. *Bacteria*	unclass. *Bacteria*	unclass. *Bacteria*	*Bacteria*
Otu0949	0	0.2	0.07	0.49	0.14	80.93	unclass. *Gammaproteobacteria*	unclass. *Gammaproteobacteria*	unclass. *Gammaproteobacteria*	*Gammaproteobacteria*	*Proteobacteria*	*Bacteria*
Otu0950	0	0.2	0.07	0.49	0.14	81.07	unclass. *Gammaproteobacteria*	unclass. *Gammaproteobacteria*	unclass. *Gammaproteobacteria*	*Gammaproteobacteria*	*Proteobacteria*	*Bacteria*
Otu0952	0	0.2	0.07	0.49	0.14	81.21	unclass. *Bacteria*	unclass. *Bacteria*	unclass. *Bacteria*	unclass. *Bacteria*	unclass. *Bacteria*	*Bacteria*
Otu0953	0	0.2	0.07	0.49	0.14	81.34	unclass. *Proteobacteria*	unclass. *Proteobacteria*	unclass. *Proteobacteria*	unclass. *Proteobacteria*	*Proteobacteria*	*Bacteria*
Otu0955	0	0.2	0.07	0.49	0.14	81.48	*Lewinella*	*Saprospiraceae*	*Sphingobacteriales*	*Sphingobacteria*	*Bacteroidetes*	*Bacteria*
Otu0956	0	0.2	0.07	0.49	0.14	81.62	unclass. *Flavobacteriaceae*	*Flavobacteriaceae*	*Flavobacteriales*	*Flavobacteria*	*Bacteroidetes*	*Bacteria*
Otu0958	0	0.2	0.07	0.49	0.14	81.76	*Ulvibacter*	*Flavobacteriaceae*	*Flavobacteriales*	*Flavobacteria*	*Bacteroidetes*	*Bacteria*
Otu0027	0.2	0	0.07	0.49	0.13	81.9	*Pseudoalteromonas*	*Pseudoalteromonadaceae*	*Alteromonadales*	*Gammaproteobacteria*	*Proteobacteria*	*Bacteria*
Otu0167	0.2	0	0.07	0.49	0.13	82.03	unclass. *Rhodobacteraceae*	*Rhodobacteraceae*	*Rhodobacterales*	*Alphaproteobacteria*	*Proteobacteria*	*Bacteria*
Otu0213	0.2	0	0.07	0.49	0.13	82.16	unclass. *Gammaproteobacteria*	unclass. *Gammaproteobacteria*	unclass. *Gammaproteobacteria*	*Gammaproteobacteria*	*Proteobacteria*	*Bacteria*
Otu0290	0.2	0	0.07	0.49	0.13	82.3	unclass. *Flavobacteriaceae*	*Flavobacteriaceae*	*Flavobacteriales*	*Flavobacteria*	*Bacteroidetes*	*Bacteria*
Otu0594	0.2	0	0.07	0.49	0.13	82.43	unclass. *Bacteria*	unclass. *Bacteria*	unclass. *Bacteria*	unclass. *Bacteria*	unclass. *Bacteria*	*Bacteria*
Otu0702	0.2	0	0.07	0.49	0.13	82.57	unclass. *Flavobacteriales*	unclass. *Flavobacteriales*	*Flavobacteriales*	*Flavobacteria*	*Bacteroidetes*	*Bacteria*
Otu0709	0.2	0	0.07	0.49	0.13	82.7	unclass. *Bacteria*	unclass. *Bacteria*	unclass. *Bacteria*	unclass. *Bacteria*	unclass. *Bacteria*	*Bacteria*
Otu0712	0.2	0	0.07	0.49	0.13	82.84	unclass. *Alphaproteobacteria*	unclass. *Alphaproteobacteria*	unclass. *Alphaproteobacteria*	*Alphaproteobacteria*	*Proteobacteria*	*Bacteria*
Otu0719	0.2	0	0.07	0.49	0.13	82.97	unclass. *Flavobacteriales*	unclass. *Flavobacteriales*	*Flavobacteriales*	*Flavobacteria*	*Bacteroidetes*	*Bacteria*
Otu0722	0.2	0	0.07	0.49	0.13	83.11	unclass. *Rhodobacteraceae*	*Rhodobacteraceae*	*Rhodobacterales*	*Alphaproteobacteria*	*Proteobacteria*	*Bacteria*
Otu0726	0.2	0	0.07	0.49	0.13	83.24	unclass. *Bacteroidetes*	unclass. *Bacteroidetes*	unclass. *Bacteroidetes*	unclass. *Bacteroidetes*	*Bacteroidetes*	*Bacteria*
Otu0747	0.2	0	0.07	0.49	0.13	83.37	unclass. *Rhodobacteraceae*	*Rhodobacteraceae*	*Rhodobacterales*	*Alphaproteobacteria*	*Proteobacteria*	*Bacteria*
Otu0748	0.2	0	0.07	0.49	0.13	83.51	unclass. *Proteobacteria*	unclass. *Proteobacteria*	unclass. *Proteobacteria*	unclass. *Proteobacteria*	*Proteobacteria*	*Bacteria*
Otu0749	0.2	0	0.07	0.49	0.13	83.64	unclass. *Gammaproteobacteria*	unclass. *Gammaproteobacteria*	unclass. *Gammaproteobacteria*	*Gammaproteobacteria*	*Proteobacteria*	*Bacteria*

OTU	Av.A_i (*pH in situ*)	Av.A_i (pH 7.67)	Av.δ_i	Av.δ_i/SD	Av.δ_i %	∑Av.δ_i %	Genus	Family	Order	Class	Phylum	Domain
Otu0750	0.2	0	0.07	0.49	0.13	83.78	unclass. *Gammaproteobacteria*	unclass. *Gammaproteobacteria*	unclass. *Gammaproteobacteria*	*Gammaproteobacteria*	*Proteobacteria*	*Bacteria*
Otu0083	0.2	0	0.07	0.49	0.13	83.91	unclass. *Rhodobacteraceae*	*Rhodobacteraceae*	*Rhodobacterales*	*Alphaproteobacteria*	*Proteobacteria*	*Bacteria*
Otu0165	0.2	0	0.07	0.49	0.13	84.05	*Marinomonas*	*Oceanospirillaceae*	*Oceanospirillales*	*Gammaproteobacteria*	*Proteobacteria*	*Bacteria*
Otu0381	0.2	0	0.07	0.49	0.13	84.18	unclass. *Chitinophagaceae*	*Chitinophagaceae*	*Sphingobacteriales*	*Sphingobacteria*	*Bacteroidetes*	*Bacteria*
Otu0483	0.2	0	0.07	0.49	0.13	84.31	unclass. *Flavobacteriaceae*	*Flavobacteriaceae*	*Flavobacteriales*	*Flavobacteria*	*Bacteroidetes*	*Bacteria*
Otu0499	0.2	0	0.07	0.49	0.13	84.45	unclass. *Flavobacteriaceae*	*Flavobacteriaceae*	*Flavobacteriales*	*Flavobacteria*	*Bacteroidetes*	*Bacteria*
Otu0730	0.2	0	0.07	0.49	0.13	84.58	unclass. *Proteobacteria*	unclass. *Proteobacteria*	unclass. *Proteobacteria*	unclass. *Proteobacteria*	*Proteobacteria*	*Bacteria*
Otu0756	0.2	0	0.07	0.49	0.13	84.72	unclass. *Bacteroidetes*	unclass. *Bacteroidetes*	unclass. *Bacteroidetes*	unclass. *Bacteroidetes*	*Bacteroidetes*	*Bacteria*
Otu0769	0.2	0	0.07	0.49	0.13	84.85	unclass. *Bacteroidetes*	unclass. *Bacteroidetes*	unclass. *Bacteroidetes*	unclass. *Bacteroidetes*	*Bacteroidetes*	*Bacteria*
Otu0775	0.2	0	0.07	0.49	0.13	84.98	unclass. *Flavobacteriales*	unclass. *Flavobacteriales*	*Flavobacteriales*	*Flavobacteria*	*Bacteroidetes*	*Bacteria*
Otu0777	0.2	0	0.07	0.49	0.13	85.12	unclass. *Flavobacteriaceae*	*Flavobacteriaceae*	*Flavobacteriales*	*Flavobacteria*	*Bacteroidetes*	*Bacteria*
Otu0778	0.2	0	0.07	0.49	0.13	85.25	unclass. *Gammaproteobacteria*	unclass. *Gammaproteobacteria*	unclass. *Gammaproteobacteria*	*Gammaproteobacteria*	*Proteobacteria*	*Bacteria*
Otu0318	0.2	0	0.07	0.49	0.13	85.38	unclass. *Verrucomicrobiaceae*	*Verrucomicrobiaceae*	*Verrucomicrobiales*	*Verrucomicrobiae*	*Verrucomicrobia*	*Bacteria*
Otu0780	0.2	0	0.07	0.49	0.13	85.51	unclass. *Gammaproteobacteria*	unclass. *Gammaproteobacteria*	unclass. *Gammaproteobacteria*	*Gammaproteobacteria*	*Proteobacteria*	*Bacteria*
Otu0784	0.2	0	0.07	0.49	0.13	85.64	unclass. *Bacteria*	unclass. *Bacteria*	unclass. *Bacteria*	unclass. *Bacteria*	unclass. *Bacteria*	*Bacteria*
Otu0785	0.2	0	0.07	0.49	0.13	85.77	unclass. *Bacteroidetes*	unclass. *Bacteroidetes*	unclass. *Bacteroidetes*	unclass. *Bacteroidetes*	*Bacteroidetes*	*Bacteria*
Otu0788	0.2	0	0.07	0.49	0.13	85.9	*Methylohalomonas*	*Gammaproteobacteria_incertae_sedis*	*Gammaproteobacteria_order_incertae_sedis*	*Gammaproteobacteria*	*Proteobacteria*	*Bacteria*
Otu0790	0.2	0	0.07	0.49	0.13	86.03	unclass. *Sphingobacteriales*	unclass. *Sphingobacteriales*	*Sphingobacteriales*	*Sphingobacteria*	*Bacteroidetes*	*Bacteria*
Otu0791	0.2	0	0.07	0.49	0.13	86.16	*Thalassomonas*	*Colwelliaceae*	*Alteromonadales*	*Gammaproteobacteria*	*Proteobacteria*	*Bacteria*
Otu0793	0.2	0	0.07	0.49	0.13	86.29	unclass. *Gammaproteobacteria*	unclass. *Gammaproteobacteria*	unclass. *Gammaproteobacteria*	*Gammaproteobacteria*	*Proteobacteria*	*Bacteria*
Otu0794	0.2	0	0.07	0.49	0.13	86.42	*Legionella*	*Legionellaceae*	*Legionellales*	*Gammaproteobacteria*	*Proteobacteria*	*Bacteria*
Otu0796	0.2	0	0.07	0.49	0.13	86.55	unclass. *Bacteroidetes*	unclass. *Bacteroidetes*	unclass. *Bacteroidetes*	unclass. *Bacteroidetes*	*Bacteroidetes*	*Bacteria*
Otu0797	0.2	0	0.07	0.49	0.13	86.68	unclass. *Bacteria*	unclass. *Bacteria*	unclass. *Bacteria*	unclass. *Bacteria*	unclass. *Bacteria*	*Bacteria*
Otu0799	0.2	0	0.07	0.49	0.13	86.81	unclass. *Gammaproteobacteria*	unclass. *Gammaproteobacteria*	unclass. *Gammaproteobacteria*	*Gammaproteobacteria*	*Proteobacteria*	*Bacteria*
Otu0801	0.2	0	0.07	0.49	0.13	86.94	unclass. *Verrucomicrobiaceae*	*Verrucomicrobiaceae*	*Verrucomicrobiales*	*Verrucomicrobiae*	*Verrucomicrobia*	*Bacteria*
Otu0802	0.2	0	0.07	0.49	0.13	87.07	unclass. *Flammeovirgaceae*	*Flammeovirgaceae*	*Sphingobacteriales*	*Sphingobacteria*	*Bacteroidetes*	*Bacteria*
Otu0804	0.2	0	0.07	0.49	0.13	87.2	unclass. *Bacteria*	unclass. *Bacteria*	unclass. *Bacteria*	unclass. *Bacteria*	unclass. *Bacteria*	*Bacteria*

OTU	Av.A_i (*pH in situ*)	Av.A_i (pH 7.67)	Av.δ_i	Av.δ_i/SD	Av.δ_i %	∑Av.δ_i %	Genus	Family	Order	Class	Phylum	Domain
Otu0805	0.2	0	0.07	0.49	0.13	87.33	unclass. *Proteobacteria*	unclass. *Proteobacteria*	unclass. *Proteobacteria*	unclass. *Proteobacteria*	*Proteobacteria*	*Bacteria*
Otu0806	0.2	0	0.07	0.49	0.13	87.46	*Nocardioides*	*Nocardioidaceae*	*Actinomycetales*	*Actinobacteria*	*Actinobacteria*	*Bacteria*
Otu0813	0.2	0	0.07	0.49	0.13	87.59	unclass. *Flavobacteriaceae*	*Flavobacteriaceae*	*Flavobacteriales*	*Flavobacteria*	*Bacteroidetes*	*Bacteria*
Otu0078	0.2	0	0.07	0.49	0.13	87.72	*Erythrobacter*	*Erythrobacteraceae*	*Sphingomonadales*	*Alphaproteobacteria*	*Proteobacteria*	*Bacteria*
Otu0168	0.2	0	0.07	0.49	0.13	87.84	unclass. *Flavobacteriaceae*	*Flavobacteriaceae*	*Flavobacteriales*	*Flavobacteria*	*Bacteroidetes*	*Bacteria*
Otu0285	0.2	0	0.07	0.49	0.13	87.97	unclass. *Gammaproteobacteria*	unclass. *Gammaproteobacteria*	unclass. *Gammaproteobacteria*	*Gammaproteobacteria*	*Proteobacteria*	*Bacteria*
Otu0335	0.2	0	0.07	0.49	0.13	88.1	unclass. *Flavobacteriaceae*	*Flavobacteriaceae*	*Flavobacteriales*	*Flavobacteria*	*Bacteroidetes*	*Bacteria*
Otu0815	0.2	0	0.07	0.49	0.13	88.23	unclass. *Proteobacteria*	unclass. *Proteobacteria*	unclass. *Proteobacteria*	unclass. *Proteobacteria*	*Proteobacteria*	*Bacteria*
Otu0816	0.2	0	0.07	0.49	0.13	88.36	unclass. *Bacteria*	unclass. *Bacteria*	unclass. *Bacteria*	unclass. *Bacteria*	unclass. *Bacteria*	*Bacteria*
Otu0820	0.2	0	0.07	0.49	0.13	88.49	unclass. *Gammaproteobacteria*	unclass. *Gammaproteobacteria*	unclass. *Gammaproteobacteria*	*Gammaproteobacteria*	*Proteobacteria*	*Bacteria*
Otu0825	0.2	0	0.07	0.49	0.13	88.62	unclass. *Saprospiraceae*	*Saprospiraceae*	*Sphingobacteriales*	*Sphingobacteria*	*Bacteroidetes*	*Bacteria*
Otu0829	0.2	0	0.07	0.49	0.13	88.75	unclass. *Flavobacteriaceae*	*Flavobacteriaceae*	*Flavobacteriales*	*Flavobacteria*	*Bacteroidetes*	*Bacteria*
Otu0830	0.2	0	0.07	0.49	0.13	88.88	unclass. *Gammaproteobacteria*	unclass. *Gammaproteobacteria*	unclass. *Gammaproteobacteria*	*Gammaproteobacteria*	*Proteobacteria*	*Bacteria*
Otu0831	0.2	0	0.07	0.49	0.13	89.01	unclass. *Proteobacteria*	unclass. *Proteobacteria*	unclass. *Proteobacteria*	unclass. *Proteobacteria*	*Proteobacteria*	*Bacteria*
Otu0837	0.2	0	0.07	0.49	0.13	89.14	unclass. *Bacteria*	unclass. *Bacteria*	unclass. *Bacteria*	unclass. *Bacteria*	unclass. *Bacteria*	*Bacteria*
Otu0096	0	0.2	0.07	0.49	0.13	89.27	unclass. *Actinobacteria*	unclass. *Actinobacteria*	unclass. *Actinobacteria*	*Actinobacteria*	*Actinobacteria*	*Bacteria*
Otu0101	0	0.2	0.07	0.49	0.13	89.39	unclass. *Bacteroidetes*	unclass. *Bacteroidetes*	unclass. *Bacteroidetes*	unclass. *Bacteroidetes*	*Bacteroidetes*	*Bacteria*
Otu0291	0	0.2	0.07	0.49	0.13	89.52	unclass. *Flavobacteriaceae*	*Flavobacteriaceae*	*Flavobacteriales*	*Flavobacteria*	*Bacteroidetes*	*Bacteria*
Otu0358	0	0.2	0.07	0.49	0.13	89.64	*Ilumatobacter*	*Acidimicrobidae_incertae_sedis*	*Actinobacteria_order_incertae_ sedis*	*Actinobacteria*	*Actinobacteria*	*Bacteria*
Otu0398	0	0.2	0.07	0.49	0.13	89.77	unclass. *Flavobacteriaceae*	*Flavobacteriaceae*	*Flavobacteriales*	*Flavobacteria*	*Bacteroidetes*	*Bacteria*
Otu0486	0	0.2	0.07	0.49	0.13	89.89	unclass. *Flavobacteriaceae*	*Flavobacteriaceae*	*Flavobacteriales*	*Flavobacteria*	*Bacteroidetes*	*Bacteria*
Otu0652	0	0.2	0.07	0.49	0.13	90.02	*Marinobacter*	*Alteromonadaceae*	*Alteromonadales*	*Gammaproteobacteria*	*Proteobacteria*	*Bacteria*
Summer 'serial dilution' (average dissimilarity: 57.5%)												
Otu0459	10.06	1.53	6.95	1.85	12.08	12.08	*Croceibacter*	*Flavobacteriaceae*	*Flavobacteriales*	*Flavobacteria*	*Bacteroidetes*	*Bacteria*
Otu0471	6.52	13.99	6.25	1.92	10.86	22.95	*Oceaniserpentilla*	*Oceanospirillaceae*	*Oceanospirillales*	*Gammaproteobacteria*	*Proteobacteria*	*Bacteria*
Otu0132	11.11	13.17	5.7	1.28	9.91	32.86	unclass. *Alteromonadales*	unclass. *Alteromonadales*	*Alteromonadales*	*Gammaproteobacteria*	*Proteobacteria*	*Bacteria*
Otu0525	2.2	6.66	3.66	1.95	6.36	39.22	*Reinekea*	*Oceanospirillaceae*	*Oceanospirillales*	*Gammaproteobacteria*	*Proteobacteria*	*Bacteria*

OTU	Av.A_i (*pH in situ*)	Av.A_i (pH 7.67)	Av.δ_i	Av.δ_i/SD	Av.δ_i %	∑Av.δ_i %	Genus	Family	Order	Class	Phylum	Domain
Otu0125	4.15	0.4	3.03	1.61	5.27	44.49	*Leeuwenhoekiella*	*Flavobacteriaceae*	*Flavobacteriales*	*Flavobacteria*	*Bacteroidetes*	*Bacteria*
Otu0521	4.58	1.5	2.85	1.42	4.96	49.46	unclass. *Rhodobacteraceae*	*Rhodobacteraceae*	*Rhodobacterales*	*Alphaproteobacteria*	*Proteobacteria*	*Bacteria*
Otu0460	3.42	0.93	2.22	1.13	3.86	53.32	*Arcobacter*	*Campylobacteraceae*	*Campylobacterales*	*Epsilonproteobacteria*	*Proteobacteria*	*Bacteria*
Otu0523	2.3	0.2	1.65	1.6	2.87	56.19	*Pseudidiomarina*	*Idiomarinaceae*	*Alteromonadales*	*Gammaproteobacteria*	*Proteobacteria*	*Bacteria*
Otu0533	0.55	2.33	1.5	1.26	2.61	58.8	*Nisaea*	*Rhodospirillaceae*	*Rhodospirillales*	*Alphaproteobacteria*	*Proteobacteria*	*Bacteria*
Otu0524	1.85	0	1.41	1.81	2.44	61.24	*Pseudidiomarina*	*Idiomarinaceae*	*Alteromonadales*	*Gammaproteobacteria*	*Proteobacteria*	*Bacteria*
Otu0522	1.75	0	1.32	1.82	2.29	63.53	unclass. *Flavobacteriaceae*	*Flavobacteriaceae*	*Flavobacteriales*	*Flavobacteria*	*Bacteroidetes*	*Bacteria*
Otu0487	1.38	0.2	1.07	0.9	1.86	65.4	unclass. *Chitinophagaceae*	*Chitinophagaceae*	*Sphingobacteriales*	*Sphingobacteria*	*Bacteroidetes*	*Bacteria*
Otu0526	1.39	0.2	1.04	1.08	1.81	67.2	*Maribacter*	*Flavobacteriaceae*	*Flavobacteriales*	*Flavobacteria*	*Bacteroidetes*	*Bacteria*
Otu0537	1.38	0.2	0.98	1.68	1.7	68.9	unclass. *Flavobacteriaceae*	*Flavobacteriaceae*	*Flavobacteriales*	*Flavobacteria*	*Bacteroidetes*	*Bacteria*
Otu0531	1.7	0.88	0.91	1.49	1.58	70.48	unclass. *Flavobacteriaceae*	*Flavobacteriaceae*	*Flavobacteriales*	*Flavobacteria*	*Bacteroidetes*	*Bacteria*
Otu0180	0.48	1.45	0.91	1.41	1.57	72.05	*Reichenbachiella*	*Flammeovirgaceae*	*Sphingobacteriales*	*Sphingobacteria*	*Bacteroidetes*	*Bacteria*
Otu0549	1.26	0	0.87	0.49	1.5	73.56	unclass. *Bacteroidetes*	unclass. *Bacteroidetes*	unclass. *Bacteroidetes*	unclass. *Bacteroidetes*	*Bacteroidetes*	*Bacteria*
Otu0110	0.55	1.01	0.82	1.13	1.42	74.98	unclass. *Rhodobacteraceae*	*Rhodobacteraceae*	*Rhodobacterales*	*Alphaproteobacteria*	*Proteobacteria*	*Bacteria*
Otu0027	1.11	0	0.77	0.69	1.35	76.32	*Pseudoalteromonas*	*Pseudoalteromonadaceae*	*Alteromonadales*	*Gammaproteobacteria*	*Proteobacteria*	*Bacteria*
Otu0123	1.08	0.77	0.77	1.23	1.34	77.66	unclass. *Gammaproteobacteria*	unclass. *Gammaproteobacteria*	unclass. *Gammaproteobacteria*	*Gammaproteobacteria*	*Proteobacteria*	*Bacteria*
Otu0041	0.88	0.2	0.74	0.89	1.29	78.96	*Sulfitobacter*	*Rhodobacteraceae*	*Rhodobacterales*	*Alphaproteobacteria*	*Proteobacteria*	*Bacteria*
Otu0029	1	0	0.68	0.49	1.19	80.15	unclass. *Bacteroidetes*	unclass. *Bacteroidetes*	unclass. *Bacteroidetes*	unclass. *Bacteroidetes*	*Bacteroidetes*	*Bacteria*
Otu0532	0.88	0.48	0.67	1.13	1.17	81.32	unclass. *Rhodobacteraceae*	*Rhodobacteraceae*	*Rhodobacterales*	*Alphaproteobacteria*	*Proteobacteria*	*Bacteria*
Otu0548	0.98	0	0.67	0.49	1.17	82.48	unclass. *Bacteroidetes*	unclass. *Bacteroidetes*	unclass. *Bacteroidetes*	unclass. *Bacteroidetes*	*Bacteroidetes*	*Bacteria*
Otu0527	0.83	0.2	0.63	1.15	1.1	83.58	*Idiomarina*	*Idiomarinaceae*	*Alteromonadales*	*Gammaproteobacteria*	*Proteobacteria*	*Bacteria*
Otu0534	0.4	0.69	0.62	0.92	1.08	84.66	unclass. *Alphaproteobacteria*	unclass. *Alphaproteobacteria*	unclass. *Alphaproteobacteria*	*Alphaproteobacteria*	*Proteobacteria*	*Bacteria*
Otu0109	0.88	0.6	0.61	1.24	1.07	85.73	*Alteromonas*	*Alteromonadaceae*	*Alteromonadales*	*Gammaproteobacteria*	*Proteobacteria*	*Bacteria*
Otu0529	0.48	0.48	0.49	0.97	0.85	86.58	unclass. *Sphingobacteriales*	unclass. *Sphingobacteriales*	*Sphingobacteriales*	*Sphingobacteria*	*Bacteroidetes*	*Bacteria*
Otu0545	0.2	0.55	0.47	0.84	0.82	87.4	*Reinekea*	*Oceanospirillaceae*	*Oceanospirillales*	*Gammaproteobacteria*	*Proteobacteria*	*Bacteria*
Otu0538	0.48	0.4	0.47	1.01	0.81	88.21	*Oceaniserpentilla*	*Oceanospirillaceae*	*Oceanospirillales*	*Gammaproteobacteria*	*Proteobacteria*	*Bacteria*
Otu0535	0.48	0.4	0.46	0.99	0.79	89.01	*Loktanella*	*Rhodobacteraceae*	*Rhodobacterales*	*Alphaproteobacteria*	*Proteobacteria*	*Bacteria*

OTU	Av.A$_i$ (*pH in situ*)	Av.A$_i$ (pH 7.67)	Av.δ$_i$	Av.δ$_i$/SD	Av.δ$_i$ %	∑Av.δ$_i$ %	Genus	Family	Order	Class	Phylum	Domain
Otu0540	0.2	0.4	0.37	0.84	0.64	89.65	unclass. *Flammeovirgaceae*	*Flammeovirgaceae*	*Sphingobacteriales*	*Sphingobacteria*	*Bacteroidetes*	*Bacteria*
Otu0550	0.49	0	0.34	0.49	0.58	90.23	unclass. *Flavobacteriales*	unclass. *Flavobacteriales*	*Flavobacteriales*	*Flavobacteria*	*Bacteroidetes*	*Bacteria*
Summer 'initial dilution' (average dissimilarity: 49.0%)												
Otu0551	1.57	6.75	2.46	1.36	5.01	5.01	*Arcobacter*	*Campylobacteraceae*	*Campylobacterales*	*Epsilonproteobacteria*	*Proteobacteria*	*Bacteria*
Otu0109	18.3	14.05	2.02	1.6	4.12	9.13	*Alteromonas*	*Alteromonadaceae*	*Alteromonadales*	*Gammaproteobacteria*	*Proteobacteria*	*Bacteria*
Otu0588	1.8	4.34	1.23	1.84	2.5	11.63	*Amphritea*	*Oceanospirillaceae*	*Oceanospirillales*	*Gammaproteobacteria*	*Proteobacteria*	*Bacteria*
Otu0234	0.85	2.68	1.14	0.96	2.33	13.96	*Arcobacter*	*Campylobacteraceae*	*Campylobacterales*	*Epsilonproteobacteria*	*Proteobacteria*	*Bacteria*
Otu0041	3.93	2.89	1	1.24	2.04	16	*Sulfitobacter*	*Rhodobacteraceae*	*Rhodobacterales*	*Alphaproteobacteria*	*Proteobacteria*	*Bacteria*
Otu0521	3.2	1.98	0.96	1.47	1.95	17.96	unclass. *Rhodobacteraceae*	*Rhodobacteraceae*	*Rhodobacterales*	*Alphaproteobacteria*	*Proteobacteria*	*Bacteria*
Otu0027	2.28	3.93	0.89	1.59	1.81	19.77	*Pseudoalteromonas*	*Pseudoalteromonadaceae*	*Alteromonadales*	*Gammaproteobacteria*	*Proteobacteria*	*Bacteria*
Otu0583	1.17	2.84	0.84	1.37	1.71	21.47	unclass. *Flavobacteriaceae*	*Flavobacteriaceae*	*Flavobacteriales*	*Flavobacteria*	*Bacteroidetes*	*Bacteria*
Otu0576	1.81	3.39	0.8	1.4	1.63	23.11	unclass. *Alphaproteobacteria*	unclass. *Alphaproteobacteria*	unclass. *Alphaproteobacteria*	*Alphaproteobacteria*	*Proteobacteria*	*Bacteria*
Otu0125	0.48	1.32	0.73	0.81	1.48	24.59	*Leeuwenhoekiella*	*Flavobacteriaceae*	*Flavobacteriales*	*Flavobacteria*	*Bacteroidetes*	*Bacteria*
Otu0110	2.08	0.85	0.72	2.14	1.48	26.07	unclass. *Rhodobacteraceae*	*Rhodobacteraceae*	*Rhodobacterales*	*Alphaproteobacteria*	*Proteobacteria*	*Bacteria*
Otu0035	3.47	2.9	0.7	2.09	1.43	27.5	*Colwellia*	*Colwelliaceae*	*Alteromonadales*	*Gammaproteobacteria*	*Proteobacteria*	*Bacteria*
Otu0578	0.97	2.18	0.64	1.34	1.3	28.8	*Winogradskyella*	*Flavobacteriaceae*	*Flavobacteriales*	*Flavobacteria*	*Bacteroidetes*	*Bacteria*
Otu0072	0	1.29	0.62	3.46	1.25	30.06	*Sulfitobacter*	*Rhodobacteraceae*	*Rhodobacterales*	*Alphaproteobacteria*	*Proteobacteria*	*Bacteria*
Otu0597	0.55	1.29	0.61	1.02	1.25	31.31	*Arenibacter*	*Flavobacteriaceae*	*Flavobacteriales*	*Flavobacteria*	*Bacteroidetes*	*Bacteria*
Otu0346	0.68	1.56	0.58	1.35	1.19	32.5	unclass. *Bacteria*	unclass. *Bacteria*	unclass. *Bacteria*	unclass. *Bacteria*	unclass. *Bacteria*	*Bacteria*
Otu0489	1	2.2	0.58	1.41	1.19	33.68	unclass. *Vibrionaceae*	*Vibrionaceae*	*Vibrionales*	*Gammaproteobacteria*	*Proteobacteria*	*Bacteria*
Otu0542	1.09	1.08	0.57	1.37	1.17	34.85	unclass. *Oceanospirillaceae*	*Oceanospirillaceae*	*Oceanospirillales*	*Gammaproteobacteria*	*Proteobacteria*	*Bacteria*
Otu0533	0.95	1.62	0.55	1.6	1.12	35.98	*Nisaea*	*Rhodospirillaceae*	*Rhodospirillales*	*Alphaproteobacteria*	*Proteobacteria*	*Bacteria*
Otu0532	2.73	2.64	0.55	1.35	1.12	37.1	unclass. *Rhodobacteraceae*	*Rhodobacteraceae*	*Rhodobacterales*	*Alphaproteobacteria*	*Proteobacteria*	*Bacteria*
Otu0569	0.4	1.11	0.53	0.98	1.09	38.19	unclass. *Proteobacteria*	unclass. *Proteobacteria*	unclass. *Proteobacteria*	unclass. *Proteobacteria*	*Proteobacteria*	*Bacteria*
Otu0107	1.25	1.72	0.52	1.51	1.07	39.26	unclass. *Rhodobacteraceae*	*Rhodobacteraceae*	*Rhodobacterales*	*Alphaproteobacteria*	*Proteobacteria*	*Bacteria*
Otu0615	1.12	1.29	0.51	1.3	1.04	40.3	*Neptuniibacter*	*Oceanospirillaceae*	*Oceanospirillales*	*Gammaproteobacteria*	*Proteobacteria*	*Bacteria*
Otu0525	1.62	2.36	0.49	1.42	1	41.3	*Reinekea*	*Oceanospirillaceae*	*Oceanospirillales*	*Gammaproteobacteria*	*Proteobacteria*	*Bacteria*

OTU	Av.A_i (*pH in situ*)	Av.A_i (pH 7.67)	Av.δ_i	Av.δ_i/SD	Av.δ_i %	$\sum$Av.δ_i %	Genus	Family	Order	Class	Phylum	Domain
Otu0460	1.32	1.51	0.48	1.06	0.99	42.29	*Arcobacter*	*Campylobacteraceae*	*Campylobacterales*	*Epsilonproteobacteria*	*Proteobacteria*	*Bacteria*
Otu0553	1.75	2.25	0.46	1.52	0.94	43.23	*Vibrio*	*Vibrionaceae*	*Vibrionales*	*Gammaproteobacteria*	*Proteobacteria*	*Bacteria*
Otu0608	0.48	1.21	0.45	1.34	0.91	44.14	unclass. *Flavobacteriaceae*	*Flavobacteriaceae*	*Flavobacteriales*	*Flavobacteria*	*Bacteroidetes*	*Bacteria*
Otu0534	0	0.96	0.45	0.83	0.91	45.05	unclass. *Alphaproteobacteria*	unclass. *Alphaproteobacteria*	unclass. *Alphaproteobacteria*	*Alphaproteobacteria*	*Proteobacteria*	*Bacteria*
Otu0459	0.68	1.22	0.43	1.21	0.88	45.93	*Croceibacter*	*Flavobacteriaceae*	*Flavobacteriales*	*Flavobacteria*	*Bacteroidetes*	*Bacteria*
Otu0584	0.55	0.85	0.41	1.11	0.84	46.77	unclass. *Proteobacteria*	unclass. *Proteobacteria*	unclass. *Proteobacteria*	unclass. *Proteobacteria*	*Proteobacteria*	*Bacteria*
Otu0112	0.4	0.87	0.4	1.27	0.82	47.59	*Polaribacter*	*Flavobacteriaceae*	*Flavobacteriales*	*Flavobacteria*	*Bacteroidetes*	*Bacteria*
Otu0261	1.24	1.77	0.39	1.14	0.8	48.39	*Winogradskyella*	*Flavobacteriaceae*	*Flavobacteriales*	*Flavobacteria*	*Bacteroidetes*	*Bacteria*
Otu0232	0.8	0.35	0.39	1.11	0.79	49.18	unclass. *Rhodobacteraceae*	*Rhodobacteraceae*	*Rhodobacterales*	*Alphaproteobacteria*	*Proteobacteria*	*Bacteria*
Otu0113	0.55	0.79	0.39	1.1	0.79	49.98	*Colwellia*	*Colwelliaceae*	*Alteromonadales*	*Gammaproteobacteria*	*Proteobacteria*	*Bacteria*
Otu0103	0.6	0.85	0.38	1.42	0.77	50.74	*Pseudomonas*	*Pseudomonadaceae*	*Pseudomonadales*	*Gammaproteobacteria*	*Proteobacteria*	*Bacteria*
Otu0115	2.35	2.56	0.37	2.06	0.76	51.51	*Glaciecola*	*Alteromonadaceae*	*Alteromonadales*	*Gammaproteobacteria*	*Proteobacteria*	*Bacteria*
Otu0600	0.28	0.85	0.37	1.46	0.76	52.27	*Jannaschia*	*Rhodobacteraceae*	*Rhodobacterales*	*Alphaproteobacteria*	*Proteobacteria*	*Bacteria*
Otu0297	0.2	0.79	0.37	1.08	0.76	53.02	*Lutibacter*	*Flavobacteriaceae*	*Flavobacteriales*	*Flavobacteria*	*Bacteroidetes*	*Bacteria*
Otu0111	0.4	0.79	0.37	1.18	0.75	53.77	*Marinomonas*	*Oceanospirillaceae*	*Oceanospirillales*	*Gammaproteobacteria*	*Proteobacteria*	*Bacteria*
Otu0580	0.91	1.37	0.37	1.3	0.75	54.52	*Neptuniibacter*	*Oceanospirillaceae*	*Oceanospirillales*	*Gammaproteobacteria*	*Proteobacteria*	*Bacteria*
Otu0036	0.95	0.71	0.37	1.42	0.74	55.27	*Glaciecola*	*Alteromonadaceae*	*Alteromonadales*	*Gammaproteobacteria*	*Proteobacteria*	*Bacteria*
Otu0599	0.8	0.35	0.36	1.78	0.74	56	*Winogradskyella*	*Flavobacteriaceae*	*Flavobacteriales*	*Flavobacteria*	*Bacteroidetes*	*Bacteria*
Otu0263	0.4	0.75	0.36	1.01	0.73	56.73	*Krokinobacter*	*Flavobacteriaceae*	*Flavobacteriales*	*Flavobacteria*	*Bacteroidetes*	*Bacteria*
Otu0626	0.2	0.75	0.35	0.95	0.71	57.44	*Marinomonas*	*Oceanospirillaceae*	*Oceanospirillales*	*Gammaproteobacteria*	*Proteobacteria*	*Bacteria*
Otu0108	0	0.75	0.35	1.67	0.71	58.15	unclass. *Colwelliaceae*	*Colwelliaceae*	*Alteromonadales*	*Gammaproteobacteria*	*Proteobacteria*	*Bacteria*
Otu0526	0.2	0.66	0.35	0.71	0.71	58.86	*Maribacter*	*Flavobacteriaceae*	*Flavobacteriales*	*Flavobacteria*	*Bacteroidetes*	*Bacteria*
Otu0535	0.75	0	0.34	0.49	0.69	59.55	*Loktanella*	*Rhodobacteraceae*	*Rhodobacterales*	*Alphaproteobacteria*	*Proteobacteria*	*Bacteria*
Otu0536	0.4	1.1	0.34	1.3	0.69	60.24	*Maribacter*	*Flavobacteriaceae*	*Flavobacteriales*	*Flavobacteria*	*Bacteroidetes*	*Bacteria*
Otu0165	0.83	0.85	0.33	1.22	0.68	60.92	*Marinomonas*	*Oceanospirillaceae*	*Oceanospirillales*	*Gammaproteobacteria*	*Proteobacteria*	*Bacteria*
Otu0577	0.73	0	0.32	0.77	0.66	61.58	unclass. *Bacteroidetes*	unclass. *Bacteroidetes*	unclass. *Bacteroidetes*	unclass. *Bacteroidetes*	*Bacteroidetes*	*Bacteria*
Otu0010	0.68	0	0.3	0.78	0.62	62.2	unclass. *Flavobacteriaceae*	*Flavobacteriaceae*	*Flavobacteriales*	*Flavobacteria*	*Bacteroidetes*	*Bacteria*

OTU	Av.A_i (*pH in situ*)	Av.A_i (pH 7.67)	Av.δ_i	Av.δ_i/SD	Av.δ_i %	$\sum$Av.δ_i %	Genus	Family	Order	Class	Phylum	Domain
Otu0487	2.26	2.54	0.3	1.12	0.62	62.82	unclass. *Chitinophagaceae*	*Chitinophagaceae*	*Sphingobacteriales*	*Sphingobacteria*	*Bacteroidetes*	*Bacteria*
Otu0434	0.4	0.6	0.29	1.05	0.6	63.41	*Maribacter*	*Flavobacteriaceae*	*Flavobacteriales*	*Flavobacteria*	*Bacteroidetes*	*Bacteria*
Otu0244	0.55	0.25	0.29	0.87	0.59	64.01	unclass. *Rhodobacteraceae*	*Rhodobacteraceae*	*Rhodobacterales*	*Alphaproteobacteria*	*Proteobacteria*	*Bacteria*
Otu0575	0.65	0	0.29	0.71	0.59	64.6	unclass. *Bacteroidetes*	unclass. *Bacteroidetes*	unclass. *Bacteroidetes*	unclass. *Bacteroidetes*	*Bacteroidetes*	*Bacteria*
Otu0596	0.48	0.35	0.28	0.9	0.58	65.17	*Neptunomonas*	*Oceanospirillaceae*	*Oceanospirillales*	*Gammaproteobacteria*	*Proteobacteria*	*Bacteria*
Otu0649	0	0.6	0.28	0.95	0.57	65.75	unclass. *Bacteroidetes*	unclass. *Bacteroidetes*	unclass. *Bacteroidetes*	unclass. *Bacteroidetes*	*Bacteroidetes*	*Bacteria*
Otu0320	0.4	0.6	0.28	1.06	0.57	66.32	*Pseudoalteromonas*	*Pseudoalteromonadaceae*	*Alteromonadales*	*Gammaproteobacteria*	*Proteobacteria*	*Bacteria*
Otu0543	0.48	0.35	0.27	0.91	0.56	66.88	*Muricauda*	*Flavobacteriaceae*	*Flavobacteriales*	*Flavobacteria*	*Bacteroidetes*	*Bacteria*
Otu0607	0.55	0	0.27	0.73	0.55	67.43	unclass. *Flavobacteriaceae*	*Flavobacteriaceae*	*Flavobacteriales*	*Flavobacteria*	*Bacteroidetes*	*Bacteria*
Otu0593	0.4	0.35	0.26	0.95	0.53	67.96	*Psychroserpens*	*Flavobacteriaceae*	*Flavobacteriales*	*Flavobacteria*	*Bacteroidetes*	*Bacteria*
Otu0591	0.2	0.43	0.25	0.73	0.52	68.47	*Winogradskyella*	*Flavobacteriaceae*	*Flavobacteriales*	*Flavobacteria*	*Bacteroidetes*	*Bacteria*
Otu0639	0	0.5	0.25	0.97	0.51	68.98	unclass. *Rhodobacteraceae*	*Rhodobacteraceae*	*Rhodobacterales*	*Alphaproteobacteria*	*Proteobacteria*	*Bacteria*
Otu0634	0.2	0.5	0.24	0.97	0.5	69.48	*Winogradskyella*	*Flavobacteriaceae*	*Flavobacteriales*	*Flavobacteria*	*Bacteroidetes*	*Bacteria*
Otu0544	0.2	0.5	0.24	0.97	0.5	69.97	*Alteromonas*	*Alteromonadaceae*	*Alteromonadales*	*Gammaproteobacteria*	*Proteobacteria*	*Bacteria*
Otu0663	0	0.5	0.24	0.97	0.49	70.46	unclass. *Proteobacteria*	unclass. *Proteobacteria*	unclass. *Proteobacteria*	unclass. *Proteobacteria*	*Proteobacteria*	*Bacteria*
Otu0581	0	0.5	0.23	0.97	0.48	70.94	*Polaribacter*	*Flavobacteriaceae*	*Flavobacteriales*	*Flavobacteria*	*Bacteroidetes*	*Bacteria*
Otu0653	0	0.5	0.23	0.97	0.48	71.42	*Winogradskyella*	*Flavobacteriaceae*	*Flavobacteriales*	*Flavobacteria*	*Bacteroidetes*	*Bacteria*
Otu0324	0.48	0	0.23	0.79	0.47	71.89	*Nisaea*	*Rhodospirillaceae*	*Rhodospirillales*	*Alphaproteobacteria*	*Proteobacteria*	*Bacteria*
Otu0547	0.48	0	0.23	0.76	0.47	72.36	*Phenylobacterium*	*Caulobacteraceae*	*Caulobacterales*	*Alphaproteobacteria*	*Proteobacteria*	*Bacteria*
Otu0423	0.48	0	0.22	0.77	0.44	72.8	unclass. *Flavobacteriaceae*	*Flavobacteriaceae*	*Flavobacteriales*	*Flavobacteria*	*Bacteroidetes*	*Bacteria*
Otu0595	0.48	0	0.22	0.77	0.44	73.24	unclass. *Legionellaceae*	*Legionellaceae*	*Legionellales*	*Gammaproteobacteria*	*Proteobacteria*	*Bacteria*
Otu0632	0.49	0	0.21	0.49	0.44	73.67	unclass. *Flavobacteriaceae*	*Flavobacteriaceae*	*Flavobacteriales*	*Flavobacteria*	*Bacteroidetes*	*Bacteria*
Otu0598	0.4	0.25	0.21	0.88	0.44	74.11	*Arcobacter*	*Campylobacteraceae*	*Campylobacterales*	*Epsilonproteobacteria*	*Proteobacteria*	*Bacteria*
Otu0628	0.4	0.25	0.21	0.87	0.43	74.54	*Lacinutrix*	*Flavobacteriaceae*	*Flavobacteriales*	*Flavobacteria*	*Bacteroidetes*	*Bacteria*
Otu0555	0.4	0.25	0.21	0.88	0.42	74.96	*Maricaulis*	*Hyphomonadaceae*	*Caulobacterales*	*Alphaproteobacteria*	*Proteobacteria*	*Bacteria*
Otu0641	0.28	0.25	0.2	0.75	0.41	75.37	*Flavobacterium*	*Flavobacteriaceae*	*Flavobacteriales*	*Flavobacteria*	*Bacteroidetes*	*Bacteria*
Otu0568	0.4	0	0.19	0.79	0.39	75.76	unclass. *Flavobacteriales*	unclass. *Flavobacteriales*	*Flavobacteriales*	*Flavobacteria*	*Bacteroidetes*	*Bacteria*

OTU	Av.A_i (*pH in situ*)	Av.A_i (pH 7.67)	Av.δ_i	Av.δ_i/SD	Av.δ_i %	∑Av.δ_i %	Genus	Family	Order	Class	Phylum	Domain
Otu0604	0.4	0	0.19	0.79	0.39	76.15	unclass. *Bacteroidetes*	unclass. *Bacteroidetes*	unclass. *Bacteroidetes*	unclass. *Bacteroidetes*	*Bacteroidetes*	*Bacteria*
Otu0564	0.4	0	0.18	0.49	0.37	76.53	unclass. *Bacteroidetes*	unclass. *Bacteroidetes*	unclass. *Bacteroidetes*	unclass. *Bacteroidetes*	*Bacteroidetes*	*Bacteria*
Otu0616	0.4	0	0.18	0.49	0.37	76.9	unclass. *Bacteria*	unclass. *Bacteria*	unclass. *Bacteria*	unclass. *Bacteria*	unclass. *Bacteria*	*Bacteria*
Otu0582	0.4	0	0.18	0.79	0.37	77.27	unclass. *Rhodobacteraceae*	*Rhodobacteraceae*	*Rhodobacterales*	*Alphaproteobacteria*	*Proteobacteria*	*Bacteria*
Otu0132	0	0.35	0.18	0.56	0.37	77.64	unclass. *Alteromonadales*	unclass. *Alteromonadales*	*Alteromonadales*	*Gammaproteobacteria*	*Proteobacteria*	*Bacteria*
Otu0500	0.4	0	0.18	0.79	0.36	78	*Marinobacter*	*Alteromonadaceae*	*Alteromonadales*	*Gammaproteobacteria*	*Proteobacteria*	*Bacteria*
Otu0054	0.2	0.25	0.17	0.71	0.34	78.34	*Pseudomonas*	*Pseudomonadaceae*	*Pseudomonadales*	*Gammaproteobacteria*	*Proteobacteria*	*Bacteria*
Otu0180	0.2	0.25	0.17	0.71	0.34	78.68	*Reichenbachiella*	*Flammeovirgaceae*	*Sphingobacteriales*	*Sphingobacteria*	*Bacteroidetes*	*Bacteria*
Otu0005	0.2	0.25	0.17	0.71	0.34	79.02	*Pelagibacter*	SAR11-clade	*Rickettsiales*	*Alphaproteobacteria*	*Proteobacteria*	*Bacteria*
Otu0561	0.2	0.25	0.17	0.71	0.34	79.36	*Cellulophaga*	*Flavobacteriaceae*	*Flavobacteriales*	*Flavobacteria*	*Bacteroidetes*	*Bacteria*
Otu0603	0.2	0.25	0.17	0.71	0.34	79.7	unclass. *Sphingobacteriales*	unclass. *Sphingobacteriales*	*Sphingobacteriales*	*Sphingobacteria*	*Bacteroidetes*	*Bacteria*
Otu0631	0.2	0.25	0.17	0.71	0.34	80.04	*Arcobacter*	*Campylobacteraceae*	*Campylobacterales*	*Epsilonproteobacteria*	*Proteobacteria*	*Bacteria*
Otu0633	0.2	0.25	0.17	0.71	0.34	80.38	*Neptuniibacter*	*Oceanospirillaceae*	*Oceanospirillales*	*Gammaproteobacteria*	*Proteobacteria*	*Bacteria*
Otu0617	0.2	0.25	0.16	0.71	0.33	80.71	*Algoriphagus*	*Cyclobacteriaceae*	*Sphingobacteriales*	*Sphingobacteria*	*Bacteroidetes*	*Bacteria*
Otu0563	0	0.35	0.16	0.56	0.33	81.03	unclass. *Oceanospirillaceae*	*Oceanospirillaceae*	*Oceanospirillales*	*Gammaproteobacteria*	*Proteobacteria*	*Bacteria*
Otu0680	0	0.35	0.16	0.56	0.33	81.36	unclass. *Bacteria*	unclass. *Bacteria*	unclass. *Bacteria*	unclass. *Bacteria*	unclass. *Bacteria*	*Bacteria*
Otu0539	0.2	0.25	0.16	0.71	0.32	81.68	unclass. *Bacteroidetes*	unclass. *Bacteroidetes*	unclass. *Bacteroidetes*	unclass. *Bacteroidetes*	*Bacteroidetes*	*Bacteria*
Otu0579	0.35	0	0.16	0.49	0.32	82	unclass. *Bacteroidetes*	unclass. *Bacteroidetes*	unclass. *Bacteroidetes*	unclass. *Bacteroidetes*	*Bacteroidetes*	*Bacteria*
Otu0618	0.28	0	0.13	0.49	0.26	82.27	*Glaciecola*	*Alteromonadaceae*	*Alteromonadales*	*Gammaproteobacteria*	*Proteobacteria*	*Bacteria*
Otu0574	0.28	0	0.13	0.49	0.26	82.53	unclass. *Alphaproteobacteria*	unclass. *Alphaproteobacteria*	unclass. *Alphaproteobacteria*	*Alphaproteobacteria*	*Proteobacteria*	*Bacteria*
Otu0585	0.28	0	0.13	0.49	0.26	82.79	*Neptuniibacter*	*Oceanospirillaceae*	*Oceanospirillales*	*Gammaproteobacteria*	*Proteobacteria*	*Bacteria*
Otu0127	0	0.25	0.13	0.56	0.26	83.05	*Pseudomonas*	*Pseudomonadaceae*	*Pseudomonadales*	*Gammaproteobacteria*	*Proteobacteria*	*Bacteria*
Otu0301	0	0.25	0.13	0.56	0.26	83.31	*Paracoccus*	*Rhodobacteraceae*	*Rhodobacterales*	*Alphaproteobacteria*	*Proteobacteria*	*Bacteria*
Otu0545	0	0.25	0.13	0.56	0.26	83.57	*Reinekea*	*Oceanospirillaceae*	*Oceanospirillales*	*Gammaproteobacteria*	*Proteobacteria*	*Bacteria*
Otu0606	0	0.25	0.13	0.56	0.26	83.83	*Polaribacter*	*Flavobacteriaceae*	*Flavobacteriales*	*Flavobacteria*	*Bacteroidetes*	*Bacteria*
Otu0646	0	0.25	0.13	0.56	0.26	84.09	unclass. *Rhodobacteraceae*	*Rhodobacteraceae*	*Rhodobacterales*	*Alphaproteobacteria*	*Proteobacteria*	*Bacteria*
Otu0675	0	0.25	0.13	0.56	0.26	84.35	unclass. *Flavobacteriales*	unclass. *Flavobacteriales*	*Flavobacteriales*	*Flavobacteria*	*Bacteroidetes*	*Bacteria*

OTU	Av.A_i (*pH in situ*)	Av.A_i (pH 7.67)	Av.δ_i	Av.δ_i/SD	Av.δ_i %	∑Av.δ_i %	Genus	Family	Order	Class	Phylum	Domain
Otu0676	0	0.25	0.13	0.56	0.26	84.6	unclass. *Bacteria*	unclass. *Bacteria*	unclass. *Bacteria*	unclass. *Bacteria*	unclass. *Bacteria*	*Bacteria*
Otu0678	0	0.25	0.13	0.56	0.26	84.86	*Alteromonas*	*Alteromonadaceae*	*Alteromonadales*	*Gammaproteobacteria*	*Proteobacteria*	*Bacteria*
Otu0679	0	0.25	0.13	0.56	0.26	85.12	unclass. *Rhodobacteraceae*	*Rhodobacteraceae*	*Rhodobacterales*	*Alphaproteobacteria*	*Proteobacteria*	*Bacteria*
Otu0635	0.28	0	0.12	0.49	0.25	85.37	unclass. *Rhodobacteraceae*	*Rhodobacteraceae*	*Rhodobacterales*	*Alphaproteobacteria*	*Proteobacteria*	*Bacteria*
Otu0644	0.28	0	0.12	0.49	0.25	85.63	*Polaribacter*	*Flavobacteriaceae*	*Flavobacteriales*	*Flavobacteria*	*Bacteroidetes*	*Bacteria*
Otu0039	0	0.25	0.12	0.56	0.25	85.87	*Pseudoalteromonas*	*Pseudoalteromonadaceae*	*Alteromonadales*	*Gammaproteobacteria*	*Proteobacteria*	*Bacteria*
Otu0124	0	0.25	0.12	0.56	0.25	86.12	*Loktanella*	*Rhodobacteraceae*	*Rhodobacterales*	*Alphaproteobacteria*	*Proteobacteria*	*Bacteria*
Otu0189	0	0.25	0.12	0.56	0.25	86.37	*Psychrobacter*	*Moraxellaceae*	*Pseudomonadales*	*Gammaproteobacteria*	*Proteobacteria*	*Bacteria*
Otu0193	0	0.25	0.12	0.56	0.25	86.61	unclass. *Rhodobacteraceae*	*Rhodobacteraceae*	*Rhodobacterales*	*Alphaproteobacteria*	*Proteobacteria*	*Bacteria*
Otu0645	0	0.25	0.12	0.56	0.25	86.86	*Arcobacter*	*Campylobacteraceae*	*Campylobacterales*	*Epsilonproteobacteria*	*Proteobacteria*	*Bacteria*
Otu0647	0	0.25	0.12	0.56	0.25	87.1	*Maribacter*	*Flavobacteriaceae*	*Flavobacteriales*	*Flavobacteria*	*Bacteroidetes*	*Bacteria*
Otu0648	0	0.25	0.12	0.56	0.25	87.35	*Terasakiella*	*Methylocystaceae*	*Rhizobiales*	*Alphaproteobacteria*	*Proteobacteria*	*Bacteria*
Otu0652	0	0.25	0.12	0.56	0.25	87.6	*Marinobacter*	*Alteromonadaceae*	*Alteromonadales*	*Gammaproteobacteria*	*Proteobacteria*	*Bacteria*
Otu0654	0	0.25	0.12	0.56	0.25	87.84	*Marinobacter*	*Alteromonadaceae*	*Alteromonadales*	*Gammaproteobacteria*	*Proteobacteria*	*Bacteria*
Otu0207	0	0.25	0.11	0.56	0.23	88.08	unclass. *Flavobacteriaceae*	*Flavobacteriaceae*	*Flavobacteriales*	*Flavobacteria*	*Bacteroidetes*	*Bacteria*
Otu0554	0	0.25	0.11	0.56	0.23	88.31	unclass. *Rhodobacteraceae*	*Rhodobacteraceae*	*Rhodobacterales*	*Alphaproteobacteria*	*Proteobacteria*	*Bacteria*
Otu0592	0	0.25	0.11	0.56	0.23	88.54	*Pseudidiomarina*	*Idiomarinaceae*	*Alteromonadales*	*Gammaproteobacteria*	*Proteobacteria*	*Bacteria*
Otu0681	0	0.25	0.11	0.56	0.23	88.77	unclass. *Bacteria*	unclass. *Bacteria*	unclass. *Bacteria*	unclass. *Bacteria*	unclass. *Bacteria*	*Bacteria*
Otu0683	0	0.25	0.11	0.56	0.23	89	*Tenacibaculum*	*Flavobacteriaceae*	*Flavobacteriales*	*Flavobacteria*	*Bacteroidetes*	*Bacteria*
Otu0684	0	0.25	0.11	0.56	0.23	89.23	unclass. *Rhodobacteraceae*	*Rhodobacteraceae*	*Rhodobacterales*	*Alphaproteobacteria*	*Proteobacteria*	*Bacteria*
Otu0685	0	0.25	0.11	0.56	0.23	89.46	unclass. *Alphaproteobacteria*	unclass. *Alphaproteobacteria*	unclass. *Alphaproteobacteria*	*Alphaproteobacteria*	*Proteobacteria*	*Bacteria*
Otu0686	0	0.25	0.11	0.56	0.23	89.69	*Marinomonas*	*Oceanospirillaceae*	*Oceanospirillales*	*Gammaproteobacteria*	*Proteobacteria*	*Bacteria*
Otu0687	0	0.25	0.11	0.56	0.23	89.92	unclass. *Bacteria*	unclass. *Bacteria*	unclass. *Bacteria*	unclass. *Bacteria*	unclass. *Bacteria*	*Bacteria*
Otu0688	0	0.25	0.11	0.56	0.23	90.16	unclass. *Oceanospirillaceae*	*Oceanospirillaceae*	*Oceanospirillales*	*Gammaproteobacteria*	*Proteobacteria*	*Bacteria*
Autumn 'no dilution' (average dissimilarity: 62.8%)												
Otu1090	5.7	2.33	0.63	3.11	1	1	unclass. *Bacteria*	unclass. *Bacteria*	unclass. *Bacteria*	unclass. *Bacteria*	unclass. *Bacteria*	*Bacteria*
Otu0001	4.28	1.18	0.6	1.37	0.96	1.96	unclass. *Flavobacteriaceae*	*Flavobacteriaceae*	*Flavobacteriales*	*Flavobacteria*	*Bacteroidetes*	*Bacteria*

OTU	Av.A_i (*pH in situ*)	Av.A_i (pH 7.67)	Av.δ_i	Av.δ_i/SD	Av.δ_i %	∑Av.δ_i %	Genus	Family	Order	Class	Phylum	Domain
Otu0735	0	2.76	0.52	4.3	0.83	2.79	unclass. *Gammaproteobacteria*	unclass. *Gammaproteobacteria*	unclass. *Gammaproteobacteria*	*Gammaproteobacteria*	*Proteobacteria*	*Bacteria*
Otu0005	4.32	3.46	0.43	1.34	0.68	3.47	*Pelagibacter*	SAR11-clade	*Rickettsiales*	*Alphaproteobacteria*	*Proteobacteria*	*Bacteria*
Otu0691	1.94	4.06	0.4	1.86	0.64	4.11	*Planctomyces*	*Planctomycetaceae*	*Planctomycetales*	*Planctomycetacia*	*Planctomycetes*	*Bacteria*
Otu0272	3.28	1.31	0.4	2.05	0.63	4.74	unclass. *Flavobacteriaceae*	*Flavobacteriaceae*	*Flavobacteriales*	*Flavobacteria*	*Bacteroidetes*	*Bacteria*
Otu0830	0.2	2.18	0.37	2.88	0.6	5.33	unclass. *Gammaproteobacteria*	unclass. *Gammaproteobacteria*	unclass. *Gammaproteobacteria*	*Gammaproteobacteria*	*Proteobacteria*	*Bacteria*
Otu0408	2.67	0.68	0.37	3.28	0.6	5.93	unclass. *Flavobacteriaceae*	*Flavobacteriaceae*	*Flavobacteriales*	*Flavobacteria*	*Bacteroidetes*	*Bacteria*
Otu0983	2.52	0.55	0.37	2.04	0.59	6.52	unclass. *Flavobacteriales*	unclass. *Flavobacteriales*	*Flavobacteriales*	*Flavobacteria*	*Bacteroidetes*	*Bacteria*
Otu0765	0.68	2.45	0.33	2.5	0.53	7.04	*Pelagicoccus*	*Puniceicoccaceae*	*Puniceicoccales*	*Opitutae*	*Verrucomicrobia*	*Bacteria*
Otu0776	1.76	0	0.33	3.23	0.52	7.57	unclass. *Proteobacteria*	unclass. *Proteobacteria*	unclass. *Proteobacteria*	unclass. *Proteobacteria*	*Proteobacteria*	*Bacteria*
Otu0764	2.18	0.48	0.32	2.61	0.51	8.08	unclass. *Proteobacteria*	unclass. *Proteobacteria*	unclass. *Proteobacteria*	unclass. *Proteobacteria*	*Proteobacteria*	*Bacteria*
Otu1092	1.84	1.89	0.31	1.14	0.5	8.58	*Cycloclasticus*	*Piscirickettsiaceae*	*Thiotrichales*	*Gammaproteobacteria*	*Proteobacteria*	*Bacteria*
Otu0494	2.08	0.48	0.3	1.65	0.48	9.06	unclass. *Flavobacteriaceae*	*Flavobacteriaceae*	*Flavobacteriales*	*Flavobacteria*	*Bacteroidetes*	*Bacteria*
Otu0823	3.6	4.66	0.29	2.11	0.46	9.52	unclass. *Legionellaceae*	*Legionellaceae*	*Legionellales*	*Gammaproteobacteria*	*Proteobacteria*	*Bacteria*
Otu0467	2.24	1.01	0.28	1.48	0.45	9.97	unclass. *Rhodospirillaceae*	*Rhodospirillaceae*	*Rhodospirillales*	*Alphaproteobacteria*	*Proteobacteria*	*Bacteria*
Otu1062	0.2	1.68	0.28	2.38	0.45	10.42	unclass. *Gammaproteobacteria*	unclass. *Gammaproteobacteria*	unclass. *Gammaproteobacteria*	*Gammaproteobacteria*	*Proteobacteria*	*Bacteria*
Otu0972	1.48	0	0.28	3.46	0.44	10.86	*Nisaea*	*Rhodospirillaceae*	*Rhodospirillales*	*Alphaproteobacteria*	*Proteobacteria*	*Bacteria*
Otu0261	1.17	2.47	0.26	1.44	0.41	11.27	*Winogradskyella*	*Flavobacteriaceae*	*Flavobacteriales*	*Flavobacteria*	*Bacteroidetes*	*Bacteria*
Otu1105	1.37	0	0.26	4.09	0.41	11.68	unclass. *Proteobacteria*	unclass. *Proteobacteria*	unclass. *Proteobacteria*	unclass. *Proteobacteria*	*Proteobacteria*	*Bacteria*
Otu1102	0.93	1.21	0.25	1.19	0.4	12.08	unclass. *Proteobacteria*	unclass. *Proteobacteria*	unclass. *Proteobacteria*	unclass. *Proteobacteria*	*Proteobacteria*	*Bacteria*
Otu0495	0.4	1.71	0.25	1.53	0.4	12.47	unclass. *Flavobacteriales*	unclass. *Flavobacteriales*	*Flavobacteriales*	*Flavobacteria*	*Bacteroidetes*	*Bacteria*
Otu0681	2.85	1.59	0.25	1.52	0.39	12.87	unclass. *Bacteria*	unclass. *Bacteria*	unclass. *Bacteria*	unclass. *Bacteria*	unclass. *Bacteria*	*Bacteria*
Otu0705	2.29	2.94	0.25	1.13	0.39	13.26	*Planctomyces*	*Planctomycetaceae*	*Planctomycetales*	*Planctomycetacia*	*Planctomycetes*	*Bacteria*
Otu0157	1.04	1.46	0.24	1.22	0.39	13.65	unclass. *Gammaproteobacteria*	unclass. *Gammaproteobacteria*	unclass. *Gammaproteobacteria*	*Gammaproteobacteria*	*Proteobacteria*	*Bacteria*
Otu1097	1.77	0.48	0.24	1.84	0.39	14.03	unclass. *Proteobacteria*	unclass. *Proteobacteria*	unclass. *Proteobacteria*	unclass. *Proteobacteria*	*Proteobacteria*	*Bacteria*
Otu0003	0	1.21	0.23	1.38	0.37	14.4	unclass. *Rhodobacteraceae*	*Rhodobacteraceae*	*Rhodobacterales*	*Alphaproteobacteria*	*Proteobacteria*	*Bacteria*
Otu1386	0	1.21	0.23	1.11	0.36	14.76	unclass. *Bacteria*	unclass. *Bacteria*	unclass. *Bacteria*	unclass. *Bacteria*	unclass. *Bacteria*	*Bacteria*
Otu0359	0.48	1.68	0.23	1.85	0.36	15.13	unclass. *Gammaproteobacteria*	unclass. *Gammaproteobacteria*	unclass. *Gammaproteobacteria*	*Gammaproteobacteria*	*Proteobacteria*	*Bacteria*

OTU	Av.A_i (*pH in situ*)	Av.A_i (pH 7.67)	Av.δ_i	Av.δ_i/SD	Av.δ_i %	∑Av.δ_i %	Genus	Family	Order	Class	Phylum	Domain
Otu1350	0.4	1.18	0.22	1.1	0.36	15.48	unclass. *Bacteria*	unclass. *Bacteria*	unclass. *Bacteria*	unclass. *Bacteria*	unclass. *Bacteria*	*Bacteria*
Otu0134	0	1.17	0.22	1.43	0.35	15.84	unclass. *Gammaproteobacteria*	unclass. *Gammaproteobacteria*	unclass. *Gammaproteobacteria*	*Gammaproteobacteria*	*Proteobacteria*	*Bacteria*
Otu0156	0.2	1.29	0.22	1.37	0.35	16.19	unclass. *Gammaproteobacteria*	unclass. *Gammaproteobacteria*	unclass. *Gammaproteobacteria*	*Gammaproteobacteria*	*Proteobacteria*	*Bacteria*
Otu0895	0	1.18	0.22	1.78	0.35	16.54	unclass. *Actinobacteria*	unclass. *Actinobacteria*	unclass. *Actinobacteria*	*Actinobacteria*	*Actinobacteria*	*Bacteria*
Otu0053	3.86	2.81	0.21	1.34	0.34	16.88	unclass. *Rhodobacteraceae*	*Rhodobacteraceae*	*Rhodobacterales*	*Alphaproteobacteria*	*Proteobacteria*	*Bacteria*
Otu0849	0.35	1.21	0.21	1.51	0.33	17.22	unclass. *Alphaproteobacteria*	unclass. *Alphaproteobacteria*	unclass. *Alphaproteobacteria*	*Alphaproteobacteria*	*Proteobacteria*	*Bacteria*
Otu0771	1.48	0.4	0.21	1.56	0.33	17.55	unclass. *Rhodobacteraceae*	*Rhodobacteraceae*	*Rhodobacterales*	*Alphaproteobacteria*	*Proteobacteria*	*Bacteria*
Otu0960	1.23	0.2	0.21	1.58	0.33	17.88	unclass. *Bacteria*	unclass. *Bacteria*	unclass. *Bacteria*	unclass. *Bacteria*	unclass. *Bacteria*	*Bacteria*
Otu1100	1.09	0	0.21	1.68	0.33	18.2	unclass. *Planctomycetaceae*	*Planctomycetaceae*	*Planctomycetales*	*Planctomycetacia*	*Planctomycetes*	*Bacteria*
Otu0450	0	1.09	0.2	1.69	0.33	18.53	*Sphingobium*	*Sphingomonadaceae*	*Sphingomonadales*	*Alphaproteobacteria*	*Proteobacteria*	*Bacteria*
Otu1081	1.66	1.68	0.2	1.53	0.32	18.85	unclass. *Flavobacteriales*	unclass. *Flavobacteriales*	*Flavobacteriales*	*Flavobacteria*	*Bacteroidetes*	*Bacteria*
Otu1217	0.85	0.53	0.2	0.89	0.32	19.17	unclass. *Alteromonadales*	unclass. *Alteromonadales*	*Alteromonadales*	*Gammaproteobacteria*	*Proteobacteria*	*Bacteria*
Otu0194	0.4	1.29	0.2	1.27	0.32	19.49	unclass. *Bacteroidetes*	unclass. *Bacteroidetes*	unclass. *Bacteroidetes*	unclass. *Bacteroidetes*	*Bacteroidetes*	*Bacteria*
Otu0139	0	1.04	0.2	1.05	0.31	19.8	unclass. *Gammaproteobacteria*	unclass. *Gammaproteobacteria*	unclass. *Gammaproteobacteria*	*Gammaproteobacteria*	*Proteobacteria*	*Bacteria*
Otu1209	0	1.03	0.19	1.74	0.31	20.11	unclass. *Bacteria*	unclass. *Bacteria*	unclass. *Bacteria*	unclass. *Bacteria*	unclass. *Bacteria*	*Bacteria*
Otu0710	1.24	1.89	0.19	1.31	0.31	20.41	unclass. *Gammaproteobacteria*	unclass. *Gammaproteobacteria*	unclass. *Gammaproteobacteria*	*Gammaproteobacteria*	*Proteobacteria*	*Bacteria*
Otu1125	1.11	1.53	0.19	1.29	0.31	20.72	*Planctomyces*	*Planctomycetaceae*	*Planctomycetales*	*Planctomycetacia*	*Planctomycetes*	*Bacteria*
Otu0840	1.01	0	0.19	0.8	0.3	21.02	unclass. *Flavobacteriaceae*	*Flavobacteriaceae*	*Flavobacteriales*	*Flavobacteria*	*Bacteroidetes*	*Bacteria*
Otu0758	0.69	1.37	0.19	1.82	0.3	21.33	unclass. *Bacteria*	unclass. *Bacteria*	unclass. *Bacteria*	unclass. *Bacteria*	unclass. *Bacteria*	*Bacteria*
Otu0611	0.83	0.49	0.19	1.24	0.3	21.63	unclass. *Bacteroidetes*	unclass. *Bacteroidetes*	unclass. *Bacteroidetes*	unclass. *Bacteroidetes*	*Bacteroidetes*	*Bacteria*
Otu0898	1.13	1.04	0.19	1.36	0.3	21.92	*Sphingopyxis*	*Sphingomonadaceae*	*Sphingomonadales*	*Alphaproteobacteria*	*Proteobacteria*	*Bacteria*
Otu1219	0.88	0.6	0.19	1.35	0.29	22.22	unclass. *Bacteria*	unclass. *Bacteria*	unclass. *Bacteria*	unclass. *Bacteria*	unclass. *Bacteria*	*Bacteria*
Otu0621	1.15	0.6	0.18	1.39	0.29	22.51	*Colwellia*	*Colwelliaceae*	*Alteromonadales*	*Gammaproteobacteria*	*Proteobacteria*	*Bacteria*
Otu0947	0.8	0.28	0.18	0.64	0.29	22.8	unclass. *Bacteroidetes*	unclass. *Bacteroidetes*	unclass. *Bacteroidetes*	unclass. *Bacteroidetes*	*Bacteroidetes*	*Bacteria*
Otu0714	3.04	2.12	0.18	1.81	0.28	23.08	unclass. *Alphaproteobacteria*	unclass. *Alphaproteobacteria*	unclass. *Alphaproteobacteria*	*Alphaproteobacteria*	*Proteobacteria*	*Bacteria*
Otu0517	0.2	1.05	0.18	1.6	0.28	23.36	unclass. *Bacteroidetes*	unclass. *Bacteroidetes*	unclass. *Bacteroidetes*	unclass. *Bacteroidetes*	*Bacteroidetes*	*Bacteria*
Otu1115	1.17	0.4	0.17	1.4	0.28	23.63	*Marinobacter*	*Alteromonadaceae*	*Alteromonadales*	*Gammaproteobacteria*	*Proteobacteria*	*Bacteria*

OTU	Av.A_i (*pH in situ*)	Av.A_i (pH 7.67)	Av.δ_i	Av.δ_i/SD	Av.δ_i %	∑Av.δ_i %	Genus	Family	Order	Class	Phylum	Domain
Otu0959	0.88	0.4	0.17	1.84	0.28	23.91	unclass. *Rhodobacteraceae*	*Rhodobacteraceae*	*Rhodobacterales*	*Alphaproteobacteria*	*Proteobacteria*	*Bacteria*
Otu0778	1.17	0.4	0.17	1.43	0.27	24.19	unclass. *Gammaproteobacteria*	unclass. *Gammaproteobacteria*	unclass. *Gammaproteobacteria*	*Gammaproteobacteria*	*Proteobacteria*	*Bacteria*
Otu1099	0.77	1.29	0.17	1.11	0.27	24.46	unclass. *Lentisphaeria*	unclass. *Lentisphaeria*	unclass. *Lentisphaeria*	*Lentisphaeria*	*Lentisphaerae*	*Bacteria*
Otu1093	3.01	2.75	0.17	1.37	0.27	24.73	unclass. *Proteobacteria*	unclass. *Proteobacteria*	unclass. *Proteobacteria*	unclass. *Proteobacteria*	*Proteobacteria*	*Bacteria*
Otu1430	0	0.88	0.17	1.84	0.27	24.99	unclass. *Bacteria*	unclass. *Bacteria*	unclass. *Bacteria*	unclass. *Bacteria*	unclass. *Bacteria*	*Bacteria*
Otu1270	0	0.88	0.17	1.84	0.27	25.26	unclass. *Flavobacteriaceae*	*Flavobacteriaceae*	*Flavobacteriales*	*Flavobacteria*	*Bacteroidetes*	*Bacteria*
Otu0695	1.29	0.4	0.17	1.47	0.26	25.52	unclass. *Proteobacteria*	unclass. *Proteobacteria*	unclass. *Proteobacteria*	unclass. *Proteobacteria*	*Proteobacteria*	*Bacteria*
Otu1147	0.4	1.11	0.17	1.42	0.26	25.78	unclass. *Bacteria*	unclass. *Bacteria*	unclass. *Bacteria*	unclass. *Bacteria*	unclass. *Bacteria*	*Bacteria*
Otu0068	3.94	4.27	0.16	1.3	0.26	26.05	*Pelagibacter*	SAR11-clade	*Rickettsiales*	*Alphaproteobacteria*	*Proteobacteria*	*Bacteria*
Otu1173	0.2	0.89	0.16	1.15	0.26	26.3	*Opitutus*	*Opitutaceae*	*Opitutales*	*Opitutae*	*Verrucomicrobia*	*Bacteria*
Otu0844	0.85	1.08	0.16	1.28	0.26	26.56	unclass. *Flavobacteriaceae*	*Flavobacteriaceae*	*Flavobacteriales*	*Flavobacteria*	*Bacteroidetes*	*Bacteria*
Otu0971	0.2	0.97	0.16	1.5	0.26	26.81	unclass. *Gammaproteobacteria*	unclass. *Gammaproteobacteria*	unclass. *Gammaproteobacteria*	*Gammaproteobacteria*	*Proteobacteria*	*Bacteria*
Otu0048	1.71	0.91	0.16	1.08	0.26	27.07	unclass. *Flavobacteriaceae*	*Flavobacteriaceae*	*Flavobacteriales*	*Flavobacteria*	*Bacteroidetes*	*Bacteria*
Otu0521	1.49	1.43	0.16	1.3	0.25	27.32	unclass. *Rhodobacteraceae*	*Rhodobacteraceae*	*Rhodobacterales*	*Alphaproteobacteria*	*Proteobacteria*	*Bacteria*
Otu0696	1.48	1.11	0.16	1.3	0.25	27.58	unclass. *Flavobacteriaceae*	*Flavobacteriaceae*	*Flavobacteriales*	*Flavobacteria*	*Bacteroidetes*	*Bacteria*
Otu1078	0.2	0.72	0.16	0.6	0.25	27.83	unclass. *Campylobacterales*	unclass. *Campylobacterales*	*Campylobacterales*	*Epsilonproteobacteria*	*Proteobacteria*	*Bacteria*
Otu0281	1.52	2.15	0.16	1.85	0.25	28.08	unclass. *Flavobacteriales*	unclass. *Flavobacteriales*	*Flavobacteriales*	*Flavobacteria*	*Bacteroidetes*	*Bacteria*
Otu0473	1.92	1.11	0.16	1.28	0.25	28.33	unclass. *Alphaproteobacteria*	unclass. *Alphaproteobacteria*	unclass. *Alphaproteobacteria*	*Alphaproteobacteria*	*Proteobacteria*	*Bacteria*
Otu1091	3.7	2.89	0.16	1.57	0.25	28.58	unclass. *Flavobacteriaceae*	*Flavobacteriaceae*	*Flavobacteriales*	*Flavobacteria*	*Bacteroidetes*	*Bacteria*
Otu0645	0.97	1.52	0.16	1.23	0.25	28.83	*Arcobacter*	*Campylobacteraceae*	*Campylobacterales*	*Epsilonproteobacteria*	*Proteobacteria*	*Bacteria*
Otu0692	2.7	2.39	0.15	1.27	0.25	29.08	unclass. *Bacteria*	unclass. *Bacteria*	unclass. *Bacteria*	unclass. *Bacteria*	unclass. *Bacteria*	*Bacteria*
Otu0485	1.26	0.83	0.15	1.18	0.24	29.32	unclass. *Alphaproteobacteria*	unclass. *Alphaproteobacteria*	unclass. *Alphaproteobacteria*	*Alphaproteobacteria*	*Proteobacteria*	*Bacteria*
Otu0503	0.68	1.15	0.15	1.25	0.24	29.57	unclass. *Bacteroidetes*	unclass. *Bacteroidetes*	unclass. *Bacteroidetes*	unclass. *Bacteroidetes*	*Bacteroidetes*	*Bacteria*
Otu1131	0.4	0.57	0.15	0.81	0.24	29.81	unclass. *Bacteria*	unclass. *Bacteria*	unclass. *Bacteria*	unclass. *Bacteria*	unclass. *Bacteria*	*Bacteria*
Otu0826	2.8	2.19	0.15	1.37	0.24	30.05	unclass. *Bacteroidetes*	unclass. *Bacteroidetes*	unclass. *Bacteroidetes*	unclass. *Bacteroidetes*	*Bacteroidetes*	*Bacteria*
Otu0682	0.89	1.35	0.15	1.35	0.24	30.28	unclass. *Oceanospirillaceae*	*Oceanospirillaceae*	*Oceanospirillales*	*Gammaproteobacteria*	*Proteobacteria*	*Bacteria*
Otu1486	0	0.79	0.15	0.79	0.24	30.52	unclass. *Flavobacteriaceae*	*Flavobacteriaceae*	*Flavobacteriales*	*Flavobacteria*	*Bacteroidetes*	*Bacteria*

OTU	Av.A_i (*pH in situ*)	Av.A_i (pH 7.67)	Av.δ_i	Av.δ_i/SD	Av.δ_i %	∑Av.δ_i %	Genus	Family	Order	Class	Phylum	Domain
Otu1333	0.4	1.03	0.15	1.3	0.24	30.76	unclass. *Legionellaceae*	*Legionellaceae*	*Legionellales*	*Gammaproteobacteria*	*Proteobacteria*	*Bacteria*
Otu1193	0.48	1.05	0.15	1.29	0.24	31	unclass. *Saprospiraceae*	*Saprospiraceae*	*Sphingobacteriales*	*Sphingobacteria*	*Bacteroidetes*	*Bacteria*
Otu0243	0.77	0.63	0.15	1.17	0.24	31.23	unclass. *Gammaproteobacteria*	unclass. *Gammaproteobacteria*	unclass. *Gammaproteobacteria*	*Gammaproteobacteria*	*Proteobacteria*	*Bacteria*
Otu0075	0.63	0.77	0.15	1.18	0.23	31.46	unclass. *Alphaproteobacteria*	unclass. *Alphaproteobacteria*	unclass. *Alphaproteobacteria*	*Alphaproteobacteria*	*Proteobacteria*	*Bacteria*
Otu0191	0.68	0.4	0.15	1.09	0.23	31.7	unclass. *Proteobacteria*	unclass. *Proteobacteria*	unclass. *Proteobacteria*	unclass. *Proteobacteria*	*Proteobacteria*	*Bacteria*
Otu1111	0.88	0.2	0.15	1.45	0.23	31.93	unclass. *Proteobacteria*	unclass. *Proteobacteria*	unclass. *Proteobacteria*	unclass. *Proteobacteria*	*Proteobacteria*	*Bacteria*
Otu0596	1.79	1.17	0.14	1.09	0.23	32.16	*Neptunomonas*	*Oceanospirillaceae*	*Oceanospirillales*	*Gammaproteobacteria*	*Proteobacteria*	*Bacteria*
Otu1186	0.68	0.63	0.14	1.24	0.23	32.39	unclass. *Bacteria*	unclass. *Bacteria*	unclass. *Bacteria*	unclass. *Bacteria*	unclass. *Bacteria*	*Bacteria*
Otu1118	0.77	0	0.14	1.18	0.23	32.62	unclass. *Oceanospirillales*	unclass. *Oceanospirillales*	*Oceanospirillales*	*Gammaproteobacteria*	*Proteobacteria*	*Bacteria*
Otu1198	0.2	0.88	0.14	1.46	0.23	32.85	unclass. *Gammaproteobacteria*	unclass. *Gammaproteobacteria*	unclass. *Gammaproteobacteria*	*Gammaproteobacteria*	*Proteobacteria*	*Bacteria*
Otu1107	0.68	1.35	0.14	1.22	0.23	33.07	unclass. *Planctomycetaceae*	*Planctomycetaceae*	*Planctomycetales*	*Planctomycetacia*	*Planctomycetes*	*Bacteria*
Otu1234	0.4	0.83	0.14	1.18	0.23	33.3	unclass. *Oceanospirillaceae*	*Oceanospirillaceae*	*Oceanospirillales*	*Gammaproteobacteria*	*Proteobacteria*	*Bacteria*
Otu0186	0.85	1.17	0.14	1.11	0.23	33.52	unclass. *Planctomycetaceae*	*Planctomycetaceae*	*Planctomycetales*	*Planctomycetacia*	*Planctomycetes*	*Bacteria*
Otu0464	0.4	0.83	0.14	1.19	0.22	33.75	unclass. *Bacteroidetes*	unclass. *Bacteroidetes*	unclass. *Bacteroidetes*	unclass. *Bacteroidetes*	*Bacteroidetes*	*Bacteria*
Otu0703	0.63	0.6	0.14	1.38	0.22	33.97	unclass. *Gammaproteobacteria*	unclass. *Gammaproteobacteria*	unclass. *Gammaproteobacteria*	*Gammaproteobacteria*	*Proteobacteria*	*Bacteria*
Otu0986	0.75	0.77	0.14	1.22	0.22	34.19	unclass. *Planctomycetaceae*	*Planctomycetaceae*	*Planctomycetales*	*Planctomycetacia*	*Planctomycetes*	*Bacteria*
Otu0809	0.49	0.4	0.14	0.87	0.22	34.41	unclass. *Gammaproteobacteria*	unclass. *Gammaproteobacteria*	unclass. *Gammaproteobacteria*	*Gammaproteobacteria*	*Proteobacteria*	*Bacteria*
Otu0502	0.83	0.95	0.14	1.27	0.22	34.63	unclass. *Gammaproteobacteria*	unclass. *Gammaproteobacteria*	unclass. *Gammaproteobacteria*	*Gammaproteobacteria*	*Proteobacteria*	*Bacteria*
Otu0846	0.83	0.68	0.14	1.22	0.22	34.84	unclass. *Nannocystaceae*	*Nannocystaceae*	*Myxococcales*	*Deltaproteobacteria*	*Proteobacteria*	*Bacteria*
Otu0255	0.97	1.23	0.14	1.22	0.22	35.06	*Sphingopyxis*	*Sphingomonadaceae*	*Sphingomonadales*	*Alphaproteobacteria*	*Proteobacteria*	*Bacteria*
Otu0707	0.55	0.8	0.13	1.44	0.21	35.27	*Haliea*	*Alteromonadaceae*	*Alteromonadales*	*Gammaproteobacteria*	*Proteobacteria*	*Bacteria*
Otu0734	0.28	0.68	0.13	1.17	0.21	35.48	unclass. *Bacteria*	unclass. *Bacteria*	unclass. *Bacteria*	unclass. *Bacteria*	unclass. *Bacteria*	*Bacteria*
Otu0202	0.88	0.48	0.13	1.29	0.21	35.69	unclass. *Alteromonadaceae*	*Alteromonadaceae*	*Alteromonadales*	*Gammaproteobacteria*	*Proteobacteria*	*Bacteria*
Otu0284	0.68	0	0.13	1.17	0.21	35.9	unclass. *Saprospiraceae*	*Saprospiraceae*	*Sphingobacteriales*	*Sphingobacteria*	*Bacteroidetes*	*Bacteria*
Otu0761	0.48	0.88	0.13	1.32	0.21	36.11	unclass. *Verrucomicrobiaceae*	*Verrucomicrobiaceae*	*Verrucomicrobiales*	*Verrucomicrobiae*	*Verrucomicrobia*	*Bacteria*
Otu1256	0	0.68	0.13	1.15	0.2	36.31	unclass. *Sphingobacteriales*	unclass. *Sphingobacteriales*	*Sphingobacteriales*	*Sphingobacteria*	*Bacteroidetes*	*Bacteria*
Otu0824	0	0.68	0.13	1.15	0.2	36.51	unclass. *Bacteria*	unclass. *Bacteria*	unclass. *Bacteria*	unclass. *Bacteria*	unclass. *Bacteria*	*Bacteria*

OTU	Av.A_i (*pH in situ*)	Av.A_i (pH 7.67)	Av.δ_i	Av.δ_i/SD	Av.δ_i %	∑Av.δ_i %	Genus	Family	Order	Class	Phylum	Domain
Otu1432	0	0.68	0.13	1.16	0.2	36.72	unclass. *Oceanospirillales*	unclass. *Oceanospirillales*	*Oceanospirillales*	*Gammaproteobacteria*	*Proteobacteria*	*Bacteria*
Otu1130	0.4	0.6	0.13	0.98	0.2	36.92	unclass. *Bacteria*	unclass. *Bacteria*	unclass. *Bacteria*	unclass. *Bacteria*	unclass. *Bacteria*	*Bacteria*
Otu0742	0.77	0.4	0.13	1.2	0.2	37.13	unclass. *Rhodobacteraceae*	*Rhodobacteraceae*	*Rhodobacterales*	*Alphaproteobacteria*	*Proteobacteria*	*Bacteria*
Otu0394	0.2	0.8	0.13	1.42	0.2	37.33	unclass. *Bacteria*	unclass. *Bacteria*	unclass. *Bacteria*	unclass. *Bacteria*	unclass. *Bacteria*	*Bacteria*
Otu0483	0.68	0	0.13	1.14	0.2	37.53	unclass. *Flavobacteriaceae*	*Flavobacteriaceae*	*Flavobacteriales*	*Flavobacteria*	*Bacteroidetes*	*Bacteria*
Otu1101	0.68	0.75	0.13	1.17	0.2	37.73	*Porphyrobacter*	*Erythrobacteraceae*	*Sphingomonadales*	*Alphaproteobacteria*	*Proteobacteria*	*Bacteria*
Otu1338	0.2	0.63	0.13	0.93	0.2	37.94	unclass. *Bacteroidetes*	unclass. *Bacteroidetes*	unclass. *Bacteroidetes*	unclass. *Bacteroidetes*	*Bacteroidetes*	*Bacteria*
Otu1281	0.63	0	0.13	0.49	0.2	38.14	unclass. *Proteobacteria*	unclass. *Proteobacteria*	unclass. *Proteobacteria*	unclass. *Proteobacteria*	*Proteobacteria*	*Bacteria*
Otu0375	0.75	0.4	0.13	1.09	0.2	38.34	unclass. *Bacteroidetes*	unclass. *Bacteroidetes*	unclass. *Bacteroidetes*	unclass. *Bacteroidetes*	*Bacteroidetes*	*Bacteria*
Otu0373	0.2	0.63	0.13	0.92	0.2	38.54	unclass. *Bacteria*	unclass. *Bacteria*	unclass. *Bacteria*	unclass. *Bacteria*	unclass. *Bacteria*	*Bacteria*
Otu0052	2.7	2.32	0.13	1.36	0.2	38.74	unclass. *Betaproteobacteria*	unclass. *Betaproteobacteria*	unclass. *Betaproteobacteria*	*Betaproteobacteria*	*Proteobacteria*	*Bacteria*
Otu0998	0.4	0.75	0.13	1.09	0.2	38.94	unclass. *Gammaproteobacteria*	unclass. *Gammaproteobacteria*	unclass. *Gammaproteobacteria*	*Gammaproteobacteria*	*Proteobacteria*	*Bacteria*
Otu1110	0.68	0.48	0.12	1.14	0.2	39.14	unclass. *Alphaproteobacteria*	unclass. *Alphaproteobacteria*	unclass. *Alphaproteobacteria*	*Alphaproteobacteria*	*Proteobacteria*	*Bacteria*
Otu0469	2.44	2.49	0.12	1.37	0.2	39.34	unclass. *Bacteria*	unclass. *Bacteria*	unclass. *Bacteria*	unclass. *Bacteria*	unclass. *Bacteria*	*Bacteria*
Otu0219	0.4	0.57	0.12	1.11	0.19	39.53	unclass. *Erythrobacteraceae*	*Erythrobacteraceae*	*Sphingomonadales*	*Alphaproteobacteria*	*Proteobacteria*	*Bacteria*
Otu1417	0	0.65	0.12	0.71	0.19	39.73	unclass. *Gammaproteobacteria*	unclass. *Gammaproteobacteria*	unclass. *Gammaproteobacteria*	*Gammaproteobacteria*	*Proteobacteria*	*Bacteria*
Otu0142	0.28	0.6	0.12	1.27	0.19	39.92	unclass. *Gammaproteobacteria*	unclass. *Gammaproteobacteria*	unclass. *Gammaproteobacteria*	*Gammaproteobacteria*	*Proteobacteria*	*Bacteria*
Otu1135	0.2	0.68	0.12	1.12	0.19	40.11	unclass. *Sphingobacteriales*	unclass. *Sphingobacteriales*	*Sphingobacteriales*	*Sphingobacteria*	*Bacteroidetes*	*Bacteria*
Otu0395	0.2	0.68	0.12	1.12	0.19	40.31	unclass. *Sphingobacteriales*	unclass. *Sphingobacteriales*	*Sphingobacteriales*	*Sphingobacteria*	*Bacteroidetes*	*Bacteria*
Otu1109	0.77	0.6	0.12	1.24	0.19	40.5	unclass. *Saprospiraceae*	*Saprospiraceae*	*Sphingobacteriales*	*Sphingobacteria*	*Bacteroidetes*	*Bacteria*
Otu1195	0.2	0.68	0.12	1.12	0.19	40.69	unclass. *Bacteria*	unclass. *Bacteria*	unclass. *Bacteria*	unclass. *Bacteria*	unclass. *Bacteria*	*Bacteria*
Otu1076	0.28	0.55	0.12	0.9	0.19	40.88	*Acinetobacter*	*Moraxellaceae*	*Pseudomonadales*	*Gammaproteobacteria*	*Proteobacteria*	*Bacteria*
Otu0465	0	0.63	0.12	0.79	0.19	41.07	unclass. *Bacteroidetes*	unclass. *Bacteroidetes*	unclass. *Bacteroidetes*	unclass. *Bacteroidetes*	*Bacteroidetes*	*Bacteria*
Otu1119	0.68	0.2	0.12	1.13	0.19	41.26	unclass. *Bacteroidetes*	unclass. *Bacteroidetes*	unclass. *Bacteroidetes*	unclass. *Bacteroidetes*	*Bacteroidetes*	*Bacteria*
Otu1030	0.35	0.48	0.12	0.92	0.19	41.45	unclass. *Proteobacteria*	unclass. *Proteobacteria*	unclass. *Proteobacteria*	unclass. *Proteobacteria*	*Proteobacteria*	*Bacteria*
Otu0541	1.6	1.03	0.12	1.12	0.19	41.64	unclass. *Rhodobacteraceae*	*Rhodobacteraceae*	*Rhodobacterales*	*Alphaproteobacteria*	*Proteobacteria*	*Bacteria*
Otu0812	0.48	0.48	0.12	1.01	0.18	41.82	unclass. *Gammaproteobacteria*	unclass. *Gammaproteobacteria*	unclass. *Gammaproteobacteria*	*Gammaproteobacteria*	*Proteobacteria*	*Bacteria*

OTU	Av.A_i (*pH in situ*)	Av.A_i (pH 7.67)	Av.δ_i	Av.δ_i/SD	Av.δ_i %	∑Av.δ_i %	Genus	Family	Order	Class	Phylum	Domain
Otu0882	0	0.6	0.11	1.2	0.18	42	unclass. *Bacteria*	unclass. *Bacteria*	unclass. *Bacteria*	unclass. *Bacteria*	unclass. *Bacteria*	*Bacteria*
Otu1095	0.6	0	0.11	0.74	0.18	42.18	unclass. *Bacteria*	unclass. *Bacteria*	unclass. *Bacteria*	unclass. *Bacteria*	unclass. *Bacteria*	*Bacteria*
Otu0677	0.6	0	0.11	0.73	0.18	42.36	*Thalassobius*	*Rhodobacteraceae*	*Rhodobacterales*	*Alphaproteobacteria*	*Proteobacteria*	*Bacteria*
Otu1133	0.6	0	0.11	1.2	0.18	42.53	unclass. *Bacteria*	unclass. *Bacteria*	unclass. *Bacteria*	unclass. *Bacteria*	unclass. *Bacteria*	*Bacteria*
Otu1023	2.35	2.9	0.11	1.34	0.18	42.71	unclass. *Lentisphaeria*	unclass. *Lentisphaeria*	unclass. *Lentisphaeria*	*Lentisphaeria*	*Lentisphaerae*	*Bacteria*
Otu0731	0.45	0.2	0.11	0.65	0.18	42.89	*Lentisphaera*	*Lentisphaeraceae*	*Lentisphaerales*	*Lentisphaeria*	*Lentisphaerae*	*Bacteria*
Otu0509	1.39	1.08	0.11	1.19	0.18	43.06	unclass. *Gammaproteobacteria*	unclass. *Gammaproteobacteria*	unclass. *Gammaproteobacteria*	*Gammaproteobacteria*	*Proteobacteria*	*Bacteria*
Otu0694	1.41	1.19	0.11	1.06	0.17	43.24	unclass. *Alphaproteobacteria*	unclass. *Alphaproteobacteria*	unclass. *Alphaproteobacteria*	*Alphaproteobacteria*	*Proteobacteria*	*Bacteria*
Otu1113	1.11	1.13	0.11	0.9	0.17	43.41	unclass. *Gammaproteobacteria*	unclass. *Gammaproteobacteria*	unclass. *Gammaproteobacteria*	*Gammaproteobacteria*	*Proteobacteria*	*Bacteria*
Otu1211	0.28	0.48	0.11	0.89	0.17	43.58	unclass. *Oceanospirillaceae*	*Oceanospirillaceae*	*Oceanospirillales*	*Gammaproteobacteria*	*Proteobacteria*	*Bacteria*
Otu0044	0.6	0.2	0.11	1.1	0.17	43.75	unclass. *Gammaproteobacteria*	unclass. *Gammaproteobacteria*	unclass. *Gammaproteobacteria*	*Gammaproteobacteria*	*Proteobacteria*	*Bacteria*
Otu1552	0	0.57	0.11	0.8	0.17	43.92	unclass. *Gammaproteobacteria*	unclass. *Gammaproteobacteria*	unclass. *Gammaproteobacteria*	*Gammaproteobacteria*	*Proteobacteria*	*Bacteria*
Otu0973	0.4	0.8	0.11	1.1	0.17	44.09	unclass. *Flavobacteriaceae*	*Flavobacteriaceae*	*Flavobacteriales*	*Flavobacteria*	*Bacteroidetes*	*Bacteria*
Otu0504	0.6	0.2	0.11	1.1	0.17	44.26	unclass. *Alphaproteobacteria*	unclass. *Alphaproteobacteria*	unclass. *Alphaproteobacteria*	*Alphaproteobacteria*	*Proteobacteria*	*Bacteria*
Otu0767	0.2	0.6	0.11	1.1	0.17	44.43	unclass. *Bacteria*	unclass. *Bacteria*	unclass. *Bacteria*	unclass. *Bacteria*	unclass. *Bacteria*	*Bacteria*
Otu0934	0.4	0.48	0.11	1	0.17	44.6	unclass. *Bacteria*	unclass. *Bacteria*	unclass. *Bacteria*	unclass. *Bacteria*	unclass. *Bacteria*	*Bacteria*
Otu0062	0.55	0	0.11	0.74	0.17	44.77	*Haliea*	*Alteromonadaceae*	*Alteromonadales*	*Gammaproteobacteria*	*Proteobacteria*	*Bacteria*
Otu0817	0.8	0.4	0.11	1.1	0.17	44.93	unclass. *Bacteroidetes*	unclass. *Bacteroidetes*	unclass. *Bacteroidetes*	unclass. *Bacteroidetes*	*Bacteroidetes*	*Bacteria*
Otu0170	0.8	0.4	0.11	1.1	0.17	45.1	unclass. *Bacteria*	unclass. *Bacteria*	unclass. *Bacteria*	unclass. *Bacteria*	unclass. *Bacteria*	*Bacteria*
Otu1485	0	0.57	0.1	0.49	0.17	45.27	unclass. *Gammaproteobacteria*	unclass. *Gammaproteobacteria*	unclass. *Gammaproteobacteria*	*Gammaproteobacteria*	*Proteobacteria*	*Bacteria*
Otu0974	0.57	0	0.1	0.8	0.17	45.44	unclass. *Flavobacteriaceae*	*Flavobacteriaceae*	*Flavobacteriales*	*Flavobacteria*	*Bacteroidetes*	*Bacteria*
Otu1421	0	0.55	0.1	0.76	0.17	45.6	unclass. *Gammaproteobacteria*	unclass. *Gammaproteobacteria*	unclass. *Gammaproteobacteria*	*Gammaproteobacteria*	*Proteobacteria*	*Bacteria*
Otu1207	0	0.55	0.1	0.76	0.16	45.77	unclass. *Bacteroidetes*	unclass. *Bacteroidetes*	unclass. *Bacteroidetes*	unclass. *Bacteroidetes*	*Bacteroidetes*	*Bacteria*
Otu1550	0	0.55	0.1	0.76	0.16	45.93	unclass. *Gammaproteobacteria*	unclass. *Gammaproteobacteria*	unclass. *Gammaproteobacteria*	*Gammaproteobacteria*	*Proteobacteria*	*Bacteria*
Otu0379	0.55	0	0.1	0.77	0.16	46.09	unclass. *Proteobacteria*	unclass. *Proteobacteria*	unclass. *Proteobacteria*	unclass. *Proteobacteria*	*Proteobacteria*	*Bacteria*
Otu0428	0	0.55	0.1	0.75	0.16	46.26	unclass. *Gammaproteobacteria*	unclass. *Gammaproteobacteria*	unclass. *Gammaproteobacteria*	*Gammaproteobacteria*	*Proteobacteria*	*Bacteria*
Otu1197	0.4	0.2	0.1	0.67	0.16	46.42	unclass. *Gammaproteobacteria*	unclass. *Gammaproteobacteria*	unclass. *Gammaproteobacteria*	*Gammaproteobacteria*	*Proteobacteria*	*Bacteria*

OTU	Av.A_i (*pH in situ*)	Av.A_i (pH 7.67)	Av.δ_i	Av.δ_i/SD	Av.δ_i %	∑Av.δ_i %	Genus	Family	Order	Class	Phylum	Domain
Otu1096	1.18	1.52	0.1	0.97	0.16	46.58	unclass. *Lentisphaeria*	unclass. *Lentisphaeria*	unclass. *Lentisphaeria*	*Lentisphaeria*	*Lentisphaerae*	*Bacteria*
Otu1296	0.4	0.28	0.1	0.94	0.16	46.74	unclass. *Proteobacteria*	unclass. *Proteobacteria*	unclass. *Proteobacteria*	unclass. *Proteobacteria*	*Proteobacteria*	*Bacteria*
Otu0737	1.08	1.58	0.1	1.52	0.16	46.9	unclass. *Gammaproteobacteria*	unclass. *Gammaproteobacteria*	unclass. *Gammaproteobacteria*	*Gammaproteobacteria*	*Proteobacteria*	*Bacteria*
Otu1222	0.2	0.48	0.1	0.88	0.16	47.06	unclass. *Flavobacteriaceae*	*Flavobacteriaceae*	*Flavobacteriales*	*Flavobacteria*	*Bacteroidetes*	*Bacteria*
Otu0035	0.28	0.4	0.1	0.94	0.16	47.21	*Colwellia*	*Colwelliaceae*	*Alteromonadales*	*Gammaproteobacteria*	*Proteobacteria*	*Bacteria*
Otu0622	0.2	0.48	0.1	0.88	0.16	47.37	*Colwellia*	*Colwelliaceae*	*Alteromonadales*	*Gammaproteobacteria*	*Proteobacteria*	*Bacteria*
Otu1156	0.2	0.4	0.1	0.67	0.16	47.53	unclass. *Gammaproteobacteria*	unclass. *Gammaproteobacteria*	unclass. *Gammaproteobacteria*	*Gammaproteobacteria*	*Proteobacteria*	*Bacteria*
Otu1232	0.2	0.48	0.1	0.89	0.16	47.68	*Haliea*	*Alteromonadaceae*	*Alteromonadales*	*Gammaproteobacteria*	*Proteobacteria*	*Bacteria*
Otu0046	0.48	0.2	0.1	0.89	0.16	47.84	unclass. *Flavobacteriaceae*	*Flavobacteriaceae*	*Flavobacteriales*	*Flavobacteria*	*Bacteroidetes*	*Bacteria*
Otu1188	0.2	0.48	0.1	0.89	0.16	47.99	unclass. *Flammeovirgaceae*	*Flammeovirgaceae*	*Sphingobacteriales*	*Sphingobacteria*	*Bacteroidetes*	*Bacteria*
Otu0030	0.28	0.4	0.1	0.94	0.16	48.15	unclass. *Gammaproteobacteria*	unclass. *Gammaproteobacteria*	unclass. *Gammaproteobacteria*	*Gammaproteobacteria*	*Proteobacteria*	*Bacteria*
Otu0213	0.48	0.2	0.1	0.89	0.16	48.3	unclass. *Gammaproteobacteria*	unclass. *Gammaproteobacteria*	unclass. *Gammaproteobacteria*	*Gammaproteobacteria*	*Proteobacteria*	*Bacteria*
Otu0744	0.48	0.2	0.1	0.89	0.16	48.46	unclass. *Gammaproteobacteria*	unclass. *Gammaproteobacteria*	unclass. *Gammaproteobacteria*	*Gammaproteobacteria*	*Proteobacteria*	*Bacteria*
Otu0333	0.6	0.4	0.1	1.02	0.16	48.61	unclass. *Gammaproteobacteria*	unclass. *Gammaproteobacteria*	unclass. *Gammaproteobacteria*	*Gammaproteobacteria*	*Proteobacteria*	*Bacteria*
Otu0510	0.48	0.2	0.1	0.89	0.15	48.77	unclass. *Gammaproteobacteria*	unclass. *Gammaproteobacteria*	unclass. *Gammaproteobacteria*	*Gammaproteobacteria*	*Proteobacteria*	*Bacteria*
Otu0421	0.48	0.2	0.1	0.88	0.15	48.92	unclass. *Gammaproteobacteria*	unclass. *Gammaproteobacteria*	unclass. *Gammaproteobacteria*	*Gammaproteobacteria*	*Proteobacteria*	*Bacteria*
Otu1094	2.68	3.09	0.1	1.22	0.15	49.08	unclass. *Bacteroidetes*	unclass. *Bacteroidetes*	unclass. *Bacteroidetes*	unclass. *Bacteroidetes*	*Bacteroidetes*	*Bacteria*
Otu0143	0.48	0	0.1	0.78	0.15	49.23	unclass. *Gammaproteobacteria*	unclass. *Gammaproteobacteria*	unclass. *Gammaproteobacteria*	*Gammaproteobacteria*	*Proteobacteria*	*Bacteria*
Otu0192	0.48	0	0.1	0.78	0.15	49.38	unclass. *Gammaproteobacteria*	unclass. *Gammaproteobacteria*	unclass. *Gammaproteobacteria*	*Gammaproteobacteria*	*Proteobacteria*	*Bacteria*
Otu1114	0.48	0	0.09	0.78	0.15	49.53	unclass. *Microbacteriaceae*	*Microbacteriaceae*	*Actinomycetales*	*Actinobacteria*	*Actinobacteria*	*Bacteria*
Otu0084	1.23	1.52	0.09	1.04	0.15	49.68	unclass. *Gammaproteobacteria*	unclass. *Gammaproteobacteria*	unclass. *Gammaproteobacteria*	*Gammaproteobacteria*	*Proteobacteria*	*Bacteria*
Otu0520	0	0.48	0.09	0.78	0.15	49.83	unclass. *Alphaproteobacteria*	unclass. *Alphaproteobacteria*	unclass. *Alphaproteobacteria*	*Alphaproteobacteria*	*Proteobacteria*	*Bacteria*
Otu1433	0	0.48	0.09	0.78	0.15	49.97	unclass. *Bacteria*	unclass. *Bacteria*	unclass. *Bacteria*	unclass. *Bacteria*	unclass. *Bacteria*	*Bacteria*
Otu1470	0	0.48	0.09	0.78	0.15	50.12	unclass. *Gammaproteobacteria*	unclass. *Gammaproteobacteria*	unclass. *Gammaproteobacteria*	*Gammaproteobacteria*	*Proteobacteria*	*Bacteria*
Otu1496	0	0.48	0.09	0.78	0.14	50.26	unclass. *Bacteria*	unclass. *Bacteria*	unclass. *Bacteria*	unclass. *Bacteria*	unclass. *Bacteria*	*Bacteria*
Otu1378	0	0.48	0.09	0.78	0.14	50.41	unclass. *Gammaproteobacteria*	unclass. *Gammaproteobacteria*	unclass. *Gammaproteobacteria*	*Gammaproteobacteria*	*Proteobacteria*	*Bacteria*
Otu1441	0	0.48	0.09	0.78	0.14	50.55	unclass. *Deltaproteobacteria*	unclass. *Deltaproteobacteria*	unclass. *Deltaproteobacteria*	*Deltaproteobacteria*	*Proteobacteria*	*Bacteria*

OTU	Av.A_i (*pH in situ*)	Av.A_i (pH 7.67)	Av.δ_i	Av.δ_i/SD	Av.δ_i %	∑Av.δ_i %	Genus	Family	Order	Class	Phylum	Domain
Otu1578	0	0.48	0.09	0.78	0.14	50.7	unclass. *Planctomycetaceae*	*Planctomycetaceae*	*Planctomycetales*	*Planctomycetacia*	*Planctomycetes*	*Bacteria*
Otu0057	0	0.48	0.09	0.78	0.14	50.84	*Ulvibacter*	*Flavobacteriaceae*	*Flavobacteriales*	*Flavobacteria*	*Bacteroidetes*	*Bacteria*
Otu0426	0.4	0.4	0.09	0.94	0.14	50.99	unclass. *Gammaproteobacteria*	unclass. *Gammaproteobacteria*	unclass. *Gammaproteobacteria*	*Gammaproteobacteria*	*Proteobacteria*	*Bacteria*
Otu0136	0	0.48	0.09	0.78	0.14	51.13	unclass. *Bacteroidetes*	unclass. *Bacteroidetes*	unclass. *Bacteroidetes*	unclass. *Bacteroidetes*	*Bacteroidetes*	*Bacteria*
Otu0269	0	0.48	0.09	0.78	0.14	51.27	unclass. *Gammaproteobacteria*	unclass. *Gammaproteobacteria*	unclass. *Gammaproteobacteria*	*Gammaproteobacteria*	*Proteobacteria*	*Bacteria*
Otu1106	0.4	0.4	0.09	0.94	0.14	51.42	unclass. *Bacteria*	unclass. *Bacteria*	unclass. *Bacteria*	unclass. *Bacteria*	unclass. *Bacteria*	*Bacteria*
Otu1343	0.48	0	0.09	0.79	0.14	51.56	*Planctomyces*	*Planctomycetaceae*	*Planctomycetales*	*Planctomycetacia*	*Planctomycetes*	*Bacteria*
Otu0833	0.48	0	0.09	0.79	0.14	51.7	*Alcanivorax*	*Alcanivoracaceae*	*Oceanospirillales*	*Gammaproteobacteria*	*Proteobacteria*	*Bacteria*
Otu0008	0.2	0.35	0.09	0.68	0.14	51.84	unclass. *Gammaproteobacteria*	unclass. *Gammaproteobacteria*	unclass. *Gammaproteobacteria*	*Gammaproteobacteria*	*Proteobacteria*	*Bacteria*
Otu0137	0.88	1.23	0.09	0.88	0.14	51.98	unclass. *Bacteroidetes*	unclass. *Bacteroidetes*	unclass. *Bacteroidetes*	unclass. *Bacteroidetes*	*Bacteroidetes*	*Bacteria*
Otu1103	0.2	0.35	0.09	0.68	0.14	52.11	unclass. *Bacteria*	unclass. *Bacteria*	unclass. *Bacteria*	unclass. *Bacteria*	unclass. *Bacteria*	*Bacteria*
Otu0821	0.35	0.2	0.09	0.69	0.14	52.25	unclass. *Bacteria*	unclass. *Bacteria*	unclass. *Bacteria*	unclass. *Bacteria*	unclass. *Bacteria*	*Bacteria*
Otu0915	0.4	0.2	0.08	0.87	0.13	52.38	unclass. *Alphaproteobacteria*	unclass. *Alphaproteobacteria*	unclass. *Alphaproteobacteria*	*Alphaproteobacteria*	*Proteobacteria*	*Bacteria*
Otu0011	0.4	0.2	0.08	0.87	0.13	52.52	unclass. *Flavobacteriaceae*	*Flavobacteriaceae*	*Flavobacteriales*	*Flavobacteria*	*Bacteroidetes*	*Bacteria*
Otu0093	0.2	0.4	0.08	0.87	0.13	52.65	*Aestuariicola*	*Flavobacteriaceae*	*Flavobacteriales*	*Flavobacteria*	*Bacteroidetes*	*Bacteria*
Otu1304	0.2	0.4	0.08	0.87	0.13	52.78	unclass. *Bacteria*	unclass. *Bacteria*	unclass. *Bacteria*	unclass. *Bacteria*	unclass. *Bacteria*	*Bacteria*
Otu0316	0.2	0.4	0.08	0.87	0.13	52.92	unclass. *Bacteria*	unclass. *Bacteria*	unclass. *Bacteria*	unclass. *Bacteria*	unclass. *Bacteria*	*Bacteria*
Otu1334	0.2	0.4	0.08	0.87	0.13	53.05	unclass. *Gammaproteobacteria*	unclass. *Gammaproteobacteria*	unclass. *Gammaproteobacteria*	*Gammaproteobacteria*	*Proteobacteria*	*Bacteria*
Otu1282	0.2	0.4	0.08	0.87	0.13	53.18	unclass. *Gammaproteobacteria*	unclass. *Gammaproteobacteria*	unclass. *Gammaproteobacteria*	*Gammaproteobacteria*	*Proteobacteria*	*Bacteria*
Otu1286	0.2	0.4	0.08	0.87	0.13	53.31	unclass. *Gammaproteobacteria*	unclass. *Gammaproteobacteria*	unclass. *Gammaproteobacteria*	*Gammaproteobacteria*	*Proteobacteria*	*Bacteria*
Otu1346	0.2	0.4	0.08	0.87	0.13	53.45	unclass. *Planctomycetaceae*	*Planctomycetaceae*	*Planctomycetales*	*Planctomycetacia*	*Planctomycetes*	*Bacteria*
Otu0007	0.2	0.4	0.08	0.87	0.13	53.58	unclass. *Flavobacteriales*	unclass. *Flavobacteriales*	*Flavobacteriales*	*Flavobacteria*	*Bacteroidetes*	*Bacteria*
Otu0601	0.8	0.6	0.08	0.87	0.13	53.71	unclass. *Flammeovirgaceae*	*Flammeovirgaceae*	*Sphingobacteriales*	*Sphingobacteria*	*Bacteroidetes*	*Bacteria*
Otu0064	0.2	0.4	0.08	0.87	0.13	53.84	unclass. *Flavobacteriaceae*	*Flavobacteriaceae*	*Flavobacteriales*	*Flavobacteria*	*Bacteroidetes*	*Bacteria*
Otu1396	0.2	0.4	0.08	0.87	0.13	53.97	unclass. *Flavobacteriaceae*	*Flavobacteriaceae*	*Flavobacteriales*	*Flavobacteria*	*Bacteroidetes*	*Bacteria*
Otu0709	0.2	0.4	0.08	0.87	0.13	54.1	unclass. *Bacteria*	unclass. *Bacteria*	unclass. *Bacteria*	unclass. *Bacteria*	unclass. *Bacteria*	*Bacteria*
Otu0203	0.4	0.2	0.08	0.87	0.13	54.23	*Pelagibacter*	SAR11-clade	*Rickettsiales*	*Alphaproteobacteria*	*Proteobacteria*	*Bacteria*

OTU	Av.A_i (*pH in situ*)	Av.A_i (pH 7.67)	Av.δ_i	Av.δ_i/SD	Av.δ_i %	∑Av.δ_i %	Genus	Family	Order	Class	Phylum	Domain
Otu1049	0.4	0.2	0.08	0.87	0.13	54.36	unclass. *Gammaproteobacteria*	unclass. *Gammaproteobacteria*	unclass. *Gammaproteobacteria*	*Gammaproteobacteria*	*Proteobacteria*	*Bacteria*
Otu1199	0.4	0.2	0.08	0.87	0.13	54.49	unclass. *Rhodobacteraceae*	*Rhodobacteraceae*	*Rhodobacterales*	*Alphaproteobacteria*	*Proteobacteria*	*Bacteria*
Otu0738	0.4	0	0.08	0.8	0.13	54.62	unclass. *Alphaproteobacteria*	unclass. *Alphaproteobacteria*	unclass. *Alphaproteobacteria*	*Alphaproteobacteria*	*Proteobacteria*	*Bacteria*
Otu1300	0.4	0	0.08	0.8	0.13	54.74	unclass. *Proteobacteria*	unclass. *Proteobacteria*	unclass. *Proteobacteria*	unclass. *Proteobacteria*	*Proteobacteria*	*Bacteria*
Otu0040	0.28	0.2	0.08	0.69	0.12	54.87	*Erythrobacter*	*Erythrobacteraceae*	*Sphingomonadales*	*Alphaproteobacteria*	*Proteobacteria*	*Bacteria*
Otu1202	0.2	0.28	0.08	0.7	0.12	54.99	unclass. *Gammaproteobacteria*	unclass. *Gammaproteobacteria*	unclass. *Gammaproteobacteria*	*Gammaproteobacteria*	*Proteobacteria*	*Bacteria*
Otu0513	0.28	0.2	0.08	0.69	0.12	55.11	unclass. *Bacteroidetes*	unclass. *Bacteroidetes*	unclass. *Bacteroidetes*	unclass. *Bacteroidetes*	*Bacteroidetes*	*Bacteria*
Otu0699	0.28	0.2	0.08	0.69	0.12	55.24	unclass. *Flavobacteriales*	unclass. *Flavobacteriales*	*Flavobacteriales*	*Flavobacteria*	*Bacteroidetes*	*Bacteria*
Otu0376	0.2	0.28	0.08	0.69	0.12	55.36	unclass. *Gammaproteobacteria*	unclass. *Gammaproteobacteria*	unclass. *Gammaproteobacteria*	*Gammaproteobacteria*	*Proteobacteria*	*Bacteria*
Otu0892	0.2	0.28	0.08	0.69	0.12	55.48	unclass. *Bacteroidetes*	unclass. *Bacteroidetes*	unclass. *Bacteroidetes*	unclass. *Bacteroidetes*	*Bacteroidetes*	*Bacteria*
Otu1317	0.28	0.2	0.08	0.69	0.12	55.6	unclass. *Bacteria*	unclass. *Bacteria*	unclass. *Bacteria*	unclass. *Bacteria*	unclass. *Bacteria*	*Bacteria*
Otu0300	0.4	0	0.08	0.8	0.12	55.72	unclass. *Gammaproteobacteria*	unclass. *Gammaproteobacteria*	unclass. *Gammaproteobacteria*	*Gammaproteobacteria*	*Proteobacteria*	*Bacteria*
Otu0368	0.4	0	0.08	0.8	0.12	55.84	unclass. *Saprospiraceae*	*Saprospiraceae*	*Sphingobacteriales*	*Sphingobacteria*	*Bacteroidetes*	*Bacteria*
Otu1126	0	0.4	0.08	0.8	0.12	55.97	unclass. *Bacteroidetes*	unclass. *Bacteroidetes*	unclass. *Bacteroidetes*	unclass. *Bacteroidetes*	*Bacteroidetes*	*Bacteria*
Otu1455	0	0.4	0.08	0.8	0.12	56.09	unclass. *Bacteria*	unclass. *Bacteria*	unclass. *Bacteria*	unclass. *Bacteria*	unclass. *Bacteria*	*Bacteria*
Otu0151	0	0.4	0.08	0.8	0.12	56.21	*Rhodococcus*	*Nocardiaceae*	*Actinomycetales*	*Actinobacteria*	*Actinobacteria*	*Bacteria*
Otu0282	0	0.4	0.08	0.8	0.12	56.33	*Haliea*	*Alteromonadaceae*	*Alteromonadales*	*Gammaproteobacteria*	*Proteobacteria*	*Bacteria*
Otu1017	0	0.4	0.08	0.8	0.12	56.45	unclass. *Actinomycetales*	unclass. *Actinomycetales*	*Actinomycetales*	*Actinobacteria*	*Actinobacteria*	*Bacteria*
Otu1563	0	0.4	0.08	0.8	0.12	56.57	unclass. *Proteobacteria*	unclass. *Proteobacteria*	unclass. *Proteobacteria*	unclass. *Proteobacteria*	*Proteobacteria*	*Bacteria*
Otu1577	0	0.4	0.08	0.8	0.12	56.69	*Planctomyces*	*Planctomycetaceae*	*Planctomycetales*	*Planctomycetacia*	*Planctomycetes*	*Bacteria*
Otu1583	0	0.4	0.08	0.8	0.12	56.81	unclass. *Bacteroidetes*	unclass. *Bacteroidetes*	unclass. *Bacteroidetes*	unclass. *Bacteroidetes*	*Bacteroidetes*	*Bacteria*
Otu1599	0	0.4	0.08	0.8	0.12	56.93	unclass. *Deltaproteobacteria*	unclass. *Deltaproteobacteria*	unclass. *Deltaproteobacteria*	*Deltaproteobacteria*	*Proteobacteria*	*Bacteria*
Otu1224	0	0.4	0.08	0.8	0.12	57.05	unclass. *Bacteria*	unclass. *Bacteria*	unclass. *Bacteria*	unclass. *Bacteria*	unclass. *Bacteria*	*Bacteria*
Otu1466	0	0.4	0.08	0.8	0.12	57.17	unclass. *Gammaproteobacteria*	unclass. *Gammaproteobacteria*	unclass. *Gammaproteobacteria*	*Gammaproteobacteria*	*Proteobacteria*	*Bacteria*
Otu1476	0	0.4	0.08	0.8	0.12	57.29	unclass. *Nannocystaceae*	*Nannocystaceae*	*Myxococcales*	*Deltaproteobacteria*	*Proteobacteria*	*Bacteria*
Otu1482	0	0.4	0.08	0.8	0.12	57.41	unclass. *Bacteroidetes*	unclass. *Bacteroidetes*	unclass. *Bacteroidetes*	unclass. *Bacteroidetes*	*Bacteroidetes*	*Bacteria*
Otu0752	0	0.4	0.08	0.8	0.12	57.53	unclass. *Gammaproteobacteria*	unclass. *Gammaproteobacteria*	unclass. *Gammaproteobacteria*	*Gammaproteobacteria*	*Proteobacteria*	*Bacteria*

OTU	Av.A_i (*pH in situ*)	Av.A_i (pH 7.67)	Av.δ_i	Av.δ_i/SD	Av.δ_i %	∑Av.δ_i %	Genus	Family	Order	Class	Phylum	Domain
Otu0755	0	0.4	0.08	0.8	0.12	57.65	unclass. *Proteobacteria*	unclass. *Proteobacteria*	unclass. *Proteobacteria*	unclass. *Proteobacteria*	*Proteobacteria*	*Bacteria*
Otu1200	0	0.4	0.08	0.8	0.12	57.78	unclass. *Bacteria*	unclass. *Bacteria*	unclass. *Bacteria*	unclass. *Bacteria*	unclass. *Bacteria*	*Bacteria*
Otu1442	0	0.4	0.08	0.8	0.12	57.9	unclass. *Bacteroidetes*	unclass. *Bacteroidetes*	unclass. *Bacteroidetes*	unclass. *Bacteroidetes*	*Bacteroidetes*	*Bacteria*
Otu1151	0.2	0.28	0.08	0.69	0.12	58.02	unclass. *Gammaproteobacteria*	unclass. *Gammaproteobacteria*	unclass. *Gammaproteobacteria*	*Gammaproteobacteria*	*Proteobacteria*	*Bacteria*
Otu1214	0.2	0.28	0.08	0.69	0.12	58.14	*Sphingobium*	*Sphingomonadaceae*	*Sphingomonadales*	*Alphaproteobacteria*	*Proteobacteria*	*Bacteria*
Otu0341	0	0.4	0.08	0.8	0.12	58.26	unclass. *Gammaproteobacteria*	unclass. *Gammaproteobacteria*	unclass. *Gammaproteobacteria*	*Gammaproteobacteria*	*Proteobacteria*	*Bacteria*
Otu1009	0	0.4	0.08	0.8	0.12	58.38	unclass. *Gammaproteobacteria*	unclass. *Gammaproteobacteria*	unclass. *Gammaproteobacteria*	*Gammaproteobacteria*	*Proteobacteria*	*Bacteria*
Otu1355	0	0.4	0.08	0.8	0.12	58.5	unclass. *Bacteria*	unclass. *Bacteria*	unclass. *Bacteria*	unclass. *Bacteria*	unclass. *Bacteria*	*Bacteria*
Otu1204	0	0.4	0.08	0.8	0.12	58.62	unclass. *Bacteroidetes*	unclass. *Bacteroidetes*	unclass. *Bacteroidetes*	unclass. *Bacteroidetes*	*Bacteroidetes*	*Bacteria*
Otu1175	0	0.4	0.08	0.8	0.12	58.74	unclass. *Gammaproteobacteria*	unclass. *Gammaproteobacteria*	unclass. *Gammaproteobacteria*	*Gammaproteobacteria*	*Proteobacteria*	*Bacteria*
Otu1363	0.2	0.28	0.07	0.69	0.12	58.85	unclass. *Rhodobacteraceae*	*Rhodobacteraceae*	*Rhodobacterales*	*Alphaproteobacteria*	*Proteobacteria*	*Bacteria*
Otu0238	0	0.4	0.07	0.8	0.12	58.97	*Haliea*	*Alteromonadaceae*	*Alteromonadales*	*Gammaproteobacteria*	*Proteobacteria*	*Bacteria*
Otu1194	0	0.4	0.07	0.8	0.12	59.09	unclass. *Gammaproteobacteria*	unclass. *Gammaproteobacteria*	unclass. *Gammaproteobacteria*	*Gammaproteobacteria*	*Proteobacteria*	*Bacteria*
Otu1229	0	0.4	0.07	0.8	0.12	59.21	*Haliea*	*Alteromonadaceae*	*Alteromonadales*	*Gammaproteobacteria*	*Proteobacteria*	*Bacteria*
Otu1483	0	0.4	0.07	0.8	0.12	59.33	unclass. *Acidobacteria_Gp3*	unclass. *Acidobacteria_Gp3*	unclass. *Acidobacteria_Gp3*	*Acidobacteria_Gp3*	*Acidobacteria*	*Bacteria*
Otu1510	0	0.4	0.07	0.8	0.12	59.45	unclass. *Bacteroidetes*	unclass. *Bacteroidetes*	unclass. *Bacteroidetes*	unclass. *Bacteroidetes*	*Bacteroidetes*	*Bacteria*
Otu0472	0	0.4	0.07	0.8	0.12	59.57	unclass. *Bacteroidetes*	unclass. *Bacteroidetes*	unclass. *Bacteroidetes*	unclass. *Bacteroidetes*	*Bacteroidetes*	*Bacteria*
Otu1141	0	0.4	0.07	0.8	0.12	59.69	unclass. *Chromatiales*	unclass. *Chromatiales*	*Chromatiales*	*Gammaproteobacteria*	*Proteobacteria*	*Bacteria*
Otu1533	0	0.4	0.07	0.8	0.12	59.81	unclass. *Saprospiraceae*	*Saprospiraceae*	*Sphingobacteriales*	*Sphingobacteria*	*Bacteroidetes*	*Bacteria*
Otu1547	0	0.4	0.07	0.8	0.12	59.92	unclass. *Gammaproteobacteria*	unclass. *Gammaproteobacteria*	unclass. *Gammaproteobacteria*	*Gammaproteobacteria*	*Proteobacteria*	*Bacteria*
Otu1348	0.4	0	0.07	0.8	0.12	60.04	unclass. *Gammaproteobacteria*	unclass. *Gammaproteobacteria*	unclass. *Gammaproteobacteria*	*Gammaproteobacteria*	*Proteobacteria*	*Bacteria*
Otu0832	0.28	0.2	0.07	0.7	0.12	60.16	unclass. *Rhodospirillaceae*	*Rhodospirillaceae*	*Rhodospirillales*	*Alphaproteobacteria*	*Proteobacteria*	*Bacteria*
Otu1400	0.2	0.28	0.07	0.69	0.12	60.28	unclass. *Gammaproteobacteria*	unclass. *Gammaproteobacteria*	unclass. *Gammaproteobacteria*	*Gammaproteobacteria*	*Proteobacteria*	*Bacteria*
Otu0177	0.4	0	0.07	0.8	0.12	60.4	*Haliea*	*Alteromonadaceae*	*Alteromonadales*	*Gammaproteobacteria*	*Proteobacteria*	*Bacteria*
Otu1153	0.4	0	0.07	0.8	0.12	60.52	unclass. *Alphaproteobacteria*	unclass. *Alphaproteobacteria*	unclass. *Alphaproteobacteria*	*Alphaproteobacteria*	*Proteobacteria*	*Bacteria*
Otu0724	0.28	0.2	0.07	0.69	0.12	60.63	unclass. *Bacteria*	unclass. *Bacteria*	unclass. *Bacteria*	unclass. *Bacteria*	unclass. *Bacteria*	*Bacteria*
Otu0741	0.4	0	0.07	0.8	0.12	60.75	unclass. *Alphaproteobacteria*	unclass. *Alphaproteobacteria*	unclass. *Alphaproteobacteria*	*Alphaproteobacteria*	*Proteobacteria*	*Bacteria*

OTU	$Av.A_i$ (*pH in situ*)	$Av.A_i$ (pH 7.67)	$Av.\delta_i$	$Av.\delta_i/SD$	$Av.\delta_i$ %	$\sum Av.\delta_i$ %	Genus	Family	Order	Class	Phylum	Domain
Otu0859	0.4	0	0.07	0.8	0.12	60.87	unclass. *Flavobacteriaceae*	*Flavobacteriaceae*	*Flavobacteriales*	*Flavobacteria*	*Bacteroidetes*	*Bacteria*
Otu1116	0.4	0	0.07	0.8	0.12	60.98	unclass. *Gammaproteobacteria*	unclass. *Gammaproteobacteria*	unclass. *Gammaproteobacteria*	*Gammaproteobacteria*	*Proteobacteria*	*Bacteria*
Otu1148	0.4	0	0.07	0.8	0.12	61.1	unclass. *Gammaproteobacteria*	unclass. *Gammaproteobacteria*	unclass. *Gammaproteobacteria*	*Gammaproteobacteria*	*Proteobacteria*	*Bacteria*
Otu0490	1.94	2.22	0.07	1.55	0.11	61.21	unclass. *Burkholderiales*	unclass. *Burkholderiales*	*Burkholderiales*	*Betaproteobacteria*	*Proteobacteria*	*Bacteria*
Otu1238	0	0.35	0.07	0.49	0.1	61.31	unclass. *Opitutae*	unclass. *Opitutae*	unclass. *Opitutae*	*Opitutae*	*Verrucomicrobia*	*Bacteria*
Otu1418	0	0.35	0.07	0.49	0.1	61.42	unclass. *Flammeovirgaceae*	*Flammeovirgaceae*	*Sphingobacteriales*	*Sphingobacteria*	*Bacteroidetes*	*Bacteria*
Otu0313	0	0.35	0.06	0.49	0.1	61.52	unclass. *Gammaproteobacteria*	unclass. *Gammaproteobacteria*	unclass. *Gammaproteobacteria*	*Gammaproteobacteria*	*Proteobacteria*	*Bacteria*
Otu1401	0	0.35	0.06	0.49	0.1	61.62	unclass. *Gammaproteobacteria*	unclass. *Gammaproteobacteria*	unclass. *Gammaproteobacteria*	*Gammaproteobacteria*	*Proteobacteria*	*Bacteria*
Otu1098	0.35	0	0.06	0.49	0.1	61.72	unclass. *Flavobacteriaceae*	*Flavobacteriaceae*	*Flavobacteriales*	*Flavobacteria*	*Bacteroidetes*	*Bacteria*
Otu0280	0.2	0.2	0.06	0.67	0.1	61.82	*Marinobacter*	*Alteromonadaceae*	*Alteromonadales*	*Gammaproteobacteria*	*Proteobacteria*	*Bacteria*
Otu0928	0.2	0.2	0.06	0.67	0.1	61.92	*Crocinitomix*	*Cryomorphaceae*	*Flavobacteriales*	*Flavobacteria*	*Bacteroidetes*	*Bacteria*
Otu0740	0.2	0.2	0.06	0.67	0.1	62.02	unclass. *Betaproteobacteria*	unclass. *Betaproteobacteria*	unclass. *Betaproteobacteria*	*Betaproteobacteria*	*Proteobacteria*	*Bacteria*
Otu1298	0.2	0.2	0.06	0.67	0.1	62.12	unclass. *Gammaproteobacteria*	unclass. *Gammaproteobacteria*	unclass. *Gammaproteobacteria*	*Gammaproteobacteria*	*Proteobacteria*	*Bacteria*
Otu1310	0.2	0.2	0.06	0.67	0.1	62.21	unclass. *Gammaproteobacteria*	unclass. *Gammaproteobacteria*	unclass. *Gammaproteobacteria*	*Gammaproteobacteria*	*Proteobacteria*	*Bacteria*
Otu0042	0.2	0.2	0.06	0.67	0.1	62.31	*Rhodococcus*	*Nocardiaceae*	*Actinomycetales*	*Actinobacteria*	*Actinobacteria*	*Bacteria*
Otu1127	0.2	0.2	0.06	0.67	0.1	62.41	unclass. *Flavobacteriales*	unclass. *Flavobacteriales*	*Flavobacteriales*	*Flavobacteria*	*Bacteroidetes*	*Bacteria*
Otu1237	0.2	0.2	0.06	0.67	0.1	62.51	unclass. *Gammaproteobacteria*	unclass. *Gammaproteobacteria*	unclass. *Gammaproteobacteria*	*Gammaproteobacteria*	*Proteobacteria*	*Bacteria*
Otu0370	0.2	0.2	0.06	0.67	0.1	62.6	unclass. *Gammaproteobacteria*	unclass. *Gammaproteobacteria*	unclass. *Gammaproteobacteria*	*Gammaproteobacteria*	*Proteobacteria*	*Bacteria*
Otu0378	0.2	0.2	0.06	0.67	0.1	62.7	unclass. *Rickettsiaceae*	*Rickettsiaceae*	*Rickettsiales*	*Alphaproteobacteria*	*Proteobacteria*	*Bacteria*
Otu1254	0.2	0.2	0.06	0.67	0.1	62.8	unclass. *Gammaproteobacteria*	unclass. *Gammaproteobacteria*	unclass. *Gammaproteobacteria*	*Gammaproteobacteria*	*Proteobacteria*	*Bacteria*
Otu0732	0.2	0.2	0.06	0.67	0.1	62.89	unclass. *Proteobacteria*	unclass. *Proteobacteria*	unclass. *Proteobacteria*	unclass. *Proteobacteria*	*Proteobacteria*	*Bacteria*
Otu1146	0.2	0.2	0.06	0.67	0.1	62.99	*Nitrosospira*	*Nitrosomonadaceae*	*Nitrosomonadales*	*Betaproteobacteria*	*Proteobacteria*	*Bacteria*
Otu1132	0.2	0.2	0.06	0.67	0.1	63.08	unclass. *Flavobacteriales*	unclass. *Flavobacteriales*	*Flavobacteriales*	*Flavobacteria*	*Bacteroidetes*	*Bacteria*
Otu1260	0.2	0.2	0.06	0.67	0.1	63.18	*Marinobacterium*	*Alteromonadaceae*	*Alteromonadales*	*Gammaproteobacteria*	*Proteobacteria*	*Bacteria*
Otu1261	0.2	0.2	0.06	0.67	0.1	63.28	unclass. *Gammaproteobacteria*	unclass. *Gammaproteobacteria*	unclass. *Gammaproteobacteria*	*Gammaproteobacteria*	*Proteobacteria*	*Bacteria*
Otu0839	0.2	0.2	0.06	0.67	0.1	63.37	unclass. *Bacteria*	unclass. *Bacteria*	unclass. *Bacteria*	unclass. *Bacteria*	unclass. *Bacteria*	*Bacteria*
Otu0651	0.2	0.2	0.06	0.67	0.1	63.47	*Arcobacter*	*Campylobacteraceae*	*Campylobacterales*	*Epsilonproteobacteria*	*Proteobacteria*	*Bacteria*

OTU	Av.A_i (*pH in situ*)	Av.A_i (pH 7.67)	Av.δ_i	Av.δ_i/SD	Av.δ_i %	∑Av.δ_i %	Genus	Family	Order	Class	Phylum	Domain
Otu1120	0.2	0.2	0.06	0.67	0.1	63.56	unclass. *Bacteria*	unclass. *Bacteria*	unclass. *Bacteria*	unclass. *Bacteria*	unclass. *Bacteria*	*Bacteria*
Otu1257	0.2	0.2	0.06	0.67	0.1	63.66	unclass. *Gammaproteobacteria*	unclass. *Gammaproteobacteria*	unclass. *Gammaproteobacteria*	*Gammaproteobacteria*	*Proteobacteria*	*Bacteria*
Otu0698	0.2	0.2	0.06	0.67	0.09	63.75	unclass. *Proteobacteria*	unclass. *Proteobacteria*	unclass. *Proteobacteria*	unclass. *Proteobacteria*	*Proteobacteria*	*Bacteria*
Otu0727	0.2	0.2	0.06	0.67	0.09	63.85	unclass. *Deltaproteobacteria*	unclass. *Deltaproteobacteria*	unclass. *Deltaproteobacteria*	*Deltaproteobacteria*	*Proteobacteria*	*Bacteria*
Otu0148	0.2	0.2	0.06	0.67	0.09	63.94	unclass. *Sphingobacteriales*	unclass. *Sphingobacteriales*	*Sphingobacteriales*	*Sphingobacteria*	*Bacteroidetes*	*Bacteria*
Otu0519	0.2	0.2	0.06	0.67	0.09	64.04	unclass. *Rhodospirillaceae*	*Rhodospirillaceae*	*Rhodospirillales*	*Alphaproteobacteria*	*Proteobacteria*	*Bacteria*
Otu1121	0.2	0.2	0.06	0.67	0.09	64.13	unclass. *Gammaproteobacteria*	unclass. *Gammaproteobacteria*	unclass. *Gammaproteobacteria*	*Gammaproteobacteria*	*Proteobacteria*	*Bacteria*
Otu1124	0.2	0.2	0.06	0.67	0.09	64.23	unclass. *Gammaproteobacteria*	unclass. *Gammaproteobacteria*	unclass. *Gammaproteobacteria*	*Gammaproteobacteria*	*Proteobacteria*	*Bacteria*
Otu1128	0.2	0.2	0.06	0.67	0.09	64.32	unclass. *Colwelliaceae*	*Colwelliaceae*	*Alteromonadales*	*Gammaproteobacteria*	*Proteobacteria*	*Bacteria*
Otu1203	0.2	0.2	0.06	0.67	0.09	64.42	unclass. *Proteobacteria*	unclass. *Proteobacteria*	unclass. *Proteobacteria*	unclass. *Proteobacteria*	*Proteobacteria*	*Bacteria*
Otu0908	0.2	0.2	0.06	0.67	0.09	64.51	unclass. *Proteobacteria*	unclass. *Proteobacteria*	unclass. *Proteobacteria*	unclass. *Proteobacteria*	*Proteobacteria*	*Bacteria*
Otu1352	0.2	0.2	0.06	0.67	0.09	64.6	unclass. *Flavobacteriaceae*	*Flavobacteriaceae*	*Flavobacteriales*	*Flavobacteria*	*Bacteroidetes*	*Bacteria*
Otu1361	0.2	0.2	0.06	0.67	0.09	64.7	unclass. *Bacteria*	unclass. *Bacteria*	unclass. *Bacteria*	unclass. *Bacteria*	unclass. *Bacteria*	*Bacteria*
Otu1408	0.2	0.2	0.06	0.67	0.09	64.79	unclass. *Oceanospirillales*	unclass. *Oceanospirillales*	*Oceanospirillales*	*Gammaproteobacteria*	*Proteobacteria*	*Bacteria*
Otu0031	0.2	0.2	0.06	0.67	0.09	64.89	unclass. *Gammaproteobacteria*	unclass. *Gammaproteobacteria*	unclass. *Gammaproteobacteria*	*Gammaproteobacteria*	*Proteobacteria*	*Bacteria*
Otu0262	0.2	0.2	0.06	0.67	0.09	64.98	unclass. *Gammaproteobacteria*	unclass. *Gammaproteobacteria*	unclass. *Gammaproteobacteria*	*Gammaproteobacteria*	*Proteobacteria*	*Bacteria*
Otu1145	0.2	0.2	0.06	0.67	0.09	65.08	unclass. *Bacteria*	unclass. *Bacteria*	unclass. *Bacteria*	unclass. *Bacteria*	unclass. *Bacteria*	*Bacteria*
Otu1356	0.2	0.2	0.06	0.67	0.09	65.17	unclass. *Gammaproteobacteria*	unclass. *Gammaproteobacteria*	unclass. *Gammaproteobacteria*	*Gammaproteobacteria*	*Proteobacteria*	*Bacteria*
Otu1377	0.2	0.2	0.06	0.67	0.09	65.26	unclass. *Proteobacteria*	unclass. *Proteobacteria*	unclass. *Proteobacteria*	unclass. *Proteobacteria*	*Proteobacteria*	*Bacteria*
Otu1405	0.2	0.2	0.06	0.67	0.09	65.36	unclass. *Bacteroidetes*	unclass. *Bacteroidetes*	unclass. *Bacteroidetes*	unclass. *Bacteroidetes*	*Bacteroidetes*	*Bacteria*
Otu0140	0.2	0.2	0.06	0.67	0.09	65.45	*Oleispira*	*Oceanospirillaceae*	*Oceanospirillales*	*Gammaproteobacteria*	*Proteobacteria*	*Bacteria*
Otu1388	0.2	0.2	0.06	0.67	0.09	65.55	unclass. *Bacteria*	unclass. *Bacteria*	unclass. *Bacteria*	unclass. *Bacteria*	unclass. *Bacteria*	*Bacteria*
Otu1283	0.28	0	0.06	0.49	0.09	65.64	*Croceibacter*	*Flavobacteriaceae*	*Flavobacteriales*	*Flavobacteria*	*Bacteroidetes*	*Bacteria*
Otu0739	0.28	0	0.05	0.49	0.09	65.72	unclass. *Proteobacteria*	unclass. *Proteobacteria*	unclass. *Proteobacteria*	unclass. *Proteobacteria*	*Proteobacteria*	*Bacteria*
Otu0273	0	0.28	0.05	0.49	0.09	65.81	unclass. *Bacteroidetes*	unclass. *Bacteroidetes*	unclass. *Bacteroidetes*	unclass. *Bacteroidetes*	*Bacteroidetes*	*Bacteria*
Otu0743	0	0.28	0.05	0.49	0.09	65.9	unclass. *Verrucomicrobia*	unclass. *Verrucomicrobia*	unclass. *Verrucomicrobia*	unclass. *Verrucomicrobia*	*Verrucomicrobia*	*Bacteria*
Otu0868	0	0.28	0.05	0.49	0.09	65.98	unclass. *Alphaproteobacteria*	unclass. *Alphaproteobacteria*	unclass. *Alphaproteobacteria*	*Alphaproteobacteria*	*Proteobacteria*	*Bacteria*

OTU	Av.A_i (*pH in situ*)	Av.A_i (pH 7.67)	Av.δ_i	Av.δ_i/SD	Av.δ_i %	∑Av.δ_i %	Genus	Family	Order	Class	Phylum	Domain
Otu1213	0	0.28	0.05	0.49	0.09	66.07	unclass. *Gammaproteobacteria*	unclass. *Gammaproteobacteria*	unclass. *Gammaproteobacteria*	*Gammaproteobacteria*	*Proteobacteria*	*Bacteria*
Otu1603	0	0.28	0.05	0.49	0.09	66.15	unclass. *Gammaproteobacteria*	unclass. *Gammaproteobacteria*	unclass. *Gammaproteobacteria*	*Gammaproteobacteria*	*Proteobacteria*	*Bacteria*
Otu1604	0	0.28	0.05	0.49	0.09	66.24	unclass. *Gammaproteobacteria*	unclass. *Gammaproteobacteria*	unclass. *Gammaproteobacteria*	*Gammaproteobacteria*	*Proteobacteria*	*Bacteria*
Otu1613	0	0.28	0.05	0.49	0.09	66.33	unclass. *Proteobacteria*	unclass. *Proteobacteria*	unclass. *Proteobacteria*	unclass. *Proteobacteria*	*Proteobacteria*	*Bacteria*
Otu1368	0	0.28	0.05	0.49	0.09	66.41	unclass. *Gammaproteobacteria*	unclass. *Gammaproteobacteria*	unclass. *Gammaproteobacteria*	*Gammaproteobacteria*	*Proteobacteria*	*Bacteria*
Otu1416	0	0.28	0.05	0.49	0.09	66.5	unclass. *Verrucomicrobia*	unclass. *Verrucomicrobia*	unclass. *Verrucomicrobia*	unclass. *Verrucomicrobia*	*Verrucomicrobia*	*Bacteria*
Otu0856	0	0.28	0.05	0.49	0.08	66.58	unclass. *Gammaproteobacteria*	unclass. *Gammaproteobacteria*	unclass. *Gammaproteobacteria*	*Gammaproteobacteria*	*Proteobacteria*	*Bacteria*
Otu1365	0	0.28	0.05	0.49	0.08	66.67	unclass. *Bacteria*	unclass. *Bacteria*	unclass. *Bacteria*	unclass. *Bacteria*	unclass. *Bacteria*	*Bacteria*
Otu1422	0	0.28	0.05	0.49	0.08	66.75	unclass. *Pseudomonadales*	unclass. *Pseudomonadales*	*Pseudomonadales*	*Gammaproteobacteria*	*Proteobacteria*	*Bacteria*
Otu1651	0	0.28	0.05	0.49	0.08	66.84	unclass. *Deltaproteobacteria*	unclass. *Deltaproteobacteria*	unclass. *Deltaproteobacteria*	*Deltaproteobacteria*	*Proteobacteria*	*Bacteria*
Otu1652	0	0.28	0.05	0.49	0.08	66.92	unclass. *Gammaproteobacteria*	unclass. *Gammaproteobacteria*	unclass. *Gammaproteobacteria*	*Gammaproteobacteria*	*Proteobacteria*	*Bacteria*
Otu1653	0	0.28	0.05	0.49	0.08	67.01	unclass. *Proteobacteria*	unclass. *Proteobacteria*	unclass. *Proteobacteria*	unclass. *Proteobacteria*	*Proteobacteria*	*Bacteria*
Otu1659	0	0.28	0.05	0.49	0.08	67.09	unclass. *Sphingomonadaceae*	*Sphingomonadaceae*	*Sphingomonadales*	*Alphaproteobacteria*	*Proteobacteria*	*Bacteria*
Otu1206	0	0.28	0.05	0.49	0.08	67.18	unclass. *Bacteria*	unclass. *Bacteria*	unclass. *Bacteria*	unclass. *Bacteria*	unclass. *Bacteria*	*Bacteria*
Otu1299	0	0.28	0.05	0.49	0.08	67.26	unclass. *Bacteroidetes*	unclass. *Bacteroidetes*	unclass. *Bacteroidetes*	unclass. *Bacteroidetes*	*Bacteroidetes*	*Bacteria*
Otu1315	0	0.28	0.05	0.49	0.08	67.35	unclass. *Gammaproteobacteria*	unclass. *Gammaproteobacteria*	unclass. *Gammaproteobacteria*	*Gammaproteobacteria*	*Proteobacteria*	*Bacteria*
Otu1439	0	0.28	0.05	0.49	0.08	67.43	unclass. *Gammaproteobacteria*	unclass. *Gammaproteobacteria*	unclass. *Gammaproteobacteria*	*Gammaproteobacteria*	*Proteobacteria*	*Bacteria*
Otu1554	0	0.28	0.05	0.49	0.08	67.51	unclass. *Bacteroidetes*	unclass. *Bacteroidetes*	unclass. *Bacteroidetes*	unclass. *Bacteroidetes*	*Bacteroidetes*	*Bacteria*
Otu1556	0	0.28	0.05	0.49	0.08	67.6	unclass. *Sphingobacteriales*	unclass. *Sphingobacteriales*	*Sphingobacteriales*	*Sphingobacteria*	*Bacteroidetes*	*Bacteria*
Otu1557	0	0.28	0.05	0.49	0.08	67.68	unclass. *Gammaproteobacteria*	unclass. *Gammaproteobacteria*	unclass. *Gammaproteobacteria*	*Gammaproteobacteria*	*Proteobacteria*	*Bacteria*
Otu1565	0	0.28	0.05	0.49	0.08	67.77	unclass. *Alphaproteobacteria*	unclass. *Alphaproteobacteria*	unclass. *Alphaproteobacteria*	*Alphaproteobacteria*	*Proteobacteria*	*Bacteria*
Otu0256	0.28	0	0.05	0.49	0.08	67.85	*Glaciecola*	*Alteromonadaceae*	*Alteromonadales*	*Gammaproteobacteria*	*Proteobacteria*	*Bacteria*
Otu1007	0.28	0	0.05	0.49	0.08	67.94	unclass. *Erythrobacteraceae*	*Erythrobacteraceae*	*Sphingomonadales*	*Alphaproteobacteria*	*Proteobacteria*	*Bacteria*
Otu1233	0.28	0	0.05	0.49	0.08	68.02	unclass. *Flavobacteriaceae*	*Flavobacteriaceae*	*Flavobacteriales*	*Flavobacteria*	*Bacteroidetes*	*Bacteria*
Otu1276	0.28	0	0.05	0.49	0.08	68.11	unclass. *Flammeovirgaceae*	*Flammeovirgaceae*	*Sphingobacteriales*	*Sphingobacteria*	*Bacteroidetes*	*Bacteria*
Otu0488	0	0.28	0.05	0.49	0.08	68.19	unclass. *Gammaproteobacteria*	unclass. *Gammaproteobacteria*	unclass. *Gammaproteobacteria*	*Gammaproteobacteria*	*Proteobacteria*	*Bacteria*
Otu1487	0	0.28	0.05	0.49	0.08	68.27	unclass. *Bacteria*	unclass. *Bacteria*	unclass. *Bacteria*	unclass. *Bacteria*	unclass. *Bacteria*	*Bacteria*

OTU	Av.A_i (*pH in situ*)	Av.A_i (pH 7.67)	Av.δ_i	Av.δ_i/SD	Av.δ_i %	∑Av.δ_i %	Genus	Family	Order	Class	Phylum	Domain
Otu1491	0	0.28	0.05	0.49	0.08	68.36	unclass. *Bacteria*	unclass. *Bacteria*	unclass. *Bacteria*	unclass. *Bacteria*	unclass. *Bacteria*	*Bacteria*
Otu1493	0	0.28	0.05	0.49	0.08	68.44	*Amphritea*	*Oceanospirillaceae*	*Oceanospirillales*	*Gammaproteobacteria*	*Proteobacteria*	*Bacteria*
Otu1536	0	0.28	0.05	0.49	0.08	68.52	*Nannocystis*	*Nannocystaceae*	*Myxococcales*	*Deltaproteobacteria*	*Proteobacteria*	*Bacteria*
Otu1134	0.28	0	0.05	0.49	0.08	68.6	unclass. *Gammaproteobacteria*	unclass. *Gammaproteobacteria*	unclass. *Gammaproteobacteria*	*Gammaproteobacteria*	*Proteobacteria*	*Bacteria*
Otu0518	0.28	0	0.05	0.49	0.08	68.69	unclass. *Proteobacteria*	unclass. *Proteobacteria*	unclass. *Proteobacteria*	unclass. *Proteobacteria*	*Proteobacteria*	*Bacteria*
Otu1235	0.28	0	0.05	0.49	0.08	68.77	unclass. *Proteobacteria*	unclass. *Proteobacteria*	unclass. *Proteobacteria*	unclass. *Proteobacteria*	*Proteobacteria*	*Bacteria*
Otu1278	0.28	0	0.05	0.49	0.08	68.85	unclass. *Betaproteobacteria*	unclass. *Betaproteobacteria*	unclass. *Betaproteobacteria*	*Betaproteobacteria*	*Proteobacteria*	*Bacteria*
Otu0253	0.2	0	0.04	0.49	0.06	68.91	unclass. *Saprospiraceae*	*Saprospiraceae*	*Sphingobacteriales*	*Sphingobacteria*	*Bacteroidetes*	*Bacteria*
Otu0324	0.2	0	0.04	0.49	0.06	68.98	*Nisaea*	*Rhodospirillaceae*	*Rhodospirillales*	*Alphaproteobacteria*	*Proteobacteria*	*Bacteria*
Otu0558	0.2	0	0.04	0.49	0.06	69.04	unclass. *Gammaproteobacteria*	unclass. *Gammaproteobacteria*	unclass. *Gammaproteobacteria*	*Gammaproteobacteria*	*Proteobacteria*	*Bacteria*
Otu0704	0.2	0	0.04	0.49	0.06	69.1	unclass. *Proteobacteria*	unclass. *Proteobacteria*	unclass. *Proteobacteria*	unclass. *Proteobacteria*	*Proteobacteria*	*Bacteria*
Otu1215	0.2	0	0.04	0.49	0.06	69.17	unclass. *Gammaproteobacteria*	unclass. *Gammaproteobacteria*	unclass. *Gammaproteobacteria*	*Gammaproteobacteria*	*Proteobacteria*	*Bacteria*
Otu1284	0.2	0	0.04	0.49	0.06	69.23	unclass. *Bacteroidetes*	unclass. *Bacteroidetes*	unclass. *Bacteroidetes*	unclass. *Bacteroidetes*	*Bacteroidetes*	*Bacteria*
Otu1285	0.2	0	0.04	0.49	0.06	69.29	unclass. *Myxococcales*	unclass. *Myxococcales*	*Myxococcales*	*Deltaproteobacteria*	*Proteobacteria*	*Bacteria*
Otu1287	0.2	0	0.04	0.49	0.06	69.36	unclass. *Flavobacteriaceae*	*Flavobacteriaceae*	*Flavobacteriales*	*Flavobacteria*	*Bacteroidetes*	*Bacteria*
Otu1288	0.2	0	0.04	0.49	0.06	69.42	unclass. *Bacteria*	unclass. *Bacteria*	unclass. *Bacteria*	unclass. *Bacteria*	unclass. *Bacteria*	*Bacteria*
Otu1289	0.2	0	0.04	0.49	0.06	69.49	unclass. *Bacteroidetes*	unclass. *Bacteroidetes*	unclass. *Bacteroidetes*	unclass. *Bacteroidetes*	*Bacteroidetes*	*Bacteria*
Otu1290	0.2	0	0.04	0.49	0.06	69.55	unclass. *Bacteria*	unclass. *Bacteria*	unclass. *Bacteria*	unclass. *Bacteria*	unclass. *Bacteria*	*Bacteria*
Otu1291	0.2	0	0.04	0.49	0.06	69.61	unclass. *Gammaproteobacteria*	unclass. *Gammaproteobacteria*	unclass. *Gammaproteobacteria*	*Gammaproteobacteria*	*Proteobacteria*	*Bacteria*
Otu1292	0.2	0	0.04	0.49	0.06	69.68	unclass. *Deltaproteobacteria*	unclass. *Deltaproteobacteria*	unclass. *Deltaproteobacteria*	*Deltaproteobacteria*	*Proteobacteria*	*Bacteria*
Otu1293	0.2	0	0.04	0.49	0.06	69.74	unclass. *Bacteroidetes*	unclass. *Bacteroidetes*	unclass. *Bacteroidetes*	unclass. *Bacteroidetes*	*Bacteroidetes*	*Bacteria*
Otu1294	0.2	0	0.04	0.49	0.06	69.8	unclass. *Bacteria*	unclass. *Bacteria*	unclass. *Bacteria*	unclass. *Bacteria*	unclass. *Bacteria*	*Bacteria*
Otu1295	0.2	0	0.04	0.49	0.06	69.87	unclass. *Actinomycetales*	unclass. *Actinomycetales*	*Actinomycetales*	*Actinobacteria*	*Actinobacteria*	*Bacteria*
Otu1301	0.2	0	0.04	0.49	0.06	69.93	unclass. *Alphaproteobacteria*	unclass. *Alphaproteobacteria*	unclass. *Alphaproteobacteria*	*Alphaproteobacteria*	*Proteobacteria*	*Bacteria*
Otu1302	0.2	0	0.04	0.49	0.06	70	unclass. *Bacteroidetes*	unclass. *Bacteroidetes*	unclass. *Bacteroidetes*	unclass. *Bacteroidetes*	*Bacteroidetes*	*Bacteria*
Otu1303	0.2	0	0.04	0.49	0.06	70.06	unclass. *Acidobacteria_Gp17*	unclass. *Acidobacteria_Gp17*	unclass. *Acidobacteria_Gp17*	*Acidobacteria_Gp17*	*Acidobacteria*	*Bacteria*
Otu1305	0.2	0	0.04	0.49	0.06	70.12	unclass. *Sphingobacteriales*	unclass. *Sphingobacteriales*	*Sphingobacteriales*	*Sphingobacteria*	*Bacteroidetes*	*Bacteria*

OTU	Av.A_i (*pH in situ*)	Av.A_i (pH 7.67)	Av.δ_i	Av.δ_i/SD	Av.δ_i %	∑Av.δ_i %	Genus	Family	Order	Class	Phylum	Domain
Otu1306	0.2	0	0.04	0.49	0.06	70.19	unclass. *Gammaproteobacteria*	unclass. *Gammaproteobacteria*	unclass. *Gammaproteobacteria*	*Gammaproteobacteria*	*Proteobacteria*	*Bacteria*
Otu1307	0.2	0	0.04	0.49	0.06	70.25	unclass. *Gammaproteobacteria*	unclass. *Gammaproteobacteria*	unclass. *Gammaproteobacteria*	*Gammaproteobacteria*	*Proteobacteria*	*Bacteria*
Otu1308	0.2	0	0.04	0.49	0.06	70.32	unclass. *Flavobacteriaceae*	*Flavobacteriaceae*	*Flavobacteriales*	*Flavobacteria*	*Bacteroidetes*	*Bacteria*
Otu1309	0.2	0	0.04	0.49	0.06	70.38	unclass. *Gammaproteobacteria*	unclass. *Gammaproteobacteria*	unclass. *Gammaproteobacteria*	*Gammaproteobacteria*	*Proteobacteria*	*Bacteria*
Otu1311	0.2	0	0.04	0.49	0.06	70.44	unclass. *Rhodobacteraceae*	*Rhodobacteraceae*	*Rhodobacterales*	*Alphaproteobacteria*	*Proteobacteria*	*Bacteria*
Otu1312	0.2	0	0.04	0.49	0.06	70.51	unclass. *Proteobacteria*	unclass. *Proteobacteria*	unclass. *Proteobacteria*	unclass. *Proteobacteria*	*Proteobacteria*	*Bacteria*
Otu1314	0.2	0	0.04	0.49	0.06	70.57	unclass. *Acidobacteria_Gp22*	unclass. *Acidobacteria_Gp22*	unclass. *Acidobacteria_Gp22*	*Acidobacteria_Gp22*	*Acidobacteria*	*Bacteria*
Otu1316	0.2	0	0.04	0.49	0.06	70.63	unclass. *Gammaproteobacteria*	unclass. *Gammaproteobacteria*	unclass. *Gammaproteobacteria*	*Gammaproteobacteria*	*Proteobacteria*	*Bacteria*
Otu0115	0.2	0	0.04	0.49	0.06	70.7	*Glaciecola*	*Alteromonadaceae*	*Alteromonadales*	*Gammaproteobacteria*	*Proteobacteria*	*Bacteria*
Otu0270	0.2	0	0.04	0.49	0.06	70.76	unclass. *Bacteroidetes*	unclass. *Bacteroidetes*	unclass. *Bacteroidetes*	unclass. *Bacteroidetes*	*Bacteroidetes*	*Bacteria*
Otu0303	0.2	0	0.04	0.49	0.06	70.82	unclass. *Bacteroidetes*	unclass. *Bacteroidetes*	unclass. *Bacteroidetes*	unclass. *Bacteroidetes*	*Bacteroidetes*	*Bacteria*
Otu0349	0.2	0	0.04	0.49	0.06	70.88	unclass. *Gammaproteobacteria*	unclass. *Gammaproteobacteria*	unclass. *Gammaproteobacteria*	*Gammaproteobacteria*	*Proteobacteria*	*Bacteria*
Otu0362	0.2	0	0.04	0.49	0.06	70.94	unclass. *Bacteroidetes*	unclass. *Bacteroidetes*	unclass. *Bacteroidetes*	unclass. *Bacteroidetes*	*Bacteroidetes*	*Bacteria*
Otu0367	0.2	0	0.04	0.49	0.06	71	unclass. *Bacteroidetes*	unclass. *Bacteroidetes*	unclass. *Bacteroidetes*	unclass. *Bacteroidetes*	*Bacteroidetes*	*Bacteria*
Otu0772	0.2	0	0.04	0.49	0.06	71.06	unclass. *Alphaproteobacteria*	unclass. *Alphaproteobacteria*	unclass. *Alphaproteobacteria*	*Alphaproteobacteria*	*Proteobacteria*	*Bacteria*
Otu0808	0.2	0	0.04	0.49	0.06	71.13	unclass. *Saprospiraceae*	*Saprospiraceae*	*Sphingobacteriales*	*Sphingobacteria*	*Bacteroidetes*	*Bacteria*
Otu0933	0.2	0	0.04	0.49	0.06	71.19	*Haliea*	*Alteromonadaceae*	*Alteromonadales*	*Gammaproteobacteria*	*Proteobacteria*	*Bacteria*
Otu1001	0.2	0	0.04	0.49	0.06	71.25	unclass. *Flavobacteriales*	unclass. *Flavobacteriales*	*Flavobacteriales*	*Flavobacteria*	*Bacteroidetes*	*Bacteria*
Otu1170	0.2	0	0.04	0.49	0.06	71.31	unclass. *Bacteria*	unclass. *Bacteria*	unclass. *Bacteria*	unclass. *Bacteria*	unclass. *Bacteria*	*Bacteria*
Otu1318	0.2	0	0.04	0.49	0.06	71.37	unclass. *Bacteroidetes*	unclass. *Bacteroidetes*	unclass. *Bacteroidetes*	unclass. *Bacteroidetes*	*Bacteroidetes*	*Bacteria*
Otu1319	0.2	0	0.04	0.49	0.06	71.43	unclass. *Gammaproteobacteria*	unclass. *Gammaproteobacteria*	unclass. *Gammaproteobacteria*	*Gammaproteobacteria*	*Proteobacteria*	*Bacteria*
Otu1320	0.2	0	0.04	0.49	0.06	71.49	unclass. *Bacteria*	unclass. *Bacteria*	unclass. *Bacteria*	unclass. *Bacteria*	unclass. *Bacteria*	*Bacteria*
Otu1321	0.2	0	0.04	0.49	0.06	71.56	unclass. *Flavobacteriaceae*	*Flavobacteriaceae*	*Flavobacteriales*	*Flavobacteria*	*Bacteroidetes*	*Bacteria*
Otu1322	0.2	0	0.04	0.49	0.06	71.62	unclass. *Bacteria*	unclass. *Bacteria*	unclass. *Bacteria*	unclass. *Bacteria*	unclass. *Bacteria*	*Bacteria*
Otu1323	0.2	0	0.04	0.49	0.06	71.68	unclass. *Oceanospirillaceae*	*Oceanospirillaceae*	*Oceanospirillales*	*Gammaproteobacteria*	*Proteobacteria*	*Bacteria*
Otu1324	0.2	0	0.04	0.49	0.06	71.74	unclass. *Alphaproteobacteria*	unclass. *Alphaproteobacteria*	unclass. *Alphaproteobacteria*	*Alphaproteobacteria*	*Proteobacteria*	*Bacteria*
Otu1325	0.2	0	0.04	0.49	0.06	71.8	*Algibacter*	*Flavobacteriaceae*	*Flavobacteriales*	*Flavobacteria*	*Bacteroidetes*	*Bacteria*

OTU	Av.A_i (*pH in situ*)	Av.A_i (pH 7.67)	Av.δ_i	Av.δ_i/SD	Av.δ_i %	∑Av.δ_i %	Genus	Family	Order	Class	Phylum	Domain
Otu1326	0.2	0	0.04	0.49	0.06	71.86	unclass. *Gammaproteobacteria*	unclass. *Gammaproteobacteria*	unclass. *Gammaproteobacteria*	*Gammaproteobacteria*	*Proteobacteria*	*Bacteria*
Otu1327	0.2	0	0.04	0.49	0.06	71.93	unclass. *Gammaproteobacteria*	unclass. *Gammaproteobacteria*	unclass. *Gammaproteobacteria*	*Gammaproteobacteria*	*Proteobacteria*	*Bacteria*
Otu1329	0.2	0	0.04	0.49	0.06	71.99	unclass. *Gammaproteobacteria*	unclass. *Gammaproteobacteria*	unclass. *Gammaproteobacteria*	*Gammaproteobacteria*	*Proteobacteria*	*Bacteria*
Otu1330	0.2	0	0.04	0.49	0.06	72.05	unclass. *Bacteria*	unclass. *Bacteria*	unclass. *Bacteria*	unclass. *Bacteria*	unclass. *Bacteria*	*Bacteria*
Otu1331	0.2	0	0.04	0.49	0.06	72.11	unclass. *Saprospiraceae*	*Saprospiraceae*	*Sphingobacteriales*	*Sphingobacteria*	*Bacteroidetes*	*Bacteria*
Otu1335	0.2	0	0.04	0.49	0.06	72.17	unclass. *Chromatiales*	unclass. *Chromatiales*	*Chromatiales*	*Gammaproteobacteria*	*Proteobacteria*	*Bacteria*
Otu1336	0.2	0	0.04	0.49	0.06	72.23	unclass. *Bacteria*	unclass. *Bacteria*	unclass. *Bacteria*	unclass. *Bacteria*	unclass. *Bacteria*	*Bacteria*
Otu1337	0.2	0	0.04	0.49	0.06	72.29	unclass. *Bacteria*	unclass. *Bacteria*	unclass. *Bacteria*	unclass. *Bacteria*	unclass. *Bacteria*	*Bacteria*
Otu1339	0.2	0	0.04	0.49	0.06	72.36	unclass. *Bacteria*	unclass. *Bacteria*	unclass. *Bacteria*	unclass. *Bacteria*	unclass. *Bacteria*	*Bacteria*
Otu1340	0.2	0	0.04	0.49	0.06	72.42	unclass. *Proteobacteria*	unclass. *Proteobacteria*	unclass. *Proteobacteria*	unclass. *Proteobacteria*	*Proteobacteria*	*Bacteria*
Otu1341	0.2	0	0.04	0.49	0.06	72.48	unclass. *Gammaproteobacteria*	unclass. *Gammaproteobacteria*	unclass. *Gammaproteobacteria*	*Gammaproteobacteria*	*Proteobacteria*	*Bacteria*
Otu1342	0.2	0	0.04	0.49	0.06	72.54	unclass. *Actinobacteria*	unclass. *Actinobacteria*	unclass. *Actinobacteria*	*Actinobacteria*	*Actinobacteria*	*Bacteria*
Otu1344	0.2	0	0.04	0.49	0.06	72.6	unclass. *Gammaproteobacteria*	unclass. *Gammaproteobacteria*	unclass. *Gammaproteobacteria*	*Gammaproteobacteria*	*Proteobacteria*	*Bacteria*
Otu1345	0.2	0	0.04	0.49	0.06	72.66	unclass. *Bacteria*	unclass. *Bacteria*	unclass. *Bacteria*	unclass. *Bacteria*	unclass. *Bacteria*	*Bacteria*
Otu1347	0.2	0	0.04	0.49	0.06	72.72	unclass. *Gammaproteobacteria*	unclass. *Gammaproteobacteria*	unclass. *Gammaproteobacteria*	*Gammaproteobacteria*	*Proteobacteria*	*Bacteria*
Otu1349	0.2	0	0.04	0.49	0.06	72.79	unclass. *Bacteria*	unclass. *Bacteria*	unclass. *Bacteria*	unclass. *Bacteria*	unclass. *Bacteria*	*Bacteria*
Otu0173	0	0.2	0.04	0.49	0.06	72.85	unclass. *Alteromonadaceae*	*Alteromonadaceae*	*Alteromonadales*	*Gammaproteobacteria*	*Proteobacteria*	*Bacteria*
Otu0204	0	0.2	0.04	0.49	0.06	72.91	unclass. *Colwelliaceae*	*Colwelliaceae*	*Alteromonadales*	*Gammaproteobacteria*	*Proteobacteria*	*Bacteria*
Otu0279	0	0.2	0.04	0.49	0.06	72.97	unclass. *Gammaproteobacteria*	unclass. *Gammaproteobacteria*	unclass. *Gammaproteobacteria*	*Gammaproteobacteria*	*Proteobacteria*	*Bacteria*
Otu0311	0	0.2	0.04	0.49	0.06	73.03	unclass. *Gammaproteobacteria*	unclass. *Gammaproteobacteria*	unclass. *Gammaproteobacteria*	*Gammaproteobacteria*	*Proteobacteria*	*Bacteria*
Otu0555	0	0.2	0.04	0.49	0.06	73.09	*Maricaulis*	*Hyphomonadaceae*	*Caulobacterales*	*Alphaproteobacteria*	*Proteobacteria*	*Bacteria*
Otu0631	0	0.2	0.04	0.49	0.06	73.15	*Arcobacter*	*Campylobacteraceae*	*Campylobacterales*	*Epsilonproteobacteria*	*Proteobacteria*	*Bacteria*
Otu0814	0	0.2	0.04	0.49	0.06	73.21	unclass. *Proteobacteria*	unclass. *Proteobacteria*	unclass. *Proteobacteria*	unclass. *Proteobacteria*	*Proteobacteria*	*Bacteria*
Otu0957	0	0.2	0.04	0.49	0.06	73.27	unclass. *Oceanospirillales*	unclass. *Oceanospirillales*	*Oceanospirillales*	*Gammaproteobacteria*	*Proteobacteria*	*Bacteria*
Otu1016	0	0.2	0.04	0.49	0.06	73.33	unclass. *Verrucomicrobiales*	unclass. *Verrucomicrobiales*	*Verrucomicrobiales*	*Verrucomicrobiae*	*Verrucomicrobia*	*Bacteria*
Otu1231	0	0.2	0.04	0.49	0.06	73.39	unclass. *Gammaproteobacteria*	unclass. *Gammaproteobacteria*	unclass. *Gammaproteobacteria*	*Gammaproteobacteria*	*Proteobacteria*	*Bacteria*
Otu1236	0	0.2	0.04	0.49	0.06	73.45	unclass. *Bacteria*	unclass. *Bacteria*	unclass. *Bacteria*	unclass. *Bacteria*	unclass. *Bacteria*	*Bacteria*

OTU	Av.A_i (*pH in situ*)	Av.A_i (pH 7.67)	Av.δ_i	Av.δ_i/SD	Av.δ_i %	∑Av.δ_i %	Genus	Family	Order	Class	Phylum	Domain
Otu1313	0	0.2	0.04	0.49	0.06	73.52	unclass. *Saprospiraceae*	*Saprospiraceae*	*Sphingobacteriales*	*Sphingobacteria*	*Bacteroidetes*	*Bacteria*
Otu1407	0	0.2	0.04	0.49	0.06	73.58	unclass. *Flavobacteriales*	unclass. *Flavobacteriales*	*Flavobacteriales*	*Flavobacteria*	*Bacteroidetes*	*Bacteria*
Otu1444	0	0.2	0.04	0.49	0.06	73.64	unclass. *Bacteria*	unclass. *Bacteria*	unclass. *Bacteria*	unclass. *Bacteria*	unclass. *Bacteria*	*Bacteria*
Otu1451	0	0.2	0.04	0.49	0.06	73.7	unclass. *Proteobacteria*	unclass. *Proteobacteria*	unclass. *Proteobacteria*	unclass. *Proteobacteria*	*Proteobacteria*	*Bacteria*
Otu1516	0	0.2	0.04	0.49	0.06	73.76	unclass. *Sphingobacteriales*	unclass. *Sphingobacteriales*	*Sphingobacteriales*	*Sphingobacteria*	*Bacteroidetes*	*Bacteria*
Otu1526	0	0.2	0.04	0.49	0.06	73.82	unclass. *Flavobacteriaceae*	*Flavobacteriaceae*	*Flavobacteriales*	*Flavobacteria*	*Bacteroidetes*	*Bacteria*
Otu1548	0	0.2	0.04	0.49	0.06	73.88	unclass. *Flavobacteriaceae*	*Flavobacteriaceae*	*Flavobacteriales*	*Flavobacteria*	*Bacteroidetes*	*Bacteria*
Otu1606	0	0.2	0.04	0.49	0.06	73.94	unclass. *Bacteria*	unclass. *Bacteria*	unclass. *Bacteria*	unclass. *Bacteria*	unclass. *Bacteria*	*Bacteria*
Otu1607	0	0.2	0.04	0.49	0.06	74	unclass. *Saprospiraceae*	*Saprospiraceae*	*Sphingobacteriales*	*Sphingobacteria*	*Bacteroidetes*	*Bacteria*
Otu1608	0	0.2	0.04	0.49	0.06	74.06	unclass. *Bacteroidetes*	unclass. *Bacteroidetes*	unclass. *Bacteroidetes*	unclass. *Bacteroidetes*	*Bacteroidetes*	*Bacteria*
Otu1609	0	0.2	0.04	0.49	0.06	74.12	unclass. *Bacteria*	unclass. *Bacteria*	unclass. *Bacteria*	unclass. *Bacteria*	unclass. *Bacteria*	*Bacteria*
Otu1610	0	0.2	0.04	0.49	0.06	74.18	unclass. *Gammaproteobacteria*	unclass. *Gammaproteobacteria*	unclass. *Gammaproteobacteria*	*Gammaproteobacteria*	*Proteobacteria*	*Bacteria*
Otu1611	0	0.2	0.04	0.49	0.06	74.25	unclass. *Alphaproteobacteria*	unclass. *Alphaproteobacteria*	unclass. *Alphaproteobacteria*	*Alphaproteobacteria*	*Proteobacteria*	*Bacteria*
Otu1612	0	0.2	0.04	0.49	0.06	74.31	unclass. *Gammaproteobacteria*	unclass. *Gammaproteobacteria*	unclass. *Gammaproteobacteria*	*Gammaproteobacteria*	*Proteobacteria*	*Bacteria*
Otu1614	0	0.2	0.04	0.49	0.06	74.37	unclass. *Bacteria*	unclass. *Bacteria*	unclass. *Bacteria*	unclass. *Bacteria*	unclass. *Bacteria*	*Bacteria*
Otu1615	0	0.2	0.04	0.49	0.06	74.43	*Rhodopirellula*	*Planctomycetaceae*	*Planctomycetales*	*Planctomycetacia*	*Planctomycetes*	*Bacteria*
Otu1616	0	0.2	0.04	0.49	0.06	74.49	*Malonomonas*	*Desulfuromonadaceae*	*Desulfuromonadales*	*Deltaproteobacteria*	*Proteobacteria*	*Bacteria*
Otu1618	0	0.2	0.04	0.49	0.06	74.55	unclass. *Chromatiales*	unclass. *Chromatiales*	*Chromatiales*	*Gammaproteobacteria*	*Proteobacteria*	*Bacteria*
Otu1619	0	0.2	0.04	0.49	0.06	74.61	unclass. *Gammaproteobacteria*	unclass. *Gammaproteobacteria*	unclass. *Gammaproteobacteria*	*Gammaproteobacteria*	*Proteobacteria*	*Bacteria*
Otu1621	0	0.2	0.04	0.49	0.06	74.67	unclass. *Bacteria*	unclass. *Bacteria*	unclass. *Bacteria*	unclass. *Bacteria*	unclass. *Bacteria*	*Bacteria*
Otu1622	0	0.2	0.04	0.49	0.06	74.73	unclass. *Gammaproteobacteria*	unclass. *Gammaproteobacteria*	unclass. *Gammaproteobacteria*	*Gammaproteobacteria*	*Proteobacteria*	*Bacteria*
Otu1623	0	0.2	0.04	0.49	0.06	74.79	unclass. *Verrucomicrobia*	unclass. *Verrucomicrobia*	unclass. *Verrucomicrobia*	unclass. *Verrucomicrobia*	*Verrucomicrobia*	*Bacteria*
Otu1624	0	0.2	0.04	0.49	0.06	74.85	*Planctomyces*	*Planctomycetaceae*	*Planctomycetales*	*Planctomycetacia*	*Planctomycetes*	*Bacteria*
Otu1625	0	0.2	0.04	0.49	0.06	74.91	unclass. *Bacteria*	unclass. *Bacteria*	unclass. *Bacteria*	unclass. *Bacteria*	unclass. *Bacteria*	*Bacteria*
Otu1626	0	0.2	0.04	0.49	0.06	74.98	unclass. *Bacteria*	unclass. *Bacteria*	unclass. *Bacteria*	unclass. *Bacteria*	unclass. *Bacteria*	*Bacteria*
Otu1627	0	0.2	0.04	0.49	0.06	75.04	unclass. *Gammaproteobacteria*	unclass. *Gammaproteobacteria*	unclass. *Gammaproteobacteria*	*Gammaproteobacteria*	*Proteobacteria*	*Bacteria*
Otu1628	0	0.2	0.04	0.49	0.06	75.1	*Lentisphaera*	*Lentisphaeraceae*	*Lentisphaerales*	*Lentisphaeria*	*Lentisphaerae*	*Bacteria*

OTU	Av.A_i (*pH in situ*)	Av.A_i (pH 7.67)	Av.δ_i	Av.δ_i/SD	Av.δ_i %	∑Av.δ_i %	Genus	Family	Order	Class	Phylum	Domain
Otu1629	0	0.2	0.04	0.49	0.06	75.16	unclass. *Flammeovirgaceae*	*Flammeovirgaceae*	*Sphingobacteriales*	*Sphingobacteria*	*Bacteroidetes*	*Bacteria*
Otu1630	0	0.2	0.04	0.49	0.06	75.22	unclass. *Rhodobacteraceae*	*Rhodobacteraceae*	*Rhodobacterales*	*Alphaproteobacteria*	*Proteobacteria*	*Bacteria*
Otu1631	0	0.2	0.04	0.49	0.06	75.28	unclass. *Actinobacteria*	unclass. *Actinobacteria*	unclass. *Actinobacteria*	*Actinobacteria*	*Actinobacteria*	*Bacteria*
Otu1632	0	0.2	0.04	0.49	0.06	75.34	*Reichenbachiella*	*Flammeovirgaceae*	*Sphingobacteriales*	*Sphingobacteria*	*Bacteroidetes*	*Bacteria*
Otu1633	0	0.2	0.04	0.49	0.06	75.4	unclass. *Bacteroidetes*	unclass. *Bacteroidetes*	unclass. *Bacteroidetes*	unclass. *Bacteroidetes*	*Bacteroidetes*	*Bacteria*
Otu1634	0	0.2	0.04	0.49	0.06	75.46	unclass. *Flavobacteriales*	unclass. *Flavobacteriales*	*Flavobacteriales*	*Flavobacteria*	*Bacteroidetes*	*Bacteria*
Otu1635	0	0.2	0.04	0.49	0.06	75.52	unclass. *Bacteria*	unclass. *Bacteria*	unclass. *Bacteria*	unclass. *Bacteria*	unclass. *Bacteria*	*Bacteria*
Otu1636	0	0.2	0.04	0.49	0.06	75.58	unclass. *Flavobacteriales*	unclass. *Flavobacteriales*	*Flavobacteriales*	*Flavobacteria*	*Bacteroidetes*	*Bacteria*
Otu1638	0	0.2	0.04	0.49	0.06	75.65	unclass. *Bacteria*	unclass. *Bacteria*	unclass. *Bacteria*	unclass. *Bacteria*	unclass. *Bacteria*	*Bacteria*
Otu1639	0	0.2	0.04	0.49	0.06	75.71	*Ilumatobacter*	*Acidimicrobidae_incertae_sedis*	*Actinobacteria_order_incertae_ sedis*	*Actinobacteria*	*Actinobacteria*	*Bacteria*
Otu1640	0	0.2	0.04	0.49	0.06	75.77	unclass. *Alphaproteobacteria*	unclass. *Alphaproteobacteria*	unclass. *Alphaproteobacteria*	*Alphaproteobacteria*	*Proteobacteria*	*Bacteria*
Otu1641	0	0.2	0.04	0.49	0.06	75.83	unclass. *Acidobacteria_Gp6*	unclass. *Acidobacteria_Gp6*	unclass. *Acidobacteria_Gp6*	*Acidobacteria_Gp6*	*Acidobacteria*	*Bacteria*
Otu1642	0	0.2	0.04	0.49	0.06	75.89	unclass. *Verrucomicrobiaceae*	*Verrucomicrobiaceae*	*Verrucomicrobiales*	*Verrucomicrobiae*	*Verrucomicrobia*	*Bacteria*
Otu1643	0	0.2	0.04	0.49	0.06	75.95	unclass. *Deltaproteobacteria*	unclass. *Deltaproteobacteria*	unclass. *Deltaproteobacteria*	*Deltaproteobacteria*	*Proteobacteria*	*Bacteria*
Otu1644	0	0.2	0.04	0.49	0.06	76.01	unclass. *Proteobacteria*	unclass. *Proteobacteria*	unclass. *Proteobacteria*	unclass. *Proteobacteria*	*Proteobacteria*	*Bacteria*
Otu1645	0	0.2	0.04	0.49	0.06	76.07	*Thalassomonas*	*Colwelliaceae*	*Alteromonadales*	*Gammaproteobacteria*	*Proteobacteria*	*Bacteria*
Otu1646	0	0.2	0.04	0.49	0.06	76.13	unclass. *Flavobacteriaceae*	*Flavobacteriaceae*	*Flavobacteriales*	*Flavobacteria*	*Bacteroidetes*	*Bacteria*
Otu1647	0	0.2	0.04	0.49	0.06	76.19	unclass. *Desulfobulbaceae*	*Desulfobulbaceae*	*Desulfobacterales*	*Deltaproteobacteria*	*Proteobacteria*	*Bacteria*
Otu1648	0	0.2	0.04	0.49	0.06	76.25	unclass. *Alphaproteobacteria*	unclass. *Alphaproteobacteria*	unclass. *Alphaproteobacteria*	*Alphaproteobacteria*	*Proteobacteria*	*Bacteria*
Otu1649	0	0.2	0.04	0.49	0.06	76.31	unclass. *Bacteroidetes*	unclass. *Bacteroidetes*	unclass. *Bacteroidetes*	unclass. *Bacteroidetes*	*Bacteroidetes*	*Bacteria*
Otu0033	0	0.2	0.04	0.49	0.06	76.38	unclass. *Erythrobacteraceae*	*Erythrobacteraceae*	*Sphingomonadales*	*Alphaproteobacteria*	*Proteobacteria*	*Bacteria*
Otu0132	0	0.2	0.04	0.49	0.06	76.44	unclass. *Alteromonadales*	unclass. *Alteromonadales*	*Alteromonadales*	*Gammaproteobacteria*	*Proteobacteria*	*Bacteria*
Otu0232	0	0.2	0.04	0.49	0.06	76.5	unclass. *Rhodobacteraceae*	*Rhodobacteraceae*	*Rhodobacterales*	*Alphaproteobacteria*	*Proteobacteria*	*Bacteria*
Otu0251	0	0.2	0.04	0.49	0.06	76.56	unclass. *Bacteroidetes*	unclass. *Bacteroidetes*	unclass. *Bacteroidetes*	unclass. *Bacteroidetes*	*Bacteroidetes*	*Bacteria*
Otu0276	0	0.2	0.04	0.49	0.06	76.62	unclass. *Gammaproteobacteria*	unclass. *Gammaproteobacteria*	unclass. *Gammaproteobacteria*	*Gammaproteobacteria*	*Proteobacteria*	*Bacteria*
Otu0334	0	0.2	0.04	0.49	0.06	76.68	unclass. *Flavobacteriaceae*	*Flavobacteriaceae*	*Flavobacteriales*	*Flavobacteria*	*Bacteroidetes*	*Bacteria*
Otu0721	0	0.2	0.04	0.49	0.06	76.74	unclass. *Alphaproteobacteria*	unclass. *Alphaproteobacteria*	unclass. *Alphaproteobacteria*	*Alphaproteobacteria*	*Proteobacteria*	*Bacteria*

OTU	Av.A_i (*pH in situ*)	Av.A_i (pH 7.67)	Av.δ_i	Av.δ_i/SD	Av.δ_i %	∑Av.δ_i %	Genus	Family	Order	Class	Phylum	Domain
Otu0798	0	0.2	0.04	0.49	0.06	76.8	unclass. *Gammaproteobacteria*	unclass. *Gammaproteobacteria*	unclass. *Gammaproteobacteria*	*Gammaproteobacteria*	*Proteobacteria*	*Bacteria*
Otu0818	0	0.2	0.04	0.49	0.06	76.86	unclass. *Planctomycetaceae*	*Planctomycetaceae*	*Planctomycetales*	*Planctomycetacia*	*Planctomycetes*	*Bacteria*
Otu0827	0	0.2	0.04	0.49	0.06	76.92	unclass. *Bacteria*	unclass. *Bacteria*	unclass. *Bacteria*	unclass. *Bacteria*	unclass. *Bacteria*	*Bacteria*
Otu0836	0	0.2	0.04	0.49	0.06	76.98	*Haliea*	*Alteromonadaceae*	*Alteromonadales*	*Gammaproteobacteria*	*Proteobacteria*	*Bacteria*
Otu0847	0	0.2	0.04	0.49	0.06	77.04	unclass. *Alphaproteobacteria*	unclass. *Alphaproteobacteria*	unclass. *Alphaproteobacteria*	*Alphaproteobacteria*	*Proteobacteria*	*Bacteria*
Otu0860	0	0.2	0.04	0.49	0.06	77.1	unclass. *Planctomycetaceae*	*Planctomycetaceae*	*Planctomycetales*	*Planctomycetacia*	*Planctomycetes*	*Bacteria*
Otu1040	0	0.2	0.04	0.49	0.06	77.16	unclass. *Gammaproteobacteria*	unclass. *Gammaproteobacteria*	unclass. *Gammaproteobacteria*	*Gammaproteobacteria*	*Proteobacteria*	*Bacteria*
Otu1044	0	0.2	0.04	0.49	0.06	77.22	unclass. *WS3*	unclass. *WS3*	unclass. *WS3*	unclass. *WS3*	*WS3*	*Bacteria*
Otu1159	0	0.2	0.04	0.49	0.06	77.28	unclass. *Gammaproteobacteria*	unclass. *Gammaproteobacteria*	unclass. *Gammaproteobacteria*	*Gammaproteobacteria*	*Proteobacteria*	*Bacteria*
Otu1179	0	0.2	0.04	0.49	0.06	77.34	unclass. *Planctomycetaceae*	*Planctomycetaceae*	*Planctomycetales*	*Planctomycetacia*	*Planctomycetes*	*Bacteria*
Otu1226	0	0.2	0.04	0.49	0.06	77.4	*Planctomyces*	*Planctomycetaceae*	*Planctomycetales*	*Planctomycetacia*	*Planctomycetes*	*Bacteria*
Otu1402	0	0.2	0.04	0.49	0.06	77.47	unclass. *Rhodobacteraceae*	*Rhodobacteraceae*	*Rhodobacterales*	*Alphaproteobacteria*	*Proteobacteria*	*Bacteria*
Otu1419	0	0.2	0.04	0.49	0.06	77.53	unclass. *Bacteria*	unclass. *Bacteria*	unclass. *Bacteria*	unclass. *Bacteria*	unclass. *Bacteria*	*Bacteria*
Otu1420	0	0.2	0.04	0.49	0.06	77.59	unclass. *Gammaproteobacteria*	unclass. *Gammaproteobacteria*	unclass. *Gammaproteobacteria*	*Gammaproteobacteria*	*Proteobacteria*	*Bacteria*
Otu1423	0	0.2	0.04	0.49	0.06	77.65	*Sphingobium*	*Sphingomonadaceae*	*Sphingomonadales*	*Alphaproteobacteria*	*Proteobacteria*	*Bacteria*
Otu1425	0	0.2	0.04	0.49	0.06	77.71	unclass. *Verrucomicrobiaceae*	*Verrucomicrobiaceae*	*Verrucomicrobiales*	*Verrucomicrobiae*	*Verrucomicrobia*	*Bacteria*
Otu1426	0	0.2	0.04	0.49	0.06	77.77	unclass. *Anaerolineaceae*	*Anaerolineaceae*	*Anaerolineales*	*Anaerolineae*	*Chloroflexi*	*Bacteria*
Otu1427	0	0.2	0.04	0.49	0.06	77.83	unclass. *Gammaproteobacteria*	unclass. *Gammaproteobacteria*	unclass. *Gammaproteobacteria*	*Gammaproteobacteria*	*Proteobacteria*	*Bacteria*
Otu1428	0	0.2	0.04	0.49	0.06	77.89	unclass. *Bacteria*	unclass. *Bacteria*	unclass. *Bacteria*	unclass. *Bacteria*	unclass. *Bacteria*	*Bacteria*
Otu1429	0	0.2	0.04	0.49	0.06	77.95	unclass. *Gammaproteobacteria*	unclass. *Gammaproteobacteria*	unclass. *Gammaproteobacteria*	*Gammaproteobacteria*	*Proteobacteria*	*Bacteria*
Otu1431	0	0.2	0.04	0.49	0.06	78.01	unclass. *Flavobacteriaceae*	*Flavobacteriaceae*	*Flavobacteriales*	*Flavobacteria*	*Bacteroidetes*	*Bacteria*
Otu1434	0	0.2	0.04	0.49	0.06	78.07	unclass. *Proteobacteria*	unclass. *Proteobacteria*	unclass. *Proteobacteria*	unclass. *Proteobacteria*	*Proteobacteria*	*Bacteria*
Otu1436	0	0.2	0.04	0.49	0.06	78.13	unclass. *Gammaproteobacteria*	unclass. *Gammaproteobacteria*	unclass. *Gammaproteobacteria*	*Gammaproteobacteria*	*Proteobacteria*	*Bacteria*
Otu1437	0	0.2	0.04	0.49	0.06	78.19	unclass. *Deltaproteobacteria*	unclass. *Deltaproteobacteria*	unclass. *Deltaproteobacteria*	*Deltaproteobacteria*	*Proteobacteria*	*Bacteria*
Otu1438	0	0.2	0.04	0.49	0.06	78.25	unclass. *Bacteroidetes*	unclass. *Bacteroidetes*	unclass. *Bacteroidetes*	unclass. *Bacteroidetes*	*Bacteroidetes*	*Bacteria*
Otu1440	0	0.2	0.04	0.49	0.06	78.31	unclass. *Actinobacteria*	unclass. *Actinobacteria*	unclass. *Actinobacteria*	*Actinobacteria*	*Actinobacteria*	*Bacteria*
Otu1443	0	0.2	0.04	0.49	0.06	78.37	unclass. *Bacteria*	unclass. *Bacteria*	unclass. *Bacteria*	unclass. *Bacteria*	unclass. *Bacteria*	*Bacteria*

OTU	Av.A_i (*pH in situ*)	Av.A_i (pH 7.67)	Av.δ_i	Av.δ_i/SD	Av.δ_i %	∑Av.δ_i %	Genus	Family	Order	Class	Phylum	Domain
Otu1445	0	0.2	0.04	0.49	0.06	78.43	unclass. *Rhizobiales*	unclass. *Rhizobiales*	*Rhizobiales*	*Alphaproteobacteria*	*Proteobacteria*	*Bacteria*
Otu1446	0	0.2	0.04	0.49	0.06	78.5	unclass. *Flavobacteriaceae*	*Flavobacteriaceae*	*Flavobacteriales*	*Flavobacteria*	*Bacteroidetes*	*Bacteria*
Otu1447	0	0.2	0.04	0.49	0.06	78.56	unclass. *Sphingomonadales*	unclass. *Sphingomonadales*	*Sphingomonadales*	*Alphaproteobacteria*	*Proteobacteria*	*Bacteria*
Otu1448	0	0.2	0.04	0.49	0.06	78.62	unclass. *Bacteria*	unclass. *Bacteria*	unclass. *Bacteria*	unclass. *Bacteria*	unclass. *Bacteria*	*Bacteria*
Otu1450	0	0.2	0.04	0.49	0.06	78.68	unclass. *Bacteria*	unclass. *Bacteria*	unclass. *Bacteria*	unclass. *Bacteria*	unclass. *Bacteria*	*Bacteria*
Otu1452	0	0.2	0.04	0.49	0.06	78.74	*Planctomyces*	*Planctomycetaceae*	*Planctomycetales*	*Planctomycetacia*	*Planctomycetes*	*Bacteria*
Otu1453	0	0.2	0.04	0.49	0.06	78.8	unclass. *Alphaproteobacteria*	unclass. *Alphaproteobacteria*	unclass. *Alphaproteobacteria*	*Alphaproteobacteria*	*Proteobacteria*	*Bacteria*
Otu1454	0	0.2	0.04	0.49	0.06	78.86	unclass. *Bacteroidetes*	unclass. *Bacteroidetes*	unclass. *Bacteroidetes*	unclass. *Bacteroidetes*	*Bacteroidetes*	*Bacteria*
Otu1456	0	0.2	0.04	0.49	0.06	78.92	unclass. *Rhodobacteraceae*	*Rhodobacteraceae*	*Rhodobacterales*	*Alphaproteobacteria*	*Proteobacteria*	*Bacteria*
Otu1457	0	0.2	0.04	0.49	0.06	78.98	unclass. *Proteobacteria*	unclass. *Proteobacteria*	unclass. *Proteobacteria*	unclass. *Proteobacteria*	*Proteobacteria*	*Bacteria*
Otu1458	0	0.2	0.04	0.49	0.06	79.04	unclass. *Gammaproteobacteria*	unclass. *Gammaproteobacteria*	unclass. *Gammaproteobacteria*	*Gammaproteobacteria*	*Proteobacteria*	*Bacteria*
Otu1459	0	0.2	0.04	0.49	0.06	79.1	unclass. *Alphaproteobacteria*	unclass. *Alphaproteobacteria*	unclass. *Alphaproteobacteria*	*Alphaproteobacteria*	*Proteobacteria*	*Bacteria*
Otu1460	0	0.2	0.04	0.49	0.06	79.16	unclass. *Bacteria*	unclass. *Bacteria*	unclass. *Bacteria*	unclass. *Bacteria*	unclass. *Bacteria*	*Bacteria*
Otu1461	0	0.2	0.04	0.49	0.06	79.22	unclass. *Planctomycetaceae*	*Planctomycetaceae*	*Planctomycetales*	*Planctomycetacia*	*Planctomycetes*	*Bacteria*
Otu1462	0	0.2	0.04	0.49	0.06	79.28	unclass. *Proteobacteria*	unclass. *Proteobacteria*	unclass. *Proteobacteria*	unclass. *Proteobacteria*	*Proteobacteria*	*Bacteria*
Otu1463	0	0.2	0.04	0.49	0.06	79.34	unclass. *Bacteria*	unclass. *Bacteria*	unclass. *Bacteria*	unclass. *Bacteria*	unclass. *Bacteria*	*Bacteria*
Otu1464	0	0.2	0.04	0.49	0.06	79.4	unclass. *Gammaproteobacteria*	unclass. *Gammaproteobacteria*	unclass. *Gammaproteobacteria*	*Gammaproteobacteria*	*Proteobacteria*	*Bacteria*
Otu1465	0	0.2	0.04	0.49	0.06	79.46	*Cellulophaga*	*Flavobacteriaceae*	*Flavobacteriales*	*Flavobacteria*	*Bacteroidetes*	*Bacteria*
Otu1467	0	0.2	0.04	0.49	0.06	79.53	unclass. *Bacteria*	unclass. *Bacteria*	unclass. *Bacteria*	unclass. *Bacteria*	unclass. *Bacteria*	*Bacteria*
Otu1468	0	0.2	0.04	0.49	0.06	79.59	unclass. *Bacteria*	unclass. *Bacteria*	unclass. *Bacteria*	unclass. *Bacteria*	unclass. *Bacteria*	*Bacteria*
Otu1469	0	0.2	0.04	0.49	0.06	79.65	unclass. *Rhodospirillaceae*	*Rhodospirillaceae*	*Rhodospirillales*	*Alphaproteobacteria*	*Proteobacteria*	*Bacteria*
Otu1471	0	0.2	0.04	0.49	0.06	79.71	unclass. *Bacteria*	unclass. *Bacteria*	unclass. *Bacteria*	unclass. *Bacteria*	unclass. *Bacteria*	*Bacteria*
Otu1472	0	0.2	0.04	0.49	0.06	79.77	unclass. *Planctomycetaceae*	*Planctomycetaceae*	*Planctomycetales*	*Planctomycetacia*	*Planctomycetes*	*Bacteria*
Otu1473	0	0.2	0.04	0.49	0.06	79.83	unclass. *Rhodobacteraceae*	*Rhodobacteraceae*	*Rhodobacterales*	*Alphaproteobacteria*	*Proteobacteria*	*Bacteria*
Otu1474	0	0.2	0.04	0.49	0.06	79.89	unclass. *Verrucomicrobia*	unclass. *Verrucomicrobia*	unclass. *Verrucomicrobia*	unclass. *Verrucomicrobia*	*Verrucomicrobia*	*Bacteria*
Otu1475	0	0.2	0.04	0.49	0.06	79.95	unclass. *Bacteria*	unclass. *Bacteria*	unclass. *Bacteria*	unclass. *Bacteria*	unclass. *Bacteria*	*Bacteria*
Otu1477	0	0.2	0.04	0.49	0.06	80.01	unclass. *Legionellaceae*	*Legionellaceae*	*Legionellales*	*Gammaproteobacteria*	*Proteobacteria*	*Bacteria*

OTU	Av.A_i (*pH in situ*)	Av.A_i (pH 7.67)	Av.δ_i	Av.δ_i/SD	Av.δ_i %	∑Av.δ_i %	Genus	Family	Order	Class	Phylum	Domain
Otu1478	0	0.2	0.04	0.49	0.06	80.07	unclass. *Bacteria*	unclass. *Bacteria*	unclass. *Bacteria*	unclass. *Bacteria*	unclass. *Bacteria*	*Bacteria*
Otu1480	0	0.2	0.04	0.49	0.06	80.13	unclass. *Flavobacteriaceae*	*Flavobacteriaceae*	*Flavobacteriales*	*Flavobacteria*	*Bacteroidetes*	*Bacteria*
Otu1484	0	0.2	0.04	0.49	0.06	80.19	unclass. *Deltaproteobacteria*	unclass. *Deltaproteobacteria*	unclass. *Deltaproteobacteria*	*Deltaproteobacteria*	*Proteobacteria*	*Bacteria*
Otu0061	0	0.2	0.04	0.49	0.06	80.25	unclass. *Actinomycetales*	unclass. *Actinomycetales*	*Actinomycetales*	*Actinobacteria*	*Actinobacteria*	*Bacteria*
Otu0201	0	0.2	0.04	0.49	0.06	80.31	unclass. *Alphaproteobacteria*	unclass. *Alphaproteobacteria*	unclass. *Alphaproteobacteria*	*Alphaproteobacteria*	*Proteobacteria*	*Bacteria*
Otu0240	0	0.2	0.04	0.49	0.06	80.37	unclass. *Chromatiales*	unclass. *Chromatiales*	*Chromatiales*	*Gammaproteobacteria*	*Proteobacteria*	*Bacteria*
Otu0245	0	0.2	0.04	0.49	0.06	80.43	unclass. *Flavobacteriales*	unclass. *Flavobacteriales*	*Flavobacteriales*	*Flavobacteria*	*Bacteroidetes*	*Bacteria*
Otu0260	0	0.2	0.04	0.49	0.06	80.49	unclass. *Gammaproteobacteria*	unclass. *Gammaproteobacteria*	unclass. *Gammaproteobacteria*	*Gammaproteobacteria*	*Proteobacteria*	*Bacteria*
Otu0357	0	0.2	0.04	0.49	0.06	80.55	*Sphingopyxis*	*Sphingomonadaceae*	*Sphingomonadales*	*Alphaproteobacteria*	*Proteobacteria*	*Bacteria*
Otu0413	0	0.2	0.04	0.49	0.06	80.61	*Eudoraea*	*Flavobacteriaceae*	*Flavobacteriales*	*Flavobacteria*	*Bacteroidetes*	*Bacteria*
Otu0462	0	0.2	0.04	0.49	0.06	80.67	*Haliea*	*Alteromonadaceae*	*Alteromonadales*	*Gammaproteobacteria*	*Proteobacteria*	*Bacteria*
Otu0618	0	0.2	0.04	0.49	0.06	80.73	*Glaciecola*	*Alteromonadaceae*	*Alteromonadales*	*Gammaproteobacteria*	*Proteobacteria*	*Bacteria*
Otu0769	0	0.2	0.04	0.49	0.06	80.79	unclass. *Bacteroidetes*	unclass. *Bacteroidetes*	unclass. *Bacteroidetes*	unclass. *Bacteroidetes*	*Bacteroidetes*	*Bacteria*
Otu0866	0	0.2	0.04	0.49	0.06	80.85	*Thalassomonas*	*Colwelliaceae*	*Alteromonadales*	*Gammaproteobacteria*	*Proteobacteria*	*Bacteria*
Otu0930	0	0.2	0.04	0.49	0.06	80.91	unclass. *Rickettsiaceae*	*Rickettsiaceae*	*Rickettsiales*	*Alphaproteobacteria*	*Proteobacteria*	*Bacteria*
Otu1060	0	0.2	0.04	0.49	0.06	80.97	unclass. *Rhodobacteraceae*	*Rhodobacteraceae*	*Rhodobacterales*	*Alphaproteobacteria*	*Proteobacteria*	*Bacteria*
Otu1067	0	0.2	0.04	0.49	0.06	81.03	unclass. *Rhodobacteraceae*	*Rhodobacteraceae*	*Rhodobacterales*	*Alphaproteobacteria*	*Proteobacteria*	*Bacteria*
Otu1123	0	0.2	0.04	0.49	0.06	81.09	unclass. *Proteobacteria*	unclass. *Proteobacteria*	unclass. *Proteobacteria*	unclass. *Proteobacteria*	*Proteobacteria*	*Bacteria*
Otu1255	0	0.2	0.04	0.49	0.06	81.15	unclass. *Bacteria*	unclass. *Bacteria*	unclass. *Bacteria*	unclass. *Bacteria*	unclass. *Bacteria*	*Bacteria*
Otu1297	0	0.2	0.04	0.49	0.06	81.21	unclass. *Alphaproteobacteria*	unclass. *Alphaproteobacteria*	unclass. *Alphaproteobacteria*	*Alphaproteobacteria*	*Proteobacteria*	*Bacteria*
Otu1479	0	0.2	0.04	0.49	0.06	81.27	unclass. *Gammaproteobacteria*	unclass. *Gammaproteobacteria*	unclass. *Gammaproteobacteria*	*Gammaproteobacteria*	*Proteobacteria*	*Bacteria*
Otu1531	0	0.2	0.04	0.49	0.06	81.33	unclass. *Nannocystaceae*	*Nannocystaceae*	*Myxococcales*	*Deltaproteobacteria*	*Proteobacteria*	*Bacteria*
Otu1539	0	0.2	0.04	0.49	0.06	81.39	unclass. *Proteobacteria*	unclass. *Proteobacteria*	unclass. *Proteobacteria*	unclass. *Proteobacteria*	*Proteobacteria*	*Bacteria*
Otu1605	0	0.2	0.04	0.49	0.06	81.45	unclass. *Gammaproteobacteria*	unclass. *Gammaproteobacteria*	unclass. *Gammaproteobacteria*	*Gammaproteobacteria*	*Proteobacteria*	*Bacteria*
Otu1650	0	0.2	0.04	0.49	0.06	81.51	unclass. *Alphaproteobacteria*	unclass. *Alphaproteobacteria*	unclass. *Alphaproteobacteria*	*Alphaproteobacteria*	*Proteobacteria*	*Bacteria*
Otu1654	0	0.2	0.04	0.49	0.06	81.57	unclass. *Proteobacteria*	unclass. *Proteobacteria*	unclass. *Proteobacteria*	unclass. *Proteobacteria*	*Proteobacteria*	*Bacteria*
Otu1655	0	0.2	0.04	0.49	0.06	81.63	unclass. *Bacteria*	unclass. *Bacteria*	unclass. *Bacteria*	unclass. *Bacteria*	unclass. *Bacteria*	*Bacteria*

OTU	Av.A_i (*pH in situ*)	Av.A_i (pH 7.67)	Av.δ_i	Av.δ_i/SD	Av.δ_i %	∑Av.δ_i %	Genus	Family	Order	Class	Phylum	Domain
Otu1656	0	0.2	0.04	0.49	0.06	81.69	unclass. *Alphaproteobacteria*	unclass. *Alphaproteobacteria*	unclass. *Alphaproteobacteria*	*Alphaproteobacteria*	*Proteobacteria*	*Bacteria*
Otu1657	0	0.2	0.04	0.49	0.06	81.75	unclass. *Bacteroidetes*	unclass. *Bacteroidetes*	unclass. *Bacteroidetes*	unclass. *Bacteroidetes*	*Bacteroidetes*	*Bacteria*
Otu1658	0	0.2	0.04	0.49	0.06	81.81	unclass. *Bacteria*	unclass. *Bacteria*	unclass. *Bacteria*	unclass. *Bacteria*	unclass. *Bacteria*	*Bacteria*
Otu1660	0	0.2	0.04	0.49	0.06	81.87	unclass. *Bacteria*	unclass. *Bacteria*	unclass. *Bacteria*	unclass. *Bacteria*	unclass. *Bacteria*	*Bacteria*
Otu1661	0	0.2	0.04	0.49	0.06	81.93	unclass. *Gammaproteobacteria*	unclass. *Gammaproteobacteria*	unclass. *Gammaproteobacteria*	*Gammaproteobacteria*	*Proteobacteria*	*Bacteria*
Otu1662	0	0.2	0.04	0.49	0.06	81.99	unclass. *Oceanospirillaceae*	*Oceanospirillaceae*	*Oceanospirillales*	*Gammaproteobacteria*	*Proteobacteria*	*Bacteria*
Otu1663	0	0.2	0.04	0.49	0.06	82.05	unclass. *Proteobacteria*	unclass. *Proteobacteria*	unclass. *Proteobacteria*	unclass. *Proteobacteria*	*Proteobacteria*	*Bacteria*
Otu1664	0	0.2	0.04	0.49	0.06	82.11	unclass. *Bacteroidetes*	unclass. *Bacteroidetes*	unclass. *Bacteroidetes*	unclass. *Bacteroidetes*	*Bacteroidetes*	*Bacteria*
Otu1665	0	0.2	0.04	0.49	0.06	82.17	unclass. *Bacteroidetes*	unclass. *Bacteroidetes*	unclass. *Bacteroidetes*	unclass. *Bacteroidetes*	*Bacteroidetes*	*Bacteria*
Otu1666	0	0.2	0.04	0.49	0.06	82.23	unclass. *Bacteria*	unclass. *Bacteria*	unclass. *Bacteria*	unclass. *Bacteria*	unclass. *Bacteria*	*Bacteria*
Otu1667	0	0.2	0.04	0.49	0.06	82.29	unclass. *Bacteria*	unclass. *Bacteria*	unclass. *Bacteria*	unclass. *Bacteria*	unclass. *Bacteria*	*Bacteria*
Otu1668	0	0.2	0.04	0.49	0.06	82.35	unclass. *Bacteria*	unclass. *Bacteria*	unclass. *Bacteria*	unclass. *Bacteria*	unclass. *Bacteria*	*Bacteria*
Otu1669	0	0.2	0.04	0.49	0.06	82.41	unclass. *Myxococcales*	unclass. *Myxococcales*	*Myxococcales*	*Deltaproteobacteria*	*Proteobacteria*	*Bacteria*
Otu1670	0	0.2	0.04	0.49	0.06	82.47	unclass. *Actinobacteria*	unclass. *Actinobacteria*	unclass. *Actinobacteria*	*Actinobacteria*	*Actinobacteria*	*Bacteria*
Otu1671	0	0.2	0.04	0.49	0.06	82.53	unclass. *Bacteria*	unclass. *Bacteria*	unclass. *Bacteria*	unclass. *Bacteria*	unclass. *Bacteria*	*Bacteria*
Otu1672	0	0.2	0.04	0.49	0.06	82.59	unclass. *Bacteria*	unclass. *Bacteria*	unclass. *Bacteria*	unclass. *Bacteria*	unclass. *Bacteria*	*Bacteria*
Otu1673	0	0.2	0.04	0.49	0.06	82.65	unclass. *Rickettsiaceae*	*Rickettsiaceae*	*Rickettsiales*	*Alphaproteobacteria*	*Proteobacteria*	*Bacteria*
Otu1674	0	0.2	0.04	0.49	0.06	82.71	unclass. *Bacteria*	unclass. *Bacteria*	unclass. *Bacteria*	unclass. *Bacteria*	unclass. *Bacteria*	*Bacteria*
Otu1675	0	0.2	0.04	0.49	0.06	82.77	unclass. *Bacteria*	unclass. *Bacteria*	unclass. *Bacteria*	unclass. *Bacteria*	unclass. *Bacteria*	*Bacteria*
Otu1676	0	0.2	0.04	0.49	0.06	82.83	unclass. *Alphaproteobacteria*	unclass. *Alphaproteobacteria*	unclass. *Alphaproteobacteria*	*Alphaproteobacteria*	*Proteobacteria*	*Bacteria*
Otu1677	0	0.2	0.04	0.49	0.06	82.89	unclass. *Bacteria*	unclass. *Bacteria*	unclass. *Bacteria*	unclass. *Bacteria*	unclass. *Bacteria*	*Bacteria*
Otu1678	0	0.2	0.04	0.49	0.06	82.95	unclass. *Bacteria*	unclass. *Bacteria*	unclass. *Bacteria*	unclass. *Bacteria*	unclass. *Bacteria*	*Bacteria*
Otu1679	0	0.2	0.04	0.49	0.06	83.01	*Actibacter*	*Flavobacteriaceae*	*Flavobacteriales*	*Flavobacteria*	*Bacteroidetes*	*Bacteria*
Otu1680	0	0.2	0.04	0.49	0.06	83.07	*Aquicella*	*Coxiellaceae*	*Legionellales*	*Gammaproteobacteria*	*Proteobacteria*	*Bacteria*
Otu1681	0	0.2	0.04	0.49	0.06	83.13	unclass. *Rhodobacteraceae*	*Rhodobacteraceae*	*Rhodobacterales*	*Alphaproteobacteria*	*Proteobacteria*	*Bacteria*
Otu1682	0	0.2	0.04	0.49	0.06	83.19	*Nitriliruptor*	*Nitriliruptoraceae*	*Nitriliruptorales*	*Actinobacteria*	*Actinobacteria*	*Bacteria*
Otu1683	0	0.2	0.04	0.49	0.06	83.25	unclass. *Gammaproteobacteria*	unclass. *Gammaproteobacteria*	unclass. *Gammaproteobacteria*	*Gammaproteobacteria*	*Proteobacteria*	*Bacteria*

OTU	Av.A_i (*pH in situ*)	Av.A_i (pH 7.67)	Av.δ_i	Av.δ_i/SD	Av.δ_i %	∑Av.δ_i %	Genus	Family	Order	Class	Phylum	Domain
Otu1684	0	0.2	0.04	0.49	0.06	83.31	unclass. *Gammaproteobacteria*	unclass. *Gammaproteobacteria*	unclass. *Gammaproteobacteria*	*Gammaproteobacteria*	*Proteobacteria*	*Bacteria*
Otu1685	0	0.2	0.04	0.49	0.06	83.37	unclass. *Bacteria*	unclass. *Bacteria*	unclass. *Bacteria*	unclass. *Bacteria*	unclass. *Bacteria*	*Bacteria*
Otu1686	0	0.2	0.04	0.49	0.06	83.43	unclass. *Bacteria*	unclass. *Bacteria*	unclass. *Bacteria*	unclass. *Bacteria*	unclass. *Bacteria*	*Bacteria*
Otu1688	0	0.2	0.04	0.49	0.06	83.49	unclass. *Saprospiraceae*	*Saprospiraceae*	*Sphingobacteriales*	*Sphingobacteria*	*Bacteroidetes*	*Bacteria*
Otu1689	0	0.2	0.04	0.49	0.06	83.55	unclass. *Gammaproteobacteria*	unclass. *Gammaproteobacteria*	unclass. *Gammaproteobacteria*	*Gammaproteobacteria*	*Proteobacteria*	*Bacteria*
Otu1690	0	0.2	0.04	0.49	0.06	83.61	unclass. *Bacteria*	unclass. *Bacteria*	unclass. *Bacteria*	unclass. *Bacteria*	unclass. *Bacteria*	*Bacteria*
Otu0369	0	0.2	0.04	0.49	0.06	83.67	unclass. *Deltaproteobacteria*	unclass. *Deltaproteobacteria*	unclass. *Deltaproteobacteria*	*Deltaproteobacteria*	*Proteobacteria*	*Bacteria*
Otu0412	0	0.2	0.04	0.49	0.06	83.73	unclass. *Bacteroidetes*	unclass. *Bacteroidetes*	unclass. *Bacteroidetes*	unclass. *Bacteroidetes*	*Bacteroidetes*	*Bacteria*
Otu0424	0	0.2	0.04	0.49	0.06	83.79	unclass. *Flavobacteriales*	unclass. *Flavobacteriales*	*Flavobacteriales*	*Flavobacteria*	*Bacteroidetes*	*Bacteria*
Otu0448	0	0.2	0.04	0.49	0.06	83.85	unclass. *Desulfuromonadaceae*	*Desulfuromonadaceae*	*Desulfuromonadales*	*Deltaproteobacteria*	*Proteobacteria*	*Bacteria*
Otu0551	0	0.2	0.04	0.49	0.06	83.91	*Arcobacter*	*Campylobacteraceae*	*Campylobacterales*	*Epsilonproteobacteria*	*Proteobacteria*	*Bacteria*
Otu0620	0	0.2	0.04	0.49	0.06	83.97	unclass. *Nannocystaceae*	*Nannocystaceae*	*Myxococcales*	*Deltaproteobacteria*	*Proteobacteria*	*Bacteria*
Otu0887	0	0.2	0.04	0.49	0.06	84.03	*Winogradskyella*	*Flavobacteriaceae*	*Flavobacteriales*	*Flavobacteria*	*Bacteroidetes*	*Bacteria*
Otu1149	0	0.2	0.04	0.49	0.06	84.09	unclass. *Bacteria*	unclass. *Bacteria*	unclass. *Bacteria*	unclass. *Bacteria*	unclass. *Bacteria*	*Bacteria*
Otu1196	0	0.2	0.04	0.49	0.06	84.15	unclass. *Bacteria*	unclass. *Bacteria*	unclass. *Bacteria*	unclass. *Bacteria*	unclass. *Bacteria*	*Bacteria*
Otu1212	0	0.2	0.04	0.49	0.06	84.21	unclass. *Bacteria*	unclass. *Bacteria*	unclass. *Bacteria*	unclass. *Bacteria*	unclass. *Bacteria*	*Bacteria*
Otu1242	0	0.2	0.04	0.49	0.06	84.27	unclass. *Bacteria*	unclass. *Bacteria*	unclass. *Bacteria*	unclass. *Bacteria*	unclass. *Bacteria*	*Bacteria*
Otu1354	0	0.2	0.04	0.49	0.06	84.33	unclass. *Gammaproteobacteria*	unclass. *Gammaproteobacteria*	unclass. *Gammaproteobacteria*	*Gammaproteobacteria*	*Proteobacteria*	*Bacteria*
Otu1395	0	0.2	0.04	0.49	0.06	84.39	unclass. *Bacteria*	unclass. *Bacteria*	unclass. *Bacteria*	unclass. *Bacteria*	unclass. *Bacteria*	*Bacteria*
Otu1424	0	0.2	0.04	0.49	0.06	84.45	unclass. *Gammaproteobacteria*	unclass. *Gammaproteobacteria*	unclass. *Gammaproteobacteria*	*Gammaproteobacteria*	*Proteobacteria*	*Bacteria*
Otu1504	0	0.2	0.04	0.49	0.06	84.51	unclass. *Proteobacteria*	unclass. *Proteobacteria*	unclass. *Proteobacteria*	unclass. *Proteobacteria*	*Proteobacteria*	*Bacteria*
Otu1551	0	0.2	0.04	0.49	0.06	84.57	unclass. *Gammaproteobacteria*	unclass. *Gammaproteobacteria*	unclass. *Gammaproteobacteria*	*Gammaproteobacteria*	*Proteobacteria*	*Bacteria*
Otu1553	0	0.2	0.04	0.49	0.06	84.63	unclass. *Flavobacteriales*	unclass. *Flavobacteriales*	*Flavobacteriales*	*Flavobacteria*	*Bacteroidetes*	*Bacteria*
Otu1555	0	0.2	0.04	0.49	0.06	84.69	unclass. *Gammaproteobacteria*	unclass. *Gammaproteobacteria*	unclass. *Gammaproteobacteria*	*Gammaproteobacteria*	*Proteobacteria*	*Bacteria*
Otu1558	0	0.2	0.04	0.49	0.06	84.75	unclass. *Flavobacteriales*	unclass. *Flavobacteriales*	*Flavobacteriales*	*Flavobacteria*	*Bacteroidetes*	*Bacteria*
Otu1559	0	0.2	0.04	0.49	0.06	84.81	unclass. *Gammaproteobacteria*	unclass. *Gammaproteobacteria*	unclass. *Gammaproteobacteria*	*Gammaproteobacteria*	*Proteobacteria*	*Bacteria*
Otu1560	0	0.2	0.04	0.49	0.06	84.87	*Gaetbulibacter*	*Flavobacteriaceae*	*Flavobacteriales*	*Flavobacteria*	*Bacteroidetes*	*Bacteria*

OTU	Av.A_i (*pH in situ*)	Av.A_i (pH 7.67)	Av.δ_i	Av.δ_i/SD	Av.δ_i %	∑Av.δ_i %	Genus	Family	Order	Class	Phylum	Domain
Otu1561	0	0.2	0.04	0.49	0.06	84.93	unclass. *Acidobacteria_Gp22*	unclass. *Acidobacteria_Gp22*	unclass. *Acidobacteria_Gp22*	*Acidobacteria_Gp22*	*Acidobacteria*	*Bacteria*
Otu1562	0	0.2	0.04	0.49	0.06	84.99	unclass. *Desulfobacteraceae*	*Desulfobacteraceae*	*Desulfobacterales*	*Deltaproteobacteria*	*Proteobacteria*	*Bacteria*
Otu1564	0	0.2	0.04	0.49	0.06	85.05	unclass. *Colwelliaceae*	*Colwelliaceae*	*Alteromonadales*	*Gammaproteobacteria*	*Proteobacteria*	*Bacteria*
Otu1567	0	0.2	0.04	0.49	0.06	85.11	unclass. *Bacteroidetes*	unclass. *Bacteroidetes*	unclass. *Bacteroidetes*	unclass. *Bacteroidetes*	*Bacteroidetes*	*Bacteria*
Otu1568	0	0.2	0.04	0.49	0.06	85.17	unclass. *Bacteroidetes*	unclass. *Bacteroidetes*	unclass. *Bacteroidetes*	unclass. *Bacteroidetes*	*Bacteroidetes*	*Bacteria*
Otu1569	0	0.2	0.04	0.49	0.06	85.23	unclass. *Flavobacteriales*	unclass. *Flavobacteriales*	*Flavobacteriales*	*Flavobacteria*	*Bacteroidetes*	*Bacteria*
Otu1570	0	0.2	0.04	0.49	0.06	85.29	unclass. *Bacteria*	unclass. *Bacteria*	unclass. *Bacteria*	unclass. *Bacteria*	unclass. *Bacteria*	*Bacteria*
Otu1571	0	0.2	0.04	0.49	0.06	85.35	unclass. *Bacteroidetes*	unclass. *Bacteroidetes*	unclass. *Bacteroidetes*	unclass. *Bacteroidetes*	*Bacteroidetes*	*Bacteria*
Otu1572	0	0.2	0.04	0.49	0.06	85.41	unclass. *Bacteroidetes*	unclass. *Bacteroidetes*	unclass. *Bacteroidetes*	unclass. *Bacteroidetes*	*Bacteroidetes*	*Bacteria*
Otu1573	0	0.2	0.04	0.49	0.06	85.47	unclass. *Flammeovirgaceae*	*Flammeovirgaceae*	*Sphingobacteriales*	*Sphingobacteria*	*Bacteroidetes*	*Bacteria*
Otu1574	0	0.2	0.04	0.49	0.06	85.53	unclass. *Bacteria*	unclass. *Bacteria*	unclass. *Bacteria*	unclass. *Bacteria*	unclass. *Bacteria*	*Bacteria*
Otu1575	0	0.2	0.04	0.49	0.06	85.59	unclass. *Bacteria*	unclass. *Bacteria*	unclass. *Bacteria*	unclass. *Bacteria*	unclass. *Bacteria*	*Bacteria*
Otu1576	0	0.2	0.04	0.49	0.06	85.65	unclass. *Deltaproteobacteria*	unclass. *Deltaproteobacteria*	unclass. *Deltaproteobacteria*	*Deltaproteobacteria*	*Proteobacteria*	*Bacteria*
Otu1579	0	0.2	0.04	0.49	0.06	85.71	unclass. *Bacteroidetes*	unclass. *Bacteroidetes*	unclass. *Bacteroidetes*	unclass. *Bacteroidetes*	*Bacteroidetes*	*Bacteria*
Otu1580	0	0.2	0.04	0.49	0.06	85.77	*Leucothrix*	*Thiotrichaceae*	*Thiotrichales*	*Gammaproteobacteria*	*Proteobacteria*	*Bacteria*
Otu1582	0	0.2	0.04	0.49	0.06	85.83	unclass. *Bacteria*	unclass. *Bacteria*	unclass. *Bacteria*	unclass. *Bacteria*	unclass. *Bacteria*	*Bacteria*
Otu1584	0	0.2	0.04	0.49	0.06	85.89	unclass. *Bacteria*	unclass. *Bacteria*	unclass. *Bacteria*	unclass. *Bacteria*	unclass. *Bacteria*	*Bacteria*
Otu1585	0	0.2	0.04	0.49	0.06	85.95	unclass. *Rhodobacteraceae*	*Rhodobacteraceae*	*Rhodobacterales*	*Alphaproteobacteria*	*Proteobacteria*	*Bacteria*
Otu1586	0	0.2	0.04	0.49	0.06	86.01	unclass. *Bacteria*	unclass. *Bacteria*	unclass. *Bacteria*	unclass. *Bacteria*	unclass. *Bacteria*	*Bacteria*
Otu1587	0	0.2	0.04	0.49	0.06	86.07	unclass. *Bacteria*	unclass. *Bacteria*	unclass. *Bacteria*	unclass. *Bacteria*	unclass. *Bacteria*	*Bacteria*
Otu1588	0	0.2	0.04	0.49	0.06	86.13	unclass. *Gammaproteobacteria*	unclass. *Gammaproteobacteria*	unclass. *Gammaproteobacteria*	*Gammaproteobacteria*	*Proteobacteria*	*Bacteria*
Otu1589	0	0.2	0.04	0.49	0.06	86.19	unclass. *Rhodobacteraceae*	*Rhodobacteraceae*	*Rhodobacterales*	*Alphaproteobacteria*	*Proteobacteria*	*Bacteria*
Otu1590	0	0.2	0.04	0.49	0.06	86.25	unclass. *Flavobacteriales*	unclass. *Flavobacteriales*	*Flavobacteriales*	*Flavobacteria*	*Bacteroidetes*	*Bacteria*
Otu1591	0	0.2	0.04	0.49	0.06	86.31	unclass. *Sphingobacteriales*	unclass. *Sphingobacteriales*	*Sphingobacteriales*	*Sphingobacteria*	*Bacteroidetes*	*Bacteria*
Otu1592	0	0.2	0.04	0.49	0.06	86.37	unclass. *Bacteroidetes*	unclass. *Bacteroidetes*	unclass. *Bacteroidetes*	unclass. *Bacteroidetes*	*Bacteroidetes*	*Bacteria*
Otu1593	0	0.2	0.04	0.49	0.06	86.43	*Planctomyces*	*Planctomycetaceae*	*Planctomycetales*	*Planctomycetacia*	*Planctomycetes*	*Bacteria*
Otu1594	0	0.2	0.04	0.49	0.06	86.49	unclass. *Gammaproteobacteria*	unclass. *Gammaproteobacteria*	unclass. *Gammaproteobacteria*	*Gammaproteobacteria*	*Proteobacteria*	*Bacteria*

OTU	Av.A_i (*pH in situ*)	Av.A_i (pH 7.67)	Av.δ_i	Av.δ_i/SD	Av.δ_i %	∑Av.δ_i %	Genus	Family	Order	Class	Phylum	Domain
Otu1595	0	0.2	0.04	0.49	0.06	86.55	unclass. *Bacteria*	unclass. *Bacteria*	unclass. *Bacteria*	unclass. *Bacteria*	unclass. *Bacteria*	*Bacteria*
Otu1596	0	0.2	0.04	0.49	0.06	86.61	unclass. *Bacteroidetes*	unclass. *Bacteroidetes*	unclass. *Bacteroidetes*	unclass. *Bacteroidetes*	*Bacteroidetes*	*Bacteria*
Otu1597	0	0.2	0.04	0.49	0.06	86.67	*Methylophaga*	*Piscirickettsiaceae*	*Thiotrichales*	*Gammaproteobacteria*	*Proteobacteria*	*Bacteria*
Otu1598	0	0.2	0.04	0.49	0.06	86.73	unclass. *Bacteria*	unclass. *Bacteria*	unclass. *Bacteria*	unclass. *Bacteria*	unclass. *Bacteria*	*Bacteria*
Otu1600	0	0.2	0.04	0.49	0.06	86.79	unclass. *Bacteroidetes*	unclass. *Bacteroidetes*	unclass. *Bacteroidetes*	unclass. *Bacteroidetes*	*Bacteroidetes*	*Bacteria*
Otu1601	0	0.2	0.04	0.49	0.06	86.85	unclass. *Bacteria*	unclass. *Bacteria*	unclass. *Bacteria*	unclass. *Bacteria*	unclass. *Bacteria*	*Bacteria*
Otu1602	0	0.2	0.04	0.49	0.06	86.91	unclass. *Bacteroidetes*	unclass. *Bacteroidetes*	unclass. *Bacteroidetes*	unclass. *Bacteroidetes*	*Bacteroidetes*	*Bacteria*
Otu0041	0.2	0	0.04	0.49	0.06	86.97	*Sulfitobacter*	*Rhodobacteraceae*	*Rhodobacterales*	*Alphaproteobacteria*	*Proteobacteria*	*Bacteria*
Otu0077	0.2	0	0.04	0.49	0.06	87.03	unclass. *Bacteria*	unclass. *Bacteria*	unclass. *Bacteria*	unclass. *Bacteria*	unclass. *Bacteria*	*Bacteria*
Otu0078	0.2	0	0.04	0.49	0.06	87.08	*Erythrobacter*	*Erythrobacteraceae*	*Sphingomonadales*	*Alphaproteobacteria*	*Proteobacteria*	*Bacteria*
Otu0196	0.2	0	0.04	0.49	0.06	87.14	unclass. *Flavobacteriaceae*	*Flavobacteriaceae*	*Flavobacteriales*	*Flavobacteria*	*Bacteroidetes*	*Bacteria*
Otu0239	0.2	0	0.04	0.49	0.06	87.2	unclass. *Gammaproteobacteria*	unclass. *Gammaproteobacteria*	unclass. *Gammaproteobacteria*	*Gammaproteobacteria*	*Proteobacteria*	*Bacteria*
Otu0297	0.2	0	0.04	0.49	0.06	87.26	*Lutibacter*	*Flavobacteriaceae*	*Flavobacteriales*	*Flavobacteria*	*Bacteroidetes*	*Bacteria*
Otu0304	0.2	0	0.04	0.49	0.06	87.32	unclass. *Bacteria*	unclass. *Bacteria*	unclass. *Bacteria*	unclass. *Bacteria*	unclass. *Bacteria*	*Bacteria*
Otu0332	0.2	0	0.04	0.49	0.06	87.38	unclass. *Gammaproteobacteria*	unclass. *Gammaproteobacteria*	unclass. *Gammaproteobacteria*	*Gammaproteobacteria*	*Proteobacteria*	*Bacteria*
Otu0342	0.2	0	0.04	0.49	0.06	87.44	unclass. *Gammaproteobacteria*	unclass. *Gammaproteobacteria*	unclass. *Gammaproteobacteria*	*Gammaproteobacteria*	*Proteobacteria*	*Bacteria*
Otu0403	0.2	0	0.04	0.49	0.06	87.5	unclass. *Acidobacteria_Gp10*	unclass. *Acidobacteria_Gp10*	unclass. *Acidobacteria_Gp10*	*Acidobacteria_Gp10*	*Acidobacteria*	*Bacteria*
Otu0431	0.2	0	0.04	0.49	0.06	87.56	unclass. *Flammeovirgaceae*	*Flammeovirgaceae*	*Sphingobacteriales*	*Sphingobacteria*	*Bacteroidetes*	*Bacteria*
Otu0491	0.2	0	0.04	0.49	0.06	87.62	unclass. *Flavobacteriales*	unclass. *Flavobacteriales*	*Flavobacteriales*	*Flavobacteria*	*Bacteroidetes*	*Bacteria*
Otu0582	0.2	0	0.04	0.49	0.06	87.68	unclass. *Rhodobacteraceae*	*Rhodobacteraceae*	*Rhodobacterales*	*Alphaproteobacteria*	*Proteobacteria*	*Bacteria*
Otu0795	0.2	0	0.04	0.49	0.06	87.74	unclass. *Bacteria*	unclass. *Bacteria*	unclass. *Bacteria*	unclass. *Bacteria*	unclass. *Bacteria*	*Bacteria*
Otu0855	0.2	0	0.04	0.49	0.06	87.8	*Dasania*	*Pseudomonadales_incertae_sedis*	*Pseudomonadales*	*Gammaproteobacteria*	*Proteobacteria*	*Bacteria*
Otu0936	0.2	0	0.04	0.49	0.06	87.86	unclass. *Gammaproteobacteria*	unclass. *Gammaproteobacteria*	unclass. *Gammaproteobacteria*	*Gammaproteobacteria*	*Proteobacteria*	*Bacteria*
Otu0951	0.2	0	0.04	0.49	0.06	87.92	*Haliea*	*Alteromonadaceae*	*Alteromonadales*	*Gammaproteobacteria*	*Proteobacteria*	*Bacteria*
Otu1022	0.2	0	0.04	0.49	0.06	87.98	unclass. *Gammaproteobacteria*	unclass. *Gammaproteobacteria*	unclass. *Gammaproteobacteria*	*Gammaproteobacteria*	*Proteobacteria*	*Bacteria*
Otu1163	0.2	0	0.04	0.49	0.06	88.04	unclass. *Bacteria*	unclass. *Bacteria*	unclass. *Bacteria*	unclass. *Bacteria*	unclass. *Bacteria*	*Bacteria*
Otu1177	0.2	0	0.04	0.49	0.06	88.1	unclass. *Erythrobacteraceae*	*Erythrobacteraceae*	*Sphingomonadales*	*Alphaproteobacteria*	*Proteobacteria*	*Bacteria*

OTU	Av.A_i (*pH in situ*)	Av.A_i (pH 7.67)	Av.δ_i	Av.δ_i/SD	Av.δ_i %	∑Av.δ_i %	Genus	Family	Order	Class	Phylum	Domain
Otu1190	0.2	0	0.04	0.49	0.06	88.16	unclass. *Gammaproteobacteria*	unclass. *Gammaproteobacteria*	unclass. *Gammaproteobacteria*	*Gammaproteobacteria*	*Proteobacteria*	*Bacteria*
Otu1218	0.2	0	0.04	0.49	0.06	88.22	unclass. *Bacteria*	unclass. *Bacteria*	unclass. *Bacteria*	unclass. *Bacteria*	unclass. *Bacteria*	*Bacteria*
Otu1220	0.2	0	0.04	0.49	0.06	88.28	unclass. *Bacteria*	unclass. *Bacteria*	unclass. *Bacteria*	unclass. *Bacteria*	unclass. *Bacteria*	*Bacteria*
Otu1221	0.2	0	0.04	0.49	0.06	88.34	unclass. *Flavobacteriales*	unclass. *Flavobacteriales*	*Flavobacteriales*	*Flavobacteria*	*Bacteroidetes*	*Bacteria*
Otu1225	0.2	0	0.04	0.49	0.06	88.4	unclass. *Proteobacteria*	unclass. *Proteobacteria*	unclass. *Proteobacteria*	unclass. *Proteobacteria*	*Proteobacteria*	*Bacteria*
Otu1227	0.2	0	0.04	0.49	0.06	88.45	unclass. *Bacteria*	unclass. *Bacteria*	unclass. *Bacteria*	unclass. *Bacteria*	unclass. *Bacteria*	*Bacteria*
Otu1230	0.2	0	0.04	0.49	0.06	88.51	unclass. *Bacteroidetes*	unclass. *Bacteroidetes*	unclass. *Bacteroidetes*	unclass. *Bacteroidetes*	*Bacteroidetes*	*Bacteria*
Otu1239	0.2	0	0.04	0.49	0.06	88.57	*Sneathiella*	*Sneathiellaceae*	*Sneathiellales*	*Alphaproteobacteria*	*Proteobacteria*	*Bacteria*
Otu1240	0.2	0	0.04	0.49	0.06	88.63	unclass. *Gammaproteobacteria*	unclass. *Gammaproteobacteria*	unclass. *Gammaproteobacteria*	*Gammaproteobacteria*	*Proteobacteria*	*Bacteria*
Otu1241	0.2	0	0.04	0.49	0.06	88.69	unclass. *Gammaproteobacteria*	unclass. *Gammaproteobacteria*	unclass. *Gammaproteobacteria*	*Gammaproteobacteria*	*Proteobacteria*	*Bacteria*
Otu1243	0.2	0	0.04	0.49	0.06	88.75	unclass. *Bacteria*	unclass. *Bacteria*	unclass. *Bacteria*	unclass. *Bacteria*	unclass. *Bacteria*	*Bacteria*
Otu1244	0.2	0	0.04	0.49	0.06	88.81	unclass. *Bacteria*	unclass. *Bacteria*	unclass. *Bacteria*	unclass. *Bacteria*	unclass. *Bacteria*	*Bacteria*
Otu1246	0.2	0	0.04	0.49	0.06	88.87	unclass. *Gammaproteobacteria*	unclass. *Gammaproteobacteria*	unclass. *Gammaproteobacteria*	*Gammaproteobacteria*	*Proteobacteria*	*Bacteria*
Otu1248	0.2	0	0.04	0.49	0.06	88.93	unclass. *Bacteria*	unclass. *Bacteria*	unclass. *Bacteria*	unclass. *Bacteria*	unclass. *Bacteria*	*Bacteria*
Otu1250	0.2	0	0.04	0.49	0.06	88.99	unclass. *Bacteria*	unclass. *Bacteria*	unclass. *Bacteria*	unclass. *Bacteria*	unclass. *Bacteria*	*Bacteria*
Otu1251	0.2	0	0.04	0.49	0.06	89.05	unclass. *Bacteria*	unclass. *Bacteria*	unclass. *Bacteria*	unclass. *Bacteria*	unclass. *Bacteria*	*Bacteria*
Otu1252	0.2	0	0.04	0.49	0.06	89.11	unclass. *Flavobacteriaceae*	*Flavobacteriaceae*	*Flavobacteriales*	*Flavobacteria*	*Bacteroidetes*	*Bacteria*
Otu1253	0.2	0	0.04	0.49	0.06	89.17	unclass. *Proteobacteria*	unclass. *Proteobacteria*	unclass. *Proteobacteria*	unclass. *Proteobacteria*	*Proteobacteria*	*Bacteria*
Otu1258	0.2	0	0.04	0.49	0.06	89.23	*Amphritea*	*Oceanospirillaceae*	*Oceanospirillales*	*Gammaproteobacteria*	*Proteobacteria*	*Bacteria*
Otu1259	0.2	0	0.04	0.49	0.06	89.29	unclass. *Nannocystaceae*	*Nannocystaceae*	*Myxococcales*	*Deltaproteobacteria*	*Proteobacteria*	*Bacteria*
Otu1262	0.2	0	0.04	0.49	0.06	89.35	unclass. *Bacteroidetes*	unclass. *Bacteroidetes*	unclass. *Bacteroidetes*	unclass. *Bacteroidetes*	*Bacteroidetes*	*Bacteria*
Otu1263	0.2	0	0.04	0.49	0.06	89.41	unclass. *Gammaproteobacteria*	unclass. *Gammaproteobacteria*	unclass. *Gammaproteobacteria*	*Gammaproteobacteria*	*Proteobacteria*	*Bacteria*
Otu1264	0.2	0	0.04	0.49	0.06	89.47	unclass. *Bacteria*	unclass. *Bacteria*	unclass. *Bacteria*	unclass. *Bacteria*	unclass. *Bacteria*	*Bacteria*
Otu1265	0.2	0	0.04	0.49	0.06	89.53	unclass. *Bacteria*	unclass. *Bacteria*	unclass. *Bacteria*	unclass. *Bacteria*	unclass. *Bacteria*	*Bacteria*
Otu1266	0.2	0	0.04	0.49	0.06	89.59	unclass. *Gammaproteobacteria*	unclass. *Gammaproteobacteria*	unclass. *Gammaproteobacteria*	*Gammaproteobacteria*	*Proteobacteria*	*Bacteria*
Otu1269	0.2	0	0.04	0.49	0.06	89.65	unclass. *Bacteria*	unclass. *Bacteria*	unclass. *Bacteria*	unclass. *Bacteria*	unclass. *Bacteria*	*Bacteria*
Otu1271	0.2	0	0.04	0.49	0.06	89.71	unclass. *Alteromonadales*	unclass. *Alteromonadales*	*Alteromonadales*	*Gammaproteobacteria*	*Proteobacteria*	*Bacteria*

OTU	Av.A_i (*pH in situ*)	Av.A_i (pH 7.67)	Av.δ_i	Av.δ_i/SD	Av.δ_i %	∑Av.δ_i %	Genus	Family	Order	Class	Phylum	Domain
Otu1272	0.2	0	0.04	0.49	0.06	89.76	unclass. *Bacteria*	unclass. *Bacteria*	unclass. *Bacteria*	unclass. *Bacteria*	unclass. *Bacteria*	*Bacteria*
Otu1275	0.2	0	0.04	0.49	0.06	89.82	unclass. *Flavobacteriaceae*	*Flavobacteriaceae*	*Flavobacteriales*	*Flavobacteria*	*Bacteroidetes*	*Bacteria*
Otu1277	0.2	0	0.04	0.49	0.06	89.88	unclass. *Bacteroidetes*	unclass. *Bacteroidetes*	unclass. *Bacteroidetes*	unclass. *Bacteroidetes*	*Bacteroidetes*	*Bacteria*
Otu1280	0.2	0	0.04	0.49	0.06	89.94	unclass. *Alphaproteobacteria*	unclass. *Alphaproteobacteria*	unclass. *Alphaproteobacteria*	*Alphaproteobacteria*	*Proteobacteria*	*Bacteria*
Otu0002	0	0.2	0.04	0.49	0.06	90	unclass. *Flavobacteriaceae*	*Flavobacteriaceae*	*Flavobacteriales*	*Flavobacteria*	*Bacteroidetes*	*Bacteria*
Winter 'no dilution' (average dissimilarity: 60.9%)												
Otu0236	4.16	0.2	0.92	6.71	1.52	1.52	unclass. *Gammaproteobacteria*	unclass. *Gammaproteobacteria*	unclass. *Gammaproteobacteria*	*Gammaproteobacteria*	*Proteobacteria*	*Bacteria*
Otu0041	4.85	1.09	0.89	2.32	1.46	2.97	*Sulfitobacter*	*Rhodobacteraceae*	*Rhodobacterales*	*Alphaproteobacteria*	*Proteobacteria*	*Bacteria*
Otu0139	4.22	5.78	0.7	1.47	1.15	4.12	unclass. *Gammaproteobacteria*	unclass. *Gammaproteobacteria*	unclass. *Gammaproteobacteria*	*Gammaproteobacteria*	*Proteobacteria*	*Bacteria*
Otu1692	0.77	3.71	0.69	2.39	1.13	5.25	unclass. *Rhodobacteraceae*	*Rhodobacteraceae*	*Rhodobacterales*	*Alphaproteobacteria*	*Proteobacteria*	*Bacteria*
Otu0572	2.33	1.93	0.63	1.22	1.03	6.28	*Croceibacter*	*Flavobacteriaceae*	*Flavobacteriales*	*Flavobacteria*	*Bacteroidetes*	*Bacteria*
Otu0409	2.38	0.69	0.6	0.78	0.98	7.26	unclass. *Flavobacteriales*	unclass. *Flavobacteriales*	*Flavobacteriales*	*Flavobacteria*	*Bacteroidetes*	*Bacteria*
Otu0055	3.73	6.14	0.57	1.57	0.93	8.19	*Colwellia*	*Colwelliaceae*	*Alteromonadales*	*Gammaproteobacteria*	*Proteobacteria*	*Bacteria*
Otu1283	4.37	2.5	0.52	1.68	0.86	9.05	*Croceibacter*	*Flavobacteriaceae*	*Flavobacteriales*	*Flavobacteria*	*Bacteroidetes*	*Bacteria*
Otu0366	1.09	3.02	0.45	2.66	0.74	9.79	unclass. *Saprospiraceae*	*Saprospiraceae*	*Sphingobacteriales*	*Sphingobacteria*	*Bacteroidetes*	*Bacteria*
Otu1815	5.57	4.03	0.45	1.44	0.74	10.53	unclass. *Flavobacteriales*	unclass. *Flavobacteriales*	*Flavobacteriales*	*Flavobacteria*	*Bacteroidetes*	*Bacteria*
Otu1694	2.94	1.78	0.39	1.23	0.64	11.17	unclass. *Flavobacteriales*	unclass. *Flavobacteriales*	*Flavobacteriales*	*Flavobacteria*	*Bacteroidetes*	*Bacteria*
Otu0248	0	1.62	0.37	0.49	0.62	11.78	unclass. *Flavobacteriaceae*	*Flavobacteriaceae*	*Flavobacteriales*	*Flavobacteria*	*Bacteroidetes*	*Bacteria*
Otu0066	0.88	2.32	0.35	1.59	0.58	12.36	*Granulosicoccus*	*Granulosicoccaceae*	*Chromatiales*	*Gammaproteobacteria*	*Proteobacteria*	*Bacteria*
Otu0469	1.92	3.36	0.34	1.65	0.56	12.92	unclass. *Bacteria*	unclass. *Bacteria*	unclass. *Bacteria*	unclass. *Bacteria*	unclass. *Bacteria*	*Bacteria*
Otu0113	1.91	0.8	0.34	1.67	0.55	13.47	*Colwellia*	*Colwelliaceae*	*Alteromonadales*	*Gammaproteobacteria*	*Proteobacteria*	*Bacteria*
Otu1931	0.2	1.4	0.34	0.85	0.55	14.02	unclass. *Bacteria*	unclass. *Bacteria*	unclass. *Bacteria*	unclass. *Bacteria*	unclass. *Bacteria*	*Bacteria*
Otu0134	4.57	3.58	0.31	1.38	0.5	14.52	unclass. *Gammaproteobacteria*	unclass. *Gammaproteobacteria*	unclass. *Gammaproteobacteria*	*Gammaproteobacteria*	*Proteobacteria*	*Bacteria*
Otu0341	0.28	1.38	0.28	1.56	0.46	14.99	unclass. *Gammaproteobacteria*	unclass. *Gammaproteobacteria*	unclass. *Gammaproteobacteria*	*Gammaproteobacteria*	*Proteobacteria*	*Bacteria*
Otu0122	1.58	0.68	0.28	1.5	0.46	15.45	*Glaciecola*	*Alteromonadaceae*	*Alteromonadales*	*Gammaproteobacteria*	*Proteobacteria*	*Bacteria*
Otu0164	1.94	0.93	0.27	1.49	0.45	15.9	*Croceibacter*	*Flavobacteriaceae*	*Flavobacteriales*	*Flavobacteria*	*Bacteroidetes*	*Bacteria*
Otu0160	3.51	3.81	0.27	1.38	0.45	16.35	*Colwellia*	*Colwelliaceae*	*Alteromonadales*	*Gammaproteobacteria*	*Proteobacteria*	*Bacteria*

OTU	Av.A_i (*pH in situ*)	Av.A_i (pH 7.67)	Av.δ_i	Av.δ_i/SD	Av.δ_i %	∑Av.δ_i %	Genus	Family	Order	Class	Phylum	Domain
Otu0143	1.28	0.2	0.27	1.51	0.45	16.79	unclass. *Gammaproteobacteria*	unclass. *Gammaproteobacteria*	unclass. *Gammaproteobacteria*	*Gammaproteobacteria*	*Proteobacteria*	*Bacteria*
Otu0044	1.35	0.2	0.27	1.92	0.44	17.23	unclass. *Gammaproteobacteria*	unclass. *Gammaproteobacteria*	unclass. *Gammaproteobacteria*	*Gammaproteobacteria*	*Proteobacteria*	*Bacteria*
Otu0062	0.95	1.17	0.26	1.28	0.43	17.67	*Haliea*	*Alteromonadaceae*	*Alteromonadales*	*Gammaproteobacteria*	*Proteobacteria*	*Bacteria*
Otu1720	1.86	1.22	0.26	1.35	0.42	18.09	unclass. *Bacteria*	unclass. *Bacteria*	unclass. *Bacteria*	unclass. *Bacteria*	unclass. *Bacteria*	*Bacteria*
Otu0753	0	1.09	0.26	1.06	0.42	18.51	unclass. *Flavobacteriales*	unclass. *Flavobacteriales*	*Flavobacteriales*	*Flavobacteria*	*Bacteroidetes*	*Bacteria*
Otu0047	2.51	3.28	0.25	1.46	0.42	18.93	unclass. *Rhodobacteraceae*	*Rhodobacteraceae*	*Rhodobacterales*	*Alphaproteobacteria*	*Proteobacteria*	*Bacteria*
Otu0026	0.68	1.22	0.25	1.41	0.42	19.34	unclass. *Rhodobacteraceae*	*Rhodobacteraceae*	*Rhodobacterales*	*Alphaproteobacteria*	*Proteobacteria*	*Bacteria*
Otu1828	0.6	1.09	0.25	1.15	0.41	19.75	unclass. *Chromatiales*	unclass. *Chromatiales*	*Chromatiales*	*Gammaproteobacteria*	*Proteobacteria*	*Bacteria*
Otu0052	1.38	1.66	0.25	1.91	0.4	20.15	unclass. *Betaproteobacteria*	unclass. *Betaproteobacteria*	unclass. *Betaproteobacteria*	*Betaproteobacteria*	*Proteobacteria*	*Bacteria*
Otu0267	0.28	1.23	0.24	2.23	0.4	20.56	unclass. *Flavobacteriaceae*	*Flavobacteriaceae*	*Flavobacteriales*	*Flavobacteria*	*Bacteroidetes*	*Bacteria*
Otu0268	1.15	0.2	0.24	1.42	0.4	20.95	unclass. *Flavobacteriaceae*	*Flavobacteriaceae*	*Flavobacteriales*	*Flavobacteria*	*Bacteroidetes*	*Bacteria*
Otu0068	3.4	3.42	0.24	1.44	0.39	21.34	*Pelagibacter*	SAR11-clade	*Rickettsiales*	*Alphaproteobacteria*	*Proteobacteria*	*Bacteria*
Otu0002	1.11	0.2	0.23	1.59	0.38	21.72	unclass. *Flavobacteriaceae*	*Flavobacteriaceae*	*Flavobacteriales*	*Flavobacteria*	*Bacteroidetes*	*Bacteria*
Otu0124	1.51	1.01	0.23	1.59	0.38	22.1	*Loktanella*	*Rhodobacteraceae*	*Rhodobacterales*	*Alphaproteobacteria*	*Proteobacteria*	*Bacteria*
Otu0001	1.91	1.24	0.23	1.36	0.38	22.48	unclass. *Flavobacteriaceae*	*Flavobacteriaceae*	*Flavobacteriales*	*Flavobacteria*	*Bacteroidetes*	*Bacteria*
Otu0269	0.99	0	0.23	1.08	0.38	22.86	unclass. *Gammaproteobacteria*	unclass. *Gammaproteobacteria*	unclass. *Gammaproteobacteria*	*Gammaproteobacteria*	*Proteobacteria*	*Bacteria*
Otu1822	2.52	1.87	0.23	0.97	0.37	23.23	unclass. *Flavobacteriales*	unclass. *Flavobacteriales*	*Flavobacteriales*	*Flavobacteria*	*Bacteroidetes*	*Bacteria*
Otu0118	1.04	0.48	0.23	1.18	0.37	23.61	*Colwellia*	*Colwelliaceae*	*Alteromonadales*	*Gammaproteobacteria*	*Proteobacteria*	*Bacteria*
Otu0025	0.75	1	0.23	1.57	0.37	23.98	*Polaribacter*	*Flavobacteriaceae*	*Flavobacteriales*	*Flavobacteria*	*Bacteroidetes*	*Bacteria*
Otu0713	0.2	1.17	0.23	2.12	0.37	24.35	unclass. *Bacteria*	unclass. *Bacteria*	unclass. *Bacteria*	unclass. *Bacteria*	unclass. *Bacteria*	*Bacteria*
Otu0261	1.51	1.66	0.22	1.37	0.37	24.72	*Winogradskyella*	*Flavobacteriaceae*	*Flavobacteriales*	*Flavobacteria*	*Bacteroidetes*	*Bacteria*
Otu0163	1.87	1.46	0.22	1.46	0.37	25.08	unclass. *Gammaproteobacteria*	unclass. *Gammaproteobacteria*	unclass. *Gammaproteobacteria*	*Gammaproteobacteria*	*Proteobacteria*	*Bacteria*
Otu0213	0.2	1.05	0.22	1.6	0.36	25.44	unclass. *Gammaproteobacteria*	unclass. *Gammaproteobacteria*	unclass. *Gammaproteobacteria*	*Gammaproteobacteria*	*Proteobacteria*	*Bacteria*
Otu1937	0	0.91	0.22	1.19	0.35	25.8	unclass. *Gammaproteobacteria*	unclass. *Gammaproteobacteria*	unclass. *Gammaproteobacteria*	*Gammaproteobacteria*	*Proteobacteria*	*Bacteria*
Otu0022	1.34	1.09	0.21	1.36	0.35	26.15	unclass. *Betaproteobacteria*	unclass. *Betaproteobacteria*	unclass. *Betaproteobacteria*	*Betaproteobacteria*	*Proteobacteria*	*Bacteria*
Otu0621	0	0.88	0.21	1.85	0.34	26.49	*Colwellia*	*Colwelliaceae*	*Alteromonadales*	*Gammaproteobacteria*	*Proteobacteria*	*Bacteria*
Otu1146	1.58	0.83	0.2	1.27	0.33	26.82	*Nitrosospira*	*Nitrosomonadaceae*	*Nitrosomonadales*	*Betaproteobacteria*	*Proteobacteria*	*Bacteria*

OTU	Av.A_i (*pH in situ*)	Av.A_i (pH 7.67)	Av.δ_i	Av.δ_i/SD	Av.δ_i %	∑Av.δ_i %	Genus	Family	Order	Class	Phylum	Domain
Otu0005	2.51	2.76	0.2	1.11	0.33	27.15	*Pelagibacter*	SAR11-clade	*Rickettsiales*	*Alphaproteobacteria*	*Proteobacteria*	*Bacteria*
Otu0013	1.09	1.35	0.2	1.52	0.33	27.49	unclass. *Flavobacteriaceae*	*Flavobacteriaceae*	*Flavobacteriales*	*Flavobacteria*	*Bacteroidetes*	*Bacteria*
Otu0032	1.79	1.03	0.2	1.39	0.33	27.81	unclass. *Gammaproteobacteria*	unclass. *Gammaproteobacteria*	unclass. *Gammaproteobacteria*	*Gammaproteobacteria*	*Proteobacteria*	*Bacteria*
Otu0094	0.4	1.09	0.2	1.33	0.33	28.14	unclass. *Flavobacteriaceae*	*Flavobacteriaceae*	*Flavobacteriales*	*Flavobacteria*	*Bacteroidetes*	*Bacteria*
Otu0010	7.33	6.83	0.2	1.39	0.32	28.46	unclass. *Flavobacteriaceae*	*Flavobacteriaceae*	*Flavobacteriales*	*Flavobacteria*	*Bacteroidetes*	*Bacteria*
Otu1923	0.2	0.79	0.19	0.92	0.32	28.78	unclass. *Bacteria*	unclass. *Bacteria*	unclass. *Bacteria*	unclass. *Bacteria*	unclass. *Bacteria*	*Bacteria*
Otu0046	0.95	0.2	0.19	1.4	0.32	29.09	unclass. *Flavobacteriaceae*	*Flavobacteriaceae*	*Flavobacteriales*	*Flavobacteria*	*Bacteroidetes*	*Bacteria*
Otu1081	0.82	0	0.19	0.49	0.31	29.41	unclass. *Flavobacteriales*	unclass. *Flavobacteriales*	*Flavobacteriales*	*Flavobacteria*	*Bacteroidetes*	*Bacteria*
Otu0282	0.4	0.68	0.19	1.27	0.31	29.72	*Haliea*	*Alteromonadaceae*	*Alteromonadales*	*Gammaproteobacteria*	*Proteobacteria*	*Bacteria*
Otu0156	0.2	0.85	0.19	1.03	0.31	30.03	unclass. *Gammaproteobacteria*	unclass. *Gammaproteobacteria*	unclass. *Gammaproteobacteria*	*Gammaproteobacteria*	*Proteobacteria*	*Bacteria*
Otu0369	0.8	0	0.19	1.96	0.3	30.33	unclass. *Deltaproteobacteria*	unclass. *Deltaproteobacteria*	unclass. *Deltaproteobacteria*	*Deltaproteobacteria*	*Proteobacteria*	*Bacteria*
Otu0076	1.63	1.64	0.18	1.18	0.3	30.63	unclass. *Flammeovirgaceae*	*Flammeovirgaceae*	*Sphingobacteriales*	*Sphingobacteria*	*Bacteroidetes*	*Bacteria*
Otu0035	1.78	2.06	0.18	1.41	0.3	30.93	*Colwellia*	*Colwelliaceae*	*Alteromonadales*	*Gammaproteobacteria*	*Proteobacteria*	*Bacteria*
Otu0140	1.09	0.77	0.18	1.27	0.3	31.23	*Oleispira*	*Oceanospirillaceae*	*Oceanospirillales*	*Gammaproteobacteria*	*Proteobacteria*	*Bacteria*
Otu0036	0.77	0	0.18	1.16	0.3	31.52	*Glaciecola*	*Alteromonadaceae*	*Alteromonadales*	*Gammaproteobacteria*	*Proteobacteria*	*Bacteria*
Otu0170	0.88	0.2	0.18	1.44	0.29	31.82	unclass. *Bacteria*	unclass. *Bacteria*	unclass. *Bacteria*	unclass. *Bacteria*	unclass. *Bacteria*	*Bacteria*
Otu0508	0.28	0.8	0.18	1.83	0.29	32.11	unclass. *Flavobacteriales*	unclass. *Flavobacteriales*	*Flavobacteriales*	*Flavobacteria*	*Bacteroidetes*	*Bacteria*
Otu0042	0.2	0.88	0.18	1.46	0.29	32.4	*Rhodococcus*	*Nocardiaceae*	*Actinomycetales*	*Actinobacteria*	*Actinobacteria*	*Bacteria*
Otu1837	0.88	0.2	0.18	1.45	0.29	32.69	unclass. *Flavobacteriales*	unclass. *Flavobacteriales*	*Flavobacteriales*	*Flavobacteria*	*Bacteroidetes*	*Bacteria*
Otu1723	1.08	0.77	0.18	1.21	0.29	32.99	unclass. *Bacteroidetes*	unclass. *Bacteroidetes*	unclass. *Bacteroidetes*	unclass. *Bacteroidetes*	*Bacteroidetes*	*Bacteria*
Otu0232	0.89	0.88	0.18	1.44	0.29	33.27	unclass. *Rhodobacteraceae*	*Rhodobacteraceae*	*Rhodobacterales*	*Alphaproteobacteria*	*Proteobacteria*	*Bacteria*
Otu0012	1.49	1.91	0.17	1.24	0.28	33.56	unclass. *Comamonadaceae*	*Comamonadaceae*	*Burkholderiales*	*Betaproteobacteria*	*Proteobacteria*	*Bacteria*
Otu0216	0.63	0.57	0.17	0.99	0.28	33.84	*Pelagibacter*	SAR11-clade	*Rickettsiales*	*Alphaproteobacteria*	*Proteobacteria*	*Bacteria*
Otu0333	0.48	0.75	0.17	1.16	0.27	34.12	unclass. *Gammaproteobacteria*	unclass. *Gammaproteobacteria*	unclass. *Gammaproteobacteria*	*Gammaproteobacteria*	*Proteobacteria*	*Bacteria*
Otu0117	0.4	0.63	0.16	1.11	0.27	34.39	*Arcobacter*	*Campylobacteraceae*	*Campylobacterales*	*Epsilonproteobacteria*	*Proteobacteria*	*Bacteria*
Otu0521	0.68	0.57	0.16	1.19	0.27	34.66	unclass. *Rhodobacteraceae*	*Rhodobacteraceae*	*Rhodobacterales*	*Alphaproteobacteria*	*Proteobacteria*	*Bacteria*
Otu0230	0.28	0.6	0.16	0.88	0.27	34.93	unclass. *Rhodobacteraceae*	*Rhodobacteraceae*	*Rhodobacterales*	*Alphaproteobacteria*	*Proteobacteria*	*Bacteria*

OTU	Av.A_i (*pH in situ*)	Av.A_i (pH 7.67)	Av.δ_i	Av.δ_i/SD	Av.δ_i %	∑Av.δ_i %	Genus	Family	Order	Class	Phylum	Domain
Otu0270	1.55	1.03	0.16	1.21	0.27	35.19	unclass. *Bacteroidetes*	unclass. *Bacteroidetes*	unclass. *Bacteroidetes*	unclass. *Bacteroidetes*	*Bacteroidetes*	*Bacteria*
Otu1821	0.68	0.28	0.16	1.16	0.27	35.46	unclass. *Bacteroidetes*	unclass. *Bacteroidetes*	unclass. *Bacteroidetes*	unclass. *Bacteroidetes*	*Bacteroidetes*	*Bacteria*
Otu0172	0.48	0.88	0.16	1.3	0.27	35.73	unclass. *Gammaproteobacteria*	unclass. *Gammaproteobacteria*	unclass. *Gammaproteobacteria*	*Gammaproteobacteria*	*Proteobacteria*	*Bacteria*
Otu1629	0.68	0.28	0.16	1.17	0.27	35.99	unclass. *Flammeovirgaceae*	*Flammeovirgaceae*	*Sphingobacteriales*	*Sphingobacteria*	*Bacteroidetes*	*Bacteria*
Otu0272	0.4	0.6	0.16	0.98	0.26	36.26	unclass. *Flavobacteriaceae*	*Flavobacteriaceae*	*Flavobacteriales*	*Flavobacteria*	*Bacteroidetes*	*Bacteria*
Otu0313	0	0.68	0.16	1.16	0.26	36.52	unclass. *Gammaproteobacteria*	unclass. *Gammaproteobacteria*	unclass. *Gammaproteobacteria*	*Gammaproteobacteria*	*Proteobacteria*	*Bacteria*
Otu0203	0.88	0.83	0.16	1.31	0.26	36.78	*Pelagibacter*	SAR11-clade	*Rickettsiales*	*Alphaproteobacteria*	*Proteobacteria*	*Bacteria*
Otu0348	0.8	0.2	0.16	1.43	0.26	37.05	unclass. *Flavobacteriaceae*	*Flavobacteriaceae*	*Flavobacteriales*	*Flavobacteria*	*Bacteroidetes*	*Bacteria*
Otu0337	0.2	0.63	0.16	0.93	0.26	37.3	unclass. *Bacteria*	unclass. *Bacteria*	unclass. *Bacteria*	unclass. *Bacteria*	unclass. *Bacteria*	*Bacteria*
Otu0190	0.2	0.63	0.16	0.93	0.26	37.56	unclass. *Proteobacteria*	unclass. *Proteobacteria*	unclass. *Proteobacteria*	unclass. *Proteobacteria*	*Proteobacteria*	*Bacteria*
Otu0262	1.17	1.29	0.16	1.63	0.25	37.81	unclass. *Gammaproteobacteria*	unclass. *Gammaproteobacteria*	unclass. *Gammaproteobacteria*	*Gammaproteobacteria*	*Proteobacteria*	*Bacteria*
Otu1848	0.48	0.68	0.15	1.14	0.25	38.07	unclass. *Gammaproteobacteria*	unclass. *Gammaproteobacteria*	unclass. *Gammaproteobacteria*	*Gammaproteobacteria*	*Proteobacteria*	*Bacteria*
Otu0051	0.6	0.28	0.15	1.26	0.25	38.31	unclass. *Flavobacteriaceae*	*Flavobacteriaceae*	*Flavobacteriales*	*Flavobacteria*	*Bacteroidetes*	*Bacteria*
Otu1920	0.28	0.6	0.15	1.27	0.25	38.56	unclass. *Gammaproteobacteria*	unclass. *Gammaproteobacteria*	unclass. *Gammaproteobacteria*	*Gammaproteobacteria*	*Proteobacteria*	*Bacteria*
Otu0359	0.2	0.68	0.15	1.13	0.25	38.81	unclass. *Gammaproteobacteria*	unclass. *Gammaproteobacteria*	unclass. *Gammaproteobacteria*	*Gammaproteobacteria*	*Proteobacteria*	*Bacteria*
Otu1119	0.48	0.8	0.15	1.35	0.25	39.06	unclass. *Bacteroidetes*	unclass. *Bacteroidetes*	unclass. *Bacteroidetes*	unclass. *Bacteroidetes*	*Bacteroidetes*	*Bacteria*
Otu1873	0.68	0.2	0.15	1.13	0.25	39.3	unclass. *Bacteria*	unclass. *Bacteria*	unclass. *Bacteria*	unclass. *Bacteria*	unclass. *Bacteria*	*Bacteria*
Otu0133	0.97	0.68	0.15	1.12	0.24	39.54	*Colwellia*	*Colwelliaceae*	*Alteromonadales*	*Gammaproteobacteria*	*Proteobacteria*	*Bacteria*
Otu0290	0.55	0.4	0.15	1.01	0.24	39.78	unclass. *Flavobacteriaceae*	*Flavobacteriaceae*	*Flavobacteriales*	*Flavobacteria*	*Bacteroidetes*	*Bacteria*
Otu0009	0.97	1	0.14	1.14	0.24	40.02	unclass. *Rhodobacteraceae*	*Rhodobacteraceae*	*Rhodobacterales*	*Alphaproteobacteria*	*Proteobacteria*	*Bacteria*
Otu0673	0.48	0.48	0.14	1	0.23	40.25	*Thalassolituus*	*Oceanospirillaceae*	*Oceanospirillales*	*Gammaproteobacteria*	*Proteobacteria*	*Bacteria*
Otu0878	0	0.6	0.14	1.2	0.23	40.48	unclass. *Bacteria*	unclass. *Bacteria*	unclass. *Bacteria*	unclass. *Bacteria*	unclass. *Bacteria*	*Bacteria*
Otu1057	0	0.6	0.14	1.2	0.23	40.72	unclass. *Gammaproteobacteria*	unclass. *Gammaproteobacteria*	unclass. *Gammaproteobacteria*	*Gammaproteobacteria*	*Proteobacteria*	*Bacteria*
Otu0072	0.48	0.6	0.14	1.17	0.23	40.95	*Sulfitobacter*	*Rhodobacteraceae*	*Rhodobacterales*	*Alphaproteobacteria*	*Proteobacteria*	*Bacteria*
Otu0173	0.55	0.2	0.14	0.87	0.23	41.17	unclass. *Alteromonadaceae*	*Alteromonadaceae*	*Alteromonadales*	*Gammaproteobacteria*	*Proteobacteria*	*Bacteria*
Otu0413	0.35	0.4	0.14	0.93	0.22	41.4	*Eudoraea*	*Flavobacteriaceae*	*Flavobacteriales*	*Flavobacteria*	*Bacteroidetes*	*Bacteria*
Otu0354	0.28	0.48	0.14	0.88	0.22	41.62	*Anderseniella*	*Rhodobiaceae*	*Rhizobiales*	*Alphaproteobacteria*	*Proteobacteria*	*Bacteria*

OTU	Av.A_i (*pH in situ*)	Av.A_i (pH 7.67)	Av.δ_i	Av.δ_i/SD	Av.δ_i %	∑Av.δ_i %	Genus	Family	Order	Class	Phylum	Domain
Otu0056	1.39	0.95	0.14	1.23	0.22	41.84	unclass. *Gammaproteobacteria*	unclass. *Gammaproteobacteria*	unclass. *Gammaproteobacteria*	*Gammaproteobacteria*	*Proteobacteria*	*Bacteria*
Otu1159	0	0.57	0.13	0.8	0.22	42.06	unclass. *Gammaproteobacteria*	unclass. *Gammaproteobacteria*	unclass. *Gammaproteobacteria*	*Gammaproteobacteria*	*Proteobacteria*	*Bacteria*
Otu0254	0.48	0.28	0.13	0.89	0.22	42.28	unclass. *Flavobacteriaceae*	*Flavobacteriaceae*	*Flavobacteriales*	*Flavobacteria*	*Bacteroidetes*	*Bacteria*
Otu0696	0.4	0.48	0.13	1	0.22	42.5	unclass. *Flavobacteriaceae*	*Flavobacteriaceae*	*Flavobacteriales*	*Flavobacteria*	*Bacteroidetes*	*Bacteria*
Otu1816	0.8	0.4	0.13	1.1	0.22	42.72	unclass. *Flavobacteriales*	unclass. *Flavobacteriales*	*Flavobacteriales*	*Flavobacteria*	*Bacteroidetes*	*Bacteria*
Otu0121	0.2	0.45	0.13	0.66	0.22	42.93	unclass. *Deltaproteobacteria*	unclass. *Deltaproteobacteria*	unclass. *Deltaproteobacteria*	*Deltaproteobacteria*	*Proteobacteria*	*Bacteria*
Otu0310	0.48	0.4	0.13	1.01	0.22	43.15	unclass. *Bacteria*	unclass. *Bacteria*	unclass. *Bacteria*	unclass. *Bacteria*	unclass. *Bacteria*	*Bacteria*
Otu0202	0.48	0.4	0.13	1.01	0.22	43.36	unclass. *Alteromonadaceae*	*Alteromonadaceae*	*Alteromonadales*	*Gammaproteobacteria*	*Proteobacteria*	*Bacteria*
Otu0027	0.57	0	0.13	0.8	0.22	43.58	*Pseudoalteromonas*	*Pseudoalteromonadaceae*	*Alteromonadales*	*Gammaproteobacteria*	*Proteobacteria*	*Bacteria*
Otu0364	0.6	0.2	0.13	1.1	0.21	43.79	unclass. *Flavobacteriaceae*	*Flavobacteriaceae*	*Flavobacteriales*	*Flavobacteria*	*Bacteroidetes*	*Bacteria*
Otu0899	0.6	0.2	0.13	1.1	0.21	44.01	unclass. *Betaproteobacteria*	unclass. *Betaproteobacteria*	unclass. *Betaproteobacteria*	*Betaproteobacteria*	*Proteobacteria*	*Bacteria*
Otu1379	0	0.55	0.13	0.75	0.21	44.21	unclass. *Gammaproteobacteria*	unclass. *Gammaproteobacteria*	unclass. *Gammaproteobacteria*	*Gammaproteobacteria*	*Proteobacteria*	*Bacteria*
Otu0204	0.2	0.48	0.12	0.89	0.2	44.42	unclass. *Colwelliaceae*	*Colwelliaceae*	*Alteromonadales*	*Gammaproteobacteria*	*Proteobacteria*	*Bacteria*
Otu0312	0.2	0.48	0.12	0.88	0.2	44.62	unclass. *Oceanospirillaceae*	*Oceanospirillaceae*	*Oceanospirillales*	*Gammaproteobacteria*	*Proteobacteria*	*Bacteria*
Otu0234	0.6	0.88	0.12	0.99	0.2	44.82	*Arcobacter*	*Campylobacteraceae*	*Campylobacterales*	*Epsilonproteobacteria*	*Proteobacteria*	*Bacteria*
Otu1820	0.2	0.48	0.12	0.89	0.2	45.02	*Glaciecola*	*Alteromonadaceae*	*Alteromonadales*	*Gammaproteobacteria*	*Proteobacteria*	*Bacteria*
Otu1223	0.2	0.48	0.12	0.89	0.2	45.22	unclass. *Desulfobulbaceae*	*Desulfobulbaceae*	*Desulfobacterales*	*Deltaproteobacteria*	*Proteobacteria*	*Bacteria*
Otu0169	0.6	0.4	0.12	1.02	0.2	45.42	unclass. *Bacteria*	unclass. *Bacteria*	unclass. *Bacteria*	unclass. *Bacteria*	unclass. *Bacteria*	*Bacteria*
Otu1637	0.2	0.48	0.12	0.89	0.2	45.62	unclass. *Proteobacteria*	unclass. *Proteobacteria*	unclass. *Proteobacteria*	unclass. *Proteobacteria*	*Proteobacteria*	*Bacteria*
Otu0291	1.23	1.03	0.12	1.06	0.19	45.81	unclass. *Flavobacteriaceae*	*Flavobacteriaceae*	*Flavobacteriales*	*Flavobacteria*	*Bacteroidetes*	*Bacteria*
Otu0037	0	0.48	0.11	0.79	0.19	46	*Marinobacter*	*Alteromonadaceae*	*Alteromonadales*	*Gammaproteobacteria*	*Proteobacteria*	*Bacteria*
Otu1770	0	0.48	0.11	0.79	0.19	46.18	unclass. *Actinobacteria*	unclass. *Actinobacteria*	unclass. *Actinobacteria*	*Actinobacteria*	*Actinobacteria*	*Bacteria*
Otu2030	0	0.48	0.11	0.79	0.19	46.37	unclass. *Bacteria*	unclass. *Bacteria*	unclass. *Bacteria*	unclass. *Bacteria*	unclass. *Bacteria*	*Bacteria*
Otu2029	0	0.48	0.11	0.78	0.18	46.56	unclass. *Bacteria*	unclass. *Bacteria*	unclass. *Bacteria*	unclass. *Bacteria*	unclass. *Bacteria*	*Bacteria*
Otu1944	0	0.48	0.11	0.78	0.18	46.74	unclass. *Proteobacteria*	unclass. *Proteobacteria*	unclass. *Proteobacteria*	unclass. *Proteobacteria*	*Proteobacteria*	*Bacteria*
Otu0215	0	0.48	0.11	0.78	0.18	46.92	unclass. *Domain*	unclass. *Domain*	unclass. *Domain*	unclass. *Domain*	unclass. *Domain*	unclass. *Domain*
Otu0153	0.48	0	0.11	0.78	0.18	47.11	unclass. *Gammaproteobacteria*	unclass. *Gammaproteobacteria*	unclass. *Gammaproteobacteria*	*Gammaproteobacteria*	*Proteobacteria*	*Bacteria*

OTU	Av.A_i (*pH in situ*)	Av.A_i (pH 7.67)	Av.δ_i	Av.δ_i/SD	Av.δ_i %	∑Av.δ_i %	Genus	Family	Order	Class	Phylum	Domain
Otu1897	0.2	0.35	0.11	0.68	0.18	47.29	unclass. *Flavobacteriaceae*	*Flavobacteriaceae*	*Flavobacteriales*	*Flavobacteria*	*Bacteroidetes*	*Bacteria*
Otu0028	0.2	0.35	0.11	0.69	0.18	47.47	unclass. *Gammaproteobacteria*	unclass. *Gammaproteobacteria*	unclass. *Gammaproteobacteria*	*Gammaproteobacteria*	*Proteobacteria*	*Bacteria*
Otu0177	1.17	1.03	0.11	1.05	0.18	47.65	*Haliea*	*Alteromonadaceae*	*Alteromonadales*	*Gammaproteobacteria*	*Proteobacteria*	*Bacteria*
Otu1111	0.45	0	0.1	0.49	0.17	47.82	unclass. *Proteobacteria*	unclass. *Proteobacteria*	unclass. *Proteobacteria*	unclass. *Proteobacteria*	*Proteobacteria*	*Bacteria*
Otu2067	0	0.45	0.1	0.49	0.17	47.99	unclass. *Bacteria*	unclass. *Bacteria*	unclass. *Bacteria*	unclass. *Bacteria*	unclass. *Bacteria*	*Bacteria*
Otu0283	0.4	0.2	0.1	0.87	0.17	48.16	unclass. *Gammaproteobacteria*	unclass. *Gammaproteobacteria*	unclass. *Gammaproteobacteria*	*Gammaproteobacteria*	*Proteobacteria*	*Bacteria*
Otu0208	0.2	0.4	0.1	0.87	0.17	48.33	unclass. *Saprospiraceae*	*Saprospiraceae*	*Sphingobacteriales*	*Sphingobacteria*	*Bacteroidetes*	*Bacteria*
Otu1249	0.2	0.4	0.1	0.87	0.17	48.5	unclass. *Gammaproteobacteria*	unclass. *Gammaproteobacteria*	unclass. *Gammaproteobacteria*	*Gammaproteobacteria*	*Proteobacteria*	*Bacteria*
Otu1861	0.4	0.2	0.1	0.87	0.17	48.67	unclass. *Gammaproteobacteria*	unclass. *Gammaproteobacteria*	unclass. *Gammaproteobacteria*	*Gammaproteobacteria*	*Proteobacteria*	*Bacteria*
Otu0315	0.2	0.4	0.1	0.87	0.17	48.84	unclass. *Gammaproteobacteria*	unclass. *Gammaproteobacteria*	unclass. *Gammaproteobacteria*	*Gammaproteobacteria*	*Proteobacteria*	*Bacteria*
Otu0199	0.2	0.4	0.1	0.87	0.17	49.01	unclass. *Flavobacteriaceae*	*Flavobacteriaceae*	*Flavobacteriales*	*Flavobacteria*	*Bacteroidetes*	*Bacteria*
Otu1836	0.4	0.2	0.1	0.87	0.17	49.18	unclass. *Bacteroidetes*	unclass. *Bacteroidetes*	unclass. *Bacteroidetes*	unclass. *Bacteroidetes*	*Bacteroidetes*	*Bacteria*
Otu0057	0.4	0.2	0.1	0.87	0.17	49.35	*Ulvibacter*	*Flavobacteriaceae*	*Flavobacteriales*	*Flavobacteria*	*Bacteroidetes*	*Bacteria*
Otu1941	0.2	0.4	0.1	0.87	0.17	49.52	*Haliea*	*Alteromonadaceae*	*Alteromonadales*	*Gammaproteobacteria*	*Proteobacteria*	*Bacteria*
Otu2098	0	0.45	0.1	0.49	0.17	49.69	unclass. *Bacteria*	unclass. *Bacteria*	unclass. *Bacteria*	unclass. *Bacteria*	unclass. *Bacteria*	*Bacteria*
Otu0448	0.4	0.2	0.1	0.87	0.17	49.86	unclass. *Desulfuromonadaceae*	*Desulfuromonadaceae*	*Desulfuromonadales*	*Deltaproteobacteria*	*Proteobacteria*	*Bacteria*
Otu1566	0.4	0.2	0.1	0.87	0.17	50.03	*Gaetbulibacter*	*Flavobacteriaceae*	*Flavobacteriales*	*Flavobacteria*	*Bacteroidetes*	*Bacteria*
Otu0356	0.4	0.2	0.1	0.87	0.17	50.2	unclass. *Comamonadaceae*	*Comamonadaceae*	*Burkholderiales*	*Betaproteobacteria*	*Proteobacteria*	*Bacteria*
Otu0019	0.4	0.2	0.1	0.87	0.17	50.37	unclass. *Gammaproteobacteria*	unclass. *Gammaproteobacteria*	unclass. *Gammaproteobacteria*	*Gammaproteobacteria*	*Proteobacteria*	*Bacteria*
Otu0319	0.4	0.2	0.1	0.87	0.17	50.54	unclass. *Flammeovirgaceae*	*Flammeovirgaceae*	*Sphingobacteriales*	*Sphingobacteria*	*Bacteroidetes*	*Bacteria*
Otu0954	0.2	0.4	0.1	0.87	0.17	50.7	unclass. *Gammaproteobacteria*	unclass. *Gammaproteobacteria*	unclass. *Gammaproteobacteria*	*Gammaproteobacteria*	*Proteobacteria*	*Bacteria*
Otu1851	0.4	0.2	0.1	0.87	0.17	50.87	*Aureispira*	*Saprospiraceae*	*Sphingobacteriales*	*Sphingobacteria*	*Bacteroidetes*	*Bacteria*
Otu0370	0.4	0.2	0.1	0.87	0.17	51.04	unclass. *Gammaproteobacteria*	unclass. *Gammaproteobacteria*	unclass. *Gammaproteobacteria*	*Gammaproteobacteria*	*Proteobacteria*	*Bacteria*
Otu0686	0.4	0.2	0.1	0.87	0.17	51.21	*Marinomonas*	*Oceanospirillaceae*	*Oceanospirillales*	*Gammaproteobacteria*	*Proteobacteria*	*Bacteria*
Otu0347	0.4	0	0.1	0.49	0.16	51.37	unclass. *Flavobacteriaceae*	*Flavobacteriaceae*	*Flavobacteriales*	*Flavobacteria*	*Bacteroidetes*	*Bacteria*
Otu0151	0.28	0.2	0.1	0.69	0.16	51.52	*Rhodococcus*	*Nocardiaceae*	*Actinomycetales*	*Actinobacteria*	*Actinobacteria*	*Bacteria*
Otu0192	0.2	0.28	0.1	0.7	0.16	51.68	unclass. *Gammaproteobacteria*	unclass. *Gammaproteobacteria*	unclass. *Gammaproteobacteria*	*Gammaproteobacteria*	*Proteobacteria*	*Bacteria*

OTU	Av.A_i (*pH in situ*)	Av.A_i (pH 7.67)	Av.δ_i	Av.δ_i/SD	Av.δ_i %	∑Av.δ_i %	Genus	Family	Order	Class	Phylum	Domain
Otu1697	0.4	0	0.1	0.8	0.16	51.84	unclass. *Bacteria*	unclass. *Bacteria*	unclass. *Bacteria*	unclass. *Bacteria*	unclass. *Bacteria*	*Bacteria*
Otu0168	0.2	0.28	0.1	0.7	0.16	51.99	unclass. *Flavobacteriaceae*	*Flavobacteriaceae*	*Flavobacteriales*	*Flavobacteria*	*Bacteroidetes*	*Bacteria*
Otu1888	0.2	0.28	0.1	0.7	0.16	52.15	unclass. *Bacteria*	unclass. *Bacteria*	unclass. *Bacteria*	unclass. *Bacteria*	unclass. *Bacteria*	*Bacteria*
Otu0212	0	0.4	0.09	0.8	0.16	52.3	*Balneola*	*Chitinophagaceae*	*Sphingobacteriales*	*Sphingobacteria*	*Bacteroidetes*	*Bacteria*
Otu1267	0.4	0	0.09	0.8	0.16	52.46	unclass. *Gammaproteobacteria*	unclass. *Gammaproteobacteria*	unclass. *Gammaproteobacteria*	*Gammaproteobacteria*	*Proteobacteria*	*Bacteria*
Otu1818	0.4	0	0.09	0.8	0.16	52.61	unclass. *Bacteria*	unclass. *Bacteria*	unclass. *Bacteria*	unclass. *Bacteria*	unclass. *Bacteria*	*Bacteria*
Otu0497	0.4	0	0.09	0.8	0.16	52.77	*Opitutus*	*Opitutaceae*	*Opitutales*	*Opitutae*	*Verrucomicrobia*	*Bacteria*
Otu1480	0.4	0	0.09	0.8	0.16	52.92	unclass. *Flavobacteriaceae*	*Flavobacteriaceae*	*Flavobacteriales*	*Flavobacteria*	*Bacteroidetes*	*Bacteria*
Otu0324	0	0.4	0.09	0.8	0.15	53.08	*Nisaea*	*Rhodospirillaceae*	*Rhodospirillales*	*Alphaproteobacteria*	*Proteobacteria*	*Bacteria*
Otu1738	0	0.4	0.09	0.8	0.15	53.23	unclass. *Proteobacteria*	unclass. *Proteobacteria*	unclass. *Proteobacteria*	unclass. *Proteobacteria*	*Proteobacteria*	*Bacteria*
Otu1927	0.2	0.28	0.09	0.69	0.15	53.39	unclass. *Flavobacteriales*	unclass. *Flavobacteriales*	*Flavobacteriales*	*Flavobacteria*	*Bacteroidetes*	*Bacteria*
Otu1789	0.2	0.28	0.09	0.69	0.15	53.54	*Colwellia*	*Colwelliaceae*	*Alteromonadales*	*Gammaproteobacteria*	*Proteobacteria*	*Bacteria*
Otu0148	0	0.4	0.09	0.8	0.15	53.7	unclass. *Sphingobacteriales*	unclass. *Sphingobacteriales*	*Sphingobacteriales*	*Sphingobacteria*	*Bacteroidetes*	*Bacteria*
Otu1997	0	0.4	0.09	0.8	0.15	53.85	unclass. *Sphingobacteriales*	unclass. *Sphingobacteriales*	*Sphingobacteriales*	*Sphingobacteria*	*Bacteroidetes*	*Bacteria*
Otu0115	0	0.4	0.09	0.49	0.15	54	*Glaciecola*	*Alteromonadaceae*	*Alteromonadales*	*Gammaproteobacteria*	*Proteobacteria*	*Bacteria*
Otu0266	0	0.4	0.09	0.49	0.15	54.16	unclass. *Bacteria*	unclass. *Bacteria*	unclass. *Bacteria*	unclass. *Bacteria*	unclass. *Bacteria*	*Bacteria*
Otu1833	0.2	0.28	0.09	0.69	0.15	54.31	unclass. *Flavobacteriaceae*	*Flavobacteriaceae*	*Flavobacteriales*	*Flavobacteria*	*Bacteroidetes*	*Bacteria*
Otu1854	0.28	0.2	0.09	0.69	0.15	54.46	unclass. *Gammaproteobacteria*	unclass. *Gammaproteobacteria*	unclass. *Gammaproteobacteria*	*Gammaproteobacteria*	*Proteobacteria*	*Bacteria*
Otu0185	0.28	0.2	0.09	0.69	0.15	54.62	unclass. *Bacteria*	unclass. *Bacteria*	unclass. *Bacteria*	unclass. *Bacteria*	unclass. *Bacteria*	*Bacteria*
Otu0015	0.4	0	0.09	0.8	0.15	54.77	*Polaribacter*	*Flavobacteriaceae*	*Flavobacteriales*	*Flavobacteria*	*Bacteroidetes*	*Bacteria*
Otu0093	0.4	0	0.09	0.8	0.15	54.92	*Aestuariicola*	*Flavobacteriaceae*	*Flavobacteriales*	*Flavobacteria*	*Bacteroidetes*	*Bacteria*
Otu0443	0.4	0	0.09	0.8	0.15	55.08	unclass. *Gammaproteobacteria*	unclass. *Gammaproteobacteria*	unclass. *Gammaproteobacteria*	*Gammaproteobacteria*	*Proteobacteria*	*Bacteria*
Otu0858	0.4	0	0.09	0.8	0.15	55.23	unclass. *Flavobacteriaceae*	*Flavobacteriaceae*	*Flavobacteriales*	*Flavobacteria*	*Bacteroidetes*	*Bacteria*
Otu1827	0.4	0	0.09	0.8	0.15	55.38	unclass. *Acidobacteria_Gp22*	unclass. *Acidobacteria_Gp22*	unclass. *Acidobacteria_Gp22*	*Acidobacteria_Gp22*	*Acidobacteria*	*Bacteria*
Otu1865	0.2	0.28	0.09	0.69	0.15	55.54	unclass. *Proteobacteria*	unclass. *Proteobacteria*	unclass. *Proteobacteria*	unclass. *Proteobacteria*	*Proteobacteria*	*Bacteria*
Otu0460	0.2	0.28	0.09	0.69	0.15	55.69	*Arcobacter*	*Campylobacteraceae*	*Campylobacterales*	*Epsilonproteobacteria*	*Proteobacteria*	*Bacteria*
Otu0919	0.2	0.28	0.09	0.69	0.15	55.84	unclass. *Gammaproteobacteria*	unclass. *Gammaproteobacteria*	unclass. *Gammaproteobacteria*	*Gammaproteobacteria*	*Proteobacteria*	*Bacteria*

OTU	Av.A_i (*pH in situ*)	Av.A_i (pH 7.67)	Av.δ_i	Av.δ_i/SD	Av.δ_i %	∑Av.δ_i %	Genus	Family	Order	Class	Phylum	Domain
Otu1875	0.4	0	0.09	0.8	0.15	55.99	unclass. *Bacteria*	unclass. *Bacteria*	unclass. *Bacteria*	unclass. *Bacteria*	unclass. *Bacteria*	*Bacteria*
Otu1913	0.4	0	0.09	0.8	0.15	56.15	unclass. *Gammaproteobacteria*	unclass. *Gammaproteobacteria*	unclass. *Gammaproteobacteria*	*Gammaproteobacteria*	*Proteobacteria*	*Bacteria*
Otu0330	0	0.4	0.09	0.8	0.15	56.3	*Photobacterium*	*Vibrionaceae*	*Vibrionales*	*Gammaproteobacteria*	*Proteobacteria*	*Bacteria*
Otu0412	0	0.4	0.09	0.8	0.15	56.45	unclass. *Bacteroidetes*	unclass. *Bacteroidetes*	unclass. *Bacteroidetes*	unclass. *Bacteroidetes*	*Bacteroidetes*	*Bacteria*
Otu1273	0	0.4	0.09	0.8	0.15	56.61	unclass. *Bacteria*	unclass. *Bacteria*	unclass. *Bacteria*	unclass. *Bacteria*	unclass. *Bacteria*	*Bacteria*
Otu1823	0	0.4	0.09	0.8	0.15	56.76	unclass. *Rhodobacteraceae*	*Rhodobacteraceae*	*Rhodobacterales*	*Alphaproteobacteria*	*Proteobacteria*	*Bacteria*
Otu2091	0	0.4	0.09	0.8	0.15	56.91	*Aureispira*	*Saprospiraceae*	*Sphingobacteriales*	*Sphingobacteria*	*Bacteroidetes*	*Bacteria*
Otu0176	0	0.4	0.09	0.8	0.15	57.06	*Colwellia*	*Colwelliaceae*	*Alteromonadales*	*Gammaproteobacteria*	*Proteobacteria*	*Bacteria*
Otu1980	0	0.4	0.09	0.8	0.15	57.22	*Sulfurovum*	*Helicobacteraceae*	*Campylobacterales*	*Epsilonproteobacteria*	*Proteobacteria*	*Bacteria*
Otu1996	0	0.4	0.09	0.8	0.15	57.37	unclass. *Bacteria*	unclass. *Bacteria*	unclass. *Bacteria*	unclass. *Bacteria*	unclass. *Bacteria*	*Bacteria*
Otu0196	0	0.4	0.09	0.8	0.15	57.52	unclass. *Flavobacteriaceae*	*Flavobacteriaceae*	*Flavobacteriales*	*Flavobacteria*	*Bacteroidetes*	*Bacteria*
Otu0286	0	0.4	0.09	0.8	0.15	57.67	unclass. *Gammaproteobacteria*	unclass. *Gammaproteobacteria*	unclass. *Gammaproteobacteria*	*Gammaproteobacteria*	*Proteobacteria*	*Bacteria*
Otu1071	0	0.4	0.09	0.8	0.15	57.82	unclass. *Colwelliaceae*	*Colwelliaceae*	*Alteromonadales*	*Gammaproteobacteria*	*Proteobacteria*	*Bacteria*
Otu1990	0	0.4	0.09	0.8	0.15	57.98	unclass. *Gammaproteobacteria*	unclass. *Gammaproteobacteria*	unclass. *Gammaproteobacteria*	*Gammaproteobacteria*	*Proteobacteria*	*Bacteria*
Otu1824	0.4	0	0.09	0.8	0.15	58.13	unclass. *Gammaproteobacteria*	unclass. *Gammaproteobacteria*	unclass. *Gammaproteobacteria*	*Gammaproteobacteria*	*Proteobacteria*	*Bacteria*
Otu1841	0.4	0	0.09	0.8	0.15	58.28	unclass. *Flavobacteriaceae*	*Flavobacteriaceae*	*Flavobacteriales*	*Flavobacteria*	*Bacteroidetes*	*Bacteria*
Otu0191	0.4	0	0.09	0.8	0.15	58.43	unclass. *Proteobacteria*	unclass. *Proteobacteria*	unclass. *Proteobacteria*	unclass. *Proteobacteria*	*Proteobacteria*	*Bacteria*
Otu1532	0	0.4	0.09	0.8	0.15	58.58	unclass. *Gammaproteobacteria*	unclass. *Gammaproteobacteria*	unclass. *Gammaproteobacteria*	*Gammaproteobacteria*	*Proteobacteria*	*Bacteria*
Otu2024	0	0.4	0.09	0.8	0.15	58.74	*Desulfosarcina*	*Desulfobacteraceae*	*Desulfobacterales*	*Deltaproteobacteria*	*Proteobacteria*	*Bacteria*
Otu0157	1.75	1.46	0.09	1.01	0.15	58.88	unclass. *Gammaproteobacteria*	unclass. *Gammaproteobacteria*	unclass. *Gammaproteobacteria*	*Gammaproteobacteria*	*Proteobacteria*	*Bacteria*
Otu0119	0.35	0	0.08	0.49	0.14	59.02	*Colwellia*	*Colwelliaceae*	*Alteromonadales*	*Gammaproteobacteria*	*Proteobacteria*	*Bacteria*
Otu1883	0.35	0	0.08	0.49	0.14	59.16	unclass. *Rhodobacteraceae*	*Rhodobacteraceae*	*Rhodobacterales*	*Alphaproteobacteria*	*Proteobacteria*	*Bacteria*
Otu0249	0	0.35	0.08	0.49	0.13	59.29	unclass. *Bacteria*	unclass. *Bacteria*	unclass. *Bacteria*	unclass. *Bacteria*	unclass. *Bacteria*	*Bacteria*
Otu0305	0	0.35	0.08	0.49	0.13	59.42	unclass. *Bacteria*	unclass. *Bacteria*	unclass. *Bacteria*	unclass. *Bacteria*	unclass. *Bacteria*	*Bacteria*
Otu0108	0.2	0.2	0.08	0.67	0.12	59.55	unclass. *Colwelliaceae*	*Colwelliaceae*	*Alteromonadales*	*Gammaproteobacteria*	*Proteobacteria*	*Bacteria*
Otu0618	0.2	0.2	0.08	0.67	0.12	59.67	*Glaciecola*	*Alteromonadaceae*	*Alteromonadales*	*Gammaproteobacteria*	*Proteobacteria*	*Bacteria*
Otu1505	0.2	0.2	0.08	0.67	0.12	59.8	unclass. *Desulfobacteraceae*	*Desulfobacteraceae*	*Desulfobacterales*	*Deltaproteobacteria*	*Proteobacteria*	*Bacteria*

OTU	Av.A_i (*pH in situ*)	Av.A_i (pH 7.67)	Av.δ_i	Av.δ_i/SD	Av.δ_i %	∑Av.δ_i %	Genus	Family	Order	Class	Phylum	Domain
Otu0240	0.2	0.2	0.08	0.67	0.12	59.92	unclass. *Chromatiales*	unclass. *Chromatiales*	*Chromatiales*	*Gammaproteobacteria*	*Proteobacteria*	*Bacteria*
Otu0931	0.2	0.2	0.08	0.67	0.12	60.04	*Robiginitalea*	*Flavobacteriaceae*	*Flavobacteriales*	*Flavobacteria*	*Bacteroidetes*	*Bacteria*
Otu1017	0.2	0.2	0.08	0.67	0.12	60.17	unclass. *Actinomycetales*	unclass. *Actinomycetales*	*Actinomycetales*	*Actinobacteria*	*Actinobacteria*	*Bacteria*
Otu0331	0.2	0.2	0.08	0.67	0.12	60.29	unclass. *Gammaproteobacteria*	unclass. *Gammaproteobacteria*	unclass. *Gammaproteobacteria*	*Gammaproteobacteria*	*Proteobacteria*	*Bacteria*
Otu0421	0.2	0.2	0.08	0.67	0.12	60.42	unclass. *Gammaproteobacteria*	unclass. *Gammaproteobacteria*	unclass. *Gammaproteobacteria*	*Gammaproteobacteria*	*Proteobacteria*	*Bacteria*
Otu1953	0.2	0.2	0.08	0.67	0.12	60.54	unclass. *Gammaproteobacteria*	unclass. *Gammaproteobacteria*	unclass. *Gammaproteobacteria*	*Gammaproteobacteria*	*Proteobacteria*	*Bacteria*
Otu0314	0.2	0.2	0.08	0.67	0.12	60.66	unclass. *Flavobacteriaceae*	*Flavobacteriaceae*	*Flavobacteriales*	*Flavobacteria*	*Bacteroidetes*	*Bacteria*
Otu1889	0.2	0.2	0.08	0.67	0.12	60.79	unclass. *Gammaproteobacteria*	unclass. *Gammaproteobacteria*	unclass. *Gammaproteobacteria*	*Gammaproteobacteria*	*Proteobacteria*	*Bacteria*
Otu1890	0.2	0.2	0.08	0.67	0.12	60.91	unclass. *Bacteria*	unclass. *Bacteria*	unclass. *Bacteria*	unclass. *Bacteria*	unclass. *Bacteria*	*Bacteria*
Otu1908	0.2	0.2	0.08	0.67	0.12	61.04	unclass. *Flavobacteriaceae*	*Flavobacteriaceae*	*Flavobacteriales*	*Flavobacteria*	*Bacteroidetes*	*Bacteria*
Otu0159	0.2	0.2	0.08	0.67	0.12	61.16	unclass. *Saprospiraceae*	*Saprospiraceae*	*Sphingobacteriales*	*Sphingobacteria*	*Bacteroidetes*	*Bacteria*
Otu0765	0.2	0.2	0.08	0.67	0.12	61.28	*Pelagicoccus*	*Puniceicoccaceae*	*Puniceicoccales*	*Opitutae*	*Verrucomicrobia*	*Bacteria*
Otu1907	0.2	0.2	0.08	0.67	0.12	61.41	unclass. *Flavobacteriaceae*	*Flavobacteriaceae*	*Flavobacteriales*	*Flavobacteria*	*Bacteroidetes*	*Bacteria*
Otu0278	0.2	0.2	0.07	0.67	0.12	61.53	unclass. *Gammaproteobacteria*	unclass. *Gammaproteobacteria*	unclass. *Gammaproteobacteria*	*Gammaproteobacteria*	*Proteobacteria*	*Bacteria*
Otu0247	0.2	0.2	0.07	0.67	0.12	61.65	unclass. *Flavobacteriaceae*	*Flavobacteriaceae*	*Flavobacteriales*	*Flavobacteria*	*Bacteroidetes*	*Bacteria*
Otu0498	0.2	0.2	0.07	0.67	0.12	61.77	unclass. *Flavobacteriales*	unclass. *Flavobacteriales*	*Flavobacteriales*	*Flavobacteria*	*Bacteroidetes*	*Bacteria*
Otu0895	0.2	0.2	0.07	0.67	0.12	61.9	unclass. *Actinobacteria*	unclass. *Actinobacteria*	unclass. *Actinobacteria*	*Actinobacteria*	*Actinobacteria*	*Bacteria*
Otu0601	0.2	0.2	0.07	0.67	0.12	62.02	unclass. *Flammeovirgaceae*	*Flammeovirgaceae*	*Sphingobacteriales*	*Sphingobacteria*	*Bacteroidetes*	*Bacteria*
Otu1666	0.2	0.2	0.07	0.67	0.12	62.14	unclass. *Bacteria*	unclass. *Bacteria*	unclass. *Bacteria*	unclass. *Bacteria*	unclass. *Bacteria*	*Bacteria*
Otu0070	0.2	0.2	0.07	0.67	0.12	62.26	*Winogradskyella*	*Flavobacteriaceae*	*Flavobacteriales*	*Flavobacteria*	*Bacteroidetes*	*Bacteria*
Otu1919	0.2	0.2	0.07	0.67	0.12	62.39	unclass. *Bacteroidetes*	unclass. *Bacteroidetes*	unclass. *Bacteroidetes*	unclass. *Bacteroidetes*	*Bacteroidetes*	*Bacteria*
Otu0426	0.2	0.2	0.07	0.67	0.12	62.51	unclass. *Gammaproteobacteria*	unclass. *Gammaproteobacteria*	unclass. *Gammaproteobacteria*	*Gammaproteobacteria*	*Proteobacteria*	*Bacteria*
Otu0274	0.2	0.2	0.07	0.67	0.12	62.63	unclass. *Alphaproteobacteria*	unclass. *Alphaproteobacteria*	unclass. *Alphaproteobacteria*	*Alphaproteobacteria*	*Proteobacteria*	*Bacteria*
Otu0445	0.2	0.2	0.07	0.67	0.12	62.75	unclass. *Gammaproteobacteria*	unclass. *Gammaproteobacteria*	unclass. *Gammaproteobacteria*	*Gammaproteobacteria*	*Proteobacteria*	*Bacteria*
Otu0031	0.2	0.2	0.07	0.67	0.12	62.88	unclass. *Gammaproteobacteria*	unclass. *Gammaproteobacteria*	unclass. *Gammaproteobacteria*	*Gammaproteobacteria*	*Proteobacteria*	*Bacteria*
Otu0373	0.2	0.2	0.07	0.67	0.12	63	unclass. *Bacteria*	unclass. *Bacteria*	unclass. *Bacteria*	unclass. *Bacteria*	unclass. *Bacteria*	*Bacteria*
Otu1825	0.2	0.2	0.07	0.67	0.12	63.12	unclass. *Gammaproteobacteria*	unclass. *Gammaproteobacteria*	unclass. *Gammaproteobacteria*	*Gammaproteobacteria*	*Proteobacteria*	*Bacteria*

OTU	Av.A_i (*pH in situ*)	Av.A_i (pH 7.67)	Av.δ_i	Av.δ_i/SD	Av.δ_i %	∑Av.δ_i %	Genus	Family	Order	Class	Phylum	Domain
Otu1842	0.2	0.2	0.07	0.67	0.12	63.24	unclass. *Bacteroidetes*	unclass. *Bacteroidetes*	unclass. *Bacteroidetes*	unclass. *Bacteroidetes*	*Bacteroidetes*	*Bacteria*
Otu1105	0.2	0.2	0.07	0.67	0.12	63.36	unclass. *Proteobacteria*	unclass. *Proteobacteria*	unclass. *Proteobacteria*	unclass. *Proteobacteria*	*Proteobacteria*	*Bacteria*
Otu0404	0.2	0.2	0.07	0.67	0.12	63.48	unclass. *Flavobacteriaceae*	*Flavobacteriaceae*	*Flavobacteriales*	*Flavobacteria*	*Bacteroidetes*	*Bacteria*
Otu1245	0.2	0.2	0.07	0.67	0.12	63.61	unclass. *Gammaproteobacteria*	unclass. *Gammaproteobacteria*	unclass. *Gammaproteobacteria*	*Gammaproteobacteria*	*Proteobacteria*	*Bacteria*
Otu1359	0.2	0.2	0.07	0.67	0.12	63.73	unclass. *Gammaproteobacteria*	unclass. *Gammaproteobacteria*	unclass. *Gammaproteobacteria*	*Gammaproteobacteria*	*Proteobacteria*	*Bacteria*
Otu0167	0.2	0.2	0.07	0.67	0.12	63.85	unclass. *Rhodobacteraceae*	*Rhodobacteraceae*	*Rhodobacterales*	*Alphaproteobacteria*	*Proteobacteria*	*Bacteria*
Otu0459	0.28	0	0.07	0.49	0.11	63.96	*Croceibacter*	*Flavobacteriaceae*	*Flavobacteriales*	*Flavobacteria*	*Bacteroidetes*	*Bacteria*
Otu1188	0.28	0	0.07	0.49	0.11	64.07	unclass. *Flammeovirgaceae*	*Flammeovirgaceae*	*Sphingobacteriales*	*Sphingobacteria*	*Bacteroidetes*	*Bacteria*
Otu1383	0	0.28	0.07	0.49	0.11	64.19	unclass. *Bacteria*	unclass. *Bacteria*	unclass. *Bacteria*	unclass. *Bacteria*	unclass. *Bacteria*	*Bacteria*
Otu1916	0	0.28	0.07	0.49	0.11	64.3	unclass. *Oceanospirillaceae*	*Oceanospirillaceae*	*Oceanospirillales*	*Gammaproteobacteria*	*Proteobacteria*	*Bacteria*
Otu0490	0.28	0	0.07	0.49	0.11	64.41	unclass. *Burkholderiales*	unclass. *Burkholderiales*	*Burkholderiales*	*Betaproteobacteria*	*Proteobacteria*	*Bacteria*
Otu1274	0.28	0	0.07	0.49	0.11	64.52	unclass. *Gammaproteobacteria*	unclass. *Gammaproteobacteria*	unclass. *Gammaproteobacteria*	*Gammaproteobacteria*	*Proteobacteria*	*Bacteria*
Otu1909	0.28	0	0.07	0.49	0.11	64.62	unclass. *Bacteria*	unclass. *Bacteria*	unclass. *Bacteria*	unclass. *Bacteria*	unclass. *Bacteria*	*Bacteria*
Otu1910	0.28	0	0.07	0.49	0.11	64.73	unclass. *Gammaproteobacteria*	unclass. *Gammaproteobacteria*	unclass. *Gammaproteobacteria*	*Gammaproteobacteria*	*Proteobacteria*	*Bacteria*
Otu1922	0.28	0	0.07	0.49	0.11	64.84	unclass. *Bacteria*	unclass. *Bacteria*	unclass. *Bacteria*	unclass. *Bacteria*	unclass. *Bacteria*	*Bacteria*
Otu0422	0	0.28	0.07	0.49	0.11	64.95	unclass. *Gammaproteobacteria*	unclass. *Gammaproteobacteria*	unclass. *Gammaproteobacteria*	*Gammaproteobacteria*	*Proteobacteria*	*Bacteria*
Otu0714	0	0.28	0.07	0.49	0.11	65.06	unclass. *Alphaproteobacteria*	unclass. *Alphaproteobacteria*	unclass. *Alphaproteobacteria*	*Alphaproteobacteria*	*Proteobacteria*	*Bacteria*
Otu1967	0	0.28	0.07	0.49	0.11	65.17	unclass. *Deltaproteobacteria*	unclass. *Deltaproteobacteria*	unclass. *Deltaproteobacteria*	*Deltaproteobacteria*	*Proteobacteria*	*Bacteria*
Otu1968	0	0.28	0.07	0.49	0.11	65.28	unclass. *Saprospiraceae*	*Saprospiraceae*	*Sphingobacteriales*	*Sphingobacteria*	*Bacteroidetes*	*Bacteria*
Otu1992	0	0.28	0.07	0.49	0.11	65.38	unclass. *Desulfobacteraceae*	*Desulfobacteraceae*	*Desulfobacterales*	*Deltaproteobacteria*	*Proteobacteria*	*Bacteria*
Otu1819	0.28	0	0.07	0.49	0.11	65.49	unclass. *Bacteroidetes*	unclass. *Bacteroidetes*	unclass. *Bacteroidetes*	unclass. *Bacteroidetes*	*Bacteroidetes*	*Bacteria*
Otu0256	0.28	0	0.07	0.49	0.11	65.6	*Glaciecola*	*Alteromonadaceae*	*Alteromonadales*	*Gammaproteobacteria*	*Proteobacteria*	*Bacteria*
Otu0985	0.28	0	0.07	0.49	0.11	65.71	unclass. *Gammaproteobacteria*	unclass. *Gammaproteobacteria*	unclass. *Gammaproteobacteria*	*Gammaproteobacteria*	*Proteobacteria*	*Bacteria*
Otu0271	0.28	0	0.07	0.49	0.11	65.81	unclass. *Proteobacteria*	unclass. *Proteobacteria*	unclass. *Proteobacteria*	unclass. *Proteobacteria*	*Proteobacteria*	*Bacteria*
Otu0691	0.28	0	0.07	0.49	0.11	65.92	*Planctomyces*	*Planctomycetaceae*	*Planctomycetales*	*Planctomycetacia*	*Planctomycetes*	*Bacteria*
Otu0880	0.28	0	0.07	0.49	0.11	66.03	unclass. *Flavobacteriaceae*	*Flavobacteriaceae*	*Flavobacteriales*	*Flavobacteria*	*Bacteroidetes*	*Bacteria*
Otu1228	0.28	0	0.07	0.49	0.11	66.14	unclass. *Desulfobulbaceae*	*Desulfobulbaceae*	*Desulfobacterales*	*Deltaproteobacteria*	*Proteobacteria*	*Bacteria*

OTU	Av.A_i (*pH in situ*)	Av.A_i (pH 7.67)	Av.δ_i	Av.δ_i/SD	Av.δ_i %	∑Av.δ_i %	Genus	Family	Order	Class	Phylum	Domain
Otu1696	0.28	0	0.07	0.49	0.11	66.24	*Pelagibacter*	SAR11-clade	*Rickettsiales*	*Alphaproteobacteria*	*Proteobacteria*	*Bacteria*
Otu0316	0	0.28	0.07	0.49	0.11	66.35	unclass. *Bacteria*	unclass. *Bacteria*	unclass. *Bacteria*	unclass. *Bacteria*	unclass. *Bacteria*	*Bacteria*
Otu1156	0	0.28	0.07	0.49	0.11	66.46	unclass. *Gammaproteobacteria*	unclass. *Gammaproteobacteria*	unclass. *Gammaproteobacteria*	*Gammaproteobacteria*	*Proteobacteria*	*Bacteria*
Otu2011	0	0.28	0.07	0.49	0.11	66.56	unclass. *Bacteria*	unclass. *Bacteria*	unclass. *Bacteria*	unclass. *Bacteria*	unclass. *Bacteria*	*Bacteria*
Otu2012	0	0.28	0.07	0.49	0.11	66.67	*Neisseria*	*Neisseriaceae*	*Neisseriales*	*Betaproteobacteria*	*Proteobacteria*	*Bacteria*
Otu2032	0	0.28	0.07	0.49	0.11	66.78	unclass. *Bacteroidetes*	unclass. *Bacteroidetes*	unclass. *Bacteroidetes*	unclass. *Bacteroidetes*	*Bacteroidetes*	*Bacteria*
Otu0217	0.2	0	0.05	0.49	0.08	66.86	unclass. *Flavobacteriales*	unclass. *Flavobacteriales*	*Flavobacteriales*	*Flavobacteria*	*Bacteroidetes*	*Bacteria*
Otu0295	0.2	0	0.05	0.49	0.08	66.94	*Haliea*	*Alteromonadaceae*	*Alteromonadales*	*Gammaproteobacteria*	*Proteobacteria*	*Bacteria*
Otu0398	0.2	0	0.05	0.49	0.08	67.02	unclass. *Flavobacteriaceae*	*Flavobacteriaceae*	*Flavobacteriales*	*Flavobacteria*	*Bacteroidetes*	*Bacteria*
Otu0436	0.2	0	0.05	0.49	0.08	67.1	unclass. *Rhodobacteraceae*	*Rhodobacteraceae*	*Rhodobacterales*	*Alphaproteobacteria*	*Proteobacteria*	*Bacteria*
Otu0479	0.2	0	0.05	0.49	0.08	67.17	unclass. *Rhodospirillaceae*	*Rhodospirillaceae*	*Rhodospirillales*	*Alphaproteobacteria*	*Proteobacteria*	*Bacteria*
Otu0718	0.2	0	0.05	0.49	0.08	67.25	*Aureispira*	*Saprospiraceae*	*Sphingobacteriales*	*Sphingobacteria*	*Bacteroidetes*	*Bacteria*
Otu1008	0.2	0	0.05	0.49	0.08	67.33	unclass. *Flavobacteriaceae*	*Flavobacteriaceae*	*Flavobacteriales*	*Flavobacteria*	*Bacteroidetes*	*Bacteria*
Otu1027	0.2	0	0.05	0.49	0.08	67.41	unclass. *Epsilonproteobacteria*	unclass. *Epsilonproteobacteria*	unclass. *Epsilonproteobacteria*	*Epsilonproteobacteria*	*Proteobacteria*	*Bacteria*
Otu1137	0.2	0	0.05	0.49	0.08	67.49	unclass. *Gammaproteobacteria*	unclass. *Gammaproteobacteria*	unclass. *Gammaproteobacteria*	*Gammaproteobacteria*	*Proteobacteria*	*Bacteria*
Otu1180	0.2	0	0.05	0.49	0.08	67.57	unclass. *Nitrosomonadaceae*	*Nitrosomonadaceae*	*Nitrosomonadales*	*Betaproteobacteria*	*Proteobacteria*	*Bacteria*
Otu1211	0.2	0	0.05	0.49	0.08	67.65	unclass. *Oceanospirillaceae*	*Oceanospirillaceae*	*Oceanospirillales*	*Gammaproteobacteria*	*Proteobacteria*	*Bacteria*
Otu1587	0.2	0	0.05	0.49	0.08	67.73	unclass. *Bacteria*	unclass. *Bacteria*	unclass. *Bacteria*	unclass. *Bacteria*	unclass. *Bacteria*	*Bacteria*
Otu1774	0.2	0	0.05	0.49	0.08	67.81	unclass. *Flavobacteriaceae*	*Flavobacteriaceae*	*Flavobacteriales*	*Flavobacteria*	*Bacteroidetes*	*Bacteria*
Otu1880	0.2	0	0.05	0.49	0.08	67.89	unclass. *Bacteria*	unclass. *Bacteria*	unclass. *Bacteria*	unclass. *Bacteria*	unclass. *Bacteria*	*Bacteria*
Otu1881	0.2	0	0.05	0.49	0.08	67.97	unclass. *Bacteria*	unclass. *Bacteria*	unclass. *Bacteria*	unclass. *Bacteria*	unclass. *Bacteria*	*Bacteria*
Otu1882	0.2	0	0.05	0.49	0.08	68.05	*Truepera*	*Trueperaceae*	*Deinococcales*	*Deinococci*	*Deinococcus-Thermus*	*Bacteria*
Otu1885	0.2	0	0.05	0.49	0.08	68.13	unclass. *Gammaproteobacteria*	unclass. *Gammaproteobacteria*	unclass. *Gammaproteobacteria*	*Gammaproteobacteria*	*Proteobacteria*	*Bacteria*
Otu1886	0.2	0	0.05	0.49	0.08	68.21	unclass. *Bacteria*	unclass. *Bacteria*	unclass. *Bacteria*	unclass. *Bacteria*	unclass. *Bacteria*	*Bacteria*
Otu1891	0.2	0	0.05	0.49	0.08	68.29	unclass. *Flavobacteriales*	unclass. *Flavobacteriales*	*Flavobacteriales*	*Flavobacteria*	*Bacteroidetes*	*Bacteria*
Otu1892	0.2	0	0.05	0.49	0.08	68.36	unclass. *Bacteroidetes*	unclass. *Bacteroidetes*	unclass. *Bacteroidetes*	unclass. *Bacteroidetes*	*Bacteroidetes*	*Bacteria*
Otu1896	0.2	0	0.05	0.49	0.08	68.44	unclass. *Gammaproteobacteria*	unclass. *Gammaproteobacteria*	unclass. *Gammaproteobacteria*	*Gammaproteobacteria*	*Proteobacteria*	*Bacteria*

OTU	Av.A_i (*pH in situ*)	Av.A_i (pH 7.67)	Av.δ_i	Av.δ_i/SD	Av.δ_i %	∑Av.δ_i %	Genus	Family	Order	Class	Phylum	Domain
Otu1898	0.2	0	0.05	0.49	0.08	68.52	*Winogradskyella*	*Flavobacteriaceae*	*Flavobacteriales*	*Flavobacteria*	*Bacteroidetes*	*Bacteria*
Otu1899	0.2	0	0.05	0.49	0.08	68.6	unclass. *Ectothiorhodospiraceae*	*Ectothiorhodospiraceae*	*Chromatiales*	*Gammaproteobacteria*	*Proteobacteria*	*Bacteria*
Otu1900	0.2	0	0.05	0.49	0.08	68.68	unclass. *Flavobacteriaceae*	*Flavobacteriaceae*	*Flavobacteriales*	*Flavobacteria*	*Bacteroidetes*	*Bacteria*
Otu1901	0.2	0	0.05	0.49	0.08	68.76	*Glaciecola*	*Alteromonadaceae*	*Alteromonadales*	*Gammaproteobacteria*	*Proteobacteria*	*Bacteria*
Otu1902	0.2	0	0.05	0.49	0.08	68.84	unclass. *Bacteria*	unclass. *Bacteria*	unclass. *Bacteria*	unclass. *Bacteria*	unclass. *Bacteria*	*Bacteria*
Otu1903	0.2	0	0.05	0.49	0.08	68.92	unclass. *Flavobacteriales*	unclass. *Flavobacteriales*	*Flavobacteriales*	*Flavobacteria*	*Bacteroidetes*	*Bacteria*
Otu1904	0.2	0	0.05	0.49	0.08	69	unclass. *Flavobacteriales*	unclass. *Flavobacteriales*	*Flavobacteriales*	*Flavobacteria*	*Bacteroidetes*	*Bacteria*
Otu1905	0.2	0	0.05	0.49	0.08	69.08	*Dasania*	*Pseudomonadales_incertae_sedis*	*Pseudomonadales*	*Gammaproteobacteria*	*Proteobacteria*	*Bacteria*
Otu1906	0.2	0	0.05	0.49	0.08	69.16	unclass. *Bacteria*	unclass. *Bacteria*	unclass. *Bacteria*	unclass. *Bacteria*	unclass. *Bacteria*	*Bacteria*
Otu0080	0	0.2	0.05	0.49	0.08	69.24	*Polaribacter*	*Flavobacteriaceae*	*Flavobacteriales*	*Flavobacteria*	*Bacteroidetes*	*Bacteria*
Otu0161	0	0.2	0.05	0.49	0.08	69.32	unclass. *Flavobacteriales*	unclass. *Flavobacteriales*	*Flavobacteriales*	*Flavobacteria*	*Bacteroidetes*	*Bacteria*
Otu0288	0	0.2	0.05	0.49	0.08	69.4	*Ulvibacter*	*Flavobacteriaceae*	*Flavobacteriales*	*Flavobacteria*	*Bacteroidetes*	*Bacteria*
Otu0329	0	0.2	0.05	0.49	0.08	69.48	*Gaetbulibacter*	*Flavobacteriaceae*	*Flavobacteriales*	*Flavobacteria*	*Bacteroidetes*	*Bacteria*
Otu0372	0	0.2	0.05	0.49	0.08	69.55	unclass. *Flavobacteriaceae*	*Flavobacteriaceae*	*Flavobacteriales*	*Flavobacteria*	*Bacteroidetes*	*Bacteria*
Otu0377	0	0.2	0.05	0.49	0.08	69.63	*Reichenbachiella*	*Flammeovirgaceae*	*Sphingobacteriales*	*Sphingobacteria*	*Bacteroidetes*	*Bacteria*
Otu0446	0	0.2	0.05	0.49	0.08	69.71	unclass. *Bacteroidetes*	unclass. *Bacteroidetes*	unclass. *Bacteroidetes*	unclass. *Bacteroidetes*	*Bacteroidetes*	*Bacteria*
Otu0481	0	0.2	0.05	0.49	0.08	69.79	*Haliea*	*Alteromonadaceae*	*Alteromonadales*	*Gammaproteobacteria*	*Proteobacteria*	*Bacteria*
Otu0482	0	0.2	0.05	0.49	0.08	69.87	unclass. *Microbacteriaceae*	*Microbacteriaceae*	*Actinomycetales*	*Actinobacteria*	*Actinobacteria*	*Bacteria*
Otu0724	0	0.2	0.05	0.49	0.08	69.95	unclass. *Bacteria*	unclass. *Bacteria*	unclass. *Bacteria*	unclass. *Bacteria*	unclass. *Bacteria*	*Bacteria*
Otu1328	0	0.2	0.05	0.49	0.08	70.03	unclass. *Gammaproteobacteria*	unclass. *Gammaproteobacteria*	unclass. *Gammaproteobacteria*	*Gammaproteobacteria*	*Proteobacteria*	*Bacteria*
Otu1371	0	0.2	0.05	0.49	0.08	70.11	*Haliea*	*Alteromonadaceae*	*Alteromonadales*	*Gammaproteobacteria*	*Proteobacteria*	*Bacteria*
Otu1581	0	0.2	0.05	0.49	0.08	70.19	unclass. *Deltaproteobacteria*	unclass. *Deltaproteobacteria*	unclass. *Deltaproteobacteria*	*Deltaproteobacteria*	*Proteobacteria*	*Bacteria*
Otu1688	0	0.2	0.05	0.49	0.08	70.27	unclass. *Saprospiraceae*	*Saprospiraceae*	*Sphingobacteriales*	*Sphingobacteria*	*Bacteroidetes*	*Bacteria*
Otu1749	0	0.2	0.05	0.49	0.08	70.35	unclass. *Gammaproteobacteria*	unclass. *Gammaproteobacteria*	unclass. *Gammaproteobacteria*	*Gammaproteobacteria*	*Proteobacteria*	*Bacteria*
Otu1895	0	0.2	0.05	0.49	0.08	70.43	unclass. *Flavobacteriales*	unclass. *Flavobacteriales*	*Flavobacteriales*	*Flavobacteria*	*Bacteroidetes*	*Bacteria*
Otu2043	0	0.2	0.05	0.49	0.08	70.51	*Cellulophaga*	*Flavobacteriaceae*	*Flavobacteriales*	*Flavobacteria*	*Bacteroidetes*	*Bacteria*
Otu2044	0	0.2	0.05	0.49	0.08	70.59	unclass. *Alphaproteobacteria*	unclass. *Alphaproteobacteria*	unclass. *Alphaproteobacteria*	*Alphaproteobacteria*	*Proteobacteria*	*Bacteria*

OTU	Av.A_i (*pH in situ*)	Av.A_i (pH 7.67)	Av.δ_i	Av.δ_i/SD	Av.δ_i %	∑Av.δ_i %	Genus	Family	Order	Class	Phylum	Domain
Otu2045	0	0.2	0.05	0.49	0.08	70.66	unclass. *Bacteria*	unclass. *Bacteria*	unclass. *Bacteria*	unclass. *Bacteria*	unclass. *Bacteria*	*Bacteria*
Otu2046	0	0.2	0.05	0.49	0.08	70.74	unclass. *Bacteria*	unclass. *Bacteria*	unclass. *Bacteria*	unclass. *Bacteria*	unclass. *Bacteria*	*Bacteria*
Otu2047	0	0.2	0.05	0.49	0.08	70.82	unclass. *Bacteria*	unclass. *Bacteria*	unclass. *Bacteria*	unclass. *Bacteria*	unclass. *Bacteria*	*Bacteria*
Otu2048	0	0.2	0.05	0.49	0.08	70.9	*Pseudonocardia*	*Pseudonocardiaceae*	*Actinomycetales*	*Actinobacteria*	*Actinobacteria*	*Bacteria*
Otu2049	0	0.2	0.05	0.49	0.08	70.98	unclass. *Gammaproteobacteria*	unclass. *Gammaproteobacteria*	unclass. *Gammaproteobacteria*	*Gammaproteobacteria*	*Proteobacteria*	*Bacteria*
Otu2050	0	0.2	0.05	0.49	0.08	71.06	unclass. *Bacteria*	unclass. *Bacteria*	unclass. *Bacteria*	unclass. *Bacteria*	unclass. *Bacteria*	*Bacteria*
Otu2051	0	0.2	0.05	0.49	0.08	71.14	unclass. *Flavobacteriales*	unclass. *Flavobacteriales*	*Flavobacteriales*	*Flavobacteria*	*Bacteroidetes*	*Bacteria*
Otu2052	0	0.2	0.05	0.49	0.08	71.22	*Sneathiella*	*Sneathiellaceae*	*Sneathiellales*	*Alphaproteobacteria*	*Proteobacteria*	*Bacteria*
Otu2053	0	0.2	0.05	0.49	0.08	71.3	unclass. *Gammaproteobacteria*	unclass. *Gammaproteobacteria*	unclass. *Gammaproteobacteria*	*Gammaproteobacteria*	*Proteobacteria*	*Bacteria*
Otu2054	0	0.2	0.05	0.49	0.08	71.38	unclass. *Proteobacteria*	unclass. *Proteobacteria*	unclass. *Proteobacteria*	unclass. *Proteobacteria*	*Proteobacteria*	*Bacteria*
Otu2055	0	0.2	0.05	0.49	0.08	71.46	*Glaciecola*	*Alteromonadaceae*	*Alteromonadales*	*Gammaproteobacteria*	*Proteobacteria*	*Bacteria*
Otu2056	0	0.2	0.05	0.49	0.08	71.54	unclass. *Bacteroidetes*	unclass. *Bacteroidetes*	unclass. *Bacteroidetes*	unclass. *Bacteroidetes*	*Bacteroidetes*	*Bacteria*
Otu2057	0	0.2	0.05	0.49	0.08	71.62	unclass. *Gammaproteobacteria*	unclass. *Gammaproteobacteria*	unclass. *Gammaproteobacteria*	*Gammaproteobacteria*	*Proteobacteria*	*Bacteria*
Otu2058	0	0.2	0.05	0.49	0.08	71.7	unclass. *Rhodobacteraceae*	*Rhodobacteraceae*	*Rhodobacterales*	*Alphaproteobacteria*	*Proteobacteria*	*Bacteria*
Otu2059	0	0.2	0.05	0.49	0.08	71.77	unclass. *Rhizobiales*	unclass. *Rhizobiales*	*Rhizobiales*	*Alphaproteobacteria*	*Proteobacteria*	*Bacteria*
Otu2060	0	0.2	0.05	0.49	0.08	71.85	unclass. *Chromatiales*	unclass. *Chromatiales*	*Chromatiales*	*Gammaproteobacteria*	*Proteobacteria*	*Bacteria*
Otu2061	0	0.2	0.05	0.49	0.08	71.93	unclass. *Anaerolineaceae*	*Anaerolineaceae*	*Anaerolineales*	*Anaerolineae*	*Chloroflexi*	*Bacteria*
Otu2062	0	0.2	0.05	0.49	0.08	72.01	unclass. *Rhodobacteraceae*	*Rhodobacteraceae*	*Rhodobacterales*	*Alphaproteobacteria*	*Proteobacteria*	*Bacteria*
Otu2063	0	0.2	0.05	0.49	0.08	72.09	unclass. *Actinomycetales*	unclass. *Actinomycetales*	*Actinomycetales*	*Actinobacteria*	*Actinobacteria*	*Bacteria*
Otu2064	0	0.2	0.05	0.49	0.08	72.17	unclass. *Gammaproteobacteria*	unclass. *Gammaproteobacteria*	unclass. *Gammaproteobacteria*	*Gammaproteobacteria*	*Proteobacteria*	*Bacteria*
Otu2065	0	0.2	0.05	0.49	0.08	72.25	unclass. *Bacteria*	unclass. *Bacteria*	unclass. *Bacteria*	unclass. *Bacteria*	unclass. *Bacteria*	*Bacteria*
Otu2066	0	0.2	0.05	0.49	0.08	72.33	unclass. *Sphingobacteriales*	unclass. *Sphingobacteriales*	*Sphingobacteriales*	*Sphingobacteria*	*Bacteroidetes*	*Bacteria*
Otu0011	0.2	0	0.05	0.49	0.08	72.41	unclass. *Flavobacteriaceae*	*Flavobacteriaceae*	*Flavobacteriales*	*Flavobacteria*	*Bacteroidetes*	*Bacteria*
Otu0088	0.2	0	0.05	0.49	0.08	72.48	unclass. *Flavobacteriaceae*	*Flavobacteriaceae*	*Flavobacteriales*	*Flavobacteria*	*Bacteroidetes*	*Bacteria*
Otu0150	0.2	0	0.05	0.49	0.08	72.56	unclass. *Bacteria*	unclass. *Bacteria*	unclass. *Bacteria*	unclass. *Bacteria*	unclass. *Bacteria*	*Bacteria*
Otu0221	0.2	0	0.05	0.49	0.08	72.64	unclass. *Flavobacteriaceae*	*Flavobacteriaceae*	*Flavobacteriales*	*Flavobacteria*	*Bacteroidetes*	*Bacteria*
Otu0229	0.2	0	0.05	0.49	0.08	72.71	unclass. *Saprospiraceae*	*Saprospiraceae*	*Sphingobacteriales*	*Sphingobacteria*	*Bacteroidetes*	*Bacteria*

OTU	Av.A_i (*pH in situ*)	Av.A_i (pH 7.67)	Av.δ_i	Av.δ_i/SD	Av.δ_i %	∑Av.δ_i %	Genus	Family	Order	Class	Phylum	Domain
Otu0231	0.2	0	0.05	0.49	0.08	72.79	unclass. *Gammaproteobacteria*	unclass. *Gammaproteobacteria*	unclass. *Gammaproteobacteria*	*Gammaproteobacteria*	*Proteobacteria*	*Bacteria*
Otu0238	0.2	0	0.05	0.49	0.08	72.87	*Haliea*	*Alteromonadaceae*	*Alteromonadales*	*Gammaproteobacteria*	*Proteobacteria*	*Bacteria*
Otu0242	0.2	0	0.05	0.49	0.08	72.95	unclass. *Gammaproteobacteria*	unclass. *Gammaproteobacteria*	unclass. *Gammaproteobacteria*	*Gammaproteobacteria*	*Proteobacteria*	*Bacteria*
Otu0302	0.2	0	0.05	0.49	0.08	73.02	*Colwellia*	*Colwelliaceae*	*Alteromonadales*	*Gammaproteobacteria*	*Proteobacteria*	*Bacteria*
Otu0334	0.2	0	0.05	0.49	0.08	73.1	unclass. *Flavobacteriaceae*	*Flavobacteriaceae*	*Flavobacteriales*	*Flavobacteria*	*Bacteroidetes*	*Bacteria*
Otu0335	0.2	0	0.05	0.49	0.08	73.18	unclass. *Flavobacteriaceae*	*Flavobacteriaceae*	*Flavobacteriales*	*Flavobacteria*	*Bacteroidetes*	*Bacteria*
Otu0565	0.2	0	0.05	0.49	0.08	73.25	*Staphylococcus*	*Staphylococcaceae*	*Bacillales*	*Bacilli*	*Firmicutes*	*Bacteria*
Otu0760	0.2	0	0.05	0.49	0.08	73.33	unclass. *Bacteria*	unclass. *Bacteria*	unclass. *Bacteria*	unclass. *Bacteria*	unclass. *Bacteria*	*Bacteria*
Otu0780	0.2	0	0.05	0.49	0.08	73.41	unclass. *Gammaproteobacteria*	unclass. *Gammaproteobacteria*	unclass. *Gammaproteobacteria*	*Gammaproteobacteria*	*Proteobacteria*	*Bacteria*
Otu0798	0.2	0	0.05	0.49	0.08	73.48	unclass. *Gammaproteobacteria*	unclass. *Gammaproteobacteria*	unclass. *Gammaproteobacteria*	*Gammaproteobacteria*	*Proteobacteria*	*Bacteria*
Otu1052	0.2	0	0.05	0.49	0.08	73.56	unclass. *Desulfobacteraceae*	*Desulfobacteraceae*	*Desulfobacterales*	*Deltaproteobacteria*	*Proteobacteria*	*Bacteria*
Otu1115	0.2	0	0.05	0.49	0.08	73.64	*Marinobacter*	*Alteromonadaceae*	*Alteromonadales*	*Gammaproteobacteria*	*Proteobacteria*	*Bacteria*
Otu1204	0.2	0	0.05	0.49	0.08	73.71	unclass. *Bacteroidetes*	unclass. *Bacteroidetes*	unclass. *Bacteroidetes*	unclass. *Bacteroidetes*	*Bacteroidetes*	*Bacteria*
Otu1246	0.2	0	0.05	0.49	0.08	73.79	unclass. *Gammaproteobacteria*	unclass. *Gammaproteobacteria*	unclass. *Gammaproteobacteria*	*Gammaproteobacteria*	*Proteobacteria*	*Bacteria*
Otu1403	0.2	0	0.05	0.49	0.08	73.87	unclass. *Bacteroidetes*	unclass. *Bacteroidetes*	unclass. *Bacteroidetes*	unclass. *Bacteroidetes*	*Bacteroidetes*	*Bacteria*
Otu1620	0.2	0	0.05	0.49	0.08	73.95	*Nitrospira*	*Nitrospiraceae*	*Nitrospirales*	*Nitrospira*	*Nitrospira*	*Bacteria*
Otu1741	0.2	0	0.05	0.49	0.08	74.02	*Pelagibacter*	SAR11-clade	*Rickettsiales*	*Alphaproteobacteria*	*Proteobacteria*	*Bacteria*
Otu1775	0.2	0	0.05	0.49	0.08	74.1	unclass. *Bacteria*	unclass. *Bacteria*	unclass. *Bacteria*	unclass. *Bacteria*	unclass. *Bacteria*	*Bacteria*
Otu1894	0.2	0	0.05	0.49	0.08	74.18	*Colwellia*	*Colwelliaceae*	*Alteromonadales*	*Gammaproteobacteria*	*Proteobacteria*	*Bacteria*
Otu1911	0.2	0	0.05	0.49	0.08	74.25	unclass. *Flavobacteriales*	unclass. *Flavobacteriales*	*Flavobacteriales*	*Flavobacteria*	*Bacteroidetes*	*Bacteria*
Otu1912	0.2	0	0.05	0.49	0.08	74.33	*Pelagibacter*	SAR11-clade	*Rickettsiales*	*Alphaproteobacteria*	*Proteobacteria*	*Bacteria*
Otu1914	0.2	0	0.05	0.49	0.08	74.41	*Microscilla*	*Cytophagaceae*	*Sphingobacteriales*	*Sphingobacteria*	*Bacteroidetes*	*Bacteria*
Otu1915	0.2	0	0.05	0.49	0.08	74.48	unclass. *Proteobacteria*	unclass. *Proteobacteria*	unclass. *Proteobacteria*	unclass. *Proteobacteria*	*Proteobacteria*	*Bacteria*
Otu1917	0.2	0	0.05	0.49	0.08	74.56	unclass. *Bacteria*	unclass. *Bacteria*	unclass. *Bacteria*	unclass. *Bacteria*	unclass. *Bacteria*	*Bacteria*
Otu1918	0.2	0	0.05	0.49	0.08	74.64	unclass. *Flavobacteriaceae*	*Flavobacteriaceae*	*Flavobacteriales*	*Flavobacteria*	*Bacteroidetes*	*Bacteria*
Otu1921	0.2	0	0.05	0.49	0.08	74.72	unclass. *Bacteria*	unclass. *Bacteria*	unclass. *Bacteria*	unclass. *Bacteria*	unclass. *Bacteria*	*Bacteria*
Otu1924	0.2	0	0.05	0.49	0.08	74.79	unclass. *Bacteria*	unclass. *Bacteria*	unclass. *Bacteria*	unclass. *Bacteria*	unclass. *Bacteria*	*Bacteria*

OTU	Av.A_i (*pH in situ*)	Av.A_i (pH 7.67)	Av.δ_i	Av.δ_i/SD	Av.δ_i %	∑Av.δ_i %	Genus	Family	Order	Class	Phylum	Domain
Otu1925	0.2	0	0.05	0.49	0.08	74.87	unclass. *Gammaproteobacteria*	unclass. *Gammaproteobacteria*	unclass. *Gammaproteobacteria*	*Gammaproteobacteria*	*Proteobacteria*	*Bacteria*
Otu1926	0.2	0	0.05	0.49	0.08	74.95	unclass. *Flavobacteriaceae*	*Flavobacteriaceae*	*Flavobacteriales*	*Flavobacteria*	*Bacteroidetes*	*Bacteria*
Otu1928	0.2	0	0.05	0.49	0.08	75.02	unclass. *Flavobacteriales*	unclass. *Flavobacteriales*	*Flavobacteriales*	*Flavobacteria*	*Bacteroidetes*	*Bacteria*
Otu1929	0.2	0	0.05	0.49	0.08	75.1	unclass. *OD1*	unclass. *OD1*	unclass. *OD1*	unclass. *OD1*	*OD1*	*Bacteria*
Otu1930	0.2	0	0.05	0.49	0.08	75.18	unclass. *Flavobacteriaceae*	*Flavobacteriaceae*	*Flavobacteriales*	*Flavobacteria*	*Bacteroidetes*	*Bacteria*
Otu1932	0.2	0	0.05	0.49	0.08	75.25	unclass. *Bacteria*	unclass. *Bacteria*	unclass. *Bacteria*	unclass. *Bacteria*	unclass. *Bacteria*	*Bacteria*
Otu1933	0.2	0	0.05	0.49	0.08	75.33	unclass. *Flavobacteriaceae*	*Flavobacteriaceae*	*Flavobacteriales*	*Flavobacteria*	*Bacteroidetes*	*Bacteria*
Otu1934	0.2	0	0.05	0.49	0.08	75.41	unclass. *Flavobacteriaceae*	*Flavobacteriaceae*	*Flavobacteriales*	*Flavobacteria*	*Bacteroidetes*	*Bacteria*
Otu1936	0.2	0	0.05	0.49	0.08	75.49	unclass. *Gammaproteobacteria*	unclass. *Gammaproteobacteria*	unclass. *Gammaproteobacteria*	*Gammaproteobacteria*	*Proteobacteria*	*Bacteria*
Otu1938	0.2	0	0.05	0.49	0.08	75.56	unclass. *Planctomycetaceae*	*Planctomycetaceae*	*Planctomycetales*	*Planctomycetacia*	*Planctomycetes*	*Bacteria*
Otu0102	0	0.2	0.05	0.49	0.08	75.64	unclass. *Flavobacteriaceae*	*Flavobacteriaceae*	*Flavobacteriales*	*Flavobacteria*	*Bacteroidetes*	*Bacteria*
Otu0251	0	0.2	0.05	0.49	0.08	75.72	unclass. *Bacteroidetes*	unclass. *Bacteroidetes*	unclass. *Bacteroidetes*	unclass. *Bacteroidetes*	*Bacteroidetes*	*Bacteria*
Otu0258	0	0.2	0.05	0.49	0.08	75.79	*Haliea*	*Alteromonadaceae*	*Alteromonadales*	*Gammaproteobacteria*	*Proteobacteria*	*Bacteria*
Otu0280	0	0.2	0.05	0.49	0.08	75.87	*Marinobacter*	*Alteromonadaceae*	*Alteromonadales*	*Gammaproteobacteria*	*Proteobacteria*	*Bacteria*
Otu0300	0	0.2	0.05	0.49	0.08	75.95	unclass. *Gammaproteobacteria*	unclass. *Gammaproteobacteria*	unclass. *Gammaproteobacteria*	*Gammaproteobacteria*	*Proteobacteria*	*Bacteria*
Otu0382	0	0.2	0.05	0.49	0.08	76.02	unclass. *Flavobacteriales*	unclass. *Flavobacteriales*	*Flavobacteriales*	*Flavobacteria*	*Bacteroidetes*	*Bacteria*
Otu0670	0	0.2	0.05	0.49	0.08	76.1	unclass. *Flavobacteriaceae*	*Flavobacteriaceae*	*Flavobacteriales*	*Flavobacteria*	*Bacteroidetes*	*Bacteria*
Otu0721	0	0.2	0.05	0.49	0.08	76.18	unclass. *Alphaproteobacteria*	unclass. *Alphaproteobacteria*	unclass. *Alphaproteobacteria*	*Alphaproteobacteria*	*Proteobacteria*	*Bacteria*
Otu0857	0	0.2	0.05	0.49	0.08	76.25	unclass. *Flavobacteriaceae*	*Flavobacteriaceae*	*Flavobacteriales*	*Flavobacteria*	*Bacteroidetes*	*Bacteria*
Otu1157	0	0.2	0.05	0.49	0.08	76.33	unclass. *Flavobacteriaceae*	*Flavobacteriaceae*	*Flavobacteriales*	*Flavobacteria*	*Bacteroidetes*	*Bacteria*
Otu1332	0	0.2	0.05	0.49	0.08	76.41	unclass. *Gammaproteobacteria*	unclass. *Gammaproteobacteria*	unclass. *Gammaproteobacteria*	*Gammaproteobacteria*	*Proteobacteria*	*Bacteria*
Otu1341	0	0.2	0.05	0.49	0.08	76.49	unclass. *Gammaproteobacteria*	unclass. *Gammaproteobacteria*	unclass. *Gammaproteobacteria*	*Gammaproteobacteria*	*Proteobacteria*	*Bacteria*
Otu1556	0	0.2	0.05	0.49	0.08	76.56	unclass. *Sphingobacteriales*	unclass. *Sphingobacteriales*	*Sphingobacteriales*	*Sphingobacteria*	*Bacteroidetes*	*Bacteria*
Otu1674	0	0.2	0.05	0.49	0.08	76.64	unclass. *Bacteria*	unclass. *Bacteria*	unclass. *Bacteria*	unclass. *Bacteria*	unclass. *Bacteria*	*Bacteria*
Otu1695	0	0.2	0.05	0.49	0.08	76.72	*Balneola*	*Chitinophagaceae*	*Sphingobacteriales*	*Sphingobacteria*	*Bacteroidetes*	*Bacteria*
Otu1758	0	0.2	0.05	0.49	0.08	76.79	unclass. *Deltaproteobacteria*	unclass. *Deltaproteobacteria*	unclass. *Deltaproteobacteria*	*Deltaproteobacteria*	*Proteobacteria*	*Bacteria*
Otu1769	0	0.2	0.05	0.49	0.08	76.87	unclass. *Flavobacteriaceae*	*Flavobacteriaceae*	*Flavobacteriales*	*Flavobacteria*	*Bacteroidetes*	*Bacteria*

OTU	Av.A_i (*pH in situ*)	Av.A_i (pH 7.67)	Av.δ_i	Av.δ_i/SD	Av.δ_i %	∑Av.δ_i %	Genus	Family	Order	Class	Phylum	Domain
Otu1791	0	0.2	0.05	0.49	0.08	76.95	*Colwellia*	*Colwelliaceae*	*Alteromonadales*	*Gammaproteobacteria*	*Proteobacteria*	*Bacteria*
Otu2068	0	0.2	0.05	0.49	0.08	77.02	unclass. *Alphaproteobacteria*	unclass. *Alphaproteobacteria*	unclass. *Alphaproteobacteria*	*Alphaproteobacteria*	*Proteobacteria*	*Bacteria*
Otu2069	0	0.2	0.05	0.49	0.08	77.1	*Colwellia*	*Colwelliaceae*	*Alteromonadales*	*Gammaproteobacteria*	*Proteobacteria*	*Bacteria*
Otu2070	0	0.2	0.05	0.49	0.08	77.18	unclass. *Bacteria*	unclass. *Bacteria*	unclass. *Bacteria*	unclass. *Bacteria*	unclass. *Bacteria*	*Bacteria*
Otu2071	0	0.2	0.05	0.49	0.08	77.25	unclass. *Bacteria*	unclass. *Bacteria*	unclass. *Bacteria*	unclass. *Bacteria*	unclass. *Bacteria*	*Bacteria*
Otu2072	0	0.2	0.05	0.49	0.08	77.33	unclass. *Desulfobacteraceae*	*Desulfobacteraceae*	*Desulfobacterales*	*Deltaproteobacteria*	*Proteobacteria*	*Bacteria*
Otu2073	0	0.2	0.05	0.49	0.08	77.41	*Rothia*	*Micrococcaceae*	*Actinomycetales*	*Actinobacteria*	*Actinobacteria*	*Bacteria*
Otu2074	0	0.2	0.05	0.49	0.08	77.49	unclass. *Bacteria*	unclass. *Bacteria*	unclass. *Bacteria*	unclass. *Bacteria*	unclass. *Bacteria*	*Bacteria*
Otu2075	0	0.2	0.05	0.49	0.08	77.56	unclass. *Chromatiales*	unclass. *Chromatiales*	*Chromatiales*	*Gammaproteobacteria*	*Proteobacteria*	*Bacteria*
Otu2076	0	0.2	0.05	0.49	0.08	77.64	unclass. *Sphingobacteriales*	unclass. *Sphingobacteriales*	*Sphingobacteriales*	*Sphingobacteria*	*Bacteroidetes*	*Bacteria*
Otu2077	0	0.2	0.05	0.49	0.08	77.72	unclass. *Bacteroidetes*	unclass. *Bacteroidetes*	unclass. *Bacteroidetes*	unclass. *Bacteroidetes*	*Bacteroidetes*	*Bacteria*
Otu2078	0	0.2	0.05	0.49	0.08	77.79	unclass. *Proteobacteria*	unclass. *Proteobacteria*	unclass. *Proteobacteria*	unclass. *Proteobacteria*	*Proteobacteria*	*Bacteria*
Otu2079	0	0.2	0.05	0.49	0.08	77.87	unclass. *Bacteria*	unclass. *Bacteria*	unclass. *Bacteria*	unclass. *Bacteria*	unclass. *Bacteria*	*Bacteria*
Otu2080	0	0.2	0.05	0.49	0.08	77.95	unclass. *Bacteria*	unclass. *Bacteria*	unclass. *Bacteria*	unclass. *Bacteria*	unclass. *Bacteria*	*Bacteria*
Otu2081	0	0.2	0.05	0.49	0.08	78.02	*Reichenbachiella*	*Flammeovirgaceae*	*Sphingobacteriales*	*Sphingobacteria*	*Bacteroidetes*	*Bacteria*
Otu2082	0	0.2	0.05	0.49	0.08	78.1	unclass. *Bacteria*	unclass. *Bacteria*	unclass. *Bacteria*	unclass. *Bacteria*	unclass. *Bacteria*	*Bacteria*
Otu2083	0	0.2	0.05	0.49	0.08	78.18	unclass. *Proteobacteria*	unclass. *Proteobacteria*	unclass. *Proteobacteria*	unclass. *Proteobacteria*	*Proteobacteria*	*Bacteria*
Otu2084	0	0.2	0.05	0.49	0.08	78.25	unclass. *Bacteroidetes*	unclass. *Bacteroidetes*	unclass. *Bacteroidetes*	unclass. *Bacteroidetes*	*Bacteroidetes*	*Bacteria*
Otu2085	0	0.2	0.05	0.49	0.08	78.33	unclass. *Bacteria*	unclass. *Bacteria*	unclass. *Bacteria*	unclass. *Bacteria*	unclass. *Bacteria*	*Bacteria*
Otu2086	0	0.2	0.05	0.49	0.08	78.41	unclass. *Proteobacteria*	unclass. *Proteobacteria*	unclass. *Proteobacteria*	unclass. *Proteobacteria*	*Proteobacteria*	*Bacteria*
Otu2087	0	0.2	0.05	0.49	0.08	78.48	unclass. *Bacteria*	unclass. *Bacteria*	unclass. *Bacteria*	unclass. *Bacteria*	unclass. *Bacteria*	*Bacteria*
Otu2088	0	0.2	0.05	0.49	0.08	78.56	unclass. *Bacteria*	unclass. *Bacteria*	unclass. *Bacteria*	unclass. *Bacteria*	unclass. *Bacteria*	*Bacteria*
Otu2089	0	0.2	0.05	0.49	0.08	78.64	*Devosia*	*Hyphomicrobiaceae*	*Rhizobiales*	*Alphaproteobacteria*	*Proteobacteria*	*Bacteria*
Otu2090	0	0.2	0.05	0.49	0.08	78.72	unclass. *Bacteroidetes*	unclass. *Bacteroidetes*	unclass. *Bacteroidetes*	unclass. *Bacteroidetes*	*Bacteroidetes*	*Bacteria*
Otu2092	0	0.2	0.05	0.49	0.08	78.79	unclass. *Alphaproteobacteria*	unclass. *Alphaproteobacteria*	unclass. *Alphaproteobacteria*	*Alphaproteobacteria*	*Proteobacteria*	*Bacteria*
Otu2093	0	0.2	0.05	0.49	0.08	78.87	unclass. *Sphingobacteriales*	unclass. *Sphingobacteriales*	*Sphingobacteriales*	*Sphingobacteria*	*Bacteroidetes*	*Bacteria*
Otu2094	0	0.2	0.05	0.49	0.08	78.95	unclass. *Oceanospirillaceae*	*Oceanospirillaceae*	*Oceanospirillales*	*Gammaproteobacteria*	*Proteobacteria*	*Bacteria*

OTU	Av.A_i (*pH in situ*)	Av.A_i (pH 7.67)	Av.δ_i	Av.δ_i/SD	Av.δ_i %	∑Av.δ_i %	Genus	Family	Order	Class	Phylum	Domain
Otu2095	0	0.2	0.05	0.49	0.08	79.02	*Colwellia*	*Colwelliaceae*	*Alteromonadales*	*Gammaproteobacteria*	*Proteobacteria*	*Bacteria*
Otu2096	0	0.2	0.05	0.49	0.08	79.1	unclass. *Bacteria*	unclass. *Bacteria*	unclass. *Bacteria*	unclass. *Bacteria*	unclass. *Bacteria*	*Bacteria*
Otu2097	0	0.2	0.05	0.49	0.08	79.18	unclass. *OD1*	unclass. *OD1*	unclass. *OD1*	unclass. *OD1*	*OD1*	*Bacteria*
Otu0103	0	0.2	0.05	0.49	0.08	79.25	*Pseudomonas*	*Pseudomonadaceae*	*Pseudomonadales*	*Gammaproteobacteria*	*Proteobacteria*	*Bacteria*
Otu0228	0	0.2	0.05	0.49	0.08	79.33	*Jannaschia*	*Rhodobacteraceae*	*Rhodobacterales*	*Alphaproteobacteria*	*Proteobacteria*	*Bacteria*
Otu0297	0	0.2	0.05	0.49	0.08	79.41	*Lutibacter*	*Flavobacteriaceae*	*Flavobacteriales*	*Flavobacteria*	*Bacteroidetes*	*Bacteria*
Otu0299	0	0.2	0.05	0.49	0.08	79.48	unclass. *Rhodobacteraceae*	*Rhodobacteraceae*	*Rhodobacterales*	*Alphaproteobacteria*	*Proteobacteria*	*Bacteria*
Otu0357	0	0.2	0.05	0.49	0.08	79.56	*Sphingopyxis*	*Sphingomonadaceae*	*Sphingomonadales*	*Alphaproteobacteria*	*Proteobacteria*	*Bacteria*
Otu0520	0	0.2	0.05	0.49	0.08	79.64	unclass. *Alphaproteobacteria*	unclass. *Alphaproteobacteria*	unclass. *Alphaproteobacteria*	*Alphaproteobacteria*	*Proteobacteria*	*Bacteria*
Otu0655	0	0.2	0.05	0.49	0.08	79.71	*Maricaulis*	*Hyphomonadaceae*	*Caulobacterales*	*Alphaproteobacteria*	*Proteobacteria*	*Bacteria*
Otu0802	0	0.2	0.05	0.49	0.08	79.79	unclass. *Flammeovirgaceae*	*Flammeovirgaceae*	*Sphingobacteriales*	*Sphingobacteria*	*Bacteroidetes*	*Bacteria*
Otu0807	0	0.2	0.05	0.49	0.08	79.87	unclass. *Bacteroidetes*	unclass. *Bacteroidetes*	unclass. *Bacteroidetes*	unclass. *Bacteroidetes*	*Bacteroidetes*	*Bacteria*
Otu1234	0	0.2	0.05	0.49	0.08	79.94	unclass. *Oceanospirillaceae*	*Oceanospirillaceae*	*Oceanospirillales*	*Gammaproteobacteria*	*Proteobacteria*	*Bacteria*
Otu1759	0	0.2	0.05	0.49	0.08	80.02	unclass. *Campylobacterales*	unclass. *Campylobacterales*	*Campylobacterales*	*Epsilonproteobacteria*	*Proteobacteria*	*Bacteria*
Otu1768	0	0.2	0.05	0.49	0.08	80.1	unclass. *OD1*	unclass. *OD1*	unclass. *OD1*	unclass. *OD1*	*OD1*	*Bacteria*
Otu1869	0	0.2	0.05	0.49	0.08	80.17	unclass. *Bacteroidetes*	unclass. *Bacteroidetes*	unclass. *Bacteroidetes*	unclass. *Bacteroidetes*	*Bacteroidetes*	*Bacteria*
Otu1884	0	0.2	0.05	0.49	0.08	80.25	unclass. *Bacteria*	unclass. *Bacteria*	unclass. *Bacteria*	unclass. *Bacteria*	unclass. *Bacteria*	*Bacteria*
Otu1969	0	0.2	0.05	0.49	0.08	80.33	unclass. *Bacteria*	unclass. *Bacteria*	unclass. *Bacteria*	unclass. *Bacteria*	unclass. *Bacteria*	*Bacteria*
Otu1970	0	0.2	0.05	0.49	0.08	80.4	*Desulfonema*	*Desulfobacteraceae*	*Desulfobacterales*	*Deltaproteobacteria*	*Proteobacteria*	*Bacteria*
Otu1971	0	0.2	0.05	0.49	0.08	80.48	unclass. *Flavobacteriales*	unclass. *Flavobacteriales*	*Flavobacteriales*	*Flavobacteria*	*Bacteroidetes*	*Bacteria*
Otu1972	0	0.2	0.05	0.49	0.08	80.56	unclass. *Flavobacteriaceae*	*Flavobacteriaceae*	*Flavobacteriales*	*Flavobacteria*	*Bacteroidetes*	*Bacteria*
Otu1973	0	0.2	0.05	0.49	0.08	80.63	unclass. *Flavobacteriales*	unclass. *Flavobacteriales*	*Flavobacteriales*	*Flavobacteria*	*Bacteroidetes*	*Bacteria*
Otu1974	0	0.2	0.05	0.49	0.08	80.71	*Oleispira*	*Oceanospirillaceae*	*Oceanospirillales*	*Gammaproteobacteria*	*Proteobacteria*	*Bacteria*
Otu1975	0	0.2	0.05	0.49	0.08	80.79	unclass. *Gammaproteobacteria*	unclass. *Gammaproteobacteria*	unclass. *Gammaproteobacteria*	*Gammaproteobacteria*	*Proteobacteria*	*Bacteria*
Otu1976	0	0.2	0.05	0.49	0.08	80.86	*Pelagibacter*	SAR11-clade	*Rickettsiales*	*Alphaproteobacteria*	*Proteobacteria*	*Bacteria*
Otu1977	0	0.2	0.05	0.49	0.08	80.94	unclass. *Bacteria*	unclass. *Bacteria*	unclass. *Bacteria*	unclass. *Bacteria*	unclass. *Bacteria*	*Bacteria*
Otu1978	0	0.2	0.05	0.49	0.08	81.02	unclass. *Bacteroidetes*	unclass. *Bacteroidetes*	unclass. *Bacteroidetes*	unclass. *Bacteroidetes*	*Bacteroidetes*	*Bacteria*

OTU	Av.A_i (*pH in situ*)	Av.A_i (pH 7.67)	Av.δ_i	Av.δ_i/SD	Av.δ_i %	∑Av.δ_i %	Genus	Family	Order	Class	Phylum	Domain
Otu1979	0	0.2	0.05	0.49	0.08	81.09	*Pelagibacter*	SAR11-clade	*Rickettsiales*	*Alphaproteobacteria*	*Proteobacteria*	*Bacteria*
Otu1981	0	0.2	0.05	0.49	0.08	81.17	unclass. *Bacteria*	unclass. *Bacteria*	unclass. *Bacteria*	unclass. *Bacteria*	unclass. *Bacteria*	*Bacteria*
Otu1982	0	0.2	0.05	0.49	0.08	81.25	unclass. *Gammaproteobacteria*	unclass. *Gammaproteobacteria*	unclass. *Gammaproteobacteria*	*Gammaproteobacteria*	*Proteobacteria*	*Bacteria*
Otu1983	0	0.2	0.05	0.49	0.08	81.32	unclass. *Gammaproteobacteria*	unclass. *Gammaproteobacteria*	unclass. *Gammaproteobacteria*	*Gammaproteobacteria*	*Proteobacteria*	*Bacteria*
Otu1984	0	0.2	0.05	0.49	0.08	81.4	unclass. *Bacteria*	unclass. *Bacteria*	unclass. *Bacteria*	unclass. *Bacteria*	unclass. *Bacteria*	*Bacteria*
Otu1985	0	0.2	0.05	0.49	0.08	81.48	unclass. *Bacteria*	unclass. *Bacteria*	unclass. *Bacteria*	unclass. *Bacteria*	unclass. *Bacteria*	*Bacteria*
Otu1986	0	0.2	0.05	0.49	0.08	81.55	unclass. *Bacteria*	unclass. *Bacteria*	unclass. *Bacteria*	unclass. *Bacteria*	unclass. *Bacteria*	*Bacteria*
Otu1987	0	0.2	0.05	0.49	0.08	81.63	unclass. *Gammaproteobacteria*	unclass. *Gammaproteobacteria*	unclass. *Gammaproteobacteria*	*Gammaproteobacteria*	*Proteobacteria*	*Bacteria*
Otu1988	0	0.2	0.05	0.49	0.08	81.71	*Salinisphaera*	*Salinisphaeraceae*	*Salinisphaerales*	*Gammaproteobacteria*	*Proteobacteria*	*Bacteria*
Otu1989	0	0.2	0.05	0.49	0.08	81.78	*Desulfocapsa*	*Desulfobulbaceae*	*Desulfobacterales*	*Deltaproteobacteria*	*Proteobacteria*	*Bacteria*
Otu1991	0	0.2	0.05	0.49	0.08	81.86	unclass. *Saprospiraceae*	*Saprospiraceae*	*Sphingobacteriales*	*Sphingobacteria*	*Bacteroidetes*	*Bacteria*
Otu1993	0	0.2	0.05	0.49	0.08	81.94	unclass. *Bacteria*	unclass. *Bacteria*	unclass. *Bacteria*	unclass. *Bacteria*	unclass. *Bacteria*	*Bacteria*
Otu1994	0	0.2	0.05	0.49	0.08	82.01	unclass. *Rhodobacteraceae*	*Rhodobacteraceae*	*Rhodobacterales*	*Alphaproteobacteria*	*Proteobacteria*	*Bacteria*
Otu1995	0	0.2	0.05	0.49	0.08	82.09	unclass. *Rhodobacteraceae*	*Rhodobacteraceae*	*Rhodobacterales*	*Alphaproteobacteria*	*Proteobacteria*	*Bacteria*
Otu1998	0	0.2	0.05	0.49	0.08	82.17	*Polaribacter*	*Flavobacteriaceae*	*Flavobacteriales*	*Flavobacteria*	*Bacteroidetes*	*Bacteria*
Otu1999	0	0.2	0.05	0.49	0.08	82.24	unclass. *Burkholderiales*	unclass. *Burkholderiales*	*Burkholderiales*	*Betaproteobacteria*	*Proteobacteria*	*Bacteria*
Otu2000	0	0.2	0.05	0.49	0.08	82.32	unclass. *Desulfuromonadales*	unclass. *Desulfuromonadales*	*Desulfuromonadales*	*Deltaproteobacteria*	*Proteobacteria*	*Bacteria*
Otu2001	0	0.2	0.05	0.49	0.08	82.4	unclass. *Flavobacteriaceae*	*Flavobacteriaceae*	*Flavobacteriales*	*Flavobacteria*	*Bacteroidetes*	*Bacteria*
Otu2002	0	0.2	0.05	0.49	0.08	82.47	*Colwellia*	*Colwelliaceae*	*Alteromonadales*	*Gammaproteobacteria*	*Proteobacteria*	*Bacteria*
Otu2003	0	0.2	0.05	0.49	0.08	82.55	*Jannaschia*	*Rhodobacteraceae*	*Rhodobacterales*	*Alphaproteobacteria*	*Proteobacteria*	*Bacteria*
Otu2004	0	0.2	0.05	0.49	0.08	82.63	unclass. *Bacteria*	unclass. *Bacteria*	unclass. *Bacteria*	unclass. *Bacteria*	unclass. *Bacteria*	*Bacteria*
Otu2005	0	0.2	0.05	0.49	0.08	82.7	unclass. *OD1*	unclass. *OD1*	unclass. *OD1*	unclass. *OD1*	*OD1*	*Bacteria*
Otu2006	0	0.2	0.05	0.49	0.08	82.78	unclass. *Actinomycetales*	unclass. *Actinomycetales*	*Actinomycetales*	*Actinobacteria*	*Actinobacteria*	*Bacteria*
Otu2007	0	0.2	0.05	0.49	0.08	82.86	unclass. *Gammaproteobacteria*	unclass. *Gammaproteobacteria*	unclass. *Gammaproteobacteria*	*Gammaproteobacteria*	*Proteobacteria*	*Bacteria*
Otu2008	0	0.2	0.05	0.49	0.08	82.93	unclass. *Flammeovirgaceae*	*Flammeovirgaceae*	*Sphingobacteriales*	*Sphingobacteria*	*Bacteroidetes*	*Bacteria*
Otu2009	0	0.2	0.05	0.49	0.08	83.01	*Polaribacter*	*Flavobacteriaceae*	*Flavobacteriales*	*Flavobacteria*	*Bacteroidetes*	*Bacteria*
Otu2010	0	0.2	0.05	0.49	0.08	83.09	unclass. *Bacteroidetes*	unclass. *Bacteroidetes*	unclass. *Bacteroidetes*	unclass. *Bacteroidetes*	*Bacteroidetes*	*Bacteria*

OTU	Av.A$_i$ (*pH in situ*)	Av.A$_i$ (pH 7.67)	Av.δ$_i$	Av.δ$_i$/SD	Av.δ$_i$ %	∑Av.δ$_i$ %	Genus	Family	Order	Class	Phylum	Domain
Otu0142	0.2	0	0.05	0.49	0.08	83.16	unclass. *Gammaproteobacteria*	unclass. *Gammaproteobacteria*	unclass. *Gammaproteobacteria*	*Gammaproteobacteria*	*Proteobacteria*	*Bacteria*
Otu0144	0.2	0	0.05	0.49	0.08	83.24	*Lewinella*	*Saprospiraceae*	*Sphingobacteriales*	*Sphingobacteria*	*Bacteroidetes*	*Bacteria*
Otu0211	0.2	0	0.05	0.49	0.08	83.31	unclass. *Alphaproteobacteria*	unclass. *Alphaproteobacteria*	unclass. *Alphaproteobacteria*	*Alphaproteobacteria*	*Proteobacteria*	*Bacteria*
Otu0308	0.2	0	0.05	0.49	0.08	83.39	unclass. *Gammaproteobacteria*	unclass. *Gammaproteobacteria*	unclass. *Gammaproteobacteria*	*Gammaproteobacteria*	*Proteobacteria*	*Bacteria*
Otu0321	0.2	0	0.05	0.49	0.08	83.47	unclass. *Alphaproteobacteria*	unclass. *Alphaproteobacteria*	unclass. *Alphaproteobacteria*	*Alphaproteobacteria*	*Proteobacteria*	*Bacteria*
Otu0361	0.2	0	0.05	0.49	0.08	83.54	unclass. *Flavobacteriaceae*	*Flavobacteriaceae*	*Flavobacteriales*	*Flavobacteria*	*Bacteroidetes*	*Bacteria*
Otu0389	0.2	0	0.05	0.49	0.08	83.62	unclass. *Flavobacteriaceae*	*Flavobacteriaceae*	*Flavobacteriales*	*Flavobacteria*	*Bacteroidetes*	*Bacteria*
Otu0461	0.2	0	0.05	0.49	0.08	83.7	unclass. *Microbacteriaceae*	*Microbacteriaceae*	*Actinomycetales*	*Actinobacteria*	*Actinobacteria*	*Bacteria*
Otu0717	0.2	0	0.05	0.49	0.08	83.77	unclass. *Rhodobacteraceae*	*Rhodobacteraceae*	*Rhodobacterales*	*Alphaproteobacteria*	*Proteobacteria*	*Bacteria*
Otu0873	0.2	0	0.05	0.49	0.08	83.85	unclass. *Gammaproteobacteria*	unclass. *Gammaproteobacteria*	unclass. *Gammaproteobacteria*	*Gammaproteobacteria*	*Proteobacteria*	*Bacteria*
Otu1004	0.2	0	0.05	0.49	0.08	83.92	unclass. *Gammaproteobacteria*	unclass. *Gammaproteobacteria*	unclass. *Gammaproteobacteria*	*Gammaproteobacteria*	*Proteobacteria*	*Bacteria*
Otu1229	0.2	0	0.05	0.49	0.08	84	*Haliea*	*Alteromonadaceae*	*Alteromonadales*	*Gammaproteobacteria*	*Proteobacteria*	*Bacteria*
Otu1362	0.2	0	0.05	0.49	0.08	84.08	unclass. *Bacteria*	unclass. *Bacteria*	unclass. *Bacteria*	unclass. *Bacteria*	unclass. *Bacteria*	*Bacteria*
Otu1683	0.2	0	0.05	0.49	0.08	84.15	unclass. *Gammaproteobacteria*	unclass. *Gammaproteobacteria*	unclass. *Gammaproteobacteria*	*Gammaproteobacteria*	*Proteobacteria*	*Bacteria*
Otu1687	0.2	0	0.05	0.49	0.08	84.23	*Haliea*	*Alteromonadaceae*	*Alteromonadales*	*Gammaproteobacteria*	*Proteobacteria*	*Bacteria*
Otu1699	0.2	0	0.05	0.49	0.08	84.3	*Flavobacterium*	*Flavobacteriaceae*	*Flavobacteriales*	*Flavobacteria*	*Bacteroidetes*	*Bacteria*
Otu1703	0.2	0	0.05	0.49	0.08	84.38	unclass. *Proteobacteria*	unclass. *Proteobacteria*	unclass. *Proteobacteria*	unclass. *Proteobacteria*	*Proteobacteria*	*Bacteria*
Otu1735	0.2	0	0.05	0.49	0.08	84.46	*Sulfurovum*	*Helicobacteraceae*	*Campylobacterales*	*Epsilonproteobacteria*	*Proteobacteria*	*Bacteria*
Otu1748	0.2	0	0.05	0.49	0.08	84.53	unclass. *Epsilonproteobacteria*	unclass. *Epsilonproteobacteria*	unclass. *Epsilonproteobacteria*	*Epsilonproteobacteria*	*Proteobacteria*	*Bacteria*
Otu1817	0.2	0	0.05	0.49	0.08	84.61	unclass. *Flavobacteriaceae*	*Flavobacteriaceae*	*Flavobacteriales*	*Flavobacteria*	*Bacteroidetes*	*Bacteria*
Otu1826	0.2	0	0.05	0.49	0.08	84.68	unclass. *Sphingobacteriales*	unclass. *Sphingobacteriales*	*Sphingobacteriales*	*Sphingobacteria*	*Bacteroidetes*	*Bacteria*
Otu1829	0.2	0	0.05	0.49	0.08	84.76	unclass. *Bacteria*	unclass. *Bacteria*	unclass. *Bacteria*	unclass. *Bacteria*	unclass. *Bacteria*	*Bacteria*
Otu1830	0.2	0	0.05	0.49	0.08	84.84	unclass. *Flavobacteriaceae*	*Flavobacteriaceae*	*Flavobacteriales*	*Flavobacteria*	*Bacteroidetes*	*Bacteria*
Otu1831	0.2	0	0.05	0.49	0.08	84.91	unclass. *Proteobacteria*	unclass. *Proteobacteria*	unclass. *Proteobacteria*	unclass. *Proteobacteria*	*Proteobacteria*	*Bacteria*
Otu1832	0.2	0	0.05	0.49	0.08	84.99	*Neptunomonas*	*Oceanospirillaceae*	*Oceanospirillales*	*Gammaproteobacteria*	*Proteobacteria*	*Bacteria*
Otu1835	0.2	0	0.05	0.49	0.08	85.07	unclass. *Gammaproteobacteria*	unclass. *Gammaproteobacteria*	unclass. *Gammaproteobacteria*	*Gammaproteobacteria*	*Proteobacteria*	*Bacteria*
Otu1838	0.2	0	0.05	0.49	0.08	85.14	unclass. *Bacteria*	unclass. *Bacteria*	unclass. *Bacteria*	unclass. *Bacteria*	unclass. *Bacteria*	*Bacteria*

OTU	Av.A_i (*pH in situ*)	Av.A_i (pH 7.67)	Av.δ_i	Av.δ_i/SD	Av.δ_i %	∑Av.δ_i %	Genus	Family	Order	Class	Phylum	Domain
Otu1839	0.2	0	0.05	0.49	0.08	85.22	unclass. *Flavobacteriaceae*	*Flavobacteriaceae*	*Flavobacteriales*	*Flavobacteria*	*Bacteroidetes*	*Bacteria*
Otu1840	0.2	0	0.05	0.49	0.08	85.29	unclass. *Desulfobulbaceae*	*Desulfobulbaceae*	*Desulfobacterales*	*Deltaproteobacteria*	*Proteobacteria*	*Bacteria*
Otu1843	0.2	0	0.05	0.49	0.08	85.37	unclass. *Bacteria*	unclass. *Bacteria*	unclass. *Bacteria*	unclass. *Bacteria*	unclass. *Bacteria*	*Bacteria*
Otu1844	0.2	0	0.05	0.49	0.08	85.45	unclass. *Bacteroidetes*	unclass. *Bacteroidetes*	unclass. *Bacteroidetes*	unclass. *Bacteroidetes*	*Bacteroidetes*	*Bacteria*
Otu1845	0.2	0	0.05	0.49	0.08	85.52	unclass. *Sphingobacteriales*	unclass. *Sphingobacteriales*	*Sphingobacteriales*	*Sphingobacteria*	*Bacteroidetes*	*Bacteria*
Otu1846	0.2	0	0.05	0.49	0.08	85.6	unclass. *Flavobacteriaceae*	*Flavobacteriaceae*	*Flavobacteriales*	*Flavobacteria*	*Bacteroidetes*	*Bacteria*
Otu1847	0.2	0	0.05	0.49	0.08	85.67	unclass. *Gammaproteobacteria*	unclass. *Gammaproteobacteria*	unclass. *Gammaproteobacteria*	*Gammaproteobacteria*	*Proteobacteria*	*Bacteria*
Otu1849	0.2	0	0.05	0.49	0.08	85.75	unclass. *Alphaproteobacteria*	unclass. *Alphaproteobacteria*	unclass. *Alphaproteobacteria*	*Alphaproteobacteria*	*Proteobacteria*	*Bacteria*
Otu1850	0.2	0	0.05	0.49	0.08	85.83	*Reichenbachiella*	*Flammeovirgaceae*	*Sphingobacteriales*	*Sphingobacteria*	*Bacteroidetes*	*Bacteria*
Otu1852	0.2	0	0.05	0.49	0.08	85.9	unclass. *Acidobacteria_Gp22*	unclass. *Acidobacteria_Gp22*	unclass. *Acidobacteria_Gp22*	*Acidobacteria_Gp22*	*Acidobacteria*	*Bacteria*
Otu1853	0.2	0	0.05	0.49	0.08	85.98	unclass. *Bacteria*	unclass. *Bacteria*	unclass. *Bacteria*	unclass. *Bacteria*	unclass. *Bacteria*	*Bacteria*
Otu0006	0.2	0	0.05	0.49	0.08	86.05	*Polaribacter*	*Flavobacteriaceae*	*Flavobacteriales*	*Flavobacteria*	*Bacteroidetes*	*Bacteria*
Otu0014	0.2	0	0.05	0.49	0.08	86.13	*Reinekea*	*Oceanospirillaceae*	*Oceanospirillales*	*Gammaproteobacteria*	*Proteobacteria*	*Bacteria*
Otu0064	0.2	0	0.05	0.49	0.08	86.21	unclass. *Flavobacteriaceae*	*Flavobacteriaceae*	*Flavobacteriales*	*Flavobacteria*	*Bacteroidetes*	*Bacteria*
Otu0081	0.2	0	0.05	0.49	0.08	86.28	*Altererythrobacter*	*Erythrobacteraceae*	*Sphingomonadales*	*Alphaproteobacteria*	*Proteobacteria*	*Bacteria*
Otu0374	0.2	0	0.05	0.49	0.08	86.36	unclass. *Flavobacteriaceae*	*Flavobacteriaceae*	*Flavobacteriales*	*Flavobacteria*	*Bacteroidetes*	*Bacteria*
Otu0420	0.2	0	0.05	0.49	0.08	86.43	unclass. *Gammaproteobacteria*	unclass. *Gammaproteobacteria*	unclass. *Gammaproteobacteria*	*Gammaproteobacteria*	*Proteobacteria*	*Bacteria*
Otu0488	0.2	0	0.05	0.49	0.08	86.51	unclass. *Gammaproteobacteria*	unclass. *Gammaproteobacteria*	unclass. *Gammaproteobacteria*	*Gammaproteobacteria*	*Proteobacteria*	*Bacteria*
Otu0515	0.2	0	0.05	0.49	0.08	86.59	unclass. *Gammaproteobacteria*	unclass. *Gammaproteobacteria*	unclass. *Gammaproteobacteria*	*Gammaproteobacteria*	*Proteobacteria*	*Bacteria*
Otu0535	0.2	0	0.05	0.49	0.08	86.66	*Loktanella*	*Rhodobacteraceae*	*Rhodobacterales*	*Alphaproteobacteria*	*Proteobacteria*	*Bacteria*
Otu0710	0.2	0	0.05	0.49	0.08	86.74	unclass. *Gammaproteobacteria*	unclass. *Gammaproteobacteria*	unclass. *Gammaproteobacteria*	*Gammaproteobacteria*	*Proteobacteria*	*Bacteria*
Otu0791	0.2	0	0.05	0.49	0.08	86.81	*Thalassomonas*	*Colwelliaceae*	*Alteromonadales*	*Gammaproteobacteria*	*Proteobacteria*	*Bacteria*
Otu0870	0.2	0	0.05	0.49	0.08	86.89	unclass. *Gammaproteobacteria*	unclass. *Gammaproteobacteria*	unclass. *Gammaproteobacteria*	*Gammaproteobacteria*	*Proteobacteria*	*Bacteria*
Otu0892	0.2	0	0.05	0.49	0.08	86.97	unclass. *Bacteroidetes*	unclass. *Bacteroidetes*	unclass. *Bacteroidetes*	unclass. *Bacteroidetes*	*Bacteroidetes*	*Bacteria*
Otu0946	0.2	0	0.05	0.49	0.08	87.04	*Pelagibacter*	SAR11-clade	*Rickettsiales*	*Alphaproteobacteria*	*Proteobacteria*	*Bacteria*
Otu0949	0.2	0	0.05	0.49	0.08	87.12	unclass. *Gammaproteobacteria*	unclass. *Gammaproteobacteria*	unclass. *Gammaproteobacteria*	*Gammaproteobacteria*	*Proteobacteria*	*Bacteria*
Otu1126	0.2	0	0.05	0.49	0.08	87.19	unclass. *Bacteroidetes*	unclass. *Bacteroidetes*	unclass. *Bacteroidetes*	unclass. *Bacteroidetes*	*Bacteroidetes*	*Bacteria*

OTU	Av.A$_i$ (*pH in situ*)	Av.A$_i$ (pH 7.67)	Av.δ$_i$	Av.δ$_i$/SD	Av.δ$_i$ %	∑Av.δ$_i$ %	Genus	Family	Order	Class	Phylum	Domain
Otu1145	0.2	0	0.05	0.49	0.08	87.27	unclass. *Bacteria*	unclass. *Bacteria*	unclass. *Bacteria*	unclass. *Bacteria*	unclass. *Bacteria*	*Bacteria*
Otu1415	0.2	0	0.05	0.49	0.08	87.35	*Polaribacter*	*Flavobacteriaceae*	*Flavobacteriales*	*Flavobacteria*	*Bacteroidetes*	*Bacteria*
Otu1542	0.2	0	0.05	0.49	0.08	87.42	unclass. *Burkholderiales*	unclass. *Burkholderiales*	*Burkholderiales*	*Betaproteobacteria*	*Proteobacteria*	*Bacteria*
Otu1645	0.2	0	0.05	0.49	0.08	87.5	*Thalassomonas*	*Colwelliaceae*	*Alteromonadales*	*Gammaproteobacteria*	*Proteobacteria*	*Bacteria*
Otu1713	0.2	0	0.05	0.49	0.08	87.57	unclass. *Flavobacteriaceae*	*Flavobacteriaceae*	*Flavobacteriales*	*Flavobacteria*	*Bacteroidetes*	*Bacteria*
Otu1767	0.2	0	0.05	0.49	0.08	87.65	unclass. *Bacteria*	unclass. *Bacteria*	unclass. *Bacteria*	unclass. *Bacteria*	unclass. *Bacteria*	*Bacteria*
Otu1795	0.2	0	0.05	0.49	0.08	87.73	*Colwellia*	*Colwelliaceae*	*Alteromonadales*	*Gammaproteobacteria*	*Proteobacteria*	*Bacteria*
Otu1813	0.2	0	0.05	0.49	0.08	87.8	*Colwellia*	*Colwelliaceae*	*Alteromonadales*	*Gammaproteobacteria*	*Proteobacteria*	*Bacteria*
Otu1834	0.2	0	0.05	0.49	0.08	87.88	*Eudoraea*	*Flavobacteriaceae*	*Flavobacteriales*	*Flavobacteria*	*Bacteroidetes*	*Bacteria*
Otu1855	0.2	0	0.05	0.49	0.08	87.95	unclass. *Gammaproteobacteria*	unclass. *Gammaproteobacteria*	unclass. *Gammaproteobacteria*	*Gammaproteobacteria*	*Proteobacteria*	*Bacteria*
Otu1856	0.2	0	0.05	0.49	0.08	88.03	unclass. *Bacteria*	unclass. *Bacteria*	unclass. *Bacteria*	unclass. *Bacteria*	unclass. *Bacteria*	*Bacteria*
Otu1857	0.2	0	0.05	0.49	0.08	88.1	*Gaetbulibacter*	*Flavobacteriaceae*	*Flavobacteriales*	*Flavobacteria*	*Bacteroidetes*	*Bacteria*
Otu1858	0.2	0	0.05	0.49	0.08	88.18	*Glaciecola*	*Alteromonadaceae*	*Alteromonadales*	*Gammaproteobacteria*	*Proteobacteria*	*Bacteria*
Otu1859	0.2	0	0.05	0.49	0.08	88.26	*Ferrimonas*	*Ferrimonadaceae*	*Alteromonadales*	*Gammaproteobacteria*	*Proteobacteria*	*Bacteria*
Otu1860	0.2	0	0.05	0.49	0.08	88.33	unclass. *Alteromonadaceae*	*Alteromonadaceae*	*Alteromonadales*	*Gammaproteobacteria*	*Proteobacteria*	*Bacteria*
Otu1862	0.2	0	0.05	0.49	0.08	88.41	unclass. *Bacteroidetes*	unclass. *Bacteroidetes*	unclass. *Bacteroidetes*	unclass. *Bacteroidetes*	*Bacteroidetes*	*Bacteria*
Otu1863	0.2	0	0.05	0.49	0.08	88.48	unclass. *Desulfuromonadaceae*	*Desulfuromonadaceae*	*Desulfuromonadales*	*Deltaproteobacteria*	*Proteobacteria*	*Bacteria*
Otu1864	0.2	0	0.05	0.49	0.08	88.56	*Lewinella*	*Saprospiraceae*	*Sphingobacteriales*	*Sphingobacteria*	*Bacteroidetes*	*Bacteria*
Otu1866	0.2	0	0.05	0.49	0.08	88.64	*Algibacter*	*Flavobacteriaceae*	*Flavobacteriales*	*Flavobacteria*	*Bacteroidetes*	*Bacteria*
Otu1867	0.2	0	0.05	0.49	0.08	88.71	unclass. *Bacteria*	unclass. *Bacteria*	unclass. *Bacteria*	unclass. *Bacteria*	unclass. *Bacteria*	*Bacteria*
Otu1868	0.2	0	0.05	0.49	0.08	88.79	*Maribacter*	*Flavobacteriaceae*	*Flavobacteriales*	*Flavobacteria*	*Bacteroidetes*	*Bacteria*
Otu1870	0.2	0	0.05	0.49	0.08	88.86	unclass. *Gammaproteobacteria*	unclass. *Gammaproteobacteria*	unclass. *Gammaproteobacteria*	*Gammaproteobacteria*	*Proteobacteria*	*Bacteria*
Otu1871	0.2	0	0.05	0.49	0.08	88.94	unclass. *Flavobacteriaceae*	*Flavobacteriaceae*	*Flavobacteriales*	*Flavobacteria*	*Bacteroidetes*	*Bacteria*
Otu1872	0.2	0	0.05	0.49	0.08	89.02	unclass. *Domain*	unclass. *Domain*	unclass. *Domain*	unclass. *Domain*	unclass. *Domain*	unclass. *Domain*
Otu1874	0.2	0	0.05	0.49	0.08	89.09	unclass. *Gammaproteobacteria*	unclass. *Gammaproteobacteria*	unclass. *Gammaproteobacteria*	*Gammaproteobacteria*	*Proteobacteria*	*Bacteria*
Otu1876	0.2	0	0.05	0.49	0.08	89.17	*Glaciecola*	*Alteromonadaceae*	*Alteromonadales*	*Gammaproteobacteria*	*Proteobacteria*	*Bacteria*
Otu1877	0.2	0	0.05	0.49	0.08	89.24	unclass. *Bacteria*	unclass. *Bacteria*	unclass. *Bacteria*	unclass. *Bacteria*	unclass. *Bacteria*	*Bacteria*

OTU	Av.A_i (*pH in situ*)	Av.A_i (pH 7.67)	Av.δ_i	Av.δ_i/SD	Av.δ_i %	$\sum$Av.δ_i %	Genus	Family	Order	Class	Phylum	Domain
Otu1878	0.2	0	0.05	0.49	0.08	89.32	unclass. *Bacteria*	unclass. *Bacteria*	unclass. *Bacteria*	unclass. *Bacteria*	unclass. *Bacteria*	*Bacteria*
Otu1879	0.2	0	0.05	0.49	0.08	89.4	unclass. *Flavobacteriaceae*	*Flavobacteriaceae*	*Flavobacteriales*	*Flavobacteria*	*Bacteroidetes*	*Bacteria*
Otu0007	0.2	0	0.05	0.49	0.08	89.47	unclass. *Flavobacteriales*	unclass. *Flavobacteriales*	*Flavobacteriales*	*Flavobacteria*	*Bacteroidetes*	*Bacteria*
Otu0048	0.2	0	0.05	0.49	0.08	89.55	unclass. *Flavobacteriaceae*	*Flavobacteriaceae*	*Flavobacteriales*	*Flavobacteria*	*Bacteroidetes*	*Bacteria*
Otu0131	0.2	0	0.05	0.49	0.08	89.62	*Roseivirga*	*Flammeovirgaceae*	*Sphingobacteriales*	*Sphingobacteria*	*Bacteroidetes*	*Bacteria*
Otu0180	0.2	0	0.05	0.49	0.08	89.7	*Reichenbachiella*	*Flammeovirgaceae*	*Sphingobacteriales*	*Sphingobacteria*	*Bacteroidetes*	*Bacteria*
Otu0222	0.2	0	0.05	0.49	0.08	89.78	*Haliea*	*Alteromonadaceae*	*Alteromonadales*	*Gammaproteobacteria*	*Proteobacteria*	*Bacteria*
Otu0253	0.2	0	0.05	0.49	0.08	89.85	unclass. *Saprospiraceae*	*Saprospiraceae*	*Sphingobacteriales*	*Sphingobacteria*	*Bacteroidetes*	*Bacteria*
Otu0265	0.2	0	0.05	0.49	0.08	89.93	unclass. *Bacteria*	unclass. *Bacteria*	unclass. *Bacteria*	unclass. *Bacteria*	unclass. *Bacteria*	*Bacteria*
Otu0323	0.2	0	0.05	0.49	0.08	90	unclass. *Gammaproteobacteria_incertae_sedis*	*Gammaproteobacteria_incertae_sedis*	*Gammaproteobacteria_order_incertae_sedis*	*Gammaproteobacteria*	*Proteobacteria*	*Bacteria*
Winter 'serial dilution' (average dissimilarity: 56.6%)												
Otu0117	0.88	14.6	9.3	6.9	16.44	16.44	*Arcobacter*	*Campylobacteraceae*	*Campylobacterales*	*Epsilonproteobacteria*	*Proteobacteria*	*Bacteria*
Otu1748	0	6.37	4.31	5.61	7.61	24.05	unclass. *Epsilonproteobacteria*	unclass. *Epsilonproteobacteria*	unclass. *Epsilonproteobacteria*	*Epsilonproteobacteria*	*Proteobacteria*	*Bacteria*
Otu0055	9.17	4.54	3.77	1.75	6.67	30.72	*Colwellia*	*Colwelliaceae*	*Alteromonadales*	*Gammaproteobacteria*	*Proteobacteria*	*Bacteria*
Otu0686	0.4	5.23	3.25	2.1	5.75	36.47	*Marinomonas*	*Oceanospirillaceae*	*Oceanospirillales*	*Gammaproteobacteria*	*Proteobacteria*	*Bacteria*
Otu0132	8.64	4.05	3.11	2.16	5.5	41.97	unclass. *Alteromonadales*	unclass. *Alteromonadales*	*Alteromonadales*	*Gammaproteobacteria*	*Proteobacteria*	*Bacteria*
Otu0106	12.27	7.72	3.1	1.86	5.48	47.45	*Marinomonas*	*Oceanospirillaceae*	*Oceanospirillales*	*Gammaproteobacteria*	*Proteobacteria*	*Bacteria*
Otu0234	0.2	4.68	3.03	3.7	5.36	52.81	*Arcobacter*	*Campylobacteraceae*	*Campylobacterales*	*Epsilonproteobacteria*	*Proteobacteria*	*Bacteria*
Otu0160	5.46	1.2	2.98	1.78	5.27	58.09	*Colwellia*	*Colwelliaceae*	*Alteromonadales*	*Gammaproteobacteria*	*Proteobacteria*	*Bacteria*
Otu0123	5.62	2.75	2.21	1.05	3.91	62	unclass. *Gammaproteobacteria*	unclass. *Gammaproteobacteria*	unclass. *Gammaproteobacteria*	*Gammaproteobacteria*	*Proteobacteria*	*Bacteria*
Otu0471	4.28	1.03	2.19	2.03	3.88	65.88	*Oceaniserpentilla*	*Oceanospirillaceae*	*Oceanospirillales*	*Gammaproteobacteria*	*Proteobacteria*	*Bacteria*
Otu1778	0.2	2.28	1.44	0.89	2.55	68.43	*Arcobacter*	*Campylobacteraceae*	*Campylobacterales*	*Epsilonproteobacteria*	*Proteobacteria*	*Bacteria*
Otu0035	2.68	1.23	1.09	0.92	1.92	70.35	*Colwellia*	*Colwelliaceae*	*Alteromonadales*	*Gammaproteobacteria*	*Proteobacteria*	*Bacteria*
Otu1780	0.77	1.81	0.98	1.95	1.74	72.09	unclass. *Oceanospirillales*	unclass. *Oceanospirillales*	*Oceanospirillales*	*Gammaproteobacteria*	*Proteobacteria*	*Bacteria*
Otu1801	0	1.31	0.89	4.41	1.57	73.66	*Arcobacter*	*Campylobacteraceae*	*Campylobacterales*	*Epsilonproteobacteria*	*Proteobacteria*	*Bacteria*
Otu0460	0	1.08	0.74	1.64	1.3	74.96	*Arcobacter*	*Campylobacteraceae*	*Campylobacterales*	*Epsilonproteobacteria*	*Proteobacteria*	*Bacteria*
Otu1777	5.49	5.32	0.69	1.56	1.22	76.18	*Oceaniserpentilla*	*Oceanospirillaceae*	*Oceanospirillales*	*Gammaproteobacteria*	*Proteobacteria*	*Bacteria*

OTU	Av.A_i (*pH in situ*)	Av.A_i (pH 7.67)	Av.δ_i	Av.δ_i/SD	Av.δ_i %	$\sum$Av.δ_i %	Genus	Family	Order	Class	Phylum	Domain
Otu0542	0.91	0	0.62	1.18	1.09	77.28	unclass. *Oceanospirillaceae*	*Oceanospirillaceae*	*Oceanospirillales*	*Gammaproteobacteria*	*Proteobacteria*	*Bacteria*
Otu0571	1.95	1.28	0.59	1.19	1.04	78.31	*Marinomonas*	*Oceanospirillaceae*	*Oceanospirillales*	*Gammaproteobacteria*	*Proteobacteria*	*Bacteria*
Otu1781	1.09	0.48	0.59	1.37	1.04	79.35	*Oceaniserpentilla*	*Oceanospirillaceae*	*Oceanospirillales*	*Gammaproteobacteria*	*Proteobacteria*	*Bacteria*
Otu0209	1.54	1.38	0.58	1.4	1.03	80.38	unclass. *Oceanospirillales*	unclass. *Oceanospirillales*	*Oceanospirillales*	*Gammaproteobacteria*	*Proteobacteria*	*Bacteria*
Otu0010	0.97	0.2	0.57	1.51	1.01	81.39	unclass. *Flavobacteriaceae*	*Flavobacteriaceae*	*Flavobacteriales*	*Flavobacteria*	*Bacteroidetes*	*Bacteria*
Otu0140	1.08	0.68	0.5	1.19	0.88	82.27	*Oleispira*	*Oceanospirillaceae*	*Oceanospirillales*	*Gammaproteobacteria*	*Proteobacteria*	*Bacteria*
Otu1027	0	0.69	0.48	0.8	0.84	83.11	unclass. *Epsilonproteobacteria*	unclass. *Epsilonproteobacteria*	unclass. *Epsilonproteobacteria*	*Epsilonproteobacteria*	*Proteobacteria*	*Bacteria*
Otu1804	0	0.6	0.41	1.2	0.72	83.83	unclass. *Epsilonproteobacteria*	unclass. *Epsilonproteobacteria*	unclass. *Epsilonproteobacteria*	*Epsilonproteobacteria*	*Proteobacteria*	*Bacteria*
Otu0176	0.6	0.2	0.38	1.1	0.67	84.5	*Colwellia*	*Colwelliaceae*	*Alteromonadales*	*Gammaproteobacteria*	*Proteobacteria*	*Bacteria*
Otu1783	0.6	0.2	0.38	1.1	0.67	85.17	*Marinomonas*	*Oceanospirillaceae*	*Oceanospirillales*	*Gammaproteobacteria*	*Proteobacteria*	*Bacteria*
Otu0533	0.48	0	0.33	0.78	0.59	85.76	*Nisaea*	*Rhodospirillaceae*	*Rhodospirillales*	*Alphaproteobacteria*	*Proteobacteria*	*Bacteria*
Otu1084	0.48	0	0.32	0.78	0.57	86.33	*Marinomonas*	*Oceanospirillaceae*	*Oceanospirillales*	*Gammaproteobacteria*	*Proteobacteria*	*Bacteria*
Otu1231	0.48	0	0.32	0.78	0.57	86.9	unclass. *Gammaproteobacteria*	unclass. *Gammaproteobacteria*	unclass. *Gammaproteobacteria*	*Gammaproteobacteria*	*Proteobacteria*	*Bacteria*
Otu1079	0.4	0	0.27	0.8	0.48	87.38	*Marinomonas*	*Oceanospirillaceae*	*Oceanospirillales*	*Gammaproteobacteria*	*Proteobacteria*	*Bacteria*
Otu1802	0	0.4	0.27	0.8	0.48	87.86	*Oceaniserpentilla*	*Oceanospirillaceae*	*Oceanospirillales*	*Gammaproteobacteria*	*Proteobacteria*	*Bacteria*
Otu1065	0.4	0	0.27	0.8	0.48	88.34	*Marinomonas*	*Oceanospirillaceae*	*Oceanospirillales*	*Gammaproteobacteria*	*Proteobacteria*	*Bacteria*
Otu0110	0	0.4	0.26	0.49	0.47	88.81	unclass. *Rhodobacteraceae*	*Rhodobacteraceae*	*Rhodobacterales*	*Alphaproteobacteria*	*Proteobacteria*	*Bacteria*
Otu1807	0	0.4	0.26	0.8	0.47	89.27	*Arcobacter*	*Campylobacteraceae*	*Campylobacterales*	*Epsilonproteobacteria*	*Proteobacteria*	*Bacteria*
Otu1782	0.2	0.2	0.21	0.67	0.37	89.65	*Oceaniserpentilla*	*Oceanospirillaceae*	*Oceanospirillales*	*Gammaproteobacteria*	*Proteobacteria*	*Bacteria*
Otu0204	0	0.28	0.2	0.49	0.35	89.99	unclass. *Colwelliaceae*	*Colwelliaceae*	*Alteromonadales*	*Gammaproteobacteria*	*Proteobacteria*	*Bacteria*
Otu0115	0.28	0	0.18	0.49	0.33	90.32	*Glaciecola*	*Alteromonadaceae*	*Alteromonadales*	*Gammaproteobacteria*	*Proteobacteria*	*Bacteria*
